# Biologistics—Diffusion coefficients for complete proteome of *Escherichia coli*

**DOI:** 10.1093/bioinformatics/bts537

**Published:** 2012-08-31

**Authors:** Tomasz Kalwarczyk, Marcin Tabaka, Robert Holyst

**Affiliations:** Institute of Physical Chemistry, Polish Academy of Sciences, Kasprzaka 44/52, 01-224 Warsaw, Poland

## Abstract

**Motivation:** Biologistics provides data for quantitative analysis of transport (diffusion) processes and their spatio-temporal correlations in cells. Mobility of proteins is one of the few parameters necessary to describe reaction rates for gene regulation. Although understanding of diffusion-limited biochemical reactions *in vivo* requires mobility data for the largest possible number of proteins in their native forms, currently, there is no database that would contain the complete information about the diffusion coefficients (DCs) of proteins in a given cell type.

**Results:** We demonstrate a method for the determination of *in vivo* DCs for any molecule—regardless of its molecular weight, size and structure—in any type of cell. We exemplify the method with the database of *in vivo* DC for all proteins (4302 records) from the proteome of K12 strain of *Escherichia coli*, together with examples of DC of amino acids, sugars, RNA and DNA. The database follows from the scale-dependent viscosity reference curve (sdVRC). Construction of sdVRC for prokaryotic or eukaryotic cell requires ~20 *in vivo* measurements using techniques such as fluorescence correlation spectroscopy (FCS), fluorescence recovery after photobleaching (FRAP), nuclear magnetic resonance (NMR) or particle tracking. The shape of the sdVRC would be different for each organism, but the mathematical form of the curve remains the same. The presented method has a high predictive power, as the measurements of DCs of several inert, properly chosen probes in a single cell type allows to determine the DCs of thousands of proteins. Additionally, obtained mobility data allow quantitative study of biochemical interactions *in vivo*.

**Contact:** rholyst@ichf.edu.pl

**Supplementary information:** Supplementary data are available at *Bioinformatics* Online.

## 1 INTRODUCTION

Biologistics and biochemistry in a crowded environment are two emerging interdisciplinary fields of science. They provide quantitative analysis of transport of proteins and their spatio-temporal correlations involved in gene expression and regulation. According to the current state-of-the-art theory of gene expression (activation or repression) in bacteria (Elf *et al.*, 2007; Li *et al.*, 2009), mobility of proteins is one of the few parameters necessary to describe reaction rates of gene regulation. The mobility is understood as a three-dimensional diffusion or one-dimensional sliding along DNA (for prokaryotes and eukaryotes), or by velocity of molecular motors (in eukaryotic cells). Understanding of diffusion-limited biochemical reactions requires accurate *in vivo* mobility data for the largest possible number of proteins in their native forms. The three-dimensional diffusion of different types of macromolecules in the cytoplasm of *Escherichia coli* has been experimentally studied in several cases (Bakshi *et al.*, 2012; Campbell and Mullins, 2007; Cluzel *et al.*, 2000; Derman *et al.*, 2008; Elowitz *et al.*, 1999; English *et al.*, 2011; Golding and Cox, 2004; Jasnin *et al.*, 2008; Konopka *et al.*, 2006; Kumar *et al.*, 2010; Mika *et al.*, 2010; Mullineaux *et al.*, 2006; Nenninger *et al.*, 2010; Slade *et al.*, 2009; van den Bogaart *et al.*, 2007), but experimental determination of the mobility of all proteins is technically an impossible task because of their large number in a given cell. For example, the proteome of the K12 strain of *E. coli* (Blattner *et al.*, 1997) contains more than 4300 proteins. Moreover, most of the recent studies concern measurements mainly performed with the use of green fluorescent protein (GFP) (Elowitz *et al.*, 1999; Konopka *et al.*, 2006; Kumar *et al.*, 2010; Mika *et al.*, 2010; Nenninger *et al.*, 2010; Slade *et al.*, 2009; van den Bogaart *et al.*, 2007) or GFP fusion proteins (Jennifer *et al.*, 2001).

Attempts to study the diffusion of many proteins simultaneously, under conditions resembling the interior of the cells, were performed *in silico* by McGuffee and Elcock (2010). Computational methods, however, have limitations arising from the speed and capacity of computing hardware and small number of interacting proteins in the system (~50 different types of proteins) (McGuffee and Elcock, 2010). An alternative approach is the quantitative analysis of available literature data. Mika and Poolman (2011) gathered literature data of diffusion coefficients (DCs) of ~20 different types of proteins in *E. coli* and proposed a power law dependence of the DC on the molecular weight of proteins. This power law, however (Mika and Poolman, 2011), can be applied only for the proteins in a narrow range of molecular weights, i.e. between 20 and 30 kDa.

In this work, we present a method for predictions of the DCs of proteins for the proteome of any cell. We collected all available literature data (Bakshi *et al.*, 2012; Campbell and Mullins, 2007; Cluzel *et al.*, 2000; Derman *et al.*, 2008; Elowitz *et al.*, 1999; English *et al.*, 2011; Golding and Cox, 2004; Jasnin *et al.*, 2008; Konopka *et al.*, 2006; Kumar *et al.*, 2010; Mika *et al.*, 2010; Mullineaux *et al.*, 2006; Nenninger *et al.*, 2010; Slade *et al.*, 2009; van den Bogaart *et al.*, 2007) on diffusion of various probes, including small molecules (water, glucose), proteins and plasmids, in the cytoplasm of *E. coli*. We used those data and the scaling function of viscosity (Hołyst *et al.*, 2009; Kalwarczyk *et al.*, 2011; Szymański *et al.*, 2006a, b) to predict the mobility of macromolecules in the bacterial cytoplasm. We also predicted the DCs of amino acids, sugars, proteins and DNA. We created a unique database, including the DCs of all proteins of strain K12 of *E. coli* (4302 proteins), their oligomers and their potential complexes with translocation proteins; 6600 records in total.

## 2 METHODS

### 2.1 A brief description of the method

**Fig. 1.**
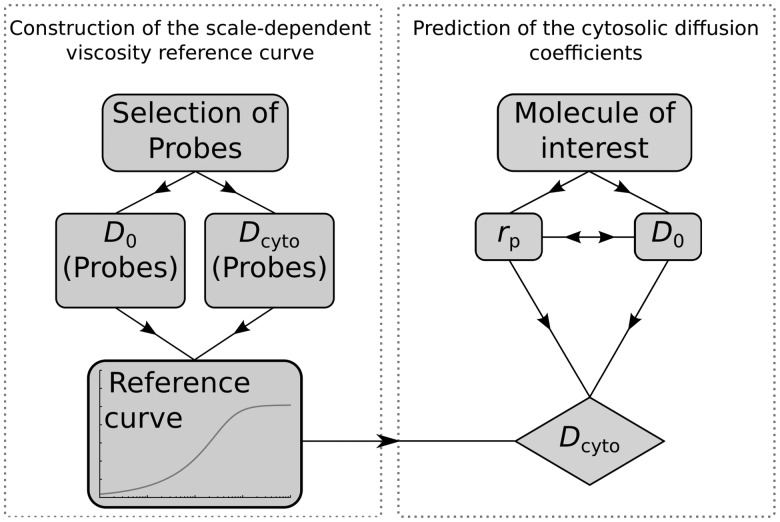
Diagram of a method of predicting the DC of any molecule in the cell cytoplasm. To predict the DCs of molecules in the cytoplasm, it is essential to correctly select the probes that will be used to determine the reference curve. Next, one need to measure the DCs of selected probes in water (buffer) 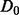 and the DC in the cytoplasm of studied cell 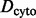. Using 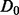 and 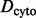, we create the sdVRC. To predict the DC of a given molecule, it is necessary to know its hydrodynamic radius 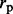 or 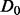. Although sdVRC depends on both 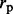 and 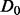, in practice, both parameters can be calculated knowing only one of them. Finally, by substituting the values of 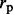 and 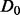 to sdVRC, the DC in the cytoplasm 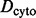 can be determined

Our predictions of DCs of proteins in the bacterial cytoplasm are based on experimental data on diffusion in the cytoplasm of *E. coli* available in the literature (Bakshi *et al.*, 2012; Campbell and Mullins, 2007; Cluzel *et al.*, 2000; Derman *et al.*, 2008; Elowitz *et al.*, 1999; English *et al.*, 2011; Golding and Cox, 2004; Jasnin *et al.*, 2008; Konopka *et al.*, 2006; Kumar *et al.*, 2010; Mika *et al.*, 2010; Mullineaux *et al.*, 2006; Nenninger *et al.*, 2010; Slade *et al.*, 2009; van den Bogaart *et al.*, 2007). The method relies on the dependence 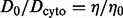, where 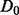 is the DC of macromolecule in water of viscosity 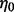, and 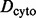 is the DC of macromolecule in the cytoplasm. 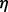 is the effective viscosity experienced by the macromolecule during diffusion in the cytoplasm. The protocol of determination of DCs is graphically represented in Figure 1.

### 2.2 Calculation of hydrodynamic radii and DCs in water

Hydrodynamic radius of proteins was determined using the following formula (Dill *et al.*, 2011):
(1)
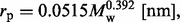

while for RNA we used Equation (2) (Werner, 2011).
(2)
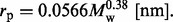



Dependence of the hydrodynamic radii of linear, circular or super coiled DNA on molecular weight [Equations (3)–(5), respectively] was obtained from DCs of DNA constructs (Robertson *et al.*, 2006) using Equation (6).
(3)
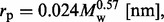

(4)
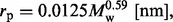

(5)
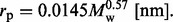

Radii of amino acids and sugars have been calculated, assuming that the hydrodynamic radius 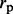 corresponds to the van der Waals radius 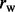 calculated according to the procedure described elsewhere (Zhao *et al.*, 2003).

For each probe, we use the literature values of 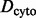, while the values of 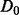 (if not available) were calculated using the Stokes–Sutherland–Einstein equation [Equation (6)].
(6)
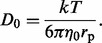



### 2.3 Calculation of DCs of various molecules in the cytoplasm of *E. coli*

Using the molecular weights from Uniprot protein database (Apweiler *et al.*, 2011; Jain *et al.*, 2009), we calculated the DCs for the complete proteome of *E. coli* (K12 strain). We identified the cellular localization of each protein as well as its quaternary structure (a single polypeptide chain or multiple chain aggregates or complexes). In the case of membrane or periplasmic proteins, we adopted the assumption that, after synthesis, the proteins diffuse via the cytoplasm to its target in the membrane, through one of two transport pathways [twin-arginine translocation (TAT) or the general secretion system (Sec)] (Driessen and Nouwen, 2008; Sargent, 2007). Consequently, these proteins were considered as single polypeptide chains (the TAT pathway) or protein complexes with SecB or Tig proteins (the Sec pathway). Hydrodynamic radius of proteins was determined using Equation (1). When the protein was composed of several subunits, the molecular weight of all polypeptide chains comprising the protein was added together. On the basis of cumulative molecular weight of the complex, hydrodynamic radius of the protein 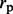 and further its DC 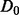 was calculated [Equations (1) and (6)]. Then, using Equation (7), we calculated the relative DCs for all analysed proteins, and we calculated the DCs of proteins in the cytoplasm 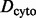. The calculated DCs of all proteins in the cytoplasm are summarized in Supplementary Table S1.

## 3 RESULTS AND DISCUSSION

### 3.1 Construction of the scale-dependent viscosity reference curve

**Fig. 2.**
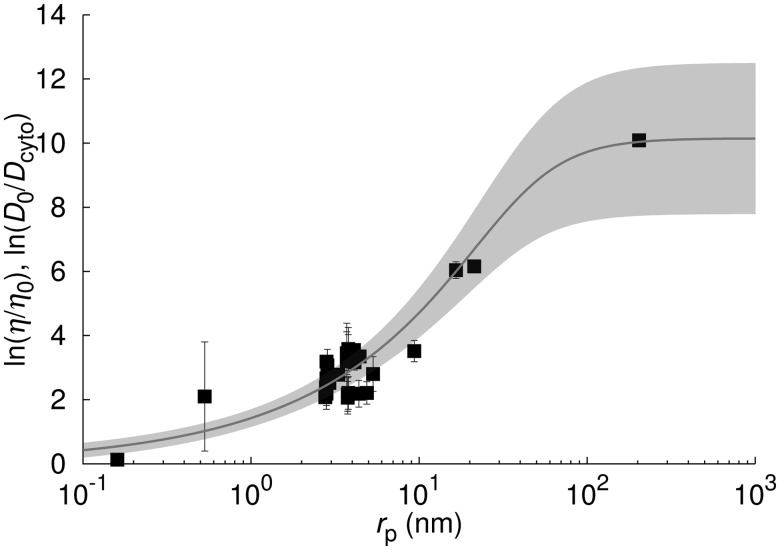
The sdVRC. The logarithm of viscosity 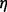 divided by the viscosity of water 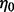 [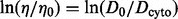] as a function of the hydrodynamic radius 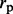 of various probes (Table 1) of radii from 0.16 nm to 203 nm (closed square). The cytoplasmic DCs 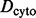 of probes were taken from the literature (Bakshi *et al.*, 2012; Campbell and Mullins, 2007; Cluzel *et al.*, 2000; Elowitz *et al.*, 1999; English *et al.*, 2011; Golding and Cox, 2004; Jasnin *et al.*, 2008; Konopka *et al.*, 2006; Kumar *et al.*, 2010; Mika *et al.*, 2010; Mullineaux *et al.*, 2006; Nenninger *et al.*, 2010; Slade *et al.*, 2009; van den Bogaart *et al.*, 2007) (cf. Table 1). By fitting the data with Equation (7) (solid line), we determined two length scales: 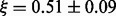 nm and 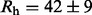 nm. We also determined the macroscopic viscosity of the cytoplasm 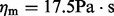, i.e. 26 000 times higher than the viscosity of water 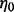 at 310 K. Shading represents the maximum error of fitting

We collected the literature data (Bakshi *et al.*, 2012; Campbell and Mullins, 2007; Cluzel *et al.*, 2000; Elowitz *et al.*, 1999; English *et al.*, 2011; Golding and Cox, 2004; Jasnin *et al.*, 2008; Konopka *et al.*, 2006; Kumar *et al.*, 2010; Mika *et al.*, 2010; Mullineaux *et al.*, 2006; Nenninger *et al.*, 2010; Slade *et al.*, 2009; van den Bogaart *et al.*, 2007) for DCs of different solutes and macromolecules in the cytoplasm of *E. coli* (Fig. 2 and Table 1). We used the least squares method to fit those data with Equation (7) (Kalwarczyk *et al.*, 2011).
(7)
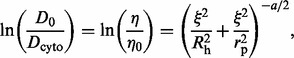

here 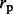 is the hydrodynamic radius of the probe, and 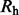 and 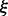 are length scales characterizing the cytoplasm. 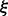 (an average distance between surfaces of proteins), 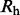 (average hydrodynamic radius of the biggest crowders) and *a* (a constant of the order of one) are the fitting parameters whose values for the cytoplasm of *E. coli* are as follows: 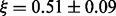 nm, 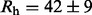 nm and 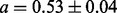. From the scale-dependent viscosity reference curve (sdVRC), we directly determined the macroscopic viscosity 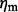 of the cytoplasm. We found that 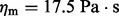 (26 000 times greater than the viscosity of water – 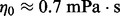 at 310 K). 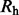 is comparable to the radius of the loops (Kim et al., 2004) of DNA covered with proteins. The second length scale determined from sdVRC, 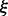, is comparable to the average distance between surfaces of proteins. 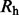 determines the length scale above which the viscosity ceases to depend on the size of the probe and reaches the macroscopic value. For a probe smaller than *ξ*, the experienced viscosity has a value comparable to the viscosity of water.

**Table 1.** Data used in the construction of sdVRC—cf. Figure 2

Probe	*M*_w_ (kDa)	*r*_p_ (nm)	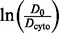	Reference
Water	0.018	0.16	0.1	Jasnin *et al.* (2008)
Glucose	0.423	0.53	2.1	Mika *et al.* (2010)
mEos2	26	2.8	2.1	English *et al.* (2011)
EYFP	27	2.8	2.4	Kumar *et al.* (2010)
GFP	27	2.8	2.4	Elowitz *et al.* (1999)
GFP	27	2.8	3.2	Elowitz *et al.* (1999)
GFP	27	2.8	2.2	van den Bogaart *et al.* (2007)
GFP	27	2.8	2.6	Slade *et al.* (2009)
GFP2	27	2.8	2.3	Nenninger *et al.* (2010)
GFP	27	2.8	3.2	Mika *et al.* (2010)
GFP	27	2.8	2.7	Konopka *et al.* (2006)
GFP-His6	28	2.8	3.1	Elowitz *et al.* (1999)
torA-GFP	30	2.9	2.5	Mullineaux *et al.* (2006)
CheY-GFP	41	3.3	2.8	Cluzel *et al.* (2000)
NlpA-GFP	55	3.7	3.4	Nenninger *et al.* (2010)
NlpA 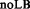 -GFP	55	3.7	3.2	Nenninger *et al.* (2010)
torA-GFP2	57	3.8	2.2	Nenninger *et al.* (2010)
torA-GFP2	57	3.8	2.1	Nenninger *et al.* (2010)
AmiA-GFP	58	3.8	3.6	Nenninger *et al.* (2010)
AmiA-GFP	58	3.8	3.6	Nenninger *et al.* (2010)
AmiA 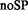 -GFP	58	3.8	2.2	Nenninger *et al.* (2010)
CFP-CheW-YFP	71	4.1	3.5	Kumar *et al.* (2010)
cMBP-GFP	72	4.1	3.2	Elowitz *et al.* (1999)
torA-GFP3	84	4.4	2.2	Nenninger *et al.* (2010)
CFP-CheR-YFP	86	4.4	3.3	Kumar *et al.* (2010)
torA-GFP4	111	4.9	2.2	Nenninger *et al.* (2010)
torA-GFP5	138	5.3	2.8	Nenninger *et al.* (2010)
(β-Gal-GFP)_4_	582	9.4	3.5	Mika *et al.* (2010)
Ribosome 70S	2,500	16.6	6.0	Bakshi *et al.* (2012)
mRNA-GFP	6,000	21.3	6.2	Golding and Cox (2004)
Plasmid-GFP	18,480	203.9	10.1	Campbell and Mullins (2007)

We used as-obtained sdVRC [Equation (7)] as a tool for prediction of DCs of all known proteins of K12 strain (Blattner *et al.*, 1997) of *E. coli* as well as other molecules and macromolecules.

### 3.2 Interpretation of sdVRC

For more than a decade, diffusion of various proteins in the cytoplasm of *E. coli* has been studied (Table 1) (Bakshi *et al.*, 2012; Campbell and Mullins, 2007; Cluzel *et al.*, 2000; Elowitz *et al.*, 1999; English *et al.*, 2011; Golding and Cox, 2004; Jasnin *et al.*, 2008; Konopka *et al.*, 2006; Kumar *et al.*, 2010; Mika *et al.*, 2010; Mullineaux *et al.*, 2006; Nenninger *et al.*, 2010; Slade *et al.*, 2009; van den Bogaart *et al.*, 2007). Those experimental data show that the DCs exponentially depend on the size of the diffusing molecule. For example, GFP with a molecular weight 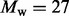 kDa and hydrodynamic radius 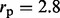 nm is characterized by cytoplasmic DC (Elowitz *et al.*, 1999) 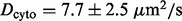. On the other hand, the DC of large oligomeric protein consisting of four subunits of GFP-tagged β-galactosidase (β-gal-GFP)_4_, of radius almost three times greater than GFP (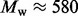 kDa, 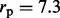 nm), is equal to 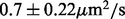 (Mika *et al.,* 2010). The above differences are explained in terms of scale-dependent viscosity (Kalwarczyk *et al.*, 2011) experienced by the diffusing molecule [cf. sdVRC, Equation (7)]. Equation (7) is an empirical equation primarily found for synthetic systems such as polymer or micellar solutions (Hołyst *et al.*, 2009; Kalwarczyk *et al.*, 2011; Szymański *et al.*, 2006a, b). Interpretation of four parameters in Equation (7) (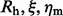 and 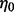) is taken from those studies (Hołyst *et al.*, 2009; Kalwarczyk *et al.*, 2011; Szymański *et al.*, 2006a, b). In synthetic systems, 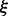 is the average distance between macromolecular components of the complex liquid and 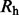 is equal to the hydrodynamic radius of a polymer random coil or of a micelle. In sdVRC, both 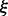 and 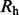 determine the viscosity experienced by a probe diffusing in the investigated liquid. For 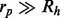, the probe experiences the macroscopic viscosity 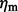. A probe of radius 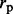 smaller than 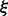 moving in the liquid experiences the viscosity of the solvent 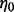. On the other hand, a probe of 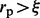 will experience a viscosity higher than the viscosity of the solvent. Finally, the effective viscosity 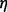 experienced by a probe of radius between 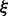 and 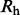 (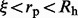) depends exponentially on 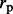. In case of the cytoplasm of mammalian cells, 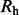 corresponds to the hydrodynamic radius of the filaments forming the cellular cytoskeleton in the volume of the cytoplasm (Kalwarczyk *et al.*, 2011). The bacterial cytoskeleton (Shih and Rothfield, 2006), however, is located directly next to the inner membrane (Pogliano, 2008). We can therefore assume that it should not have a large contribution to the viscosity experienced by the proteins diffusing across the cytoplasm. This assumption is also supported by the value of 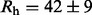 nm determined from fitting, which is similar to the radius of the objects identified as fragments of the bacterial nucleoid (around 40 nm) (Kim *et al.*, 2004), i.e. loops of DNA covered with structural proteins. This value can be compared with the value of the hydrodynamic radius of the filaments forming the bacterial cytoskeleton (Hou *et al.*, 2012; Pogliano, 2008) (fragments of length *L* = 100 nm and a radius *r* = 2.5 nm), which is ~17 nm (Vandesande and Persoons, 1985), well below 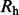, obtained from the fit. Therefore, the length scale, 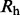, is neither correlated with the hydrodynamic radius of the filaments nor with the proteins whose highest hydrodynamic radius is about 10 nm. 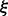 in the cytoplasm of *E. coli* equals 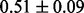 nm and is comparable with the average distance between proteins. Parameters of the sdVRC (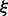 and 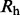) depend on the internal structure of the cytoplasm (proteins density, size of the nucleoid, etc.). Thus, each cell type will be characterized by a different shape of the reference curve (due to differences in parameters 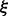 and 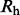), while the mathematical form of the sdVRC will not change, and such curve can be constructed for other cell types.

### 3.3 Other models of diffusion in the cytoplasm

**Fig. 3.**
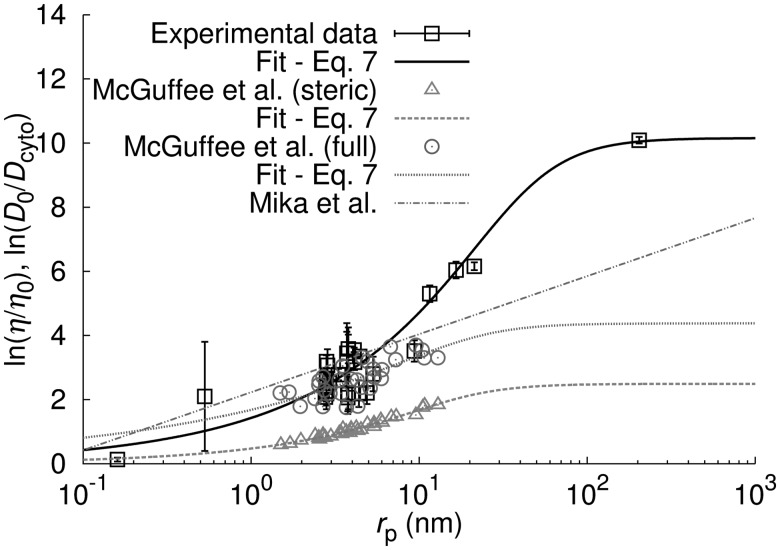
The comparison of sdVRC with other existing models. The plot shows the literature values for the logarithm of 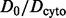 (open squares) (Bakshi *et al.,* 2012; Campbell and Mullins, 2007; Cluzel *et al.,* 2000; Elowitz *et al.,* 1999; English *et al.,* 2011; Golding and Cox, 2004; Jasnin *et al.,* 2008; Konopka *et al.,* 2006; Kumar *et al.,* 2010; Mika *et al.,* 2010; Mullineaux *et al.,* 2006; Nenninger *et al.,* 2010; Slade *et al.,* 2009; van den Bogaart *et al.,* 2007). Black solid line represents Equation (7) with parameters: 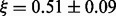 nm, 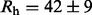 nm and 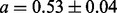. We compared our results with data generated by McGuffee and Elcock (2010) and Mika and Poolman (2011). The data generated by McGuffee and Elcock (2010) were fitted by Equation (7), yielding the following parameters: for the ‘full’ model 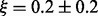 nm, 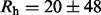 nm and 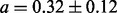 (dotted circle, dotted line), for the ‘steric’ model 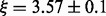 nm, *R*_h_ = 17 ± 6 nm and 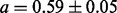 (open diamond, dashed line). The model proposed by Mika and Poolman (2011) where 
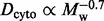
 is plotted as dashed–dotted line

**Fig. 4.**
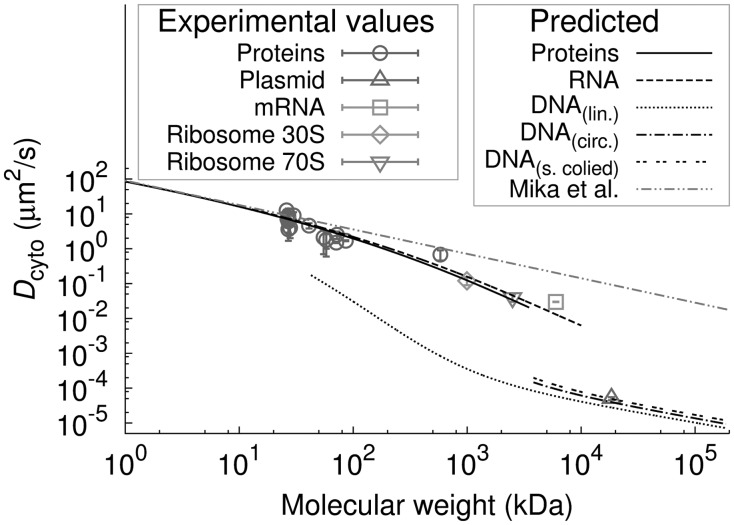
Comparison of measured and predicted 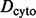 as a function of molecular weight of the investigated probes. Predicted dependencies shown in the graph are expressed by Equation (7). The hydrodynamic radius 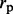 of each type of macromolecules is given by the relationship: 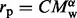 nm, where 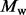 is the molecular weight of the macromolecule. For proteins *C* = 0.0514 and *α* = 0.392—Equation (1); RNA *C* = 0.0566 and *α* = 0.38—Equation (2), linear DNA *C* = 0.024 and *α* = 0.57—Equation (3); circular DNA *C* = 0.0125 and *α* = 0.59—Equation (4); super coiled *C* = 0.0145 and *α* = 0.57—Equation (5). For comparison, we present experimental data on DCs of proteins (Cluzel *et al.,* 2000; Elowitz *et al.,* 1999; English *et al.,* 2011; Konopka *et al.,* 2006; Kumar *et al.,* 2010; Mika *et al.,* 2010; Mullineaux *et al.,* 2006; Nenninger *et al.,* 2010; Slade *et al.,* 2009), RNA (Golding and Cox, 2004), plasmid (Campbell and Mullins, 2007) and ribosomes 30S and 70S (Bakshi *et al.,* 2012). The dashed–dotted straight line indicates the relationship 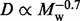 proposed by Mika and Poolman (2011). The dependence of 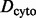 on 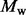 proposed by Mika and Poolman (2011), when applied to large plasmids (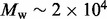 kDa), yields several orders of magnitude overestimation of DC

We compared our results with three models of diffusion in the cytoplasm of *E. coli*, available in the literature (Figures 3 and 4). McGuffee and Elcock (2010) proposed two models of diffusion in the cytoplasm: the ‘steric’ model, which takes into account only steric interactions between diffusing proteins, and the ‘full’ model, which includes steric, electrostatic and hydrodynamic interactions between diffusing entities. Comparison of the results (Figure 3) shows that the model we propose takes into account possible interactions between the diffusing probes and the surrounding environment. Moreover, we show that the full information needed to build the sdVRC can be obtained only after taking into account the probes whose 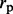 greatly exceeds 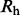. For example, simulations conducted by McGuffee and Elcock (2010) include proteins that are most abundant in the cytoplasm, but the absence of large objects such as the nucleoid leads to underestimated values of 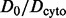. The effect starts to be meaningful for probes whose 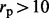 nm. In that case, the values of 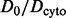 are lower by an order of magnitude with respect to experimental results.

We also compared our results with the model proposed by Mika and Poolman (2011), where 
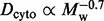
. As can be seen, the power law dependence of 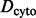 on 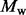 may also lead to underestimated values of 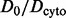. For example, for the ribosome 70S 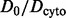 measured experimentally is five times higher than predicted using power law dependence. Therefore, the power law dependence proposed by Mika and Poolman (2011) holds for the proteins in a small range of molecular weights 20–30 kDa and, moreover, is not applicable to macromolecules other than proteins. This is because each type of macromolecules (DNA, RNA, proteins, polymers, etc.), has different shape and thus different dependence of 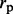 on 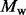 [Equations (1)–(5)]. The shape of the macromolecule and in consequence its radius translates into the DC. The dependence of DC 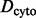 of different types of macromolecules (proteins, RNA and DNA) on their molecular weight is shown in Figure 4.

### 3.4 Accuracy of the model

Accuracy in determination of the sdVRC strongly depends on the amount of available data. One would expect that increasing the amount of data for probes of 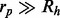 and 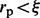, would significantly decrease the maximum error of the sdVRC (compare Fig. 2).

To test the accuracy of the presented method, we perform an analysis of the error of calculation of DC 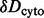 for GFP as a function of the number of experimental data points. Using Equation (7), we generated 10 datasets, where the number of data points ranges from 6 to 100. The generated data were uniformly distributed on a logarithmic scale and were randomly drawn on the assumption that measurement error is described by a normal distribution with standard deviation 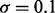. We assumed that the error of 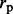 equals to 5%. We found that 20 data points are sufficient to obtain 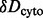 at the level of 20% for the GFP (averaged over 10 generated datasets). In comparison, 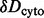 obtained from the analysis of the literature data was at the level of 40% (cf. Fig. 2). This is mainly because of the small number of available experimental data. Furthermore, most of the experimental data are available for a narrow range of hydrodynamic radii (around 3 nm, cf. Fig. 2) which is not preferred in this type of analysis. To date, however, there is no experimental data which would improve the accuracy of the sdVRC. Therefore, to improve the accuracy, additional experiments are needed to cover a wider range of 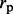 of the probes and also uncertainties of 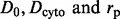 should be minimized.

### 3.5 DCs of proteins

**Fig. 5.**
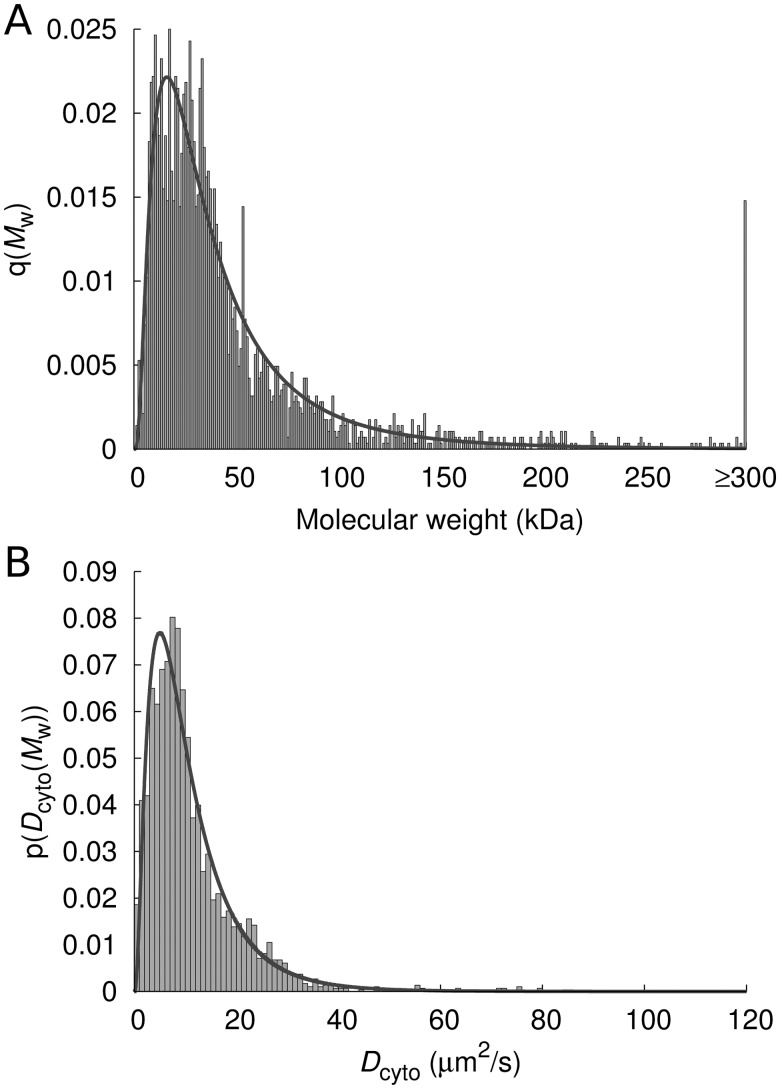
Distributions of molecular weights and DCs of cytoplasmic proteins in *E.coli*. (**A**) Histogram of molecular weights of cytoplasmic proteins (created using data from the Uniprot database). The histogram is described by log-normal distribution 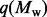 with standard deviation 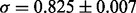 and the mean molecular weight 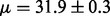 kDa. (**B**) Histogram of DCs of cytoplasmic proteins (from our database) and the probability density function 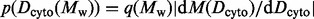
—solid line

Preparing a database of DCs of the entire proteome, one should keep in mind that about 45% of the proteome are proteins forming a larger macromolecular complex (homo-, hetero-oligomers and complexes of membrane proteins with translocation proteins). Thus, the calculation of DCs of proteins should be carried out also for protein complexes. The Uniprot protein database (Apweiler *et al.*, 2011; Jain *et al.*, 2009) contains information on the molecular weight of proteins, their quaternary structure and their location in cell. Using these data and sdVRC (cf. Fig. 2) we calculated the DCs 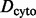 of all proteins in *E. coli* (Supplementary Table S1) present in the cytoplasm as monomers (single polypeptide chains) or as multimers (homo- or hetero-oligomers) or complexes composed of many chains, see Fig. 5). Figure 5A shows the histogram of molecular weights of cytoplasmic proteins, including homo- and hetero-multimers. Distribution of molecular weights of proteins is given by log-normal distribution with probability density function 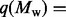

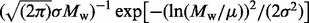
, where standard deviation 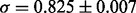 and mean molecular weight 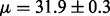 kDa. The relationship between the DC and the molecular weight of protein is expressed by the Equations (1) and (7). A histogram of DCs of cytoplasmic proteins is shown in Figure 5B (same proteins as in Fig. 5A). The distribution follows the curve given by the probability density function: 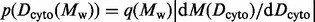.

We also calculated 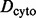 of membrane proteins that are ~30% of the proteome of *E. coli*. Membrane proteins, after synthesis by the ribosome, are transported to the membrane, according to one of the two pathways: the TAT (Sargent, 2007) in which proteins are transported as single polypeptides in a folded state and the Sec (Driessen and Nouwen, 2008) in which unfolded proteins are complexed mainly by one of the two proteins: SecB or Tig.

We created a database (Supplementary Table S1) listing the DCs of all proteins, including their monomeric forms, the possible homo- and hetero-multimers, and in the case of membrane proteins also the complexes with translocation proteins (SecB and Tig). Apart from DCs of proteins, we calculated 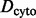 of small molecules such as amino acids or sugars and for macromolecules such as RNA or DNA (linear, circular and super coiled). Calculated values of DCs are listed in Table 2.

**Table 2.** Predicted, cytoplasmic DCs of small amino acids, sugars, selected proteins and ribosomes and DNA constructs

Molecule	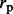 (nm)	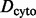 ( 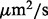 )
Guanine	0.29	539
Histidine	0.32	478
Galactose	0.33	458
Arginine	0.34	428
Lactose	0.41	328
ATP	0.43	302
TrpR–Monomer	2.1	19.71
TrpR–Dimer	2.7	10.92
LacI–Monomer	3.2	7.28
LacI–Tetramer	5.6	1.79
RNAP Holoenzyme	8.5	0.5
Ribosome 30s	11.6	0.18
Ribosome 50s	13.2	0.11
Ribosome 70s	16.6	0.05
Pyes2	142^a^	1.13 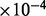
CTD-2657L24	802^b^	1.62 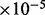

^a^Hydrodynamic radius calculated using Equation (3). ^b^Hydrodynamic radius calculated using Equation (5).

The predicted DCs refer only to three-dimensional diffusion. In cells, particularly eukaryotes, there are also other types of transport such as molecular motors (Vale, 2003). Nevertheless, mobility, irrespective of the type of motion, is inversely proportional to the viscosity of the surrounding environment. Since the viscosity is dependent on the scale (Hołyst *et al.*, 2009; Kalwarczyk *et al.*, 2011; Szymański *et al.*, 2006a, b), each type of motion will depend exponentially [Equation (7)] on the size of a moving object.

### 3.6 Application of DC database in studies of biochemical processes occurring in cells

Using the database of DCs, one can determine quantitatively whether the protein diffuses freely or interacts and forms complexes with much larger macromolecules, e.g. plasmids. Capoulade *et al.* (2011) performed diffusion measurements and showed that, in the nucleus of eukaryotic cell, euchromatin creates domains of high and low affinity for heterochromatin protein (HP1α).

Another kind of analysis was performed by Elf *et al.* (2007). Authors compared *in vivo* DCs of both: the lactose repressor in its native form and the lactose repressor devoid of the DNA-binding domain. Order of magnitude difference in the coefficient of diffusion of both proteins led to the conclusion that the native lactose repressor spends 87% of the time attached to the DNA. This shows that the presence of attractive interactions between diffusing particles will result in a slowdown of diffusion of molecules.

To clarify the method, consider a hypothetical protein of hydrodynamic radii 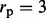 nm. The DCs of this protein 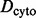 (calculated from sdVRC) is approximately equal to 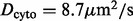. The time required by the protein to visit every place in the cell volume [for *E. coli V* 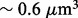 (Kubitschek, 1990)] is approximately equal to 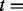

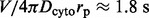
. Now suppose that the protein binds to a plasmid whose molecular weight equals to 10 000 kDa, the DC of the plasmid is of the order of 
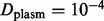
 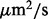. Suppose further that the protein spends one-tenth of the time diffusing freely 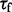, and the remaining 90% of time 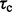 as a complex with the plasmid (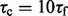). The effective DCs of the complexes 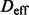, defined as 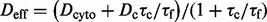, and under assumption that 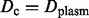, will be nearly an order of magnitude lower than the predicted one (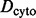):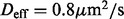. According to the above analysis, we can assume that any deviation of experimentally measured DC from the proposed sdVRC will result from intermolecular interactions such as specific or non-specific binding.

### 3.7 Diffusion in the cytoplasm and the diffusion in organelles of eukaryotes

Prokaryotic cells are characterized by small sizes [volume of *E. coli* is approximately *V* 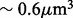 (Kubitschek, 1990)]. Measurements of diffusion in the cytoplasm of these cells are performed for the entire volume of the cytoplasm. Thereby, the effective DC measured in these experiments is the value averaged over the entire volume of the cytoplasm. Because the sdVRC was found on the basis of DCs, in the case of *E. coli*, this curve is also averaged over the entire volume of the cell. At this point, it should be stressed that the sdVRC should not be used to describe diffusion on the cell membrane due to structural differences between membrane and cytoplasm, and the two-dimensional nature of such diffusion.

Small sizes of prokaryotic cell also affect the long-time behaviour of diffusing objects. This is known as confined diffusion (Ochab-Marcinek and Holyst, 2011). Nevertheless, from the normal, three-dimensional DCs (short time diffusion), one can draw constructive conclusions. For example, English *et al.* (2011) on the basis of short-time diffusion measurements have characterized the catalytic cycle of RelA protein.

Eukaryotic cells are much larger than bacteria. Therefore, measurements of diffusion in these cells are easier and can be performed in the individual organelles [e.g. nucleus (Pederson, 2000)]. In previous work, we showed that it is possible to construct a reference curve for the cytoplasm of mammalian HeLa and Swiss 3T3 cells (Kalwarczyk, *et al.*, 2011). However, based on comparison of the results obtained by Lukacs *et al.* (2000) for the cytoplasm and the nucleus of HeLa cancer cell, we expect that the sdVRC determined for each cellular organelle is different. Furthermore, as sdVRC depends on the structure of the environment where diffusion occurs, it should be unique for a given cell or even organelle.

## 4 CONCLUSION

The method presented above has a high predictive power. Although, so far a large error of the method (40% for proteins), the experimentally measured DCs coincide remarkably well with the predicted DCs (cf. Fig. 4). Therefore, measurements of DCs of several inert probes in a single cell type allow to determine the DCs of thousands of proteins and other (macro)molecules. Correctly designed experiment would require involvement of different experimental techniques (NMR, FRAP, FCS, particle tracking) and the use of probes in a wide range of sizes. One needs to know the DC of a given probe in water and/or the hydrodynamic radius of this probe. Additionally for the same probe, measurements of diffusion in cytoplasm of the cell should be performed. Sizes of selected probes should be uniformly distributed along the logarithmic scale of sizes. We showed that only 20 measurements are required to predict the cytoplasmic DC of the typical protein with 20% accuracy.

Analysis of the sdVRC allows to determine the characteristic length scales 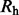 and 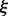, and the DC of any (macro)molecule in the cytoplasm. For the cytoplasm of *E. coli,* we found that 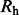 is surprisingly well correlated with the average radius of the DNA loops forming the nucleoid. This suggests that the nucloeid is the main crowding agent (responsible for the macroscopic viscosity) in the cytoplasm of *E. coli*.

Finally, it should be noted that there are no additional requirements (except experimental data) to construct analogous database of DCs in other systems such as the nucleus or mitochondria of eukaryotic cells. We also believe that sdVRC can be easily adopted to calculate other types of mobility, including one-dimensional sliding, velocity of molecular motors, etc*.*, as they all are inversely proportional to the viscosity.

## Supplementary Material

Supplementary Data
