# Supplementary material for: Biologistics—Diffusion coefficients for complete proteome of Escherichia coli
Source: Bioinformatics. 2012 Aug 31;28(22):2971–8. doi: 10.1093/bioinformatics/bts537 (PMC3496334; doi:10.1093/bioinformatics/bts537)
Supplement: Supplementary Data [file supp_28_22_2971__index.html]

Biologistics – diffusion coefficients for complete proteome of Escherichia coli — Biologistics—Diffusion coefficients for complete proteome of Escherichia coli — Supplementary Data 

# Biologistics—Diffusion coefficients for complete proteome of *Escherichia coli*

## Supplementary Data

files

**Files in this Data Supplement:**

- Supplementary Data - pdf file
