# Supplementary material for: Biologistics—Diffusion coefficients for complete proteome of Escherichia coli
Source: Bioinformatics. 2012 Aug 31;28(22):2971–8. doi: 10.1093/bioinformatics/bts537 (PMC3496334; doi:10.1093/bioinformatics/bts537)
Supplement: Supplementary Data [file supp_bts537_Table_S1.pdf]

Table S1: The diffusion coefficients of proteins from the proteome of K12 strain of *E. coli*. Table contains Uniprot database accession number, protein name, location in the cell, information about the quaternary structure (monomer, multimer or, for membrane proteins, complex with translocation proteins), diffusion coefficient in water at temperatures of 20, 30, and 37 Celsius degrees (293, 303, and 310 K), and the diffusion coefficients in the cytosol, at same temperatures.

UniProtKB A. N.	Protein name	Subcellular location	Subunit	D_0 $\left(\frac{\mu m^2}{s}\right)$			D_{cyto} $\left(\frac{\mu m^2}{s}\right)$		
				310 K	303 K	293 K	310 K	303 K	293 K
P46482	AaeA	Cell inner membrane	Monomer	105.8	89.4	69.1	8.0	6.8	5.2
			Complex with SecB	68.9	56.3	45.0	2.7	2.2	1.8
			Complex with TF (Tig)	75.3	63.6	49.1	3.5	2.9	2.3
P46481	AaeB	Cell inner membrane	Monomer	78.9	66.6	51.5	3.9	3.3	2.5
			Complex with SecB	60.9	49.7	39.7	2.0	1.6	1.3
			Complex with TF (Tig)	64.8	54.7	42.3	2.3	2.0	1.5
P67662	AaeR		Monomer	106.2	89.7	69.3	8.1	6.8	5.3
P46478	AaeX	Cell membrane	Monomer	189.7	160.3	123.8	28.3	23.9	18.5
			Complex with SecB	77.5	63.3	50.6	3.7	3.0	2.4
			Complex with TF (Tig)	87.8	74.2	57.3	5.1	4.3	3.3
P31119	Aas	Cell inner membrane	Monomer	76.1	64.3	49.7	3.6	3.0	2.3
			Complex with SecB	59.7	48.8	39.0	1.8	1.5	1.2
			Complex with TF (Tig)	63.3	53.5	41.3	2.2	1.8	1.4
P0A8P1	Aat	Cytoplasm	Monomer	117.5	99.3	76.7	10.2	8.6	6.7
P77357	AbgA		Monomer	94.4	79.7	61.6	6.1	5.2	4.0
P76052	AbgB		Monomer	90.3	76.3	58.9	5.5	4.6	3.6
P77744	AbgR		Monomer	106.7	90.1	69.6	8.2	6.9	5.3
P46133	AbgT	Cell inner membrane	Monomer	88.5	74.8	57.8	5.2	4.4	3.4
			Complex with SecB	64.3	52.5	42.0	2.3	1.9	1.5
			Complex with TF (Tig)	69.1	58.4	45.1	2.8	2.3	1.8
P75747	AbrB	Cell inner membrane	Monomer	103.4	87.4	67.5	7.6	6.4	5.0
			Complex with SecB	68.4	55.9	44.6	2.7	2.2	1.7
			Complex with TF (Tig)	74.5	63.0	48.6	3.4	2.8	2.2
P0ABD5	AccA	Cytoplasm	Monomer	105.3	89.0	68.7	7.9	6.7	5.2
			Heterohexamer (AccB$-$AccC$-$AccA$_2$$-$AccD)	56.8	48.0	37.1	1.6	1.4	1.1

UniProtKB A. N.	Protein name	Subcellular location	Subunit	D_0 $\left(\frac{\mu m^2}{s}\right)$			D_{cyto} $\left(\frac{\mu m^2}{s}\right)$		
				310 K	303 K	293 K	310 K	303 K	293 K
P0ABD8	AccB		Monomer	141.1	119.2	92.1	15.3	13.0	10.0
			Homodimer	107.6	87.9	70.2	8.3	6.8	5.4
			Heterohexamer (AccB−AccC−$AccA_2$−AccD)	56.8	48.0	37.1	1.6	1.4	1.1
P24182	AccC		Monomer	92.3	78.0	60.2	5.8	4.9	3.8
			Heterohexamer (AccB−AccC−$AccA_2$−AccD)	56.8	48.0	37.1	1.6	1.4	1.1
P0A9Q5	AccD	Cytoplasm	Monomer	107.6	90.9	70.2	8.3	7.0	5.4
			Heterohexamer (AccB−AccC−$AccA_2$−AccD)	56.8	48.0	37.1	1.6	1.4	1.1
P0A9G6	AceA	Cytoplasm	Monomer	93.6	79.1	61.1	6.0	5.1	3.9
			Homotetramer	54.4	44.4	35.5	1.4	1.2	0.9
P08997	AceB	Cytoplasm	Monomer	85.3	72.1	55.7	4.8	4.0	3.1
P0AFG8	AceE		Monomer	70.0	59.2	45.7	2.9	2.4	1.9
			Homodimer	53.4	43.6	34.8	1.3	1.1	0.9
P06959	AceF		Monomer	82.3	69.5	53.7	4.3	3.7	2.8
P11071	AceK	Cytoplasm	Monomer	81.5	68.9	53.2	4.2	3.6	2.8
P0A6A3	AckA	Cytoplasm	Monomer	97.1	82.1	63.4	6.5	5.5	4.3
			Homodimer	74.0	60.5	48.3	3.3	2.7	2.2
P25516	AcnA		Monomer	70.6	59.6	46.1	2.9	2.5	1.9
P36683	AcnB		Monomer	71.8	60.7	46.9	3.1	2.6	2.0
P21515	AcpH		Monomer	124.5	105.2	81.3	11.6	9.8	7.6
P0A6A8	AcpP	Cytoplasm	Monomer	182.7	154.3	119.2	26.3	22.2	17.1
P24224	AcpS	Cytoplasm	Monomer	151.0	127.6	98.5	17.7	15.0	11.6
			Homodimer	115.1	94.0	75.1	9.7	7.9	6.3
P37623	AcpT		Monomer	127.2	107.4	83.0	12.2	10.3	8.0
P0AE06	AcrA	Cell inner membrane	Monomer	98.1	82.9	64.0	6.7	5.7	4.4
			Complex with SecB	67.1	54.8	43.8	2.5	2.1	1.7
			Complex with TF (Tig)	72.8	61.5	47.5	3.2	2.7	2.1
P31224	AcrB	Cell inner membrane	Monomer	66.6	56.2	43.4	2.5	2.1	1.6
			Complex with SecB	55.2	45.1	36.0	1.5	1.2	1.0
			Complex with TF (Tig)	57.9	48.9	37.8	1.7	1.4	1.1
P24177	AcrD	Cell inner membrane	Monomer	66.7	56.3	43.5	2.5	2.1	1.6
			Complex with SecB	55.3	45.2	36.1	1.5	1.2	1.0

UniProtKB A. N.	Protein name	Subcellular location	Subunit	D_0 $\left(\frac{\mu m^2}{s}\right)$			D_{cyto} $\left(\frac{\mu m^2}{s}\right)$		
				310 K	303 K	293 K	310 K	303 K	293 K
			Complex with TF (Tig)	58.0	49.0	37.9	1.7	1.4	1.1
P24180	AcrE	Cell inner membrane	Monomer	98.9	83.6	64.6	6.8	5.8	4.5
			Complex with SecB	67.3	55.0	43.9	2.6	2.1	1.7
			Complex with TF (Tig)	73.1	61.7	47.7	3.2	2.7	2.1
P24181	AcrF	Cell inner membrane	Monomer	67.0	56.6	43.8	2.5	2.1	1.7
			Complex with SecB	55.5	45.3	36.2	1.5	1.2	1.0
			Complex with TF (Tig)	58.2	49.2	38.0	1.7	1.5	1.1
P0ACS9	AcrR		Monomer	120.9	102.1	78.9	10.9	9.2	7.1
P27550	Acs		Monomer	79.5	67.2	51.9	4.0	3.4	2.6
P32705	ActP	Cell inner membrane	Monomer	85.9	72.6	56.1	4.8	4.1	3.2
			Complex with SecB	63.4	51.8	41.4	2.2	1.8	1.4
			Complex with TF (Tig)	68.0	57.5	44.4	2.6	2.2	1.7
P06134	Ada		Monomer	100.9	85.2	65.8	7.2	6.0	4.7
P22333	Add		Monomer	104.0	87.8	67.8	7.7	6.5	5.0
P31441	Ade		Monomer	83.5	70.5	54.5	4.5	3.8	2.9
			Homodimer	63.6	52.0	41.5	2.2	1.8	1.4
P0A9Q7	AdhE		Monomer	71.0	60.0	46.4	3.0	2.5	1.9
P39451	AdhP		Monomer	105.1	88.8	68.6	7.9	6.7	5.1
P28629	AdiA	Cytoplasm	Monomer	74.8	63.2	48.8	3.4	2.9	2.2
			Homodecamer	30.3	24.8	19.8	0.2	0.2	0.1
P60061	AdiC	Cell inner membrane	Monomer	94.2	79.6	61.5	6.1	5.1	4.0
			Complex with SecB	66.0	53.9	43.1	2.4	2.0	1.6
			Complex with TF (Tig)	71.4	60.3	46.6	3.0	2.5	2.0
P33234	AdiY		Monomer	113.6	96.0	74.2	9.5	8.0	6.2
P69441	Adk	Cytoplasm	Monomer	123.2	104.1	80.4	11.4	9.6	7.4
P0AAP1	AdrA	Cell inner membrane	Monomer	98.7	83.4	64.4	6.8	5.7	4.4
			Complex with SecB	67.2	54.9	43.9	2.6	2.1	1.7
			Complex with TF (Tig)	73.0	61.7	47.6	3.2	2.7	2.1
P76261	AdrB		Monomer	85.6	72.3	55.9	4.8	4.1	3.1
P37127	AegA		Monomer	79.6	67.3	52.0	4.0	3.4	2.6
P50466	Aer	Cell inner membrane	Monomer	88.4	74.7	57.7	5.2	4.4	3.4

UniProtKB A. N.	Protein name	Subcellular location	Subunit	D_0 $\left(\frac{\mu m^2}{s}\right)$			D_{cyto} $\left(\frac{\mu m^2}{s}\right)$		
				310 K	303 K	293 K	310 K	303 K	293 K
			Complex with SecB	64.3	52.5	41.9	2.3	1.9	1.5
			Complex with TF (Tig)	69.1	58.4	45.1	2.8	2.3	1.8
P23872	Aes	Cytoplasm	Monomer	104.4	88.2	68.1	7.8	6.6	5.1
			Homodimer	79.5	65.0	51.9	4.0	3.3	2.6
P42906	AgaA		Monomer	138.5	117.0	90.4	14.7	12.4	9.6
P42909	AgaB	Cytoplasm	Monomer	138.2	116.7	90.2	14.6	12.4	9.6
P42910	AgaC	Cell inner membrane	Monomer	114.2	96.5	74.5	9.6	8.1	6.2
			Complex with SecB	70.6	57.7	46.1	2.9	2.4	1.9
			Complex with TF (Tig)	77.6	65.5	50.6	3.7	3.2	2.4
P42911	AgaD	Cell inner membrane	Monomer	113.6	96.0	74.2	9.5	8.0	6.2
			Complex with SecB	70.5	57.6	46.0	2.9	2.4	1.9
			Complex with TF (Tig)	77.4	65.4	50.5	3.7	3.1	2.4
P42912	AgaI		Monomer	115.7	97.7	75.5	9.8	8.3	6.4
P0ACK2	AgaR		Monomer	112.8	95.3	73.6	9.3	7.9	6.1
P42907	AgaS		Monomer	98.5	83.2	64.3	6.8	5.7	4.4
P42904	AgaV	Cytoplasm	Monomer	139.8	118.1	91.3	15.0	12.7	9.8
P42905	AgaW	Cell inner membrane	Monomer	152.4	128.7	99.4	18.1	15.3	11.8
			Complex with SecB	75.3	61.5	49.1	3.5	2.8	2.3
			Complex with TF (Tig)	84.4	71.3	55.1	4.6	3.9	3.0
P19926	Agp	Periplasm	Monomer	95.1	80.3	62.1	6.2	5.2	4.1
			Complex with SecB	66.3	54.1	43.2	2.5	2.0	1.6
			Complex with TF (Tig)	71.7	60.6	46.8	3.0	2.6	2.0
P0AE08	AhpC		Monomer	129.6	109.5	84.5	12.7	10.7	8.3
			Homodimer	98.7	80.7	64.4	6.8	5.6	4.4
P35340	AhpF		Monomer	87.7	74.1	57.2	5.1	4.3	3.3
			Homodimer	66.8	54.6	43.6	2.5	2.1	1.6
P33224	AidB	Cytoplasm	Monomer	85.1	71.9	55.6	4.7	4.0	3.1
			Homotetramer	49.4	40.4	32.3	1.1	0.9	0.7
P45565	Ais	Periplasm	Monomer	126.1	106.5	82.3	12.0	10.1	7.8
			Complex with SecB	72.5	59.2	47.3	3.1	2.6	2.0
			Complex with TF (Tig)	80.3	67.8	52.4	4.1	3.4	2.7

UniProtKB A. N.	Protein name	Subcellular location	Subunit	D_0 $\left(\frac{\mu m^2}{s}\right)$			D_{cyto} $\left(\frac{\mu m^2}{s}\right)$		
				310 K	303 K	293 K	310 K	303 K	293 K
P00957	AlaS	Cytoplasm	Monomer	71.1	60.0	46.4	3.0	2.5	1.9
			Homotetramer	41.3	33.7	26.9	0.6	0.5	0.4
P25553	AldA		Monomer	90.2	76.2	58.9	5.5	4.6	3.6
			Homotetramer	52.4	42.8	34.2	1.3	1.0	0.8
P37685	AldB		Monomer	87.6	74.0	57.2	5.1	4.3	3.3
			Homotetramer	50.9	41.6	33.2	1.2	1.0	0.8
P04395	AlkA		Monomer	110.2	93.1	71.9	8.8	7.4	5.7
P05050	AlkB		Monomer	122.3	103.3	79.8	11.2	9.4	7.3
P77731	AllA		Monomer	136.5	115.3	89.1	14.3	12.0	9.3
			Homodimer	104.0	85.0	67.9	7.7	6.3	5.0
P77671	AllB		Monomer	92.1	77.8	60.1	5.7	4.9	3.7
			Homotetramer	53.5	43.7	34.9	1.4	1.1	0.9
P77425	AllC		Monomer	95.1	80.3	62.1	6.2	5.2	4.1
			Homodimer	72.5	59.2	47.3	3.1	2.6	2.0
P77555	AllD	Cytoplasm	Monomer	102.3	86.4	66.7	7.4	6.2	4.8
P0ACN4	AllR		Monomer	113.2	95.7	73.9	9.4	7.9	6.1
			Homodimer	86.3	70.5	56.3	4.9	4.0	3.2
			Homotetramer	65.8	55.6	42.9	2.4	2.0	1.6
P0ACR0	AllS		Monomer	106.2	89.7	69.3	8.1	6.8	5.3
P33997	AlpA		Monomer	187.1	158.0	122.1	27.6	23.3	18.0
P0A6B4	Alr		Monomer	101.0	85.4	65.9	7.2	6.1	4.7
P32721	AlsA	Cell inner membrane	Monomer	87.4	73.8	57.0	5.0	4.3	3.3
			Complex with SecB	63.9	52.2	41.7	2.2	1.8	1.5
			Complex with TF (Tig)	68.6	58.0	44.8	2.7	2.3	1.8
P39265	AlsB	Periplasm	Monomer	108.2	91.4	70.6	8.4	7.1	5.5
			Complex with SecB	69.4	56.7	45.3	2.8	2.3	1.8
			Complex with TF (Tig)	75.9	64.2	49.6	3.5	3.0	2.3
P32720	AlsC	Cell inner membrane	Monomer	106.4	89.9	69.4	8.1	6.9	5.3
			Complex with SecB	69.0	56.4	45.1	2.7	2.2	1.8
			Complex with TF (Tig)	75.4	63.7	49.2	3.5	2.9	2.3
P32719	AlsE		Monomer	118.4	100.0	77.3	10.4	8.8	6.8

UniProtKB A. N.	Protein name	Subcellular location	Subunit	D_0 $\left(\frac{\mu m^2}{s}\right)$			D_{cyto} $\left(\frac{\mu m^2}{s}\right)$		
				310 K	303 K	293 K	310 K	303 K	293 K
			Homohexamer	58.7	47.9	38.3	1.8	1.4	1.1
P32718	AlsK		Monomer	107.0	90.4	69.8	8.2	6.9	5.4
P42601	Alx	Cell inner membrane	Monomer	104.5	88.3	68.2	7.8	6.6	5.1
			Complex with SecB	68.6	56.1	44.8	2.7	2.2	1.8
			Complex with TF (Tig)	74.9	63.3	48.9	3.4	2.9	2.2
P36548	AmiA	Periplasm	Monomer	110.1	93.1	71.9	8.8	7.4	5.7
			Complex with SecB	69.8	57.0	45.6	2.8	2.3	1.8
			Complex with TF (Tig)	76.5	64.6	49.9	3.6	3.0	2.4
P26365	AmiB	Periplasm	Monomer	93.3	78.8	60.9	5.9	5.0	3.9
			Complex with SecB	65.8	53.7	42.9	2.4	2.0	1.6
			Complex with TF (Tig)	71.0	60.0	46.4	3.0	2.5	1.9
P63883	AmiC	Periplasm	Monomer	95.1	80.4	62.1	6.2	5.3	4.1
			Complex with SecB	66.3	54.2	43.3	2.5	2.0	1.6
			Complex with TF (Tig)	71.7	60.6	46.8	3.0	2.6	2.0
P75820	AmiD	Cell outer membrane	Monomer	110.6	93.5	72.2	8.9	7.5	5.8
			Complex with SecB	69.9	57.1	45.6	2.8	2.3	1.9
			Complex with TF (Tig)	76.6	64.7	50.0	3.6	3.1	2.4
P0AE12	Amn		Monomer	89.1	75.3	58.1	5.3	4.5	3.5
P00811	AmpC	Periplasm	Monomer	98.7	83.4	64.4	6.8	5.7	4.4
			Complex with SecB	67.2	54.9	43.9	2.6	2.1	1.7
			Complex with TF (Tig)	73.0	61.7	47.6	3.2	2.7	2.1
P13016	AmpD	Cytoplasm	Monomer	130.1	109.9	84.9	12.8	10.8	8.4
P0AE14	AmpE	Cell inner membrane	Monomer	109.2	92.2	71.2	8.6	7.3	5.6
			Complex with SecB	69.6	56.9	45.4	2.8	2.3	1.8
			Complex with TF (Tig)	76.2	64.4	49.7	3.6	3.0	2.3
P0AE16	AmpG	Cell inner membrane	Monomer	89.6	75.7	58.4	5.4	4.5	3.5
			Complex with SecB	64.6	52.8	42.2	2.3	1.9	1.5
			Complex with TF (Tig)	69.6	58.8	45.4	2.8	2.4	1.8
P0AD70	AmpH		Monomer	98.4	83.2	64.2	6.7	5.7	4.4
P69681	AmtB	Cell inner membrane	Monomer	96.1	81.2	62.7	6.4	5.4	4.2
			Complex with SecB	66.5	54.4	43.4	2.5	2.0	1.6

UniProtKB A. N.	Protein name	Subcellular location	Subunit	D_0 $\left(\frac{\mu m^2}{s}\right)$			D_{cyto} $\left(\frac{\mu m^2}{s}\right)$		
				310 K	303 K	293 K	310 K	303 K	293 K
			Complex with TF (Tig)	72.1	60.9	47.0	3.1	2.6	2.0
P26612	AmyA	Cytoplasm	Monomer	87.4	73.9	57.0	5.1	4.3	3.3
P77570	AnmK		Monomer	100.7	85.1	65.7	7.1	6.0	4.6
P0A962	AnsA	Cytoplasm	Monomer	103.2	87.2	67.3	7.5	6.4	4.9
			Homotetramer	59.9	48.9	39.1	1.9	1.5	1.2
P00805	AnsB	Periplasm	Monomer	103.5	87.4	67.5	7.6	6.4	5.0
			Complex with SecB	68.4	55.9	44.6	2.7	2.2	1.7
			Complex with TF (Tig)	74.5	63.0	48.6	3.4	2.8	2.2
P77610	AnsP	Cell inner membrane	Monomer	88.9	75.1	58.0	5.3	4.5	3.4
			Complex with SecB	64.4	52.6	42.0	2.3	1.9	1.5
			Complex with TF (Tig)	69.3	58.5	45.2	2.8	2.3	1.8
P62672	ApaG		Monomer	151.8	128.2	99.0	17.9	15.1	11.7
P05637	ApaH		Monomer	110.3	93.2	72.0	8.8	7.5	5.8
P0AB85	ApbE	Cell membrane	Monomer	101.7	85.9	66.3	7.3	6.2	4.8
			Complex with SecB	68.0	55.5	44.3	2.6	2.2	1.7
			Complex with TF (Tig)	74.0	62.5	48.3	3.3	2.8	2.2
P0AE22	AphA	Periplasm	Monomer	118.4	100.1	77.3	10.4	8.8	6.8
			Complex with SecB	71.3	58.3	46.5	3.0	2.4	2.0
			Complex with TF (Tig)	78.6	66.4	51.3	3.9	3.3	2.5
P07102	AppA	Periplasm	Monomer	94.0	79.4	61.3	6.0	5.1	3.9
			Complex with SecB	66.0	53.9	43.0	2.4	2.0	1.6
			Complex with TF (Tig)	71.3	60.2	46.5	3.0	2.5	2.0
P26458	AppB	Cell inner membrane	Monomer	97.9	82.7	63.9	6.7	5.6	4.3
			Complex with SecB	67.0	54.8	43.7	2.5	2.1	1.7
			Complex with TF (Tig)	72.7	61.4	47.4	3.2	2.7	2.1
P26459	AppC	Cell inner membrane	Monomer	86.7	73.2	56.5	4.9	4.2	3.2
			Complex with SecB	63.7	52.0	41.6	2.2	1.8	1.4
			Complex with TF (Tig)	68.3	57.7	44.6	2.7	2.3	1.7
P05052	AppY		Monomer	114.0	96.3	74.4	9.5	8.0	6.2
P69503	Apt	Cytoplasm	Monomer	131.8	111.4	86.0	13.2	11.2	8.6
			Homodimer	100.5	82.1	65.6	7.1	5.8	4.6

UniProtKB A. N.	Protein name	Subcellular location	Subunit	D_0 $\left(\frac{\mu m^2}{s}\right)$			D_{cyto} $\left(\frac{\mu m^2}{s}\right)$		
				310 K	303 K	293 K	310 K	303 K	293 K
P60844	AqpZ	Cell inner membrane	Monomer	123.0	103.9	80.3	11.3	9.6	7.4
			Complex with SecB	72.0	58.8	47.0	3.1	2.5	2.0
			Complex with TF (Tig)	79.6	67.3	52.0	4.0	3.4	2.6
P08202	AraA		Monomer	87.8	74.1	57.3	5.1	4.3	3.3
			Homohexamer	43.5	35.5	28.4	0.7	0.6	0.5
P08204	AraB		Monomer	84.9	71.7	55.4	4.7	4.0	3.1
P0A9E0	AraC	Cytoplasm	Monomer	107.5	90.9	70.2	8.3	7.0	5.4
			Homodimer	82.0	67.0	53.5	4.3	3.5	2.8
P08203	AraD		Monomer	119.5	100.9	78.0	10.6	9.0	6.9
			Homotetramer	69.4	56.7	45.3	2.8	2.3	1.8
P0AE24	AraE	Cell inner membrane	Monomer	90.6	76.6	59.1	5.5	4.7	3.6
			Complex with SecB	65.0	53.1	42.4	2.3	1.9	1.5
			Complex with TF (Tig)	70.0	59.1	45.7	2.9	2.4	1.9
P02924	AraF	Periplasm	Monomer	104.9	88.7	68.5	7.9	6.6	5.1
			Complex with SecB	68.7	56.1	44.8	2.7	2.2	1.8
			Complex with TF (Tig)	75.0	63.4	48.9	3.4	2.9	2.2
P0AAF3	AraG	Cell inner membrane	Monomer	88.4	74.7	57.7	5.2	4.4	3.4
			Complex with SecB	64.3	52.5	41.9	2.3	1.9	1.5
			Complex with TF (Tig)	69.1	58.4	45.1	2.8	2.3	1.8
P0AE26	AraH	Cell inner membrane	Monomer	106.5	90.0	69.5	8.1	6.9	5.3
			Complex with SecB	69.1	56.4	45.1	2.8	2.2	1.8
			Complex with TF (Tig)	75.5	63.8	49.2	3.5	2.9	2.3
P23910	AraJ	Cell inner membrane	Monomer	98.4	83.1	64.2	6.7	5.7	4.4
			Complex with SecB	67.1	54.9	43.8	2.6	2.1	1.7
			Complex with TF (Tig)	72.9	61.6	47.6	3.2	2.7	2.1
P0A9Q1	ArcA	Cytoplasm	Monomer	116.4	98.3	75.9	10.0	8.4	6.5
P0AEC3	ArcB	Cell inner membrane	Monomer	73.6	62.1	48.0	3.3	2.7	2.1
			Complex with SecB	58.6	47.9	38.2	1.8	1.4	1.1
			Complex with TF (Tig)	62.0	52.4	40.4	2.1	1.7	1.3
P37306	ArcC	Cytoplasm	Monomer	109.8	92.8	71.7	8.7	7.4	5.7
			Homodimer	83.7	68.4	54.6	4.5	3.7	3.0

UniProtKB A. N.	Protein name	Subcellular location	Subunit	D_0 $\left(\frac{\mu m^2}{s}\right)$			D_{cyto} $\left(\frac{\mu m^2}{s}\right)$		
				310 K	303 K	293 K	310 K	303 K	293 K
P0A6C5	ArgA	Cytoplasm	Monomer	92.4	78.0	60.3	5.8	4.9	3.8
			Homohexamer	45.8	37.4	29.9	0.9	0.7	0.6
P0A6C8	ArgB	Cytoplasm	Monomer	116.6	98.5	76.1	10.0	8.5	6.5
			Homodimer	88.9	72.6	58.0	5.3	4.3	3.4
P11446	ArgC	Cytoplasm	Monomer	104.5	88.3	68.2	7.8	6.6	5.1
P18335	ArgD	Cytoplasm	Monomer	96.7	81.7	63.1	6.5	5.5	4.2
			Homodimer	73.7	60.2	48.1	3.3	2.7	2.1
P23908	ArgE	Cytoplasm	Monomer	98.0	82.8	63.9	6.7	5.6	4.4
			Homodimer	74.7	61.0	48.7	3.4	2.8	2.2
P06960	ArgF	Cytoplasm	Monomer	103.5	87.4	67.5	7.6	6.4	5.0
P0A6E4	ArgG	Cytoplasm	Monomer	91.9	77.6	60.0	5.7	4.8	3.7
			Homotetramer	53.4	43.6	34.8	1.3	1.1	0.9
P11447	ArgH	Cytoplasm	Monomer	91.6	77.4	59.8	5.7	4.8	3.7
P04391	ArgI	Cytoplasm	Monomer	103.4	87.4	67.5	7.6	6.4	5.0
P27254	ArgK	Cell inner membrane	Monomer	103.6	87.5	67.6	7.6	6.4	5.0
			Complex with SecB	68.4	55.9	44.7	2.7	2.2	1.8
			Complex with TF (Tig)	74.6	63.0	48.7	3.4	2.8	2.2
P11667	ArgO	Cell inner membrane	Monomer	124.1	104.8	81.0	11.5	9.8	7.5
			Complex with SecB	72.2	59.0	47.1	3.1	2.5	2.0
			Complex with TF (Tig)	79.8	67.5	52.1	4.0	3.4	2.6
P0A6D0	ArgR	Cytoplasm	Monomer	140.1	118.4	91.4	15.1	12.8	9.9
			Homohexamer	69.4	56.7	45.3	2.8	2.3	1.8
P11875	ArgS	Cytoplasm	Monomer	83.0	70.1	54.2	4.4	3.7	2.9
P09551	ArgT	Periplasm	Monomer	115.2	97.4	75.2	9.8	8.2	6.4
			Complex with SecB	70.8	57.8	46.2	2.9	2.4	1.9
			Complex with TF (Tig)	77.8	65.8	50.8	3.8	3.2	2.5
P75993	AriR		Monomer	174.6	147.5	114.0	24.0	20.3	15.6
			Homodimer	133.1	108.7	86.8	13.5	11.0	8.8
P77398	ArnA		Monomer	78.6	66.4	51.3	3.9	3.3	2.5
			Homohexamer	38.9	31.8	25.4	0.5	0.4	0.3
P77690	ArnB		Monomer	98.6	83.3	64.4	6.8	5.7	4.4

UniProtKB A. N.	Protein name	Subcellular location	Subunit	D_0 $\left(\frac{\mu m^2}{s}\right)$			D_{cyto} $\left(\frac{\mu m^2}{s}\right)$		
				310 K	303 K	293 K	310 K	303 K	293 K
			Homodimer	75.2	61.4	49.0	3.4	2.8	2.2
P77757	ArnC	Cell inner membrane	Monomer	104.0	87.9	67.9	7.7	6.5	5.0
			Complex with SecB	68.5	56.0	44.7	2.7	2.2	1.8
			Complex with TF (Tig)	74.7	63.1	48.8	3.4	2.9	2.2
P76472	ArnD		Monomer	107.9	91.2	70.4	8.4	7.1	5.5
Q47377	ArnE	Cell inner membrane	Monomer	159.6	134.9	104.2	19.9	16.8	13.0
			Complex with SecB	75.9	62.0	49.5	3.5	2.9	2.3
			Complex with TF (Tig)	85.3	72.0	55.6	4.7	4.0	3.1
P76474	ArnF	Cell inner membrane	Monomer	150.8	127.4	98.4	17.7	14.9	11.5
			Complex with SecB	75.2	61.4	49.1	3.4	2.8	2.2
			Complex with TF (Tig)	84.2	71.2	55.0	4.6	3.9	3.0
P76473	ArnT	Cell inner membrane	Monomer	84.1	71.0	54.9	4.6	3.9	3.0
			Complex with SecB	62.8	51.3	41.0	2.1	1.7	1.4
			Complex with TF (Tig)	67.2	56.8	43.9	2.6	2.2	1.7
P0A6D3	AroA	Cytoplasm	Monomer	94.8	80.1	61.8	6.2	5.2	4.0
P07639	AroB	Cytoplasm	Monomer	101.3	85.6	66.1	7.2	6.1	4.7
P12008	AroC		Monomer	101.1	85.4	65.9	7.2	6.1	4.7
			Homotetramer	58.7	47.9	38.3	1.8	1.4	1.1
P05194	AroD		Monomer	116.1	98.1	75.8	9.9	8.4	6.5
			Homodimer	88.5	72.3	57.7	5.2	4.3	3.4
P15770	AroE		Monomer	113.0	95.5	73.8	9.3	7.9	6.1
P00888	AroF		Monomer	101.4	85.7	66.2	7.2	6.1	4.7
P0AB91	AroG		Monomer	102.2	86.4	66.7	7.4	6.2	4.8
			Homotetramer	59.4	48.5	38.7	1.8	1.5	1.2
P00887	AroH		Monomer	101.5	85.7	66.2	7.3	6.1	4.7
P0A6D7	AroK	Cytoplasm	Monomer	132.7	112.1	86.6	13.4	11.3	8.7
P0A6E1	AroL	Cytoplasm	Monomer	133.7	113.0	87.3	13.6	11.5	8.9
P0AE28	AroM		Monomer	120.6	101.9	78.7	10.8	9.2	7.1
P15993	AroP	Cell inner membrane	Monomer	92.0	77.7	60.0	5.7	4.8	3.7
			Complex with SecB	65.4	53.4	42.7	2.4	1.9	1.6
			Complex with TF (Tig)	70.5	59.6	46.0	2.9	2.5	1.9

UniProtKB A. N.	Protein name	Subcellular location	Subunit	D_0 $\left(\frac{\mu m^2}{s}\right)$			D_{cyto} $\left(\frac{\mu m^2}{s}\right)$		
				310 K	303 K	293 K	310 K	303 K	293 K
P23325	ArpA		Monomer	75.4	63.7	49.2	3.5	2.9	2.3
P76205	ArpB		Monomer	80.2	67.7	52.3	4.1	3.4	2.7
P78285	ArrD		Monomer	137.1	115.8	89.5	14.4	12.2	9.4
P76159	ArrQ		Monomer	132.3	111.8	86.4	13.3	11.3	8.7
P0AB93	ArsB	Cell inner membrane	Monomer	95.3	80.5	62.2	6.2	5.3	4.1
			Complex with SecB	66.3	54.2	43.3	2.5	2.0	1.6
			Complex with TF (Tig)	71.8	60.6	46.8	3.0	2.6	2.0
P0AB96	ArsC		Monomer	144.0	121.7	94.0	16.0	13.5	10.4
P37309	ArsR		Monomer	154.5	130.5	100.8	18.6	15.7	12.1
			Homodimer	117.7	96.2	76.8	10.3	8.4	6.7
P30859	ArtI	Periplasm	Monomer	117.0	98.8	76.3	10.1	8.5	6.6
			Complex with SecB	71.1	58.1	46.4	3.0	2.4	1.9
			Complex with TF (Tig)	78.3	66.1	51.1	3.8	3.2	2.5
P30860	ArtJ	Periplasm	Monomer	117.2	99.0	76.5	10.1	8.6	6.6
			Complex with SecB	71.1	58.1	46.4	3.0	2.4	1.9
			Complex with TF (Tig)	78.3	66.1	51.1	3.8	3.2	2.5
P0AE30	ArtM	Cell inner membrane	Monomer	120.6	101.9	78.7	10.8	9.2	7.1
			Complex with SecB	71.7	58.5	46.8	3.0	2.5	2.0
			Complex with TF (Tig)	79.1	66.8	51.6	3.9	3.3	2.6
P0AAF6	ArtP	Cell inner membrane	Monomer	116.8	98.7	76.2	10.1	8.5	6.6
			Complex with SecB	71.0	58.0	46.4	3.0	2.4	1.9
			Complex with TF (Tig)	78.2	66.1	51.0	3.8	3.2	2.5
P0AE34	ArtQ	Cell inner membrane	Monomer	118.2	99.9	77.2	10.4	8.7	6.8
			Complex with SecB	71.3	58.2	46.5	3.0	2.4	2.0
			Complex with TF (Tig)	78.6	66.4	51.3	3.9	3.3	2.5
P24240	AscB		Monomer	89.1	75.3	58.2	5.3	4.5	3.5
P24241	AscF	Cell inner membrane	Monomer	91.1	76.9	59.4	5.6	4.7	3.6
			Complex with SecB	65.1	53.2	42.5	2.3	1.9	1.5
			Complex with TF (Tig)	70.2	59.3	45.8	2.9	2.4	1.9
P24242	AscG		Monomer	103.4	87.3	67.4	7.6	6.4	4.9
P0A9Q9	Asd		Monomer	100.2	84.6	65.4	7.0	5.9	4.6

UniProtKB A. N.	Protein name	Subcellular location	Subunit	D_0 $\left(\frac{\mu m^2}{s}\right)$			D_{cyto} $\left(\frac{\mu m^2}{s}\right)$		
				310 K	303 K	293 K	310 K	303 K	293 K
			Homodimer	76.3	62.4	49.8	3.6	2.9	2.3
P25549	AslA	Periplasm	Monomer	85.1	71.9	55.5	4.7	4.0	3.1
			Complex with SecB	63.2	51.6	41.2	2.2	1.8	1.4
			Complex with TF (Tig)	67.7	57.2	44.1	2.6	2.2	1.7
P25550	AslB		Monomer	94.4	79.8	61.6	6.1	5.2	4.0
P28249	AsmA	Periplasm	Monomer	80.9	68.4	52.8	4.2	3.5	2.7
			Complex with SecB	61.6	50.4	40.2	2.0	1.7	1.3
			Complex with TF (Tig)	65.7	55.5	42.9	2.4	2.0	1.6
P00963	AsnA	Cytoplasm	Monomer	103.7	87.6	67.7	7.6	6.5	5.0
			Homodimer	79.0	64.5	51.6	3.9	3.2	2.6
P22106	AsnB		Monomer	84.0	71.0	54.8	4.6	3.9	3.0
			Homodimer	64.0	52.3	41.8	2.2	1.8	1.5
P0ACI6	AsnC		Monomer	140.5	118.7	91.7	15.2	12.8	9.9
P0A8M0	AsnS	Cytoplasm	Monomer	90.0	76.0	58.7	5.4	4.6	3.5
			Homodimer	68.6	56.0	44.8	2.7	2.2	1.8
P0AC38	AspA		Monomer	90.2	76.2	58.8	5.5	4.6	3.6
			Homotetramer	52.4	42.8	34.2	1.3	1.0	0.8
P00509	AspC	Cytoplasm	Monomer	96.9	81.9	63.2	6.5	5.5	4.2
			Homodimer	73.8	60.3	48.2	3.3	2.7	2.1
P21889	AspS	Cytoplasm	Monomer	82.4	69.6	53.8	4.4	3.7	2.8
			Homodimer	62.8	51.3	41.0	2.1	1.7	1.4
P36560	Asr	Periplasm	Monomer	168.7	142.5	110.1	22.3	18.9	14.6
			Complex with SecB	76.5	62.5	49.9	3.6	2.9	2.3
			Complex with TF (Tig)	86.2	72.8	56.2	4.9	4.1	3.2
P0AE37	AstA		Monomer	101.7	86.0	66.4	7.3	6.2	4.8
P76216	AstB		Monomer	92.3	78.0	60.2	5.8	4.9	3.8
			Homodimer	70.3	57.5	45.9	2.9	2.4	1.9
P77581	AstC		Monomer	96.8	81.8	63.2	6.5	5.5	4.2
P76217	AstD		Monomer	89.7	75.8	58.5	5.4	4.5	3.5
P76215	AstE		Monomer	104.6	88.4	68.3	7.8	6.6	5.1
P76459	AtoA		Monomer	124.5	105.2	81.3	11.6	9.8	7.6

UniProtKB A. N.	Protein name	Subcellular location	Subunit	D_0 $\left(\frac{\mu m^2}{s}\right)$			D_{cyto} $\left(\frac{\mu m^2}{s}\right)$		
				310 K	303 K	293 K	310 K	303 K	293 K
			Heterodimer (AtoA−AtoD)	94.5	79.8	61.6	6.1	5.2	4.0
P76461	AtoB	Cytoplasm	Monomer	99.8	84.4	65.2	7.0	5.9	4.6
Q06065	AtoC	Cytoplasm	Monomer	90.3	76.3	58.9	5.5	4.6	3.6
P76458	AtoD		Monomer	123.4	104.2	80.5	11.4	9.6	7.4
			Heterodimer (AtoA−AtoD)	94.5	79.8	61.6	6.1	5.2	4.0
P76460	AtoE	Cell inner membrane	Monomer	93.6	79.1	61.1	6.0	5.1	3.9
			Complex with SecB	65.9	53.8	43.0	2.4	2.0	1.6
			Complex with TF (Tig)	71.2	60.1	46.4	3.0	2.5	1.9
Q06067	AtoS	Cell inner membrane	Monomer	81.5	68.8	53.2	4.2	3.6	2.8
			Complex with SecB	61.9	50.5	40.4	2.0	1.7	1.3
			Complex with TF (Tig)	66.0	55.8	43.1	2.4	2.1	1.6
P0ABB0	AtpA	Cell inner membrane	Monomer	88.3	74.6	57.6	5.2	4.4	3.4
			Complex with SecB	64.2	52.5	41.9	2.3	1.8	1.5
			Complex with TF (Tig)	69.0	58.3	45.1	2.8	2.3	1.8
P0AB98	AtpB	Cell inner membrane	Monomer	111.7	94.4	72.9	9.1	7.7	5.9
			Complex with SecB	70.1	57.3	45.8	2.9	2.3	1.9
			Complex with TF (Tig)	76.9	65.0	50.2	3.7	3.1	2.4
P0A6E6	AtpC	Cell inner membrane	Monomer	146.9	124.1	95.9	16.7	14.1	10.9
			Complex with SecB	74.8	61.1	48.8	3.4	2.8	2.2
			Complex with TF (Tig)	83.7	70.7	54.6	4.5	3.8	3.0
P0ABB4	AtpD	Cell inner membrane	Monomer	91.6	77.4	59.8	5.7	4.8	3.7
			Complex with SecB	65.3	53.3	42.6	2.4	1.9	1.5
			Complex with TF (Tig)	70.4	59.4	45.9	2.9	2.4	1.9
P68699	AtpE	Cell inner membrane	Monomer	186.0	157.1	121.4	27.2	23.0	17.8
			Complex with SecB	77.4	63.2	50.5	3.7	3.0	2.4
			Complex with TF (Tig)	87.5	74.0	57.1	5.1	4.3	3.3
P0ABA0	AtpF	Cell inner membrane	Monomer	139.3	117.7	90.9	14.9	12.6	9.7
			Complex with SecB	74.1	60.5	48.3	3.3	2.7	2.2
			Complex with TF (Tig)	82.6	69.8	53.9	4.4	3.7	2.9
P0ABA6	AtpG	Cell inner membrane	Monomer	109.9	92.9	71.7	8.8	7.4	5.7
			Complex with SecB	69.8	57.0	45.5	2.8	2.3	1.8

UniProtKB A. N.	Protein name	Subcellular location	Subunit	$D_0\left(\frac{\mu m^2}{s}\right)$			$D_{\text{cyto}}\left(\frac{\mu m^2}{s}\right)$		
				310 K	303 K	293 K	310 K	303 K	293 K
			Complex with TF (Tig)	76.4	64.6	49.9	3.6	3.0	2.3
P0ABA4	AtpH	Cell inner membrane	Monomer	133.2	112.6	86.9	13.5	11.4	8.8
			Complex with SecB	73.4	60.0	47.9	3.2	2.6	2.1
			Complex with TF (Tig)	81.6	68.9	53.2	4.2	3.6	2.8
P0ABC0	AtpI	Cell inner membrane	Monomer	152.8	129.1	99.7	18.2	15.3	11.9
			Complex with SecB	75.4	61.6	49.2	3.5	2.8	2.3
			Complex with TF (Tig)	84.5	71.4	55.1	4.6	3.9	3.0
P09053	AvtA	Cytoplasm	Monomer	94.3	79.6	61.5	6.1	5.1	4.0
			Homodimer	71.8	58.7	46.9	3.1	2.5	2.0
P41407	AzoR		Monomer	127.4	107.7	83.2	12.3	10.4	8.0
			Homodimer	97.1	79.3	63.4	6.5	5.3	4.3
C1P605	AzuC	Membrane	Monomer	263.1	222.3	171.7	53.0	44.8	34.6
			Complex with SecB	79.3	64.8	51.8	4.0	3.2	2.6
			Complex with TF (Tig)	90.7	76.6	59.2	5.5	4.7	3.6
P75688	Unnamed		Monomer	189.7	160.2	123.8	28.3	23.9	18.5
P75971	Unnamed	Cell membrane	Monomer	166.4	140.6	108.6	21.7	18.3	14.2
			Complex with SecB	76.3	62.4	49.8	3.6	2.9	2.3
			Complex with TF (Tig)	85.9	72.6	56.1	4.8	4.1	3.2
P11291	Unnamed		Monomer	139.0	117.5	90.7	14.8	12.5	9.7
P08339	Unnamed		Monomer	168.7	142.5	110.1	22.3	18.9	14.6
Q47719	Unnamed		Monomer	146.9	124.1	95.9	16.7	14.1	10.9
P69228	BaeR	Cytoplasm	Monomer	115.8	97.8	75.6	9.9	8.3	6.4
P30847	BaeS	Cell inner membrane	Monomer	90.4	76.4	59.0	5.5	4.6	3.6
			Complex with SecB	64.9	53.0	42.4	2.3	1.9	1.5
			Complex with TF (Tig)	69.9	59.1	45.6	2.8	2.4	1.9
P0AEC5	BarA	Cell inner membrane	Monomer	69.3	58.5	45.2	2.8	2.3	1.8
			Complex with SecB	56.6	46.3	36.9	1.6	1.3	1.0
			Complex with TF (Tig)	59.6	50.3	38.9	1.8	1.6	1.2
P30843	BasR	Cytoplasm	Monomer	120.4	101.7	78.6	10.8	9.1	7.0
			Homodimer	91.8	75.0	59.9	5.7	4.6	3.7
P30844	BasS	Cell inner membrane	Monomer	99.2	83.8	64.7	6.9	5.8	4.5

UniProtKB A. N.	Protein name	Subcellular location	Subunit	D_0 $\left(\frac{\mu m^2}{s}\right)$			D_{cyto} $\left(\frac{\mu m^2}{s}\right)$		
				310 K	303 K	293 K	310 K	303 K	293 K
			Complex with SecB	67.4	55.0	44.0	2.6	2.1	1.7
			Complex with TF (Tig)	73.2	61.8	47.7	3.2	2.7	2.1
P27297	Bax		Monomer	110.9	93.7	72.4	8.9	7.6	5.8
P0AE52	Bcp		Monomer	138.1	116.7	90.1	14.6	12.4	9.5
P28246	Bcr	Cell inner membrane	Monomer	97.1	82.0	63.3	6.5	5.5	4.3
			Complex with SecB	66.8	54.6	43.6	2.5	2.1	1.6
			Complex with TF (Tig)	72.4	61.2	47.3	3.1	2.6	2.0
P37653	BcsA	Cell inner membrane	Monomer	70.0	59.2	45.7	2.9	2.4	1.9
			Complex with SecB	57.0	46.5	37.2	1.6	1.3	1.1
			Complex with TF (Tig)	60.0	50.7	39.1	1.9	1.6	1.2
P37652	BcsB	Cell inner membrane	Monomer	74.2	62.7	48.4	3.3	2.8	2.2
			Complex with SecB	58.9	48.1	38.4	1.8	1.5	1.2
			Complex with TF (Tig)	62.3	52.7	40.7	2.1	1.8	1.4
P37650	BcsC		Monomer	63.6	53.7	41.5	2.2	1.9	1.4
P37651	BcsZ	Secreted	Monomer	98.6	83.3	64.3	6.8	5.7	4.4
			Complex with SecB	67.2	54.9	43.9	2.6	2.1	1.7
			Complex with TF (Tig)	72.9	61.6	47.6	3.2	2.7	2.1
P76127	Bdm		Monomer	188.4	159.2	122.9	27.9	23.6	18.2
P17444	BetA	Cell membrane	Monomer	84.4	71.3	55.1	4.6	3.9	3.0
			Complex with SecB	62.9	51.4	41.1	2.1	1.7	1.4
			Complex with TF (Tig)	67.4	56.9	44.0	2.6	2.2	1.7
P17445	BetB		Monomer	89.8	75.9	58.6	5.4	4.6	3.5
			Homotetramer	52.1	42.6	34.0	1.3	1.0	0.8
P17446	BetI		Monomer	127.1	107.4	82.9	12.2	10.3	7.9
P0ABC9	BetT	Cell inner membrane	Monomer	78.0	65.9	50.9	3.8	3.2	2.5
			Complex with SecB	60.5	49.4	39.5	1.9	1.6	1.3
			Complex with TF (Tig)	64.3	54.3	42.0	2.3	1.9	1.5
P0AE56	Bfd		Monomer	194.5	164.3	126.9	29.8	25.2	19.4
P0ABD3	Bfr		Monomer	135.6	114.5	88.5	14.0	11.9	9.2
Q46829	BglA		Monomer	88.2	74.5	57.6	5.2	4.4	3.4
P11988	BglB		Monomer	89.6	75.7	58.5	5.4	4.5	3.5

UniProtKB A. N.	Protein name	Subcellular location	Subunit	$D_0 \left(\frac{\mu m^2}{s}\right)$			$D_{\text{cyto}} \left(\frac{\mu m^2}{s}\right)$		
				310 K	303 K	293 K	310 K	303 K	293 K
P08722	BglF	Cell inner membrane	Monomer	82.1	69.4	53.6	4.3	3.6	2.8
			Complex with SecB	62.1	50.7	40.5	2.1	1.7	1.3
			Complex with TF (Tig)	66.3	56.0	43.3	2.5	2.1	1.6
P11989	BglG		Monomer	109.2	92.3	71.3	8.6	7.3	5.6
P26218	BglH	Cell outer membrane	Monomer	85.1	71.9	55.5	4.7	4.0	3.1
			Complex with SecB	63.2	51.6	41.2	2.2	1.8	1.4
			Complex with TF (Tig)	67.7	57.2	44.2	2.6	2.2	1.7
P39404	BglJ		Monomer	119.3	100.8	77.9	10.6	8.9	6.9
P33363	BglX	Periplasm	Monomer	75.1	63.4	49.0	3.4	2.9	2.2
			Complex with SecB	59.3	48.4	38.7	1.8	1.5	1.2
			Complex with TF (Tig)	62.8	53.1	41.0	2.1	1.8	1.4
P0AB40	BhsA	Periplasm	Monomer	181.3	153.1	118.3	25.9	21.8	16.9
			Complex with SecB	77.1	63.0	50.3	3.7	3.0	2.4
			Complex with TF (Tig)	87.2	73.7	56.9	5.0	4.2	3.3
P12995	BioA	Cytoplasm	Monomer	93.8	79.2	61.2	6.0	5.1	3.9
			Homodimer	71.5	58.4	46.6	3.0	2.5	2.0
P12996	BioB		Monomer	101.5	85.8	66.3	7.3	6.1	4.7
			Homodimer	77.4	63.2	50.5	3.7	3.0	2.4
P12999	BioC		Monomer	114.8	97.0	74.9	9.7	8.2	6.3
P13000	BioD1	Cytoplasm	Monomer	122.1	103.2	79.7	11.1	9.4	7.3
			Homodimer	93.1	76.0	60.7	5.9	4.8	3.8
P0A6E9	BioD2	Cytoplasm	Monomer	120.5	101.8	78.6	10.8	9.1	7.1
			Homodimer	91.8	75.0	59.9	5.7	4.7	3.7
P12998	BioF		Monomer	98.7	83.4	64.4	6.8	5.7	4.4
			Homodimer	75.2	61.4	49.1	3.4	2.8	2.2
P13001	BioH	Cytoplasm	Monomer	114.4	96.7	74.7	9.6	8.1	6.3
P06709	BirA		Monomer	105.2	88.9	68.7	7.9	6.7	5.2
P20099	BisC		Monomer	74.3	62.7	48.5	3.3	2.8	2.2
P0A901	Blc	Cell outer membrane	Monomer	131.9	111.4	86.0	13.2	11.2	8.6
			Complex with SecB	73.2	59.8	47.8	3.2	2.6	2.1
			Complex with TF (Tig)	81.4	68.7	53.1	4.2	3.6	2.8

UniProtKB A. N.	Protein name	Subcellular location	Subunit	D_0 $\left(\frac{\mu m^2}{s}\right)$			D_{cyto} $\left(\frac{\mu m^2}{s}\right)$		
				310 K	303 K	293 K	310 K	303 K	293 K
P56976	Blr	Cell inner membrane	Monomer	234.8	198.3	153.2	42.9	36.2	28.0
			Complex with SecB	78.9	64.4	51.5	3.9	3.2	2.5
			Complex with TF (Tig)	89.9	75.9	58.7	5.4	4.6	3.5
P0ABE2	BolA		Monomer	160.6	135.7	104.8	20.2	17.1	13.2
P77330	BorD	Cell membrane	Monomer	169.6	143.3	110.7	22.6	19.1	14.7
			Complex with SecB	76.5	62.5	49.9	3.6	2.9	2.4
			Complex with TF (Tig)	86.2	72.9	56.3	4.9	4.1	3.2
P0AD99	BrnQ	Cell inner membrane	Monomer	94.7	80.0	61.8	6.1	5.2	4.0
			Complex with SecB	66.2	54.0	43.2	2.5	2.0	1.6
			Complex with TF (Tig)	71.6	60.5	46.7	3.0	2.6	2.0
P39297	BsmA	Cell membrane	Monomer	160.4	135.5	104.7	20.1	17.0	13.1
			Complex with SecB	75.9	62.0	49.5	3.5	2.9	2.3
			Complex with TF (Tig)	85.3	72.1	55.7	4.8	4.0	3.1
P0AAY1	BssR		Monomer	149.0	125.9	97.2	17.2	14.5	11.2
P0AB33	BssS		Monomer	174.8	147.7	114.1	24.0	20.3	15.7
P06129	BtuB	Cell outer membrane	Monomer	81.2	68.6	53.0	4.2	3.5	2.7
			Complex with SecB	61.7	50.4	40.3	2.0	1.7	1.3
			Complex with TF (Tig)	65.9	55.6	43.0	2.4	2.0	1.6
P06609	BtuC	Cell inner membrane	Monomer	105.6	89.2	68.9	8.0	6.7	5.2
			Complex with SecB	68.9	56.3	44.9	2.7	2.2	1.8
			Complex with TF (Tig)	75.2	63.5	49.1	3.4	2.9	2.2
P06611	BtuD	Cell inner membrane	Monomer	116.7	98.6	76.2	10.1	8.5	6.6
			Complex with SecB	71.0	58.0	46.4	3.0	2.4	1.9
			Complex with TF (Tig)	78.2	66.1	51.0	3.8	3.2	2.5
P06610	BtuE	Periplasm	Monomer	130.3	110.1	85.0	12.9	10.9	8.4
			Complex with SecB	73.0	59.7	47.7	3.2	2.6	2.1
			Complex with TF (Tig)	81.1	68.5	52.9	4.2	3.5	2.7
P37028	BtuF	Periplasm	Monomer	113.1	95.5	73.8	9.4	7.9	6.1
			Complex with SecB	70.4	57.5	45.9	2.9	2.4	1.9
			Complex with TF (Tig)	77.3	65.3	50.4	3.7	3.1	2.4
P0A9H5	BtuR	Cytoplasm	Monomer	126.7	107.0	82.6	12.1	10.2	7.9

UniProtKB A. N.	Protein name	Subcellular location	Subunit	D_0 $\left(\frac{\mu m^2}{s}\right)$			D_{cyto} $\left(\frac{\mu m^2}{s}\right)$		
				310 K	303 K	293 K	310 K	303 K	293 K
P0A9H3	CadA	Cytoplasm	Monomer	75.9	64.1	49.5	3.5	3.0	2.3
			Homodimer	57.8	47.2	37.7	1.7	1.4	1.1
			Homodecamer	30.8	26.0	20.1	0.2	0.2	0.2
P0AAE8	CadB	Cell inner membrane	Monomer	94.3	79.7	61.5	6.1	5.1	4.0
			Complex with SecB	66.1	54.0	43.1	2.4	2.0	1.6
			Complex with TF (Tig)	71.4	60.3	46.6	3.0	2.5	2.0
P23890	CadC	Cell membrane	Monomer	86.7	73.3	56.6	5.0	4.2	3.2
			Complex with SecB	63.7	52.1	41.6	2.2	1.8	1.4
			Complex with TF (Tig)	68.4	57.8	44.6	2.7	2.3	1.7
P60584	CaiA		Monomer	97.8	82.6	63.8	6.6	5.6	4.3
			Homotetramer	56.8	46.4	37.1	1.6	1.3	1.0
P31572	CaiB	Cytoplasm	Monomer	95.6	80.7	62.4	6.3	5.3	4.1
			Homodimer	72.8	59.5	47.5	3.2	2.6	2.1
P31552	CaiC		Monomer	86.3	72.9	56.3	4.9	4.1	3.2
P31551	CaiD		Monomer	114.9	97.1	75.0	9.7	8.2	6.3
P39206	CaiE		Monomer	128.4	108.5	83.8	12.5	10.5	8.1
P0AE58	CaiF		Monomer	145.5	122.9	95.0	16.4	13.8	10.7
P31553	CaiT	Cell inner membrane	Monomer	87.5	73.9	57.1	5.1	4.3	3.3
			Complex with SecB	64.0	52.3	41.7	2.2	1.8	1.5
			Complex with TF (Tig)	68.7	58.0	44.8	2.7	2.3	1.8
P61517	Can		Monomer	120.3	101.6	78.5	10.8	9.1	7.0
			Homodimer	91.7	74.9	59.8	5.7	4.6	3.7
P0A6F1	CarA		Monomer	98.8	83.5	64.5	6.8	5.8	4.4
			Heterotetramer ($CarA_2-CarB_2$)	44.4	37.5	29.0	0.8	0.7	0.5
P00968	CarB		Monomer	65.6	55.4	42.8	2.4	2.0	1.6
			Heterotetramer ($CarA_2-CarB_2$)	44.4	37.5	29.0	0.8	0.7	0.5
Q47083	Cbl		Monomer	104.6	88.4	68.2	7.8	6.6	5.1
P36659	CbpA	Cytoplasm > nucleoid	Monomer	106.2	89.7	69.3	8.1	6.8	5.3
P63264	CbpM		Monomer	163.3	137.9	106.5	20.9	17.6	13.6
P31456	CbrA		Monomer	100.1	84.5	65.3	7.0	5.9	4.6

UniProtKB A. N.	Protein name	Subcellular location	Subunit	$D_0 \left(\frac{\mu m^2}{s}\right)$			$D_{\text{cyto}} \left(\frac{\mu m^2}{s}\right)$		
				310 K	303 K	293 K	310 K	303 K	293 K
P31468	CbrB	Cell inner membrane	Monomer	141.2	119.3	92.1	15.3	13.0	10.0
			Complex with SecB	74.3	60.7	48.5	3.3	2.7	2.2
			Complex with TF (Tig)	82.9	70.0	54.1	4.4	3.7	2.9
P31469	CbrC		Monomer	125.6	106.1	81.9	11.9	10.0	7.7
P64524	CbtA		Monomer	151.6	128.1	98.9	17.9	15.1	11.7
P06961	Cca		Monomer	94.5	79.8	61.6	6.1	5.2	4.0
			Homodimer	72.0	58.8	47.0	3.1	2.5	2.0
P33931	CcmA	Cell inner membrane	Monomer	124.3	105.1	81.1	11.6	9.8	7.6
			Complex with SecB	72.2	59.0	47.1	3.1	2.5	2.0
			Complex with TF (Tig)	79.9	67.5	52.1	4.0	3.4	2.6
P0ABL8	CcmB	Cell inner membrane	Monomer	123.2	104.1	80.4	11.4	9.6	7.4
			Complex with SecB	72.1	58.9	47.0	3.1	2.5	2.0
			Complex with TF (Tig)	79.7	67.3	52.0	4.0	3.4	2.6
P0ABM1	CcmC	Cell inner membrane	Monomer	115.4	97.5	75.3	9.8	8.3	6.4
			Complex with SecB	70.8	57.8	46.2	2.9	2.4	1.9
			Complex with TF (Tig)	77.9	65.8	50.8	3.8	3.2	2.5
P0ABM5	CcmD	Cell inner membrane	Monomer	190.7	161.1	124.4	28.6	24.2	18.7
			Complex with SecB	77.6	63.4	50.6	3.7	3.0	2.4
			Complex with TF (Tig)	87.8	74.2	57.3	5.1	4.3	3.3
P69490	CcmE	Cell inner membrane	Monomer	137.9	116.5	90.0	14.6	12.3	9.5
			Complex with SecB	73.9	60.4	48.3	3.3	2.7	2.2
			Complex with TF (Tig)	82.4	69.6	53.8	4.4	3.7	2.8
P33927	CcmF	Cell inner membrane	Monomer	79.8	67.4	52.1	4.0	3.4	2.6
			Complex with SecB	61.2	50.0	40.0	2.0	1.6	1.3
			Complex with TF (Tig)	65.2	55.1	42.6	2.4	2.0	1.5
P0ABM9	CcmH	Cell inner membrane	Monomer	101.1	85.4	66.0	7.2	6.1	4.7
			Complex with SecB	67.8	55.4	44.3	2.6	2.1	1.7
			Complex with TF (Tig)	73.8	62.3	48.2	3.3	2.8	2.1
P37047	CdaR		Monomer	96.8	81.8	63.2	6.5	5.5	4.2
P0ABF6	Cdd		Monomer	110.0	92.9	71.8	8.8	7.4	5.7
			Homodimer	83.8	68.5	54.7	4.5	3.7	3.0

UniProtKB A. N.	Protein name	Subcellular location	Subunit	D_0 $\left(\frac{\mu m^2}{s}\right)$			D_{cyto} $\left(\frac{\mu m^2}{s}\right)$		
				310 K	303 K	293 K	310 K	303 K	293 K
P06282	Cdh	Cell inner membrane	Monomer	114.5	96.7	74.7	9.6	8.1	6.3
			Complex with SecB	70.6	57.7	46.1	2.9	2.4	1.9
			Complex with TF (Tig)	77.6	65.6	50.7	3.7	3.2	2.4
P0ABG1	CdsA	Cell inner membrane	Monomer	110.1	93.0	71.8	8.8	7.4	5.7
			Complex with SecB	69.8	57.0	45.6	2.8	2.3	1.8
			Complex with TF (Tig)	76.5	64.6	49.9	3.6	3.0	2.3
P0AE60	CedA		Monomer	176.9	149.5	115.5	24.6	20.8	16.1
P0A9H7	Cfa	Cytoplasm	Monomer	96.6	81.6	63.0	6.4	5.4	4.2
P31801	ChaA	Cell inner membrane	Monomer	101.0	85.3	65.9	7.2	6.1	4.7
			Complex with SecB	67.8	55.4	44.2	2.6	2.1	1.7
			Complex with TF (Tig)	73.8	62.3	48.1	3.3	2.8	2.1
P0AE63	ChaB		Monomer	180.2	152.3	117.6	25.6	21.6	16.7
P39163	ChaC		Monomer	119.5	101.0	78.0	10.6	9.0	6.9
P69791	ChbA	Cytoplasm	Monomer	156.9	132.5	102.4	19.2	16.2	12.5
			Homodimer	119.5	97.7	78.0	10.6	8.7	6.9
P69795	ChbB	Cytoplasm	Monomer	163.7	138.3	106.8	21.0	17.7	13.7
P17334	ChbC	Cell inner membrane	Monomer	93.0	78.6	60.7	5.9	5.0	3.8
			Complex with SecB	65.7	53.7	42.9	2.4	2.0	1.6
			Complex with TF (Tig)	70.9	59.9	46.3	3.0	2.5	1.9
P17411	ChbF		Monomer	91.4	77.2	59.7	5.6	4.8	3.7
			Homotetramer	53.1	43.4	34.7	1.3	1.1	0.9
P37794	ChbG		Monomer	115.6	97.7	75.4	9.8	8.3	6.4
P17410	ChbR		Monomer	108.1	91.3	70.5	8.4	7.1	5.5
			Homodimer	82.4	67.3	53.7	4.4	3.6	2.8
P07363	CheA	Cytoplasm	Monomer	79.8	67.5	52.1	4.0	3.4	2.6
P07330	CheB	Cytoplasm	Monomer	102.8	86.8	67.1	7.5	6.3	4.9
P07364	CheR		Monomer	108.2	91.4	70.6	8.4	7.1	5.5
P0A964	CheW	Cytoplasm	Monomer	136.8	115.5	89.2	14.3	12.1	9.3
P0AE67	CheY	Cytoplasm	Monomer	150.8	127.4	98.4	17.7	14.9	11.5
P0A9H9	CheZ	Cytoplasm	Monomer	122.5	103.4	79.9	11.2	9.5	7.3
			Homodimer	93.3	76.2	60.9	5.9	4.8	3.9

UniProtKB A. N.	Protein name	Subcellular location	Subunit	D_0 $\left(\frac{\mu m^2}{s}\right)$ 310 K	D_0 303 K	D_0 293 K	D_{cyto} $\left(\frac{\mu m^2}{s}\right)$ 310 K	D_{cyto} 303 K	D_{cyto} 293 K
P13656	ChiA	Periplasm	Monomer	70.8	59.8	46.2	2.9	2.5	1.9
			Complex with SecB	57.3	46.8	37.4	1.6	1.3	1.1
			Complex with TF (Tig)	60.4	51.1	39.4	1.9	1.6	1.2
P76213	Cho		Monomer	107.1	90.5	69.9	8.2	7.0	5.4
P33647	ChpB		Monomer	158.1	133.6	103.2	19.5	16.5	12.7
			Homodimer	120.5	98.4	78.6	10.8	8.8	7.1
P08365	ChpS		Monomer	177.7	150.1	116.0	24.8	21.0	16.2
P17315	CirA	Cell outer membrane	Monomer	78.8	66.5	51.4	3.9	3.3	2.5
			Complex with SecB	60.8	49.7	39.7	1.9	1.6	1.3
			Complex with TF (Tig)	64.7	54.7	42.2	2.3	1.9	1.5
P77390	CitC		Monomer	100.1	84.6	65.3	7.0	5.9	4.6
P69330	CitD	Cytoplasm	Monomer	168.1	142.0	109.7	22.2	18.7	14.5
P0A9I1	CitE	Cytoplasm	Monomer	107.9	91.2	70.4	8.4	7.1	5.5
P75726	CitF	Cytoplasm	Monomer	88.3	74.6	57.6	5.2	4.4	3.4
P77231	CitG		Monomer	109.8	92.8	71.7	8.7	7.4	5.7
P0AE74	CitT	Cell inner membrane	Monomer	89.7	75.7	58.5	5.4	4.5	3.5
			Complex with SecB	64.7	52.8	42.2	2.3	1.9	1.5
			Complex with TF (Tig)	69.6	58.8	45.4	2.8	2.4	1.8
P0A6G5	CitX		Monomer	130.8	110.5	85.3	13.0	11.0	8.5
P37019	ClcA	Cell inner membrane	Monomer	91.5	77.3	59.7	5.7	4.8	3.7
			Complex with SecB	65.2	53.3	42.6	2.4	1.9	1.5
			Complex with TF (Tig)	70.4	59.4	45.9	2.9	2.4	1.9
P76175	ClcB	Cell inner membrane	Monomer	96.4	81.4	62.9	6.4	5.4	4.2
			Complex with SecB	66.6	54.4	43.5	2.5	2.0	1.6
			Complex with TF (Tig)	72.2	61.0	47.1	3.1	2.6	2.0
P0ABH9	ClpA		Monomer	74.8	63.2	48.8	3.4	2.9	2.2
P63284	ClpB	Cytoplasm	Monomer	71.2	60.2	46.5	3.0	2.5	1.9
			Homohexamer	35.3	28.8	23.0	0.4	0.3	0.2
P0A6G7	ClpP	Cytoplasm	Monomer	124.1	104.8	81.0	11.5	9.8	7.5
			Heterodimer (ClpP−ClpX)	80.7	68.1	52.6	4.1	3.5	2.7
P0A8Q6	ClpS		Monomer	159.7	134.9	104.2	19.9	16.8	13.0

UniProtKB A. N.	Protein name	Subcellular location	Subunit	D_0 $\left(\frac{\mu m^2}{s}\right)$			D_{cyto} $\left(\frac{\mu m^2}{s}\right)$		
				310 K	303 K	293 K	310 K	303 K	293 K
P0A6H1	ClpX		Monomer	94.6	79.9	61.7	6.1	5.2	4.0
			Heterodimer (ClpP−ClpX)	80.7	68.1	52.6	4.1	3.5	2.7
P0A6H8	Cls	Cell inner membrane	Monomer	88.5	74.8	57.8	5.2	4.4	3.4
			Complex with SecB	64.3	52.5	42.0	2.3	1.9	1.5
			Complex with TF (Tig)	69.1	58.4	45.1	2.8	2.3	1.8
P0A6I0	Cmk	Cytoplasm	Monomer	120.9	102.2	78.9	10.9	9.2	7.1
P76290	CmoA		Monomer	115.6	97.6	75.4	9.8	8.3	6.4
P76291	CmoB		Monomer	103.3	87.3	67.4	7.6	6.4	4.9
P69826	CmtA	Cell inner membrane	Monomer	92.6	78.2	60.4	5.8	4.9	3.8
			Complex with SecB	65.5	53.5	42.8	2.4	2.0	1.6
			Complex with TF (Tig)	70.8	59.8	46.2	2.9	2.5	1.9
P69824	CmtB	Cytoplasm	Monomer	143.3	121.1	93.5	15.8	13.4	10.3
P64467	Cnu		Monomer	184.6	155.9	120.4	26.8	22.7	17.5
P0A6I3	CoaA	Cytoplasm	Monomer	104.0	87.9	67.9	7.7	6.5	5.0
P0ABQ0	CoaBC		Monomer	97.0	82.0	63.3	6.5	5.5	4.3
			Homododecamer	36.6	29.9	23.9	0.4	0.3	0.3
P0A6I6	CoaD	Cytoplasm	Monomer	137.5	116.2	89.7	14.5	12.2	9.5
			Homohexamer	68.1	55.7	44.5	2.7	2.2	1.7
P0A6I9	CoaE	Cytoplasm	Monomer	125.3	105.8	81.7	11.8	10.0	7.7
P75960	CobB	Cytoplasm	Monomer	117.4	99.2	76.6	10.2	8.6	6.6
P52086	CobC		Monomer	123.8	104.6	80.8	11.5	9.7	7.5
P36561	CobS	Cell inner membrane	Monomer	117.9	99.6	77.0	10.3	8.7	6.7
			Complex with SecB	71.2	58.2	46.5	3.0	2.4	1.9
			Complex with TF (Tig)	78.5	66.3	51.2	3.8	3.2	2.5
P36562	CobT		Monomer	103.3	87.3	67.4	7.6	6.4	4.9
			Homodimer	78.7	64.3	51.4	3.9	3.2	2.5
P0AE76	CobU		Monomer	131.1	110.7	85.5	13.0	11.0	8.5
			Homodimer	99.9	81.6	65.2	7.0	5.7	4.6
P25524	CodA		Monomer	93.6	79.1	61.1	6.0	5.0	3.9
			Homotetramer	54.4	44.4	35.5	1.4	1.2	0.9
P0AA82	CodB	Cell inner membrane	Monomer	96.8	81.8	63.2	6.5	5.5	4.2

UniProtKB A. N.	Protein name	Subcellular location	Subunit	D_0 $\left(\frac{\mu m^2}{s}\right)$			D_{cyto} $\left(\frac{\mu m^2}{s}\right)$		
				310 K	303 K	293 K	310 K	303 K	293 K
			Complex with SecB	66.7	54.5	43.6	2.5	2.1	1.6
			Complex with TF (Tig)	72.3	61.1	47.2	3.1	2.6	2.0
P46891	Cof		Monomer	111.6	94.3	72.8	9.1	7.7	5.9
P75974	CohE		Monomer	120.3	101.6	78.5	10.8	9.1	7.0
Q59385	CopA	Cell membrane	Monomer	73.6	62.2	48.0	3.3	2.8	2.1
			Complex with SecB	58.6	47.9	38.3	1.8	1.4	1.1
			Complex with TF (Tig)	62.0	52.4	40.5	2.1	1.7	1.3
P0ABI4	CorA	Cell inner membrane	Monomer	103.8	87.7	67.7	7.6	6.5	5.0
			Complex with SecB	68.5	55.9	44.7	2.7	2.2	1.8
			Complex with TF (Tig)	74.6	63.1	48.7	3.4	2.9	2.2
P0AE78	CorC		Monomer	107.7	91.0	70.3	8.3	7.0	5.4
P08331	CpdB	Periplasm	Monomer	80.1	67.7	52.3	4.1	3.4	2.6
			Complex with SecB	61.3	50.1	40.0	2.0	1.6	1.3
			Complex with TF (Tig)	65.3	55.2	42.6	2.4	2.0	1.5
P0CB39	CptA	Cell inner membrane	Monomer	82.0	69.3	53.5	4.3	3.6	2.8
			Complex with SecB	62.1	50.7	40.5	2.1	1.7	1.3
			Complex with TF (Tig)	66.3	56.0	43.2	2.5	2.1	1.6
P0AE82	CpxA	Cell inner membrane	Monomer	90.7	76.6	59.2	5.5	4.7	3.6
			Complex with SecB	65.0	53.1	42.4	2.3	1.9	1.5
			Complex with TF (Tig)	70.0	59.1	45.7	2.9	2.4	1.9
P0AE85	CpxP	Periplasm	Monomer	134.2	113.4	87.6	13.7	11.6	9.0
			Complex with SecB	73.5	60.1	48.0	3.2	2.7	2.1
			Complex with TF (Tig)	81.8	69.1	53.4	4.3	3.6	2.8
P0AE88	CpxR	Cytoplasm	Monomer	118.1	99.7	77.0	10.3	8.7	6.7
P37001	CrcA		Monomer	127.2	107.4	83.0	12.2	10.3	8.0
P37002	CrcB	Cell inner membrane	Monomer	152.2	128.5	99.3	18.0	15.2	11.7
			Complex with SecB	75.3	61.5	49.1	3.5	2.8	2.3
			Complex with TF (Tig)	84.4	71.3	55.1	4.6	3.9	3.0
P0AE91	CreA		Monomer	139.8	118.1	91.2	15.0	12.7	9.8
P08368	CreB	Cytoplasm	Monomer	118.4	100.0	77.3	10.4	8.8	6.8
P08401	CreC	Cell inner membrane	Monomer	90.3	76.3	58.9	5.5	4.6	3.6

UniProtKB A. N.	Protein name	Subcellular location	Subunit	D_0 $\left(\frac{\mu m^2}{s}\right)$			D_{cyto} $\left(\frac{\mu m^2}{s}\right)$		
				310 K	303 K	293 K	310 K	303 K	293 K
			Complex with SecB	64.9	53.0	42.3	2.3	1.9	1.5
			Complex with TF (Tig)	69.9	59.0	45.6	2.8	2.4	1.9
P08369	CreD	Cell inner membrane	Monomer	91.9	77.7	60.0	5.7	4.8	3.7
			Complex with SecB	65.4	53.4	42.7	2.4	1.9	1.5
			Complex with TF (Tig)	70.5	59.6	46.0	2.9	2.5	1.9
P24251	Crl	Cytoplasm	Monomer	144.7	122.3	94.4	16.2	13.7	10.6
P75975	CroE		Monomer	194.1	164.0	126.7	29.7	25.1	19.4
P0ACJ8	Crp		Monomer	123.1	104.0	80.3	11.3	9.6	7.4
P69783	Crr	Cytoplasm	Monomer	136.3	115.1	88.9	14.2	12.0	9.3
			Heterodimer (Crr−GlpK)	78.5	66.3	51.2	3.9	3.3	2.5
Q46925	CsdA		Monomer	97.2	82.1	63.4	6.5	5.5	4.3
			Homodimer	74.1	60.5	48.3	3.3	2.7	2.2
P28307	CsgA	Fimbrium	Monomer	147.0	124.2	95.9	16.7	14.1	10.9
			Complex with SecB	74.9	61.1	48.8	3.4	2.8	2.2
			Complex with TF (Tig)	83.7	70.7	54.6	4.5	3.8	3.0
P0ABK7	CsgB	Fimbrium	Monomer	143.9	121.6	93.9	16.0	13.5	10.4
			Complex with SecB	74.6	60.9	48.7	3.4	2.8	2.2
			Complex with TF (Tig)	83.3	70.4	54.4	4.5	3.8	2.9
P52107	CsgC	Periplasm	Monomer	160.8	135.8	104.9	20.2	17.1	13.2
			Complex with SecB	76.0	62.0	49.6	3.5	2.9	2.3
			Complex with TF (Tig)	85.4	72.1	55.7	4.8	4.0	3.1
P52106	CsgD	Cell inner membrane	Monomer	120.6	101.9	78.7	10.8	9.1	7.1
			Complex with SecB	71.7	58.5	46.8	3.0	2.5	2.0
			Complex with TF (Tig)	79.1	66.8	51.6	3.9	3.3	2.6
P0AE95	CsgE		Monomer	147.6	124.7	96.3	16.9	14.3	11.0
P0AE98	CsgF		Monomer	147.0	124.1	95.9	16.7	14.1	10.9
P0AEA2	CsgG	Cell membrane	Monomer	111.3	94.1	72.7	9.0	7.6	5.9
			Complex with SecB	70.0	57.2	45.7	2.9	2.3	1.9
			Complex with TF (Tig)	76.8	64.9	50.1	3.6	3.1	2.4
P76621	CsiD		Monomer	102.9	86.9	67.2	7.5	6.3	4.9
			Homotetramer	59.8	48.8	39.0	1.9	1.5	1.2

UniProtKB A. N.	Protein name	Subcellular location	Subunit	$D_0\left(\frac{\mu m^2}{s}\right)$			$D_{\text{cyto}}\left(\frac{\mu m^2}{s}\right)$		
				310 K	303 K	293 K	310 K	303 K	293 K
P54901	CsiE		Monomer	92.5	78.1	60.4	5.8	4.9	3.8
P37338	CsiR	Cytoplasm	Monomer	120.5	101.8	78.6	10.8	9.1	7.0
P0A9X9	CspA	Cytoplasm	Monomer	194.1	164.0	126.7	29.7	25.1	19.4
P36995	CspB	Cytoplasm	Monomer	191.0	161.3	124.6	28.7	24.3	18.7
P0A9Y6	CspC	Cytoplasm	Monomer	194.1	164.0	126.7	29.7	25.1	19.4
P0A968	CspD	Cytoplasm	Monomer	188.6	159.3	123.1	28.0	23.7	18.3
			Homodimer	143.7	117.4	93.8	15.9	13.0	10.4
P0A972	CspE	Cytoplasm	Monomer	193.5	163.5	126.3	29.5	24.9	19.2
P0A976	CspF	Cytoplasm	Monomer	192.5	162.6	125.6	29.2	24.6	19.0
P0A978	CspG	Cytoplasm	Monomer	190.3	160.8	124.2	28.5	24.1	18.6
P0A982	CspH	Cytoplasm	Monomer	190.9	161.3	124.6	28.7	24.2	18.7
P0A986	CspI	Cytoplasm	Monomer	191.3	161.6	124.8	28.8	24.3	18.8
P69913	CsrA		Monomer	200.0	169.0	130.5	31.5	26.6	20.5
			Homodimer	152.4	124.5	99.5	18.1	14.8	11.8
P13518	CsrD	Cell membrane	Monomer	79.0	66.7	51.6	3.9	3.3	2.6
			Complex with SecB	60.9	49.7	39.7	2.0	1.6	1.3
			Complex with TF (Tig)	64.8	54.8	42.3	2.3	2.0	1.5
P15078	CstA	Cell inner membrane	Monomer	78.3	66.1	51.1	3.8	3.2	2.5
			Complex with SecB	60.6	49.5	39.5	1.9	1.6	1.3
			Complex with TF (Tig)	64.4	54.4	42.1	2.3	1.9	1.5
P36649	CueO	Periplasm	Monomer	87.5	73.9	57.1	5.1	4.3	3.3
			Complex with SecB	64.0	52.3	41.7	2.2	1.8	1.5
			Complex with TF (Tig)	68.7	58.0	44.8	2.7	2.3	1.8
P0A9G4	CueR	Cytoplasm	Monomer	146.3	123.6	95.4	16.6	14.0	10.8
			Homodimer	111.5	91.1	72.7	9.0	7.4	5.9
P38054	CusA	Cell inner membrane	Monomer	66.3	56.0	43.3	2.5	2.1	1.6
			Complex with SecB	55.1	45.0	36.0	1.5	1.2	1.0
			Complex with TF (Tig)	57.8	48.8	37.7	1.7	1.4	1.1
P77239	CusB		Monomer	96.3	81.3	62.8	6.4	5.4	4.2
P77211	CusC	Cell outer membrane	Monomer	91.6	77.4	59.8	5.7	4.8	3.7
			Complex with SecB	65.3	53.3	42.6	2.4	1.9	1.5

UniProtKB A. N.	Protein name	Subcellular location	Subunit	D_0 $\left(\frac{\mu m^2}{s}\right)$			D_{cyto} $\left(\frac{\mu m^2}{s}\right)$		
				310 K	303 K	293 K	310 K	303 K	293 K
			Complex with TF (Tig)	70.4	59.5	45.9	2.9	2.4	1.9
P77214	CusF	Periplasm	Monomer	159.3	134.6	104.0	19.8	16.8	12.9
			Complex with SecB	75.8	62.0	49.5	3.5	2.9	2.3
			Complex with TF (Tig)	85.2	72.0	55.6	4.7	4.0	3.1
P0ACZ8	CusR	Cytoplasm	Monomer	119.7	101.1	78.1	10.7	9.0	7.0
P77485	CusS	Cell inner membrane	Monomer	89.2	75.4	58.2	5.3	4.5	3.5
			Complex with SecB	64.5	52.7	42.1	2.3	1.9	1.5
			Complex with TF (Tig)	69.4	58.7	45.3	2.8	2.4	1.8
P69488	CutA	Cytoplasm	Monomer	158.9	134.3	103.7	19.7	16.7	12.9
			Homotrimer	103.3	84.4	67.4	7.6	6.2	4.9
P67826	CutC	Cytoplasm	Monomer	117.3	99.1	76.5	10.2	8.6	6.6
			Homodimer	89.4	73.0	58.3	5.3	4.4	3.5
P08550	CvpA	Cell inner membrane	Monomer	137.3	116.0	89.6	14.4	12.2	9.4
			Complex with SecB	73.9	60.4	48.2	3.3	2.7	2.1
			Complex with TF (Tig)	82.3	69.5	53.7	4.3	3.7	2.8
P76007	CvrA	Cell inner membrane	Monomer	84.2	71.2	55.0	4.6	3.9	3.0
			Complex with SecB	62.9	51.3	41.0	2.1	1.7	1.4
			Complex with TF (Tig)	67.3	56.8	43.9	2.6	2.2	1.7
P00936	CyaA	Cytoplasm	Monomer	70.6	59.7	46.1	2.9	2.5	1.9
P27838	CyaY		Monomer	159.4	134.7	104.0	19.9	16.8	13.0
P0ABE5	CybB	Cell inner membrane	Monomer	130.7	110.5	85.3	13.0	11.0	8.5
			Complex with SecB	73.1	59.7	47.7	3.2	2.6	2.1
			Complex with TF (Tig)	81.2	68.6	53.0	4.2	3.5	2.7
P0AAE0	CycA	Cell inner membrane	Monomer	90.6	76.6	59.1	5.5	4.7	3.6
			Complex with SecB	65.0	53.1	42.4	2.3	1.9	1.5
			Complex with TF (Tig)	70.0	59.1	45.7	2.9	2.4	1.9
P0ABJ9	CydA	Cell inner membrane	Monomer	86.5	73.1	56.4	4.9	4.2	3.2
			Complex with SecB	63.6	52.0	41.5	2.2	1.8	1.4
			Complex with TF (Tig)	68.3	57.7	44.6	2.7	2.3	1.7
P0ABK2	CydB	Cell inner membrane	Monomer	97.9	82.7	63.9	6.7	5.6	4.3
			Complex with SecB	67.0	54.8	43.7	2.5	2.1	1.7

UniProtKB A. N.	Protein name	Subcellular location	Subunit	D_0 $\left(\frac{\mu m^2}{s}\right)$			D_{cyto} $\left(\frac{\mu m^2}{s}\right)$		
				310 K	303 K	293 K	310 K	303 K	293 K
			Complex with TF (Tig)	72.7	61.4	47.4	3.2	2.7	2.1
P23886	CydC	Cell inner membrane	Monomer	83.9	70.9	54.7	4.6	3.8	3.0
			Complex with SecB	62.7	51.3	40.9	2.1	1.7	1.4
			Complex with TF (Tig)	67.1	56.7	43.8	2.6	2.2	1.7
P29018	CydD	Cell inner membrane	Monomer	82.8	69.9	54.0	4.4	3.7	2.9
			Complex with SecB	62.3	50.9	40.7	2.1	1.7	1.4
			Complex with TF (Tig)	66.6	56.3	43.5	2.5	2.1	1.6
P27111	CynR	Cytoplasm	Monomer	108.1	91.3	70.5	8.4	7.1	5.5
P00816	CynS		Monomer	140.0	118.2	91.3	15.1	12.7	9.8
			Homodecamer	56.8	46.4	37.0	1.6	1.3	1.0
P0ABE9	CynT		Monomer	122.9	103.8	80.2	11.3	9.5	7.4
P17583	CynX	Cell inner membrane	Monomer	98.9	83.5	64.5	6.8	5.8	4.5
			Complex with SecB	67.3	55.0	43.9	2.6	2.1	1.7
			Complex with TF (Tig)	73.0	61.7	47.7	3.2	2.7	2.1
P0ABJ1	CyoA	Cell inner membrane	Monomer	105.7	89.3	69.0	8.0	6.7	5.2
			Complex with SecB	68.9	56.3	45.0	2.7	2.2	1.8
			Complex with TF (Tig)	75.2	63.5	49.1	3.4	2.9	2.2
P0ABI8	CyoB	Cell inner membrane	Monomer	78.6	66.4	51.3	3.9	3.3	2.5
			Complex with SecB	60.7	49.6	39.6	1.9	1.6	1.3
			Complex with TF (Tig)	64.6	54.6	42.2	2.3	1.9	1.5
P0ABJ3	CyoC	Cell inner membrane	Monomer	125.3	105.8	81.7	11.8	10.0	7.7
			Complex with SecB	72.4	59.1	47.2	3.1	2.5	2.0
			Complex with TF (Tig)	80.1	67.7	52.3	4.1	3.4	2.6
P0ABJ6	CyoD	Cell inner membrane	Monomer	160.5	135.6	104.7	20.1	17.0	13.1
			Complex with SecB	75.9	62.0	49.5	3.5	2.9	2.3
			Complex with TF (Tig)	85.3	72.1	55.7	4.8	4.0	3.1
P0AEA5	CyoE	Cell inner membrane	Monomer	109.0	92.1	71.1	8.6	7.3	5.6
			Complex with SecB	69.6	56.8	45.4	2.8	2.3	1.8
			Complex with TF (Tig)	76.2	64.4	49.7	3.6	3.0	2.3
P16676	CysA	Cell inner membrane	Monomer	99.2	83.8	64.7	6.9	5.8	4.5
			Complex with SecB	67.4	55.0	44.0	2.6	2.1	1.7

UniProtKB A. N.	Protein name	Subcellular location	Subunit	D_0 $\left(\frac{\mu m^2}{s}\right)$			D_{cyto} $\left(\frac{\mu m^2}{s}\right)$		
				310 K	303 K	293 K	310 K	303 K	293 K
			Complex with TF (Tig)	73.1	61.8	47.7	3.2	2.7	2.1
P0A9F3	CysB	Cytoplasm	Monomer	104.2	88.1	68.0	7.7	6.5	5.0
			Homotetramer	60.5	49.5	39.5	1.9	1.6	1.3
P0A6J1	CysC		Monomer	125.9	106.4	82.2	11.9	10.1	7.8
P21156	CysD		Monomer	105.4	89.0	68.7	7.9	6.7	5.2
			Heterodimer (CysD−CysN)	73.6	62.2	48.1	3.3	2.8	2.1
P0A9D4	CysE	Cytoplasm	Monomer	113.2	95.6	73.8	9.4	7.9	6.1
			Homohexamer	56.1	45.8	36.6	1.5	1.3	1.0
P0AEA8	CysG		Monomer	91.8	77.6	59.9	5.7	4.8	3.7
P17854	CysH	Cytoplasm	Monomer	115.3	97.4	75.2	9.8	8.3	6.4
			Homodimer	87.8	71.8	57.3	5.1	4.2	3.3
P17846	CysI		Monomer	83.3	70.4	54.4	4.5	3.8	2.9
P38038	CysJ		Monomer	82.2	69.4	53.6	4.3	3.7	2.8
P0ABK5	CysK		Monomer	106.2	89.7	69.3	8.1	6.8	5.3
			Homodimer	80.9	66.1	52.8	4.2	3.4	2.7
P16703	CysM		Monomer	108.5	91.6	70.8	8.5	7.2	5.5
			Homodimer	82.7	67.5	53.9	4.4	3.6	2.9
P23845	CysN		Monomer	90.0	76.1	58.7	5.4	4.6	3.5
			Heterodimer (CysD−CysN)	73.6	62.2	48.1	3.3	2.8	2.1
P16700	CysP	Periplasm	Monomer	102.6	86.7	67.0	7.5	6.3	4.9
			Complex with SecB	68.2	55.7	44.5	2.7	2.2	1.7
			Complex with TF (Tig)	74.3	62.8	48.5	3.3	2.8	2.2
P22255	CysQ	Cytoplasm. Cell inner membrane	Monomer	116.6	98.5	76.1	10.0	8.5	6.5
P21888	CysS	Cytoplasm	Monomer	90.3	76.3	58.9	5.5	4.6	3.6
P16701	CysU	Cell inner membrane	Monomer	111.7	94.4	72.9	9.1	7.7	5.9
			Complex with SecB	70.1	57.3	45.8	2.9	2.3	1.9
			Complex with TF (Tig)	76.9	65.0	50.2	3.7	3.1	2.4
P0AEB0	CysW	Cell inner membrane	Monomer	108.6	91.8	70.9	8.5	7.2	5.6
			Complex with SecB	69.5	56.8	45.4	2.8	2.3	1.8
			Complex with TF (Tig)	76.1	64.3	49.6	3.6	3.0	2.3

UniProtKB A. N.	Protein name	Subcellular location	Subunit	D_0 $\left(\frac{\mu m^2}{s}\right)$			D_{cyto} $\left(\frac{\mu m^2}{s}\right)$		
				310 K	303 K	293 K	310 K	303 K	293 K
P0A6J3	CysZ	Cell inner membrane	Monomer	113.2	95.6	73.9	9.4	7.9	6.1
			Complex with SecB	70.4	57.5	45.9	2.9	2.4	1.9
			Complex with TF (Tig)	77.3	65.3	50.4	3.7	3.1	2.4
P0ACN7	CytR		Monomer	102.4	86.5	66.8	7.4	6.3	4.8
P0AEB2	DacA	Cell inner membrane	Monomer	96.1	81.2	62.7	6.4	5.4	4.2
P24228	DacB	Periplasm	Monomer	90.5	76.5	59.1	5.5	4.7	3.6
			Complex with SecB	64.9	53.1	42.4	2.3	1.9	1.5
			Complex with TF (Tig)	70.0	59.1	45.7	2.8	2.4	1.9
P08506	DacC	Cell inner membrane	Monomer	96.9	81.8	63.2	6.5	5.5	4.2
			Complex with SecB	66.8	54.5	43.6	2.5	2.1	1.6
			Complex with TF (Tig)	72.3	61.1	47.2	3.1	2.6	2.0
P33013	DacD	Cell inner membrane	Monomer	97.1	82.0	63.4	6.5	5.5	4.3
			Complex with SecB	66.8	54.6	43.6	2.5	2.1	1.6
			Complex with TF (Tig)	72.4	61.2	47.3	3.1	2.6	2.0
P0A6J5	DadA	Cell inner membrane	Monomer	93.6	79.1	61.1	6.0	5.0	3.9
			Complex with SecB	65.8	53.8	43.0	2.4	2.0	1.6
			Complex with TF (Tig)	71.1	60.1	46.4	3.0	2.5	1.9
P29012	DadX		Monomer	101.3	85.6	66.1	7.2	6.1	4.7
P0AEE8	Dam		Monomer	109.2	92.3	71.3	8.6	7.3	5.6
P11557	DamX	Cell membrane	Monomer	94.7	80.0	61.8	6.2	5.2	4.0
			Complex with SecB	66.2	54.1	43.2	2.5	2.0	1.6
			Complex with TF (Tig)	71.6	60.5	46.7	3.0	2.6	2.0
P0A6L2	DapA	Cytoplasm	Monomer	110.3	93.2	72.0	8.8	7.5	5.8
			Homotetramer	64.1	52.4	41.8	2.2	1.8	1.5
P04036	DapB	Cytoplasm	Monomer	114.0	96.3	74.4	9.5	8.1	6.2
			Homotetramer	66.2	54.1	43.2	2.5	2.0	1.6
P0A9D8	DapD	Cytoplasm	Monomer	112.3	94.9	73.3	9.2	7.8	6.0
			Homotrimer	73.0	59.6	47.6	3.2	2.6	2.1
P0AED7	DapE		Monomer	99.0	83.6	64.6	6.8	5.8	4.5
			Homodimer	75.4	61.6	49.2	3.5	2.8	2.3
P0A6K1	DapF	Cytoplasm	Monomer	111.8	94.5	73.0	9.1	7.7	5.9

UniProtKB A. N.	Protein name	Subcellular location	Subunit	D_0 $\left(\frac{\mu m^2}{s}\right)$			D_{cyto} $\left(\frac{\mu m^2}{s}\right)$		
				310 K	303 K	293 K	310 K	303 K	293 K
P21693	DbpA	Cytoplasm	Monomer	92.4	78.1	60.3	5.8	4.9	3.8
P28248	Dcd		Monomer	128.4	108.5	83.8	12.5	10.5	8.1
			Homotetramer	74.6	60.9	48.7	3.4	2.8	2.2
P0AED9	Dcm		Monomer	89.4	75.5	58.4	5.3	4.5	3.5
P24171	Dcp	Cytoplasm	Monomer	77.3	65.3	50.4	3.7	3.1	2.4
P0AEE1	DcrB	Periplasm	Monomer	132.0	111.5	86.2	13.3	11.2	8.6
			Complex with SecB	73.3	59.8	47.8	3.2	2.6	2.1
			Complex with TF (Tig)	81.4	68.8	53.1	4.2	3.6	2.8
P0A830	DctA	Cell inner membrane	Monomer	95.3	80.5	62.2	6.2	5.3	4.1
			Complex with SecB	66.3	54.2	43.3	2.5	2.0	1.6
			Complex with TF (Tig)	71.8	60.6	46.8	3.0	2.6	2.0
P37195	DctR		Monomer	130.4	110.2	85.1	12.9	10.9	8.4
P0ABN5	DcuA	Cell inner membrane	Monomer	95.1	80.3	62.0	6.2	5.2	4.0
			Complex with SecB	66.3	54.1	43.2	2.5	2.0	1.6
			Complex with TF (Tig)	71.7	60.6	46.8	3.0	2.6	2.0
P0ABN9	DcuB	Cell inner membrane	Monomer	93.3	78.8	60.9	5.9	5.0	3.9
			Complex with SecB	65.8	53.7	42.9	2.4	2.0	1.6
			Complex with TF (Tig)	71.0	60.0	46.4	3.0	2.5	1.9
P0ABP3	DcuC	Cell inner membrane	Monomer	93.0	78.5	60.7	5.9	5.0	3.8
			Complex with SecB	65.7	53.6	42.9	2.4	2.0	1.6
			Complex with TF (Tig)	70.9	59.9	46.3	3.0	2.5	1.9
P45428	DcuD	Cell inner membrane	Monomer	92.7	78.3	60.5	5.8	4.9	3.8
			Complex with SecB	65.6	53.6	42.8	2.4	2.0	1.6
			Complex with TF (Tig)	70.8	59.8	46.2	2.9	2.5	1.9
P0AD01	DcuR	Cytoplasm	Monomer	116.1	98.1	75.7	9.9	8.4	6.5
P0AEC8	DcuS	Cell inner membrane	Monomer	85.2	71.9	55.6	4.7	4.0	3.1
			Complex with SecB	63.2	51.6	41.2	2.2	1.8	1.4
			Complex with TF (Tig)	67.7	57.2	44.2	2.6	2.2	1.7
P76316	DcyD		Monomer	105.4	89.0	68.8	7.9	6.7	5.2
			Homodimer	80.3	65.6	52.4	4.1	3.3	2.7
P0A6J8	DdlA	Cytoplasm	Monomer	100.9	85.2	65.8	7.2	6.0	4.7

UniProtKB A. N.	Protein name	Subcellular location	Subunit	$D_0 \left(\frac{\mu m^2}{s}\right)$			$D_{\text{cyto}} \left(\frac{\mu m^2}{s}\right)$		
				310 K	303 K	293 K	310 K	303 K	293 K
P07862	DdlB	Cytoplasm	Monomer	108.2	91.4	70.6	8.4	7.1	5.5
P76128	DdpA	Periplasm	Monomer	86.8	73.3	56.7	5.0	4.2	3.2
			Complex with SecB	63.7	52.1	41.6	2.2	1.8	1.4
			Complex with TF (Tig)	68.4	57.8	44.6	2.7	2.3	1.8
P77308	DdpB	Cell inner membrane	Monomer	102.9	87.0	67.2	7.5	6.3	4.9
			Complex with SecB	68.3	55.8	44.5	2.7	2.2	1.7
			Complex with TF (Tig)	74.4	62.8	48.5	3.3	2.8	2.2
P77463	DdpC	Cell inner membrane	Monomer	109.4	92.4	71.4	8.7	7.3	5.6
			Complex with SecB	69.7	56.9	45.5	2.8	2.3	1.8
			Complex with TF (Tig)	76.3	64.5	49.8	3.6	3.0	2.3
P77268	DdpD	Cell inner membrane	Monomer	104.3	88.1	68.1	7.7	6.5	5.1
			Complex with SecB	68.6	56.0	44.8	2.7	2.2	1.8
			Complex with TF (Tig)	74.8	63.2	48.8	3.4	2.9	2.2
P77622	DdpF	Cell inner membrane	Monomer	106.0	89.6	69.2	8.0	6.8	5.3
			Complex with SecB	69.0	56.3	45.0	2.7	2.2	1.8
			Complex with TF (Tig)	75.3	63.6	49.2	3.5	2.9	2.3
P77790	DdpX	Secreted	Monomer	128.5	108.5	83.8	12.5	10.5	8.1
			Complex with SecB	72.8	59.5	47.5	3.2	2.6	2.1
			Complex with TF (Tig)	80.7	68.2	52.7	4.1	3.5	2.7
P0A9P6	DeaD	Cytoplasm	Monomer	80.2	67.8	52.3	4.1	3.4	2.7
P0ABP6	DedA	Cell inner membrane	Monomer	121.4	102.6	79.2	11.0	9.3	7.2
			Complex with SecB	71.8	58.6	46.8	3.1	2.5	2.0
			Complex with TF (Tig)	79.3	67.0	51.7	3.9	3.3	2.6
P09549	DedD		Monomer	124.6	105.3	81.3	11.7	9.8	7.6
P0A6K3	Def		Monomer	133.2	112.6	86.9	13.5	11.4	8.8
P0C0V0	DegP	Periplasm	Monomer	92.3	77.9	60.2	5.8	4.9	3.8
			Complex with SecB	65.5	53.5	42.7	2.4	1.9	1.6
			Complex with TF (Tig)	70.6	59.7	46.1	2.9	2.5	1.9
P39099	DegQ	Periplasm	Monomer	93.9	79.3	61.3	6.0	5.1	3.9
			Complex with SecB	65.9	53.9	43.0	2.4	2.0	1.6
			Complex with TF (Tig)	71.3	60.2	46.5	3.0	2.5	2.0

UniProtKB A. N.	Protein name	Subcellular location	Subunit	D_0 $\left(\frac{\mu m^2}{s}\right)$			D_{cyto} $\left(\frac{\mu m^2}{s}\right)$		
				310 K	303 K	293 K	310 K	303 K	293 K
P0AEE3	DegS	Periplasm	Monomer	102.7	86.7	67.0	7.5	6.3	4.9
			Complex with SecB	68.2	55.7	44.5	2.7	2.2	1.7
			Complex with TF (Tig)	74.3	62.8	48.5	3.3	2.8	2.2
P07650	DeoA		Monomer	93.9	79.3	61.3	6.0	5.1	3.9
			Homodimer	71.6	58.5	46.7	3.0	2.5	2.0
P0A6K6	DeoB	Cytoplasm	Monomer	96.2	81.3	62.8	6.4	5.4	4.2
P0A6L0	DeoC	Cytoplasm	Monomer	115.7	97.7	75.5	9.8	8.3	6.4
			Homodimer	88.1	72.0	57.5	5.2	4.2	3.4
P0ABP8	DeoD		Monomer	118.7	100.3	77.5	10.4	8.8	6.8
			Homohexamer	58.8	48.0	38.4	1.8	1.4	1.2
P0ACK5	DeoR		Monomer	114.4	96.6	74.6	9.6	8.1	6.3
			Homooctamer	50.6	41.3	33.0	1.2	0.9	0.8
P0A6P5	Der		Monomer	88.4	74.7	57.7	5.2	4.4	3.4
P0ABN1	DgkA	Cell inner membrane	Monomer	154.5	130.5	100.8	18.6	15.7	12.1
			Complex with SecB	75.5	61.7	49.3	3.5	2.8	2.3
			Complex with TF (Tig)	84.7	71.5	55.3	4.7	3.9	3.0
Q6BF16	DgoA		Monomer	128.1	108.2	83.6	12.4	10.5	8.1
Q6BF17	DgoD		Monomer	97.8	82.6	63.8	6.6	5.6	4.3
P31459	DgoK		Monomer	110.2	93.1	71.9	8.8	7.4	5.7
P31460	DgoR		Monomer	118.5	100.1	77.3	10.4	8.8	6.8
P0AA76	DgoT	Cell inner membrane	Monomer	94.0	79.4	61.3	6.0	5.1	3.9
			Complex with SecB	66.0	53.9	43.0	2.4	2.0	1.6
			Complex with TF (Tig)	71.3	60.2	46.5	3.0	2.5	2.0
P15723	Dgt		Monomer	85.8	72.5	56.0	4.8	4.1	3.1
			Homotetramer	49.8	40.7	32.5	1.1	0.9	0.7
P76015	DhaK		Monomer	102.0	86.2	66.6	7.3	6.2	4.8
			Homodimer	77.7	63.5	50.7	3.8	3.1	2.4
P76014	DhaL		Monomer	125.3	105.8	81.7	11.8	10.0	7.7
			Homodimer	95.5	78.0	62.3	6.3	5.1	4.1
P37349	DhaM		Monomer	90.8	76.7	59.2	5.5	4.7	3.6
			Homodimer	69.2	56.5	45.1	2.8	2.3	1.8

UniProtKB A. N.	Protein name	Subcellular location	Subunit	D_0 $\left(\frac{\mu m^2}{s}\right)$			D_{cyto} $\left(\frac{\mu m^2}{s}\right)$		
				310 K	303 K	293 K	310 K	303 K	293 K
P76016	DhaR		Monomer	80.2	67.8	52.3	4.1	3.4	2.7
P66817	DiaA		Monomer	128.7	108.7	84.0	12.5	10.6	8.2
			Homodimer	98.1	80.1	64.0	6.7	5.5	4.4
P06966	DicA		Monomer	144.7	122.3	94.4	16.2	13.7	10.6
P09557	DicB		Monomer	198.8	168.0	129.7	31.1	26.3	20.3
P06965	DicC		Monomer	183.2	154.8	119.6	26.4	22.3	17.2
Q47155	DinB	Cytoplasm	Monomer	100.7	85.0	65.7	7.1	6.0	4.6
P23840	DinD		Monomer	110.6	93.4	72.2	8.9	7.5	5.8
P28303	DinF	Cell inner membrane	Monomer	92.1	77.8	60.1	5.7	4.8	3.7
			Complex with SecB	65.4	53.4	42.7	2.4	1.9	1.6
			Complex with TF (Tig)	70.6	59.6	46.0	2.9	2.5	1.9
P27296	DinG		Monomer	75.8	64.1	49.5	3.5	3.0	2.3
P0ABR1	DinI		Monomer	180.2	152.2	117.6	25.6	21.6	16.7
Q47150	DinJ		Monomer	176.7	149.3	115.3	24.6	20.7	16.0
A5A624	DinQ	Membrane	Monomer	228.5	193.0	149.1	40.7	34.4	26.6
			Complex with SecB	78.7	64.3	51.4	3.9	3.2	2.5
			Complex with TF (Tig)	89.7	75.8	58.5	5.4	4.5	3.5
P31680	DjlA	Cell inner membrane	Monomer	111.3	94.0	72.6	9.0	7.6	5.9
			Complex with SecB	70.0	57.2	45.7	2.9	2.3	1.9
			Complex with TF (Tig)	76.8	64.9	50.1	3.6	3.1	2.4
P77381	DjlB		Monomer	88.3	74.6	57.6	5.2	4.4	3.4
P77359	DjlC		Monomer	88.0	74.3	57.4	5.1	4.3	3.4
Q46857	DkgA	Cytoplasm	Monomer	110.6	93.4	72.1	8.9	7.5	5.8
P30863	DkgB	Cytoplasm	Monomer	113.0	95.5	73.7	9.3	7.9	6.1
P0ABS1	DksA		Monomer	138.4	117.0	90.3	14.7	12.4	9.6
P06149	Dld	Cell membrane	Monomer	83.0	70.1	54.2	4.4	3.7	2.9
P37672	DlgD	Cytoplasm	Monomer	103.8	87.7	67.7	7.7	6.5	5.0
			Homodimer	79.1	64.6	51.6	3.9	3.2	2.6
P76251	DmlA	Cytoplasm	Monomer	99.9	84.4	65.2	7.0	5.9	4.6
P76250	DmlR		Monomer	106.0	89.6	69.2	8.0	6.8	5.3
P18775	DmsA	Cell membrane	Monomer	72.8	61.5	47.5	3.2	2.7	2.1

UniProtKB A. N.	Protein name	Subcellular location	Subunit	D_0 $\left(\frac{\mu m^2}{s}\right)$			D_{cyto} $\left(\frac{\mu m^2}{s}\right)$		
				310 K	303 K	293 K	310 K	303 K	293 K
P18776	DmsB		Monomer	124.7	105.4	81.4	11.7	9.9	7.6
P18777	DmsC	Cell inner membrane	Monomer	111.0	93.7	72.4	9.0	7.6	5.8
			Complex with SecB	70.0	57.2	45.7	2.9	2.3	1.9
			Complex with TF (Tig)	76.7	64.8	50.1	3.6	3.1	2.4
P69853	DmsD	Cell inner membrane	Monomer	123.7	104.5	80.7	11.5	9.7	7.5
P03004	DnaA	Cytoplasm	Monomer	90.0	76.1	58.7	5.4	4.6	3.5
P0ACB0	DnaB		Monomer	90.1	76.1	58.8	5.4	4.6	3.6
			Homohexamer	44.7	36.5	29.1	0.8	0.6	0.5
P0AEF0	DnaC		Monomer	115.3	97.4	75.3	9.8	8.3	6.4
P10443	DnaE	Cytoplasm	Monomer	63.1	53.3	41.2	2.2	1.8	1.4
P0ABS5	DnaG		Monomer	82.5	69.7	53.9	4.4	3.7	2.9
P08622	DnaJ	Cytoplasm	Monomer	99.1	83.7	64.7	6.9	5.8	4.5
			Homodimer	75.5	61.7	49.3	3.5	2.8	2.3
P0A6Y8	DnaK	Cytoplasm. Cell inner membrane	Monomer	80.9	68.3	52.8	4.2	3.5	2.7
P0A988	DnaN	Cytoplasm	Monomer	99.6	84.2	65.0	6.9	5.9	4.5
			Homodimer	75.9	62.0	49.5	3.5	2.9	2.3
P03007	DnaQ		Monomer	116.7	98.6	76.2	10.1	8.5	6.6
P0A8J2	DnaT		Monomer	132.9	112.3	86.7	13.4	11.4	8.8
P06710	DnaX		Monomer	79.9	67.5	52.2	4.0	3.4	2.6
P0AA89	DosC		Monomer	89.6	75.7	58.5	5.4	4.5	3.5
P76129	DosP		Monomer	72.8	61.5	47.5	3.2	2.7	2.1
			Homodimer	55.5	45.3	36.2	1.5	1.2	1.0
P0AEF4	DpiA	Cytoplasm	Monomer	119.6	101.1	78.1	10.6	9.0	6.9
P77510	DpiB	Cell inner membrane	Monomer	84.5	71.4	55.2	4.6	3.9	3.0
			Complex with SecB	63.0	51.4	41.1	2.1	1.8	1.4
			Complex with TF (Tig)	67.4	57.0	44.0	2.6	2.2	1.7
P23847	DppA	Periplasm	Monomer	85.3	72.1	55.7	4.8	4.0	3.1
			Complex with SecB	63.2	51.7	41.3	2.2	1.8	1.4
			Complex with TF (Tig)	67.8	57.2	44.2	2.6	2.2	1.7
P0AEF8	DppB	Cell inner membrane	Monomer	102.8	86.8	67.1	7.5	6.3	4.9

UniProtKB A. N.	Protein name	Subcellular location	Subunit	D_0 $\left(\frac{\mu m^2}{s}\right)$			D_{cyto} $\left(\frac{\mu m^2}{s}\right)$		
				310 K	303 K	293 K	310 K	303 K	293 K
			Complex with SecB	68.2	55.7	44.5	2.7	2.2	1.7
			Complex with TF (Tig)	74.3	62.8	48.5	3.3	2.8	2.2
P0AEG1	DppC	Cell inner membrane	Monomer	108.9	92.0	71.1	8.6	7.2	5.6
			Complex with SecB	69.6	56.8	45.4	2.8	2.3	1.8
			Complex with TF (Tig)	76.2	64.3	49.7	3.6	3.0	2.3
P0AAG0	DppD	Cell inner membrane	Monomer	104.6	88.4	68.3	7.8	6.6	5.1
			Complex with SecB	68.6	56.1	44.8	2.7	2.2	1.8
			Complex with TF (Tig)	74.9	63.3	48.9	3.4	2.9	2.2
P37313	DppF	Cell inner membrane	Monomer	102.7	86.8	67.0	7.5	6.3	4.9
			Complex with SecB	68.2	55.7	44.5	2.7	2.2	1.7
			Complex with TF (Tig)	74.3	62.8	48.5	3.3	2.8	2.2
P0ABT2	Dps	Cytoplasm > nucleoid	Monomer	135.0	114.0	88.1	13.9	11.8	9.1
			Homododecamer	51.0	41.6	33.3	1.2	1.0	0.8
P0AEG4	DsbA	Periplasm	Monomer	124.2	105.0	81.1	11.6	9.8	7.6
			Complex with SecB	72.2	59.0	47.1	3.1	2.5	2.0
			Complex with TF (Tig)	79.9	67.5	52.1	4.0	3.4	2.6
P0A6M2	DsbB	Cell inner membrane	Monomer	131.1	110.8	85.6	13.0	11.0	8.5
			Complex with SecB	73.1	59.8	47.7	3.2	2.6	2.1
			Complex with TF (Tig)	81.2	68.6	53.0	4.2	3.5	2.7
P0AEG6	DsbC	Periplasm	Monomer	119.3	100.8	77.9	10.6	8.9	6.9
			Complex with SecB	71.5	58.4	46.6	3.0	2.5	2.0
			Complex with TF (Tig)	78.8	66.6	51.4	3.9	3.3	2.5
P36655	DsbD	Cell inner membrane	Monomer	84.5	71.4	55.1	4.6	3.9	3.0
			Complex with SecB	62.9	51.4	41.1	2.1	1.7	1.4
			Complex with TF (Tig)	67.4	56.9	44.0	2.6	2.2	1.7
P0AA86	DsbE	Cell inner membrane	Monomer	129.4	109.4	84.5	12.7	10.7	8.3
			Complex with SecB	72.9	59.6	47.6	3.2	2.6	2.1
			Complex with TF (Tig)	80.9	68.4	52.8	4.2	3.5	2.7
P77202	DsbG	Periplasm	Monomer	116.1	98.0	75.7	9.9	8.4	6.5
			Complex with SecB	70.9	57.9	46.3	3.0	2.4	1.9
			Complex with TF (Tig)	78.0	65.9	50.9	3.8	3.2	2.5

UniProtKB A. N.	Protein name	Subcellular location	Subunit	D_0 $\left(\frac{\mu m^2}{s}\right)$ 310 K	303 K	293 K	D_{cyto} $\left(\frac{\mu m^2}{s}\right)$ 310 K	303 K	293 K
P00926	DsdA		Monomer	93.4	78.9	60.9	5.9	5.0	3.9
P46068	DsdC		Monomer	105.2	88.9	68.6	7.9	6.7	5.2
P08555	DsdX	Cell inner membrane	Monomer	93.9	79.3	61.3	6.0	5.1	3.9
			Complex with SecB	65.9	53.9	43.0	2.4	2.0	1.6
			Complex with TF (Tig)	71.3	60.2	46.5	3.0	2.5	2.0
P0AEG8	DsrB		Monomer	199.0	168.1	129.9	31.2	26.3	20.3
P0A6M4	Dtd	Cytoplasm	Monomer	143.7	121.4	93.7	15.9	13.5	10.4
			Homodimer	109.5	89.4	71.4	8.7	7.1	5.7
P77304	DtpA	Cell inner membrane	Monomer	89.1	75.3	58.1	5.3	4.5	3.5
			Complex with SecB	64.5	52.7	42.1	2.3	1.9	1.5
			Complex with TF (Tig)	69.4	58.6	45.3	2.8	2.4	1.8
P36837	DtpB	Cell inner membrane	Monomer	89.3	75.5	58.3	5.3	4.5	3.5
			Complex with SecB	64.6	52.7	42.1	2.3	1.9	1.5
			Complex with TF (Tig)	69.5	58.7	45.3	2.8	2.4	1.8
P75742	DtpD	Cell inner membrane	Monomer	89.0	75.2	58.1	5.3	4.5	3.4
			Complex with SecB	64.4	52.7	42.1	2.3	1.9	1.5
			Complex with TF (Tig)	69.3	58.6	45.2	2.8	2.3	1.8
P32695	DusA		Monomer	103.5	87.4	67.5	7.6	6.4	5.0
P0ABT5	DusB		Monomer	104.6	88.3	68.2	7.8	6.6	5.1
P33371	DusC		Monomer	105.3	89.0	68.7	7.9	6.7	5.2
P06968	Dut		Monomer	142.9	120.8	93.3	15.8	13.3	10.3
			Homotrimer	92.9	75.9	60.6	5.9	4.8	3.8
P45568	Dxr		Monomer	97.0	82.0	63.3	6.5	5.5	4.3
			Homodimer	74.0	60.4	48.3	3.3	2.7	2.2
P77488	Dxs		Monomer	81.6	68.9	53.2	4.2	3.6	2.8
			Homodimer	62.2	50.8	40.6	2.1	1.7	1.3
P36943	EaeH		Monomer	107.8	91.1	70.4	8.4	7.1	5.5
P31125	EamA	Cell inner membrane	Monomer	108.9	92.0	71.1	8.6	7.2	5.6
			Complex with SecB	69.6	56.8	45.4	2.8	2.3	1.8
			Complex with TF (Tig)	76.2	64.3	49.7	3.6	3.0	2.3
P38101	EamB	Cell inner membrane	Monomer	128.4	108.5	83.8	12.5	10.5	8.1

UniProtKB A. N.	Protein name	Subcellular location	Subunit	D_0 $\left(\frac{\mu m^2}{s}\right)$			D_{cyto} $\left(\frac{\mu m^2}{s}\right)$		
				310 K	303 K	293 K	310 K	303 K	293 K
			Complex with SecB	72.8	59.5	47.5	3.2	2.6	2.1
			Complex with TF (Tig)	80.7	68.2	52.7	4.1	3.5	2.7
P06864	EbgA		Monomer	65.6	55.4	42.8	2.4	2.0	1.6
			Heterooctamer ($EbgA_4-EbgC_4$)	36.1	30.5	23.5	0.4	0.3	0.3
P0AC73	EbgC		Monomer	138.7	117.2	90.5	14.8	12.5	9.6
			Heterooctamer ($EbgA_4-EbgC_4$)	36.1	30.5	23.5	0.4	0.3	0.3
P06846	EbgR		Monomer	104.2	88.0	68.0	7.7	6.5	5.0
P0ADB4	EcnA	Cell membrane	Monomer	238.9	201.8	155.9	44.3	37.4	28.9
			Complex with SecB	78.9	64.5	51.5	3.9	3.2	2.5
			Complex with TF (Tig)	90.0	76.1	58.7	5.4	4.6	3.5
P0ADB7	EcnB	Cell membrane	Monomer	229.8	194.2	150.0	41.2	34.8	26.9
			Complex with SecB	78.8	64.3	51.4	3.9	3.2	2.5
			Complex with TF (Tig)	89.7	75.8	58.5	5.4	4.6	3.5
P23827	Eco	Periplasm	Monomer	136.4	115.3	89.0	14.2	12.0	9.3
			Complex with SecB	73.8	60.3	48.1	3.3	2.7	2.1
			Complex with TF (Tig)	82.1	69.4	53.6	4.3	3.7	2.8
P33128	EcpD	Periplasm	Monomer	116.8	98.7	76.2	10.1	8.5	6.6
			Complex with SecB	71.0	58.0	46.4	3.0	2.4	1.9
			Complex with TF (Tig)	78.2	66.1	51.0	3.8	3.2	2.5
P0A955	Eda	Cytoplasm	Monomer	126.0	106.5	82.2	12.0	10.1	7.8
			Homotrimer	81.9	66.9	53.5	4.3	3.5	2.8
P0ADF6	Edd		Monomer	83.0	70.1	54.2	4.4	3.7	2.9
P75901	EfeU	Cell inner membrane	Monomer	111.7	94.4	72.9	9.1	7.7	5.9
			Complex with SecB	70.1	57.3	45.8	2.9	2.3	1.9
			Complex with TF (Tig)	76.9	65.0	50.2	3.7	3.1	2.4
P0A6N4	Efp	Cytoplasm	Monomer	130.0	109.8	84.8	12.8	10.8	8.4
P0AEH3	ElaA		Monomer	138.8	117.2	90.6	14.8	12.5	9.6
P0AEH5	ElaB		Monomer	164.4	138.9	107.3	21.2	17.9	13.8
Q47013	ElaD		Monomer	94.8	80.1	61.9	6.2	5.2	4.0
P0ABU5	ElbB		Monomer	124.5	105.2	81.2	11.6	9.8	7.6
P27303	EmrA	Cell inner membrane	Monomer	97.6	82.5	63.7	6.6	5.6	4.3

UniProtKB A. N.	Protein name	Subcellular location	Subunit	D_0 $\left(\frac{\mu m^2}{s}\right)$			D_{cyto} $\left(\frac{\mu m^2}{s}\right)$		
				310 K	303 K	293 K	310 K	303 K	293 K
			Complex with SecB	67.0	54.7	43.7	2.5	2.1	1.7
			Complex with TF (Tig)	72.6	61.3	47.4	3.1	2.7	2.1
P0AEJ0	EmrB	Cell inner membrane	Monomer	88.0	74.4	57.5	5.1	4.3	3.4
			Complex with SecB	64.2	52.4	41.9	2.3	1.8	1.5
			Complex with TF (Tig)	68.9	58.2	45.0	2.7	2.3	1.8
P31442	EmrD	Cell inner membrane	Monomer	98.1	82.9	64.0	6.7	5.7	4.4
			Complex with SecB	67.1	54.8	43.8	2.5	2.1	1.7
			Complex with TF (Tig)	72.8	61.5	47.5	3.2	2.7	2.1
P23895	EmrE	Cell inner membrane	Monomer	160.8	135.9	105.0	20.2	17.1	13.2
			Complex with SecB	76.0	62.1	49.6	3.5	2.9	2.3
			Complex with TF (Tig)	85.4	72.1	55.7	4.8	4.0	3.1
P52599	EmrK	Cell inner membrane	Monomer	97.8	82.6	63.8	6.6	5.6	4.3
			Complex with SecB	67.0	54.7	43.7	2.5	2.1	1.7
			Complex with TF (Tig)	72.7	61.4	47.4	3.1	2.7	2.1
P52600	EmrY	Cell inner membrane	Monomer	87.8	74.2	57.3	5.1	4.3	3.3
			Complex with SecB	64.1	52.3	41.8	2.2	1.8	1.5
			Complex with TF (Tig)	68.8	58.2	44.9	2.7	2.3	1.8
P0C960	EmtA	Cell outer membrane	Monomer	126.1	106.6	82.3	12.0	10.1	7.8
			Complex with SecB	72.5	59.2	47.3	3.1	2.6	2.0
			Complex with TF (Tig)	80.3	67.8	52.4	4.1	3.4	2.7
P25736	EndA	Periplasm	Monomer	117.4	99.2	76.6	10.2	8.6	6.6
			Complex with SecB	71.1	58.1	46.4	3.0	2.4	1.9
			Complex with TF (Tig)	78.3	66.2	51.1	3.8	3.2	2.5
P0A6P7	EngB		Monomer	123.3	104.2	80.5	11.4	9.6	7.4
P0ABU2	EngD		Monomer	100.5	84.9	65.6	7.1	6.0	4.6
P0A6P9	Eno	Cytoplasm > cytoskeleton Secreted Cell surface	Monomer	95.1	80.4	62.1	6.2	5.3	4.1
			Homodimer	72.5	59.2	47.3	3.1	2.6	2.0
P15047	EntA		Monomer	118.2	99.8	77.1	10.3	8.7	6.7
			Homooctamer	52.3	42.7	34.1	1.3	1.0	0.8
P0ADI4	EntB		Monomer	108.6	91.8	70.9	8.5	7.2	5.6
P0AEJ2	EntC		Monomer	97.5	82.3	63.6	6.6	5.6	4.3

UniProtKB A. N.	Protein name	Subcellular location	Subunit	D_0 $\left(\frac{\mu m^2}{s}\right)$			D_{cyto} $\left(\frac{\mu m^2}{s}\right)$		
				310 K	303 K	293 K	310 K	303 K	293 K
P19925	EntD		Monomer	123.2	104.1	80.4	11.4	9.6	7.4
P10378	EntE		Monomer	86.0	72.6	56.1	4.8	4.1	3.2
P11454	EntF		Monomer	61.0	51.5	39.8	2.0	1.7	1.3
P24077	EntS	Cell inner membrane	Monomer	97.1	82.1	63.4	6.5	5.5	4.3
			Complex with SecB	66.8	54.6	43.6	2.5	2.1	1.6
			Complex with TF (Tig)	72.4	61.2	47.3	3.1	2.6	2.0
P37690	EnvC	Periplasm	Monomer	94.4	79.7	61.6	6.1	5.2	4.0
			Complex with SecB	66.1	54.0	43.1	2.4	2.0	1.6
			Complex with TF (Tig)	71.4	60.4	46.6	3.0	2.5	2.0
P0ACT2	EnvR		Monomer	120.1	101.5	78.4	10.7	9.1	7.0
P10805	EnvY		Monomer	113.6	96.0	74.1	9.5	8.0	6.2
P0AEJ4	EnvZ	Cell inner membrane	Monomer	91.6	77.4	59.7	5.7	4.8	3.7
			Complex with SecB	65.3	53.3	42.6	2.4	1.9	1.5
			Complex with TF (Tig)	70.4	59.4	45.9	2.9	2.4	1.9
P0A9B6	Epd	Cytoplasm	Monomer	103.0	87.0	67.2	7.5	6.3	4.9
			Homotetramer	59.8	48.9	39.0	1.9	1.5	1.2
P30845	EptA	Cell inner membrane	Monomer	84.6	71.4	55.2	4.6	3.9	3.0
			Complex with SecB	63.0	51.4	41.1	2.1	1.8	1.4
			Complex with TF (Tig)	67.4	57.0	44.0	2.6	2.2	1.7
P37661	EptB	Cell inner membrane	Monomer	83.4	70.5	54.4	4.5	3.8	2.9
			Complex with SecB	62.6	51.1	40.8	2.1	1.7	1.4
			Complex with TF (Tig)	66.9	56.5	43.7	2.5	2.1	1.7
P06616	Era	Cytoplasm. Cell inner membrane	Monomer	107.0	90.4	69.8	8.2	6.9	5.4
P39176	ErfK	Periplasm	Monomer	106.3	89.8	69.4	8.1	6.8	5.3
			Complex with SecB	69.0	56.4	45.0	2.7	2.2	1.8
			Complex with TF (Tig)	75.4	63.7	49.2	3.5	2.9	2.3
P0ACC3	ErpA		Monomer	160.1	135.3	104.5	20.0	16.9	13.1
			Homodimer	122.0	99.7	79.6	11.1	9.1	7.3
P0A9R2	EssD		Monomer	190.4	160.8	124.2	28.5	24.1	18.6
P77237	EssQ		Monomer	190.7	161.1	124.5	28.6	24.2	18.7

UniProtKB A. N.	Protein name	Subcellular location	Subunit	D_0 $\left(\frac{\mu m^2}{s}\right)$			D_{cyto} $\left(\frac{\mu m^2}{s}\right)$		
				310 K	303 K	293 K	310 K	303 K	293 K
P38134	Etk	Cell inner membrane	Monomer	75.9	64.1	49.5	3.5	3.0	2.3
			Complex with SecB	59.6	48.7	38.9	1.8	1.5	1.2
			Complex with TF (Tig)	63.2	53.4	41.3	2.2	1.8	1.4
P0ACZ2	Etp		Monomer	142.2	120.1	92.8	15.6	13.2	10.2
P76551	EutA		Monomer	92.1	77.8	60.1	5.7	4.9	3.7
P0AEJ6	EutB		Monomer	92.2	77.9	60.2	5.8	4.9	3.8
			Heterodimer (EutB−EutC)	75.9	64.1	49.5	3.5	3.0	2.3
P19636	EutC		Monomer	109.6	92.6	71.5	8.7	7.4	5.7
			Heterodimer (EutB−EutC)	75.9	64.1	49.5	3.5	3.0	2.3
P77218	EutD		Monomer	104.3	88.1	68.1	7.8	6.5	5.1
P77445	EutE		Monomer	92.5	78.2	60.4	5.8	4.9	3.8
P76553	EutG		Monomer	99.2	83.8	64.7	6.9	5.8	4.5
P76552	EutH	Cell inner membrane	Monomer	97.6	82.4	63.7	6.6	5.6	4.3
			Complex with SecB	66.9	54.7	43.7	2.5	2.1	1.7
			Complex with TF (Tig)	72.6	61.3	47.4	3.1	2.7	2.0
P77277	EutJ		Monomer	112.0	94.6	73.1	9.2	7.7	6.0
P76540	EutK		Monomer	137.3	116.0	89.6	14.4	12.2	9.4
P76541	EutL		Monomer	124.9	105.5	81.5	11.7	9.9	7.6
P0ABF4	EutM		Monomer	173.4	146.5	113.2	23.6	20.0	15.4
P0AEJ8	EutN		Monomer	172.8	146.0	112.8	23.5	19.8	15.3
P76556	EutP		Monomer	138.0	116.6	90.1	14.6	12.3	9.5
P76555	EutQ		Monomer	119.7	101.1	78.1	10.6	9.0	6.9
P36547	EutR		Monomer	100.0	84.5	65.3	7.0	5.9	4.6
P63746	EutS		Monomer	162.5	137.3	106.0	20.7	17.5	13.5
P65643	EutT		Monomer	111.9	94.5	73.0	9.1	7.7	6.0
P0ACZ4	EvgA	Cytoplasm	Monomer	125.1	105.7	81.7	11.8	9.9	7.7
			Homodimer	95.4	77.9	62.2	6.3	5.1	4.1
P30855	EvgS	Cell inner membrane	Monomer	62.2	52.6	40.6	2.1	1.8	1.4
			Complex with SecB	52.9	43.2	34.5	1.3	1.1	0.9
			Complex with TF (Tig)	55.2	46.6	36.0	1.5	1.3	1.0
P0ABU7	ExbB	Cell inner membrane	Monomer	118.1	99.8	77.1	10.3	8.7	6.7

UniProtKB A. N.	Protein name	Subcellular location	Subunit	D_0 $\left(\frac{\mu m^2}{s}\right)$			D_{cyto} $\left(\frac{\mu m^2}{s}\right)$		
				310 K	303 K	293 K	310 K	303 K	293 K
			Complex with SecB	71.3	58.2	46.5	3.0	2.4	2.0
			Complex with TF (Tig)	78.5	66.3	51.2	3.9	3.3	2.5
P0ABV2	ExbD	Cell inner membrane	Monomer	145.2	122.7	94.7	16.3	13.8	10.6
			Complex with SecB	74.7	61.0	48.7	3.4	2.8	2.2
			Complex with TF (Tig)	83.5	70.5	54.5	4.5	3.8	2.9
P0AEK0	ExoX		Monomer	120.2	101.6	78.4	10.7	9.1	7.0
P0ACL2	ExuR		Monomer	112.4	95.0	73.3	9.2	7.8	6.0
P0AA78	ExuT	Cell inner membrane	Monomer	90.7	76.6	59.2	5.5	4.7	3.6
			Complex with SecB	65.0	53.1	42.4	2.3	1.9	1.5
			Complex with TF (Tig)	70.0	59.2	45.7	2.9	2.4	1.9
P0A6Q3	FabA	Cytoplasm	Monomer	134.2	113.4	87.6	13.7	11.6	9.0
			Homodimer	102.3	83.6	66.7	7.4	6.0	4.8
P0A953	FabB	Cytoplasm	Monomer	97.7	82.6	63.8	6.6	5.6	4.3
			Homodimer	74.5	60.8	48.6	3.4	2.7	2.2
P0AAI9	FabD		Monomer	108.8	91.9	71.0	8.5	7.2	5.6
P0AAI5	FabF		Monomer	97.3	82.2	63.5	6.6	5.6	4.3
			Homodimer	74.2	60.6	48.4	3.3	2.7	2.2
P0AEK2	FabG		Monomer	119.4	100.9	77.9	10.6	8.9	6.9
			Homotetramer	69.4	56.7	45.3	2.8	2.3	1.8
P0A6R0	FabH	Cytoplasm	Monomer	107.4	90.7	70.1	8.3	7.0	5.4
			Homodimer	81.8	66.9	53.4	4.3	3.5	2.8
P0AEK4	FabI		Monomer	115.4	97.5	75.3	9.8	8.3	6.4
			Homotetramer	67.0	54.8	43.8	2.5	2.1	1.7
P0ACU5	FabR	Cytoplasm	Monomer	121.6	102.7	79.4	11.0	9.3	7.2
			Homodimer	92.7	75.7	60.5	5.8	4.8	3.8
P0A6Q6	FabZ	Cytoplasm	Monomer	140.0	118.3	91.4	15.1	12.7	9.8
P21151	FadA	Cytoplasm	Monomer	99.3	83.9	64.8	6.9	5.8	4.5
			Heterotetramer ($FadA_2-FadB_2$)	49.6	41.9	32.3	1.1	0.9	0.7
P21177	FadB		Monomer	76.5	64.6	49.9	3.6	3.0	2.4
			Heterotetramer ($FadA_2-FadB_2$)	49.6	41.9	32.3	1.1	0.9	0.7
P69451	FadD	Membrane	Monomer	84.2	71.1	54.9	4.6	3.9	3.0

UniProtKB A. N.	Protein name	Subcellular location	Subunit	D_0 $\left(\frac{\mu m^2}{s}\right)$			D_{cyto} $\left(\frac{\mu m^2}{s}\right)$		
				310 K	303 K	293 K	310 K	303 K	293 K
			Complex with SecB	62.8	51.3	41.0	2.1	1.7	1.4
			Complex with TF (Tig)	67.3	56.8	43.9	2.6	2.2	1.7
Q47146	FadE		Monomer	73.2	61.8	47.7	3.2	2.7	2.1
P42593	FadH		Monomer	79.3	67.0	51.7	3.9	3.3	2.6
P76503	FadI	Cytoplasm	Monomer	94.4	79.8	61.6	6.1	5.2	4.0
			Heterotetramer ($FadI_2-FadJ_2$)	59.0	49.9	38.5	1.8	1.5	1.2
P77399	FadJ	Cytoplasm	Monomer	77.5	65.5	50.6	3.7	3.1	2.4
			Heterotetramer ($FadI_2-FadJ_2$)	59.0	49.9	38.5	1.8	1.5	1.2
P38135	FadK	Membrane	Monomer	85.0	71.8	55.5	4.7	4.0	3.1
			Complex with SecB	63.1	51.6	41.2	2.2	1.8	1.4
			Complex with TF (Tig)	67.6	57.1	44.1	2.6	2.2	1.7
P10384	FadL	Cell outer membrane	Monomer	92.9	78.5	60.6	5.9	5.0	3.8
			Complex with SecB	65.6	53.6	42.8	2.4	2.0	1.6
			Complex with TF (Tig)	70.9	59.9	46.2	2.9	2.5	1.9
P0A8V6	FadR	Cytoplasm	Monomer	116.9	98.8	76.3	10.1	8.5	6.6
			Homodimer	89.1	72.8	58.1	5.3	4.3	3.5
P0AB71	FbaA		Monomer	101.0	85.4	65.9	7.2	6.1	4.7
			Homodimer	77.0	62.9	50.2	3.7	3.0	2.4
P0A991	FbaB	Cytoplasm	Monomer	102.1	86.3	66.6	7.4	6.2	4.8
			Homooctamer	45.2	36.9	29.5	0.8	0.7	0.5
			Homodecamer	41.4	35.0	27.0	0.6	0.5	0.4
P0A993	Fbp	Cytoplasm	Monomer	103.5	87.4	67.5	7.6	6.4	5.0
			Homotetramer	60.1	49.1	39.2	1.9	1.5	1.2
P75681	FbpB	Cell inner membrane	Monomer	157.9	133.4	103.0	19.5	16.4	12.7
			Complex with SecB	75.7	61.9	49.4	3.5	2.9	2.3
			Complex with TF (Tig)	85.1	71.9	55.5	4.7	4.0	3.1
P37009	FbpC	Cell inner membrane	Monomer	101.1	85.4	66.0	7.2	6.1	4.7
			Complex with SecB	67.8	55.4	44.3	2.6	2.1	1.7
			Complex with TF (Tig)	73.8	62.3	48.2	3.3	2.8	2.1
P32055	Fcl	Cytoplasm	Monomer	104.3	88.1	68.0	7.7	6.5	5.0
			Homodimer	79.4	64.9	51.8	4.0	3.2	2.6

UniProtKB A. N.	Protein name	Subcellular location	Subunit	D_0 $\left(\frac{\mu m^2}{s}\right)$			D_{cyto} $\left(\frac{\mu m^2}{s}\right)$		
				310 K	303 K	293 K	310 K	303 K	293 K
P32177	FdhD	Cytoplasm	Monomer	111.3	94.1	72.7	9.0	7.6	5.9
P13024	FdhE	Cytoplasm	Monomer	105.9	89.4	69.1	8.0	6.8	5.2
P07658	FdhF		Monomer	76.6	64.7	50.0	3.6	3.1	2.4
P24183	FdnG	Periplasm	Monomer	66.7	56.3	43.5	2.5	2.1	1.6
			Complex with SecB	55.3	45.2	36.1	1.5	1.2	1.0
			Complex with TF (Tig)	58.0	49.0	37.9	1.7	1.4	1.1
P0AAJ3	FdnH	Cell membrane	Monomer	109.0	92.1	71.1	8.6	7.3	5.6
			Complex with SecB	69.6	56.9	45.4	2.8	2.3	1.8
			Complex with TF (Tig)	76.2	64.4	49.7	3.6	3.0	2.3
P0AEK7	FdnI	Cell inner membrane	Monomer	119.8	101.2	78.2	10.7	9.0	7.0
			Complex with SecB	71.5	58.4	46.7	3.0	2.5	2.0
			Complex with TF (Tig)	78.9	66.7	51.5	3.9	3.3	2.5
P32176	FdoG	Periplasm	Monomer	66.8	56.4	43.6	2.5	2.1	1.6
			Complex with SecB	55.4	45.2	36.1	1.5	1.2	1.0
			Complex with TF (Tig)	58.1	49.1	37.9	1.7	1.4	1.1
P0AAJ5	FdoH	Cell membrane	Monomer	107.9	91.2	70.4	8.4	7.1	5.5
			Complex with SecB	69.4	56.7	45.3	2.8	2.3	1.8
			Complex with TF (Tig)	75.9	64.1	49.5	3.5	3.0	2.3
P0AEL0	FdoI	Cell inner membrane	Monomer	121.2	102.4	79.1	11.0	9.3	7.1
			Complex with SecB	71.8	58.6	46.8	3.0	2.5	2.0
			Complex with TF (Tig)	79.2	66.9	51.7	3.9	3.3	2.6
Q47208	FdrA	Cell membrane	Monomer	86.3	72.9	56.3	4.9	4.1	3.2
			Complex with SecB	63.6	51.9	41.5	2.2	1.8	1.4
			Complex with TF (Tig)	68.2	57.6	44.5	2.7	2.2	1.7
P0A9R4	Fdx		Monomer	158.9	134.3	103.7	19.7	16.7	12.9
P80668	FeaB		Monomer	89.3	75.4	58.3	5.3	4.5	3.5
			Homodimer	68.0	55.6	44.4	2.6	2.2	1.7
Q47129	FeaR		Monomer	106.0	89.6	69.2	8.0	6.8	5.3
P13036	FecA	Cell outer membrane	Monomer	74.4	62.9	48.6	3.4	2.8	2.2
			Complex with SecB	59.0	48.2	38.5	1.8	1.5	1.2
			Complex with TF (Tig)	62.5	52.8	40.8	2.1	1.8	1.4

UniProtKB A. N.	Protein name	Subcellular location	Subunit	D_0 $\left(\frac{\mu m^2}{s}\right)$			D_{cyto} $\left(\frac{\mu m^2}{s}\right)$		
				310 K	303 K	293 K	310 K	303 K	293 K
P15028	FecB	Periplasm	Monomer	107.9	91.1	70.4	8.4	7.1	5.5
			Complex with SecB	69.3	56.7	45.3	2.8	2.3	1.8
			Complex with TF (Tig)	75.9	64.1	49.5	3.5	3.0	2.3
P15030	FecC	Cell inner membrane	Monomer	105.7	89.3	69.0	8.0	6.8	5.2
			Complex with SecB	68.9	56.3	45.0	2.7	2.2	1.8
			Complex with TF (Tig)	75.2	63.6	49.1	3.4	2.9	2.2
P15029	FecD	Cell inner membrane	Monomer	106.6	90.1	69.6	8.2	6.9	5.3
			Complex with SecB	69.1	56.4	45.1	2.8	2.3	1.8
			Complex with TF (Tig)	75.5	63.8	49.3	3.5	2.9	2.3
P15031	FecE	Cell inner membrane	Monomer	114.9	97.1	75.0	9.7	8.2	6.3
			Complex with SecB	70.7	57.8	46.1	2.9	2.4	1.9
			Complex with TF (Tig)	77.7	65.7	50.7	3.8	3.2	2.5
P23484	FecI		Monomer	132.8	112.2	86.7	13.4	11.3	8.8
P23485	FecR	Periplasm	Monomer	105.0	88.7	68.5	7.9	6.6	5.1
			Complex with SecB	68.7	56.1	44.8	2.7	2.2	1.8
			Complex with TF (Tig)	75.0	63.4	48.9	3.4	2.9	2.2
P0AEL3	FeoA		Monomer	185.0	156.3	120.7	26.9	22.8	17.6
P33650	FeoB	Cell inner membrane	Monomer	74.7	63.1	48.8	3.4	2.9	2.2
			Complex with SecB	59.1	48.3	38.6	1.8	1.5	1.2
			Complex with TF (Tig)	62.6	52.9	40.9	2.1	1.8	1.4
P64638	FeoC		Monomer	182.5	154.2	119.1	26.2	22.2	17.1
P05825	FepA	Cell outer membrane	Monomer	75.6	63.8	49.3	3.5	2.9	2.3
			Complex with SecB	59.5	48.6	38.8	1.8	1.5	1.2
			Complex with TF (Tig)	63.1	53.3	41.2	2.2	1.8	1.4
P0AEL6	FepB	Periplasm	Monomer	106.4	89.9	69.5	8.1	6.9	5.3
			Complex with SecB	69.0	56.4	45.1	2.8	2.2	1.8
			Complex with TF (Tig)	75.4	63.7	49.2	3.5	2.9	2.3
P23878	FepC	Cell inner membrane	Monomer	112.5	95.0	73.4	9.2	7.8	6.0
			Complex with SecB	70.3	57.4	45.9	2.9	2.4	1.9
			Complex with TF (Tig)	77.1	65.2	50.3	3.7	3.1	2.4
P23876	FepD	Cell inner membrane	Monomer	106.9	90.3	69.8	8.2	6.9	5.4

UniProtKB A. N.	Protein name	Subcellular location	Subunit	D_0 $\left(\frac{\mu m^2}{s}\right)$			D_{cyto} $\left(\frac{\mu m^2}{s}\right)$		
				310 K	303 K	293 K	310 K	303 K	293 K
			Complex with SecB	69.2	56.5	45.1	2.8	2.3	1.8
			Complex with TF (Tig)	75.6	63.9	49.3	3.5	2.9	2.3
P26266	FepE	Cell inner membrane	Monomer	98.2	83.0	64.1	6.7	5.7	4.4
			Complex with SecB	67.1	54.8	43.8	2.5	2.1	1.7
			Complex with TF (Tig)	72.8	61.5	47.5	3.2	2.7	2.1
P23877	FepG	Cell inner membrane	Monomer	105.7	89.3	69.0	8.0	6.7	5.2
			Complex with SecB	68.9	56.3	45.0	2.7	2.2	1.8
			Complex with TF (Tig)	75.2	63.5	49.1	3.4	2.9	2.2
P13039	Fes	Cytoplasm	Monomer	97.8	82.6	63.8	6.6	5.6	4.3
P0AGD7	Ffh		Monomer	92.0	77.7	60.0	5.7	4.8	3.7
Q47153	FhiA	Cell inner membrane	Monomer	83.8	70.8	54.7	4.5	3.8	3.0
			Complex with SecB	62.7	51.2	40.9	2.1	1.7	1.4
			Complex with TF (Tig)	67.1	56.7	43.8	2.5	2.1	1.7
P19323	FhlA		Monomer	76.9	65.0	50.2	3.7	3.1	2.4
P06971	FhuA	Cell outer membrane	Monomer	75.6	63.8	49.3	3.5	2.9	2.3
			Complex with SecB	59.5	48.6	38.8	1.8	1.5	1.2
			Complex with TF (Tig)	63.0	53.3	41.1	2.2	1.8	1.4
P06972	FhuB	Cell inner membrane	Monomer	80.3	67.8	52.4	4.1	3.4	2.7
			Complex with SecB	61.4	50.2	40.1	2.0	1.6	1.3
			Complex with TF (Tig)	65.4	55.3	42.7	2.4	2.0	1.6
P07821	FhuC	Cell inner membrane	Monomer	113.8	96.2	74.3	9.5	8.0	6.2
			Complex with SecB	70.5	57.6	46.0	2.9	2.4	1.9
			Complex with TF (Tig)	77.5	65.5	50.6	3.7	3.1	2.4
P07822	FhuD	Periplasm	Monomer	108.0	91.3	70.5	8.4	7.1	5.5
			Complex with SecB	69.4	56.7	45.3	2.8	2.3	1.8
			Complex with TF (Tig)	75.9	64.1	49.5	3.5	3.0	2.3
P16869	FhuE	Cell outer membrane	Monomer	75.9	64.1	49.5	3.5	3.0	2.3
			Complex with SecB	59.6	48.7	38.9	1.8	1.5	1.2
			Complex with TF (Tig)	63.2	53.4	41.3	2.2	1.8	1.4
P39405	FhuF	Cytoplasm. Cell membrane	Monomer	112.0	94.6	73.1	9.1	7.7	6.0

UniProtKB A. N.	Protein name	Subcellular location	Subunit	D_0 $\left(\frac{\mu m^2}{s}\right)$			D_{cyto} $\left(\frac{\mu m^2}{s}\right)$		
				310 K	303 K	293 K	310 K	303 K	293 K
P20605	Fic		Monomer	124.5	105.2	81.3	11.6	9.8	7.6
P69380	FieF	Cell inner membrane	Monomer	108.1	91.4	70.6	8.4	7.1	5.5
			Complex with SecB	69.4	56.7	45.3	2.8	2.3	1.8
			Complex with TF (Tig)	75.9	64.2	49.6	3.5	3.0	2.3
P04128	FimA	Fimbrium	Monomer	136.7	115.5	89.2	14.3	12.1	9.3
			Complex with SecB	73.8	60.3	48.2	3.3	2.7	2.1
			Complex with TF (Tig)	82.2	69.4	53.6	4.3	3.7	2.8
P0ADH5	FimB		Monomer	124.5	105.2	81.2	11.6	9.8	7.6
P31697	FimC	Periplasm	Monomer	117.4	99.2	76.6	10.2	8.6	6.6
			Complex with SecB	71.1	58.1	46.4	3.0	2.4	1.9
			Complex with TF (Tig)	78.4	66.2	51.1	3.8	3.2	2.5
P30130	FimD	Cell outer membrane	Monomer	70.9	59.9	46.3	3.0	2.5	1.9
			Complex with SecB	57.4	46.9	37.5	1.7	1.4	1.1
			Complex with TF (Tig)	60.5	51.1	39.5	1.9	1.6	1.3
P0ADH7	FimE		Monomer	124.2	104.9	81.1	11.6	9.8	7.6
P08189	FimF	Fimbrium	Monomer	134.9	114.0	88.1	13.9	11.7	9.1
			Complex with SecB	73.6	60.1	48.0	3.3	2.7	2.1
			Complex with TF (Tig)	81.9	69.2	53.4	4.3	3.6	2.8
P08190	FimG	Fimbrium	Monomer	139.1	117.5	90.8	14.9	12.6	9.7
			Complex with SecB	74.1	60.5	48.3	3.3	2.7	2.2
			Complex with TF (Tig)	82.6	69.8	53.9	4.4	3.7	2.9
P08191	FimH	Fimbrium	Monomer	110.1	93.0	71.8	8.8	7.4	5.7
			Complex with SecB	69.8	57.0	45.5	2.8	2.3	1.8
			Complex with TF (Tig)	76.5	64.6	49.9	3.6	3.0	2.3
P39264	FimI	Fimbrium	Monomer	133.1	112.5	86.9	13.5	11.4	8.8
			Complex with SecB	73.4	60.0	47.9	3.2	2.6	2.1
			Complex with TF (Tig)	81.6	68.9	53.2	4.2	3.6	2.8
P0AEL8	FimZ	Cytoplasm	Monomer	123.1	104.0	80.3	11.3	9.6	7.4
P0A6R3	Fis		Monomer	164.8	139.2	107.5	21.3	18.0	13.9
			Homodimer	125.6	102.6	81.9	11.9	9.7	7.7
P75780	Fiu	Cell outer membrane	Monomer	75.6	63.9	49.4	3.5	3.0	2.3

UniProtKB A. N.	Protein name	Subcellular location	Subunit	D_0 $\left(\frac{\mu m^2}{s}\right)$			D_{cyto} $\left(\frac{\mu m^2}{s}\right)$		
				310 K	303 K	293 K	310 K	303 K	293 K
			Complex with SecB	59.5	48.6	38.8	1.8	1.5	1.2
			Complex with TF (Tig)	63.1	53.3	41.2	2.2	1.8	1.4
P60566	FixA		Monomer	116.6	98.5	76.1	10.0	8.5	6.5
			Heterodimer (FixA−FixB)	85.1	71.9	55.5	4.7	4.0	3.1
P31574	FixB		Monomer	107.4	90.7	70.1	8.3	7.0	5.4
			Heterodimer (FixA−FixB)	85.1	71.9	55.5	4.7	4.0	3.1
P68644	FixC		Monomer	95.1	80.3	62.1	6.2	5.2	4.1
P68646	FixX		Monomer	169.4	143.1	110.5	22.5	19.0	14.7
P0A9L3	FklB	Cytoplasm Periplasm	Monomer	126.2	106.6	82.3	12.0	10.1	7.8
			Homodimer	96.1	78.5	62.7	6.4	5.2	4.2
P45523	FkpA	Periplasm	Monomer	113.8	96.2	74.3	9.5	8.0	6.2
			Complex with SecB	70.5	57.6	46.0	2.9	2.4	1.9
			Complex with TF (Tig)	77.5	65.5	50.6	3.7	3.1	2.4
P0AEM0	FkpB		Monomer	143.2	121.0	93.4	15.8	13.4	10.3
P61949	FldA		Monomer	132.2	111.6	86.2	13.3	11.2	8.7
P0ABY4	FldB		Monomer	132.3	111.7	86.3	13.3	11.2	8.7
P75933	FlgA	Periplasm	Monomer	123.4	104.2	80.5	11.4	9.6	7.4
			Complex with SecB	72.1	58.9	47.0	3.1	2.5	2.0
			Complex with TF (Tig)	79.7	67.3	52.0	4.0	3.4	2.6
P0ABW9	FlgB	Bacterial flagellum basal body	Monomer	146.3	123.6	95.4	16.5	14.0	10.8
			Complex with SecB	74.8	61.1	48.8	3.4	2.8	2.2
			Complex with TF (Tig)	83.6	70.6	54.6	4.5	3.8	2.9
P0ABX2	FlgC	Bacterial flagellum basal body	Monomer	151.3	127.9	98.8	17.8	15.0	11.6
			Complex with SecB	75.2	61.5	49.1	3.4	2.8	2.3
			Complex with TF (Tig)	84.3	71.2	55.0	4.6	3.9	3.0
P75936	FlgD		Monomer	123.3	104.1	80.4	11.4	9.6	7.4
P75937	FlgE	Bacterial flagellum basal body	Monomer	98.3	83.0	64.1	6.7	5.7	4.4
			Complex with SecB	67.1	54.8	43.8	2.5	2.1	1.7
			Complex with TF (Tig)	72.8	61.5	47.5	3.2	2.7	2.1
P75938	FlgF	Bacterial flagellum basal body	Monomer	118.8	100.3	77.5	10.5	8.8	6.8
			Complex with SecB	71.4	58.3	46.6	3.0	2.5	2.0

UniProtKB A. N.	Protein name	Subcellular location	Subunit	D_0 $\left(\frac{\mu m^2}{s}\right)$			D_{cyto} $\left(\frac{\mu m^2}{s}\right)$		
				310 K	303 K	293 K	310 K	303 K	293 K
			Complex with TF (Tig)	78.7	66.5	51.3	3.9	3.3	2.5
P0ABX5	FlgG	Bacterial flagellum basal body	Monomer	115.6	97.7	75.5	9.8	8.3	6.4
			Complex with SecB	70.8	57.9	46.2	2.9	2.4	1.9
			Complex with TF (Tig)	77.9	65.8	50.9	3.8	3.2	2.5
P0A6S0	FlgH	Cell outer membrane	Monomer	121.2	102.4	79.1	10.9	9.3	7.1
			Complex with SecB	71.8	58.6	46.8	3.0	2.5	2.0
			Complex with TF (Tig)	79.2	66.9	51.7	3.9	3.3	2.6
P0A6S3	FlgI	Periplasm Bacterial flagellum basal body	Monomer	102.0	86.2	66.6	7.4	6.2	4.8
			Complex with SecB	68.1	55.6	44.4	2.6	2.2	1.7
			Complex with TF (Tig)	74.1	62.6	48.4	3.3	2.8	2.2
P75942	FlgJ	Periplasm	Monomer	106.2	89.7	69.3	8.1	6.8	5.3
			Complex with SecB	69.0	56.4	45.0	2.7	2.2	1.8
			Complex with TF (Tig)	75.4	63.7	49.2	3.5	2.9	2.3
P33235	FlgK	Secreted Bacterial flagellum	Monomer	86.7	73.2	56.5	4.9	4.2	3.2
			Complex with SecB	63.7	52.0	41.6	2.2	1.8	1.4
			Complex with TF (Tig)	68.3	57.7	44.6	2.7	2.3	1.7
P29744	FlgL	Secreted Bacterial flagellum	Monomer	106.4	89.9	69.5	8.1	6.9	5.3
			Complex with SecB	69.0	56.4	45.1	2.8	2.2	1.8
			Complex with TF (Tig)	75.4	63.7	49.2	3.5	2.9	2.3
P0AEM4	FlgM		Monomer	170.3	143.8	111.1	22.8	19.2	14.9
P43533	FlgN	Cytoplasm	Monomer	144.0	121.6	93.9	16.0	13.5	10.4
P76298	FlhA	Cell inner membrane	Monomer	78.4	66.2	51.1	3.8	3.2	2.5
			Complex with SecB	60.6	49.5	39.6	1.9	1.6	1.3
			Complex with TF (Tig)	64.5	54.5	42.1	2.3	1.9	1.5
P76299	FlhB	Cell inner membrane	Monomer	98.1	82.9	64.0	6.7	5.7	4.4
			Complex with SecB	67.1	54.8	43.8	2.5	2.1	1.7
			Complex with TF (Tig)	72.8	61.5	47.5	3.2	2.7	2.1
P0ABY7	FlhC	Cytoplasm	Monomer	127.6	107.8	83.3	12.3	10.4	8.0
			Heterohexamer ($FlhC_2-FlhD_4$)	89.6	75.7	58.4	5.4	4.5	3.5
P0A8S9	FlhD	Cytoplasm	Monomer	154.2	130.3	100.6	18.5	15.6	12.1
			Homodimer	117.5	96.0	76.7	10.2	8.3	6.7

UniProtKB A. N.	Protein name	Subcellular location	Subunit	D_0 $\left(\frac{\mu m^2}{s}\right)$			D_{cyto} $\left(\frac{\mu m^2}{s}\right)$		
				310 K	303 K	293 K	310 K	303 K	293 K
			Heterohexamer ($FlhC_2-FlhD_4$)	89.6	75.7	58.4	5.4	4.5	3.5
P76297	FlhE		Monomer	151.0	127.5	98.5	17.7	15.0	11.6
P0AEM6	FliA		Monomer	116.0	98.0	75.7	9.9	8.4	6.5
P04949	FliC	Secreted Bacterial flagellum	Monomer	90.9	76.8	59.3	5.6	4.7	3.6
			Complex with SecB	65.0	53.1	42.4	2.3	1.9	1.5
			Complex with TF (Tig)	70.1	59.2	45.7	2.9	2.4	1.9
P24216	FliD	Secreted Bacterial flagellum	Monomer	92.9	78.5	60.6	5.9	5.0	3.8
			Complex with SecB	65.7	53.6	42.8	2.4	2.0	1.6
			Complex with TF (Tig)	70.9	59.9	46.3	3.0	2.5	1.9
P0A8T5	FliE	Bacterial flagellum basal body	Monomer	165.4	139.8	108.0	21.5	18.1	14.0
			Complex with SecB	76.3	62.3	49.8	3.6	2.9	2.3
			Complex with TF (Tig)	85.8	72.5	56.0	4.8	4.1	3.2
P25798	FliF	Cell inner membrane	Monomer	85.1	71.9	55.6	4.7	4.0	3.1
			Complex with SecB	63.2	51.6	41.2	2.2	1.8	1.4
			Complex with TF (Tig)	67.7	57.2	44.2	2.6	2.2	1.7
P0ABZ1	FliG	Cell inner membrane	Monomer	103.5	87.5	67.6	7.6	6.4	5.0
P31068	FliH	Cytoplasm	Monomer	120.4	101.7	78.5	10.8	9.1	7.0
P52612	FliI	Cytoplasm	Monomer	92.3	78.0	60.2	5.8	4.9	3.8
P52613	FliJ	Cell membrane	Monomer	139.1	117.5	90.8	14.9	12.6	9.7
P52614	FliK		Monomer	100.9	85.2	65.8	7.2	6.0	4.7
P0ABX8	FliL	Cell inner membrane	Monomer	139.4	117.8	91.0	14.9	12.6	9.7
			Complex with SecB	74.1	60.5	48.4	3.3	2.7	2.2
			Complex with TF (Tig)	82.6	69.8	53.9	4.4	3.7	2.9
P06974	FliM	Cell inner membrane	Monomer	102.4	86.5	66.8	7.4	6.3	4.8
			Complex with SecB	68.1	55.7	44.5	2.7	2.2	1.7
			Complex with TF (Tig)	74.2	62.7	48.4	3.3	2.8	2.2
P15070	FliN	Cell inner membrane	Monomer	147.7	124.8	96.4	16.9	14.3	11.0
P22586	FliO	Cell membrane	Monomer	157.2	132.8	102.6	19.3	16.3	12.6
			Complex with SecB	75.7	61.8	49.4	3.5	2.9	2.3
			Complex with TF (Tig)	85.0	71.8	55.5	4.7	4.0	3.1
P0AC05	FliP	Cell inner membrane	Monomer	117.0	98.8	76.3	10.1	8.5	6.6

UniProtKB A. N.	Protein name	Subcellular location	Subunit	D_0 $\left(\frac{\mu m^2}{s}\right)$ 310 K	D_0 303 K	D_0 293 K	D_{cyto} $\left(\frac{\mu m^2}{s}\right)$ 310 K	D_{cyto} 303 K	D_{cyto} 293 K
			Complex with SecB	71.1	58.1	46.4	3.0	2.4	1.9
			Complex with TF (Tig)	78.3	66.1	51.1	3.8	3.2	2.5
P0AC07	FliQ	Cell inner membrane	Monomer	175.1	147.9	114.2	24.1	20.4	15.7
			Complex with SecB	76.8	62.8	50.1	3.6	3.0	2.4
			Complex with TF (Tig)	86.7	73.3	56.6	5.0	4.2	3.2
P33135	FliR	Cell inner membrane	Monomer	114.4	96.6	74.6	9.6	8.1	6.3
			Complex with SecB	70.6	57.7	46.1	2.9	2.4	1.9
			Complex with TF (Tig)	77.6	65.6	50.6	3.7	3.2	2.4
P26608	FliS	Cytoplasm > cytosol	Monomer	147.4	124.5	96.2	16.8	14.2	11.0
P0ABY2	FliT	Cytoplasm > cytosol	Monomer	151.9	128.4	99.1	17.9	15.2	11.7
			Homodimer	115.8	94.6	75.6	9.9	8.1	6.4
P0AEM9	FliY	Periplasm	Monomer	113.6	96.0	74.1	9.4	8.0	6.2
			Complex with SecB	70.5	57.6	46.0	2.9	2.4	1.9
			Complex with TF (Tig)	77.4	65.4	50.5	3.7	3.1	2.4
P52627	FliZ		Monomer	127.4	107.7	83.2	12.3	10.4	8.0
P15286	Flk	Cell inner membrane	Monomer	103.7	87.6	67.6	7.6	6.4	5.0
			Complex with SecB	68.4	55.9	44.7	2.7	2.2	1.8
			Complex with TF (Tig)	74.6	63.0	48.7	3.4	2.9	2.2
P39180	Flu	Periplasm. Secreted. Cell surface. Cell outer membrane	Monomer	68.2	57.6	44.5	2.7	2.2	1.7
			Complex with SecB	56.1	45.8	36.6	1.5	1.3	1.0
			Complex with TF (Tig)	58.9	49.8	38.4	1.8	1.5	1.2
P77609	FlxA		Monomer	159.5	134.8	104.1	19.9	16.8	13.0
P23882	Fmt		Monomer	106.6	90.0	69.5	8.1	6.9	5.3
P0A9E5	Fnr	Cytoplasm	Monomer	115.3	97.4	75.2	9.8	8.3	6.4
			Homodimer	87.9	71.8	57.3	5.1	4.2	3.3
P0AC23	FocA	Cell inner membrane	Monomer	110.7	93.5	72.3	8.9	7.5	5.8
			Complex with SecB	69.9	57.1	45.6	2.8	2.3	1.9
			Complex with TF (Tig)	76.7	64.8	50.0	3.6	3.1	2.4
P77733	FocB	Cell inner membrane	Monomer	111.3	94.1	72.7	9.0	7.6	5.9
			Complex with SecB	70.0	57.2	45.7	2.9	2.3	1.9
			Complex with TF (Tig)	76.8	64.9	50.1	3.6	3.1	2.4

UniProtKB A. N.	Protein name	Subcellular location	Subunit	D_0 $\left(\frac{\mu m^2}{s}\right)$			D_{cyto} $\left(\frac{\mu m^2}{s}\right)$		
				310 K	303 K	293 K	310 K	303 K	293 K
P0ABQ4	FolA		Monomer	137.0	115.8	89.4	14.4	12.1	9.4
P0AC16	FolB		Monomer	152.8	129.1	99.7	18.2	15.4	11.9
			Homooctamer	67.6	55.3	44.1	2.6	2.1	1.7
P08192	FolC		Monomer	95.3	80.5	62.2	6.2	5.3	4.1
P24186	FolD		Monomer	110.7	93.5	72.2	8.9	7.5	5.8
			Homodimer	84.3	68.9	55.0	4.6	3.8	3.0
P0A6T5	FolE		Monomer	120.8	102.0	78.8	10.9	9.2	7.1
			Homodecamer	49.0	40.0	32.0	1.0	0.9	0.7
P26281	FolK		Monomer	136.8	115.6	89.3	14.3	12.1	9.3
P0AFS3	FolM		Monomer	118.0	99.7	77.0	10.3	8.7	6.7
P0AC13	FolP		Monomer	111.3	94.0	72.6	9.0	7.6	5.9
			Homodimer	84.8	69.3	55.3	4.7	3.8	3.1
P0AC19	FolX		Monomer	150.9	127.4	98.4	17.7	14.9	11.5
			Homooctamer	66.8	54.5	43.6	2.5	2.1	1.6
P28861	Fpr		Monomer	115.6	97.7	75.5	9.8	8.3	6.4
P69902	Frc		Monomer	95.0	80.2	62.0	6.2	5.2	4.0
			Homodimer	72.4	59.1	47.2	3.1	2.5	2.0
P00363	FrdA		Monomer	82.3	69.6	53.7	4.3	3.7	2.8
P0AC47	FrdB		Monomer	116.7	98.6	76.1	10.0	8.5	6.6
P0A8Q0	FrdC	Cell inner membrane	Monomer	147.1	124.3	96.0	16.8	14.2	10.9
			Complex with SecB	74.9	61.2	48.9	3.4	2.8	2.2
			Complex with TF (Tig)	83.7	70.7	54.6	4.5	3.8	3.0
P0A8Q3	FrdD	Cell inner membrane	Monomer	155.2	131.1	101.2	18.8	15.9	12.2
			Complex with SecB	75.5	61.7	49.3	3.5	2.8	2.3
			Complex with TF (Tig)	84.8	71.6	55.3	4.7	3.9	3.1
P0AEN1	Fre		Monomer	118.2	99.8	77.1	10.3	8.7	6.8
P45539	FrlA	Cell inner membrane	Monomer	93.6	79.1	61.1	6.0	5.0	3.9
			Complex with SecB	65.9	53.8	43.0	2.4	2.0	1.6
			Complex with TF (Tig)	71.2	60.1	46.4	3.0	2.5	1.9
P0AC00	FrlB		Monomer	101.6	85.9	66.3	7.3	6.2	4.8
			Homododecamer	38.4	31.3	25.0	0.5	0.4	0.3

UniProtKB A. N.	Protein name	Subcellular location	Subunit	D_0 $\left(\frac{\mu m^2}{s}\right)$			D_{cyto} $\left(\frac{\mu m^2}{s}\right)$		
				310 K	303 K	293 K	310 K	303 K	293 K
P45541	FrlC		Monomer	110.5	93.3	72.1	8.9	7.5	5.8
P45543	FrlD		Monomer	114.7	96.9	74.8	9.7	8.2	6.3
P45544	FrlR		Monomer	115.5	97.6	75.4	9.8	8.3	6.4
P25437	FrmA	Cytoplasm	Monomer	100.8	85.2	65.8	7.1	6.0	4.7
			Homodimer	76.8	62.8	50.1	3.6	3.0	2.4
P51025	FrmB		Monomer	110.1	93.0	71.9	8.8	7.4	5.7
P0AAP3	FrmR	Cytoplasm	Monomer	170.4	144.0	111.2	22.8	19.3	14.9
P0A805	Frr	Cytoplasm	Monomer	129.9	109.7	84.7	12.8	10.8	8.3
P04335	FrsA		Monomer	94.0	79.5	61.4	6.0	5.1	3.9
P20966	FruA	Cell inner membrane	Monomer	86.9	73.4	56.7	5.0	4.2	3.2
			Complex with SecB	63.8	52.1	41.6	2.2	1.8	1.4
			Complex with TF (Tig)	68.5	57.8	44.7	2.7	2.3	1.8
P69811	FruB	Cytoplasm	Monomer	100.5	84.9	65.6	7.1	6.0	4.6
P0AEW9	FruK		Monomer	107.1	90.5	69.9	8.2	7.0	5.4
P0ADF0	FruL		Monomer	266.7	225.3	174.0	54.4	45.9	35.5
P0ACP1	FruR		Monomer	102.2	86.4	66.7	7.4	6.2	4.8
P32155	FrvA	Cytoplasm	Monomer	143.2	120.9	93.4	15.8	13.4	10.3
P32154	FrvB	Cell inner membrane	Monomer	91.0	76.9	59.4	5.6	4.7	3.6
			Complex with SecB	65.1	53.2	42.5	2.3	1.9	1.5
			Complex with TF (Tig)	70.1	59.3	45.8	2.9	2.4	1.9
P32152	FrvR		Monomer	82.3	69.6	53.7	4.3	3.7	2.8
P32153	FrvX		Monomer	101.5	85.7	66.2	7.3	6.1	4.7
P69816	FrwB	Cytoplasm	Monomer	164.7	139.2	107.5	21.3	18.0	13.9
P32672	FrwC	Cell inner membrane	Monomer	103.2	87.2	67.3	7.6	6.4	4.9
			Complex with SecB	68.3	55.8	44.6	2.7	2.2	1.7
			Complex with TF (Tig)	74.5	62.9	48.6	3.4	2.8	2.2
P32676	FrwD	Cytoplasm	Monomer	157.4	133.0	102.7	19.3	16.3	12.6
P77439	FryA	Cytoplasm	Monomer	72.2	61.0	47.1	3.1	2.6	2.0
P69808	FryB	Cytoplasm	Monomer	162.0	136.9	105.7	20.5	17.4	13.4
P77579	FryC	Cell inner membrane	Monomer	96.5	81.6	63.0	6.4	5.4	4.2
			Complex with SecB	66.7	54.5	43.5	2.5	2.0	1.6

UniProtKB A. N.	Protein name	Subcellular location	Subunit	D_0 $\left(\frac{\mu m^2}{s}\right)$			D_{cyto} $\left(\frac{\mu m^2}{s}\right)$		
				310 K	303 K	293 K	310 K	303 K	293 K
			Complex with TF (Tig)	72.2	61.0	47.1	3.1	2.6	2.0
P78055	FsaA	Cytoplasm	Monomer	124.5	105.2	81.2	11.6	9.8	7.6
			Homododecamer	47.0	38.4	30.7	0.9	0.8	0.6
P32669	FsaB	Cytoplasm	Monomer	123.3	104.2	80.5	11.4	9.6	7.4
P52067	Fsr	Cell inner membrane	Monomer	97.1	82.0	63.4	6.5	5.5	4.3
			Complex with SecB	66.8	54.6	43.6	2.5	2.1	1.6
			Complex with TF (Tig)	72.4	61.2	47.3	3.1	2.6	2.0
P0A998	FtnA	Cytoplasm	Monomer	133.0	112.3	86.8	13.5	11.4	8.8
			Homooligomer	38.3	32.3	25.0	0.5	0.4	0.3
P0A9A2	FtnB	Cytoplasm	Monomer	134.4	113.6	87.7	13.8	11.6	9.0
P0ABH0	FtsA		Monomer	95.4	80.6	62.3	6.3	5.3	4.1
P0A6S5	FtsB	Cell inner membrane	Monomer	162.6	137.4	106.1	20.7	17.5	13.5
			Complex with SecB	76.1	62.2	49.6	3.6	2.9	2.3
			Complex with TF (Tig)	85.6	72.3	55.8	4.8	4.0	3.1
P0A9R7	FtsE		Monomer	121.5	102.7	79.3	11.0	9.3	7.2
P0AAI3	FtsH	Cell inner membrane	Monomer	80.1	67.7	52.3	4.1	3.4	2.6
			Complex with SecB	61.3	50.1	40.0	2.0	1.6	1.3
			Complex with TF (Tig)	65.4	55.2	42.7	2.4	2.0	1.5
P0AD68	FtsI	Cell inner membrane	Monomer	83.4	70.5	54.4	4.5	3.8	2.9
			Complex with SecB	62.6	51.1	40.8	2.1	1.7	1.4
			Complex with TF (Tig)	66.9	56.5	43.7	2.5	2.1	1.6
P46889	FtsK	Cell inner membrane	Monomer	60.2	50.9	39.3	1.9	1.6	1.2
			Homohexamer	29.8	24.4	19.5	0.2	0.2	0.1
P0AEN4	FtsL	Cell inner membrane	Monomer	152.8	129.1	99.7	18.2	15.3	11.9
			Complex with SecB	75.4	61.6	49.2	3.5	2.8	2.3
			Complex with TF (Tig)	84.5	71.4	55.1	4.6	3.9	3.0
P29131	FtsN	Cell inner membrane	Monomer	104.7	88.4	68.3	7.8	6.6	5.1
			Complex with SecB	68.7	56.1	44.8	2.7	2.2	1.8
			Complex with TF (Tig)	74.9	63.3	48.9	3.4	2.9	2.2
P06136	FtsQ	Cell inner membrane	Monomer	110.1	93.0	71.9	8.8	7.4	5.7
			Complex with SecB	69.8	57.0	45.6	2.8	2.3	1.8

UniProtKB A. N.	Protein name	Subcellular location	Subunit	D_0 $\left(\frac{\mu m^2}{s}\right)$			D_{cyto} $\left(\frac{\mu m^2}{s}\right)$		
				310 K	303 K	293 K	310 K	303 K	293 K
			Complex with TF (Tig)	76.5	64.6	49.9	3.6	3.0	2.3
P0ABG4	FtsW	Cell inner membrane	Monomer	94.9	80.1	61.9	6.2	5.2	4.0
			Complex with SecB	66.2	54.1	43.2	2.5	2.0	1.6
			Complex with TF (Tig)	71.6	60.5	46.7	3.0	2.6	2.0
P0AC30	FtsX	Cell inner membrane	Monomer	101.7	85.9	66.3	7.3	6.2	4.8
			Complex with SecB	68.0	55.5	44.4	2.6	2.2	1.7
			Complex with TF (Tig)	74.0	62.5	48.3	3.3	2.8	2.2
P10121	FtsY	Cell inner membrane	Monomer	88.7	75.0	57.9	5.2	4.4	3.4
			Complex with SecB	64.4	52.6	42.0	2.3	1.9	1.5
			Complex with TF (Tig)	69.2	58.5	45.2	2.8	2.3	1.8
P0A9A6	FtsZ	Cytoplasm	Monomer	99.9	84.4	65.2	7.0	5.9	4.6
P0AB87	FucA		Monomer	122.9	103.8	80.2	11.3	9.5	7.4
			Homotetramer	71.3	58.3	46.6	3.0	2.5	2.0
P69922	FucI	Cytoplasm	Monomer	82.8	70.0	54.1	4.4	3.7	2.9
			Homohexamer	41.0	33.5	26.8	0.6	0.5	0.4
P11553	FucK		Monomer	89.6	75.7	58.4	5.4	4.5	3.5
P0A9S1	FucO		Monomer	99.6	84.1	65.0	6.9	5.9	4.5
P11551	FucP	Cell inner membrane	Monomer	93.6	79.1	61.1	6.0	5.1	3.9
			Complex with SecB	65.9	53.8	43.0	2.4	2.0	1.6
			Complex with TF (Tig)	71.2	60.1	46.4	3.0	2.5	1.9
P0ACK8	FucR		Monomer	116.3	98.2	75.9	10.0	8.4	6.5
P0AEN8	FucU	Cytoplasm	Monomer	145.4	122.8	94.9	16.3	13.8	10.7
			Homodecamer	59.0	48.2	38.5	1.8	1.5	1.2
P0AC33	FumA		Monomer	85.3	72.1	55.7	4.8	4.0	3.1
			Homodimer	65.0	53.1	42.4	2.3	1.9	1.5
P14407	FumB		Monomer	85.4	72.2	55.7	4.8	4.0	3.1
			Homodimer	65.1	53.2	42.5	2.3	1.9	1.5
P05042	FumC	Cytoplasm	Monomer	91.4	77.3	59.7	5.6	4.8	3.7
			Homotetramer	53.1	43.4	34.7	1.3	1.1	0.9
P0A9A9	Fur	Cytoplasm	Monomer	140.8	118.9	91.9	15.2	12.9	9.9
			Homodimer	107.3	87.6	70.0	8.3	6.8	5.4

UniProtKB A. N.	Protein name	Subcellular location	Subunit	$D_0 \left(\frac{\mu m^2}{s}\right)$			$D_{\text{cyto}} \left(\frac{\mu m^2}{s}\right)$		
				310 K	303 K	293 K	310 K	303 K	293 K
P0A6M8	FusA	Cytoplasm	Monomer	77.3	65.3	50.4	3.7	3.1	2.4
P37147	FxsA	Cell inner membrane	Monomer	137.9	116.5	90.0	14.6	12.3	9.5
			Complex with SecB	73.9	60.4	48.3	3.3	2.7	2.2
			Complex with TF (Tig)	82.4	69.6	53.8	4.4	3.7	2.8
P25526	GabD		Monomer	90.6	76.5	59.1	5.5	4.7	3.6
			Homotetramer	52.6	43.0	34.3	1.3	1.1	0.8
P25527	GabP	Cell inner membrane	Monomer	91.0	76.9	59.4	5.6	4.7	3.6
			Complex with SecB	65.1	53.2	42.5	2.3	1.9	1.5
			Complex with TF (Tig)	70.2	59.3	45.8	2.9	2.4	1.9
P22256	GabT		Monomer	95.0	80.3	62.0	6.2	5.2	4.0
			Homotetramer	55.2	45.1	36.0	1.5	1.2	1.0
P69908	GadA		Monomer	89.9	76.0	58.7	5.4	4.6	3.5
			Homohexamer	44.6	36.4	29.1	0.8	0.6	0.5
P69910	GadB	Cytoplasm Membrane	Monomer	89.9	76.0	58.7	5.4	4.6	3.5
			Homohexamer	44.6	36.4	29.1	0.8	0.6	0.5
P63235	GadC	Cell inner membrane	Monomer	88.4	74.7	57.7	5.2	4.4	3.4
			Complex with SecB	64.3	52.5	41.9	2.3	1.9	1.5
			Complex with TF (Tig)	69.1	58.4	45.1	2.8	2.3	1.8
P63204	GadE		Monomer	130.0	109.8	84.8	12.8	10.8	8.4
P63201	GadW		Monomer	115.2	97.3	75.2	9.8	8.2	6.4
			Homodimer	87.8	71.7	57.3	5.1	4.2	3.3
P37639	GadX		Monomer	109.9	92.9	71.7	8.8	7.4	5.7
			Homodimer	83.8	68.4	54.7	4.5	3.7	3.0
P09147	GalE		Monomer	103.0	87.0	67.2	7.5	6.4	4.9
			Homodimer	78.5	64.1	51.2	3.8	3.1	2.5
P0AAB6	GalF		Monomer	108.3	91.5	70.6	8.5	7.1	5.5
P0A6T3	GalK	Cytoplasm	Monomer	98.8	83.5	64.5	6.8	5.8	4.4
P0A9C3	GalM	Cytoplasm	Monomer	102.0	86.2	66.6	7.4	6.2	4.8
P0AEP1	GalP	Cell inner membrane	Monomer	91.1	77.0	59.4	5.6	4.7	3.6
			Complex with SecB	65.1	53.2	42.5	2.3	1.9	1.5
			Complex with TF (Tig)	70.2	59.3	45.8	2.9	2.4	1.9

UniProtKB A. N.	Protein name	Subcellular location	Subunit	D_0 $\left(\frac{\mu m^2}{s}\right)$			D_{cyto} $\left(\frac{\mu m^2}{s}\right)$		
				310 K	303 K	293 K	310 K	303 K	293 K
P03024	GalR		Monomer	103.2	87.2	67.3	7.6	6.4	4.9
			Homodimer	78.6	64.2	51.3	3.9	3.2	2.5
P25748	GalS		Monomer	102.9	86.9	67.2	7.5	6.3	4.9
			Homodimer	78.4	64.1	51.2	3.8	3.1	2.5
P09148	GalT		Monomer	100.5	84.9	65.6	7.1	6.0	4.6
			Homodimer	76.6	62.6	50.0	3.6	3.0	2.4
P0AEP3	GalU		Monomer	108.1	91.3	70.5	8.4	7.1	5.5
			Homotetramer	62.8	51.3	41.0	2.1	1.7	1.4
			Homopentamer	57.5	48.6	37.5	1.7	1.4	1.1
P0A9B2	GapA	Cytoplasm	Monomer	105.0	88.7	68.5	7.9	6.6	5.1
			Homotetramer	61.0	49.8	39.8	2.0	1.6	1.3
P33898	GapC	Cytoplasm	Monomer	104.8	88.5	68.4	7.8	6.6	5.1
			Homotetramer	60.9	49.7	39.7	2.0	1.6	1.3
P39829	GarD		Monomer	87.6	74.0	57.1	5.1	4.3	3.3
P23524	GarK		Monomer	101.1	85.4	66.0	7.2	6.1	4.7
P23522	GarL		Monomer	116.2	98.2	75.8	10.0	8.4	6.5
			Homohexamer	57.6	47.0	37.6	1.7	1.4	1.1
P0AA80	GarP	Cell inner membrane	Monomer	92.5	78.2	60.4	5.8	4.9	3.8
			Complex with SecB	65.5	53.5	42.8	2.4	2.0	1.6
			Complex with TF (Tig)	70.7	59.8	46.2	2.9	2.5	1.9
P0ABQ2	GarR		Monomer	111.5	94.2	72.8	9.1	7.7	5.9
P69828	GatA	Cytoplasm	Monomer	140.4	118.6	91.6	15.2	12.8	9.9
P37188	GatB	Cytoplasm	Monomer	171.0	144.5	111.6	23.0	19.4	15.0
P69831	GatC	Cell inner membrane	Monomer	93.0	78.6	60.7	5.9	5.0	3.8
			Complex with SecB	65.7	53.7	42.9	2.4	2.0	1.6
			Complex with TF (Tig)	70.9	59.9	46.3	3.0	2.5	1.9
P0A9S3	GatD		Monomer	102.9	86.9	67.1	7.5	6.3	4.9
P36930	GatR		Monomer	115.3	97.4	75.2	9.8	8.3	6.4
P0C8J6	GatY		Monomer	111.0	93.8	72.4	9.0	7.6	5.8
P0C8J8	GatZ		Monomer	94.0	79.4	61.3	6.0	5.1	3.9
P15877	Gcd	Cell inner membrane	Monomer	74.0	62.5	48.3	3.3	2.8	2.2

UniProtKB A. N.	Protein name	Subcellular location	Subunit	D_0 $\left(\frac{\mu m^2}{s}\right)$			D_{cyto} $\left(\frac{\mu m^2}{s}\right)$		
				310 K	303 K	293 K	310 K	303 K	293 K
			Complex with SecB	58.8	48.0	38.4	1.8	1.4	1.2
			Complex with TF (Tig)	62.2	52.6	40.6	2.1	1.8	1.4
P0AEP7	Gcl		Monomer	83.0	70.1	54.1	4.4	3.7	2.9
			Homotetramer	48.2	39.4	31.4	1.0	0.8	0.6
P05852	Gcp	Cytoplasm	Monomer	104.4	88.2	68.1	7.8	6.6	5.1
P0A9F6	GcvA	Cytoplasm	Monomer	106.3	89.8	69.4	8.1	6.8	5.3
P0A6T9	GcvH		Monomer	152.0	128.4	99.2	18.0	15.2	11.7
P33195	GcvP		Monomer	68.8	58.1	44.9	2.7	2.3	1.8
P0A9I3	GcvR	Cytoplasm	Monomer	129.5	109.4	84.5	12.7	10.7	8.3
P27248	GcvT		Monomer	100.0	84.5	65.3	7.0	5.9	4.6
P00370	GdhA		Monomer	92.8	78.4	60.6	5.9	4.9	3.8
			Homohexamer	46.0	37.6	30.0	0.9	0.7	0.6
P75885	GfcA	Cell inner membrane	Monomer	176.0	148.7	114.8	24.3	20.6	15.9
			Complex with SecB	76.9	62.8	50.2	3.6	3.0	2.4
			Complex with TF (Tig)	86.8	73.3	56.6	5.0	4.2	3.2
P75884	GfcB	Cell membrane	Monomer	121.9	103.0	79.5	11.1	9.4	7.2
			Complex with SecB	71.9	58.7	46.9	3.1	2.5	2.0
			Complex with TF (Tig)	79.4	67.1	51.8	4.0	3.3	2.6
P75883	GfcC		Monomer	116.4	98.3	75.9	10.0	8.4	6.5
P75882	GfcD	Cell membrane	Monomer	76.8	64.9	50.1	3.6	3.1	2.4
			Complex with SecB	60.0	49.0	39.2	1.9	1.5	1.2
			Complex with TF (Tig)	63.7	53.8	41.6	2.2	1.9	1.4
P0A932	GfcE	Cell outer membrane	Monomer	98.5	83.2	64.3	6.8	5.7	4.4
			Complex with SecB	67.2	54.9	43.8	2.6	2.1	1.7
			Complex with TF (Tig)	72.9	61.6	47.6	3.2	2.7	2.1
P18956	Ggt	Periplasm	Monomer	84.5	71.4	55.1	4.6	3.9	3.0
			Complex with SecB	63.0	51.4	41.1	2.1	1.7	1.4
			Complex with TF (Tig)	67.4	56.9	44.0	2.6	2.2	1.7
P75913	GhrA	Cytoplasm	Monomer	105.2	88.9	68.6	7.9	6.7	5.2
P37666	GhrB	Cytoplasm	Monomer	105.1	88.8	68.6	7.9	6.7	5.1
			Homodimer	80.1	65.4	52.3	4.1	3.3	2.6

UniProtKB A. N.	Protein name	Subcellular location	Subunit	D_0 $\left(\frac{\mu m^2}{s}\right)$			D_{cyto} $\left(\frac{\mu m^2}{s}\right)$		
				310 K	303 K	293 K	310 K	303 K	293 K
Q46839	GlcA	Cell inner membrane	Monomer	86.1	72.7	56.2	4.9	4.1	3.2
			Complex with SecB	63.5	51.9	41.4	2.2	1.8	1.4
			Complex with TF (Tig)	68.1	57.5	44.4	2.7	2.2	1.7
P37330	GlcB	Cytoplasm	Monomer	76.2	64.3	49.7	3.6	3.0	2.3
P0ACL5	GlcC		Monomer	113.9	96.2	74.3	9.5	8.0	6.2
P0AEP9	GlcD		Monomer	89.2	75.4	58.2	5.3	4.5	3.5
P52073	GlcE		Monomer	101.8	86.0	66.5	7.3	6.2	4.8
P52074	GlcF		Monomer	95.6	80.7	62.4	6.3	5.3	4.1
P0AEQ1	GlcG		Monomer	152.3	128.7	99.4	18.0	15.2	11.8
P0A9S5	GldA		Monomer	101.5	85.7	66.2	7.3	6.1	4.7
			Homodimer	77.3	63.2	50.5	3.7	3.0	2.4
			Homooctamer	44.9	37.9	29.3	0.8	0.7	0.5
P37747	Glf		Monomer	97.4	82.3	63.6	6.6	5.6	4.3
P0A6U8	GlgA		Monomer	89.8	75.9	58.6	5.4	4.6	3.5
P07762	GlgB		Monomer	74.8	63.2	48.8	3.4	2.9	2.2
P0A6V1	GlgC		Monomer	92.8	78.4	60.5	5.8	4.9	3.8
			Homotetramer	53.9	44.0	35.2	1.4	1.1	0.9
P0AC86	GlgP		Monomer	71.9	60.8	46.9	3.1	2.6	2.0
P26649	GlgS		Monomer	189.3	159.9	123.5	28.2	23.8	18.4
P15067	GlgX		Monomer	78.9	66.7	51.5	3.9	3.3	2.5
P0A6V8	Glk	Cytoplasm	Monomer	105.9	89.5	69.1	8.0	6.8	5.2
P31120	GlmM		Monomer	93.6	79.1	61.1	6.0	5.1	3.9
P17169	GlmS	Cytoplasm	Monomer	81.9	69.2	53.4	4.3	3.6	2.8
			Homodimer	62.4	51.0	40.7	2.1	1.7	1.4
P0ACC7	GlmU	Cytoplasm	Monomer	92.4	78.1	60.3	5.8	4.9	3.8
			Homotrimer	60.1	49.1	39.2	1.9	1.5	1.2
			Homohexamer	45.8	38.7	29.9	0.9	0.7	0.6
P0A9C5	GlnA	Cytoplasm	Monomer	90.5	76.4	59.0	5.5	4.6	3.6
P0A9Z1	GlnB		Monomer	158.4	133.9	103.4	19.6	16.6	12.8
			Homotrimer	103.0	84.1	67.2	7.5	6.1	4.9
P27249	GlnD		Monomer	69.3	58.6	45.2	2.8	2.3	1.8

UniProtKB A. N.	Protein name	Subcellular location	Subunit	D_0 $\left(\frac{\mu m^2}{s}\right)$			D_{cyto} $\left(\frac{\mu m^2}{s}\right)$		
				310 K	303 K	293 K	310 K	303 K	293 K
P30870	GlnE		Monomer	67.8	57.3	44.2	2.6	2.2	1.7
P0AFB8	GlnG		Monomer	90.2	76.2	58.9	5.5	4.6	3.6
P0AEQ3	GlnH	Periplasm	Monomer	116.6	98.5	76.1	10.0	8.5	6.5
			Complex with SecB	71.0	58.0	46.3	3.0	2.4	1.9
			Complex with TF (Tig)	78.2	66.0	51.0	3.8	3.2	2.5
P0AC55	GlnK		Monomer	159.3	134.6	103.9	19.8	16.7	12.9
			Homotrimer	103.5	84.6	67.6	7.6	6.2	5.0
P0AFB5	GlnL		Monomer	101.6	85.9	66.3	7.3	6.2	4.8
P0AEQ6	GlnP	Cell inner membrane	Monomer	121.7	102.8	79.4	11.0	9.3	7.2
			Complex with SecB	71.8	58.7	46.9	3.1	2.5	2.0
			Complex with TF (Tig)	79.3	67.0	51.8	4.0	3.3	2.6
P10346	GlnQ	Cell inner membrane	Monomer	117.3	99.1	76.6	10.2	8.6	6.6
			Complex with SecB	71.1	58.1	46.4	3.0	2.4	1.9
			Complex with TF (Tig)	78.3	66.2	51.1	3.8	3.2	2.5
P00962	GlnS	Cytoplasm	Monomer	83.6	70.6	54.6	4.5	3.8	2.9
P0AC81	GloA		Monomer	147.5	124.6	96.2	16.8	14.2	11.0
			Homodimer	112.4	91.8	73.3	9.2	7.5	6.0
P0AC84	GloB		Monomer	114.5	96.8	74.7	9.6	8.1	6.3
P0A9C0	GlpA	Cell inner membrane	Monomer	86.1	72.7	56.2	4.9	4.1	3.2
			Complex with SecB	63.5	51.9	41.4	2.2	1.8	1.4
			Complex with TF (Tig)	68.1	57.5	44.4	2.6	2.2	1.7
P13033	GlpB	Cell inner membrane	Monomer	95.4	80.6	62.2	6.3	5.3	4.1
			Complex with SecB	66.3	54.2	43.3	2.5	2.0	1.6
			Complex with TF (Tig)	71.8	60.7	46.9	3.1	2.6	2.0
P0A996	GlpC	Cell inner membrane	Monomer	96.4	81.5	62.9	6.4	5.4	4.2
			Complex with SecB	66.6	54.4	43.5	2.5	2.0	1.6
			Complex with TF (Tig)	72.2	61.0	47.1	3.1	2.6	2.0
P13035	GlpD	Cytoplasm	Monomer	87.4	73.8	57.0	5.0	4.3	3.3
P0A6V5	GlpE	Cytoplasm	Monomer	160.2	135.3	104.5	20.1	16.9	13.1
			Homodimer	122.1	99.7	79.7	11.1	9.1	7.3
P0AER0	GlpF	Cell inner membrane	Monomer	112.5	95.0	73.4	9.2	7.8	6.0

UniProtKB A. N.	Protein name	Subcellular location	Subunit	D_0 $\left(\frac{\mu m^2}{s}\right)$ 310 K	303 K	293 K	D_{cyto} $\left(\frac{\mu m^2}{s}\right)$ 310 K	303 K	293 K
			Complex with SecB	70.3	57.4	45.9	2.9	2.4	1.9
			Complex with TF (Tig)	77.1	65.2	50.3	3.7	3.1	2.4
P09391	GlpG	Cell inner membrane	Monomer	110.3	93.2	72.0	8.8	7.5	5.8
			Complex with SecB	69.8	57.1	45.6	2.8	2.3	1.9
			Complex with TF (Tig)	76.5	64.7	49.9	3.6	3.0	2.4
P0A6F3	GlpK		Monomer	87.7	74.1	57.2	5.1	4.3	3.3
			Homotetramer	50.9	41.6	33.2	1.2	1.0	0.8
			Heterodimer (Crr−GlpK)	78.5	66.3	51.2	3.9	3.3	2.5
P09394	GlpQ	Periplasm	Monomer	99.4	84.0	64.8	6.9	5.8	4.5
			Complex with SecB	67.4	55.1	44.0	2.6	2.1	1.7
			Complex with TF (Tig)	73.2	61.9	47.8	3.2	2.7	2.1
P0ACL0	GlpR		Monomer	115.1	97.3	75.1	9.7	8.2	6.4
P08194	GlpT	Cell inner membrane	Monomer	91.6	77.4	59.8	5.7	4.8	3.7
			Complex with SecB	65.3	53.3	42.6	2.4	1.9	1.5
			Complex with TF (Tig)	70.4	59.5	45.9	2.9	2.4	1.9
P0A9C9	GlpX	Cytoplasm	Monomer	104.6	88.4	68.2	7.8	6.6	5.1
			Homodimer	79.7	65.1	52.0	4.0	3.3	2.6
P77454	GlsA1		Monomer	108.2	91.4	70.6	8.4	7.1	5.5
P0A6W0	GlsA2		Monomer	107.4	90.7	70.1	8.3	7.0	5.4
P0ABH7	GltA		Monomer	93.3	78.8	60.9	5.9	5.0	3.9
			Homohexamer	46.2	37.7	30.2	0.9	0.7	0.6
P09831	GltB		Monomer	57.7	48.8	37.7	1.7	1.4	1.1
			Heterooctamer ($(GltB-GltD)_4$)	30.1	25.4	19.6	0.2	0.2	0.1
P09832	GltD		Monomer	90.4	76.4	59.0	5.5	4.6	3.6
			Heterooctamer ($(GltB-GltD)_4$)	30.1	25.4	19.6	0.2	0.2	0.1
P28721	GltF	Cell membrane	Monomer	118.0	99.7	77.0	10.3	8.7	6.7
			Complex with SecB	71.2	58.2	46.5	3.0	2.4	2.0
			Complex with TF (Tig)	78.5	66.3	51.2	3.8	3.3	2.5
P37902	GltI	Periplasm	Monomer	107.5	90.8	70.2	8.3	7.0	5.4
			Complex with SecB	69.3	56.6	45.2	2.8	2.3	1.8
			Complex with TF (Tig)	75.8	64.0	49.4	3.5	3.0	2.3

UniProtKB A. N.	Protein name	Subcellular location	Subunit	D_0 $\left(\frac{\mu m^2}{s}\right)$			D_{cyto} $\left(\frac{\mu m^2}{s}\right)$		
				310 K	303 K	293 K	310 K	303 K	293 K
P0AER3	GltJ	Cell inner membrane	Monomer	116.0	98.0	75.7	9.9	8.4	6.5
			Complex with SecB	70.9	57.9	46.3	3.0	2.4	1.9
			Complex with TF (Tig)	78.0	65.9	50.9	3.8	3.2	2.5
P0AER5	GltK	Cell inner membrane	Monomer	120.6	101.9	78.7	10.8	9.2	7.1
			Complex with SecB	71.7	58.5	46.8	3.0	2.5	2.0
			Complex with TF (Tig)	79.1	66.8	51.6	3.9	3.3	2.6
P0AAG3	GltL	Cell inner membrane	Monomer	117.5	99.2	76.6	10.2	8.6	6.7
			Complex with SecB	71.2	58.1	46.4	3.0	2.4	1.9
			Complex with TF (Tig)	78.4	66.2	51.1	3.8	3.2	2.5
P21345	GltP	Cell inner membrane	Monomer	93.9	79.4	61.3	6.0	5.1	3.9
			Complex with SecB	65.9	53.9	43.0	2.4	2.0	1.6
			Complex with TF (Tig)	71.3	60.2	46.5	3.0	2.5	2.0
P0AER8	GltS	Cell inner membrane	Monomer	97.9	82.7	63.9	6.7	5.6	4.3
			Complex with SecB	67.0	54.8	43.7	2.5	2.1	1.7
			Complex with TF (Tig)	72.7	61.4	47.4	3.2	2.7	2.1
P04805	GltX	Cytoplasm	Monomer	89.2	75.3	58.2	5.3	4.5	3.5
P27305	GluQ		Monomer	105.7	89.3	69.0	8.0	6.8	5.2
P69789	GlvB	Cell membrane	Monomer	138.1	116.7	90.1	14.6	12.4	9.5
			Complex with SecB	74.0	60.4	48.3	3.3	2.7	2.2
			Complex with TF (Tig)	82.4	69.6	53.8	4.4	3.7	2.8
P31452	GlvC	Cell inner membrane	Monomer	100.5	84.9	65.6	7.1	6.0	4.6
			Complex with SecB	67.7	55.3	44.2	2.6	2.1	1.7
			Complex with TF (Tig)	73.6	62.2	48.0	3.3	2.8	2.1
P31450	GlvG		Monomer	123.0	103.9	80.2	11.3	9.6	7.4
P77364	GlxK		Monomer	101.5	85.7	66.2	7.3	6.1	4.7
P77161	GlxR		Monomer	111.0	93.8	72.4	9.0	7.6	5.8
P0A825	GlyA	Cytoplasm	Monomer	95.4	80.6	62.3	6.3	5.3	4.1
			Homotetramer	55.4	45.3	36.2	1.5	1.2	1.0
P00960	GlyQ	Cytoplasm	Monomer	105.8	89.4	69.1	8.0	6.8	5.2
P00961	GlyS	Cytoplasm	Monomer	77.6	65.5	50.6	3.7	3.2	2.4
P0AC88	Gmd		Monomer	98.2	83.0	64.1	6.7	5.7	4.4

UniProtKB A. N.	Protein name	Subcellular location	Subunit	D_0 $\left(\frac{\mu m^2}{s}\right)$ 310 K	303 K	293 K	D_{cyto} $\left(\frac{\mu m^2}{s}\right)$ 310 K	303 K	293 K
			Homodimer	74.9	61.2	48.9	3.4	2.8	2.2
P63224	GmhA	Cytoplasm	Monomer	129.4	109.3	84.5	12.7	10.7	8.3
			Homotetramer	75.2	61.4	49.0	3.4	2.8	2.2
P63228	GmhB	Cytoplasm	Monomer	128.3	108.4	83.7	12.4	10.5	8.1
P60546	Gmk	Cytoplasm	Monomer	123.2	104.1	80.4	11.4	9.6	7.4
			Homodimer	93.9	76.7	61.3	6.0	4.9	3.9
			Homotetramer	71.6	60.5	46.7	3.0	2.6	2.0
P77334	Gmr		Monomer	78.4	66.3	51.2	3.8	3.2	2.5
P00350	Gnd		Monomer	90.8	76.7	59.2	5.5	4.7	3.6
			Homodimer	69.2	56.5	45.1	2.8	2.3	1.8
P0AC92	GnsA		Monomer	203.3	171.8	132.7	32.5	27.5	21.2
P77695	GnsB		Monomer	203.7	172.1	132.9	32.6	27.5	21.3
P46859	GntK		Monomer	132.7	112.1	86.6	13.4	11.3	8.7
P0AC94	GntP	Cell inner membrane	Monomer	93.9	79.4	61.3	6.0	5.1	3.9
			Complex with SecB	65.9	53.9	43.0	2.4	2.0	1.6
			Complex with TF (Tig)	71.3	60.2	46.5	3.0	2.5	2.0
P0ACP5	GntR		Monomer	103.9	87.8	67.8	7.7	6.5	5.0
P39835	GntT	Cell inner membrane	Monomer	94.9	80.2	61.9	6.2	5.2	4.0
			Complex with SecB	66.2	54.1	43.2	2.5	2.0	1.6
			Complex with TF (Tig)	71.6	60.5	46.7	3.0	2.6	2.0
P0AC96	GntU	Cell inner membrane	Monomer	94.5	79.8	61.7	6.1	5.2	4.0
			Complex with SecB	66.1	54.0	43.1	2.4	2.0	1.6
			Complex with TF (Tig)	71.5	60.4	46.7	3.0	2.5	2.0
P46846	GntX		Monomer	119.1	100.6	77.7	10.5	8.9	6.9
P06715	Gor	Cytoplasm	Monomer	92.7	78.3	60.5	5.8	4.9	3.8
			Homodimer	70.6	57.7	46.1	2.9	2.4	1.9
P32662	Gph		Monomer	116.2	98.2	75.8	10.0	8.4	6.5
P62707	GpmA		Monomer	114.3	96.6	74.6	9.6	8.1	6.3
			Homodimer	87.1	71.2	56.9	5.0	4.1	3.3
P0A7A2	GpmB		Monomer	122.3	103.3	79.8	11.2	9.4	7.3
P37689	GpmI		Monomer	87.7	74.1	57.2	5.1	4.3	3.3

UniProtKB A. N.	Protein name	Subcellular location	Subunit	D_0 $\left(\frac{\mu m^2}{s}\right)$			D_{cyto} $\left(\frac{\mu m^2}{s}\right)$		
				310 K	303 K	293 K	310 K	303 K	293 K
P25552	GppA		Monomer	88.5	74.8	57.8	5.2	4.4	3.4
P0A6S7	GpsA	Cytoplasm	Monomer	104.0	87.9	67.9	7.7	6.5	5.0
P0A9M5	Gpt	Cell inner membrane	Monomer	140.2	118.5	91.5	15.1	12.8	9.9
			Complex with SecB	74.2	60.6	48.4	3.3	2.7	2.2
			Complex with TF (Tig)	82.7	69.9	54.0	4.4	3.7	2.9
P68066	GrcA		Monomer	150.0	126.7	97.9	17.5	14.8	11.4
P0A6W5	GreA		Monomer	138.1	116.7	90.1	14.6	12.4	9.5
P30128	GreB		Monomer	135.5	114.4	88.4	14.0	11.8	9.2
P0A6F5	GroL	Cytoplasm	Monomer	87.0	73.5	56.8	5.0	4.2	3.3
P0A6F9	GroS	Cytoplasm	Monomer	170.0	143.6	110.9	22.7	19.2	14.8
P09372	GrpE	Cytoplasm	Monomer	127.1	107.4	82.9	12.2	10.3	8.0
			Homodimer	96.9	79.1	63.2	6.5	5.3	4.2
P68688	GrxA		Monomer	174.7	147.6	114.0	24.0	20.3	15.7
P0AC59	GrxB		Monomer	121.7	102.8	79.4	11.1	9.3	7.2
P0AC62	GrxC		Monomer	178.7	151.0	116.6	25.1	21.2	16.4
P0AC69	GrxD	Cytoplasm	Monomer	156.2	132.0	101.9	19.0	16.1	12.4
			Homodimer	119.1	97.3	77.7	10.5	8.6	6.9
P0A6W9	GshA		Monomer	86.5	73.0	56.4	4.9	4.2	3.2
P04425	GshB		Monomer	104.9	88.6	68.5	7.9	6.6	5.1
			Homotetramer	60.9	49.8	39.8	2.0	1.6	1.3
P75796	GsiA	Cell inner membrane	Monomer	80.9	68.3	52.8	4.2	3.5	2.7
			Complex with SecB	61.6	50.3	40.2	2.0	1.6	1.3
			Complex with TF (Tig)	65.7	55.5	42.9	2.4	2.0	1.6
P75797	GsiB	Periplasm	Monomer	87.5	73.9	57.1	5.1	4.3	3.3
			Complex with SecB	64.0	52.3	41.8	2.2	1.8	1.5
			Complex with TF (Tig)	68.7	58.1	44.8	2.7	2.3	1.8
P75798	GsiC	Cell inner membrane	Monomer	106.7	90.1	69.6	8.2	6.9	5.3
			Complex with SecB	69.1	56.5	45.1	2.8	2.3	1.8
			Complex with TF (Tig)	75.5	63.8	49.3	3.5	2.9	2.3
P75799	GsiD	Cell inner membrane	Monomer	107.7	91.0	70.3	8.4	7.1	5.5
			Complex with SecB	69.3	56.6	45.2	2.8	2.3	1.8

UniProtKB A. N.	Protein name	Subcellular location	Subunit	D_0 $\left(\frac{\mu m^2}{s}\right)$			D_{cyto} $\left(\frac{\mu m^2}{s}\right)$		
				310 K	303 K	293 K	310 K	303 K	293 K
			Complex with TF (Tig)	75.8	64.1	49.5	3.5	3.0	2.3
P0AEW6	Gsk		Monomer	92.9	78.5	60.6	5.9	5.0	3.8
P0AES0	Gsp		Monomer	80.2	67.8	52.3	4.1	3.4	2.7
P45756	GspA		Monomer	88.2	74.6	57.6	5.2	4.4	3.4
P45757	GspC	Cell inner membrane	Monomer	112.1	94.7	73.1	9.2	7.7	6.0
			Complex with SecB	70.2	57.3	45.8	2.9	2.3	1.9
			Complex with TF (Tig)	77.0	65.1	50.3	3.7	3.1	2.4
P45758	GspD	Cell outer membrane	Monomer	80.1	67.7	52.3	4.1	3.4	2.6
			Complex with SecB	61.3	50.1	40.0	2.0	1.6	1.3
			Complex with TF (Tig)	65.4	55.2	42.7	2.4	2.0	1.5
P45759	GspE	Cytoplasm	Monomer	88.7	74.9	57.9	5.2	4.4	3.4
P45760	GspI		Monomer	151.6	128.1	98.9	17.9	15.1	11.7
P45761	GspJ		Monomer	126.1	106.6	82.3	12.0	10.1	7.8
P45762	GspK	Cell inner membrane	Monomer	102.6	86.7	67.0	7.4	6.3	4.9
			Complex with SecB	68.2	55.7	44.5	2.7	2.2	1.7
			Complex with TF (Tig)	74.3	62.7	48.5	3.3	2.8	2.2
P45763	GspL	Cell inner membrane	Monomer	96.1	81.1	62.7	6.4	5.4	4.2
			Complex with SecB	66.5	54.4	43.4	2.5	2.0	1.6
			Complex with TF (Tig)	72.1	60.9	47.0	3.1	2.6	2.0
P0A9D2	Gst	Cytoplasm	Monomer	124.7	105.4	81.4	11.7	9.9	7.6
			Homodimer	95.1	77.7	62.0	6.2	5.1	4.0
P04079	GuaA		Monomer	86.2	72.8	56.3	4.9	4.1	3.2
			Homodimer	65.7	53.7	42.9	2.4	2.0	1.6
P0ADG7	GuaB		Monomer	90.4	76.4	59.0	5.5	4.6	3.6
			Homotetramer	52.5	42.9	34.3	1.3	1.0	0.8
P60560	GuaC		Monomer	102.9	86.9	67.1	7.5	6.3	4.9
			Homotetramer	59.7	48.8	39.0	1.9	1.5	1.2
P76641	GuaD		Monomer	91.6	77.4	59.8	5.7	4.8	3.7
P0AES2	GudD		Monomer	92.4	78.1	60.3	5.8	4.9	3.8
Q46916	GudP	Cell inner membrane	Monomer	92.4	78.1	60.3	5.8	4.9	3.8
			Complex with SecB	65.5	53.5	42.7	2.4	2.0	1.6

UniProtKB A. N.	Protein name	Subcellular location	Subunit	D_0 $\left(\frac{\mu m^2}{s}\right)$			D_{cyto} $\left(\frac{\mu m^2}{s}\right)$		
				310 K	303 K	293 K	310 K	303 K	293 K
			Complex with TF (Tig)	70.7	59.7	46.1	2.9	2.5	1.9
Q46915	GudX		Monomer	92.6	78.3	60.5	5.8	4.9	3.8
P15081	GutM		Monomer	155.9	131.7	101.7	18.9	16.0	12.4
P17115	GutQ		Monomer	106.7	90.2	69.7	8.2	6.9	5.3
			Homotetramer	62.0	50.6	40.5	2.1	1.7	1.3
P0AES4	GyrA	Cytoplasm	Monomer	70.8	59.8	46.2	2.9	2.5	1.9
P0AES6	GyrB	Cytoplasm	Monomer	72.9	61.6	47.6	3.2	2.7	2.1
P0CI31	HcaB		Monomer	114.4	96.7	74.7	9.6	8.1	6.3
P0ABW0	HcaC		Monomer	164.3	138.8	107.2	21.1	17.9	13.8
P77650	HcaD		Monomer	96.5	81.6	63.0	6.4	5.4	4.2
P0ABR5	HcaE		Monomer	91.0	76.9	59.4	5.6	4.7	3.6
Q47140	HcaF		Monomer	130.0	109.8	84.8	12.8	10.8	8.4
Q47141	HcaR		Monomer	108.2	91.4	70.6	8.4	7.1	5.5
Q47142	HcaT	Cell inner membrane	Monomer	98.7	83.4	64.4	6.8	5.7	4.4
			Complex with SecB	67.2	54.9	43.9	2.6	2.1	1.7
			Complex with TF (Tig)	73.0	61.7	47.6	3.2	2.7	2.1
P31658	HchA	Cytoplasm	Monomer	110.5	93.3	72.1	8.9	7.5	5.8
			Homodimer	84.2	68.8	54.9	4.6	3.8	3.0
P75825	Hcp	Cytoplasm	Monomer	85.4	72.2	55.7	4.8	4.0	3.1
P75824	Hcr		Monomer	104.7	88.5	68.3	7.8	6.6	5.1
P69931	Hda		Monomer	117.5	99.3	76.7	10.2	8.6	6.7
P0AES9	HdeA	Periplasm	Monomer	161.4	136.3	105.3	20.4	17.2	13.3
			Complex with SecB	76.0	62.1	49.6	3.5	2.9	2.3
			Complex with TF (Tig)	85.4	72.2	55.8	4.8	4.0	3.1
P0AET2	HdeB		Monomer	160.4	135.5	104.7	20.1	17.0	13.1
P0AET5	HdeD	Cell inner membrane	Monomer	129.2	109.2	84.3	12.6	10.7	8.2
			Complex with SecB	72.9	59.6	47.6	3.2	2.6	2.1
			Complex with TF (Tig)	80.9	68.3	52.8	4.2	3.5	2.7
P0A8R9	HdfR		Monomer	109.7	92.7	71.6	8.7	7.4	5.7
P0AET8	HdhA		Monomer	117.3	99.1	76.5	10.2	8.6	6.6
			Homotetramer	68.1	55.6	44.4	2.7	2.2	1.7

UniProtKB A. N.	Protein name	Subcellular location	Subunit	D_0 $\left(\frac{\mu m^2}{s}\right)$			D_{cyto} $\left(\frac{\mu m^2}{s}\right)$		
				310 K	303 K	293 K	310 K	303 K	293 K
P15038	HelD		Monomer	77.1	65.2	50.3	3.7	3.1	2.4
P0A6X1	HemA		Monomer	94.6	79.9	61.7	6.1	5.2	4.0
			Homodimer	72.1	58.9	47.0	3.1	2.5	2.0
P0ACB2	HemB		Monomer	104.8	88.6	68.4	7.8	6.6	5.1
			Homooctamer	46.4	37.9	30.3	0.9	0.7	0.6
P06983	HemC		Monomer	107.0	90.4	69.8	8.2	6.9	5.4
P09126	HemD		Monomer	115.6	97.6	75.4	9.8	8.3	6.4
P29680	HemE	Cytoplasm	Monomer	100.9	85.3	65.9	7.2	6.1	4.7
			Homodimer	76.9	62.8	50.2	3.7	3.0	2.4
P36553	HemF	Cytoplasm	Monomer	106.4	89.9	69.4	8.1	6.9	5.3
			Homodimer	81.1	66.2	52.9	4.2	3.4	2.7
P0ACB4	HemG		Monomer	128.4	108.5	83.8	12.5	10.5	8.1
P23871	HemH	Cytoplasm	Monomer	104.5	88.3	68.2	7.8	6.6	5.1
P0ACC1	HemK		Monomer	110.8	93.6	72.3	8.9	7.5	5.8
P23893	HemL	Cytoplasm	Monomer	95.4	80.6	62.2	6.3	5.3	4.1
			Homodimer	72.7	59.4	47.4	3.2	2.6	2.1
P32131	HemN	Cytoplasm	Monomer	89.9	76.0	58.7	5.4	4.6	3.5
P09127	HemX	Cell inner membrane	Monomer	97.4	82.3	63.6	6.6	5.6	4.3
			Complex with SecB	66.9	54.7	43.7	2.5	2.1	1.6
			Complex with TF (Tig)	72.5	61.3	47.3	3.1	2.6	2.0
P0ACB7	HemY	Cell inner membrane	Monomer	95.5	80.7	62.3	6.3	5.3	4.1
			Complex with SecB	66.4	54.2	43.3	2.5	2.0	1.6
			Complex with TF (Tig)	71.8	60.7	46.9	3.1	2.6	2.0
P46118	HexR		Monomer	109.4	92.4	71.4	8.7	7.3	5.6
P0ABC3	HflC	Cell inner membrane	Monomer	102.6	86.7	66.9	7.4	6.3	4.9
			Complex with SecB	68.2	55.7	44.5	2.7	2.2	1.7
			Complex with TF (Tig)	74.3	62.7	48.5	3.3	2.8	2.2
P25746	HflD	Cytoplasm. Cell inner membrane	Monomer	124.6	105.2	81.3	11.6	9.8	7.6
P0ABC7	HflK	Cell inner membrane	Monomer	95.2	80.4	62.1	6.2	5.3	4.1
			Complex with SecB	66.3	54.2	43.3	2.5	2.0	1.6

UniProtKB A. N.	Protein name	Subcellular location	Subunit	D_0 $\left(\frac{\mu m^2}{s}\right)$			D_{cyto} $\left(\frac{\mu m^2}{s}\right)$		
				310 K	303 K	293 K	310 K	303 K	293 K
			Complex with TF (Tig)	71.8	60.6	46.8	3.0	2.6	2.0
P25519	HflX	Cytoplasm	Monomer	93.0	78.6	60.7	5.9	5.0	3.8
P0A6X3	Hfq		Monomer	165.2	139.6	107.8	21.4	18.1	14.0
			Homohexamer	81.9	66.9	53.4	4.3	3.5	2.8
P0ACE3	Hha		Monomer	182.8	154.4	119.3	26.3	22.2	17.2
P76106	HicA		Monomer	200.9	169.7	131.1	31.7	26.8	20.7
P67697	HicB		Monomer	146.2	123.5	95.4	16.5	14.0	10.8
P67701	HigA		Monomer	147.2	124.3	96.0	16.8	14.2	10.9
P64578	HigB		Monomer	160.1	135.2	104.5	20.0	16.9	13.1
P0ACE7	HinT		Monomer	154.5	130.6	100.8	18.6	15.7	12.1
P23874	HipA		Monomer	92.3	78.0	60.2	5.8	4.9	3.8
			Heterotetramer ($HipA_2-HipB_2$)	65.4	55.3	42.7	2.4	2.0	1.6
P23873	HipB		Monomer	172.4	145.7	112.5	23.4	19.7	15.2
			Homodimer	131.4	107.3	85.7	13.1	10.7	8.6
			Heterotetramer ($HipA_2-HipB_2$)	65.4	55.3	42.7	2.4	2.0	1.6
P10371	HisA	Cytoplasm	Monomer	118.6	100.2	77.4	10.4	8.8	6.8
P06987	HisB	Cytoplasm	Monomer	99.9	84.4	65.2	7.0	5.9	4.6
P06986	HisC		Monomer	100.8	85.2	65.8	7.1	6.0	4.7
			Homodimer	76.8	62.8	50.1	3.6	3.0	2.4
P06988	HisD		Monomer	94.8	80.1	61.8	6.2	5.2	4.0
			Homodimer	72.2	59.0	47.1	3.1	2.5	2.0
P60664	HisF	Cytoplasm	Monomer	114.5	96.7	74.7	9.6	8.1	6.3
			Heterodimer (HisH−HisF)	91.7	77.5	59.9	5.7	4.8	3.7
P60757	HisG	Cytoplasm	Monomer	107.6	90.9	70.2	8.3	7.0	5.4
P60595	HisH	Cytoplasm	Monomer	127.4	107.7	83.2	12.3	10.4	8.0
			Heterodimer (HisH−HisF)	91.7	77.5	59.9	5.7	4.8	3.7
P06989	HisI	Cytoplasm	Monomer	125.0	105.6	81.6	11.7	9.9	7.7
P0AEU0	HisJ	Periplasm	Monomer	114.5	96.7	74.7	9.6	8.1	6.3
			Complex with SecB	70.6	57.7	46.1	2.9	2.4	1.9
			Complex with TF (Tig)	77.6	65.6	50.7	3.7	3.2	2.4

UniProtKB A. N.	Protein name	Subcellular location	Subunit	D_0 $\left(\frac{\mu m^2}{s}\right)$			D_{cyto} $\left(\frac{\mu m^2}{s}\right)$		
				310 K	303 K	293 K	310 K	303 K	293 K
P60995	HisL		Monomer	319.2	269.7	208.3	75.1	63.4	49.0
P0AEU3	HisM	Cell inner membrane	Monomer	117.1	98.9	76.4	10.1	8.6	6.6
			Complex with SecB	71.1	58.1	46.4	3.0	2.4	1.9
			Complex with TF (Tig)	78.3	66.1	51.1	3.8	3.2	2.5
P07109	HisP	Cell inner membrane	Monomer	114.2	96.5	74.5	9.6	8.1	6.2
			Complex with SecB	70.6	57.7	46.1	2.9	2.4	1.9
			Complex with TF (Tig)	77.6	65.5	50.6	3.7	3.2	2.4
P52094	HisQ	Cell inner membrane	Monomer	121.1	102.3	79.0	10.9	9.2	7.1
			Complex with SecB	71.7	58.6	46.8	3.0	2.5	2.0
			Complex with TF (Tig)	79.2	66.9	51.7	3.9	3.3	2.6
P60906	HisS	Cytoplasm	Monomer	94.0	79.4	61.4	6.0	5.1	3.9
			Homodimer	71.7	58.5	46.8	3.0	2.5	2.0
P76341	HiuH	Periplasm	Monomer	145.4	122.9	94.9	16.4	13.8	10.7
			Complex with SecB	74.7	61.0	48.8	3.4	2.8	2.2
			Complex with TF (Tig)	83.5	70.6	54.5	4.5	3.8	2.9
P67910	HldD		Monomer	105.7	89.3	69.0	8.0	6.8	5.2
			Homopentamer	56.2	45.9	36.7	1.6	1.3	1.0
P76658	HldE		Monomer	91.1	76.9	59.4	5.6	4.7	3.6
			Homodimer	69.4	56.7	45.3	2.8	2.3	1.8
P77335	HlyE	Secreted. Periplasm. Host cell membrane	Monomer	107.1	90.5	69.9	8.2	7.0	5.4
			Complex with SecB	69.2	56.5	45.1	2.8	2.3	1.8
			Complex with TF (Tig)	75.6	63.9	49.4	3.5	3.0	2.3
P24232	Hmp	Cytoplasm	Monomer	96.6	81.6	63.1	6.5	5.5	4.2
P0AEV1	Hnr		Monomer	103.0	87.0	67.2	7.5	6.3	4.9
P0ACF8	Hns		Monomer	145.1	122.6	94.7	16.3	13.8	10.6
			Homodimer	110.6	90.4	72.2	8.9	7.3	5.8
P36645	HofB		Monomer	91.4	77.2	59.6	5.6	4.8	3.7
P36646	HofC	Cell inner membrane	Monomer	96.1	81.2	62.7	6.4	5.4	4.2
			Complex with SecB	66.6	54.4	43.4	2.5	2.0	1.6
			Complex with TF (Tig)	72.1	60.9	47.0	3.1	2.6	2.0

UniProtKB A. N.	Protein name	Subcellular location	Subunit	D_0 $\left(\frac{\mu m^2}{s}\right)$			D_{cyto} $\left(\frac{\mu m^2}{s}\right)$		
				310 K	303 K	293 K	310 K	303 K	293 K
P25960	HofD	Cell inner membrane	Monomer	120.5	101.8	78.7	10.8	9.1	7.1
			Complex with SecB	71.7	58.5	46.8	3.0	2.5	2.0
			Complex with TF (Tig)	79.1	66.8	51.6	3.9	3.3	2.6
P41441	HofF	Cell inner membrane	Monomer	96.2	81.3	62.8	6.4	5.4	4.2
			Complex with SecB	66.6	54.4	43.4	2.5	2.0	1.6
			Complex with TF (Tig)	72.1	60.9	47.1	3.1	2.6	2.0
P41442	HofG		Monomer	143.8	121.5	93.9	16.0	13.5	10.4
P41443	HofH		Monomer	135.4	114.4	88.3	14.0	11.8	9.1
P45753	HofM		Monomer	113.7	96.0	74.2	9.5	8.0	6.2
P64634	HofN	Cell membrane	Monomer	129.5	109.4	84.5	12.7	10.7	8.3
			Complex with SecB	72.9	59.6	47.6	3.2	2.6	2.1
			Complex with TF (Tig)	80.9	68.4	52.8	4.2	3.5	2.7
P45751	HofO	Cell membrane	Monomer	140.6	118.8	91.8	15.2	12.8	9.9
			Complex with SecB	74.2	60.6	48.4	3.3	2.7	2.2
			Complex with TF (Tig)	82.8	70.0	54.0	4.4	3.7	2.9
P45750	HofP		Monomer	146.8	124.0	95.8	16.7	14.1	10.9
P34749	HofQ	Cell outer membrane	Monomer	95.9	81.0	62.6	6.3	5.4	4.1
			Complex with SecB	66.5	54.3	43.4	2.5	2.0	1.6
			Complex with TF (Tig)	72.0	60.8	47.0	3.1	2.6	2.0
P37305	HokA	Membrane	Monomer	210.8	178.1	137.6	34.9	29.5	22.8
			Complex with SecB	78.3	63.9	51.1	3.8	3.1	2.5
			Complex with TF (Tig)	88.9	75.1	58.0	5.3	4.5	3.4
P77494	HokB	Membrane	Monomer	216.1	182.6	141.0	36.6	30.9	23.9
			Complex with SecB	78.4	64.1	51.2	3.8	3.1	2.5
			Complex with TF (Tig)	89.2	75.3	58.2	5.3	4.5	3.5
P0ACG4	HokC	Cell inner membrane	Monomer	218.0	184.2	142.3	37.2	31.5	24.3
			Complex with SecB	78.5	64.1	51.2	3.8	3.1	2.5
			Complex with TF (Tig)	89.3	75.4	58.3	5.3	4.5	3.5
P0ACG6	HokD	Cell membrane	Monomer	214.5	181.2	140.0	36.1	30.5	23.5
			Complex with SecB	78.4	64.0	51.1	3.8	3.1	2.5
			Complex with TF (Tig)	89.1	75.3	58.2	5.3	4.5	3.5

UniProtKB A. N.	Protein name	Subcellular location	Subunit	D_0 $\left(\frac{\mu m^2}{s}\right)$			D_{cyto} $\left(\frac{\mu m^2}{s}\right)$		
				310 K	303 K	293 K	310 K	303 K	293 K
P77091	HokE	Membrane	Monomer	217.1	183.4	141.7	36.9	31.2	24.1
			Complex with SecB	78.4	64.1	51.2	3.8	3.1	2.5
			Complex with TF (Tig)	89.2	75.4	58.2	5.3	4.5	3.5
P28630	HolA		Monomer	101.5	85.7	66.2	7.3	6.1	4.7
P28631	HolB		Monomer	103.4	87.3	67.5	7.6	6.4	4.9
P28905	HolC		Monomer	141.3	119.4	92.2	15.4	13.0	10.0
P28632	HolD		Monomer	146.5	123.8	95.6	16.6	14.0	10.8
P0ABS8	HolE		Monomer	181.0	152.9	118.1	25.8	21.8	16.8
P0A9M2	Hpt	Cytoplasm	Monomer	131.2	110.8	85.6	13.1	11.0	8.5
			Homotetramer	76.2	62.2	49.7	3.6	2.9	2.3
P43329	HrpA		Monomer	59.8	50.5	39.0	1.9	1.6	1.2
P37024	HrpB		Monomer	73.2	61.8	47.8	3.2	2.7	2.1
P54745	HrsA	Cell inner membrane	Monomer	80.6	68.1	52.6	4.1	3.5	2.7
			Complex with SecB	61.5	50.3	40.1	2.0	1.6	1.3
			Complex with TF (Tig)	65.6	55.4	42.8	2.4	2.0	1.6
P0A6Z1	HscA		Monomer	82.5	69.7	53.8	4.4	3.7	2.9
P0A6L9	HscB		Monomer	131.1	110.8	85.6	13.1	11.0	8.5
P77319	HscC		Monomer	84.4	71.3	55.1	4.6	3.9	3.0
P08957	HsdM		Monomer	85.9	72.5	56.0	4.8	4.1	3.2
P08956	HsdR		Monomer	62.4	52.7	40.7	2.1	1.8	1.4
P05719	HsdS		Monomer	90.8	76.7	59.3	5.5	4.7	3.6
P52644	HslJ		Monomer	146.5	123.8	95.6	16.6	14.0	10.8
P0A6Y5	HslO	Cytoplasm	Monomer	108.6	91.8	70.9	8.5	7.2	5.6
P0ACG8	HslR		Monomer	145.3	122.8	94.8	16.3	13.8	10.6
P0A6H5	HslU	Cytoplasm	Monomer	92.1	77.8	60.1	5.7	4.9	3.7
			Homohexamer	45.6	37.3	29.8	0.8	0.7	0.6
P0A7B8	HslV	Cytoplasm	Monomer	133.9	113.1	87.4	13.7	11.5	8.9
			Homohexamer	66.3	54.2	43.3	2.5	2.0	1.6
P0AB20	HspQ	Cytoplasm	Monomer	161.8	136.7	105.6	20.5	17.3	13.4
P31474	HsrA	Cell inner membrane	Monomer	90.8	76.7	59.2	5.5	4.7	3.6
			Complex with SecB	65.0	53.1	42.4	2.3	1.9	1.5

UniProtKB A. N.	Protein name	Subcellular location	Subunit	D_0 $\left(\frac{\mu m^2}{s}\right)$			D_{cyto} $\left(\frac{\mu m^2}{s}\right)$		
				310 K	303 K	293 K	310 K	303 K	293 K
			Complex with TF (Tig)	70.1	59.2	45.7	2.9	2.4	1.9
P28697	HtgA		Monomer	128.4	108.5	83.8	12.5	10.5	8.1
P0A6Z3	HtpG	Cytoplasm. Cell inner membrane	Monomer	79.8	67.4	52.1	4.0	3.4	2.6
			Homodimer	60.8	49.7	39.7	1.9	1.6	1.3
P23894	HtpX	Cell inner membrane	Monomer	109.5	92.5	71.4	8.7	7.3	5.7
			Complex with SecB	69.7	56.9	45.5	2.8	2.3	1.8
			Complex with TF (Tig)	76.3	64.5	49.8	3.6	3.0	2.3
P0ACV0	HtrB	Cell inner membrane	Monomer	105.1	88.8	68.6	7.9	6.7	5.1
			Complex with SecB	68.8	56.2	44.9	2.7	2.2	1.8
			Complex with TF (Tig)	75.0	63.4	49.0	3.4	2.9	2.2
P27375	HtrC		Monomer	128.7	108.7	84.0	12.5	10.6	8.2
P33129	HtrE	Cell outer membrane	Monomer	71.2	60.2	46.5	3.0	2.5	1.9
			Complex with SecB	57.5	47.0	37.5	1.7	1.4	1.1
			Complex with TF (Tig)	60.7	51.3	39.6	1.9	1.6	1.3
P25666	HtrL		Monomer	107.5	90.8	70.1	8.3	7.0	5.4
P0ACF0	HupA		Monomer	175.8	148.5	114.7	24.3	20.5	15.9
			Heterodimer (HupA−HupB)	134.8	113.9	88.0	13.9	11.7	9.1
P0ACF4	HupB		Monomer	178.1	150.4	116.2	24.9	21.1	16.3
			Heterodimer (HupA−HupB)	134.8	113.9	88.0	13.9	11.7	9.1
P69739	HyaA	Cell inner membrane	Monomer	99.5	84.1	64.9	6.9	5.9	4.5
			Complex with SecB	67.4	55.1	44.0	2.6	2.1	1.7
			Complex with TF (Tig)	73.3	61.9	47.8	3.2	2.7	2.1
P0ACD8	HyaB	Cell membrane	Monomer	82.2	69.5	53.6	4.3	3.7	2.8
			Complex with SecB	62.1	50.8	40.5	2.1	1.7	1.3
			Complex with TF (Tig)	66.4	56.1	43.3	2.5	2.1	1.6
P0AAM1	HyaC	Cell inner membrane	Monomer	115.9	97.9	75.6	9.9	8.4	6.5
			Complex with SecB	70.9	57.9	46.3	2.9	2.4	1.9
			Complex with TF (Tig)	78.0	65.9	50.9	3.8	3.2	2.5
P19930	HyaD		Monomer	127.7	107.9	83.3	12.3	10.4	8.0
P19931	HyaE		Monomer	147.6	124.7	96.3	16.9	14.3	11.0
P19932	HyaF		Monomer	110.1	93.0	71.9	8.8	7.4	5.7

UniProtKB A. N.	Protein name	Subcellular location	Subunit	D_0 $\left(\frac{\mu m^2}{s}\right)$			D_{cyto} $\left(\frac{\mu m^2}{s}\right)$		
				310 K	303 K	293 K	310 K	303 K	293 K
P0AAJ8	HybA	Periplasm	Monomer	104.4	88.2	68.1	7.8	6.6	5.1
			Complex with SecB	68.6	56.0	44.8	2.7	2.2	1.8
			Complex with TF (Tig)	74.8	63.2	48.8	3.4	2.9	2.2
P37180	HybB	Cell inner membrane	Monomer	96.9	81.8	63.2	6.5	5.5	4.2
			Complex with SecB	66.8	54.5	43.6	2.5	2.1	1.6
			Complex with TF (Tig)	72.3	61.1	47.2	3.1	2.6	2.0
P0ACE0	HybC	Cell membrane	Monomer	84.1	71.1	54.9	4.6	3.9	3.0
			Complex with SecB	62.8	51.3	41.0	2.1	1.7	1.4
			Complex with TF (Tig)	67.2	56.8	43.9	2.6	2.2	1.7
P37182	HybD		Monomer	137.8	116.4	89.9	14.5	12.3	9.5
P0AAN1	HybE		Monomer	137.1	115.8	89.5	14.4	12.2	9.4
P0A703	HybF		Monomer	157.1	132.7	102.5	19.3	16.3	12.6
P0AAM7	HybG		Monomer	181.3	153.2	118.3	25.9	21.9	16.9
P69741	HybO	Cell membrane	Monomer	100.5	84.9	65.6	7.1	6.0	4.6
			Complex with SecB	67.7	55.3	44.2	2.6	2.1	1.7
			Complex with TF (Tig)	73.6	62.2	48.0	3.3	2.8	2.1
P0AEV4	HycA		Monomer	138.1	116.7	90.1	14.6	12.4	9.5
P0AAK1	HycB		Monomer	126.9	107.2	82.8	12.1	10.3	7.9
P16429	HycC	Cell inner membrane	Monomer	83.3	70.4	54.4	4.5	3.8	2.9
			Complex with SecB	62.5	51.1	40.8	2.1	1.7	1.4
			Complex with TF (Tig)	66.9	56.5	43.6	2.5	2.1	1.6
P16430	HycD	Cell inner membrane	Monomer	108.0	91.2	70.5	8.4	7.1	5.5
			Complex with SecB	69.4	56.7	45.3	2.8	2.3	1.8
			Complex with TF (Tig)	75.9	64.1	49.5	3.5	3.0	2.3
P16431	HycE		Monomer	82.8	70.0	54.1	4.4	3.7	2.9
P16432	HycF		Monomer	130.7	110.4	85.3	13.0	10.9	8.5
P16433	HycG		Monomer	115.2	97.3	75.2	9.8	8.2	6.4
P0AEV7	HycH		Monomer	145.4	122.8	94.9	16.3	13.8	10.7
P0AEV9	HycI		Monomer	139.9	118.2	91.3	15.0	12.7	9.8
P0AAK4	HydN		Monomer	134.1	113.3	87.5	13.7	11.6	8.9
P23481	HyfA		Monomer	126.3	106.7	82.4	12.0	10.1	7.8

UniProtKB A. N.	Protein name	Subcellular location	Subunit	D_0 $\left(\frac{\mu m^2}{s}\right)$			D_{cyto} $\left(\frac{\mu m^2}{s}\right)$		
				310 K	303 K	293 K	310 K	303 K	293 K
P23482	HyfB	Cell inner membrane	Monomer	79.3	67.0	51.8	4.0	3.3	2.6
			Complex with SecB	61.0	49.9	39.8	2.0	1.6	1.3
			Complex with TF (Tig)	65.0	54.9	42.4	2.3	2.0	1.5
P77858	HyfC	Cell inner membrane	Monomer	106.3	89.8	69.4	8.1	6.8	5.3
			Complex with SecB	69.0	56.4	45.0	2.7	2.2	1.8
			Complex with TF (Tig)	75.4	63.7	49.2	3.5	2.9	2.3
P77416	HyfD	Cell inner membrane	Monomer	90.6	76.5	59.1	5.5	4.7	3.6
			Complex with SecB	64.9	53.1	42.4	2.3	1.9	1.5
			Complex with TF (Tig)	70.0	59.1	45.7	2.8	2.4	1.9
P0AEW1	HyfE	Cell inner membrane	Monomer	123.7	104.5	80.7	11.5	9.7	7.5
			Complex with SecB	72.1	58.9	47.1	3.1	2.5	2.0
			Complex with TF (Tig)	79.8	67.4	52.1	4.0	3.4	2.6
P77437	HyfF	Cell inner membrane	Monomer	87.3	73.8	57.0	5.0	4.3	3.3
			Complex with SecB	63.9	52.2	41.7	2.2	1.8	1.5
			Complex with TF (Tig)	68.6	58.0	44.8	2.7	2.3	1.8
P77329	HyfG		Monomer	83.6	70.7	54.6	4.5	3.8	3.0
P77423	HyfH		Monomer	131.1	110.7	85.5	13.0	11.0	8.5
P77668	HyfI		Monomer	115.1	97.2	75.1	9.7	8.2	6.3
P77453	HyfJ		Monomer	145.0	122.5	94.6	16.2	13.7	10.6
P71229	HyfR		Monomer	78.2	66.1	51.0	3.8	3.2	2.5
P30147	Hyi		Monomer	113.1	95.5	73.8	9.3	7.9	6.1
			Homodimer	86.2	70.4	56.2	4.9	4.0	3.2
P0A700	HypA		Monomer	154.9	130.8	101.1	18.7	15.8	12.2
P0AAN3	HypB		Monomer	109.9	92.9	71.7	8.8	7.4	5.7
P0AAM3	HypC		Monomer	174.4	147.3	113.8	23.9	20.2	15.6
P24192	HypD		Monomer	98.9	83.5	64.5	6.8	5.8	4.5
P24193	HypE		Monomer	105.5	89.1	68.8	7.9	6.7	5.2
P30131	HypF		Monomer	75.6	63.9	49.3	3.5	2.9	2.3
Q46806	HyuA		Monomer	91.1	76.9	59.4	5.6	4.7	3.6
			Homotetramer	52.9	43.2	34.5	1.3	1.1	0.9
P37595	IaaA	nie wiedomo co	Monomer	107.5	90.8	70.2	8.3	7.0	5.4

UniProtKB A. N.	Protein name	Subcellular location	Subunit	D_0 $\left(\frac{\mu m^2}{s}\right)$			D_{cyto} $\left(\frac{\mu m^2}{s}\right)$		
				310 K	303 K	293 K	310 K	303 K	293 K
			Complex with SecB	69.3	56.6	45.2	2.8	2.3	1.8
			Complex with TF (Tig)	75.8	64.0	49.4	3.5	3.0	2.3
P39377	IadA	Cytoplasm	Monomer	99.1	83.8	64.7	6.9	5.8	4.5
P10423	Iap		Monomer	102.3	86.4	66.8	7.4	6.3	4.8
P0C054	IbpA	Cytoplasm	Monomer	144.3	121.9	94.2	16.1	13.6	10.5
			Homooligomer	23.7	20.0	15.5	0.1	0.1	0.1
P0C058	IbpB	Cytoplasm	Monomer	143.2	120.9	93.4	15.8	13.4	10.3
			Homodimer	109.1	89.1	71.2	8.6	7.0	5.6
			Homooligomer	23.5	19.9	15.4	0.1	0.1	0.1
C1P607	IbsA		Monomer	310.6	262.4	202.7	71.5	60.4	46.7
C1P608	IbsB		Monomer	320.2	270.5	209.0	75.5	63.8	49.3
C1P615	IbsC		Monomer	311.8	263.4	203.5	72.0	60.9	47.0
C1P616	IbsD	Membrane	Monomer	315.1	266.2	205.6	73.4	62.0	47.9
			Complex with SecB	79.9	65.3	52.1	4.0	3.3	2.6
			Complex with TF (Tig)	91.6	77.3	59.7	5.7	4.8	3.7
C1P617	IbsE		Monomer	311.9	263.5	203.6	72.1	60.9	47.0
P0AEW4	Icc		Monomer	110.8	93.6	72.3	8.9	7.5	5.8
P08200	Icd		Monomer	95.0	80.3	62.0	6.2	5.2	4.0
			Homodimer	72.4	59.2	47.3	3.1	2.6	2.0
P0A8S1	IciA		Monomer	107.4	90.8	70.1	8.3	7.0	5.4
			Homodimer	81.9	66.9	53.4	4.3	3.5	2.8
P16528	IclR		Monomer	112.5	95.1	73.4	9.2	7.8	6.0
			Homodimer	85.8	70.1	56.0	4.8	3.9	3.1
			Homotetramer	65.4	55.2	42.6	2.4	2.0	1.5
Q46822	Idi	Cytoplasm	Monomer	130.2	110.0	85.0	12.8	10.9	8.4
			Homodimer	99.2	81.0	64.7	6.9	5.6	4.5
P39346	IdnD		Monomer	103.1	87.1	67.3	7.5	6.4	4.9
P39208	IdnK		Monomer	129.0	109.0	84.2	12.6	10.6	8.2
P0A9P9	IdnO	Cytoplasm	Monomer	115.9	97.9	75.7	9.9	8.4	6.5
P39343	IdnR		Monomer	102.7	86.8	67.0	7.5	6.3	4.9
P39344	IdnT	Cell inner membrane	Monomer	94.8	80.1	61.9	6.2	5.2	4.0

UniProtKB A. N.	Protein name	Subcellular location	Subunit	D_0 $\left(\frac{\mu m^2}{s}\right)$ 310 K	D_0 303 K	D_0 293 K	D_{cyto} $\left(\frac{\mu m^2}{s}\right)$ 310 K	D_{cyto} 303 K	D_{cyto} 293 K
			Complex with SecB	66.2	54.1	43.2	2.5	2.0	1.6
			Complex with TF (Tig)	71.6	60.5	46.7	3.0	2.6	2.0
P0A6X7	IhfA		Monomer	164.1	138.7	107.1	21.1	17.8	13.8
			Heterodimer (IhfA−IhfB)	126.6	107.0	82.6	12.1	10.2	7.9
P0A6Y1	IhfB		Monomer	168.3	142.2	109.8	22.2	18.8	14.5
			Heterodimer (IhfA−IhfB)	126.6	107.0	82.6	12.1	10.2	7.9
P00956	IleS	Cytoplasm	Monomer	68.8	58.1	44.9	2.7	2.3	1.8
P04968	IlvA		Monomer	87.7	74.1	57.2	5.1	4.3	3.3
			Homotetramer	50.9	41.6	33.2	1.2	1.0	0.8
P08142	IlvB		Monomer	85.2	72.0	55.6	4.7	4.0	3.1
P05793	IlvC		Monomer	89.0	75.2	58.1	5.3	4.5	3.4
P05791	IlvD		Monomer	82.6	69.8	53.9	4.4	3.7	2.9
			Homodimer	62.9	51.4	41.1	2.1	1.7	1.4
P0AB80	IlvE		Monomer	106.7	90.1	69.6	8.2	6.9	5.3
			Homohexamer	52.8	43.2	34.5	1.3	1.1	0.9
P00892	IlvG		Monomer	85.9	72.5	56.0	4.8	4.1	3.2
P00894	IlvH		Monomer	137.1	115.8	89.5	14.4	12.2	9.4
P00893	IlvI		Monomer	83.9	70.8	54.7	4.6	3.8	3.0
P62522	IlvL		Monomer	268.6	227.0	175.3	55.1	46.5	36.0
P0ADG1	IlvM		Monomer	174.6	147.5	113.9	24.0	20.2	15.6
P0ADF8	IlvN		Monomer	165.6	139.9	108.0	21.5	18.2	14.0
C1P619	IlvX		Monomer	330.1	278.9	215.4	79.6	67.3	52.0
P05827	IlvY	Cytoplasm	Monomer	107.8	91.1	70.3	8.4	7.1	5.5
P27294	InaA		Monomer	119.9	101.3	78.2	10.7	9.0	7.0
P69222	InfA	Cytoplasm	Monomer	186.0	157.2	121.4	27.2	23.0	17.8
P0A705	InfB	Cytoplasm	Monomer	70.7	59.7	46.1	2.9	2.5	1.9
P0A707	InfC	Cytoplasm	Monomer	130.0	109.9	84.9	12.8	10.8	8.4
P0CF07	InsA1		Monomer	173.4	146.5	113.2	23.6	20.0	15.4
P0CF08	InsA2		Monomer	173.2	146.3	113.0	23.6	19.9	15.4
P0CF09	InsA3		Monomer	173.2	146.3	113.0	23.6	19.9	15.4
P0CF10	InsA4		Monomer	173.2	146.3	113.0	23.6	19.9	15.4

UniProtKB A. N.	Protein name	Subcellular location	Subunit	$D_0 \left(\frac{\mu m^2}{s}\right)$			$D_{\text{cyto}} \left(\frac{\mu m^2}{s}\right)$		
				310 K	303 K	293 K	310 K	303 K	293 K
P0CF11	InsA5		Monomer	173.4	146.5	113.2	23.6	20.0	15.4
P0CF12	InsA6		Monomer	173.4	146.5	113.2	23.6	20.0	15.4
P19767	InsA7		Monomer	173.0	146.2	112.9	23.5	19.9	15.4
P0CF25	InsB1		Monomer	132.6	112.0	86.5	13.4	11.3	8.7
P0CF26	InsB2		Monomer	132.1	111.6	86.2	13.3	11.2	8.7
P0CF27	InsB3		Monomer	132.1	111.6	86.2	13.3	11.2	8.7
P57998	InsB4		Monomer	132.3	111.7	86.3	13.3	11.2	8.7
P0CF28	InsB5		Monomer	132.6	112.0	86.5	13.4	11.3	8.7
P0CF29	InsB6		Monomer	132.6	112.0	86.5	13.4	11.3	8.7
P0CF40	InsC1		Monomer	153.6	129.8	100.2	18.4	15.5	12.0
P0CF41	InsC2		Monomer	153.6	129.8	100.2	18.4	15.5	12.0
P0CF42	InsC3		Monomer	153.6	129.8	100.2	18.4	15.5	12.0
P0CF43	InsC4		Monomer	153.6	129.8	100.2	18.4	15.5	12.0
P0CF44	InsC5		Monomer	153.6	129.8	100.2	18.4	15.5	12.0
P0CF45	InsC6		Monomer	153.6	129.8	100.2	18.4	15.5	12.0
P0CF53	InsD1		Monomer	106.3	89.8	69.4	8.1	6.8	5.3
P0CF54	InsD2		Monomer	106.3	89.8	69.4	8.1	6.8	5.3
P0CF55	InsD3		Monomer	106.3	89.8	69.4	8.1	6.8	5.3
P0CF56	InsD4		Monomer	106.3	89.8	69.4	8.1	6.8	5.3
P0CF57	InsD5		Monomer	106.3	89.8	69.4	8.1	6.8	5.3
P0CF58	InsD6		Monomer	106.3	89.8	69.4	8.1	6.8	5.3
P0CF60	InsD8		Monomer	121.0	102.2	78.9	10.9	9.2	7.1
P0CF66	InsE1		Monomer	163.1	137.8	106.4	20.8	17.6	13.6
P0CF67	InsE2		Monomer	163.1	137.8	106.4	20.8	17.6	13.6
P0CF68	InsE3		Monomer	163.1	137.8	106.4	20.8	17.6	13.6
P0CF69	InsE4		Monomer	163.1	137.8	106.4	20.8	17.6	13.6
P0CF70	InsE5		Monomer	163.1	137.8	106.4	20.8	17.6	13.6
P0CF79	InsF1		Monomer	107.4	90.7	70.1	8.3	7.0	5.4
P0CF80	InsF2		Monomer	107.4	90.7	70.1	8.3	7.0	5.4
P0CF81	InsF3		Monomer	107.4	90.7	70.1	8.3	7.0	5.4
P0CF82	InsF4		Monomer	107.4	90.7	70.1	8.3	7.0	5.4

UniProtKB A. N.	Protein name	Subcellular location	Subunit	$D_0\left(\frac{\mu m^2}{s}\right)$			$D_{\text{cyto}}\left(\frac{\mu m^2}{s}\right)$		
				310 K	303 K	293 K	310 K	303 K	293 K
P0CF83	InsF5		Monomer	107.4	90.7	70.1	8.3	7.0	5.4
P03835	InsG		Monomer	91.5	77.3	59.7	5.7	4.8	3.7
P0CE49	InsH1		Monomer	100.9	85.2	65.8	7.2	6.0	4.7
P0CE57	InsH10		Monomer	100.9	85.2	65.8	7.2	6.0	4.7
P0CE58	InsH11		Monomer	100.9	85.2	65.8	7.2	6.0	4.7
P0CE50	InsH2		Monomer	100.9	85.2	65.8	7.2	6.0	4.7
P0CE51	InsH3		Monomer	100.9	85.2	65.8	7.2	6.0	4.7
P0CE52	InsH4		Monomer	100.9	85.2	65.8	7.2	6.0	4.7
P76071	InsH5		Monomer	102.5	86.6	66.9	7.4	6.3	4.8
P0CE53	InsH6		Monomer	101.0	85.3	65.9	7.2	6.1	4.7
P0CE54	InsH7		Monomer	100.9	85.2	65.8	7.2	6.0	4.7
P0CE55	InsH8		Monomer	100.9	85.2	65.8	7.2	6.0	4.7
P0CE56	InsH9		Monomer	100.9	85.2	65.8	7.2	6.0	4.7
P0CF88	InsI1		Monomer	96.3	81.3	62.8	6.4	5.4	4.2
P0CF89	InsI3		Monomer	96.3	81.3	62.8	6.4	5.4	4.2
P0CF90	InsI4		Monomer	96.3	81.3	62.8	6.4	5.4	4.2
P19768	InsJ		Monomer	132.2	111.7	86.3	13.3	11.2	8.7
P19769	InsK		Monomer	107.4	90.8	70.1	8.3	7.0	5.4
P0CF91	InsL1		Monomer	99.3	83.9	64.8	6.9	5.8	4.5
P0CF92	InsL2		Monomer	99.3	83.9	64.8	6.9	5.8	4.5
P0CF93	InsL3		Monomer	99.3	83.9	64.8	6.9	5.8	4.5
P75679	InsN1		Monomer	146.2	123.5	95.4	16.5	14.0	10.8
P39212	InsN2		Monomer	163.0	137.7	106.4	20.8	17.6	13.6
P75680	InsO1		Monomer	144.0	121.7	94.0	16.0	13.5	10.4
Q47718	InsO2		Monomer	126.1	106.5	82.3	12.0	10.1	7.8
P76102	InsQ		Monomer	97.1	82.0	63.3	6.5	5.5	4.3
P32053	IntA		Monomer	94.3	79.7	61.6	6.1	5.1	4.0
P39347	IntB		Monomer	95.2	80.4	62.1	6.2	5.3	4.1
P24218	IntD		Monomer	95.6	80.8	62.4	6.3	5.3	4.1
P75969	IntE		Monomer	97.6	82.4	63.7	6.6	5.6	4.3
P71298	IntF		Monomer	89.7	75.8	58.5	5.4	4.5	3.5

UniProtKB A. N.	Protein name	Subcellular location	Subunit	D_0 $\left(\frac{\mu m^2}{s}\right)$			D_{cyto} $\left(\frac{\mu m^2}{s}\right)$		
				310 K	303 K	293 K	310 K	303 K	293 K
P76323	IntG		Monomer	167.8	141.8	109.5	22.1	18.7	14.4
P76168	IntQ		Monomer	96.8	81.8	63.2	6.5	5.5	4.2
P76056	IntR		Monomer	93.6	79.1	61.1	6.0	5.1	3.9
P37326	IntS		Monomer	96.5	81.5	62.9	6.4	5.4	4.2
P76542	IntZ		Monomer	95.4	80.6	62.2	6.3	5.3	4.1
P39375	IraD	Cytoplasm	Monomer	148.1	125.2	96.7	17.0	14.4	11.1
P75987	IraM	Cytoplasm	Monomer	159.9	135.1	104.4	20.0	16.9	13.0
P0AAN9	IraP	Cytoplasm	Monomer	172.9	146.1	112.9	23.5	19.9	15.3
P0AAC8	IscA		Monomer	163.0	137.7	106.4	20.8	17.6	13.6
			Homodimer	124.2	101.5	81.1	11.6	9.5	7.6
			Homotetramer	94.7	80.0	61.8	6.1	5.2	4.0
P0AGK8	IscR		Monomer	139.0	117.5	90.7	14.8	12.5	9.7
P0A6B7	IscS	Cytoplasm	Monomer	95.6	80.8	62.4	6.3	5.3	4.1
			Homodimer	72.8	59.5	47.5	3.2	2.6	2.1
P0C0L9	IscX		Monomer	190.8	161.2	124.5	28.7	24.2	18.7
P22939	IspA	Cytoplasm	Monomer	109.1	92.2	71.2	8.6	7.3	5.6
P0AD57	IspB		Monomer	105.3	89.0	68.7	7.9	6.7	5.2
Q46893	IspD		Monomer	119.1	100.6	77.7	10.5	8.9	6.9
			Homodimer	90.8	74.1	59.2	5.5	4.5	3.6
P62615	IspE		Monomer	110.8	93.6	72.3	8.9	7.5	5.8
			Homodimer	84.5	69.0	55.1	4.6	3.8	3.0
P62617	IspF		Monomer	140.4	118.7	91.7	15.2	12.8	9.9
			Homotrimer	91.3	74.6	59.6	5.6	4.6	3.7
P62620	IspG		Monomer	99.5	84.1	64.9	6.9	5.9	4.5
P62623	IspH		Monomer	105.8	89.4	69.1	8.0	6.8	5.2
			Homodimer	80.7	65.9	52.6	4.1	3.4	2.7
P0A710	IspZ	Cell inner membrane	Monomer	129.5	109.4	84.5	12.7	10.7	8.3
			Complex with SecB	72.9	59.6	47.6	3.2	2.6	2.1
			Complex with TF (Tig)	80.9	68.4	52.8	4.2	3.5	2.7
P03061	IvbL		Monomer	269.5	227.6	175.8	55.4	46.8	36.2
P0AD59	Ivy	Periplasm	Monomer	140.5	118.7	91.7	15.2	12.8	9.9

UniProtKB A. N.	Protein name	Subcellular location	Subunit	D_0 $\left(\frac{\mu m^2}{s}\right)$			D_{cyto} $\left(\frac{\mu m^2}{s}\right)$		
				310 K	303 K	293 K	310 K	303 K	293 K
			Complex with SecB	74.2	60.6	48.4	3.3	2.7	2.2
			Complex with TF (Tig)	82.8	69.9	54.0	4.4	3.7	2.9
P21179	KatE	Cytoplasm	Monomer	74.8	63.2	48.8	3.4	2.9	2.2
			Homotetramer	43.5	35.5	28.4	0.7	0.6	0.5
P13029	KatG		Monomer	76.3	64.5	49.8	3.6	3.0	2.3
			Homotetramer	44.3	36.2	28.9	0.8	0.6	0.5
P0AB74	KbaY		Monomer	110.3	93.2	72.0	8.8	7.5	5.8
			Homotetramer	64.1	52.3	41.8	2.2	1.8	1.5
P0C8K0	KbaZ		Monomer	93.9	79.3	61.3	6.0	5.1	3.9
P0AB77	Kbl		Monomer	97.3	82.2	63.5	6.6	5.5	4.3
			Homodimer	74.1	60.6	48.4	3.3	2.7	2.2
P31069	Kch	Cell inner membrane	Monomer	94.8	80.1	61.9	6.2	5.2	4.0
			Complex with SecB	66.2	54.1	43.2	2.5	2.0	1.6
			Complex with TF (Tig)	71.6	60.5	46.7	3.0	2.6	2.0
P37647	KdgK		Monomer	106.8	90.2	69.7	8.2	6.9	5.3
P76268	KdgR		Monomer	112.1	94.7	73.2	9.2	7.7	6.0
P0A712	KdgT	Cell inner membrane	Monomer	107.2	90.6	69.9	8.3	7.0	5.4
			Complex with SecB	69.2	56.5	45.2	2.8	2.3	1.8
			Complex with TF (Tig)	75.7	63.9	49.4	3.5	3.0	2.3
P03959	KdpA	Cell inner membrane	Monomer	85.9	72.6	56.1	4.8	4.1	3.2
			Complex with SecB	63.4	51.8	41.4	2.2	1.8	1.4
			Complex with TF (Tig)	68.0	57.5	44.4	2.6	2.2	1.7
P03960	KdpB	Cell inner membrane	Monomer	79.5	67.2	51.9	4.0	3.4	2.6
			Complex with SecB	61.1	49.9	39.9	2.0	1.6	1.3
			Complex with TF (Tig)	65.0	55.0	42.4	2.3	2.0	1.5
P03961	KdpC	Cell inner membrane	Monomer	130.8	110.5	85.3	13.0	11.0	8.5
			Complex with SecB	73.1	59.7	47.7	3.2	2.6	2.1
			Complex with TF (Tig)	81.2	68.6	53.0	4.2	3.5	2.7
P21865	KdpD	Cell inner membrane	Monomer	70.3	59.4	45.9	2.9	2.4	1.9
			Complex with SecB	57.1	46.7	37.3	1.6	1.3	1.1
			Complex with TF (Tig)	60.2	50.8	39.3	1.9	1.6	1.2

UniProtKB A. N.	Protein name	Subcellular location	Subunit	D_0 $\left(\frac{\mu m^2}{s}\right)$			D_{cyto} $\left(\frac{\mu m^2}{s}\right)$		
				310 K	303 K	293 K	310 K	303 K	293 K
P21866	KdpE	Cytoplasm	Monomer	119.8	101.2	78.2	10.7	9.0	7.0
P36937	KdpF	Cell inner membrane	Monomer	274.0	231.5	178.8	57.1	48.3	37.3
			Complex with SecB	79.5	64.9	51.9	4.0	3.2	2.6
			Complex with TF (Tig)	90.9	76.8	59.3	5.6	4.7	3.6
P0A715	KdsA	Cytoplasm	Monomer	111.0	93.7	72.4	8.9	7.6	5.8
			Homotrimer	72.1	58.9	47.1	3.1	2.5	2.0
P04951	KdsB	Cytoplasm	Monomer	115.9	97.9	75.6	9.9	8.4	6.4
P0ABZ4	KdsC		Monomer	131.5	111.1	85.8	13.1	11.1	8.6
			Homotetramer	76.4	62.4	49.8	3.6	2.9	2.3
P45395	KdsD		Monomer	105.3	89.0	68.7	7.9	6.7	5.2
			Homotetramer	61.2	50.0	39.9	2.0	1.6	1.3
P37769	KduD		Monomer	116.8	98.6	76.2	10.1	8.5	6.6
Q46938	KduI		Monomer	110.6	93.4	72.2	8.9	7.5	5.8
			Homohexamer	54.8	44.8	35.8	1.5	1.2	0.9
P77338	KefA	Cell inner membrane	Monomer	63.7	53.8	41.5	2.2	1.9	1.4
			Complex with SecB	53.7	43.9	35.0	1.4	1.1	0.9
			Complex with TF (Tig)	56.1	47.4	36.6	1.6	1.3	1.0
P45522	KefB	Cell inner membrane	Monomer	82.1	69.4	53.6	4.3	3.6	2.8
			Complex with SecB	62.1	50.7	40.5	2.1	1.7	1.3
			Complex with TF (Tig)	66.3	56.0	43.3	2.5	2.1	1.6
P03819	KefC	Cell inner membrane	Monomer	81.5	68.8	53.2	4.2	3.6	2.8
			Complex with SecB	61.9	50.5	40.4	2.0	1.7	1.3
			Complex with TF (Tig)	66.0	55.8	43.1	2.4	2.1	1.6
P0A754	KefF		Monomer	131.0	110.7	85.5	13.0	11.0	8.5
P0A756	KefG		Monomer	129.1	109.1	84.2	12.6	10.7	8.2
P0AEX3	KgtP	Cell inner membrane	Monomer	94.0	79.4	61.3	6.0	5.1	3.9
			Complex with SecB	66.0	53.9	43.0	2.4	2.0	1.6
			Complex with TF (Tig)	71.3	60.2	46.5	3.0	2.5	2.0
P38393	Kil		Monomer	184.4	155.8	120.3	26.8	22.6	17.5
P39380	KptA		Monomer	130.1	109.9	84.9	12.8	10.8	8.4
P63183	Kup	Cell inner membrane	Monomer	80.8	68.2	52.7	4.1	3.5	2.7

UniProtKB A. N.	Protein name	Subcellular location	Subunit	D_0 $\left(\frac{\mu m^2}{s}\right)$			D_{cyto} $\left(\frac{\mu m^2}{s}\right)$		
				310 K	303 K	293 K	310 K	303 K	293 K
			Complex with SecB	61.6	50.3	40.2	2.0	1.6	1.3
			Complex with TF (Tig)	65.7	55.5	42.9	2.4	2.0	1.6
P07464	LacA	Cytoplasm	Monomer	124.9	105.5	81.5	11.7	9.9	7.6
			Homodimer	95.2	77.8	62.1	6.2	5.1	4.1
P03023	LacI		Monomer	101.6	85.8	66.3	7.3	6.2	4.8
			Homotetramer	59.0	48.2	38.5	1.8	1.5	1.2
P02920	LacY	Cell inner membrane	Monomer	94.4	79.8	61.6	6.1	5.2	4.0
			Complex with SecB	66.1	54.0	43.1	2.4	2.0	1.6
			Complex with TF (Tig)	71.5	60.4	46.6	3.0	2.5	2.0
P00722	LacZ		Monomer	65.9	55.7	43.0	2.4	2.0	1.6
			Homotetramer	38.3	31.3	25.0	0.5	0.4	0.3
P02943	LamB	Cell outer membrane	Monomer	91.9	77.6	59.9	5.7	4.8	3.7
			Complex with SecB	65.3	53.4	42.6	2.4	1.9	1.5
			Complex with TF (Tig)	70.5	59.5	46.0	2.9	2.5	1.9
P33229	Lar		Monomer	198.2	167.4	129.3	30.9	26.1	20.2
P37005	LasT		Monomer	120.0	101.4	78.3	10.7	9.0	7.0
P76008	LdcA	Cytoplasm	Monomer	107.3	90.7	70.0	8.3	7.0	5.4
P52095	LdcC		Monomer	76.1	64.3	49.7	3.6	3.0	2.3
			Homodecamer	30.9	25.2	20.1	0.2	0.2	0.2
P52643	LdhA		Monomer	103.8	87.7	67.7	7.7	6.5	5.0
Q6BF86	LdrA; ldrC		Monomer	246.7	208.4	161.0	47.1	39.8	30.7
Q6BF87	LdrB		Monomer	247.8	209.3	161.7	47.4	40.1	31.0
Q6BF25	LdrD		Monomer	249.1	210.5	162.6	47.9	40.5	31.3
P60785	LepA	Cell inner membrane	Monomer	82.1	69.3	53.5	4.3	3.6	2.8
P00803	LepB	Cell inner membrane	Monomer	104.5	88.2	68.2	7.8	6.6	5.1
			Complex with SecB	68.6	56.1	44.8	2.7	2.2	1.8
			Complex with TF (Tig)	74.9	63.2	48.8	3.4	2.9	2.2
P09151	LeuA		Monomer	87.0	73.5	56.8	5.0	4.2	3.3
			Homotetramer	50.5	41.3	33.0	1.1	0.9	0.7
P30125	LeuB	Cytoplasm	Monomer	100.7	85.0	65.7	7.1	6.0	4.6
			Homodimer	76.7	62.7	50.1	3.6	3.0	2.4

UniProtKB A. N.	Protein name	Subcellular location	Subunit	$D_0\left(\frac{\mu m^2}{s}\right)$			$D_{\text{cyto}}\left(\frac{\mu m^2}{s}\right)$		
				310 K	303 K	293 K	310 K	303 K	293 K
P0A6A6	LeuC		Monomer	91.9	77.6	60.0	5.7	4.8	3.7
			Heterodimer (LeuC−LeuD)	79.4	67.1	51.8	4.0	3.3	2.6
P30126	LeuD		Monomer	125.6	106.1	81.9	11.9	10.0	7.7
			Heterodimer (LeuC−LeuD)	79.4	67.1	51.8	4.0	3.3	2.6
P76249	LeuE	Cell membrane	Monomer	124.0	104.8	80.9	11.5	9.7	7.5
			Complex with SecB	72.2	59.0	47.1	3.1	2.5	2.0
			Complex with TF (Tig)	79.8	67.4	52.1	4.0	3.4	2.6
P0AD79	LeuL		Monomer	271.5	229.3	177.1	56.2	47.4	36.6
P10151	LeuO		Monomer	104.8	88.5	68.4	7.8	6.6	5.1
P07813	LeuS	Cytoplasm	Monomer	70.7	59.8	46.2	2.9	2.5	1.9
P0A7C2	LexA		Monomer	125.9	106.3	82.1	11.9	10.1	7.8
			Homodimer	95.9	78.3	62.6	6.3	5.2	4.1
P60955	Lgt	Cell inner membrane	Monomer	107.9	91.2	70.4	8.4	7.1	5.5
			Complex with SecB	69.4	56.7	45.3	2.8	2.3	1.8
			Complex with TF (Tig)	75.9	64.1	49.5	3.5	3.0	2.3
P37339	LhgO		Monomer	94.8	80.1	61.9	6.2	5.2	4.0
P30015	Lhr		Monomer	56.9	48.1	37.1	1.6	1.4	1.1
P15042	LigA		Monomer	78.9	66.6	51.5	3.9	3.3	2.5
P25772	LigB		Monomer	83.8	70.8	54.7	4.5	3.8	3.0
P37025	LigT		Monomer	131.6	111.2	85.9	13.2	11.1	8.6
P60716	LipA	Cytoplasm	Monomer	104.3	88.1	68.1	7.8	6.5	5.1
			Homodimer	79.5	65.0	51.9	4.0	3.2	2.6
P60720	LipB	Cytoplasm	Monomer	122.6	103.6	80.0	11.2	9.5	7.3
			Homotrimer	79.7	65.1	52.0	4.0	3.3	2.6
P11072	Lit	Cell membrane	Monomer	107.1	90.5	69.9	8.2	7.0	5.4
			Complex with SecB	69.2	56.5	45.1	2.8	2.3	1.8
			Complex with TF (Tig)	75.6	63.9	49.4	3.5	3.0	2.3
P22731	LivF		Monomer	118.1	99.7	77.0	10.3	8.7	6.7
P0A9S7	LivG		Monomer	114.5	96.8	74.7	9.6	8.1	6.3
P0AEX7	LivH	Cell inner membrane	Monomer	108.1	91.3	70.5	8.4	7.1	5.5
			Complex with SecB	69.4	56.7	45.3	2.8	2.3	1.8

UniProtKB A. N.	Protein name	Subcellular location	Subunit	D_0 $\left(\frac{\mu m^2}{s}\right)$ 310 K	303 K	293 K	D_{cyto} $\left(\frac{\mu m^2}{s}\right)$ 310 K	303 K	293 K
			Complex with TF (Tig)	75.9	64.1	49.5	3.5	3.0	2.3
P0AD96	LivJ	Periplasm	Monomer	101.1	85.4	66.0	7.2	6.1	4.7
			Complex with SecB	67.8	55.4	44.3	2.6	2.1	1.7
			Complex with TF (Tig)	73.8	62.3	48.2	3.3	2.8	2.1
P04816	LivK	Periplasm	Monomer	100.8	85.2	65.8	7.1	6.0	4.7
			Complex with SecB	67.8	55.4	44.2	2.6	2.1	1.7
			Complex with TF (Tig)	73.7	62.3	48.1	3.3	2.8	2.1
P22729	LivM	Cell inner membrane	Monomer	94.6	79.9	61.8	6.1	5.2	4.0
			Complex with SecB	66.1	54.0	43.2	2.5	2.0	1.6
			Complex with TF (Tig)	71.5	60.4	46.7	3.0	2.6	2.0
P33232	LldD		Monomer	97.6	82.5	63.7	6.6	5.6	4.3
P33231	LldP	Cell inner membrane	Monomer	85.9	72.6	56.1	4.8	4.1	3.2
			Complex with SecB	63.5	51.8	41.4	2.2	1.8	1.4
			Complex with TF (Tig)	68.0	57.5	44.4	2.6	2.2	1.7
P0ACL7	LldR		Monomer	113.4	95.8	74.0	9.4	7.9	6.1
P23930	Lnt	Cell inner membrane	Monomer	87.2	73.6	56.9	5.0	4.2	3.3
			Complex with SecB	63.9	52.2	41.7	2.2	1.8	1.5
			Complex with TF (Tig)	68.6	57.9	44.7	2.7	2.3	1.8
P61316	LolA	Periplasm	Monomer	125.5	106.1	81.9	11.9	10.0	7.7
			Complex with SecB	72.4	59.1	47.2	3.1	2.5	2.0
			Complex with TF (Tig)	80.1	67.7	52.3	4.1	3.4	2.6
P61320	LolB	Cell outer membrane	Monomer	123.3	104.2	80.5	11.4	9.6	7.4
			Complex with SecB	72.1	58.9	47.0	3.1	2.5	2.0
			Complex with TF (Tig)	79.7	67.3	52.0	4.0	3.4	2.6
P0ADC3	LolC	Cell inner membrane	Monomer	97.2	82.1	63.4	6.5	5.5	4.3
			Complex with SecB	66.8	54.6	43.6	2.5	2.1	1.6
			Complex with TF (Tig)	72.4	61.2	47.3	3.1	2.6	2.0
P75957	LolD	Cell inner membrane	Monomer	119.6	101.1	78.1	10.6	9.0	6.9
			Complex with SecB	71.5	58.4	46.7	3.0	2.5	2.0
			Complex with TF (Tig)	78.9	66.6	51.5	3.9	3.3	2.5
P75958	LolE	Cell inner membrane	Monomer	95.4	80.6	62.2	6.3	5.3	4.1

UniProtKB A. N.	Protein name	Subcellular location	Subunit	$D_0 \left(\frac{\mu m^2}{s}\right)$			$D_{\text{cyto}} \left(\frac{\mu m^2}{s}\right)$		
				310 K	303 K	293 K	310 K	303 K	293 K
			Complex with SecB	66.4	54.2	43.3	2.5	2.0	1.6
			Complex with TF (Tig)	71.8	60.7	46.9	3.1	2.6	2.0
P77184	LomR		Monomer	137.8	116.4	89.9	14.6	12.3	9.5
P0A9M0	Lon	Cytoplasm	Monomer	73.7	62.3	48.1	3.3	2.8	2.1
			Homohexamer	36.5	29.8	23.8	0.4	0.3	0.3
P0A9P0	LpdA	Cytoplasm. Cell inner membrane	Monomer	91.3	77.1	59.6	5.6	4.8	3.7
			Homodimer	69.6	56.8	45.4	2.8	2.3	1.8
P32099	LplA	Cytoplasm	Monomer	102.3	86.4	66.8	7.4	6.3	4.8
P39196	LplT	Cell inner membrane	Monomer	98.6	83.3	64.3	6.8	5.7	4.4
			Complex with SecB	67.2	54.9	43.9	2.6	2.1	1.7
			Complex with TF (Tig)	73.0	61.6	47.6	3.2	2.7	2.1
P45464	LpoA	Cell outer membrane	Monomer	79.2	66.9	51.7	3.9	3.3	2.6
			Complex with SecB	61.0	49.8	39.8	2.0	1.6	1.3
			Complex with TF (Tig)	64.9	54.8	42.4	2.3	2.0	1.5
P0AB38	LpoB	Cell outer membrane	Monomer	125.5	106.0	81.9	11.8	10.0	7.7
			Complex with SecB	72.4	59.1	47.2	3.1	2.5	2.0
			Complex with TF (Tig)	80.1	67.7	52.3	4.1	3.4	2.6
P69776	Lpp	Cell outer membrane	Monomer	185.4	156.6	121.0	27.1	22.9	17.7
			Complex with SecB	77.3	63.2	50.5	3.7	3.0	2.4
			Complex with TF (Tig)	87.5	73.9	57.1	5.1	4.3	3.3
P0ADV1	LptA	Periplasm	Monomer	131.1	110.8	85.6	13.1	11.0	8.5
			Complex with SecB	73.2	59.8	47.7	3.2	2.6	2.1
			Complex with TF (Tig)	81.2	68.6	53.0	4.2	3.5	2.7
P0A9V1	LptB	Cytoplasm. Cell inner membrane	Monomer	117.2	99.0	76.5	10.2	8.6	6.6
P0ADV9	LptC	Cell inner membrane	Monomer	127.3	107.6	83.1	12.2	10.3	8.0
			Complex with SecB	72.7	59.4	47.4	3.1	2.6	2.1
			Complex with TF (Tig)	80.5	68.0	52.5	4.1	3.5	2.7
P31554	LptD	Cell outer membrane	Monomer	73.0	61.7	47.6	3.2	2.7	2.1
			Complex with SecB	58.4	47.7	38.1	1.7	1.4	1.1
			Complex with TF (Tig)	61.7	52.1	40.3	2.0	1.7	1.3

UniProtKB A. N.	Protein name	Subcellular location	Subunit	D_0 $\left(\frac{\mu m^2}{s}\right)$			D_{cyto} $\left(\frac{\mu m^2}{s}\right)$		
				310 K	303 K	293 K	310 K	303 K	293 K
P0ADC1	LptE	Cell outer membrane	Monomer	128.1	108.2	83.6	12.4	10.5	8.1
			Complex with SecB	72.8	59.4	47.5	3.2	2.6	2.1
			Complex with TF (Tig)	80.7	68.1	52.6	4.1	3.5	2.7
P0AF98	LptF	Cell inner membrane	Monomer	99.8	84.3	65.2	7.0	5.9	4.6
			Complex with SecB	67.5	55.2	44.1	2.6	2.1	1.7
			Complex with TF (Tig)	73.4	62.0	47.9	3.2	2.7	2.1
P0ADC6	LptG	Cell inner membrane	Monomer	100.6	85.0	65.6	7.1	6.0	4.6
			Complex with SecB	67.7	55.3	44.2	2.6	2.1	1.7
			Complex with TF (Tig)	73.6	62.2	48.0	3.3	2.8	2.1
P0A722	LpxA	Cytoplasm	Monomer	115.1	97.2	75.1	9.7	8.2	6.4
			Homotrimer	74.8	61.1	48.8	3.4	2.8	2.2
P10441	LpxB		Monomer	97.9	82.7	63.9	6.7	5.6	4.4
P0A725	LpxC		Monomer	106.8	90.3	69.7	8.2	6.9	5.3
P21645	LpxD		Monomer	104.4	88.2	68.1	7.8	6.6	5.1
P43341	LpxH	Cytoplasm	Monomer	117.1	98.9	76.4	10.1	8.5	6.6
P27300	LpxK		Monomer	104.9	88.6	68.4	7.8	6.6	5.1
P0ACV2	LpxP	Cell inner membrane	Monomer	105.0	88.7	68.5	7.9	6.6	5.1
			Complex with SecB	68.7	56.2	44.9	2.7	2.2	1.8
			Complex with TF (Tig)	75.0	63.4	49.0	3.4	2.9	2.2
P36771	LrhA		Monomer	106.1	89.6	69.2	8.1	6.8	5.3
P0ACJ0	Lrp		Monomer	134.5	113.6	87.7	13.8	11.7	9.0
			Homodimer	102.5	83.7	66.9	7.4	6.1	4.8
P00804	LspA	Cell inner membrane	Monomer	136.6	115.4	89.1	14.3	12.1	9.3
			Complex with SecB	73.8	60.3	48.2	3.3	2.7	2.1
			Complex with TF (Tig)	82.2	69.4	53.6	4.3	3.7	2.8
P77257	LsrA	Cell inner membrane	Monomer	87.9	74.3	57.4	5.1	4.3	3.3
			Complex with SecB	64.1	52.4	41.8	2.3	1.8	1.5
			Complex with TF (Tig)	68.9	58.2	45.0	2.7	2.3	1.8
P76142	LsrB	Periplasm	Monomer	103.6	87.6	67.6	7.6	6.4	5.0
			Complex with SecB	68.4	55.9	44.7	2.7	2.2	1.8
			Complex with TF (Tig)	74.6	63.0	48.7	3.4	2.9	2.2

UniProtKB A. N.	Protein name	Subcellular location	Subunit	D_0 $\left(\frac{\mu m^2}{s}\right)$			D_{cyto} $\left(\frac{\mu m^2}{s}\right)$		
				310 K	303 K	293 K	310 K	303 K	293 K
P77672	LsrC	Cell inner membrane	Monomer	104.0	87.8	67.8	7.7	6.5	5.0
			Complex with SecB	68.5	56.0	44.7	2.7	2.2	1.8
			Complex with TF (Tig)	74.7	63.1	48.7	3.4	2.9	2.2
P0AFS1	LsrD	Cell inner membrane	Monomer	106.2	89.7	69.3	8.1	6.8	5.3
			Complex with SecB	69.0	56.4	45.0	2.7	2.2	1.8
			Complex with TF (Tig)	75.4	63.7	49.2	3.5	2.9	2.3
P76143	LsrF	Cytoplasm	Monomer	109.5	92.5	71.4	8.7	7.3	5.7
P64461	LsrG	Cytoplasm	Monomer	164.7	139.1	107.5	21.3	18.0	13.9
P77432	LsrK	Cytoplasm	Monomer	86.9	73.4	56.7	5.0	4.2	3.2
P76141	LsrR	Cytoplasm	Monomer	107.0	90.4	69.8	8.2	7.0	5.4
P75823	LtaE		Monomer	103.9	87.7	67.8	7.7	6.5	5.0
			Homotetramer	60.3	49.3	39.4	1.9	1.6	1.2
P45578	LuxS		Monomer	133.0	112.4	86.8	13.5	11.4	8.8
			Homodimer	101.4	82.8	66.1	7.2	5.9	4.7
P00861	LysA		Monomer	94.7	80.0	61.8	6.1	5.2	4.0
P08660	LysC		Monomer	92.9	78.5	60.6	5.9	5.0	3.8
			Homodimer	70.8	57.8	46.2	2.9	2.4	1.9
			Homotetramer	53.9	45.6	35.2	1.4	1.2	0.9
P25737	LysP	Cell inner membrane	Monomer	89.3	75.5	58.3	5.3	4.5	3.5
			Complex with SecB	64.6	52.7	42.1	2.3	1.9	1.5
			Complex with TF (Tig)	69.5	58.7	45.3	2.8	2.4	1.8
P03030	LysR	Cytoplasm	Monomer	106.3	89.8	69.4	8.1	6.8	5.3
P0A8N3	LysS	Cytoplasm	Monomer	86.8	73.4	56.7	5.0	4.2	3.2
			Homodimer	66.2	54.1	43.2	2.5	2.0	1.6
P0A8N5	LysU	Cytoplasm	Monomer	86.7	73.3	56.6	5.0	4.2	3.2
			Homodimer	66.1	54.0	43.1	2.4	2.0	1.6
P37677	Lyx		Monomer	88.3	74.6	57.6	5.2	4.4	3.4
			Homodimer	67.3	55.0	43.9	2.6	2.1	1.7
P77791	Maa		Monomer	131.2	110.9	85.6	13.1	11.0	8.5
			Homodimer	100.0	81.7	65.3	7.0	5.7	4.6
P75830	MacA	Cell inner membrane	Monomer	99.6	84.1	65.0	6.9	5.9	4.5

UniProtKB A. N.	Protein name	Subcellular location	Subunit	D_0 $\left(\frac{\mu m^2}{s}\right)$			D_{cyto} $\left(\frac{\mu m^2}{s}\right)$		
				310 K	303 K	293 K	310 K	303 K	293 K
			Complex with SecB	67.5	55.1	44.0	2.6	2.1	1.7
			Complex with TF (Tig)	73.3	61.9	47.8	3.2	2.7	2.1
P75831	MacB	Cell inner membrane	Monomer	80.1	67.7	52.3	4.1	3.4	2.6
			Complex with SecB	61.3	50.1	40.0	2.0	1.6	1.3
			Complex with TF (Tig)	65.4	55.2	42.7	2.4	2.0	1.5
P26616	MaeA		Monomer	83.7	70.7	54.6	4.5	3.8	3.0
			Homotetramer	48.6	39.7	31.7	1.0	0.8	0.7
P76558	MaeB		Monomer	75.5	63.8	49.2	3.5	2.9	2.3
			Homooligomer	33.4	28.2	21.8	0.3	0.3	0.2
P23917	Mak		Monomer	108.7	91.8	70.9	8.5	7.2	5.6
P0AEX9	MalE	Periplasm	Monomer	97.0	82.0	63.3	6.5	5.5	4.3
			Complex with SecB	66.8	54.6	43.6	2.5	2.1	1.6
			Complex with TF (Tig)	72.4	61.2	47.3	3.1	2.6	2.0
P02916	MalF	Cell inner membrane	Monomer	87.2	73.7	56.9	5.0	4.2	3.3
			Complex with SecB	63.9	52.2	41.7	2.2	1.8	1.5
			Complex with TF (Tig)	68.6	57.9	44.8	2.7	2.3	1.8
P68183	MalG	Cell inner membrane	Monomer	109.0	92.1	71.2	8.6	7.3	5.6
			Complex with SecB	69.6	56.9	45.4	2.8	2.3	1.8
			Complex with TF (Tig)	76.2	64.4	49.7	3.6	3.0	2.3
P18811	MalI		Monomer	103.7	87.6	67.7	7.6	6.5	5.0
P68187	MalK	Cell inner membrane	Monomer	99.2	83.8	64.8	6.9	5.8	4.5
			Complex with SecB	67.4	55.0	44.0	2.6	2.1	1.7
			Complex with TF (Tig)	73.2	61.8	47.7	3.2	2.7	2.1
P03841	MalM	Periplasm	Monomer	109.4	92.4	71.4	8.7	7.3	5.7
			Complex with SecB	69.7	56.9	45.5	2.8	2.3	1.8
			Complex with TF (Tig)	76.3	64.5	49.8	3.6	3.0	2.3
P00490	MalP		Monomer	72.7	61.5	47.5	3.2	2.7	2.1
			Homodimer	55.4	45.3	36.2	1.5	1.2	1.0
P15977	MalQ	Cytoplasm	Monomer	76.9	65.0	50.2	3.7	3.1	2.4
P25718	MalS	Periplasm	Monomer	78.0	65.9	50.9	3.8	3.2	2.5
			Complex with SecB	60.5	49.4	39.5	1.9	1.6	1.3

UniProtKB A. N.	Protein name	Subcellular location	Subunit	D_0 $\left(\frac{\mu m^2}{s}\right)$			D_{cyto} $\left(\frac{\mu m^2}{s}\right)$		
				310 K	303 K	293 K	310 K	303 K	293 K
			Complex with TF (Tig)	64.3	54.3	42.0	2.3	1.9	1.5
P06993	MalT		Monomer	69.1	58.4	45.1	2.8	2.3	1.8
P19642	MalX	Cell inner membrane	Monomer	87.4	73.9	57.1	5.1	4.3	3.3
			Complex with SecB	64.0	52.2	41.7	2.2	1.8	1.5
			Complex with TF (Tig)	68.7	58.0	44.8	2.7	2.3	1.8
P23256	MalY		Monomer	96.8	81.8	63.2	6.5	5.5	4.2
			Homodimer	73.8	60.3	48.2	3.3	2.7	2.1
P21517	MalZ	Cytoplasm	Monomer	80.8	68.3	52.7	4.1	3.5	2.7
P00946	ManA	Cytoplasm	Monomer	97.5	82.4	63.6	6.6	5.6	4.3
P24175	ManB		Monomer	91.5	77.3	59.7	5.6	4.8	3.7
P24174	ManC		Monomer	89.7	75.8	58.5	5.4	4.6	3.5
P69797	ManX	Cytoplasm. Cell inner membrane	Monomer	105.5	89.1	68.9	8.0	6.7	5.2
P69801	ManY	Cell inner membrane	Monomer	115.8	97.8	75.6	9.9	8.3	6.4
			Complex with SecB	70.9	57.9	46.2	2.9	2.4	1.9
			Complex with TF (Tig)	78.0	65.9	50.9	3.8	3.2	2.5
P69805	ManZ	Cell inner membrane	Monomer	110.3	93.2	72.0	8.8	7.5	5.8
			Complex with SecB	69.8	57.1	45.6	2.8	2.3	1.9
			Complex with TF (Tig)	76.5	64.7	49.9	3.6	3.0	2.4
P77455	MaoC		Monomer	79.1	66.9	51.6	3.9	3.3	2.6
P0AE18	Map		Monomer	113.1	95.6	73.8	9.4	7.9	6.1
P0ACH5	MarA		Monomer	146.5	123.7	95.6	16.6	14.0	10.8
P31121	MarB		Monomer	192.7	162.8	125.8	29.2	24.7	19.1
P0AEY1	MarC	Cell inner membrane	Monomer	123.2	104.1	80.4	11.4	9.6	7.4
			Complex with SecB	72.1	58.9	47.0	3.1	2.5	2.0
			Complex with TF (Tig)	79.7	67.3	52.0	4.0	3.4	2.6
P27245	MarR		Monomer	143.3	121.0	93.5	15.8	13.4	10.3
P71301	MatA	Cytoplasm	Monomer	123.9	104.7	80.8	11.5	9.7	7.5
P0AAA3	MatB		Monomer	131.2	110.8	85.6	13.1	11.0	8.5
P77188	MatC		Monomer	121.4	102.5	79.2	11.0	9.3	7.2
P0AE72	MazE		Monomer	177.1	149.6	115.6	24.7	20.8	16.1

UniProtKB A. N.	Protein name	Subcellular location	Subunit	D_0 $\left(\frac{\mu m^2}{s}\right)$			D_{cyto} $\left(\frac{\mu m^2}{s}\right)$		
				310 K	303 K	293 K	310 K	303 K	293 K
			Heterohexamer ($MazF_2-MazE_2-MazF_2$)	81.8	69.1	53.4	4.3	3.6	2.8
P0AE70	MazF		Monomer	160.1	135.3	104.5	20.0	16.9	13.1
			Homodimer	122.0	99.7	79.6	11.1	9.1	7.3
			Heterohexamer ($MazF_2-MazE_2-MazF_2$)	81.8	69.1	53.4	4.3	3.6	2.8
P0AEY3	MazG		Monomer	111.6	94.2	72.8	9.1	7.7	5.9
			Homodimer	85.0	69.4	55.5	4.7	3.8	3.1
Q47154	MbhA		Monomer	122.8	103.8	80.2	11.3	9.5	7.4
P0AAX6	McbA	Periplasm	Monomer	180.6	152.6	117.9	25.7	21.7	16.8
			Complex with SecB	77.1	63.0	50.3	3.7	3.0	2.4
			Complex with TF (Tig)	87.1	73.6	56.9	5.0	4.2	3.3
P76114	McbR		Monomer	120.2	101.5	78.4	10.7	9.1	7.0
P24200	McrA		Monomer	110.2	93.1	71.9	8.8	7.4	5.7
P15005	McrB		Monomer	89.6	75.7	58.5	5.4	4.5	3.5
P15006	McrC		Monomer	99.6	84.2	65.0	6.9	5.9	4.5
P0AEY5	MdaB		Monomer	126.9	107.2	82.8	12.1	10.3	7.9
P0AEY8	MdfA	Cell inner membrane	Monomer	96.2	81.3	62.8	6.4	5.4	4.2
			Complex with SecB	66.6	54.4	43.5	2.5	2.0	1.6
			Complex with TF (Tig)	72.1	60.9	47.1	3.1	2.6	2.0
P61889	Mdh		Monomer	108.9	92.0	71.1	8.6	7.2	5.6
			Homodimer	83.0	67.8	54.2	4.4	3.6	2.9
P77265	MdlA	Cell inner membrane	Monomer	82.3	69.5	53.7	4.3	3.7	2.8
			Complex with SecB	62.2	50.8	40.6	2.1	1.7	1.4
			Complex with TF (Tig)	66.4	56.1	43.3	2.5	2.1	1.6
P0AAG5	MdlB	Cell inner membrane	Monomer	82.7	69.9	54.0	4.4	3.7	2.9
			Complex with SecB	62.3	50.9	40.7	2.1	1.7	1.4
			Complex with TF (Tig)	66.6	56.3	43.5	2.5	2.1	1.6
P39401	MdoB	Cell inner membrane	Monomer	74.4	62.8	48.5	3.3	2.8	2.2
			Complex with SecB	59.0	48.2	38.5	1.8	1.5	1.2
			Complex with TF (Tig)	62.4	52.7	40.7	2.1	1.8	1.4
P75920	MdoC	Cell membrane	Monomer	95.9	81.0	62.6	6.3	5.4	4.1
			Complex with SecB	66.5	54.3	43.4	2.5	2.0	1.6

UniProtKB A. N.	Protein name	Subcellular location	Subunit	D_0 $\left(\frac{\mu m^2}{s}\right)$			D_{cyto} $\left(\frac{\mu m^2}{s}\right)$		
				310 K	303 K	293 K	310 K	303 K	293 K
			Complex with TF (Tig)	72.0	60.8	47.0	3.1	2.6	2.0
P40120	MdoD	Periplasm	Monomer	84.0	70.9	54.8	4.6	3.9	3.0
			Complex with SecB	62.8	51.3	41.0	2.1	1.7	1.4
			Complex with TF (Tig)	67.2	56.7	43.8	2.6	2.2	1.7
P33136	MdoG	Periplasm	Monomer	86.7	73.2	56.6	4.9	4.2	3.2
			Complex with SecB	63.7	52.0	41.6	2.2	1.8	1.4
			Complex with TF (Tig)	68.4	57.7	44.6	2.7	2.3	1.7
P62517	MdoH	Cell inner membrane	Monomer	70.8	59.8	46.2	2.9	2.5	1.9
			Complex with SecB	57.3	46.8	37.4	1.7	1.3	1.1
			Complex with TF (Tig)	60.5	51.1	39.4	1.9	1.6	1.2
P76397	MdtA	Cell inner membrane	Monomer	96.1	81.2	62.7	6.4	5.4	4.2
			Complex with SecB	66.6	54.4	43.4	2.5	2.0	1.6
			Complex with TF (Tig)	72.1	60.9	47.0	3.1	2.6	2.0
P76398	MdtB	Cell inner membrane	Monomer	66.9	56.5	43.7	2.5	2.1	1.6
			Complex with SecB	55.4	45.3	36.2	1.5	1.2	1.0
			Complex with TF (Tig)	58.1	49.1	37.9	1.7	1.5	1.1
P76399	MdtC	Cell inner membrane	Monomer	67.2	56.7	43.8	2.6	2.2	1.7
			Complex with SecB	55.5	45.4	36.2	1.5	1.2	1.0
			Complex with TF (Tig)	58.3	49.3	38.0	1.7	1.5	1.1
P36554	MdtD	Cell inner membrane	Monomer	91.2	77.0	59.5	5.6	4.7	3.7
			Complex with SecB	65.1	53.2	42.5	2.4	1.9	1.5
			Complex with TF (Tig)	70.2	59.3	45.8	2.9	2.4	1.9
P37636	MdtE	Cell inner membrane	Monomer	99.0	83.7	64.6	6.8	5.8	4.5
			Complex with SecB	67.3	55.0	43.9	2.6	2.1	1.7
			Complex with TF (Tig)	73.1	61.8	47.7	3.2	2.7	2.1
P37637	MdtF	Cell inner membrane	Monomer	67.0	56.6	43.7	2.5	2.1	1.7
			Complex with SecB	55.5	45.3	36.2	1.5	1.2	1.0
			Complex with TF (Tig)	58.2	49.2	38.0	1.7	1.5	1.1
P25744	MdtG	Cell inner membrane	Monomer	96.6	81.6	63.1	6.5	5.5	4.2
			Complex with SecB	66.7	54.5	43.5	2.5	2.0	1.6
			Complex with TF (Tig)	72.3	61.0	47.2	3.1	2.6	2.0

UniProtKB A. N.	Protein name	Subcellular location	Subunit	D_0 $\left(\frac{\mu m^2}{s}\right)$			D_{cyto} $\left(\frac{\mu m^2}{s}\right)$		
				310 K	303 K	293 K	310 K	303 K	293 K
P69367	MdtH	Cell inner membrane	Monomer	96.2	81.3	62.8	6.4	5.4	4.2
			Complex with SecB	66.6	54.4	43.4	2.5	2.0	1.6
			Complex with TF (Tig)	72.1	60.9	47.1	3.1	2.6	2.0
P69210	MdtI	Cell inner membrane	Monomer	162.1	137.0	105.8	20.6	17.4	13.4
			Complex with SecB	76.0	62.1	49.6	3.5	2.9	2.3
			Complex with TF (Tig)	85.5	72.2	55.8	4.8	4.0	3.1
P69212	MdtJ	Cell inner membrane	Monomer	155.1	131.0	101.2	18.8	15.8	12.2
			Complex with SecB	75.5	61.7	49.3	3.5	2.8	2.3
			Complex with TF (Tig)	84.7	71.6	55.3	4.7	3.9	3.1
P37340	MdtK	Cell inner membrane	Monomer	92.2	77.9	60.2	5.8	4.9	3.8
			Complex with SecB	65.4	53.5	42.7	2.4	1.9	1.6
			Complex with TF (Tig)	70.6	59.7	46.1	2.9	2.5	1.9
P31462	MdtL	Cell inner membrane	Monomer	98.8	83.4	64.4	6.8	5.7	4.4
			Complex with SecB	67.2	54.9	43.9	2.6	2.1	1.7
			Complex with TF (Tig)	73.0	61.7	47.6	3.2	2.7	2.1
P39386	MdtM	Cell inner membrane	Monomer	95.9	81.0	62.6	6.3	5.4	4.1
			Complex with SecB	66.5	54.3	43.4	2.5	2.0	1.6
			Complex with TF (Tig)	72.0	60.8	47.0	3.1	2.6	2.0
P32716	MdtN	Cell inner membrane	Monomer	103.4	87.4	67.5	7.6	6.4	5.0
			Complex with SecB	68.4	55.9	44.6	2.7	2.2	1.7
			Complex with TF (Tig)	74.5	63.0	48.6	3.4	2.8	2.2
P32715	MdtO	Cell inner membrane	Monomer	77.8	65.8	50.8	3.8	3.2	2.5
			Complex with SecB	60.4	49.4	39.4	1.9	1.6	1.2
			Complex with TF (Tig)	64.2	54.3	41.9	2.3	1.9	1.5
P32714	MdtP	Cell outer membrane	Monomer	89.4	75.5	58.4	5.3	4.5	3.5
			Complex with SecB	64.6	52.8	42.2	2.3	1.9	1.5
			Complex with TF (Tig)	69.5	58.7	45.4	2.8	2.4	1.8
P33369	MdtQ	Cell outer membrane	Monomer	90.3	76.3	58.9	5.5	4.6	3.6
			Complex with SecB	64.9	53.0	42.3	2.3	1.9	1.5
			Complex with TF (Tig)	69.9	59.0	45.6	2.8	2.4	1.9
P06720	MelA		Monomer	91.3	77.2	59.6	5.6	4.8	3.7

UniProtKB A. N.	Protein name	Subcellular location	Subunit	D_0 $\left(\frac{\mu m^2}{s}\right)$ 310 K	D_0 303 K	D_0 293 K	D_{cyto} $\left(\frac{\mu m^2}{s}\right)$ 310 K	D_{cyto} 303 K	D_{cyto} 293 K
			Homodimer	69.6	56.9	45.4	2.8	2.3	1.8
P02921	MelB	Cell inner membrane	Monomer	90.3	76.2	58.9	5.5	4.6	3.6
			Complex with SecB	64.9	53.0	42.3	2.3	1.9	1.5
			Complex with TF (Tig)	69.8	59.0	45.6	2.8	2.4	1.9
P0ACH8	MelR		Monomer	105.7	89.3	68.9	8.0	6.7	5.2
P32166	MenA	Cell inner membrane	Monomer	107.3	90.6	70.0	8.3	7.0	5.4
			Complex with SecB	69.2	56.6	45.2	2.8	2.3	1.8
			Complex with TF (Tig)	75.7	63.9	49.4	3.5	3.0	2.3
P0ABU0	MenB		Monomer	109.8	92.8	71.7	8.7	7.4	5.7
			Homohexamer	54.4	44.5	35.5	1.4	1.2	0.9
P29208	MenC		Monomer	105.0	88.7	68.5	7.9	6.6	5.1
P17109	MenD		Monomer	84.7	71.6	55.3	4.7	3.9	3.0
			Homodimer	64.6	52.7	42.1	2.3	1.9	1.5
P37353	MenE		Monomer	91.7	77.4	59.8	5.7	4.8	3.7
P38051	MenF		Monomer	92.7	78.3	60.5	5.8	4.9	3.8
P37355	MenH		Monomer	115.7	97.8	75.5	9.9	8.3	6.4
P0C0T5	MepA	Periplasm	Monomer	112.0	94.6	73.1	9.1	7.7	6.0
			Complex with SecB	70.2	57.3	45.8	2.9	2.3	1.9
			Complex with TF (Tig)	77.0	65.0	50.2	3.7	3.1	2.4
P07623	MetA	Cytoplasm	Monomer	104.7	88.5	68.3	7.8	6.6	5.1
			Homodimer	79.8	65.2	52.1	4.0	3.3	2.6
P00935	MetB	Cytoplasm	Monomer	98.7	83.4	64.4	6.8	5.7	4.4
			Homotetramer	57.3	46.8	37.4	1.6	1.3	1.1
P06721	MetC	Cytoplasm	Monomer	97.2	82.1	63.4	6.5	5.5	4.3
			Homotetramer	56.5	46.1	36.8	1.6	1.3	1.0
P25665	MetE		Monomer	74.7	63.1	48.7	3.4	2.9	2.2
P0AEZ1	MetF		Monomer	107.9	91.2	70.4	8.4	7.1	5.5
			Homotetramer	62.7	51.2	40.9	2.1	1.7	1.4
P00959	MetG	Cytoplasm	Monomer	77.8	65.7	50.8	3.8	3.2	2.5
			Homodimer	59.3	48.4	38.7	1.8	1.5	1.2
P13009	MetH		Monomer	62.0	52.4	40.5	2.1	1.7	1.3

UniProtKB A. N.	Protein name	Subcellular location	Subunit	D_0 $\left(\frac{\mu m^2}{s}\right)$			D_{cyto} $\left(\frac{\mu m^2}{s}\right)$		
				310 K	303 K	293 K	310 K	303 K	293 K
P31547	MetI	Cell inner membrane	Monomer	123.9	104.7	80.9	11.5	9.7	7.5
			Complex with SecB	72.2	59.0	47.1	3.1	2.5	2.0
			Complex with TF (Tig)	79.8	67.4	52.1	4.0	3.4	2.6
P0A8U6	MetJ	Cytoplasm	Monomer	159.9	135.1	104.3	20.0	16.9	13.0
			Homodimer	121.8	99.5	79.5	11.1	9.1	7.2
P0A817	MetK	Cytoplasm	Monomer	98.3	83.1	64.2	6.7	5.7	4.4
			Homotetramer	57.1	46.7	37.3	1.6	1.3	1.1
P00562	MetL		Monomer	73.3	61.9	47.8	3.2	2.7	2.1
			Homotetramer	42.5	34.8	27.8	0.7	0.6	0.4
P30750	MetN	Cell inner membrane	Monomer	102.4	86.6	66.9	7.4	6.3	4.8
			Complex with SecB	68.2	55.7	44.5	2.7	2.2	1.7
			Complex with TF (Tig)	74.2	62.7	48.4	3.3	2.8	2.2
P28635	MetQ	Cell membrane	Monomer	113.0	95.5	73.7	9.3	7.9	6.1
			Complex with SecB	70.4	57.5	45.9	2.9	2.4	1.9
			Complex with TF (Tig)	77.3	65.3	50.4	3.7	3.1	2.4
P0A9F9	MetR	Cytoplasm	Monomer	104.8	88.6	68.4	7.8	6.6	5.1
P30958	Mfd		Monomer	63.1	53.3	41.2	2.2	1.8	1.4
P0AAG8	MglA	Cell inner membrane	Monomer	87.6	74.0	57.1	5.1	4.3	3.3
			Complex with SecB	64.0	52.3	41.8	2.2	1.8	1.5
			Complex with TF (Tig)	68.7	58.1	44.9	2.7	2.3	1.8
P0AEE5	MglB	Periplasm	Monomer	104.7	88.5	68.4	7.8	6.6	5.1
			Complex with SecB	68.7	56.1	44.8	2.7	2.2	1.8
			Complex with TF (Tig)	74.9	63.3	48.9	3.4	2.9	2.2
P23200	MglC	Cell inner membrane	Monomer	104.9	88.6	68.5	7.9	6.6	5.1
			Complex with SecB	68.7	56.1	44.8	2.7	2.2	1.8
			Complex with TF (Tig)	75.0	63.4	48.9	3.4	2.9	2.2
P64512	MgrB		Monomer	217.3	183.6	141.8	37.0	31.2	24.1
P0A731	MgsA	Cytoplasm	Monomer	140.4	118.6	91.6	15.2	12.8	9.9
			Homohexamer	69.5	56.8	45.4	2.8	2.3	1.8
P0ABB8	MgtA	Cell inner membrane	Monomer	70.1	59.2	45.7	2.9	2.4	1.9
			Complex with SecB	57.0	46.6	37.2	1.6	1.3	1.1

UniProtKB A. N.	Protein name	Subcellular location	Subunit	D_0 $\left(\frac{\mu m^2}{s}\right)$			D_{cyto} $\left(\frac{\mu m^2}{s}\right)$		
				310 K	303 K	293 K	310 K	303 K	293 K
			Complex with TF (Tig)	60.0	50.7	39.2	1.9	1.6	1.2
E2JKY7	MgtL		Monomer	316.7	267.6	206.7	74.1	62.6	48.3
P77397	MhpA		Monomer	84.3	71.2	55.0	4.6	3.9	3.0
P0ABR9	MhpB		Monomer	106.5	90.0	69.5	8.1	6.9	5.3
			Homotetramer	61.9	50.5	40.4	2.0	1.7	1.3
P77044	MhpC		Monomer	108.6	91.7	70.9	8.5	7.2	5.6
			Homodimer	82.7	67.6	54.0	4.4	3.6	2.9
P77608	MhpD		Monomer	113.8	96.2	74.3	9.5	8.0	6.2
P51020	MhpE		Monomer	103.9	87.8	67.8	7.7	6.5	5.0
P77580	MhpF		Monomer	107.5	90.8	70.1	8.3	7.0	5.4
P77569	MhpR		Monomer	110.3	93.2	72.0	8.8	7.5	5.8
P77589	MhpT	Cell inner membrane	Monomer	98.7	83.4	64.4	6.8	5.7	4.4
			Complex with SecB	67.2	54.9	43.9	2.6	2.1	1.7
			Complex with TF (Tig)	73.0	61.7	47.6	3.2	2.7	2.1
P16384	MiaA		Monomer	105.5	89.1	68.8	8.0	6.7	5.2
P0AEI1	MiaB	Cytoplasm	Monomer	89.3	75.4	58.3	5.3	4.5	3.5
P18196	MinC		Monomer	120.9	102.1	78.9	10.9	9.2	7.1
			Homodimer	92.1	75.3	60.1	5.7	4.7	3.8
P0AEZ3	MinD	Cell inner membrane	Monomer	112.7	95.2	73.6	9.3	7.8	6.1
			Complex with SecB	70.3	57.4	45.9	2.9	2.4	1.9
			Complex with TF (Tig)	77.2	65.2	50.4	3.7	3.1	2.4
P0A734	MinE		Monomer	171.0	144.4	111.6	23.0	19.4	15.0
P03817	MioC		Monomer	144.2	121.8	94.1	16.0	13.6	10.5
			Homodimer	109.9	89.8	71.7	8.7	7.1	5.7
P0A908	MipA	Cell outer membrane	Monomer	115.5	97.6	75.4	9.8	8.3	6.4
			Complex with SecB	70.8	57.9	46.2	2.9	2.4	1.9
			Complex with TF (Tig)	77.9	65.8	50.8	3.8	3.2	2.5
P76506	MlaA	Cell outer membrane	Monomer	115.2	97.3	75.1	9.7	8.2	6.4
			Complex with SecB	70.8	57.8	46.2	2.9	2.4	1.9
			Complex with TF (Tig)	77.8	65.7	50.8	3.8	3.2	2.5
P64602	MlaB	Cytoplasm	Monomer	168.1	142.0	109.7	22.2	18.7	14.5

UniProtKB A. N.	Protein name	Subcellular location	Subunit	$D_0 \left(\frac{\mu m^2}{s}\right)$			$D_{\text{cyto}} \left(\frac{\mu m^2}{s}\right)$		
				310 K	303 K	293 K	310 K	303 K	293 K
P0ADV7	MlaC	Periplasm	Monomer	122.5	103.5	79.9	11.2	9.5	7.3
			Complex with SecB	72.0	58.8	47.0	3.1	2.5	2.0
			Complex with TF (Tig)	79.5	67.2	51.9	4.0	3.4	2.6
P64604	MlaD	Cell inner membrane	Monomer	132.6	112.0	86.5	13.4	11.3	8.7
			Complex with SecB	73.3	59.9	47.9	3.2	2.6	2.1
			Complex with TF (Tig)	81.5	68.8	53.2	4.2	3.6	2.8
P64606	MlaE	Cell inner membrane	Monomer	115.4	97.5	75.3	9.8	8.3	6.4
			Complex with SecB	70.8	57.8	46.2	2.9	2.4	1.9
			Complex with TF (Tig)	77.9	65.8	50.8	3.8	3.2	2.5
P63386	MlaF	Cell inner membrane	Monomer	113.5	95.9	74.1	9.4	8.0	6.2
P50456	Mlc		Monomer	96.2	81.3	62.8	6.4	5.4	4.2
			Homodimer	73.3	59.9	47.9	3.2	2.6	2.1
P28224	MliC	Cell outer membrane	Monomer	157.7	133.3	102.9	19.4	16.4	12.7
			Complex with SecB	75.7	61.9	49.4	3.5	2.9	2.3
			Complex with TF (Tig)	85.0	71.8	55.5	4.7	4.0	3.1
P33358	MlrA		Monomer	115.2	97.3	75.1	9.7	8.2	6.4
P0A935	MltA	Cell outer membrane	Monomer	99.8	84.3	65.1	7.0	5.9	4.6
			Complex with SecB	67.5	55.1	44.1	2.6	2.1	1.7
			Complex with TF (Tig)	73.4	62.0	47.9	3.2	2.7	2.1
P41052	MltB	Cell outer membrane	Monomer	99.9	84.4	65.2	7.0	5.9	4.6
			Complex with SecB	67.5	55.2	44.1	2.6	2.1	1.7
			Complex with TF (Tig)	73.4	62.0	47.9	3.2	2.7	2.1
P0C066	MltC	Cell outer membrane	Monomer	100.1	84.5	65.3	7.0	5.9	4.6
			Complex with SecB	67.6	55.2	44.1	2.6	2.1	1.7
			Complex with TF (Tig)	73.5	62.1	47.9	3.2	2.7	2.1
P0AEZ7	MltD	Cell membrane	Monomer	92.2	77.9	60.2	5.8	4.9	3.8
			Complex with SecB	65.4	53.5	42.7	2.4	1.9	1.6
			Complex with TF (Tig)	70.6	59.7	46.1	2.9	2.5	1.9
P0AGC5	MltF	Cell outer membrane	Monomer	86.4	73.0	56.4	4.9	4.1	3.2
			Complex with SecB	63.6	52.0	41.5	2.2	1.8	1.4
			Complex with TF (Tig)	68.3	57.7	44.5	2.7	2.3	1.7

UniProtKB A. N.	Protein name	Subcellular location	Subunit	D_0 $\left(\frac{\mu m^2}{s}\right)$			D_{cyto} $\left(\frac{\mu m^2}{s}\right)$		
				310 K	303 K	293 K	310 K	303 K	293 K
P52045	MmcD		Monomer	113.4	95.8	74.0	9.4	7.9	6.1
			Homotrimer	73.7	60.2	48.1	3.3	2.7	2.1
Q47690	MmuM		Monomer	107.5	90.8	70.1	8.3	7.0	5.4
Q47689	MmuP	Cell inner membrane	Monomer	91.4	77.2	59.7	5.6	4.8	3.7
			Complex with SecB	65.2	53.3	42.6	2.4	1.9	1.5
			Complex with TF (Tig)	70.3	59.4	45.9	2.9	2.4	1.9
P54746	MngB		Monomer	70.0	59.1	45.6	2.8	2.4	1.9
P13669	MngR		Monomer	114.8	97.0	74.9	9.7	8.2	6.3
P25745	MnmA	Cytoplasm	Monomer	99.3	83.9	64.8	6.9	5.8	4.5
P77182	MnmC	Cytoplasm	Monomer	78.5	66.4	51.3	3.9	3.3	2.5
P25522	MnmE	Cytoplasm	Monomer	92.4	78.0	60.3	5.8	4.9	3.8
			Homodimer	70.4	57.5	45.9	2.9	2.4	1.9
			Heterotetramer ($MnmE_2-MnmG_2$)	49.8	42.1	32.5	1.1	0.9	0.7
P0A6U3	MnmG	Cytoplasm	Monomer	80.7	68.2	52.6	4.1	3.5	2.7
			Homodimer	61.5	50.2	40.1	2.0	1.6	1.3
			Heterotetramer ($MnmE_2-MnmG_2$)	49.8	42.1	32.5	1.1	0.9	0.7
P0A769	MntH	Cell inner membrane	Monomer	96.3	81.4	62.9	6.4	5.4	4.2
			Complex with SecB	66.6	54.4	43.5	2.5	2.0	1.6
			Complex with TF (Tig)	72.2	61.0	47.1	3.1	2.6	2.0
P0A9F1	MntR	Cytoplasm	Monomer	138.1	116.7	90.1	14.6	12.4	9.5
			Homodimer	105.2	86.0	68.7	7.9	6.5	5.2
P30745	MoaA		Monomer	102.9	87.0	67.2	7.5	6.3	4.9
			Homodimer	78.4	64.1	51.2	3.8	3.1	2.5
P0AEZ9	MoaB		Monomer	135.1	114.1	88.1	13.9	11.8	9.1
			Homohexamer	66.9	54.7	43.7	2.5	2.1	1.7
P0A738	MoaC		Monomer	138.6	117.1	90.5	14.7	12.5	9.6
			Homohexamer	68.7	56.1	44.8	2.7	2.2	1.8
P30748	MoaD		Monomer	181.7	153.5	118.6	26.0	22.0	17.0
			Heterotetramer ($MoaD_2-MoaE_2$)	106.8	90.2	69.7	8.2	6.9	5.3
P30749	MoaE		Monomer	140.2	118.4	91.5	15.1	12.8	9.9
			Heterotetramer ($MoaD_2-MoaE_2$)	106.8	90.2	69.7	8.2	6.9	5.3

UniProtKB A. N.	Protein name	Subcellular location	Subunit	D_0 $\left(\frac{\mu m^2}{s}\right)$			D_{cyto} $\left(\frac{\mu m^2}{s}\right)$		
				310 K	303 K	293 K	310 K	303 K	293 K
P32173	MobA	Cytoplasm	Monomer	127.5	107.7	83.2	12.3	10.4	8.0
P32125	MobB		Monomer	133.1	112.5	86.9	13.5	11.4	8.8
			Homodimer	101.5	82.9	66.2	7.3	5.9	4.7
P37329	ModA	Periplasm	Monomer	116.3	98.2	75.9	10.0	8.4	6.5
			Complex with SecB	70.9	58.0	46.3	3.0	2.4	1.9
			Complex with TF (Tig)	78.1	66.0	51.0	3.8	3.2	2.5
P0AF01	ModB	Cell inner membrane	Monomer	120.6	101.9	78.7	10.8	9.1	7.1
			Complex with SecB	71.7	58.5	46.8	3.0	2.5	2.0
			Complex with TF (Tig)	79.1	66.8	51.6	3.9	3.3	2.6
P09833	ModC	Cell inner membrane	Monomer	101.1	85.4	66.0	7.2	6.1	4.7
			Complex with SecB	67.8	55.4	44.3	2.6	2.1	1.7
			Complex with TF (Tig)	73.8	62.3	48.1	3.3	2.8	2.1
P0A9G8	ModE	Cytoplasm	Monomer	114.8	97.0	74.9	9.7	8.2	6.3
			Homodimer	87.5	71.5	57.1	5.1	4.1	3.3
P31060	ModF	Cell inner membrane	Monomer	88.7	75.0	57.9	5.2	4.4	3.4
			Complex with SecB	64.4	52.6	42.0	2.3	1.9	1.5
			Complex with TF (Tig)	69.2	58.5	45.2	2.8	2.3	1.8
P12281	MoeA		Monomer	96.5	81.5	62.9	6.4	5.4	4.2
			Homodimer	73.5	60.1	48.0	3.2	2.7	2.1
P12282	MoeB		Monomer	117.4	99.1	76.6	10.2	8.6	6.6
			Homodimer	89.4	73.1	58.4	5.3	4.4	3.5
P0AF03	Mog		Monomer	128.4	108.5	83.8	12.5	10.5	8.1
			Homotrimer	83.5	68.2	54.5	4.5	3.7	2.9
P76096	MokB		Monomer	212.2	179.3	138.5	35.3	29.9	23.1
P33236	MokC	Membrane	Monomer	190.7	161.1	124.5	28.6	24.2	18.7
			Complex with SecB	77.6	63.4	50.6	3.7	3.0	2.4
			Complex with TF (Tig)	87.8	74.2	57.3	5.1	4.3	3.3
P09348	MotA	Cell inner membrane	Monomer	109.3	92.4	71.3	8.6	7.3	5.6
			Complex with SecB	69.7	56.9	45.5	2.8	2.3	1.8
			Complex with TF (Tig)	76.3	64.4	49.8	3.6	3.0	2.3
P0AF06	MotB	Cell inner membrane	Monomer	106.6	90.0	69.5	8.1	6.9	5.3

UniProtKB A. N.	Protein name	Subcellular location	Subunit	D_0 $\left(\frac{\mu m^2}{s}\right)$			D_{cyto} $\left(\frac{\mu m^2}{s}\right)$		
				310 K	303 K	293 K	310 K	303 K	293 K
			Complex with SecB	69.1	56.4	45.1	2.8	2.2	1.8
			Complex with TF (Tig)	75.5	63.8	49.3	3.5	2.9	2.3
P0ACV6	MpaA		Monomer	117.6	99.4	76.8	10.2	8.6	6.7
P37773	Mpl	Secreted	Monomer	91.9	77.6	60.0	5.7	4.8	3.7
			Complex with SecB	65.3	53.4	42.6	2.4	1.9	1.5
			Complex with TF (Tig)	70.5	59.6	46.0	2.9	2.5	1.9
P77348	MppA	Periplasm	Monomer	85.5	72.3	55.8	4.8	4.0	3.1
			Complex with SecB	63.3	51.7	41.3	2.2	1.8	1.4
			Complex with TF (Tig)	67.9	57.3	44.3	2.6	2.2	1.7
P0ACR9	MprA		Monomer	130.0	109.9	84.9	12.8	10.8	8.4
P33940	Mqo		Monomer	85.3	72.1	55.7	4.8	4.0	3.1
Q46864	MqsA		Monomer	148.3	125.3	96.8	17.1	14.4	11.1
			Homodimer	113.0	92.3	73.8	9.3	7.6	6.1
			Heterotetramer (MqsR$-$MqsA$_2$$-$MqsR)	90.5	76.4	59.0	5.5	4.6	3.6
Q46865	MqsR		Monomer	164.8	139.3	107.6	21.3	18.0	13.9
			Homodimer	125.6	102.6	82.0	11.9	9.7	7.7
			Heterotetramer (MqsR$-$MqsA$_2$$-$MqsR)	90.5	76.4	59.0	5.5	4.6	3.6
P0A6W3	MraY	Cell inner membrane	Monomer	100.3	84.7	65.5	7.1	6.0	4.6
			Complex with SecB	67.6	55.3	44.1	2.6	2.1	1.7
			Complex with TF (Tig)	73.5	62.1	48.0	3.2	2.7	2.1
P22186	MraZ		Monomer	138.9	117.3	90.6	14.8	12.5	9.7
			Homooctamer	61.5	50.2	40.1	2.0	1.6	1.3
P02918	MrcA	Cell inner membrane	Monomer	71.8	60.6	46.8	3.0	2.6	2.0
			Complex with SecB	57.8	47.2	37.7	1.7	1.4	1.1
			Complex with TF (Tig)	61.0	51.5	39.8	2.0	1.7	1.3
P02919	MrcB	Cell inner membrane	Monomer	71.6	60.5	46.7	3.0	2.6	2.0
			Complex with SecB	57.7	47.1	37.7	1.7	1.4	1.1
			Complex with TF (Tig)	60.9	51.4	39.7	2.0	1.7	1.3
P0AD65	MrdA	Cell inner membrane	Monomer	80.1	67.6	52.3	4.0	3.4	2.6
			Complex with SecB	61.3	50.1	40.0	2.0	1.6	1.3
			Complex with TF (Tig)	65.3	55.2	42.6	2.4	2.0	1.5

UniProtKB A. N.	Protein name	Subcellular location	Subunit	D_0 $\left(\frac{\mu m^2}{s}\right)$			D_{cyto} $\left(\frac{\mu m^2}{s}\right)$		
				310 K	303 K	293 K	310 K	303 K	293 K
P0ABG7	MrdB	Cell inner membrane	Monomer	99.7	84.3	65.1	7.0	5.9	4.5
			Complex with SecB	67.5	55.1	44.0	2.6	2.1	1.7
			Complex with TF (Tig)	73.3	62.0	47.9	3.2	2.7	2.1
P0A9X4	MreB		Monomer	103.4	87.3	67.4	7.6	6.4	4.9
P16926	MreC		Monomer	100.7	85.0	65.7	7.1	6.0	4.6
P0ABH4	MreD	Cell inner membrane	Monomer	134.7	113.8	87.9	13.9	11.7	9.0
			Complex with SecB	73.6	60.1	48.0	3.3	2.7	2.1
			Complex with TF (Tig)	81.9	69.2	53.4	4.3	3.6	2.8
P0AF08	Mrp		Monomer	100.3	84.7	65.4	7.1	6.0	4.6
P24202	Mrr		Monomer	107.4	90.7	70.1	8.3	7.0	5.4
P60752	MsbA	Cell inner membrane	Monomer	83.1	70.2	54.2	4.4	3.8	2.9
			Complex with SecB	62.5	51.0	40.8	2.1	1.7	1.4
			Complex with TF (Tig)	66.8	56.4	43.6	2.5	2.1	1.6
P24205	MsbB	Cell inner membrane	Monomer	102.9	86.9	67.1	7.5	6.3	4.9
			Complex with SecB	68.2	55.8	44.5	2.7	2.2	1.7
			Complex with TF (Tig)	74.4	62.8	48.5	3.3	2.8	2.2
P0A742	MscL	Cell inner membrane	Monomer	147.3	124.5	96.1	16.8	14.2	11.0
			Complex with SecB	74.9	61.2	48.9	3.4	2.8	2.2
			Complex with TF (Tig)	83.8	70.8	54.7	4.5	3.8	3.0
P0AAT4	MscM	Cell inner membrane	Monomer	94.4	79.7	61.6	6.1	5.1	4.0
			Complex with SecB	66.1	54.0	43.1	2.4	2.0	1.6
			Complex with TF (Tig)	71.4	60.4	46.6	3.0	2.5	2.0
P0C0S1	MscS	Cell inner membrane	Monomer	110.9	93.7	72.3	8.9	7.5	5.8
			Complex with SecB	70.0	57.1	45.7	2.8	2.3	1.9
			Complex with TF (Tig)	76.7	64.8	50.0	3.6	3.1	2.4
P0A744	MsrA		Monomer	123.8	104.6	80.8	11.5	9.7	7.5
P0A746	MsrB		Monomer	145.5	122.9	94.9	16.4	13.8	10.7
P25738	MsyB		Monomer	150.1	126.8	98.0	17.5	14.8	11.4
P76346	MtfA	Cytoplasm	Monomer	111.7	94.4	72.9	9.1	7.7	5.9
P46022	MtgA	Cell membrane	Monomer	116.3	98.3	75.9	10.0	8.4	6.5
			Complex with SecB	71.0	58.0	46.3	3.0	2.4	1.9

UniProtKB A. N.	Protein name	Subcellular location	Subunit	D_0 $\left(\frac{\mu m^2}{s}\right)$			D_{cyto} $\left(\frac{\mu m^2}{s}\right)$		
				310 K	303 K	293 K	310 K	303 K	293 K
			Complex with TF (Tig)	78.1	66.0	51.0	3.8	3.2	2.5
P00550	MtlA	Cell inner membrane	Monomer	81.4	68.8	53.1	4.2	3.6	2.8
			Complex with SecB	61.8	50.5	40.3	2.0	1.7	1.3
			Complex with TF (Tig)	66.0	55.7	43.0	2.4	2.1	1.6
P09424	MtlD		Monomer	99.1	83.7	64.7	6.9	5.8	4.5
P0AF10	MtlR		Monomer	126.7	107.0	82.7	12.1	10.2	7.9
P0AF12	MtnN		Monomer	121.7	102.8	79.4	11.1	9.3	7.2
			Homodimer	92.7	75.8	60.5	5.8	4.8	3.8
P0AAD2	Mtr	Cell inner membrane	Monomer	96.2	81.3	62.8	6.4	5.4	4.2
			Complex with SecB	66.6	54.4	43.4	2.5	2.0	1.6
			Complex with TF (Tig)	72.1	60.9	47.1	3.1	2.6	2.0
P0A9H1	Mug	Cytoplasm	Monomer	135.1	114.1	88.1	13.9	11.8	9.1
P22523	MukB	Cytoplasm > nucleoid	Monomer	56.8	48.0	37.1	1.6	1.4	1.0
			Homodimer	43.3	35.4	28.2	0.7	0.6	0.5
P22524	MukE	Cytoplasm > nucleoid	Monomer	116.9	98.8	76.3	10.1	8.5	6.6
P60293	MukF	Cytoplasm > nucleoid	Monomer	91.4	77.2	59.6	5.6	4.8	3.7
P0A749	MurA	Cytoplasm	Monomer	95.8	81.0	62.5	6.3	5.3	4.1
P08373	MurB	Cytoplasm	Monomer	102.4	86.5	66.8	7.4	6.3	4.8
P17952	MurC	Cytoplasm	Monomer	89.3	75.5	58.3	5.3	4.5	3.5
P14900	MurD	Cytoplasm	Monomer	94.1	79.5	61.4	6.0	5.1	3.9
P22188	MurE	Cytoplasm	Monomer	89.5	75.6	58.4	5.4	4.5	3.5
P11880	MurF	Cytoplasm	Monomer	93.7	79.2	61.1	6.0	5.1	3.9
P17443	MurG	Cell inner membrane	Monomer	102.4	86.5	66.8	7.4	6.3	4.8
			Complex with SecB	68.1	55.7	44.5	2.7	2.2	1.7
			Complex with TF (Tig)	74.2	62.7	48.4	3.3	2.8	2.2
P22634	MurI		Monomer	110.7	93.5	72.2	8.9	7.5	5.8
P77272	MurP	Cell inner membrane	Monomer	91.9	77.7	60.0	5.7	4.8	3.7
			Complex with SecB	65.4	53.4	42.7	2.4	1.9	1.5
			Complex with TF (Tig)	70.5	59.6	46.0	2.9	2.5	1.9

UniProtKB A. N.	Protein name	Subcellular location	Subunit	D_0 $\left(\frac{\mu m^2}{s}\right)$			D_{cyto} $\left(\frac{\mu m^2}{s}\right)$		
				310 K	303 K	293 K	310 K	303 K	293 K
P76535	MurQ		Monomer	110.4	93.3	72.0	8.8	7.5	5.8
			Homodimer	84.1	68.7	54.9	4.6	3.8	3.0
P77245	MurR		Monomer	110.5	93.3	72.1	8.9	7.5	5.8
			Homotetramer	64.1	52.4	41.9	2.3	1.8	1.5
P06722	MutH	Cytoplasm	Monomer	119.5	100.9	78.0	10.6	9.0	6.9
P23367	MutL		Monomer	81.4	68.8	53.1	4.2	3.6	2.8
P05523	MutM		Monomer	111.7	94.4	72.9	9.1	7.7	5.9
P23909	MutS		Monomer	71.3	60.2	46.5	3.0	2.5	2.0
P08337	MutT		Monomer	147.4	124.6	96.2	16.8	14.2	11.0
P17802	MutY		Monomer	101.0	85.4	65.9	7.2	6.1	4.7
P75931	MviM		Monomer	107.2	90.5	69.9	8.3	7.0	5.4
P0AF16	MviN	Cell inner membrane	Monomer	88.3	74.6	57.6	5.2	4.4	3.4
			Complex with SecB	64.2	52.5	41.9	2.3	1.8	1.5
			Complex with TF (Tig)	69.0	58.3	45.0	2.7	2.3	1.8
Q47005	Nac		Monomer	108.3	91.5	70.6	8.4	7.1	5.5
P11458	NadA	Cytoplasm	Monomer	102.0	86.1	66.5	7.3	6.2	4.8
			Homodimer	77.7	63.5	50.7	3.8	3.1	2.4
P10902	NadB	Cytoplasm	Monomer	85.3	72.0	55.6	4.7	4.0	3.1
P30011	NadC		Monomer	108.3	91.5	70.7	8.5	7.2	5.5
			Homodimer	82.6	67.5	53.9	4.4	3.6	2.9
P0A752	NadD		Monomer	121.4	102.5	79.2	11.0	9.3	7.2
P18843	NadE		Monomer	111.2	94.0	72.6	9.0	7.6	5.9
			Homodimer	84.8	69.2	55.3	4.7	3.8	3.1
P27278	NadR	Cell membrane	Monomer	93.8	79.2	61.2	6.0	5.1	3.9
			Homotetramer	54.5	44.5	35.5	1.4	1.2	0.9
P0AF18	NagA		Monomer	99.3	83.9	64.8	6.9	5.8	4.5
P0A759	NagB		Monomer	112.5	95.0	73.4	9.2	7.8	6.0
			Homotrimer	73.1	59.7	47.7	3.2	2.6	2.1
			Homohexamer	55.7	47.1	36.4	1.5	1.3	1.0
P0AF20	NagC		Monomer	96.1	81.1	62.7	6.4	5.4	4.2
P0AF24	NagD		Monomer	116.6	98.5	76.1	10.0	8.5	6.5

UniProtKB A. N.	Protein name	Subcellular location	Subunit	D_0 $\left(\frac{\mu m^2}{s}\right)$			D_{cyto} $\left(\frac{\mu m^2}{s}\right)$		
				310 K	303 K	293 K	310 K	303 K	293 K
P09323	NagE	Cell inner membrane	Monomer	81.2	68.6	53.0	4.2	3.5	2.7
			Complex with SecB	61.8	50.4	40.3	2.0	1.7	1.3
			Complex with TF (Tig)	65.9	55.7	43.0	2.4	2.0	1.6
P75959	NagK		Monomer	108.0	91.2	70.5	8.4	7.1	5.5
P75949	NagZ	Cytoplasm	Monomer	102.7	86.7	67.0	7.5	6.3	4.9
P0A6L4	NanA	Cytoplasm	Monomer	108.6	91.7	70.8	8.5	7.2	5.6
			Homotetramer	63.0	51.5	41.1	2.2	1.8	1.4
P69856	NanC	Cell outer membrane	Monomer	115.4	97.5	75.3	9.8	8.3	6.4
			Complex with SecB	70.8	57.8	46.2	2.9	2.4	1.9
			Complex with TF (Tig)	77.9	65.8	50.8	3.8	3.2	2.5
P0A761	NanE		Monomer	122.3	103.3	79.8	11.2	9.4	7.3
P45425	NanK		Monomer	112.7	95.2	73.5	9.3	7.8	6.1
			Homodimer	85.9	70.1	56.0	4.8	3.9	3.2
P39371	NanM	Periplasm	Monomer	100.6	85.0	65.7	7.1	6.0	4.6
			Complex with SecB	67.7	55.3	44.2	2.6	2.1	1.7
			Complex with TF (Tig)	73.6	62.2	48.0	3.3	2.8	2.1
P0A8W0	NanR		Monomer	112.9	95.3	73.6	9.3	7.9	6.1
P39370	NanS	Periplasm	Monomer	103.4	87.4	67.5	7.6	6.4	5.0
			Complex with SecB	68.4	55.9	44.6	2.7	2.2	1.7
			Complex with TF (Tig)	74.5	63.0	48.6	3.4	2.8	2.2
P41036	NanT	Cell inner membrane	Monomer	89.4	75.5	58.3	5.3	4.5	3.5
			Complex with SecB	64.6	52.8	42.1	2.3	1.9	1.5
			Complex with TF (Tig)	69.5	58.7	45.3	2.8	2.4	1.8
P33937	NapA	Periplasm	Monomer	72.0	60.8	47.0	3.1	2.6	2.0
			Complex with SecB	57.9	47.3	37.8	1.7	1.4	1.1
			Complex with TF (Tig)	61.1	51.6	39.9	2.0	1.7	1.3
P0ABL3	NapB	Periplasm	Monomer	142.5	120.4	93.0	15.6	13.2	10.2
			Complex with SecB	74.4	60.8	48.6	3.4	2.7	2.2
			Complex with TF (Tig)	83.1	70.2	54.2	4.4	3.8	2.9
P0ABL5	NapC	Cell inner membrane	Monomer	124.2	105.0	81.1	11.6	9.8	7.6
			Complex with SecB	72.2	59.0	47.1	3.1	2.5	2.0

UniProtKB A. N.	Protein name	Subcellular location	Subunit	D_0 $\left(\frac{\mu m^2}{s}\right)$			D_{cyto} $\left(\frac{\mu m^2}{s}\right)$		
				310 K	303 K	293 K	310 K	303 K	293 K
			Complex with TF (Tig)	79.9	67.5	52.1	4.0	3.4	2.6
P0A9I5	NapD	Cytoplasm	Monomer	176.2	148.9	115.0	24.4	20.6	15.9
P0AAL0	NapF		Monomer	136.9	115.6	89.3	14.3	12.1	9.4
P0AAL3	NapG		Monomer	120.6	101.9	78.7	10.8	9.1	7.1
P33934	NapH	Cell inner membrane	Monomer	109.5	92.5	71.5	8.7	7.3	5.7
			Complex with SecB	69.7	56.9	45.5	2.8	2.3	1.8
			Complex with TF (Tig)	76.3	64.5	49.8	3.6	3.0	2.3
P09152	NarG	Cell membrane	Monomer	61.2	51.7	40.0	2.0	1.7	1.3
			Complex with SecB	52.3	42.8	34.2	1.3	1.0	0.8
			Complex with TF (Tig)	54.5	46.1	35.6	1.4	1.2	0.9
P11349	NarH	Cell membrane	Monomer	86.6	73.1	56.5	4.9	4.2	3.2
			Complex with SecB	63.7	52.0	41.5	2.2	1.8	1.4
			Complex with TF (Tig)	68.3	57.7	44.6	2.7	2.3	1.7
P11350	NarI	Cell inner membrane	Monomer	119.5	101.0	78.0	10.6	9.0	6.9
			Complex with SecB	71.5	58.4	46.7	3.0	2.5	2.0
			Complex with TF (Tig)	78.9	66.6	51.5	3.9	3.3	2.5
P0AF26	NarJ	Cytoplasm	Monomer	117.8	99.5	76.9	10.3	8.7	6.7
P10903	NarK	Cell inner membrane	Monomer	92.0	77.7	60.0	5.7	4.8	3.7
			Complex with SecB	65.4	53.4	42.7	2.4	1.9	1.6
			Complex with TF (Tig)	70.5	59.6	46.0	2.9	2.5	1.9
P0AF28	NarL		Monomer	122.5	103.5	80.0	11.2	9.5	7.3
P31802	NarP		Monomer	123.3	104.1	80.4	11.4	9.6	7.4
P27896	NarQ	Cell inner membrane	Monomer	83.5	70.5	54.5	4.5	3.8	2.9
			Complex with SecB	62.6	51.1	40.8	2.1	1.7	1.4
			Complex with TF (Tig)	66.9	56.6	43.7	2.5	2.1	1.7
P37758	NarU	Cell inner membrane	Monomer	91.9	77.6	60.0	5.7	4.8	3.7
			Complex with SecB	65.3	53.4	42.6	2.4	1.9	1.5
			Complex with TF (Tig)	70.5	59.6	46.0	2.9	2.5	1.9
P0AF32	NarV	Cell inner membrane	Monomer	118.6	100.2	77.4	10.4	8.8	6.8
			Complex with SecB	71.3	58.3	46.6	3.0	2.5	2.0
			Complex with TF (Tig)	78.6	66.4	51.3	3.9	3.3	2.5

UniProtKB A. N.	Protein name	Subcellular location	Subunit	D_0 $\left(\frac{\mu m^2}{s}\right)$			D_{cyto} $\left(\frac{\mu m^2}{s}\right)$		
				310 K	303 K	293 K	310 K	303 K	293 K
P19317	NarW	Cytoplasm	Monomer	118.3	100.0	77.2	10.4	8.8	6.8
P0AFA2	NarX	Cell inner membrane	Monomer	81.8	69.1	53.4	4.3	3.6	2.8
			Complex with SecB	62.0	50.6	40.4	2.1	1.7	1.3
			Complex with TF (Tig)	66.2	55.9	43.2	2.5	2.1	1.6
P19318	NarY	Cell membrane	Monomer	86.3	72.9	56.3	4.9	4.1	3.2
			Complex with SecB	63.6	51.9	41.5	2.2	1.8	1.4
			Complex with TF (Tig)	68.2	57.6	44.5	2.7	2.2	1.7
P19319	NarZ	Cell membrane	Monomer	61.3	51.8	40.0	2.0	1.7	1.3
			Complex with SecB	52.4	42.8	34.2	1.3	1.0	0.8
			Complex with TF (Tig)	54.6	46.1	35.6	1.4	1.2	0.9
P00393	Ndh	Cell membrane	Monomer	93.8	79.2	61.2	6.0	5.1	3.9
			Complex with SecB	65.9	53.8	43.0	2.4	2.0	1.6
			Complex with TF (Tig)	71.2	60.2	46.5	3.0	2.5	1.9
P0A763	Ndk	Cytoplasm	Monomer	145.4	122.9	94.9	16.3	13.8	10.7
			Homotetramer	84.5	69.0	55.1	4.6	3.8	3.0
P50465	Nei		Monomer	112.4	94.9	73.3	9.2	7.8	6.0
P77258	NemA		Monomer	100.7	85.0	65.7	7.1	6.0	4.6
P67430	NemR		Monomer	126.0	106.5	82.2	12.0	10.1	7.8
P0ADL1	NepI	Cell inner membrane	Monomer	98.4	83.2	64.2	6.7	5.7	4.4
			Complex with SecB	67.2	54.9	43.8	2.6	2.1	1.7
			Complex with TF (Tig)	72.9	61.6	47.6	3.2	2.7	2.1
P68739	Nfi	Cytoplasm	Monomer	121.1	102.3	79.0	10.9	9.2	7.1
P38489	NfnB		Monomer	122.6	103.6	80.0	11.2	9.5	7.3
			Homodimer	93.4	76.3	61.0	5.9	4.9	3.9
P0A6C1	Nfo		Monomer	110.1	93.0	71.8	8.8	7.4	5.7
P31600	NfrA	Cell outer membrane	Monomer	67.1	56.7	43.8	2.5	2.2	1.7
			Complex with SecB	55.5	45.3	36.2	1.5	1.2	1.0
			Complex with TF (Tig)	58.3	49.2	38.0	1.7	1.5	1.1
P0AFA5	NfrB	Cell inner membrane	Monomer	74.4	62.9	48.6	3.4	2.8	2.2
			Complex with SecB	59.0	48.2	38.5	1.8	1.5	1.2
			Complex with TF (Tig)	62.5	52.8	40.8	2.1	1.8	1.4

UniProtKB A. N.	Protein name	Subcellular location	Subunit	D_0 $\left(\frac{\mu m^2}{s}\right)$			D_{cyto} $\left(\frac{\mu m^2}{s}\right)$		
				310 K	303 K	293 K	310 K	303 K	293 K
P17117	NfsA		Monomer	117.2	99.0	76.5	10.2	8.6	6.6
P63020	NfuA		Monomer	129.0	109.0	84.2	12.6	10.6	8.2
			Homodimer	98.3	80.3	64.1	6.7	5.5	4.4
P13738	NhaA	Cell inner membrane	Monomer	98.9	83.5	64.5	6.8	5.8	4.5
			Complex with SecB	67.3	55.0	43.9	2.6	2.1	1.7
			Complex with TF (Tig)	73.1	61.7	47.7	3.2	2.7	2.1
P0AFA7	NhaB	Cell inner membrane	Monomer	87.4	73.8	57.0	5.0	4.3	3.3
			Complex with SecB	63.9	52.2	41.7	2.2	1.8	1.5
			Complex with TF (Tig)	68.7	58.0	44.8	2.7	2.3	1.8
P0A9G2	NhaR	Cytoplasm	Monomer	106.4	89.9	69.5	8.1	6.9	5.3
P77567	NhoA		Monomer	109.0	92.1	71.1	8.6	7.3	5.6
P0ACD4	NifU		Monomer	151.8	128.3	99.1	17.9	15.1	11.7
P33590	NikA	Periplasm	Monomer	86.2	72.8	56.2	4.9	4.1	3.2
			Complex with SecB	63.5	51.9	41.5	2.2	1.8	1.4
			Complex with TF (Tig)	68.1	57.6	44.5	2.7	2.2	1.7
P33591	NikB	Cell inner membrane	Monomer	105.3	88.9	68.7	7.9	6.7	5.2
			Complex with SecB	68.8	56.2	44.9	2.7	2.2	1.8
			Complex with TF (Tig)	75.1	63.4	49.0	3.4	2.9	2.2
P0AFA9	NikC	Cell inner membrane	Monomer	111.6	94.3	72.8	9.1	7.7	5.9
			Complex with SecB	70.1	57.3	45.7	2.9	2.3	1.9
			Complex with TF (Tig)	76.9	65.0	50.2	3.7	3.1	2.4
P33593	NikD	Cell inner membrane	Monomer	117.2	99.0	76.5	10.1	8.6	6.6
			Complex with SecB	71.1	58.1	46.4	3.0	2.4	1.9
			Complex with TF (Tig)	78.3	66.2	51.1	3.8	3.2	2.5
P33594	NikE	Cell inner membrane	Monomer	112.6	95.1	73.5	9.3	7.8	6.0
			Complex with SecB	70.3	57.4	45.9	2.9	2.4	1.9
			Complex with TF (Tig)	77.1	65.2	50.3	3.7	3.1	2.4
P0A6Z6	NikR		Monomer	146.8	124.0	95.8	16.7	14.1	10.9
			Homotetramer	85.3	69.6	55.6	4.7	3.9	3.1
Q47270	NinE		Monomer	204.3	172.6	133.3	32.8	27.7	21.4
P08201	NirB		Monomer	71.9	60.8	46.9	3.1	2.6	2.0

UniProtKB A. N.	Protein name	Subcellular location	Subunit	D_0 $\left(\frac{\mu m^2}{s}\right)$			D_{cyto} $\left(\frac{\mu m^2}{s}\right)$		
				310 K	303 K	293 K	310 K	303 K	293 K
			Homodimer	54.8	44.8	35.8	1.5	1.2	0.9
P0AC26	NirC	Cell inner membrane	Monomer	114.3	96.6	74.6	9.6	8.1	6.3
			Complex with SecB	70.6	57.7	46.1	2.9	2.4	1.9
			Complex with TF (Tig)	77.6	65.6	50.6	3.7	3.2	2.4
P0A9I8	NirD	Cytoplasm	Monomer	159.2	134.5	103.9	19.8	16.7	12.9
P04846	NlpA	Cell inner membrane	Monomer	113.0	95.5	73.7	9.3	7.9	6.1
			Complex with SecB	70.4	57.5	45.9	2.9	2.4	1.9
			Complex with TF (Tig)	77.3	65.3	50.4	3.7	3.1	2.4
P0A903	NlpB	Cell outer membrane	Monomer	103.5	87.4	67.5	7.6	6.4	5.0
			Complex with SecB	68.4	55.9	44.6	2.7	2.2	1.7
			Complex with TF (Tig)	74.5	63.0	48.6	3.4	2.8	2.2
P23898	NlpC	Cell membrane	Monomer	139.2	117.6	90.8	14.9	12.6	9.7
			Complex with SecB	74.1	60.5	48.3	3.3	2.7	2.2
			Complex with TF (Tig)	82.6	69.8	53.9	4.4	3.7	2.9
P0ADA3	NlpD	Cell inner membrane	Monomer	100.0	84.5	65.3	7.0	5.9	4.6
			Complex with SecB	67.6	55.2	44.1	2.6	2.1	1.7
			Complex with TF (Tig)	73.4	62.0	47.9	3.2	2.7	2.1
P40710	NlpE	Cell outer membrane	Monomer	118.9	100.4	77.6	10.5	8.9	6.8
			Complex with SecB	71.4	58.3	46.6	3.0	2.5	2.0
			Complex with TF (Tig)	78.7	66.5	51.4	3.9	3.3	2.5
P0AFB1	NlpI	Cell membrane	Monomer	107.3	90.6	70.0	8.3	7.0	5.4
			Complex with SecB	69.2	56.6	45.2	2.8	2.3	1.8
			Complex with TF (Tig)	75.7	63.9	49.4	3.5	3.0	2.3
P21420	NmpC	Cell outer membrane	Monomer	99.9	84.4	65.2	7.0	5.9	4.6
			Complex with SecB	67.5	55.2	44.1	2.6	2.1	1.7
			Complex with TF (Tig)	73.4	62.0	47.9	3.2	2.7	2.1
P31061	NohA		Monomer	128.0	108.2	83.5	12.4	10.5	8.1
P31062	NohB		Monomer	130.4	110.2	85.1	12.9	10.9	8.4
P37013	NorR		Monomer	88.3	74.6	57.6	5.2	4.4	3.4
Q46877	NorV	Cytoplasm	Monomer	88.9	75.1	58.0	5.3	4.5	3.4
			Homotetramer	51.6	42.2	33.7	1.2	1.0	0.8

UniProtKB A. N.	Protein name	Subcellular location	Subunit	D_0 $\left(\frac{\mu m^2}{s}\right)$			D_{cyto} $\left(\frac{\mu m^2}{s}\right)$		
				310 K	303 K	293 K	310 K	303 K	293 K
P37596	NorW	Cytoplasm	Monomer	98.8	83.5	64.5	6.8	5.8	4.4
P00452	NrdA		Monomer	74.3	62.8	48.5	3.3	2.8	2.2
P69924	NrdB		Monomer	96.9	81.9	63.3	6.5	5.5	4.2
P28903	NrdD		Monomer	76.3	64.5	49.8	3.6	3.0	2.3
P39452	NrdE		Monomer	76.2	64.4	49.7	3.6	3.0	2.3
P37146	NrdF		Monomer	103.9	87.8	67.8	7.7	6.5	5.0
P0A9N8	NrdG	Cytoplasm	Monomer	138.7	117.2	90.5	14.8	12.5	9.6
P0AC65	NrdH		Monomer	178.7	151.0	116.6	25.1	21.2	16.4
P0A772	NrdI		Monomer	145.9	123.2	95.2	16.5	13.9	10.7
P0A8D0	NrdR		Monomer	139.4	117.8	91.0	14.9	12.6	9.7
P0ABK9	NrfA	Periplasm	Monomer	89.3	75.4	58.2	5.3	4.5	3.5
			Complex with SecB	64.5	52.7	42.1	2.3	1.9	1.5
			Complex with TF (Tig)	69.4	58.7	45.3	2.8	2.4	1.8
P0ABL1	NrfB	Periplasm	Monomer	129.7	109.6	84.6	12.7	10.8	8.3
			Complex with SecB	73.0	59.6	47.6	3.2	2.6	2.1
			Complex with TF (Tig)	81.0	68.4	52.8	4.2	3.5	2.7
P0AAK7	NrfC		Monomer	121.3	102.5	79.1	11.0	9.3	7.2
P32709	NrfD	Cell inner membrane	Monomer	105.5	89.1	68.9	8.0	6.7	5.2
			Complex with SecB	68.9	56.2	44.9	2.7	2.2	1.8
			Complex with TF (Tig)	75.2	63.5	49.1	3.4	2.9	2.2
P32710	NrfE	Cell inner membrane	Monomer	84.8	71.6	55.3	4.7	4.0	3.1
			Complex with SecB	63.0	51.5	41.1	2.1	1.8	1.4
			Complex with TF (Tig)	67.5	57.0	44.1	2.6	2.2	1.7
P32711	NrfF	Periplasm	Monomer	149.0	125.9	97.3	17.2	14.6	11.2
			Complex with SecB	75.0	61.3	49.0	3.4	2.8	2.2
			Complex with TF (Tig)	84.0	71.0	54.8	4.6	3.9	3.0
P32712	NrfG		Monomer	124.9	105.5	81.5	11.7	9.9	7.6
P0A9E9	Nsr	Cytoplasm	Monomer	119.9	101.3	78.2	10.7	9.0	7.0
			Homodimer	91.4	74.7	59.6	5.6	4.6	3.7
P0AF63	NsrR		Monomer	144.9	122.5	94.6	16.2	13.7	10.6
P0AB83	Nth		Monomer	123.3	104.2	80.5	11.4	9.6	7.4

UniProtKB A. N.	Protein name	Subcellular location	Subunit	$D_0 \left(\frac{\mu m^2}{s}\right)$			$D_{\text{cyto}} \left(\frac{\mu m^2}{s}\right)$		
				310 K	303 K	293 K	310 K	303 K	293 K
P0AFC0	NudB		Monomer	139.1	117.5	90.8	14.9	12.6	9.7
P32664	NudC		Monomer	112.6	95.1	73.5	9.3	7.8	6.0
			Homodimer	85.8	70.1	56.0	4.8	3.9	3.1
P32056	NudD		Monomer	136.2	115.1	88.9	14.2	12.0	9.3
			Homodimer	103.8	84.8	67.7	7.7	6.3	5.0
P45799	NudE		Monomer	128.6	108.7	83.9	12.5	10.6	8.2
			Homodimer	98.0	80.1	64.0	6.7	5.5	4.4
Q93K97	NudF		Monomer	123.1	104.0	80.3	11.3	9.6	7.4
			Homodimer	93.8	76.6	61.2	6.0	4.9	3.9
P77788	NudG		Monomer	147.0	124.2	95.9	16.7	14.1	10.9
P52006	NudI		Monomer	142.2	120.1	92.8	15.6	13.2	10.2
P0AEI6	NudJ		Monomer	138.7	117.2	90.5	14.8	12.5	9.6
P37128	NudK		Monomer	127.2	107.5	83.0	12.2	10.3	8.0
			Homodimer	97.0	79.2	63.3	6.5	5.3	4.2
P43337	NudL		Monomer	127.9	108.1	83.5	12.4	10.4	8.1
P0AFC3	NuoA	Cell inner membrane	Monomer	141.9	119.9	92.6	15.5	13.1	10.1
			Complex with SecB	74.4	60.8	48.5	3.3	2.7	2.2
			Complex with TF (Tig)	83.0	70.1	54.2	4.4	3.7	2.9
P0AFC7	NuoB	Cell inner membrane	Monomer	120.4	101.7	78.5	10.8	9.1	7.0
P33599	NuoC	Cytoplasm. Cell inner membrane	Monomer	81.3	68.7	53.0	4.2	3.6	2.7
P0AFD1	NuoE		Monomer	135.3	114.3	88.3	14.0	11.8	9.1
P31979	NuoF		Monomer	92.3	78.0	60.2	5.8	4.9	3.8
P33602	NuoG	Cytoplasm. Cell inner membrane	Monomer	69.9	59.0	45.6	2.8	2.4	1.9
P0AFD4	NuoH	Cell inner membrane	Monomer	104.2	88.0	68.0	7.7	6.5	5.0
			Complex with SecB	68.5	56.0	44.7	2.7	2.2	1.8
			Complex with TF (Tig)	74.8	63.2	48.8	3.4	2.9	2.2
P0AFD6	NuoI	Cell inner membrane	Monomer	130.1	109.9	84.9	12.8	10.8	8.4
			Complex with SecB	73.0	59.7	47.6	3.2	2.6	2.1
			Complex with TF (Tig)	81.0	68.5	52.9	4.2	3.5	2.7

UniProtKB A. N.	Protein name	Subcellular location	Subunit	D_0 $\left(\frac{\mu m^2}{s}\right)$			D_{cyto} $\left(\frac{\mu m^2}{s}\right)$		
				310 K	303 K	293 K	310 K	303 K	293 K
P0AFE0	NuoJ	Cell inner membrane	Monomer	131.8	111.3	86.0	13.2	11.2	8.6
			Complex with SecB	73.2	59.8	47.8	3.2	2.6	2.1
			Complex with TF (Tig)	81.3	68.7	53.1	4.2	3.6	2.8
P0AFE4	NuoK	Cell inner membrane	Monomer	167.1	141.2	109.1	21.9	18.5	14.3
			Complex with SecB	76.4	62.4	49.8	3.6	2.9	2.3
			Complex with TF (Tig)	86.0	72.7	56.1	4.9	4.1	3.2
P33607	NuoL	Cell inner membrane	Monomer	82.1	69.4	53.6	4.3	3.6	2.8
			Complex with SecB	62.1	50.7	40.5	2.1	1.7	1.3
			Complex with TF (Tig)	66.3	56.0	43.3	2.5	2.1	1.6
P0AFE8	NuoM	Cell inner membrane	Monomer	87.5	73.9	57.1	5.1	4.3	3.3
			Complex with SecB	64.0	52.3	41.7	2.2	1.8	1.5
			Complex with TF (Tig)	68.7	58.0	44.8	2.7	2.3	1.8
P0AFF0	NuoN	Cell inner membrane	Monomer	90.4	76.3	59.0	5.5	4.6	3.6
			Complex with SecB	64.9	53.0	42.3	2.3	1.9	1.5
			Complex with TF (Tig)	69.9	59.0	45.6	2.8	2.4	1.9
P0AFF2	NupC	Cell inner membrane	Monomer	97.0	81.9	63.3	6.5	5.5	4.2
			Complex with SecB	66.8	54.6	43.6	2.5	2.1	1.6
			Complex with TF (Tig)	72.4	61.2	47.2	3.1	2.6	2.0
P0AFF4	NupG	Cell inner membrane	Monomer	94.5	79.9	61.7	6.1	5.2	4.0
			Complex with SecB	66.1	54.0	43.1	2.4	2.0	1.6
			Complex with TF (Tig)	71.5	60.4	46.7	3.0	2.5	2.0
P33021	NupX	Cell inner membrane	Monomer	97.0	82.0	63.3	6.5	5.5	4.3
			Complex with SecB	66.8	54.6	43.6	2.5	2.1	1.6
			Complex with TF (Tig)	72.4	61.2	47.2	3.1	2.6	2.0
P0AFF6	NusA		Monomer	88.5	74.8	57.8	5.2	4.4	3.4
P0A780	NusB		Monomer	144.6	122.2	94.4	16.2	13.6	10.5
P0AFG0	NusG		Monomer	130.1	109.9	84.9	12.8	10.8	8.4
P42641	ObgE	Cytoplasm	Monomer	97.1	82.1	63.4	6.5	5.5	4.3
P37057	OgrK		Monomer	184.5	155.8	120.4	26.8	22.6	17.5
P0AFH0	Ogt		Monomer	133.6	112.9	87.2	13.6	11.5	8.9
P0A910	OmpA	Cell outer membrane	Monomer	103.1	87.1	67.3	7.5	6.4	4.9

UniProtKB A. N.	Protein name	Subcellular location	Subunit	D_0 $\left(\frac{\mu m^2}{s}\right)$			D_{cyto} $\left(\frac{\mu m^2}{s}\right)$		
				310 K	303 K	293 K	310 K	303 K	293 K
			Complex with SecB	68.3	55.8	44.6	2.7	2.2	1.7
			Complex with TF (Tig)	74.4	62.9	48.6	3.4	2.8	2.2
P06996	OmpC	Cell outer membrane	Monomer	99.8	84.3	65.1	7.0	5.9	4.6
			Complex with SecB	67.5	55.2	44.1	2.6	2.1	1.7
			Complex with TF (Tig)	73.4	62.0	47.9	3.2	2.7	2.1
P02931	OmpF	Cell outer membrane	Monomer	100.9	85.2	65.8	7.2	6.0	4.7
			Complex with SecB	67.8	55.4	44.2	2.6	2.1	1.7
			Complex with TF (Tig)	73.7	62.3	48.1	3.3	2.8	2.1
P76045	OmpG	Cell outer membrane	Monomer	105.7	89.3	69.0	8.0	6.7	5.2
			Complex with SecB	68.9	56.3	45.0	2.7	2.2	1.8
			Complex with TF (Tig)	75.2	63.5	49.1	3.4	2.9	2.2
P76773	OmpL	Cell outer membrane	Monomer	116.5	98.5	76.0	10.0	8.5	6.5
			Complex with SecB	71.0	58.0	46.3	3.0	2.4	1.9
			Complex with TF (Tig)	78.1	66.0	51.0	3.8	3.2	2.5
P77747	OmpN	Cell outer membrane	Monomer	99.0	83.7	64.6	6.8	5.8	4.5
			Complex with SecB	67.3	55.0	43.9	2.6	2.1	1.7
			Complex with TF (Tig)	73.1	61.8	47.7	3.2	2.7	2.1
P0AA16	OmpR	Cytoplasm	Monomer	116.3	98.2	75.9	10.0	8.4	6.5
P09169	OmpT	Cell outer membrane	Monomer	104.9	88.6	68.5	7.9	6.6	5.1
			Complex with SecB	68.7	56.1	44.8	2.7	2.2	1.8
			Complex with TF (Tig)	75.0	63.4	48.9	3.4	2.9	2.2
P0A915	OmpW	Cell outer membrane	Monomer	124.6	105.3	81.3	11.7	9.8	7.6
			Complex with SecB	72.3	59.0	47.2	3.1	2.5	2.0
			Complex with TF (Tig)	80.0	67.5	52.2	4.0	3.4	2.6
P0A917	OmpX	Cell outer membrane	Monomer	135.3	114.3	88.3	14.0	11.8	9.1
			Complex with SecB	73.6	60.2	48.1	3.3	2.7	2.1
			Complex with TF (Tig)	81.9	69.2	53.5	4.3	3.6	2.8
P23843	OppA	Periplasm	Monomer	85.0	71.8	55.4	4.7	4.0	3.1
			Complex with SecB	63.1	51.6	41.2	2.2	1.8	1.4
			Complex with TF (Tig)	67.6	57.1	44.1	2.6	2.2	1.7
P0AFH2	OppB	Cell inner membrane	Monomer	107.5	90.8	70.1	8.3	7.0	5.4

UniProtKB A. N.	Protein name	Subcellular location	Subunit	D_0 $\left(\frac{\mu m^2}{s}\right)$			D_{cyto} $\left(\frac{\mu m^2}{s}\right)$		
				310 K	303 K	293 K	310 K	303 K	293 K
			Complex with SecB	69.3	56.6	45.2	2.8	2.3	1.8
			Complex with TF (Tig)	75.7	64.0	49.4	3.5	3.0	2.3
P0AFH6	OppC	Cell inner membrane	Monomer	108.0	91.2	70.5	8.4	7.1	5.5
			Complex with SecB	69.4	56.7	45.3	2.8	2.3	1.8
			Complex with TF (Tig)	75.9	64.1	49.5	3.5	3.0	2.3
P76027	OppD	Cell inner membrane	Monomer	103.1	87.1	67.3	7.5	6.4	4.9
			Complex with SecB	68.3	55.8	44.6	2.7	2.2	1.7
			Complex with TF (Tig)	74.4	62.9	48.6	3.4	2.8	2.2
P77737	OppF	Cell inner membrane	Monomer	103.1	87.1	67.3	7.5	6.4	4.9
			Complex with SecB	68.3	55.8	44.6	2.7	2.2	1.7
			Complex with TF (Tig)	74.4	62.9	48.6	3.4	2.8	2.2
P0A784	Orn	Cytoplasm	Monomer	129.4	109.3	84.5	12.7	10.7	8.3
			Homodimer	98.6	80.6	64.4	6.8	5.5	4.4
P0ADA7	OsmB	Cell membrane	Monomer	199.0	168.1	129.8	31.2	26.3	20.3
			Complex with SecB	77.9	63.6	50.8	3.8	3.1	2.5
			Complex with TF (Tig)	88.3	74.6	57.6	5.2	4.4	3.4
P0C0L2	OsmC	Cytoplasm	Monomer	146.8	124.0	95.8	16.7	14.1	10.9
P0ADB1	OsmE	Cell membrane	Monomer	160.5	135.6	104.7	20.1	17.0	13.1
			Complex with SecB	75.9	62.0	49.6	3.5	2.9	2.3
			Complex with TF (Tig)	85.3	72.1	55.7	4.8	4.0	3.1
P33362	OsmF	Periplasm	Monomer	108.5	91.7	70.8	8.5	7.2	5.5
			Complex with SecB	69.5	56.8	45.3	2.8	2.3	1.8
			Complex with TF (Tig)	76.1	64.3	49.6	3.5	3.0	2.3
P0AFH8	OsmY	Periplasm	Monomer	128.8	108.8	84.1	12.5	10.6	8.2
			Complex with SecB	72.8	59.5	47.5	3.2	2.6	2.1
			Complex with TF (Tig)	80.8	68.3	52.7	4.1	3.5	2.7
P31677	OtsA		Monomer	89.3	75.5	58.3	5.3	4.5	3.5
			Homotetramer	51.9	42.4	33.9	1.2	1.0	0.8
P31678	OtsB		Monomer	113.4	95.8	74.0	9.4	7.9	6.1
P25714	OxaA	Cell inner membrane	Monomer	84.6	71.5	55.2	4.7	3.9	3.0
			Complex with SecB	63.0	51.5	41.1	2.1	1.8	1.4

UniProtKB A. N.	Protein name	Subcellular location	Subunit	D_0 $\left(\frac{\mu m^2}{s}\right)$			D_{cyto} $\left(\frac{\mu m^2}{s}\right)$		
				310 K	303 K	293 K	310 K	303 K	293 K
			Complex with TF (Tig)	67.5	57.0	44.0	2.6	2.2	1.7
P0AFI0	Oxc		Monomer	85.1	71.9	55.6	4.7	4.0	3.1
P0ACQ4	OxyR		Monomer	106.4	89.9	69.5	8.1	6.9	5.3
			Homodimer	81.1	66.3	52.9	4.2	3.4	2.7
			Homotetramer	61.8	52.2	40.3	2.0	1.7	1.3
P76077	PaaA		Monomer	105.0	88.7	68.5	7.9	6.6	5.1
P76078	PaaB		Monomer	166.5	140.7	108.7	21.7	18.4	14.2
P76079	PaaC		Monomer	115.4	97.5	75.3	9.8	8.3	6.4
P76080	PaaD		Monomer	136.1	114.9	88.8	14.2	12.0	9.2
P76081	PaaE		Monomer	100.9	85.2	65.8	7.2	6.0	4.7
P76082	PaaF		Monomer	116.5	98.4	76.0	10.0	8.5	6.5
P77467	PaaG		Monomer	114.6	96.8	74.8	9.6	8.1	6.3
P76083	PaaH		Monomer	90.6	76.5	59.1	5.5	4.7	3.6
P76084	PaaI		Monomer	147.7	124.8	96.4	16.9	14.3	11.0
			Homotetramer	85.8	70.1	56.0	4.8	3.9	3.1
P0C7L2	PaaJ		Monomer	98.0	82.8	64.0	6.7	5.6	4.4
P76085	PaaK		Monomer	92.6	78.2	60.4	5.8	4.9	3.8
P76086	PaaX		Monomer	105.2	88.9	68.7	7.9	6.7	5.2
P77181	PaaY		Monomer	128.2	108.3	83.7	12.4	10.5	8.1
P00903	PabA		Monomer	129.5	109.4	84.5	12.7	10.7	8.3
P05041	PabB		Monomer	91.1	77.0	59.5	5.6	4.7	3.7
P28305	PabC		Monomer	112.6	95.1	73.5	9.3	7.8	6.0
			Homodimer	85.8	70.1	56.0	4.8	3.9	3.1
P0A912	Pal	Cell outer membrane	Monomer	134.6	113.7	87.9	13.8	11.7	9.0
			Complex with SecB	73.6	60.1	48.0	3.3	2.7	2.1
			Complex with TF (Tig)	81.8	69.1	53.4	4.3	3.6	2.8
P31057	PanB	Cytoplasm	Monomer	114.8	97.0	74.9	9.7	8.2	6.3
			Homodecamer	46.6	38.0	30.4	0.9	0.7	0.6
P31663	PanC	Cytoplasm	Monomer	109.9	92.8	71.7	8.8	7.4	5.7
			Homodimer	83.7	68.4	54.6	4.5	3.7	3.0
P0A790	PanD	Cytoplasm	Monomer	151.9	128.3	99.1	17.9	15.2	11.7

UniProtKB A. N.	Protein name	Subcellular location	Subunit	D_0 $\left(\frac{\mu m^2}{s}\right)$			D_{cyto} $\left(\frac{\mu m^2}{s}\right)$		
				310 K	303 K	293 K	310 K	303 K	293 K
P0A9J4	PanE	Cytoplasm	Monomer	106.9	90.3	69.8	8.2	6.9	5.4
P16256	PanF	Cell inner membrane	Monomer	90.6	76.5	59.1	5.5	4.7	3.6
			Complex with SecB	65.0	53.1	42.4	2.3	1.9	1.5
			Complex with TF (Tig)	70.0	59.1	45.7	2.9	2.4	1.9
P0AFI2	ParC	Cell membrane	Monomer	75.0	63.3	48.9	3.4	2.9	2.2
			Complex with SecB	59.2	48.4	38.6	1.8	1.5	1.2
			Complex with TF (Tig)	62.7	53.0	40.9	2.1	1.8	1.4
P20083	ParE		Monomer	80.3	67.9	52.4	4.1	3.5	2.7
P42588	PatA		Monomer	92.0	77.8	60.1	5.7	4.8	3.7
Q46790	Pbl	Periplasm	Monomer	136.2	115.1	88.9	14.2	12.0	9.3
			Complex with SecB	73.8	60.3	48.1	3.3	2.7	2.1
			Complex with TF (Tig)	82.1	69.4	53.6	4.3	3.6	2.8
P76577	PbpC	Cell inner membrane	Monomer	74.5	63.0	48.6	3.4	2.8	2.2
			Complex with SecB	59.0	48.2	38.5	1.8	1.5	1.2
			Complex with TF (Tig)	62.5	52.8	40.8	2.1	1.8	1.4
P0AFI5	PbpG	Periplasm	Monomer	106.9	90.3	69.8	8.2	6.9	5.4
			Complex with SecB	69.2	56.5	45.1	2.8	2.3	1.8
			Complex with TF (Tig)	75.6	63.9	49.3	3.5	2.9	2.3
P22259	PckA	Cytoplasm	Monomer	85.7	72.4	55.9	4.8	4.1	3.1
P0A7A5	Pcm	Cytoplasm	Monomer	123.9	104.7	80.9	11.5	9.7	7.5
P0ABF1	PcnB		Monomer	89.2	75.3	58.2	5.3	4.5	3.5
P0ACL9	PdhR		Monomer	113.0	95.5	73.7	9.3	7.9	6.1
P19624	PdxA	Cytoplasm	Monomer	105.4	89.1	68.8	7.9	6.7	5.2
			Homodimer	80.4	65.6	52.4	4.1	3.3	2.7
P05459	PdxB	Cytoplasm	Monomer	98.9	83.5	64.5	6.8	5.8	4.5
			Homodimer	75.4	61.6	49.2	3.5	2.8	2.3
P0AFI7	PdxH		Monomer	119.4	100.9	77.9	10.6	9.0	6.9
			Homodimer	91.0	74.4	59.4	5.6	4.6	3.6
P0A794	PdxJ	Cytoplasm	Monomer	117.9	99.6	77.0	10.3	8.7	6.7
			Homooctamer	52.2	42.6	34.1	1.3	1.0	0.8
P40191	PdxK		Monomer	110.9	93.7	72.4	8.9	7.6	5.8

UniProtKB A. N.	Protein name	Subcellular location	Subunit	D_0 $\left(\frac{\mu m^2}{s}\right)$			D_{cyto} $\left(\frac{\mu m^2}{s}\right)$		
				310 K	303 K	293 K	310 K	303 K	293 K
P77150	PdxY		Monomer	110.3	93.2	72.0	8.8	7.5	5.8
			Homodimer	84.0	68.7	54.8	4.6	3.7	3.0
P68767	PepA		Monomer	88.5	74.8	57.8	5.2	4.4	3.4
			Homohexamer	43.8	35.8	28.6	0.7	0.6	0.5
P37095	PepB	Cytoplasm	Monomer	94.7	80.0	61.8	6.1	5.2	4.0
			Homohexamer	46.9	38.3	30.6	0.9	0.8	0.6
P15288	PepD		Monomer	89.8	75.8	58.6	5.4	4.6	3.5
P0A7C6	PepE	Cytoplasm	Monomer	121.3	102.5	79.1	11.0	9.3	7.2
P04825	PepN	Cell inner membrane	Monomer	70.3	59.4	45.8	2.9	2.4	1.9
P15034	PepP	Cytoplasm	Monomer	91.9	77.7	60.0	5.7	4.8	3.7
			Homotetramer	53.4	43.6	34.8	1.3	1.1	0.9
P21165	PepQ		Monomer	91.7	77.4	59.8	5.7	4.8	3.7
P29745	PepT	Cytoplasm	Monomer	95.7	80.9	62.5	6.3	5.3	4.1
			Homodimer	73.0	59.6	47.6	3.2	2.6	2.1
P0AFI9	PerM	Cell membrane	Monomer	101.0	85.3	65.9	7.2	6.1	4.7
			Complex with SecB	67.8	55.4	44.2	2.6	2.1	1.7
			Complex with TF (Tig)	73.8	62.3	48.1	3.3	2.8	2.1
Q57083	PerR		Monomer	107.2	90.6	70.0	8.3	7.0	5.4
P0A796	PfkA	Cytoplasm	Monomer	105.8	89.3	69.0	8.0	6.8	5.2
			Homotetramer	61.4	50.2	40.1	2.0	1.6	1.3
P06999	PfkB		Monomer	108.7	91.9	71.0	8.5	7.2	5.6
			Homodimer	82.9	67.7	54.1	4.4	3.6	2.9
P0A9N4	PflA	Cytoplasm	Monomer	114.9	97.1	75.0	9.7	8.2	6.3
P09373	PflB	Cytoplasm	Monomer	74.4	62.9	48.6	3.4	2.8	2.2
			Homodimer	56.7	46.3	37.0	1.6	1.3	1.0
P32675	PflC	Cytoplasm	Monomer	108.8	91.9	71.0	8.5	7.2	5.6
P32674	PflD	Cytoplasm	Monomer	74.2	62.7	48.4	3.3	2.8	2.2
P69434	PgaA	Cell outer membrane	Monomer	72.2	61.0	47.1	3.1	2.6	2.0
			Complex with SecB	58.0	47.4	37.8	1.7	1.4	1.1
			Complex with TF (Tig)	61.2	51.7	40.0	2.0	1.7	1.3
P75906	PgaB	Cell outer membrane	Monomer	77.3	65.3	50.5	3.7	3.1	2.4

UniProtKB A. N.	Protein name	Subcellular location	Subunit	D_0 $\left(\frac{\mu m^2}{s}\right)$ 310 K	D_0 303 K	D_0 293 K	D_{cyto} $\left(\frac{\mu m^2}{s}\right)$ 310 K	D_{cyto} 303 K	D_{cyto} 293 K
			Complex with SecB	60.2	49.2	39.3	1.9	1.5	1.2
			Complex with TF (Tig)	64.0	54.0	41.7	2.2	1.9	1.5
P75905	PgaC	Cell inner membrane	Monomer	91.3	77.1	59.5	5.6	4.7	3.7
			Complex with SecB	65.2	53.2	42.5	2.4	1.9	1.5
			Complex with TF (Tig)	70.2	59.3	45.8	2.9	2.4	1.9
P69432	PgaD	Cell inner membrane	Monomer	143.2	121.0	93.4	15.8	13.4	10.3
			Complex with SecB	74.5	60.9	48.6	3.4	2.7	2.2
			Complex with TF (Tig)	83.2	70.3	54.3	4.5	3.8	2.9
P0A6T1	Pgi	Cytoplasm	Monomer	84.6	71.5	55.2	4.7	3.9	3.0
			Homodimer	64.5	52.7	42.1	2.3	1.9	1.5
P0A799	Pgk	Cytoplasm	Monomer	99.1	83.7	64.7	6.9	5.8	4.5
P52697	Pgl		Monomer	104.1	87.9	67.9	7.7	6.5	5.0
P36938	Pgm		Monomer	86.4	73.0	56.4	4.9	4.1	3.2
P18200	PgpA	Cell inner membrane	Monomer	133.0	112.4	86.8	13.5	11.4	8.8
			Complex with SecB	73.4	59.9	47.9	3.2	2.6	2.1
			Complex with TF (Tig)	81.6	68.9	53.2	4.2	3.6	2.8
P0A924	PgpB	Cell inner membrane	Monomer	113.6	96.0	74.1	9.5	8.0	6.2
			Complex with SecB	70.5	57.6	46.0	2.9	2.4	1.9
			Complex with TF (Tig)	77.4	65.4	50.5	3.7	3.1	2.4
P0ABF8	PgsA	Cell inner membrane	Monomer	129.7	109.6	84.6	12.7	10.8	8.3
			Complex with SecB	73.0	59.6	47.6	3.2	2.6	2.1
			Complex with TF (Tig)	81.0	68.4	52.8	4.2	3.5	2.7
P0A9J8	PheA	Cytoplasm	Monomer	97.3	82.2	63.5	6.6	5.5	4.3
P0AD72	PheL		Monomer	329.2	278.1	214.8	79.2	66.9	51.7
P0AD74	PheM		Monomer	340.7	287.8	222.3	84.1	71.1	54.9
P24207	PheP	Cell inner membrane	Monomer	91.3	77.1	59.6	5.6	4.8	3.7
			Complex with SecB	65.2	53.2	42.5	2.4	1.9	1.5
			Complex with TF (Tig)	70.3	59.4	45.9	2.9	2.4	1.9
P08312	PheS	Cytoplasm	Monomer	103.5	87.4	67.5	7.6	6.4	5.0
P07395	PheT	Cytoplasm	Monomer	73.8	62.3	48.1	3.3	2.8	2.1
P0AFJ1	PhnA		Monomer	158.8	134.2	103.7	19.7	16.7	12.9

UniProtKB A. N.	Protein name	Subcellular location	Subunit	D_0 $\left(\frac{\mu m^2}{s}\right)$			D_{cyto} $\left(\frac{\mu m^2}{s}\right)$		
				310 K	303 K	293 K	310 K	303 K	293 K
P16681	PhnB		Monomer	142.9	120.7	93.2	15.7	13.3	10.3
P16677	PhnC	Cell inner membrane	Monomer	113.0	95.5	73.7	9.3	7.9	6.1
			Complex with SecB	70.4	57.5	45.9	2.9	2.4	1.9
			Complex with TF (Tig)	77.3	65.3	50.4	3.7	3.1	2.4
P16682	PhnD	Periplasm	Monomer	102.9	86.9	67.1	7.5	6.3	4.9
			Complex with SecB	68.3	55.8	44.5	2.7	2.2	1.7
			Complex with TF (Tig)	74.4	62.8	48.5	3.3	2.8	2.2
P16683	PhnE	Cell inner membrane	Monomer	114.6	96.8	74.8	9.6	8.1	6.3
			Complex with SecB	70.7	57.7	46.1	2.9	2.4	1.9
			Complex with TF (Tig)	77.7	65.6	50.7	3.7	3.2	2.4
P16684	PhnF		Monomer	115.8	97.9	75.6	9.9	8.3	6.4
P16685	PhnG		Monomer	141.6	119.7	92.4	15.4	13.0	10.1
P16686	PhnH		Monomer	128.9	108.9	84.1	12.6	10.6	8.2
P16687	PhnI		Monomer	101.3	85.6	66.1	7.2	6.1	4.7
P16688	PhnJ		Monomer	109.6	92.6	71.5	8.7	7.3	5.7
P16678	PhnK		Monomer	115.5	97.6	75.4	9.8	8.3	6.4
P16679	PhnL		Monomer	121.0	102.2	79.0	10.9	9.2	7.1
P16689	PhnM		Monomer	98.3	83.0	64.1	6.7	5.7	4.4
P16690	PhnN		Monomer	129.6	109.5	84.6	12.7	10.8	8.3
P16691	PhnO		Monomer	141.5	119.6	92.4	15.4	13.0	10.1
P16692	PhnP		Monomer	115.5	97.6	75.4	9.8	8.3	6.4
P00634	PhoA	Periplasm	Monomer	92.2	77.9	60.2	5.8	4.9	3.8
			Complex with SecB	65.4	53.5	42.7	2.4	1.9	1.6
			Complex with TF (Tig)	70.6	59.7	46.1	2.9	2.5	1.9
P0AFJ5	PhoB	Cytoplasm	Monomer	117.9	99.6	76.9	10.3	8.7	6.7
P02932	PhoE	Cell outer membrane	Monomer	101.3	85.6	66.1	7.2	6.1	4.7
			Complex with SecB	67.9	55.4	44.3	2.6	2.1	1.7
			Complex with TF (Tig)	73.8	62.4	48.2	3.3	2.8	2.1
P0A9K1	PhoH	Cytoplasm	Monomer	100.9	85.3	65.9	7.2	6.1	4.7
P23836	PhoP	Cytoplasm	Monomer	119.5	100.9	78.0	10.6	9.0	6.9
P23837	PhoQ	Cell inner membrane	Monomer	88.2	74.5	57.6	5.2	4.4	3.4

UniProtKB A. N.	Protein name	Subcellular location	Subunit	$D_0\left(\frac{\mu m^2}{s}\right)$			$D_{\text{cyto}}\left(\frac{\mu m^2}{s}\right)$		
				310 K	303 K	293 K	310 K	303 K	293 K
			Complex with SecB	64.2	52.5	41.9	2.3	1.8	1.5
			Complex with TF (Tig)	69.0	58.3	45.0	2.7	2.3	1.8
P08400	PhoR	Cell inner membrane	Monomer	92.1	77.8	60.1	5.7	4.8	3.7
			Complex with SecB	65.4	53.4	42.7	2.4	1.9	1.6
			Complex with TF (Tig)	70.6	59.6	46.0	2.9	2.5	1.9
P0A9K7	PhoU	Cytoplasm	Monomer	116.2	98.2	75.8	9.9	8.4	6.5
P45548	Php		Monomer	108.1	91.4	70.6	8.4	7.1	5.5
P00914	PhrB		Monomer	89.3	75.4	58.3	5.3	4.5	3.5
P03014	PinE		Monomer	130.0	109.8	84.8	12.8	10.8	8.4
P76611	PinH		Monomer	180.6	152.5	117.8	25.7	21.7	16.7
P77170	PinQ		Monomer	127.0	107.3	82.9	12.2	10.3	7.9
P0ADI0	PinR		Monomer	126.9	107.2	82.8	12.1	10.2	7.9
P03825	PioO		Monomer	143.9	121.6	93.9	16.0	13.5	10.4
P0AFJ7	PitA	Cell inner membrane	Monomer	89.5	75.6	58.4	5.3	4.5	3.5
			Complex with SecB	64.6	52.8	42.2	2.3	1.9	1.5
			Complex with TF (Tig)	69.5	58.7	45.4	2.8	2.4	1.8
P43676	PitB	Cell inner membrane	Monomer	89.2	75.4	58.2	5.3	4.5	3.5
			Complex with SecB	64.5	52.7	42.1	2.3	1.9	1.5
			Complex with TF (Tig)	69.4	58.6	45.3	2.8	2.4	1.8
P0A921	PldA	Cell outer membrane	Monomer	107.8	91.1	70.4	8.4	7.1	5.5
			Complex with SecB	69.3	56.6	45.3	2.8	2.3	1.8
			Complex with TF (Tig)	75.9	64.1	49.5	3.5	3.0	2.3
P07000	PldB	Cell inner membrane	Monomer	101.2	85.5	66.0	7.2	6.1	4.7
			Complex with SecB	67.9	55.4	44.3	2.6	2.1	1.7
			Complex with TF (Tig)	73.8	62.4	48.2	3.3	2.8	2.1
P0A7A7	PlsB	Cell inner membrane	Monomer	72.5	61.2	47.3	3.1	2.6	2.0
P26647	PlsC	Cell inner membrane	Monomer	116.1	98.1	75.8	9.9	8.4	6.5
			Complex with SecB	70.9	57.9	46.3	3.0	2.4	1.9
			Complex with TF (Tig)	78.0	65.9	50.9	3.8	3.2	2.5
P27247	PlsX	Cytoplasm	Monomer	102.0	86.2	66.6	7.3	6.2	4.8
			Homodimer	77.7	63.5	50.7	3.8	3.1	2.4

UniProtKB A. N.	Protein name	Subcellular location	Subunit	D_0 $\left(\frac{\mu m^2}{s}\right)$			D_{cyto} $\left(\frac{\mu m^2}{s}\right)$		
				310 K	303 K	293 K	310 K	303 K	293 K
P60782	PlsY	Cell inner membrane	Monomer	126.2	106.6	82.4	12.0	10.1	7.8
			Complex with SecB	72.5	59.2	47.3	3.1	2.6	2.0
			Complex with TF (Tig)	80.3	67.8	52.4	4.1	3.4	2.7
P0AFK0	PmbA	Cytoplasm	Monomer	93.0	78.6	60.7	5.9	5.0	3.8
P37590	PmrD		Monomer	173.4	146.5	113.2	23.6	20.0	15.4
P21369	PncA		Monomer	123.7	104.5	80.7	11.5	9.7	7.5
P18133	PncB	Cytoplasm	Monomer	94.9	80.2	61.9	6.2	5.2	4.0
P05055	Pnp	Cytoplasm	Monomer	77.5	65.4	50.5	3.7	3.1	2.4
			Homotrimer	50.4	41.1	32.9	1.1	0.9	0.7
P07001	PntA	Cell inner membrane	Monomer	88.7	74.9	57.9	5.2	4.4	3.4
			Complex with SecB	64.4	52.6	42.0	2.3	1.9	1.5
			Complex with TF (Tig)	69.2	58.5	45.2	2.8	2.3	1.8
P0AB67	PntB	Cell inner membrane	Monomer	92.7	78.3	60.5	5.8	4.9	3.8
			Complex with SecB	65.6	53.6	42.8	2.4	2.0	1.6
			Complex with TF (Tig)	70.8	59.8	46.2	2.9	2.5	1.9
P0AFK2	PnuC	Cell inner membrane	Monomer	116.9	98.7	76.3	10.1	8.5	6.6
			Complex with SecB	71.1	58.0	46.4	3.0	2.4	1.9
			Complex with TF (Tig)	78.2	66.1	51.0	3.8	3.2	2.5
P00582	PolA		Monomer	69.1	58.4	45.1	2.8	2.3	1.8
P21189	PolB		Monomer	72.9	61.6	47.6	3.2	2.7	2.1
P69874	PotA	Cell inner membrane	Monomer	97.4	82.3	63.5	6.6	5.6	4.3
			Complex with SecB	66.9	54.6	43.6	2.5	2.1	1.6
			Complex with TF (Tig)	72.5	61.3	47.3	3.1	2.6	2.0
P0AFK4	PotB	Cell inner membrane	Monomer	110.6	93.5	72.2	8.9	7.5	5.8
			Complex with SecB	69.9	57.1	45.6	2.8	2.3	1.9
			Complex with TF (Tig)	76.6	64.7	50.0	3.6	3.1	2.4
P0AFK6	PotC	Cell inner membrane	Monomer	113.5	95.9	74.1	9.4	8.0	6.2
			Complex with SecB	70.5	57.6	46.0	2.9	2.4	1.9
			Complex with TF (Tig)	77.4	65.4	50.5	3.7	3.1	2.4
P0AFK9	PotD	Periplasm	Monomer	101.3	85.6	66.1	7.2	6.1	4.7
			Complex with SecB	67.9	55.5	44.3	2.6	2.1	1.7

UniProtKB A. N.	Protein name	Subcellular location	Subunit	D_0 $\left(\frac{\mu m^2}{s}\right)$			D_{cyto} $\left(\frac{\mu m^2}{s}\right)$		
				310 K	303 K	293 K	310 K	303 K	293 K
			Complex with TF (Tig)	73.9	62.4	48.2	3.3	2.8	2.1
P0AAF1	PotE	Cell inner membrane	Monomer	94.5	79.8	61.6	6.1	5.2	4.0
			Complex with SecB	66.1	54.0	43.1	2.4	2.0	1.6
			Complex with TF (Tig)	71.5	60.4	46.6	3.0	2.5	2.0
P31133	PotF	Periplasm	Monomer	99.4	84.0	64.8	6.9	5.8	4.5
			Complex with SecB	67.4	55.1	44.0	2.6	2.1	1.7
			Complex with TF (Tig)	73.2	61.9	47.8	3.2	2.7	2.1
P31134	PotG	Cell inner membrane	Monomer	98.4	83.1	64.2	6.7	5.7	4.4
			Complex with SecB	67.1	54.9	43.8	2.6	2.1	1.7
			Complex with TF (Tig)	72.9	61.6	47.5	3.2	2.7	2.1
P31135	PotH	Cell inner membrane	Monomer	105.0	88.7	68.5	7.9	6.6	5.1
			Complex with SecB	68.7	56.2	44.9	2.7	2.2	1.8
			Complex with TF (Tig)	75.0	63.4	49.0	3.4	2.9	2.2
P0AFL1	PotI	Cell inner membrane	Monomer	111.4	94.1	72.7	9.0	7.6	5.9
			Complex with SecB	70.1	57.2	45.7	2.9	2.3	1.9
			Complex with TF (Tig)	76.8	64.9	50.1	3.6	3.1	2.4
P0A8N7	PoxA		Monomer	103.3	87.3	67.4	7.6	6.4	4.9
P07003	PoxB	Cell membrane	Monomer	84.4	71.3	55.1	4.6	3.9	3.0
			Complex with SecB	62.9	51.4	41.1	2.1	1.7	1.4
			Complex with TF (Tig)	67.3	56.9	43.9	2.6	2.2	1.7
P0A7A9	Ppa	Cytoplasm	Monomer	132.2	111.7	86.3	13.3	11.2	8.7
			Homohexamer	65.5	53.5	42.8	2.4	2.0	1.6
P00864	Ppc		Monomer	70.2	59.3	45.8	2.9	2.4	1.9
			Homotetramer	40.8	33.3	26.6	0.6	0.5	0.4
P33554	PpdA		Monomer	137.4	116.1	89.7	14.5	12.2	9.4
P08371	PpdB		Monomer	130.2	110.0	84.9	12.8	10.8	8.4
P08372	PpdC		Monomer	160.6	135.7	104.8	20.2	17.0	13.2
P36647	PpdD	Fimbrium	Monomer	144.8	122.4	94.5	16.2	13.7	10.6
			Complex with SecB	74.7	61.0	48.7	3.4	2.8	2.2
			Complex with TF (Tig)	83.4	70.5	54.4	4.5	3.8	2.9
P55798	PphA		Monomer	119.9	101.3	78.3	10.7	9.0	7.0

UniProtKB A. N.	Protein name	Subcellular location	Subunit	D_0 $\left(\frac{\mu m^2}{s}\right)$ 310 K	303 K	293 K	D_{cyto} $\left(\frac{\mu m^2}{s}\right)$ 310 K	303 K	293 K
P55799	PphB		Monomer	120.3	101.6	78.5	10.8	9.1	7.0
P0AFL3	PpiA	Periplasm	Monomer	130.4	110.1	85.1	12.9	10.9	8.4
			Complex with SecB	73.1	59.7	47.7	3.2	2.6	2.1
			Complex with TF (Tig)	81.1	68.5	52.9	4.2	3.5	2.7
P23869	PpiB	Cytoplasm	Monomer	136.6	115.4	89.1	14.3	12.1	9.3
P0A9L5	PpiC	Cytoplasm	Monomer	171.0	144.4	111.6	23.0	19.4	15.0
P0ADY1	PpiD	Cell inner membrane	Monomer	81.3	68.7	53.1	4.2	3.6	2.7
			Complex with SecB	61.8	50.5	40.3	2.0	1.7	1.3
			Complex with TF (Tig)	65.9	55.7	43.0	2.4	2.1	1.6
P0A7B1	Ppk	Cell inner membrane	Monomer	76.2	64.4	49.7	3.6	3.0	2.3
			Complex with SecB	59.7	48.8	39.0	1.9	1.5	1.2
			Complex with TF (Tig)	63.4	53.5	41.4	2.2	1.8	1.4
P0A7B3	PpnK	Cytoplasm	Monomer	108.6	91.7	70.9	8.5	7.2	5.6
Q46836	PppA	Cell inner membrane	Monomer	112.9	95.4	73.7	9.3	7.9	6.1
			Complex with SecB	70.4	57.5	45.9	2.9	2.4	1.9
			Complex with TF (Tig)	77.2	65.3	50.4	3.7	3.1	2.4
P23538	PpsA		Monomer	73.7	62.3	48.1	3.3	2.8	2.1
			Homodimer	56.2	45.9	36.7	1.6	1.3	1.0
P31992	PptA	Cytoplasm	Monomer	182.4	154.1	119.0	26.2	22.1	17.1
			Homodimer	139.0	113.6	90.7	14.8	12.1	9.7
P0AFL6	Ppx	Cell membrane	Monomer	86.5	73.1	56.5	4.9	4.2	3.2
			Complex with SecB	63.7	52.0	41.5	2.2	1.8	1.4
			Complex with TF (Tig)	68.3	57.7	44.6	2.7	2.3	1.7
P0AFL9	PqiA	Cell inner membrane	Monomer	94.5	79.8	61.7	6.1	5.2	4.0
			Complex with SecB	66.1	54.0	43.1	2.4	2.0	1.6
			Complex with TF (Tig)	71.5	60.4	46.6	3.0	2.5	2.0
P43671	PqiB	Cell membrane	Monomer	85.2	72.0	55.6	4.7	4.0	3.1
			Complex with SecB	63.2	51.6	41.2	2.2	1.8	1.4
			Complex with TF (Tig)	67.7	57.2	44.2	2.6	2.2	1.7
P31828	PqqL		Monomer	68.7	58.1	44.8	2.7	2.3	1.8
P23865	Prc	Cell inner membrane	Monomer	77.6	65.6	50.7	3.7	3.2	2.4

UniProtKB A. N.	Protein name	Subcellular location	Subunit	D_0 $\left(\frac{\mu m^2}{s}\right)$ 310 K	303 K	293 K	D_{cyto} $\left(\frac{\mu m^2}{s}\right)$ 310 K	303 K	293 K
			Complex with SecB	60.3	49.3	39.4	1.9	1.6	1.2
			Complex with TF (Tig)	64.1	54.2	41.8	2.3	1.9	1.5
P0A7I0	PrfA	Cytoplasm	Monomer	99.7	84.2	65.1	7.0	5.9	4.5
P07012	PrfB	Cytoplasm	Monomer	99.0	83.6	64.6	6.8	5.8	4.5
P0A7I4	PrfC	Cytoplasm	Monomer	85.7	72.4	55.9	4.8	4.1	3.1
P28369	PrfH		Monomer	134.6	113.7	87.8	13.8	11.7	9.0
P17888	PriA		Monomer	75.7	64.0	49.4	3.5	3.0	2.3
P07013	PriB		Monomer	163.6	138.2	106.8	21.0	17.7	13.7
P23862	PriC		Monomer	130.5	110.3	85.2	12.9	10.9	8.4
P0AEX5	PrkB		Monomer	108.9	92.0	71.1	8.6	7.2	5.6
P27298	PrlC		Monomer	77.4	65.4	50.5	3.7	3.1	2.4
P0A8T1	PrmA	Cytoplasm	Monomer	109.5	92.5	71.5	8.7	7.3	5.7
P07004	ProA	Cytoplasm	Monomer	96.0	81.1	62.6	6.4	5.4	4.1
P0A7B5	ProB	Cytoplasm	Monomer	101.1	85.4	66.0	7.2	6.1	4.7
P0A9L8	ProC	Cytoplasm	Monomer	115.0	97.1	75.0	9.7	8.2	6.3
P0C0L7	ProP	Cell inner membrane	Monomer	88.5	74.8	57.8	5.2	4.4	3.4
			Complex with SecB	64.3	52.5	42.0	2.3	1.9	1.5
			Complex with TF (Tig)	69.1	58.4	45.1	2.8	2.3	1.8
P45577	ProQ	Cytoplasm	Monomer	118.8	100.4	77.5	10.5	8.8	6.8
P16659	ProS	Cytoplasm	Monomer	83.5	70.5	54.5	4.5	3.8	2.9
			Homodimer	63.6	52.0	41.5	2.2	1.8	1.4
P14175	ProV	Cell inner membrane	Monomer	96.4	81.4	62.9	6.4	5.4	4.2
			Complex with SecB	66.6	54.4	43.5	2.5	2.0	1.6
			Complex with TF (Tig)	72.2	61.0	47.1	3.1	2.6	2.0
P14176	ProW	Cell inner membrane	Monomer	102.6	86.7	67.0	7.5	6.3	4.9
			Complex with SecB	68.2	55.7	44.5	2.7	2.2	1.7
			Complex with TF (Tig)	74.3	62.8	48.5	3.3	2.8	2.2
P0AFM2	ProX	Periplasm	Monomer	104.4	88.2	68.1	7.8	6.6	5.1
			Complex with SecB	68.6	56.0	44.8	2.7	2.2	1.8
			Complex with TF (Tig)	74.8	63.2	48.8	3.4	2.9	2.2
P0AAE2	ProY	Cell inner membrane	Monomer	91.6	77.4	59.8	5.7	4.8	3.7

UniProtKB A. N.	Protein name	Subcellular location	Subunit	D_0 $\left(\frac{\mu m^2}{s}\right)$			D_{cyto} $\left(\frac{\mu m^2}{s}\right)$		
				310 K	303 K	293 K	310 K	303 K	293 K
			Complex with SecB	65.3	53.3	42.6	2.4	1.9	1.5
			Complex with TF (Tig)	70.4	59.5	45.9	2.9	2.4	1.9
P77541	PrpB		Monomer	109.2	92.2	71.2	8.6	7.3	5.6
P31660	PrpC		Monomer	97.3	82.2	63.5	6.6	5.5	4.3
			Homodimer	74.1	60.6	48.4	3.3	2.7	2.2
P77243	PrpD		Monomer	89.1	75.3	58.1	5.3	4.5	3.5
P77495	PrpE		Monomer	80.7	68.2	52.7	4.1	3.5	2.7
P77743	PrpR		Monomer	86.2	72.9	56.3	4.9	4.1	3.2
P77674	Prr		Monomer	91.2	77.1	59.5	5.6	4.7	3.7
			Homotetramer	53.0	43.3	34.6	1.3	1.1	0.9
P0A717	Prs	Cytoplasm	Monomer	106.5	90.0	69.5	8.1	6.9	5.3
P0A8K1	Psd	Cell membrane	Monomer	104.5	88.3	68.2	7.8	6.6	5.1
			Complex with SecB	68.6	56.1	44.8	2.7	2.2	1.8
			Complex with TF (Tig)	74.9	63.2	48.9	3.4	2.9	2.2
P36678	PshM	Cell inner membrane	Monomer	139.4	117.7	90.9	14.9	12.6	9.7
			Complex with SecB	74.1	60.5	48.4	3.3	2.7	2.2
			Complex with TF (Tig)	82.6	69.8	53.9	4.4	3.7	2.9
P0A7C8	PsiE	Cell inner membrane	Monomer	144.9	122.4	94.6	16.2	13.7	10.6
			Complex with SecB	74.7	61.0	48.7	3.4	2.8	2.2
			Complex with TF (Tig)	83.4	70.5	54.4	4.5	3.8	2.9
P0AFM4	PsiF		Monomer	162.3	137.1	105.9	20.6	17.4	13.5
P0AFM6	PspA	Cytoplasm. Cell inner membrane	Monomer	119.5	101.0	78.0	10.6	9.0	6.9
P0AFM9	PspB	Cell inner membrane	Monomer	181.7	153.5	118.6	26.0	21.9	17.0
			Complex with SecB	77.2	63.0	50.4	3.7	3.0	2.4
			Complex with TF (Tig)	87.2	73.7	56.9	5.0	4.2	3.3
P0AFN2	PspC	Cell inner membrane	Monomer	153.3	129.5	100.0	18.3	15.5	11.9
			Complex with SecB	75.4	61.6	49.2	3.5	2.8	2.3
			Complex with TF (Tig)	84.5	71.4	55.2	4.6	3.9	3.0
P0AFV8	PspD	Cytoplasm. Cell inner membrane	Monomer	187.9	158.7	122.6	27.8	23.5	18.1

UniProtKB A. N.	Protein name	Subcellular location	Subunit	$D_0 \left(\frac{\mu m^2}{s}\right)$			$D_{\text{cyto}} \left(\frac{\mu m^2}{s}\right)$		
				310 K	303 K	293 K	310 K	303 K	293 K
P23857	PspE	Periplasm	Monomer	163.5	138.1	106.7	20.9	17.7	13.7
			Complex with SecB	76.1	62.2	49.7	3.6	2.9	2.3
			Complex with TF (Tig)	85.7	72.4	55.9	4.8	4.1	3.1
P37344	PspF	Cytoplasm	Monomer	103.3	87.3	67.4	7.6	6.4	4.9
P32696	PspG	Cell inner membrane	Monomer	179.6	151.7	117.2	25.4	21.4	16.6
			Complex with SecB	77.1	63.0	50.3	3.7	3.0	2.4
			Complex with TF (Tig)	87.1	73.6	56.8	5.0	4.2	3.3
P23830	PssA	Cytoplasm. Cell inner membrane	Monomer	89.9	75.9	58.6	5.4	4.6	3.5
P07654	PstA	Cell inner membrane	Monomer	108.9	92.0	71.1	8.6	7.2	5.6
			Complex with SecB	69.6	56.8	45.4	2.8	2.3	1.8
			Complex with TF (Tig)	76.2	64.3	49.7	3.6	3.0	2.3
P0AAH0	PstB	Cell inner membrane	Monomer	113.6	96.0	74.1	9.5	8.0	6.2
			Complex with SecB	70.5	57.6	46.0	2.9	2.4	1.9
			Complex with TF (Tig)	77.4	65.4	50.5	3.7	3.1	2.4
P0AGH8	PstC	Cell inner membrane	Monomer	106.6	90.1	69.6	8.2	6.9	5.3
			Complex with SecB	69.1	56.4	45.1	2.8	2.3	1.8
			Complex with TF (Tig)	75.5	63.8	49.3	3.5	2.9	2.3
P0AG82	PstS	Periplasm	Monomer	103.3	87.2	67.4	7.6	6.4	4.9
			Complex with SecB	68.3	55.8	44.6	2.7	2.2	1.7
			Complex with TF (Tig)	74.5	62.9	48.6	3.4	2.8	2.2
P33025	PsuG		Monomer	108.2	91.4	70.6	8.4	7.1	5.5
P30235	PsuK		Monomer	107.3	90.7	70.0	8.3	7.0	5.4
P33024	PsuT	Cell inner membrane	Monomer	97.0	82.0	63.3	6.5	5.5	4.3
			Complex with SecB	66.8	54.6	43.6	2.5	2.1	1.6
			Complex with TF (Tig)	72.4	61.2	47.2	3.1	2.6	2.0
P0A9M8	Pta	Cytoplasm	Monomer	77.4	65.4	50.5	3.7	3.1	2.4
			Homohexamer	38.4	31.3	25.0	0.5	0.4	0.3
P0A7D1	Pth	Cytoplasm	Monomer	128.8	108.8	84.0	12.5	10.6	8.2
P05458	PtrA	Periplasm	Monomer	68.0	57.4	44.3	2.6	2.2	1.7
			Complex with SecB	56.0	45.7	36.5	1.5	1.3	1.0

UniProtKB A. N.	Protein name	Subcellular location	Subunit	D_0 $\left(\frac{\mu m^2}{s}\right)$			D_{cyto} $\left(\frac{\mu m^2}{s}\right)$		
				310 K	303 K	293 K	310 K	303 K	293 K
			Complex with TF (Tig)	58.8	49.7	38.4	1.8	1.5	1.2
P24555	PtrB		Monomer	76.5	64.7	49.9	3.6	3.0	2.4
P32670	PtsA	Cytoplasm	Monomer	72.4	61.1	47.2	3.1	2.6	2.0
P69786	PtsG	Cell inner membrane	Monomer	91.3	77.1	59.6	5.6	4.8	3.7
			Complex with SecB	65.2	53.2	42.5	2.4	1.9	1.5
			Complex with TF (Tig)	70.3	59.4	45.9	2.9	2.4	1.9
P0AA04	PtsH	Cytoplasm	Monomer	178.9	151.1	116.7	25.2	21.3	16.4
P08839	PtsI	Cytoplasm	Monomer	83.6	70.6	54.5	4.5	3.8	2.9
			Homodimer	63.7	52.0	41.6	2.2	1.8	1.4
P69829	PtsN	Cytoplasm	Monomer	137.1	115.9	89.5	14.4	12.2	9.4
P0A9N0	PtsO	Cytoplasm	Monomer	173.8	146.8	113.4	23.7	20.1	15.5
P37177	PtsP	Cytoplasm	Monomer	75.0	63.4	48.9	3.4	2.9	2.2
P0A7D4	PurA	Cytoplasm	Monomer	93.8	79.2	61.2	6.0	5.1	3.9
			Homodimer	71.5	58.4	46.6	3.0	2.5	2.0
P0AB89	PurB		Monomer	90.7	76.6	59.2	5.5	4.7	3.6
P0A7D7	PurC		Monomer	116.9	98.7	76.3	10.1	8.5	6.6
			Homotrimer	76.0	62.1	49.6	3.5	2.9	2.3
P15640	PurD		Monomer	94.9	80.2	61.9	6.2	5.2	4.0
P0AG18	PurE		Monomer	137.7	116.3	89.8	14.5	12.3	9.5
			Homooctamer	60.9	49.8	39.8	2.0	1.6	1.3
P0AG16	PurF		Monomer	87.5	73.9	57.1	5.1	4.3	3.3
			Homotetramer	50.8	41.5	33.2	1.2	1.0	0.8
P15639	PurH		Monomer	87.0	73.5	56.8	5.0	4.2	3.3
P09029	PurK		Monomer	100.7	85.1	65.7	7.1	6.0	4.7
			Homodimer	76.8	62.7	50.1	3.6	3.0	2.4
P15254	PurL	Cytoplasm	Monomer	61.1	51.6	39.9	2.0	1.7	1.3
P08178	PurM	Cytoplasm	Monomer	103.5	87.4	67.5	7.6	6.4	5.0
			Homodimer	78.8	64.4	51.4	3.9	3.2	2.5
P08179	PurN		Monomer	124.0	104.7	80.9	11.5	9.7	7.5
			Homodimer	94.5	77.2	61.6	6.1	5.0	4.0
P31466	PurP	Cell inner membrane	Monomer	94.2	79.5	61.4	6.1	5.1	4.0

UniProtKB A. N.	Protein name	Subcellular location	Subunit	D_0 $\left(\frac{\mu m^2}{s}\right)$			D_{cyto} $\left(\frac{\mu m^2}{s}\right)$		
				310 K	303 K	293 K	310 K	303 K	293 K
			Complex with SecB	66.0	53.9	43.1	2.4	2.0	1.6
			Complex with TF (Tig)	71.4	60.3	46.6	3.0	2.5	2.0
P0ACP7	PurR		Monomer	102.0	86.2	66.6	7.4	6.2	4.8
			Homodimer	77.8	63.5	50.7	3.8	3.1	2.5
P33221	PurT		Monomer	97.9	82.7	63.9	6.7	5.6	4.3
			Homodimer	74.6	60.9	48.7	3.4	2.8	2.2
P37051	PurU		Monomer	109.4	92.5	71.4	8.7	7.3	5.7
			Homohexamer	54.2	44.3	35.4	1.4	1.1	0.9
P09546	PutA		Monomer	60.7	51.3	39.6	1.9	1.6	1.3
			Homodimer	46.2	37.8	30.2	0.9	0.7	0.6
P07117	PutP	Cell inner membrane	Monomer	88.8	75.1	58.0	5.3	4.4	3.4
			Complex with SecB	64.4	52.6	42.0	2.3	1.9	1.5
			Complex with TF (Tig)	69.3	58.5	45.2	2.8	2.3	1.8
P78061	PuuA		Monomer	89.6	75.7	58.5	5.4	4.5	3.5
P37906	PuuB		Monomer	93.9	79.3	61.3	6.0	5.1	3.9
P23883	PuuC		Monomer	89.4	75.6	58.4	5.3	4.5	3.5
P76038	PuuD		Monomer	115.2	97.3	75.2	9.8	8.2	6.4
			Homodimer	87.8	71.7	57.3	5.1	4.2	3.3
P50457	PuuE		Monomer	95.9	81.0	62.6	6.3	5.4	4.1
P76037	PuuP	Cell inner membrane	Monomer	91.2	77.0	59.5	5.6	4.7	3.7
			Complex with SecB	65.1	53.2	42.5	2.4	1.9	1.5
			Complex with TF (Tig)	70.2	59.3	45.8	2.9	2.4	1.9
P0A9U6	PuuR		Monomer	131.2	110.9	85.6	13.1	11.0	8.5
P21599	PykA		Monomer	90.8	76.7	59.3	5.6	4.7	3.6
			Homotetramer	52.8	43.1	34.4	1.3	1.1	0.8
P0AD61	PykF		Monomer	91.3	77.1	59.6	5.6	4.7	3.7
			Homotetramer	53.0	43.3	34.6	1.3	1.1	0.9
P0A786	PyrB		Monomer	106.3	89.8	69.3	8.1	6.8	5.3
			Heterododecamer ($PyrB_6-PyrI_6$)	44.9	38.0	29.3	0.8	0.7	0.5
P05020	PyrC		Monomer	101.4	85.6	66.1	7.2	6.1	4.7
			Homodimer	77.2	63.1	50.4	3.7	3.0	2.4

UniProtKB A. N.	Protein name	Subcellular location	Subunit	D_0 $\left(\frac{\mu m^2}{s}\right)$			D_{cyto} $\left(\frac{\mu m^2}{s}\right)$		
				310 K	303 K	293 K	310 K	303 K	293 K
P0A7E1	PyrD	Cell membrane	Monomer	103.5	87.5	67.6	7.6	6.4	5.0
			Complex with SecB	68.4	55.9	44.6	2.7	2.2	1.8
			Complex with TF (Tig)	74.6	63.0	48.7	3.4	2.8	2.2
P0A7E3	PyrE		Monomer	123.3	104.1	80.4	11.4	9.6	7.4
			Homodimer	93.9	76.7	61.3	6.0	4.9	3.9
P08244	PyrF		Monomer	118.0	99.7	77.0	10.3	8.7	6.7
			Homodimer	89.9	73.5	58.7	5.4	4.4	3.5
P0A7E5	PyrG		Monomer	85.3	72.0	55.6	4.7	4.0	3.1
			Homodimer	65.0	53.1	42.4	2.3	1.9	1.5
			Homotetramer	49.5	41.8	32.3	1.1	0.9	0.7
P0A7E9	PyrH	Cytoplasm	Monomer	118.7	100.3	77.4	10.4	8.8	6.8
			Homohexamer	58.8	48.0	38.4	1.8	1.4	1.2
P0A7F3	PyrI		Monomer	139.7	118.0	91.2	15.0	12.7	9.8
			Heterododecamer ($PyrB_6-PyrI_6$)	44.9	38.0	29.3	0.8	0.7	0.5
P0AD83	PyrL		Monomer	224.7	189.8	146.6	39.4	33.3	25.7
P0AA53	QmcA	Cell inner membrane	Monomer	107.1	90.5	69.9	8.2	7.0	5.4
			Complex with SecB	69.2	56.5	45.2	2.8	2.3	1.8
			Complex with TF (Tig)	75.6	63.9	49.4	3.5	3.0	2.3
P28304	QorA		Monomer	105.4	89.0	68.8	7.9	6.7	5.2
			Homodimer	80.3	65.6	52.4	4.1	3.3	2.7
P39315	QorB		Monomer	112.5	95.1	73.4	9.2	7.8	6.0
P52076	QseB	Cytoplasm	Monomer	121.1	102.3	79.0	10.9	9.2	7.1
P40719	QseC	Cell inner membrane	Monomer	91.6	77.4	59.8	5.7	4.8	3.7
			Complex with SecB	65.3	53.3	42.6	2.4	1.9	1.5
			Complex with TF (Tig)	70.4	59.5	45.9	2.9	2.4	1.9
P39376	QseD		Monomer	105.9	89.5	69.1	8.0	6.8	5.2
P0A7F9	QueA	Cytoplasm	Monomer	100.8	85.1	65.7	7.1	6.0	4.7
P77756	QueC		Monomer	119.5	101.0	78.0	10.6	9.0	6.9
P65870	QueD		Monomer	152.2	128.6	99.3	18.0	15.2	11.8
Q46920	QueF	Cytoplasm	Monomer	108.6	91.7	70.8	8.5	7.2	5.6
			Homodimer	82.7	67.6	54.0	4.4	3.6	2.9

UniProtKB A. N.	Protein name	Subcellular location	Subunit	D_0 $\left(\frac{\mu m^2}{s}\right)$			D_{cyto} $\left(\frac{\mu m^2}{s}\right)$		
				310 K	303 K	293 K	310 K	303 K	293 K
Q47274	QuuD		Monomer	150.5	127.1	98.2	17.6	14.9	11.5
P76161	QuuQ		Monomer	115.3	97.4	75.3	9.8	8.3	6.4
P15033	RacC		Monomer	172.4	145.7	112.5	23.4	19.7	15.2
P76062	RacR		Monomer	138.0	116.6	90.1	14.6	12.3	9.5
P24554	RadA		Monomer	92.2	77.9	60.2	5.8	4.9	3.8
P0AD49	RaiA		Monomer	156.7	132.4	102.2	19.2	16.2	12.5
P60240	RapA		Monomer	67.4	57.0	44.0	2.6	2.2	1.7
P0AAZ4	RarA		Monomer	92.1	77.8	60.1	5.7	4.8	3.7
P27844	RarD	Cell inner membrane	Monomer	107.7	91.0	70.3	8.3	7.1	5.4
			Complex with SecB	69.3	56.6	45.2	2.8	2.3	1.8
			Complex with TF (Tig)	75.8	64.0	49.5	3.5	3.0	2.3
P0AGL5	RatA		Monomer	137.8	116.4	89.9	14.6	12.3	9.5
P52119	RatB		Monomer	167.5	141.5	109.3	22.0	18.6	14.4
P31473	RavA	Cytoplasm	Monomer	87.6	74.0	57.1	5.1	4.3	3.3
P0A7G2	RbfA	Cytoplasm	Monomer	146.6	123.8	95.6	16.6	14.0	10.9
P0A8V0	Rbn		Monomer	108.1	91.3	70.6	8.4	7.1	5.5
			Homodimer	82.4	67.3	53.8	4.4	3.6	2.8
P04983	RbsA	Cell inner membrane	Monomer	88.4	74.7	57.7	5.2	4.4	3.4
			Complex with SecB	64.3	52.5	41.9	2.3	1.9	1.5
			Complex with TF (Tig)	69.1	58.4	45.1	2.8	2.3	1.8
P02925	RbsB	Periplasm	Monomer	110.8	93.6	72.3	8.9	7.5	5.8
			Complex with SecB	69.9	57.1	45.6	2.8	2.3	1.9
			Complex with TF (Tig)	76.7	64.8	50.0	3.6	3.1	2.4
P0AGI1	RbsC	Cell inner membrane	Monomer	107.5	90.8	70.1	8.3	7.0	5.4
			Complex with SecB	69.3	56.6	45.2	2.8	2.3	1.8
			Complex with TF (Tig)	75.7	64.0	49.4	3.5	3.0	2.3
P04982	RbsD	Cytoplasm	Monomer	146.1	123.4	95.3	16.5	13.9	10.8
			Homodecamer	59.2	48.4	38.6	1.8	1.5	1.2
P0A9J6	RbsK	Cytoplasm	Monomer	109.0	92.1	71.1	8.6	7.2	5.6
			Homodimer	83.0	67.8	54.2	4.4	3.6	2.9
P0ACQ0	RbsR		Monomer	103.7	87.6	67.7	7.6	6.5	5.0

UniProtKB A. N.	Protein name	Subcellular location	Subunit	D_0 $\left(\frac{\mu m^2}{s}\right)$			D_{cyto} $\left(\frac{\mu m^2}{s}\right)$		
				310 K	303 K	293 K	310 K	303 K	293 K
P76425	RcnA	Cell inner membrane	Monomer	111.5	94.2	72.8	9.1	7.7	5.9
			Complex with SecB	70.1	57.3	45.7	2.9	2.3	1.9
			Complex with TF (Tig)	76.9	64.9	50.2	3.6	3.1	2.4
P64534	RcnB	Periplasm	Monomer	158.2	133.7	103.3	19.6	16.5	12.8
			Complex with SecB	75.8	61.9	49.4	3.5	2.9	2.3
			Complex with TF (Tig)	85.1	71.9	55.5	4.7	4.0	3.1
P64530	RcnR	Cytoplasm	Monomer	171.6	145.0	112.0	23.1	19.5	15.1
P69405	RcsA		Monomer	123.4	104.2	80.5	11.4	9.6	7.4
P69407	RcsB		Monomer	123.1	104.0	80.3	11.3	9.6	7.4
			Homodimer	93.8	76.6	61.2	6.0	4.9	3.9
P14376	RcsC	Cell inner membrane	Monomer	68.2	57.7	44.5	2.7	2.3	1.7
			Complex with SecB	56.1	45.8	36.6	1.6	1.3	1.0
			Complex with TF (Tig)	59.0	49.8	38.5	1.8	1.5	1.2
P39838	RcsD	Cell inner membrane	Monomer	69.9	59.0	45.6	2.8	2.4	1.9
			Complex with SecB	56.9	46.5	37.1	1.6	1.3	1.1
			Complex with TF (Tig)	59.9	50.6	39.1	1.9	1.6	1.2
P69411	RcsF	Cell outer membrane	Monomer	150.5	127.2	98.2	17.6	14.9	11.5
			Complex with SecB	75.2	61.4	49.0	3.4	2.8	2.2
			Complex with TF (Tig)	84.2	71.1	54.9	4.6	3.9	3.0
P52061	RdgB		Monomer	128.9	108.9	84.1	12.6	10.6	8.2
			Homodimer	98.2	80.2	64.1	6.7	5.5	4.4
P36767	RdgC	Cytoplasm > nucleoid	Monomer	106.8	90.2	69.7	8.2	6.9	5.3
P0C0K3	RdoA	Cytoplasm	Monomer	102.1	86.3	66.6	7.4	6.2	4.8
P0A7G6	RecA	Cytoplasm	Monomer	102.3	86.4	66.7	7.4	6.2	4.8
P08394	RecB		Monomer	62.4	52.7	40.7	2.1	1.8	1.4
P07648	RecC		Monomer	63.3	53.5	41.3	2.2	1.8	1.4
P04993	RecD		Monomer	81.9	69.2	53.4	4.3	3.6	2.8
P15032	RecE		Monomer	71.0	60.0	46.3	3.0	2.5	1.9
			Homotetramer	41.2	33.7	26.9	0.6	0.5	0.4
P0A7H0	RecF	Cytoplasm	Monomer	99.7	84.2	65.1	7.0	5.9	4.5

UniProtKB A. N.	Protein name	Subcellular location	Subunit	D_0 $\left(\frac{\mu m^2}{s}\right)$			D_{cyto} $\left(\frac{\mu m^2}{s}\right)$		
				310 K	303 K	293 K	310 K	303 K	293 K
P24230	RecG		Monomer	77.7	65.7	50.7	3.8	3.2	2.4
P21893	RecJ		Monomer	83.6	70.7	54.6	4.5	3.8	3.0
P05824	RecN		Monomer	84.7	71.6	55.3	4.7	3.9	3.0
P0A7H3	RecO		Monomer	116.2	98.2	75.8	10.0	8.4	6.5
P15043	RecQ		Monomer	81.2	68.6	53.0	4.2	3.5	2.7
P0A7H6	RecR		Monomer	126.7	107.1	82.7	12.1	10.2	7.9
P33228	RecT		Monomer	112.6	95.1	73.5	9.3	7.8	6.0
			Homotetramer	65.4	53.4	42.7	2.4	1.9	1.5
P33596	RecX	Cytoplasm	Monomer	133.0	112.3	86.8	13.5	11.4	8.8
P0AG20	RelA		Monomer	74.9	63.3	48.9	3.4	2.9	2.2
P0C079	RelB		Monomer	179.2	151.4	117.0	25.3	21.4	16.5
			Heterotetramer ($RelE_2-RelB_2$)	99.6	84.2	65.0	6.9	5.9	4.5
P0C077	RelE		Monomer	164.9	139.3	107.6	21.3	18.0	13.9
			Heterotetramer ($RelE_2-RelB_2$)	99.6	84.2	65.0	6.9	5.9	4.5
P07010	Rem		Monomer	178.0	150.4	116.1	24.9	21.1	16.3
P75718	RenD		Monomer	165.2	139.6	107.8	21.4	18.1	14.0
P09980	Rep		Monomer	77.5	65.5	50.6	3.7	3.1	2.4
P27127	RfaB		Monomer	99.4	84.0	64.9	6.9	5.8	4.5
P24173	RfaC		Monomer	104.9	88.7	68.5	7.9	6.6	5.1
P37692	RfaF		Monomer	101.1	85.5	66.0	7.2	6.1	4.7
P25740	RfaG		Monomer	98.0	82.8	64.0	6.7	5.6	4.4
P0AFW0	RfaH		Monomer	136.0	114.9	88.8	14.1	12.0	9.2
P27128	RfaI		Monomer	100.8	85.1	65.8	7.1	6.0	4.7
P27129	RfaJ		Monomer	101.1	85.5	66.0	7.2	6.1	4.7
P27243	RfaL		Monomer	94.1	79.5	61.4	6.1	5.1	4.0
		Cell inner membrane	Complex with SecB	66.0	53.9	43.1	2.4	2.0	1.6
			Complex with TF (Tig)	71.4	60.3	46.6	3.0	2.5	2.0
P25741	RfaP		Monomer	110.9	93.7	72.4	8.9	7.6	5.8
P25742	RfaQ		Monomer	101.5	85.7	66.2	7.3	6.1	4.7
P27126	RfaS		Monomer	103.6	87.5	67.6	7.6	6.4	5.0
P27240	RfaY		Monomer	116.1	98.1	75.8	9.9	8.4	6.5

UniProtKB A. N.	Protein name	Subcellular location	Subunit	D_0 $\left(\frac{\mu m^2}{s}\right)$			D_{cyto} $\left(\frac{\mu m^2}{s}\right)$		
				310 K	303 K	293 K	310 K	303 K	293 K
P27241	RfaZ		Monomer	108.1	91.4	70.6	8.4	7.1	5.5
P37759	RfbB		Monomer	99.6	84.2	65.0	6.9	5.9	4.5
			Homodimer	75.9	62.0	49.6	3.5	2.9	2.3
P37745	RfbC		Monomer	128.3	108.4	83.7	12.4	10.5	8.1
			Homodimer	97.8	79.9	63.8	6.6	5.4	4.3
P37760	RfbD		Monomer	108.4	91.6	70.8	8.5	7.2	5.5
			Homodimer	82.6	67.5	53.9	4.4	3.6	2.9
P37746	RfbX	Cell inner membrane	Monomer	95.4	80.6	62.2	6.3	5.3	4.1
			Complex with SecB	66.3	54.2	43.3	2.5	2.0	1.6
			Complex with TF (Tig)	71.8	60.7	46.9	3.1	2.6	2.0
P37748	Rfc	Cell inner membrane	Monomer	95.9	81.0	62.6	6.3	5.4	4.1
			Complex with SecB	66.5	54.3	43.4	2.5	2.0	1.6
			Complex with TF (Tig)	72.0	60.8	47.0	3.1	2.6	2.0
P27833	RffA		Monomer	98.4	83.1	64.2	6.7	5.7	4.4
P27832	RffC		Monomer	122.0	103.0	79.6	11.1	9.4	7.2
P27830	RffG		Monomer	100.4	84.8	65.5	7.1	6.0	4.6
			Homodimer	76.5	62.5	49.9	3.6	2.9	2.4
P32170	RhaA	Cytoplasm	Monomer	93.9	79.3	61.3	6.0	5.1	3.9
			Homotetramer	54.5	44.5	35.6	1.4	1.2	0.9
P32171	RhaB		Monomer	89.0	75.2	58.1	5.3	4.5	3.4
P32169	RhaD	Cytoplasm	Monomer	111.9	94.6	73.0	9.1	7.7	6.0
			Homotetramer	65.0	53.1	42.4	2.3	1.9	1.5
P32156	RhaM	Cytoplasm	Monomer	159.2	134.5	103.9	19.8	16.7	12.9
			Homodimer	121.4	99.1	79.2	11.0	9.0	7.2
P09378	RhaR	Cytoplasm	Monomer	108.9	92.0	71.0	8.6	7.2	5.6
P09377	RhaS	Cytoplasm	Monomer	108.9	92.0	71.1	8.6	7.2	5.6
P27125	RhaT	Cell inner membrane	Monomer	103.0	87.0	67.2	7.5	6.3	4.9
			Complex with SecB	68.3	55.8	44.5	2.7	2.2	1.7
			Complex with TF (Tig)	74.4	62.8	48.5	3.3	2.8	2.2
P0A8J8	RhlB		Monomer	94.0	79.4	61.3	6.0	5.1	3.9
P25888	RhlE		Monomer	91.8	77.6	59.9	5.7	4.8	3.7

UniProtKB A. N.	Protein name	Subcellular location	Subunit	D_0 $\left(\frac{\mu m^2}{s}\right)$			D_{cyto} $\left(\frac{\mu m^2}{s}\right)$		
				310 K	303 K	293 K	310 K	303 K	293 K
P76469	RhmA		Monomer	113.8	96.1	74.2	9.5	8.0	6.2
			Homohexamer	56.4	46.0	36.8	1.6	1.3	1.0
P77215	RhmD		Monomer	96.3	81.4	62.9	6.4	5.4	4.2
			Homooctamer	42.6	34.8	27.8	0.7	0.6	0.4
P77732	RhmR		Monomer	113.8	96.1	74.3	9.5	8.0	6.2
P76470	RhmT	Cell inner membrane	Monomer	94.6	79.9	61.7	6.1	5.2	4.0
			Complex with SecB	66.1	54.0	43.2	2.4	2.0	1.6
			Complex with TF (Tig)	71.5	60.4	46.7	3.0	2.6	2.0
P0AG30	Rho		Monomer	94.0	79.5	61.4	6.0	5.1	3.9
			Homohexamer	46.6	38.1	30.4	0.9	0.7	0.6
P0ADF3	RhoL		Monomer	253.4	214.1	165.4	49.5	41.8	32.3
P16916	RhsA		Monomer	58.7	49.6	38.3	1.8	1.5	1.2
P16917	RhsB		Monomer	58.3	49.2	38.0	1.7	1.5	1.1
P16918	RhsC		Monomer	58.5	49.4	38.2	1.7	1.5	1.1
P16919	RhsD		Monomer	58.2	49.2	38.0	1.7	1.5	1.1
P24211	RhsE		Monomer	77.4	65.4	50.5	3.7	3.1	2.4
P0AA67	RhtA	Cell inner membrane	Monomer	110.5	93.3	72.1	8.9	7.5	5.8
			Complex with SecB	69.9	57.1	45.6	2.8	2.3	1.9
			Complex with TF (Tig)	76.6	64.7	50.0	3.6	3.1	2.4
P0AG34	RhtB	Cell membrane	Monomer	125.7	106.2	82.0	11.9	10.0	7.8
			Complex with SecB	72.4	59.2	47.3	3.1	2.6	2.0
			Complex with TF (Tig)	80.2	67.7	52.3	4.1	3.4	2.7
P0AG38	RhtC	Cell inner membrane	Monomer	125.6	106.1	82.0	11.9	10.0	7.7
			Complex with SecB	72.4	59.2	47.3	3.1	2.5	2.0
			Complex with TF (Tig)	80.2	67.7	52.3	4.1	3.4	2.6
P0A7I7	RibA		Monomer	127.0	107.3	82.9	12.2	10.3	7.9
			Homodimer	96.8	79.1	63.2	6.5	5.3	4.2
P0A7J0	RibB	Cell membrane	Monomer	123.7	104.5	80.7	11.5	9.7	7.5
			Complex with SecB	72.1	58.9	47.1	3.1	2.5	2.0
			Complex with TF (Tig)	79.8	67.4	52.1	4.0	3.4	2.6
P25539	RibD		Monomer	99.9	84.4	65.2	7.0	5.9	4.6

UniProtKB A. N.	Protein name	Subcellular location	Subunit	$D_0\ \left(\frac{\mu m^2}{s}\right)$			$D_{\text{cyto}}\ \left(\frac{\mu m^2}{s}\right)$		
				310 K	303 K	293 K	310 K	303 K	293 K
			Homodimer	76.1	62.2	49.7	3.6	2.9	2.3
P0AFU8	RibE		Monomer	123.5	104.4	80.6	11.4	9.7	7.5
P0AG40	RibF		Monomer	105.9	89.5	69.1	8.0	6.8	5.2
P61714	RibH		Monomer	142.9	120.8	93.3	15.8	13.3	10.3
P41409	RihA		Monomer	107.0	90.4	69.8	8.2	6.9	5.4
P33022	RihB		Monomer	107.1	90.5	69.9	8.2	7.0	5.4
			Homotetramer	62.2	50.8	40.6	2.1	1.7	1.4
P22564	RihC		Monomer	108.6	91.8	70.9	8.5	7.2	5.6
P0A944	RimI	Cytoplasm	Monomer	141.4	119.5	92.3	15.4	13.0	10.0
P0A948	RimJ	Cytoplasm	Monomer	125.1	105.7	81.7	11.8	9.9	7.7
P0C0U4	RimK		Monomer	108.8	91.9	71.0	8.5	7.2	5.6
P13857	RimL	Cytoplasm	Monomer	129.8	109.6	84.7	12.8	10.8	8.3
P0A7X6	RimM	Cytoplasm	Monomer	129.9	109.8	84.8	12.8	10.8	8.3
P45748	RimN	Cytoplasm	Monomer	129.5	109.4	84.5	12.7	10.7	8.3
P0AEI4	RimO	Cytoplasm	Monomer	92.1	77.8	60.1	5.7	4.9	3.7
P0A8A8	RimP	Cytoplasm	Monomer	141.3	119.3	92.2	15.4	13.0	10.0
P36999	RlmA		Monomer	111.5	94.2	72.8	9.1	7.7	5.9
			Homodimer	85.0	69.4	55.5	4.7	3.8	3.1
P63177	RlmB	Cytoplasm	Monomer	117.6	99.4	76.8	10.2	8.6	6.7
			Homodimer	89.7	73.2	58.5	5.4	4.4	3.5
P0C0R7	RlmE	Cytoplasm	Monomer	123.8	104.6	80.8	11.5	9.7	7.5
P75782	RlmF	Cytoplasm	Monomer	106.5	90.0	69.5	8.1	6.9	5.3
P42596	RlmG	Cytoplasm	Monomer	98.0	82.8	63.9	6.7	5.6	4.4
P0A8I8	RlmH	Cytoplasm	Monomer	139.0	117.5	90.7	14.8	12.5	9.7
			Homodimer	106.0	86.6	69.1	8.0	6.6	5.2
P75876	RlmI	Cytoplasm	Monomer	96.2	81.3	62.8	6.4	5.4	4.2
			Homodimer	73.3	59.9	47.8	3.2	2.6	2.1
P75864	RlmL	Cytoplasm	Monomer	76.8	64.9	50.1	3.6	3.1	2.4
			Homodimer	58.5	47.8	38.2	1.7	1.4	1.1
P0ADR6	RlmM	Cytoplasm	Monomer	98.4	83.1	64.2	6.7	5.7	4.4
P36979	RlmN	Cytoplasm	Monomer	97.3	82.2	63.5	6.6	5.5	4.3

UniProtKB A. N.	Protein name	Subcellular location	Subunit	D_0 $\left(\frac{\mu m^2}{s}\right)$			D_{cyto} $\left(\frac{\mu m^2}{s}\right)$		
				310 K	303 K	293 K	310 K	303 K	293 K
P10100	RlpA	Cell membrane	Monomer	102.7	86.8	67.0	7.5	6.3	4.9
			Complex with SecB	68.2	55.7	44.5	2.7	2.2	1.7
			Complex with TF (Tig)	74.3	62.8	48.5	3.3	2.8	2.2
P0AA37	RluA		Monomer	120.7	102.0	78.8	10.9	9.2	7.1
P37765	RluB		Monomer	108.4	91.6	70.7	8.5	7.2	5.5
P0AA39	RluC		Monomer	104.4	88.2	68.1	7.8	6.6	5.1
P33643	RluD		Monomer	103.2	87.2	67.3	7.5	6.4	4.9
P75966	RluE		Monomer	120.7	102.0	78.8	10.8	9.2	7.1
P32684	RluF		Monomer	108.7	91.8	70.9	8.5	7.2	5.6
P0AFW2	Rmf		Monomer	204.2	172.5	133.2	32.8	27.7	21.4
P37744	RmlA1		Monomer	108.4	91.6	70.8	8.5	7.2	5.5
			Homotetramer	63.0	51.4	41.1	2.1	1.8	1.4
P61887	RmlA2		Monomer	108.4	91.6	70.7	8.5	7.2	5.5
			Homotetramer	62.9	51.4	41.1	2.1	1.7	1.4
P0AG71	RmuC		Monomer	88.6	74.8	57.8	5.2	4.4	3.4
P21338	Rna	Periplasm<br>Cytoplasm	Monomer	112.7	95.2	73.6	9.3	7.8	6.1
P30850	Rnb	Cytoplasm	Monomer	79.4	67.0	51.8	4.0	3.3	2.6
P0A7Y0	Rnc	Cytoplasm	Monomer	119.4	100.9	77.9	10.6	8.9	6.9
P09155	Rnd	Cytoplasm	Monomer	97.6	82.5	63.7	6.6	5.6	4.3
P21513	Rne	Cytoplasm	Monomer	65.5	55.4	42.8	2.4	2.0	1.6
P0A766	RnfA	Cell inner membrane	Monomer	129.2	109.2	84.3	12.6	10.7	8.2
			Complex with SecB	72.9	59.6	47.6	3.2	2.6	2.1
			Complex with TF (Tig)	80.9	68.3	52.8	4.2	3.5	2.7
P77223	RnfB	Cytoplasm. Cell<br>inner membrane	Monomer	130.1	109.9	84.9	12.8	10.8	8.4
P77611	RnfC	Cell inner membrane	Monomer	76.3	64.4	49.8	3.6	3.0	2.3
			Complex with SecB	59.8	48.8	39.0	1.9	1.5	1.2
			Complex with TF (Tig)	63.4	53.6	41.4	2.2	1.8	1.4
P76182	RnfD	Cell inner membrane	Monomer	102.1	86.2	66.6	7.4	6.2	4.8
			Complex with SecB	68.1	55.6	44.4	2.6	2.2	1.7

UniProtKB A. N.	Protein name	Subcellular location	Subunit	D_0 $\left(\frac{\mu m^2}{s}\right)$			D_{cyto} $\left(\frac{\mu m^2}{s}\right)$		
				310 K	303 K	293 K	310 K	303 K	293 K
			Complex with TF (Tig)	74.1	62.6	48.4	3.3	2.8	2.2
P77179	RnfE	Cell inner membrane	Monomer	121.5	102.6	79.3	11.0	9.3	7.2
			Complex with SecB	71.8	58.7	46.9	3.1	2.5	2.0
			Complex with TF (Tig)	79.3	67.0	51.7	3.9	3.3	2.6
P77285	RnfG	Cell inner membrane	Monomer	126.8	107.2	82.8	12.1	10.2	7.9
			Complex with SecB	72.6	59.3	47.4	3.1	2.6	2.0
			Complex with TF (Tig)	80.4	67.9	52.5	4.1	3.5	2.7
P0A9J0	Rng	Cytoplasm > cytoskeleton	Monomer	88.2	74.5	57.6	5.2	4.4	3.4
P0A7Y4	RnhA	Cytoplasm	Monomer	138.2	116.8	90.2	14.7	12.4	9.6
P10442	RnhB	Cytoplasm	Monomer	127.7	107.9	83.4	12.3	10.4	8.0
P0AFW4	Rnk		Monomer	147.4	124.6	96.2	16.8	14.2	11.0
P0A7Y8	RnpA		Monomer	152.1	128.5	99.3	18.0	15.2	11.7
P21499	Rnr	Cytoplasm	Monomer	72.2	61.0	47.1	3.1	2.6	2.0
P30014	Rnt		Monomer	123.4	104.2	80.5	11.4	9.6	7.4
			Homodimer	94.0	76.8	61.4	6.0	4.9	3.9
P0ACI0	Rob		Monomer	107.9	91.1	70.4	8.4	7.1	5.5
P27434	RodZ	Cell inner membrane	Monomer	104.2	88.0	68.0	7.7	6.5	5.0
			Complex with SecB	68.6	56.0	44.7	2.7	2.2	1.8
			Complex with TF (Tig)	74.8	63.2	48.8	3.4	2.9	2.2
P0AFW8	Rof		Monomer	176.2	148.8	115.0	24.4	20.6	15.9
P0AG07	Rpe		Monomer	121.3	102.5	79.2	11.0	9.3	7.2
P0CG19	Rph		Monomer	121.6	102.7	79.3	11.0	9.3	7.2
			Homodimer	92.6	75.7	60.5	5.8	4.8	3.8
P0A7Z0	RpiA		Monomer	124.8	105.4	81.4	11.7	9.9	7.6
			Homodimer	95.1	77.7	62.0	6.2	5.1	4.1
P37351	RpiB		Monomer	143.2	121.0	93.5	15.8	13.4	10.3
			Homodimer	109.2	89.2	71.2	8.6	7.0	5.6
P0ACS7	RpiR		Monomer	108.9	92.0	71.0	8.6	7.2	5.6
P0A7L0	RplA		Monomer	121.0	102.2	78.9	10.9	9.2	7.1
P60422	RplB		Monomer	112.4	94.9	73.3	9.2	7.8	6.0

UniProtKB A. N.	Protein name	Subcellular location	Subunit	D_0 $\left(\frac{\mu m^2}{s}\right)$			D_{cyto} $\left(\frac{\mu m^2}{s}\right)$		
				310 K	303 K	293 K	310 K	303 K	293 K
P60438	RplC		Monomer	126.1	106.5	82.3	12.0	10.1	7.8
P60723	RplD		Monomer	126.5	106.8	82.5	12.0	10.2	7.9
P62399	RplE		Monomer	130.7	110.4	85.3	13.0	10.9	8.5
P0AG55	RplF		Monomer	134.4	113.5	87.7	13.8	11.6	9.0
P0A7R1	RplI		Monomer	144.3	121.9	94.2	16.1	13.6	10.5
P0A7J3	RplJ		Monomer	137.9	116.5	90.0	14.6	12.3	9.5
P0A7J7	RplK		Monomer	147.6	124.7	96.3	16.9	14.3	11.0
P0A7K2	RplL		Monomer	159.1	134.4	103.8	19.8	16.7	12.9
P0AA10	RplM		Monomer	143.4	121.2	93.6	15.9	13.4	10.4
P0ADY3	RplN		Monomer	153.2	129.4	100.0	18.3	15.4	11.9
P02413	RplO		Monomer	147.2	124.4	96.1	16.8	14.2	11.0
P0ADY7	RplP		Monomer	146.1	123.4	95.3	16.5	13.9	10.8
P0AG44	RplQ		Monomer	149.7	126.5	97.7	17.4	14.7	11.3
P0C018	RplR		Monomer	156.7	132.4	102.3	19.2	16.2	12.5
P0A7K6	RplS		Monomer	155.0	131.0	101.2	18.7	15.8	12.2
P0A7L3	RplT		Monomer	153.4	129.6	100.1	18.3	15.5	12.0
P0AG48	RplU		Monomer	163.0	137.7	106.3	20.8	17.6	13.6
P61175	RplV		Monomer	159.4	134.7	104.0	19.9	16.8	13.0
P0ADZ0	RplW		Monomer	165.0	139.4	107.7	21.3	18.0	13.9
P60624	RplX		Monomer	164.4	138.9	107.3	21.2	17.9	13.8
P68919	RplY		Monomer	168.0	142.0	109.7	22.2	18.7	14.5
P0A7L8	RpmA		Monomer	178.8	151.1	116.7	25.2	21.3	16.4
P0A7M2	RpmB		Monomer	179.7	151.9	117.3	25.4	21.5	16.6
P0A7M6	RpmC		Monomer	195.5	165.1	127.5	30.1	25.4	19.6
P0AG51	RpmD		Monomer	203.7	172.1	133.0	32.6	27.6	21.3
P0A7M9	RpmE		Monomer	189.5	160.1	123.7	28.3	23.9	18.4
P0A7N1	RpmE2		Monomer	173.1	146.2	112.9	23.5	19.9	15.4
P0A7N4	RpmF		Monomer	204.9	173.1	133.7	33.0	27.9	21.5
P0A7N9	RpmG		Monomer	205.9	173.9	134.3	33.3	28.1	21.7
P0A7P5	RpmH		Monomer	220.0	185.8	143.5	37.9	32.0	24.7
P0A7Q1	RpmI		Monomer	195.3	165.0	127.4	30.0	25.4	19.6

UniProtKB A. N.	Protein name	Subcellular location	Subunit	D_0 $\left(\frac{\mu m^2}{s}\right)$			D_{cyto} $\left(\frac{\mu m^2}{s}\right)$		
				310 K	303 K	293 K	310 K	303 K	293 K
P0A7Q6	RpmJ		Monomer	238.8	201.7	155.8	44.3	37.4	28.9
Q2EEQ2	RpmJ2		Monomer	218.6	184.7	142.6	37.4	31.6	24.4
P0A7Z4	RpoA		Monomer	103.8	87.7	67.8	7.7	6.5	5.0
			Homodimer	79.1	64.6	51.6	3.9	3.2	2.6
P0A8V2	RpoB		Monomer	59.6	50.3	38.9	1.8	1.6	1.2
P0A8T7	RpoC		Monomer	58.9	49.8	38.4	1.8	1.5	1.2
P00579	RpoD		Monomer	80.3	67.9	52.4	4.1	3.4	2.7
P0AGB6	RpoE		Monomer	127.3	107.6	83.1	12.2	10.3	8.0
P0AGB3	RpoH		Monomer	108.7	91.9	70.9	8.5	7.2	5.6
P24255	RpoN		Monomer	89.1	75.3	58.1	5.3	4.5	3.5
P13445	RpoS		Monomer	102.3	86.4	66.7	7.4	6.2	4.8
P0A800	RpoZ		Monomer	170.9	144.4	111.5	22.9	19.4	15.0
P0A776	RppH		Monomer	129.5	109.4	84.5	12.7	10.7	8.3
P0AG67	RpsA		Monomer	84.8	71.7	55.4	4.7	4.0	3.1
P0A7V0	RpsB		Monomer	117.3	99.1	76.6	10.2	8.6	6.6
P0A7V3	RpsC		Monomer	118.7	100.2	77.4	10.4	8.8	6.8
P0A7V8	RpsD		Monomer	123.5	104.3	80.6	11.4	9.6	7.5
P0A7W1	RpsE		Monomer	138.2	116.8	90.2	14.7	12.4	9.6
P02358	RpsF		Monomer	144.5	122.1	94.3	16.1	13.6	10.5
P02359	RpsG		Monomer	131.4	111.0	85.8	13.1	11.1	8.6
P0A7W7	RpsH		Monomer	150.7	127.3	98.3	17.6	14.9	11.5
P0A7X3	RpsI		Monomer	147.7	124.8	96.4	16.9	14.3	11.0
P0A7R5	RpsJ		Monomer	162.0	136.9	105.7	20.5	17.4	13.4
P0A7R9	RpsK		Monomer	151.9	128.3	99.1	17.9	15.1	11.7
P0A7S3	RpsL		Monomer	152.3	128.7	99.4	18.0	15.2	11.8
P0A7S9	RpsM		Monomer	155.2	131.1	101.3	18.8	15.9	12.2
P0AG59	RpsN		Monomer	162.9	137.6	106.3	20.8	17.5	13.6
P0ADZ4	RpsO		Monomer	170.7	144.2	111.4	22.9	19.3	14.9
P0A7T3	RpsP		Monomer	178.3	150.6	116.4	25.0	21.1	16.3
P0AG63	RpsQ		Monomer	174.6	147.5	113.9	24.0	20.2	15.6
P0A7T7	RpsR		Monomer	179.9	152.0	117.4	25.5	21.5	16.6

UniProtKB A. N.	Protein name	Subcellular location	Subunit	D_0 $\left(\frac{\mu m^2}{s}\right)$			D_{cyto} $\left(\frac{\mu m^2}{s}\right)$		
				310 K	303 K	293 K	310 K	303 K	293 K
P0A7U3	RpsS		Monomer	169.7	143.4	110.7	22.6	19.1	14.8
P0A7U7	RpsT		Monomer	174.7	147.6	114.0	24.0	20.3	15.7
P68679	RpsU		Monomer	183.9	155.3	120.0	26.6	22.5	17.4
P0A8R0	RraA	Cytoplasm	Monomer	139.0	117.4	90.7	14.8	12.5	9.7
			Homotrimer	90.3	73.8	59.0	5.5	4.5	3.6
P0AF90	RraB	Cytoplasm	Monomer	144.9	122.4	94.6	16.2	13.7	10.6
P0AFX4	Rsd	Cytoplasm	Monomer	136.3	115.1	88.9	14.2	12.0	9.3
P0AFX7	RseA	Cell membrane	Monomer	121.8	102.9	79.5	11.1	9.3	7.2
			Complex with SecB	71.8	58.7	46.9	3.1	2.5	2.0
			Complex with TF (Tig)	79.4	67.0	51.8	4.0	3.3	2.6
P0AFX9	RseB	Periplasm	Monomer	104.7	88.5	68.3	7.8	6.6	5.1
			Complex with SecB	68.7	56.1	44.8	2.7	2.2	1.8
			Complex with TF (Tig)	74.9	63.3	48.9	3.4	2.9	2.2
P46187	RseC	Cell inner membrane	Monomer	141.3	119.4	92.2	15.4	13.0	10.0
			Complex with SecB	74.3	60.7	48.5	3.3	2.7	2.2
			Complex with TF (Tig)	82.9	70.0	54.1	4.4	3.7	2.9
P0AEH1	RseP	Cell inner membrane	Monomer	92.5	78.1	60.3	5.8	4.9	3.8
			Complex with SecB	65.5	53.5	42.8	2.4	2.0	1.6
			Complex with TF (Tig)	70.7	59.7	46.1	2.9	2.5	1.9
P39286	RsgA		Monomer	101.0	85.3	65.9	7.2	6.1	4.7
P06992	RsmA	Cytoplasm	Monomer	111.5	94.2	72.8	9.1	7.7	5.9
P36929	RsmB	Cytoplasm	Monomer	93.0	78.6	60.7	5.9	5.0	3.8
P39406	RsmC	Cytoplasm	Monomer	102.6	86.7	67.0	7.5	6.3	4.9
P0ADX9	RsmD		Monomer	127.4	107.6	83.1	12.2	10.3	8.0
P0AGL7	RsmE	Cytoplasm	Monomer	116.9	98.8	76.3	10.1	8.5	6.6
			Homodimer	89.1	72.8	58.1	5.3	4.3	3.5
P76273	RsmF	Cytoplasm	Monomer	89.6	75.7	58.5	5.4	4.5	3.5
P0A6U5	RsmG	Cytoplasm	Monomer	123.6	104.4	80.6	11.4	9.7	7.5
P60390	RsmH	Cytoplasm	Monomer	105.7	89.3	69.0	8.0	6.8	5.2
P67087	RsmI	Cytoplasm	Monomer	110.2	93.1	71.9	8.8	7.4	5.8
P38104	RspA		Monomer	94.9	80.2	61.9	6.2	5.2	4.0

UniProtKB A. N.	Protein name	Subcellular location	Subunit	D_0 $\left(\frac{\mu m^2}{s}\right)$			D_{cyto} $\left(\frac{\mu m^2}{s}\right)$		
				310 K	303 K	293 K	310 K	303 K	293 K
P38105	RspB		Monomer	103.8	87.7	67.7	7.7	6.5	5.0
P0AFR0	RssA		Monomer	108.0	91.2	70.4	8.4	7.1	5.5
P52108	RstA	Cytoplasm	Monomer	117.4	99.2	76.6	10.2	8.6	6.6
P18392	RstB	Cell inner membrane	Monomer	92.3	78.0	60.2	5.8	4.9	3.8
			Complex with SecB	65.5	53.5	42.7	2.4	1.9	1.6
			Complex with TF (Tig)	70.7	59.7	46.1	2.9	2.5	1.9
P0AA43	RsuA		Monomer	118.9	100.4	77.6	10.5	8.9	6.8
P46849	RtcA	Cytoplasm	Monomer	104.5	88.3	68.2	7.8	6.6	5.1
P46850	RtcB		Monomer	95.5	80.7	62.3	6.3	5.3	4.1
P38035	RtcR		Monomer	85.3	72.1	55.7	4.8	4.0	3.1
P76446	Rtn	Cell inner membrane	Monomer	86.3	72.9	56.3	4.9	4.1	3.2
			Complex with SecB	63.6	51.9	41.5	2.2	1.8	1.4
			Complex with TF (Tig)	68.2	57.6	44.5	2.7	2.2	1.7
P55135	RumA		Monomer	93.2	78.8	60.8	5.9	5.0	3.9
P75817	RumB		Monomer	98.3	83.1	64.2	6.7	5.7	4.4
P0AG74	RusA		Monomer	151.9	128.3	99.1	17.9	15.1	11.7
			Homodimer	115.7	94.5	75.5	9.9	8.1	6.4
P75898	RutA		Monomer	98.1	82.9	64.0	6.7	5.7	4.4
P75897	RutB		Monomer	120.1	101.4	78.3	10.7	9.1	7.0
P0AFQ5	RutC		Monomer	152.2	128.6	99.3	18.0	15.2	11.8
			Homotrimer	99.0	80.8	64.6	6.8	5.6	4.5
P75895	RutD		Monomer	113.8	96.1	74.3	9.5	8.0	6.2
P75894	RutE		Monomer	127.6	107.8	83.3	12.3	10.4	8.0
P75893	RutF		Monomer	137.8	116.4	89.9	14.5	12.3	9.5
P75892	RutG	Cell inner membrane	Monomer	95.2	80.4	62.1	6.2	5.3	4.1
			Complex with SecB	66.3	54.2	43.3	2.5	2.0	1.6
			Complex with TF (Tig)	71.7	60.6	46.8	3.0	2.6	2.0
P0ACU2	RutR		Monomer	123.0	103.9	80.3	11.3	9.6	7.4
			Homodimer	93.8	76.6	61.2	6.0	4.9	3.9
P0A809	RuvA		Monomer	126.5	106.8	82.5	12.0	10.2	7.9
			Homotetramer	73.4	60.0	47.9	3.2	2.6	2.1

UniProtKB A. N.	Protein name	Subcellular location	Subunit	D_0 $\left(\frac{\mu m^2}{s}\right)$			D_{cyto} $\left(\frac{\mu m^2}{s}\right)$		
				310 K	303 K	293 K	310 K	303 K	293 K
P0A812	RuvB		Monomer	103.1	87.1	67.3	7.5	6.4	4.9
			Homododecamer	38.9	31.8	25.4	0.5	0.4	0.3
P0A814	RuvC		Monomer	134.8	113.9	88.0	13.9	11.7	9.1
			Homodimer	102.8	84.0	67.1	7.5	6.1	4.9
P58041	RzoD	Cell outer membrane	Monomer	202.0	170.6	131.8	32.1	27.1	20.9
			Complex with SecB	78.0	63.7	50.9	3.8	3.1	2.5
			Complex with TF (Tig)	88.5	74.8	57.8	5.2	4.4	3.4
C1P601	RzoQ	Cell membrane	Monomer	176.3	148.9	115.0	24.4	20.6	15.9
			Complex with SecB	76.9	62.8	50.2	3.6	3.0	2.4
			Complex with TF (Tig)	86.8	73.3	56.6	5.0	4.2	3.2
P58042	RzoR	Cell outer membrane	Monomer	201.1	169.9	131.2	31.8	26.9	20.8
			Complex with SecB	78.0	63.7	50.9	3.8	3.1	2.5
			Complex with TF (Tig)	88.5	74.7	57.7	5.2	4.4	3.4
P75719	RzpD		Monomer	139.4	117.8	91.0	14.9	12.6	9.7
P76158	RzpQ		Monomer	136.1	115.0	88.8	14.2	12.0	9.2
P77551	RzpR		Monomer	141.8	119.8	92.5	15.5	13.1	10.1
P76149	Sad		Monomer	92.0	77.7	60.0	5.7	4.8	3.7
			Homodimer	70.1	57.3	45.8	2.9	2.3	1.9
P76136	SafA	Cell inner membrane	Monomer	195.5	165.1	127.5	30.1	25.4	19.6
			Complex with SecB	77.8	63.5	50.7	3.8	3.1	2.5
			Complex with TF (Tig)	88.1	74.5	57.5	5.2	4.4	3.4
P0AFY2	SanA	Cell inner membrane	Monomer	116.4	98.3	76.0	10.0	8.4	6.5
			Complex with SecB	71.0	58.0	46.3	3.0	2.4	1.9
			Complex with TF (Tig)	78.1	66.0	51.0	3.8	3.2	2.5
Q47622	SapA	Periplasm	Monomer	84.6	71.5	55.2	4.7	3.9	3.0
			Complex with SecB	63.0	51.5	41.1	2.1	1.8	1.4
			Complex with TF (Tig)	67.4	57.0	44.0	2.6	2.2	1.7
P0AGH3	SapB	Cell inner membrane	Monomer	104.4	88.2	68.1	7.8	6.6	5.1
			Complex with SecB	68.6	56.0	44.8	2.7	2.2	1.8
			Complex with TF (Tig)	74.8	63.2	48.8	3.4	2.9	2.2
P0AGH5	SapC	Cell inner membrane	Monomer	110.0	92.9	71.8	8.8	7.4	5.7

UniProtKB A. N.	Protein name	Subcellular location	Subunit	D_0 $\left(\frac{\mu m^2}{s}\right)$			D_{cyto} $\left(\frac{\mu m^2}{s}\right)$		
				310 K	303 K	293 K	310 K	303 K	293 K
			Complex with SecB	69.8	57.0	45.5	2.8	2.3	1.8
			Complex with TF (Tig)	76.4	64.6	49.9	3.6	3.0	2.3
P0AAH4	SapD	Cell inner membrane	Monomer	102.6	86.7	66.9	7.4	6.3	4.9
			Complex with SecB	68.2	55.7	44.5	2.7	2.2	1.7
			Complex with TF (Tig)	74.3	62.7	48.5	3.3	2.8	2.2
P0AAH8	SapF	Cell inner membrane	Monomer	111.3	94.0	72.6	9.0	7.6	5.9
			Complex with SecB	70.0	57.2	45.7	2.9	2.3	1.9
			Complex with TF (Tig)	76.8	64.9	50.1	3.6	3.1	2.4
P04995	SbcB		Monomer	88.7	75.0	57.9	5.2	4.4	3.4
P13458	SbcC		Monomer	65.4	55.3	42.7	2.4	2.0	1.6
			Heterodimer (SbcC−SbcD)	57.7	48.7	37.7	1.7	1.4	1.1
P0AG76	SbcD		Monomer	95.9	81.0	62.6	6.3	5.4	4.1
			Heterodimer (SbcC−SbcD)	57.7	48.7	37.7	1.7	1.4	1.1
P27253	Sbm		Monomer	77.2	65.2	50.4	3.7	3.1	2.4
P0AFY6	SbmA	Cell inner membrane	Monomer	94.5	79.8	61.7	6.1	5.2	4.0
			Complex with SecB	66.1	54.0	43.1	2.4	2.0	1.6
			Complex with TF (Tig)	71.5	60.4	46.6	3.0	2.5	2.0
P33012	SbmC	Cytoplasm	Monomer	136.8	115.5	89.3	14.3	12.1	9.3
P0AG78	Sbp	Periplasm	Monomer	103.7	87.6	67.7	7.6	6.5	5.0
			Complex with SecB	68.4	55.9	44.7	2.7	2.2	1.8
			Complex with TF (Tig)	74.6	63.0	48.7	3.4	2.9	2.2
P16095	SdaA		Monomer	92.6	78.2	60.4	5.8	4.9	3.8
P30744	SdaB		Monomer	92.7	78.3	60.5	5.8	4.9	3.8
P0AAD6	SdaC	Cell inner membrane	Monomer	94.1	79.5	61.4	6.1	5.1	4.0
			Complex with SecB	66.0	53.9	43.1	2.4	2.0	1.6
			Complex with TF (Tig)	71.3	60.3	46.6	3.0	2.5	2.0
P0AC41	SdhA	Cell inner membrane	Monomer	83.1	70.2	54.2	4.5	3.8	2.9
P07014	SdhB	Cell inner membrane	Monomer	117.3	99.1	76.5	10.2	8.6	6.6
			Complex with SecB	71.1	58.1	46.4	3.0	2.4	1.9
			Complex with TF (Tig)	78.3	66.2	51.1	3.8	3.2	2.5
P69054	SdhC	Cell inner membrane	Monomer	150.0	126.7	97.9	17.5	14.7	11.4

UniProtKB A. N.	Protein name	Subcellular location	Subunit	D_0 $\left(\frac{\mu m^2}{s}\right)$			D_{cyto} $\left(\frac{\mu m^2}{s}\right)$		
				310 K	303 K	293 K	310 K	303 K	293 K
			Complex with SecB	75.1	61.4	49.0	3.4	2.8	2.2
			Complex with TF (Tig)	84.1	71.1	54.9	4.6	3.9	3.0
P0AC44	SdhD	Cell inner membrane	Monomer	156.3	132.0	102.0	19.0	16.1	12.4
			Complex with SecB	75.6	61.8	49.3	3.5	2.9	2.3
			Complex with TF (Tig)	84.9	71.7	55.4	4.7	4.0	3.1
P07026	SdiA		Monomer	115.0	97.2	75.1	9.7	8.2	6.3
P10408	SecA	Cell inner membrane	Monomer	69.4	58.6	45.3	2.8	2.4	1.8
			Homodimer	52.9	43.2	34.5	1.3	1.1	0.9
P0AG86	SecB	Cytoplasm	Monomer	139.2	117.6	90.9	14.9	12.6	9.7
			Homotetramer	80.9	66.1	52.8	4.2	3.4	2.7
P0AG90	SecD	Cell inner membrane	Monomer	82.0	69.3	53.5	4.3	3.6	2.8
			Complex with SecB	62.1	50.7	40.5	2.1	1.7	1.3
			Complex with TF (Tig)	66.3	56.0	43.2	2.5	2.1	1.6
P0AG96	SecE	Cell inner membrane	Monomer	152.7	129.0	99.7	18.2	15.3	11.8
			Complex with SecB	75.3	61.6	49.2	3.5	2.8	2.3
			Complex with TF (Tig)	84.5	71.4	55.1	4.6	3.9	3.0
P0AG93	SecF	Cell inner membrane	Monomer	105.1	88.8	68.6	7.9	6.7	5.1
			Complex with SecB	68.8	56.2	44.9	2.7	2.2	1.8
			Complex with TF (Tig)	75.1	63.4	49.0	3.4	2.9	2.2
P0AG99	SecG	Cell inner membrane	Monomer	164.1	138.6	107.1	21.1	17.8	13.8
			Complex with SecB	76.2	62.2	49.7	3.6	2.9	2.3
			Complex with TF (Tig)	85.7	72.4	55.9	4.8	4.1	3.1
P62395	SecM	Cytoplasm > cytosol<br>Periplasm	Monomer	134.5	113.6	87.8	13.8	11.7	9.0
P0AGA2	SecY	Cell inner membrane	Monomer	92.9	78.5	60.6	5.9	5.0	3.8
			Complex with SecB	65.6	53.6	42.8	2.4	2.0	1.6
			Complex with TF (Tig)	70.9	59.9	46.3	2.9	2.5	1.9
P0A821	SelA	Cytoplasm	Monomer	91.4	77.2	59.6	5.6	4.8	3.7
			Homodecamer	37.0	30.3	24.2	0.4	0.4	0.3
P14081	SelB	Cytoplasm	Monomer	81.0	68.4	52.8	4.2	3.5	2.7
P16456	SelD		Monomer	103.6	87.6	67.6	7.6	6.4	5.0

UniProtKB A. N.	Protein name	Subcellular location	Subunit	D_0 $\left(\frac{\mu m^2}{s}\right)$			D_{cyto} $\left(\frac{\mu m^2}{s}\right)$		
				310 K	303 K	293 K	310 K	303 K	293 K
P33667	SelU		Monomer	99.1	83.7	64.7	6.9	5.8	4.5
P0AFY8	SeqA		Monomer	130.7	110.4	85.3	13.0	10.9	8.5
P0A9T0	SerA		Monomer	96.4	81.4	62.9	6.4	5.4	4.2
			Homotetramer	56.0	45.7	36.5	1.5	1.3	1.0
P0AGB0	SerB		Monomer	105.5	89.1	68.9	8.0	6.7	5.2
P23721	SerC	Cytoplasm	Monomer	100.4	84.8	65.5	7.1	6.0	4.6
			Homodimer	76.5	62.5	49.9	3.6	2.9	2.4
P0A8L1	SerS	Cytoplasm	Monomer	93.0	78.5	60.7	5.9	5.0	3.8
			Homodimer	70.8	57.9	46.2	2.9	2.4	1.9
P31675	SetA	Cell inner membrane	Monomer	97.6	82.5	63.7	6.6	5.6	4.3
			Complex with SecB	67.0	54.7	43.7	2.5	2.1	1.7
			Complex with TF (Tig)	72.6	61.4	47.4	3.1	2.7	2.1
P33026	SetB	Cell inner membrane	Monomer	97.6	82.5	63.7	6.6	5.6	4.3
			Complex with SecB	67.0	54.7	43.7	2.5	2.1	1.7
			Complex with TF (Tig)	72.6	61.3	47.4	3.1	2.7	2.1
P31436	SetC	Cell inner membrane	Monomer	97.0	81.9	63.3	6.5	5.5	4.2
			Complex with SecB	66.8	54.6	43.6	2.5	2.1	1.6
			Complex with TF (Tig)	72.4	61.1	47.2	3.1	2.6	2.0
P0AAA5	Sfa	Cell membrane	Monomer	182.0	153.7	118.7	26.1	22.0	17.0
			Complex with SecB	77.2	63.0	50.4	3.7	3.0	2.4
			Complex with TF (Tig)	87.3	73.7	56.9	5.0	4.2	3.3
P0ABW5	SfmA	Fimbrium	Monomer	135.6	114.6	88.5	14.1	11.9	9.2
			Complex with SecB	73.7	60.2	48.1	3.3	2.7	2.1
			Complex with TF (Tig)	82.0	69.3	53.5	4.3	3.6	2.8
P77249	SfmC	Periplasm	Monomer	119.6	101.0	78.0	10.6	9.0	6.9
			Complex with SecB	71.5	58.4	46.7	3.0	2.5	2.0
			Complex with TF (Tig)	78.9	66.6	51.5	3.9	3.3	2.5
P77468	SfmD	Cell outer membrane	Monomer	71.2	60.1	46.4	3.0	2.5	1.9
			Complex with SecB	57.5	47.0	37.5	1.7	1.4	1.1
			Complex with TF (Tig)	60.7	51.2	39.6	1.9	1.6	1.3
P38052	SfmF	Fimbrium	Monomer	136.4	115.2	89.0	14.2	12.0	9.3

UniProtKB A. N.	Protein name	Subcellular location	Subunit	D_0 $\left(\frac{\mu m^2}{s}\right)$			D_{cyto} $\left(\frac{\mu m^2}{s}\right)$		
				310 K	303 K	293 K	310 K	303 K	293 K
			Complex with SecB	73.8	60.3	48.1	3.3	2.7	2.1
			Complex with TF (Tig)	82.1	69.4	53.6	4.3	3.6	2.8
P75715	SfmH	Fimbrium	Monomer	104.8	88.5	68.4	7.8	6.6	5.1
			Complex with SecB	68.7	56.1	44.8	2.7	2.2	1.8
			Complex with TF (Tig)	74.9	63.3	48.9	3.4	2.9	2.2
P0A823	SfsA		Monomer	118.2	99.9	77.1	10.3	8.7	6.8
P0ACH1	SfsB		Monomer	169.3	143.0	110.5	22.5	19.0	14.7
P37680	SgbE		Monomer	119.4	100.9	77.9	10.6	8.9	6.9
P37678	SgbH		Monomer	123.5	104.4	80.6	11.4	9.7	7.5
			Homodimer	94.1	76.9	61.4	6.1	5.0	4.0
P37679	SgbU		Monomer	108.7	91.9	71.0	8.5	7.2	5.6
P39363	SgcA	Cytoplasm	Monomer	144.8	122.3	94.5	16.2	13.7	10.6
P58035	SgcB	Cytoplasm	Monomer	173.9	146.9	113.5	23.8	20.1	15.5
P39365	SgcC	Cell inner membrane	Monomer	94.3	79.7	61.5	6.1	5.1	4.0
			Complex with SecB	66.0	54.0	43.1	2.4	2.0	1.6
			Complex with TF (Tig)	71.4	60.3	46.6	3.0	2.5	2.0
P39362	SgcE		Monomer	124.0	104.8	80.9	11.5	9.7	7.5
P39364	SgcQ		Monomer	113.1	95.6	73.8	9.4	7.9	6.1
P39361	SgcR		Monomer	113.2	95.7	73.9	9.4	7.9	6.1
P39366	SgcX		Monomer	99.6	84.1	65.0	6.9	5.9	4.5
P33595	SgrR		Monomer	83.3	70.4	54.4	4.5	3.8	2.9
C1P5Z7	SgrT		Monomer	220.6	186.4	144.0	38.1	32.2	24.9
P76350	ShiA	Cell inner membrane	Monomer	93.4	78.9	61.0	5.9	5.0	3.9
			Complex with SecB	65.8	53.8	42.9	2.4	2.0	1.6
			Complex with TF (Tig)	71.1	60.1	46.4	3.0	2.5	1.9
C1P611	ShoB	Membrane	Monomer	272.9	230.6	178.1	56.7	47.9	37.0
			Complex with SecB	79.5	64.9	51.9	4.0	3.2	2.6
			Complex with TF (Tig)	90.9	76.8	59.3	5.6	4.7	3.6
P38392	SieB	Cell inner membrane	Monomer	133.5	112.8	87.1	13.6	11.5	8.9
			Complex with SecB	73.4	60.0	47.9	3.2	2.6	2.1
			Complex with TF (Tig)	81.6	69.0	53.3	4.3	3.6	2.8

UniProtKB A. N.	Protein name	Subcellular location	Subunit	D_0 $\left(\frac{\mu m^2}{s}\right)$			D_{cyto} $\left(\frac{\mu m^2}{s}\right)$		
				310 K	303 K	293 K	310 K	303 K	293 K
P0AGM5	SirB1		Monomer	110.9	93.7	72.4	8.9	7.6	5.8
Q46755	SirB2	Cell inner membrane	Monomer	148.6	125.5	97.0	17.1	14.5	11.2
			Complex with SecB	75.0	61.3	48.9	3.4	2.8	2.2
			Complex with TF (Tig)	83.9	70.9	54.8	4.6	3.9	3.0
P76502	SixA		Monomer	139.5	117.8	91.0	14.9	12.6	9.7
P0AEU7	Skp	Periplasm	Monomer	138.0	116.5	90.0	14.6	12.3	9.5
			Complex with SecB	74.0	60.4	48.3	3.3	2.7	2.2
			Complex with TF (Tig)	82.4	69.6	53.8	4.4	3.7	2.8
P0C093	SlmA	Cytoplasm > nucleoid	Monomer	124.8	105.4	81.4	11.7	9.9	7.6
P37194	Slp	Cell outer membrane	Monomer	129.1	109.0	84.2	12.6	10.6	8.2
			Complex with SecB	72.9	59.5	47.6	3.2	2.6	2.1
			Complex with TF (Tig)	80.8	68.3	52.8	4.1	3.5	2.7
P0AGC3	Slt	Periplasm	Monomer	79.0	66.7	51.5	3.9	3.3	2.6
			Complex with SecB	60.9	49.7	39.7	2.0	1.6	1.3
			Complex with TF (Tig)	64.8	54.7	42.3	2.3	2.0	1.5
P0A8W2	SlyA		Monomer	142.3	120.2	92.8	15.6	13.2	10.2
			Homodimer	108.4	88.6	70.7	8.5	6.9	5.5
P0A905	SlyB	Cell outer membrane	Monomer	144.9	122.4	94.6	16.2	13.7	10.6
			Complex with SecB	74.7	61.0	48.7	3.4	2.8	2.2
			Complex with TF (Tig)	83.4	70.5	54.4	4.5	3.8	2.9
P0A9K9	SlyD	Cytoplasm	Monomer	129.3	109.3	84.4	12.7	10.7	8.3
P0A8R4	SlyX		Monomer	186.3	157.4	121.6	27.3	23.1	17.8
P30852	Smf		Monomer	99.3	83.9	64.8	6.9	5.8	4.5
P0A828	Smg		Monomer	135.5	114.5	88.4	14.0	11.9	9.2
P0AGC7	Smp	Cell membrane	Monomer	122.2	103.3	79.8	11.2	9.4	7.3
			Complex with SecB	71.9	58.8	46.9	3.1	2.5	2.0
			Complex with TF (Tig)	79.5	67.1	51.8	4.0	3.4	2.6
P0A937	SmpA	Cell outer membrane	Monomer	159.1	134.4	103.8	19.8	16.7	12.9
			Complex with SecB	75.8	61.9	49.5	3.5	2.9	2.3
			Complex with TF (Tig)	85.2	72.0	55.6	4.7	4.0	3.1

UniProtKB A. N.	Protein name	Subcellular location	Subunit	D_0 $\left(\frac{\mu m^2}{s}\right)$			D_{cyto} $\left(\frac{\mu m^2}{s}\right)$		
				310 K	303 K	293 K	310 K	303 K	293 K
P0A832	SmpB	Cytoplasm	Monomer	136.2	115.1	88.9	14.2	12.0	9.3
P36566	SmtA		Monomer	112.4	95.0	73.4	9.2	7.8	6.0
P00448	SodA		Monomer	124.3	105.0	81.1	11.6	9.8	7.6
			Homodimer	94.7	77.4	61.8	6.1	5.0	4.0
P0AGD3	SodB		Monomer	128.3	108.4	83.8	12.4	10.5	8.1
			Homodimer	97.8	79.9	63.8	6.6	5.4	4.3
P0AGD1	SodC	Periplasm	Monomer	138.0	116.6	90.0	14.6	12.3	9.5
			Complex with SecB	74.0	60.4	48.3	3.3	2.7	2.2
			Complex with TF (Tig)	82.4	69.6	53.8	4.4	3.7	2.8
P15373	SohA	Cytoplasm	Monomer	158.8	134.1	103.6	19.7	16.6	12.9
			Homodimer	121.0	98.8	79.0	10.9	8.9	7.1
P0AG14	SohB	Cell inner membrane	Monomer	100.8	85.2	65.8	7.1	6.0	4.7
			Complex with SecB	67.8	55.4	44.2	2.6	2.1	1.7
			Complex with TF (Tig)	73.7	62.3	48.1	3.3	2.8	2.1
P40874	SolA		Monomer	99.3	83.9	64.8	6.9	5.8	4.5
P31122	SotB	Cell inner membrane	Monomer	97.8	82.6	63.8	6.6	5.6	4.3
			Complex with SecB	67.0	54.7	43.7	2.5	2.1	1.7
			Complex with TF (Tig)	72.7	61.4	47.4	3.2	2.7	2.1
P0ACS2	SoxR		Monomer	139.6	118.0	91.1	15.0	12.7	9.8
			Homodimer	106.4	86.9	69.4	8.1	6.6	5.3
P0A9E2	SoxS	Cytoplasm	Monomer	156.1	131.9	101.8	19.0	16.0	12.4
P21170	SpeA	Periplasm	Monomer	78.8	66.5	51.4	3.9	3.3	2.5
			Complex with SecB	60.8	49.7	39.7	1.9	1.6	1.3
			Complex with TF (Tig)	64.7	54.7	42.2	2.3	1.9	1.5
P60651	SpeB		Monomer	107.3	90.7	70.0	8.3	7.0	5.4
P21169	SpeC		Monomer	76.6	64.7	50.0	3.6	3.0	2.4
P0A7F6	SpeD		Monomer	111.6	94.3	72.8	9.1	7.7	5.9
P09158	SpeE		Monomer	108.9	92.0	71.1	8.6	7.2	5.6
			Homodimer	83.0	67.8	54.2	4.4	3.6	2.9
P24169	SpeF		Monomer	75.5	63.8	49.2	3.5	2.9	2.3
P0A951	SpeG	Cytoplasm	Monomer	126.9	107.2	82.8	12.1	10.3	7.9

UniProtKB A. N.	Protein name	Subcellular location	Subunit	D_0 $\left(\frac{\mu m^2}{s}\right)$			D_{cyto} $\left(\frac{\mu m^2}{s}\right)$		
				310 K	303 K	293 K	310 K	303 K	293 K
			Homotetramer	73.7	60.2	48.1	3.3	2.7	2.1
P0AG24	SpoT	Cytoplasm	Monomer	76.6	64.7	50.0	3.6	3.1	2.4
P08395	SppA	Cell inner membrane	Monomer	81.7	69.1	53.3	4.3	3.6	2.8
			Complex with SecB	62.0	50.6	40.4	2.0	1.7	1.3
			Complex with TF (Tig)	66.1	55.9	43.2	2.4	2.1	1.6
P0AFV4	Spr	Cell membrane	Monomer	128.9	108.9	84.1	12.6	10.6	8.2
			Complex with SecB	72.9	59.5	47.5	3.2	2.6	2.1
			Complex with TF (Tig)	80.8	68.3	52.7	4.1	3.5	2.7
P39902	SprT	Cytoplasm	Monomer	133.2	112.5	86.9	13.5	11.4	8.8
P77754	Spy	Periplasm	Monomer	136.4	115.3	89.0	14.2	12.0	9.3
			Complex with SecB	73.8	60.3	48.1	3.3	2.7	2.1
			Complex with TF (Tig)	82.1	69.4	53.6	4.3	3.7	2.8
P68191	Sra		Monomer	224.7	189.8	146.6	39.4	33.3	25.7
P56579	SrlA	Cell inner membrane	Monomer	130.0	109.8	84.8	12.8	10.8	8.4
			Complex with SecB	73.0	59.6	47.6	3.2	2.6	2.1
			Complex with TF (Tig)	81.0	68.4	52.9	4.2	3.5	2.7
P05706	SrlB	Cytoplasm	Monomer	154.3	130.3	100.7	18.5	15.7	12.1
P05707	SrlD		Monomer	115.5	97.5	75.3	9.8	8.3	6.4
			Homotetramer	67.1	54.8	43.8	2.5	2.1	1.7
P56580	SrlE	Cell inner membrane	Monomer	107.6	90.9	70.2	8.3	7.0	5.4
			Complex with SecB	69.3	56.6	45.2	2.8	2.3	1.8
			Complex with TF (Tig)	75.8	64.0	49.5	3.5	3.0	2.3
P15082	SrlR		Monomer	114.8	97.0	74.9	9.7	8.2	6.3
P21507	SrmB		Monomer	91.9	77.6	59.9	5.7	4.8	3.7
P0AGE0	Ssb		Monomer	134.2	113.4	87.6	13.7	11.6	9.0
			Homotetramer	77.9	63.7	50.9	3.8	3.1	2.5
P31142	SseA	Cytoplasm	Monomer	111.0	93.8	72.4	9.0	7.6	5.8
P0AFZ1	SseB		Monomer	114.2	96.5	74.5	9.6	8.1	6.2
Q46812	SsnA		Monomer	92.6	78.3	60.5	5.8	4.9	3.8
P0ACA3	SspA		Monomer	121.8	102.9	79.5	11.1	9.4	7.2
P0AFZ3	SspB		Monomer	136.2	115.1	88.9	14.2	12.0	9.3

UniProtKB A. N.	Protein name	Subcellular location	Subunit	D_0 $\left(\frac{\mu m^2}{s}\right)$			D_{cyto} $\left(\frac{\mu m^2}{s}\right)$		
				310 K	303 K	293 K	310 K	303 K	293 K
P0AGE4	SstT	Cell inner membrane	Monomer	97.0	81.9	63.3	6.5	5.5	4.2
			Complex with SecB	66.8	54.6	43.6	2.5	2.1	1.6
			Complex with TF (Tig)	72.4	61.2	47.2	3.1	2.6	2.0
P75853	SsuA	Periplasm	Monomer	106.1	89.6	69.2	8.1	6.8	5.3
			Complex with SecB	69.0	56.3	45.0	2.7	2.2	1.8
			Complex with TF (Tig)	75.3	63.7	49.2	3.5	2.9	2.3
P0AAI1	SsuB	Cell inner membrane	Monomer	115.7	97.7	75.5	9.8	8.3	6.4
			Complex with SecB	70.8	57.9	46.2	2.9	2.4	1.9
			Complex with TF (Tig)	77.9	65.8	50.9	3.8	3.2	2.5
P75851	SsuC	Cell inner membrane	Monomer	113.8	96.1	74.2	9.5	8.0	6.2
			Complex with SecB	70.5	57.6	46.0	2.9	2.4	1.9
			Complex with TF (Tig)	77.5	65.4	50.5	3.7	3.1	2.4
P80645	SsuD		Monomer	98.5	83.2	64.3	6.8	5.7	4.4
			Homotetramer	57.2	46.7	37.3	1.6	1.3	1.1
P80644	SsuE		Monomer	128.4	108.5	83.8	12.5	10.5	8.1
			Homodimer	97.8	79.9	63.8	6.7	5.4	4.3
P33227	StfE		Monomer	137.6	116.3	89.8	14.5	12.3	9.5
P77515	StfQ		Monomer	108.2	91.4	70.6	8.4	7.1	5.5
P76072	StfR		Monomer	66.5	56.2	43.4	2.5	2.1	1.6
P27306	SthA	Cytoplasm	Monomer	90.7	76.6	59.2	5.5	4.7	3.6
			Homooctamer	40.1	32.8	26.2	0.6	0.5	0.4
P0ACG1	StpA		Monomer	145.8	123.2	95.2	16.5	13.9	10.7
P0AFG3	SucA		Monomer	68.6	58.0	44.8	2.7	2.3	1.8
			Homodimer	52.3	42.7	34.1	1.3	1.0	0.8
P0AFG6	SucB		Monomer	96.5	81.5	63.0	6.4	5.4	4.2
P0A836	SucC		Monomer	98.9	83.5	64.5	6.8	5.8	4.4
			Heterotetramer ($SucC_2-SucD_2$)	60.9	51.5	39.8	2.0	1.7	1.3
P0AGE9	SucD		Monomer	112.5	95.0	73.4	9.2	7.8	6.0
			Heterotetramer ($SucC_2-SucD_2$)	60.9	51.5	39.8	2.0	1.7	1.3
P77667	SufA		Monomer	154.3	130.3	100.7	18.5	15.7	12.1
P77522	SufB		Monomer	88.6	74.8	57.8	5.2	4.4	3.4

UniProtKB A. N.	Protein name	Subcellular location	Subunit	D_0 $\left(\frac{\mu m^2}{s}\right)$ 310 K	303 K	293 K	D_{cyto} $\left(\frac{\mu m^2}{s}\right)$ 310 K	303 K	293 K
P77499	SufC	Cytoplasm	Monomer	115.9	97.9	75.6	9.9	8.4	6.5
P77689	SufD		Monomer	94.2	79.6	61.5	6.1	5.1	4.0
			Homodimer	71.8	58.6	46.8	3.0	2.5	2.0
P76194	SufE	Cytoplasm	Monomer	144.2	121.8	94.1	16.1	13.6	10.5
			Homodimer	109.9	89.8	71.7	8.8	7.1	5.7
P26648	SufI	Periplasm	Monomer	90.5	76.5	59.1	5.5	4.6	3.6
			Complex with SecB	64.9	53.0	42.4	2.3	1.9	1.5
			Complex with TF (Tig)	69.9	59.1	45.6	2.8	2.4	1.9
P77444	SufS	Cytoplasm	Monomer	96.1	81.2	62.7	6.4	5.4	4.2
			Homodimer	73.3	59.9	47.8	3.2	2.6	2.1
P69937	SugE	Cell inner membrane	Monomer	166.8	140.9	108.8	21.8	18.4	14.2
			Complex with SecB	76.3	62.4	49.8	3.6	2.9	2.3
			Complex with TF (Tig)	86.0	72.6	56.1	4.8	4.1	3.2
P0ADG4	SuhB		Monomer	113.4	95.8	74.0	9.4	7.9	6.1
P0AFZ5	SulA		Monomer	134.7	113.8	87.9	13.8	11.7	9.0
P75792	SupH		Monomer	111.6	94.2	72.8	9.1	7.7	5.9
P0ABZ6	SurA	Periplasm	Monomer	93.8	79.3	61.2	6.0	5.1	3.9
			Complex with SecB	65.9	53.8	43.0	2.4	2.0	1.6
			Complex with TF (Tig)	71.2	60.2	46.5	3.0	2.5	2.0
P0A840	SurE	Cytoplasm	Monomer	117.0	98.9	76.4	10.1	8.5	6.6
P75869	Sxy		Monomer	122.1	103.2	79.7	11.1	9.4	7.3
P0A8U0	Syd	Cell inner membrane	Monomer	129.7	109.6	84.6	12.7	10.8	8.3
P39394	SymE	Cytoplasm	Monomer	159.6	134.8	104.1	19.9	16.8	13.0
P68398	TadA		Monomer	134.9	114.0	88.0	13.9	11.7	9.1
			Homodimer	102.8	84.0	67.1	7.5	6.1	4.9
P05100	Tag		Monomer	128.7	108.8	84.0	12.5	10.6	8.2
P0A867	TalA	Cytoplasm	Monomer	104.8	88.5	68.4	7.8	6.6	5.1
P0A870	TalB	Cytoplasm	Monomer	105.3	89.0	68.7	7.9	6.7	5.2
			Homodimer	80.3	65.6	52.4	4.1	3.3	2.7
P76145	Tam	Cytoplasm	Monomer	113.6	96.0	74.2	9.5	8.0	6.2
P07018	Tap	Cell inner membrane	Monomer	86.9	73.4	56.7	5.0	4.2	3.2

UniProtKB A. N.	Protein name	Subcellular location	Subunit	D_0 $\left(\frac{\mu m^2}{s}\right)$			D_{cyto} $\left(\frac{\mu m^2}{s}\right)$		
				310 K	303 K	293 K	310 K	303 K	293 K
			Complex with SecB	63.8	52.1	41.6	2.2	1.8	1.4
			Complex with TF (Tig)	68.5	57.8	44.7	2.7	2.3	1.8
P07017	Tar	Cell inner membrane	Monomer	85.5	72.2	55.8	4.8	4.0	3.1
			Complex with SecB	63.3	51.7	41.3	2.2	1.8	1.4
			Complex with TF (Tig)	67.8	57.3	44.3	2.6	2.2	1.7
P0A9T4	Tas		Monomer	101.7	85.9	66.4	7.3	6.2	4.8
P69428	TatA	Cell inner membrane	Monomer	174.8	147.7	114.1	24.0	20.3	15.7
P69425	TatB	Cell inner membrane	Monomer	135.8	114.7	88.6	14.1	11.9	9.2
			Complex with SecB	73.7	60.2	48.1	3.3	2.7	2.1
			Complex with TF (Tig)	82.0	69.3	53.5	4.3	3.6	2.8
P69423	TatC	Cell inner membrane	Monomer	113.8	96.2	74.3	9.5	8.0	6.2
			Complex with SecB	70.5	57.6	46.0	2.9	2.4	1.9
			Complex with TF (Tig)	77.5	65.5	50.6	3.7	3.1	2.4
P27859	TatD	Cytoplasm	Monomer	113.7	96.0	74.2	9.5	8.0	6.2
P0A843	TatE	Cell inner membrane	Monomer	198.1	167.4	129.3	30.9	26.1	20.2
			Complex with SecB	77.8	63.6	50.8	3.8	3.1	2.5
			Complex with TF (Tig)	88.3	74.6	57.6	5.2	4.4	3.4
Q47537	TauA	Periplasm	Monomer	106.5	89.9	69.5	8.1	6.9	5.3
			Complex with SecB	69.1	56.4	45.1	2.8	2.2	1.8
			Complex with TF (Tig)	75.5	63.7	49.2	3.5	2.9	2.3
Q47538	TauB	Cell inner membrane	Monomer	114.7	96.9	74.9	9.7	8.2	6.3
			Complex with SecB	70.7	57.7	46.1	2.9	2.4	1.9
			Complex with TF (Tig)	77.7	65.6	50.7	3.7	3.2	2.4
Q47539	TauC	Cell inner membrane	Monomer	112.4	95.0	73.4	9.2	7.8	6.0
			Complex with SecB	70.3	57.4	45.8	2.9	2.4	1.9
			Complex with TF (Tig)	77.1	65.1	50.3	3.7	3.1	2.4
P37610	TauD		Monomer	108.8	91.9	71.0	8.6	7.2	5.6
			Homodimer	82.9	67.7	54.1	4.4	3.6	2.9
P0ACQ7	TdcA		Monomer	106.1	89.7	69.3	8.1	6.8	5.3
P0AGF6	TdcB		Monomer	105.3	89.0	68.7	7.9	6.7	5.2
			Homotetramer	61.2	50.0	39.9	2.0	1.6	1.3

UniProtKB A. N.	Protein name	Subcellular location	Subunit	D_0 $\left(\frac{\mu m^2}{s}\right)$			D_{cyto} $\left(\frac{\mu m^2}{s}\right)$		
				310 K	303 K	293 K	310 K	303 K	293 K
P0AAD8	TdcC	Cell inner membrane	Monomer	92.6	78.2	60.4	5.8	4.9	3.8
			Complex with SecB	65.6	53.6	42.8	2.4	2.0	1.6
			Complex with TF (Tig)	70.8	59.8	46.2	2.9	2.5	1.9
P11868	TdcD		Monomer	97.1	82.0	63.3	6.5	5.5	4.3
P42632	TdcE	Cytoplasm	Monomer	74.2	62.7	48.4	3.3	2.8	2.2
P0AGL2	TdcF		Monomer	151.2	127.7	98.6	17.8	15.0	11.6
P42630	TdcG		Monomer	92.9	78.5	60.6	5.9	5.0	3.8
P11866	TdcR		Monomer	183.0	154.6	119.4	26.3	22.3	17.2
P07913	Tdh	Cytoplasm	Monomer	103.0	87.0	67.2	7.5	6.4	4.9
			Homotetramer	59.8	48.9	39.0	1.9	1.5	1.2
P23331	Tdk	Cytoplasm	Monomer	123.5	104.3	80.6	11.4	9.7	7.5
			Homotetramer	71.7	58.6	46.8	3.0	2.5	2.0
P25396	TehA	Cell inner membrane	Monomer	104.5	88.3	68.2	7.8	6.6	5.1
			Complex with SecB	68.6	56.1	44.8	2.7	2.2	1.8
			Complex with TF (Tig)	74.9	63.2	48.9	3.4	2.9	2.2
P25397	TehB	Cytoplasm	Monomer	125.5	106.0	81.9	11.8	10.0	7.7
			Homodimer	95.6	78.1	62.4	6.3	5.1	4.1
P0ADA1	TesA	Periplasm	Monomer	123.2	104.1	80.4	11.4	9.6	7.4
			Complex with SecB	72.1	58.9	47.0	3.1	2.5	2.0
			Complex with TF (Tig)	79.7	67.3	52.0	4.0	3.4	2.6
P0AGG2	TesB		Monomer	109.4	92.4	71.4	8.7	7.3	5.7
			Homotetramer	63.5	51.9	41.5	2.2	1.8	1.4
P77712	TesC		Monomer	146.8	124.0	95.8	16.7	14.1	10.9
			Homotetramer	85.3	69.7	55.6	4.7	3.9	3.1
P77699	TfaD		Monomer	114.8	96.9	74.9	9.7	8.2	6.3
P09153	TfaE		Monomer	127.4	107.6	83.1	12.3	10.3	8.0
P76155	TfaQ		Monomer	128.2	108.3	83.6	12.4	10.5	8.1
P77163	TfaR		Monomer	128.2	108.3	83.6	12.4	10.5	8.1
P77326	TfaS		Monomer	157.3	132.9	102.6	19.3	16.3	12.6
			Monomer	97.8	82.6	63.8	6.6	5.6	4.3
P0A847	Tgt		Homodimer	74.5	60.9	48.6	3.4	2.7	2.2

UniProtKB A. N.	Protein name	Subcellular location	Subunit	$D_0\ \left(\frac{\mu m^2}{s}\right)$			$D_{\text{cyto}}\ \left(\frac{\mu m^2}{s}\right)$		
				310 K	303 K	293 K	310 K	303 K	293 K
			Homotrimer	63.5	53.7	41.5	2.2	1.9	1.4
P31550	ThiB	Periplasm	Monomer	104.2	88.1	68.0	7.7	6.5	5.0
			Complex with SecB	68.6	56.0	44.7	2.7	2.2	1.8
			Complex with TF (Tig)	74.8	63.2	48.8	3.4	2.9	2.2
P30136	ThiC		Monomer	80.1	67.6	52.3	4.0	3.4	2.6
			Homodimer	61.0	49.9	39.8	2.0	1.6	1.3
P76422	ThiD		Monomer	114.2	96.5	74.5	9.6	8.1	6.2
P30137	ThiE		Monomer	124.4	105.1	81.2	11.6	9.8	7.6
P30138	ThiF		Monomer	116.9	98.8	76.3	10.1	8.5	6.6
			Homodimer	89.1	72.8	58.1	5.3	4.3	3.5
P30139	ThiG	Cytoplasm	Monomer	117.1	98.9	76.4	10.1	8.5	6.6
			Homotetramer	68.0	55.5	44.4	2.6	2.2	1.7
			Heterodimer (ThiG−ThiH)	80.4	67.9	52.4	4.1	3.5	2.7
P30140	ThiH		Monomer	97.1	82.0	63.4	6.5	5.5	4.3
			Heterodimer (ThiG−ThiH)	80.4	67.9	52.4	4.1	3.5	2.7
P77718	ThiI	Cytoplasm	Monomer	88.4	74.7	57.7	5.2	4.4	3.4
P75948	ThiK		Monomer	108.8	91.9	71.0	8.6	7.2	5.6
P0AGG0	ThiL		Monomer	105.5	89.1	68.8	8.0	6.7	5.2
P76423	ThiM		Monomer	116.3	98.3	75.9	10.0	8.4	6.5
P31549	ThiP	Cell inner membrane	Monomer	85.7	72.4	55.9	4.8	4.1	3.1
			Complex with SecB	63.4	51.8	41.4	2.2	1.8	1.4
			Complex with TF (Tig)	67.9	57.4	44.3	2.6	2.2	1.7
P31548	ThiQ	Cell inner membrane	Monomer	120.5	101.8	78.6	10.8	9.1	7.0
			Complex with SecB	71.6	58.5	46.8	3.0	2.5	2.0
			Complex with TF (Tig)	79.1	66.8	51.6	3.9	3.3	2.6
O32583	ThiS		Monomer	195.1	164.8	127.3	29.9	25.3	19.5
P00561	ThrA		Monomer	73.2	61.8	47.8	3.2	2.7	2.1
			Homotetramer	42.5	34.7	27.7	0.7	0.6	0.4
P00547	ThrB	Cytoplasm	Monomer	107.2	90.6	70.0	8.3	7.0	5.4
P00934	ThrC		Monomer	94.0	79.4	61.3	6.0	5.1	3.9
P0AD86	ThrL		Monomer	315.8	266.8	206.1	73.7	62.3	48.1

UniProtKB A. N.	Protein name	Subcellular location	Subunit	D_0 $\left(\frac{\mu m^2}{s}\right)$			D_{cyto} $\left(\frac{\mu m^2}{s}\right)$		
				310 K	303 K	293 K	310 K	303 K	293 K
P0A8M3	ThrS	Cytoplasm	Monomer	78.7	66.5	51.4	3.9	3.3	2.5
			Homodimer	60.0	49.0	39.1	1.9	1.5	1.2
P0A884	ThyA	Cytoplasm	Monomer	111.5	94.2	72.7	9.0	7.6	5.9
			Homodimer	84.9	69.4	55.4	4.7	3.8	3.1
P0A850	Tig		Monomer	93.1	78.7	60.8	5.9	5.0	3.9
			Homodimer	71.0	58.0	46.3	3.0	2.4	1.9
P52097	TilS	Cytoplasm	Monomer	93.1	78.7	60.8	5.9	5.0	3.9
A5A627	TisB	Cell inner membrane	Monomer	268.9	227.2	175.5	55.2	46.6	36.0
			Complex with SecB	79.4	64.9	51.8	4.0	3.2	2.6
			Complex with TF (Tig)	90.8	76.7	59.3	5.5	4.7	3.6
P27302	TktA		Monomer	79.5	67.1	51.9	4.0	3.4	2.6
P33570	TktB		Monomer	79.1	66.8	51.6	3.9	3.3	2.6
P0AGG8	TldD		Monomer	90.8	76.7	59.3	5.6	4.7	3.6
P76562	TmcA	Cytoplasm	Monomer	78.4	66.2	51.1	3.8	3.2	2.5
P0A720	Tmk		Monomer	122.8	103.8	80.2	11.3	9.5	7.4
			Homodimer	93.6	76.5	61.1	6.0	4.9	3.9
P0A853	TnaA		Monomer	89.9	75.9	58.6	5.4	4.6	3.5
			Homotetramer	52.2	42.6	34.1	1.3	1.0	0.8
P23173	TnaB	Cell inner membrane	Monomer	95.5	80.7	62.3	6.3	5.3	4.1
			Complex with SecB	66.4	54.2	43.3	2.5	2.0	1.6
			Complex with TF (Tig)	71.9	60.7	46.9	3.1	2.6	2.0
P0AD89	TnaL		Monomer	280.5	237.0	183.0	59.6	50.4	38.9
P19934	TolA	Cell inner membrane	Monomer	97.3	82.2	63.5	6.6	5.5	4.3
			Complex with SecB	66.9	54.6	43.6	2.5	2.1	1.6
			Complex with TF (Tig)	72.5	61.2	47.3	3.1	2.6	2.0
P0A855	TolB	Periplasm	Monomer	94.9	80.2	61.9	6.2	5.2	4.0
			Complex with SecB	66.2	54.1	43.2	2.5	2.0	1.6
			Complex with TF (Tig)	71.6	60.5	46.7	3.0	2.6	2.0
P02930	TolC	Cell outer membrane	Monomer	89.2	75.4	58.2	5.3	4.5	3.5
			Complex with SecB	64.5	52.7	42.1	2.3	1.9	1.5
			Complex with TF (Tig)	69.4	58.7	45.3	2.8	2.4	1.8

UniProtKB A. N.	Protein name	Subcellular location	Subunit	D_0 $\left(\frac{\mu m^2}{s}\right)$			D_{cyto} $\left(\frac{\mu m^2}{s}\right)$		
				310 K	303 K	293 K	310 K	303 K	293 K
P0ABU9	TolQ	Cell inner membrane	Monomer	119.3	100.8	77.9	10.6	8.9	6.9
			Complex with SecB	71.5	58.4	46.6	3.0	2.5	2.0
			Complex with TF (Tig)	78.8	66.6	51.4	3.9	3.3	2.5
P0ABV6	TolR	Cell inner membrane	Monomer	145.7	123.1	95.1	16.4	13.9	10.7
			Complex with SecB	74.7	61.1	48.8	3.4	2.8	2.2
			Complex with TF (Tig)	83.5	70.6	54.5	4.5	3.8	2.9
P0AAR0	TomB	Cytoplasm	Monomer	148.9	125.8	97.2	17.2	14.5	11.2
P02929	TonB	Cell inner membrane	Monomer	118.5	100.1	77.3	10.4	8.8	6.8
			Complex with SecB	71.3	58.3	46.5	3.0	2.4	2.0
			Complex with TF (Tig)	78.6	66.4	51.3	3.9	3.3	2.5
P06612	TopA		Monomer	70.7	59.7	46.1	2.9	2.5	1.9
P14294	TopB		Monomer	79.1	66.8	51.6	3.9	3.3	2.6
P33225	TorA	Periplasm	Monomer	71.5	60.4	46.7	3.0	2.6	2.0
			Complex with SecB	57.7	47.1	37.6	1.7	1.4	1.1
			Complex with TF (Tig)	60.9	51.4	39.7	2.0	1.6	1.3
P33226	TorC	Cell inner membrane	Monomer	96.9	81.8	63.2	6.5	5.5	4.2
			Complex with SecB	66.8	54.5	43.6	2.5	2.1	1.6
			Complex with TF (Tig)	72.3	61.1	47.2	3.1	2.6	2.0
P36662	TorD	Cytoplasm	Monomer	125.4	105.9	81.8	11.8	10.0	7.7
Q2EES9	TorI		Monomer	191.3	161.7	124.9	28.8	24.4	18.8
P38684	TorR	Cytoplasm	Monomer	118.2	99.9	77.1	10.3	8.7	6.8
P39453	TorS	Cell inner membrane	Monomer	69.7	58.9	45.5	2.8	2.4	1.8
			Complex with SecB	56.8	46.4	37.1	1.6	1.3	1.0
			Complex with TF (Tig)	59.8	50.5	39.0	1.9	1.6	1.2
P38683	TorT	Periplasm	Monomer	102.4	86.5	66.8	7.4	6.3	4.8
			Complex with SecB	68.1	55.7	44.5	2.7	2.2	1.7
			Complex with TF (Tig)	74.2	62.7	48.4	3.3	2.8	2.2
P52005	TorY	Cell inner membrane	Monomer	99.9	84.4	65.2	7.0	5.9	4.6
			Complex with SecB	67.5	55.2	44.1	2.6	2.1	1.7
			Complex with TF (Tig)	73.4	62.0	47.9	3.2	2.7	2.1
P46923	TorZ	Periplasm	Monomer	73.2	61.9	47.8	3.2	2.7	2.1

UniProtKB A. N.	Protein name	Subcellular location	Subunit	D_0 $\left(\frac{\mu m^2}{s}\right)$			D_{cyto} $\left(\frac{\mu m^2}{s}\right)$		
				310 K	303 K	293 K	310 K	303 K	293 K
			Complex with SecB	58.5	47.8	38.2	1.7	1.4	1.1
			Complex with TF (Tig)	61.8	52.2	40.3	2.0	1.7	1.3
P0A858	TpiA	Cytoplasm	Monomer	116.9	98.8	76.3	10.1	8.5	6.6
			Homodimer	89.1	72.8	58.1	5.3	4.3	3.5
P02338	Tpr		Monomer	261.9	221.3	170.9	52.6	44.4	34.3
P0A862	Tpx	Periplasm	Monomer	137.5	116.2	89.7	14.5	12.2	9.5
			Complex with SecB	73.9	60.4	48.2	3.3	2.7	2.1
			Complex with TF (Tig)	82.3	69.5	53.7	4.3	3.7	2.8
P0AFS5	TqsA	Cell inner membrane	Monomer	102.7	86.8	67.0	7.5	6.3	4.9
			Complex with SecB	68.2	55.7	44.5	2.7	2.2	1.7
			Complex with TF (Tig)	74.3	62.8	48.5	3.3	2.8	2.2
P13482	TreA	Periplasm	Monomer	83.5	70.6	54.5	4.5	3.8	2.9
			Complex with SecB	62.6	51.1	40.9	2.1	1.7	1.4
			Complex with TF (Tig)	67.0	56.6	43.7	2.5	2.1	1.7
P36672	TreB	Cell inner membrane	Monomer	91.0	76.9	59.4	5.6	4.7	3.6
			Complex with SecB	65.1	53.2	42.5	2.3	1.9	1.5
			Complex with TF (Tig)	70.2	59.3	45.8	2.9	2.4	1.9
P28904	TreC	Cytoplasm	Monomer	83.4	70.5	54.4	4.5	3.8	2.9
P62601	TreF	Cytoplasm	Monomer	83.5	70.5	54.5	4.5	3.8	2.9
P36673	TreR		Monomer	106.1	89.7	69.3	8.1	6.8	5.3
			Homodimer	80.9	66.1	52.8	4.2	3.4	2.7
P05704	Trg	Cell inner membrane	Monomer	86.1	72.7	56.2	4.9	4.1	3.2
			Complex with SecB	63.5	51.9	41.4	2.2	1.8	1.4
			Complex with TF (Tig)	68.1	57.5	44.4	2.7	2.2	1.7
P0AGI8	TrkA	Cell inner membrane	Monomer	91.5	77.3	59.7	5.7	4.8	3.7
P23849	TrkG	Cell inner membrane	Monomer	89.1	75.3	58.1	5.3	4.5	3.5
			Complex with SecB	64.5	52.7	42.1	2.3	1.9	1.5
			Complex with TF (Tig)	69.4	58.6	45.3	2.8	2.4	1.8
P0AFZ7	TrkH	Cell inner membrane	Monomer	89.8	75.8	58.6	5.4	4.6	3.5
			Complex with SecB	64.7	52.9	42.2	2.3	1.9	1.5
			Complex with TF (Tig)	69.6	58.8	45.4	2.8	2.4	1.8

UniProtKB A. N.	Protein name	Subcellular location	Subunit	D_0 $\left(\frac{\mu m^2}{s}\right)$			D_{cyto} $\left(\frac{\mu m^2}{s}\right)$		
				310 K	303 K	293 K	310 K	303 K	293 K
P23003	TrmA		Monomer	98.3	83.1	64.2	6.7	5.7	4.4
P0A8I5	TrmB		Monomer	116.4	98.3	75.9	10.0	8.4	6.5
P0A873	TrmD	Cytoplasm	Monomer	114.6	96.8	74.8	9.6	8.1	6.3
			Homodimer	87.3	71.3	57.0	5.0	4.1	3.3
P0AGJ2	TrmH	Cytoplasm	Monomer	119.8	101.2	78.2	10.7	9.0	7.0
P0AE01	TrmJ	Cytoplasm	Monomer	116.8	98.7	76.2	10.1	8.5	6.6
			Homodimer	89.0	72.7	58.1	5.3	4.3	3.4
P0AGJ7	TrmL	Cytoplasm	Monomer	137.8	116.4	89.9	14.6	12.3	9.5
			Homodimer	105.0	85.8	68.5	7.9	6.4	5.1
P0A877	TrpA		Monomer	114.1	96.4	74.4	9.5	8.1	6.2
P0A879	TrpB		Monomer	97.4	82.3	63.6	6.6	5.6	4.3
P00909	TrpC		Monomer	92.3	77.9	60.2	5.8	4.9	3.8
P00904	TrpD		Monomer	87.3	73.7	57.0	5.0	4.3	3.3
P00895	TrpE		Monomer	86.9	73.4	56.7	5.0	4.2	3.2
P77766	TrpH		Monomer	108.6	91.7	70.9	8.5	7.2	5.6
P0AD92	TrpL		Monomer	343.7	290.4	224.3	85.4	72.2	55.7
P0A881	TrpR	Cytoplasm	Monomer	158.8	134.2	103.6	19.7	16.6	12.9
			Homodimer	121.0	98.9	79.0	10.9	8.9	7.1
P00954	TrpS	Cytoplasm	Monomer	102.8	86.9	67.1	7.5	6.3	4.9
			Homodimer	78.4	64.0	51.1	3.8	3.1	2.5
P07649	TruA		Monomer	111.6	94.3	72.8	9.1	7.7	5.9
			Homodimer	85.0	69.5	55.5	4.7	3.9	3.1
P60340	TruB		Monomer	105.5	89.1	68.8	8.0	6.7	5.2
P0AA41	TruC		Monomer	112.6	95.2	73.5	9.3	7.8	6.0
Q57261	TruD		Monomer	101.1	85.4	66.0	7.2	6.1	4.7
P0AA25	TrxA		Monomer	161.6	136.6	105.5	20.4	17.3	13.3
P0A9P4	TrxB	Cytoplasm	Monomer	106.0	89.6	69.2	8.0	6.8	5.3
			Homodimer	80.8	66.0	52.7	4.1	3.4	2.7
P0AGG4	TrxC	Cytoplasm	Monomer	145.1	122.6	94.7	16.3	13.7	10.6
P0A6P1	Tsf	Cytoplasm	Monomer	111.5	94.2	72.8	9.1	7.7	5.9
P60778	TsgA	Cell inner membrane	Monomer	97.2	82.2	63.5	6.6	5.5	4.3

UniProtKB A. N.	Protein name	Subcellular location	Subunit	D_0 $\left(\frac{\mu m^2}{s}\right)$			D_{cyto} $\left(\frac{\mu m^2}{s}\right)$		
				310 K	303 K	293 K	310 K	303 K	293 K
			Complex with SecB	66.9	54.6	43.6	2.5	2.1	1.6
			Complex with TF (Tig)	72.5	61.2	47.3	3.1	2.6	2.0
P02942	Tsr	Cell inner membrane	Monomer	85.8	72.5	56.0	4.8	4.1	3.1
			Complex with SecB	63.4	51.8	41.4	2.2	1.8	1.4
			Complex with TF (Tig)	68.0	57.4	44.4	2.6	2.2	1.7
P0A927	Tsx	Cell outer membrane	Monomer	107.3	90.6	70.0	8.3	7.0	5.4
			Complex with SecB	69.2	56.6	45.2	2.8	2.3	1.8
			Complex with TF (Tig)	75.7	63.9	49.4	3.5	3.0	2.3
P76055	TtcA	Cytoplasm	Monomer	104.9	88.6	68.5	7.9	6.6	5.1
P05847	TtdA		Monomer	108.4	91.6	70.7	8.5	7.2	5.5
			Heterotetramer ($TtdA_2-TtdB_2$)	67.2	56.8	43.8	2.6	2.2	1.7
P0AC35	TtdB		Monomer	125.1	105.7	81.7	11.8	9.9	7.7
			Heterotetramer ($TtdA_2-TtdB_2$)	67.2	56.8	43.8	2.6	2.2	1.7
P45463	TtdR		Monomer	105.2	88.9	68.7	7.9	6.7	5.2
P39414	TtdT	Cell inner membrane	Monomer	89.8	75.9	58.6	5.4	4.6	3.5
			Complex with SecB	64.7	52.9	42.2	2.3	1.9	1.5
			Complex with TF (Tig)	69.7	58.8	45.5	2.8	2.4	1.8
P0CE47	TufA	Cytoplasm. Cell inner membrane	Monomer	97.1	82.1	63.4	6.5	5.5	4.3
P0CE48	TufB	Cytoplasm. Cell inner membrane	Monomer	97.1	82.0	63.4	6.5	5.5	4.3
P16525	Tus	Cytoplasm	Monomer	104.7	88.4	68.3	7.8	6.6	5.1
P0A890	TusA	Cytoplasm	Monomer	179.1	151.3	116.8	25.2	21.3	16.5
P45530	TusB	Cytoplasm	Monomer	167.9	141.8	109.6	22.1	18.7	14.4
			Heterohexamer, ($(TusB-TusC-TusD)_2$)	78.4	66.2	51.2	3.8	3.2	2.5
P45531	TusC	Cytoplasm	Monomer	155.4	131.3	101.4	18.8	15.9	12.3
			Heterohexamer, ($(TusB-TusC-TusD)_2$)	78.4	66.2	51.2	3.8	3.2	2.5
P45532	TusD	Cytoplasm	Monomer	152.7	129.0	99.7	18.2	15.3	11.8
			Heterohexamer, ($(TusB-TusC-TusD)_2$)	78.4	66.2	51.2	3.8	3.2	2.5
P0AB18	TusE	Cytoplasm	Monomer	158.5	133.9	103.4	19.6	16.6	12.8
P46883	TynA	Periplasm	Monomer	74.8	63.2	48.8	3.4	2.9	2.2

UniProtKB A. N.	Protein name	Subcellular location	Subunit	D_0 $\left(\frac{\mu m^2}{s}\right)$			D_{cyto} $\left(\frac{\mu m^2}{s}\right)$		
				310 K	303 K	293 K	310 K	303 K	293 K
			Complex with SecB	59.1	48.3	38.6	1.8	1.5	1.2
			Complex with TF (Tig)	62.6	52.9	40.9	2.1	1.8	1.4
P32132	TypA		Monomer	81.7	69.0	53.3	4.3	3.6	2.8
P07023	TyrA	Cytoplasm	Monomer	98.3	83.0	64.1	6.7	5.7	4.4
P04693	TyrB	Cytoplasm	Monomer	96.9	81.9	63.2	6.5	5.5	4.2
			Homodimer	73.9	60.3	48.2	3.3	2.7	2.1
P0AAD4	TyrP	Cell inner membrane	Monomer	97.6	82.4	63.7	6.6	5.6	4.3
			Complex with SecB	66.9	54.7	43.7	2.5	2.1	1.7
			Complex with TF (Tig)	72.6	61.3	47.4	3.1	2.7	2.0
P07604	TyrR		Monomer	86.8	73.3	56.6	5.0	4.2	3.2
			Homodimer	66.2	54.0	43.2	2.5	2.0	1.6
P0AGJ9	TyrS	Cytoplasm	Monomer	93.6	79.1	61.1	6.0	5.1	3.9
			Homodimer	71.4	58.3	46.6	3.0	2.5	2.0
P0AGK1	UbiA	Cell inner membrane	Monomer	108.7	91.8	70.9	8.5	7.2	5.6
			Complex with SecB	69.5	56.8	45.4	2.8	2.3	1.8
			Complex with TF (Tig)	76.1	64.3	49.7	3.6	3.0	2.3
P0A6A0	UbiB	Cell inner membrane	Monomer	83.7	70.7	54.6	4.5	3.8	3.0
			Complex with SecB	62.7	51.2	40.9	2.1	1.7	1.4
			Complex with TF (Tig)	67.1	56.7	43.8	2.5	2.1	1.7
P26602	UbiC	Cytoplasm	Monomer	134.8	113.9	87.9	13.9	11.7	9.0
P0AAB4	UbiD	Cell membrane	Monomer	88.1	74.4	57.5	5.1	4.3	3.4
			Complex with SecB	64.2	52.4	41.9	2.3	1.8	1.5
			Complex with TF (Tig)	68.9	58.2	45.0	2.7	2.3	1.8
P0A887	UbiE		Monomer	115.1	97.2	75.1	9.7	8.2	6.4
P75728	UbiF		Monomer	97.4	82.3	63.6	6.6	5.6	4.3
P17993	UbiG		Monomer	117.6	99.4	76.8	10.2	8.6	6.7
			Homodimer	89.7	73.2	58.5	5.4	4.4	3.5
P25534	UbiH		Monomer	98.0	82.8	64.0	6.7	5.6	4.4
P0AG03	UbiX		Monomer	129.7	109.6	84.7	12.7	10.8	8.3
P37440	UcpA		Monomer	115.5	97.5	75.3	9.8	8.3	6.4
P0A8F4	Udk	Cytoplasm	Monomer	121.7	102.8	79.4	11.1	9.3	7.2

UniProtKB A. N.	Protein name	Subcellular location	Subunit	D_0 $\left(\frac{\mu m^2}{s}\right)$			D_{cyto} $\left(\frac{\mu m^2}{s}\right)$		
				310 K	303 K	293 K	310 K	303 K	293 K
			Homotetramer	70.7	57.7	46.1	2.9	2.4	1.9
P12758	Udp	Cytoplasm	Monomer	116.6	98.5	76.1	10.0	8.5	6.5
			Homohexamer	57.8	47.2	37.7	1.7	1.4	1.1
P76373	Ugd		Monomer	96.8	81.8	63.2	6.5	5.5	4.2
P10905	UgpA	Cell inner membrane	Monomer	107.7	91.0	70.3	8.4	7.1	5.4
			Complex with SecB	69.3	56.6	45.2	2.8	2.3	1.8
			Complex with TF (Tig)	75.8	64.0	49.5	3.5	3.0	2.3
P0AG80	UgpB	Periplasm	Monomer	92.9	78.5	60.6	5.9	5.0	3.8
			Complex with SecB	65.7	53.6	42.8	2.4	2.0	1.6
			Complex with TF (Tig)	70.9	59.9	46.3	3.0	2.5	1.9
P10907	UgpC	Cell inner membrane	Monomer	100.7	85.0	65.7	7.1	6.0	4.6
			Complex with SecB	67.7	55.3	44.2	2.6	2.1	1.7
			Complex with TF (Tig)	73.6	62.2	48.1	3.3	2.8	2.1
P10906	UgpE	Cell inner membrane	Monomer	110.0	93.0	71.8	8.8	7.4	5.7
			Complex with SecB	69.8	57.0	45.5	2.8	2.3	1.8
			Complex with TF (Tig)	76.5	64.6	49.9	3.6	3.0	2.3
P10908	UgpQ	Cytoplasm	Monomer	116.2	98.2	75.8	9.9	8.4	6.5
P0AGA6	UhpA	Cytoplasm	Monomer	129.2	109.2	84.3	12.6	10.7	8.3
P09835	UhpB	Cell inner membrane	Monomer	87.6	74.0	57.2	5.1	4.3	3.3
			Complex with SecB	64.0	52.3	41.8	2.2	1.8	1.5
			Complex with TF (Tig)	68.8	58.1	44.9	2.7	2.3	1.8
P09836	UhpC	Cell inner membrane	Monomer	93.1	78.6	60.7	5.9	5.0	3.8
			Complex with SecB	65.7	53.7	42.9	2.4	2.0	1.6
			Complex with TF (Tig)	71.0	59.9	46.3	3.0	2.5	1.9
P0AGC0	UhpT	Cell inner membrane	Monomer	91.4	77.2	59.6	5.6	4.8	3.7
			Complex with SecB	65.2	53.3	42.5	2.4	1.9	1.5
			Complex with TF (Tig)	70.3	59.4	45.9	2.9	2.4	1.9
P05804	UidA		Monomer	81.2	68.6	53.0	4.2	3.5	2.7
			Homotetramer	47.1	38.5	30.8	0.9	0.8	0.6
P0CE44	UidB	Cell inner membrane	Monomer	91.9	77.6	59.9	5.7	4.8	3.7
			Complex with SecB	65.3	53.4	42.6	2.4	1.9	1.5

UniProtKB A. N.	Protein name	Subcellular location	Subunit	D_0 $\left(\frac{\mu m^2}{s}\right)$			D_{cyto} $\left(\frac{\mu m^2}{s}\right)$		
				310 K	303 K	293 K	310 K	303 K	293 K
			Complex with TF (Tig)	70.5	59.5	46.0	2.9	2.5	1.9
Q47706	UidC	Cell outer membrane	Monomer	94.3	79.7	61.6	6.1	5.1	4.0
			Complex with SecB	66.1	54.0	43.1	2.4	2.0	1.6
			Complex with TF (Tig)	71.4	60.3	46.6	3.0	2.5	2.0
P0ACT6	UidR		Monomer	127.1	107.4	82.9	12.2	10.3	8.0
P39301	UlaA	Cell inner membrane	Monomer	91.3	77.1	59.6	5.6	4.7	3.7
			Complex with SecB	65.2	53.2	42.5	2.4	1.9	1.5
			Complex with TF (Tig)	70.3	59.4	45.8	2.9	2.4	1.9
P69822	UlaB	Cytoplasm	Monomer	166.8	140.9	108.9	21.8	18.4	14.2
P69820	UlaC	Cytoplasm	Monomer	139.4	117.7	90.9	14.9	12.6	9.7
P39304	UlaD		Monomer	123.3	104.1	80.4	11.4	9.6	7.4
			Homodimer	93.9	76.7	61.3	6.0	4.9	3.9
P39305	UlaE		Monomer	109.3	92.4	71.3	8.6	7.3	5.6
P39306	UlaF		Monomer	119.9	101.3	78.3	10.7	9.0	7.0
P39300	UlaG	Cytoplasm	Monomer	100.1	84.6	65.3	7.0	5.9	4.6
P0A9W0	UlaR	Cytoplasm	Monomer	115.9	97.9	75.6	9.9	8.4	6.5
P04152	UmuC		Monomer	93.5	79.0	61.0	6.0	5.0	3.9
P0AG11	UmuD		Monomer	146.9	124.1	95.9	16.7	14.1	10.9
P12295	Ung	Cytoplasm	Monomer	119.2	100.7	77.8	10.5	8.9	6.9
A8DYP9	Uof		Monomer	272.7	230.4	178.0	56.6	47.8	37.0
P0A8F0	Upp		Monomer	125.5	106.0	81.9	11.8	10.0	7.7
			Homodimer	95.6	78.1	62.4	6.3	5.1	4.1
			Homotrimer	81.6	68.9	53.2	4.2	3.6	2.8
P60932	UppP	Cell inner membrane	Monomer	112.5	95.0	73.4	9.2	7.8	6.0
			Complex with SecB	70.3	57.4	45.9	2.9	2.4	1.9
			Complex with TF (Tig)	77.1	65.2	50.3	3.7	3.1	2.4
P60472	UppS		Monomer	114.5	96.7	74.7	9.6	8.1	6.3
			Homodimer	87.3	71.3	56.9	5.0	4.1	3.3
P0AGM7	UraA	Cell inner membrane	Monomer	95.6	80.8	62.4	6.3	5.3	4.1
			Complex with SecB	66.4	54.3	43.3	2.5	2.0	1.6
			Complex with TF (Tig)	71.9	60.7	46.9	3.1	2.6	2.0

UniProtKB A. N.	Protein name	Subcellular location	Subunit	$D_0\left(\frac{\mu m^2}{s}\right)$			$D_{\text{cyto}}\left(\frac{\mu m^2}{s}\right)$		
				310 K	303 K	293 K	310 K	303 K	293 K
P08390	Usg		Monomer	104.0	87.9	67.9	7.7	6.5	5.0
P07024	UshA	Periplasm	Monomer	85.0	71.8	55.5	4.7	4.0	3.1
			Complex with SecB	63.1	51.6	41.2	2.2	1.8	1.4
			Complex with TF (Tig)	67.6	57.1	44.1	2.6	2.2	1.7
P0AED0	UspA	Cytoplasm	Monomer	143.3	121.0	93.5	15.8	13.4	10.3
			Homodimer	109.2	89.2	71.2	8.6	7.0	5.6
P0A8S5	UspB	Cell inner membrane	Monomer	155.5	131.4	101.5	18.9	15.9	12.3
			Complex with SecB	75.6	61.7	49.3	3.5	2.8	2.3
			Complex with TF (Tig)	84.8	71.6	55.3	4.7	4.0	3.1
P46888	UspC	Cytoplasm	Monomer	144.8	122.3	94.5	16.2	13.7	10.6
P0AAB8	UspD	Cytoplasm	Monomer	142.5	120.4	93.0	15.6	13.2	10.2
P0AAC0	UspE	Cytoplasm	Monomer	104.7	88.5	68.4	7.8	6.6	5.1
P37903	UspF		Monomer	143.4	121.2	93.6	15.9	13.4	10.4
			Homodimer	109.3	89.3	71.3	8.6	7.1	5.6
P39177	UspG		Monomer	143.7	121.4	93.8	15.9	13.5	10.4
P43672	Uup		Monomer	79.5	67.2	51.9	4.0	3.4	2.6
P0A698	UvrA	Cytoplasm	Monomer	68.9	58.2	45.0	2.7	2.3	1.8
			Heterotetramer ($UvrA_2-UvrB_2$)	42.3	35.8	27.6	0.7	0.6	0.4
P0A8F8	UvrB	Cytoplasm	Monomer	77.8	65.7	50.8	3.8	3.2	2.5
			Heterotetramer ($UvrA_2-UvrB_2$)	42.3	35.8	27.6	0.7	0.6	0.4
P0A8G0	UvrC	Cytoplasm	Monomer	81.3	68.7	53.0	4.2	3.6	2.7
P03018	UvrD		Monomer	75.6	63.9	49.3	3.5	3.0	2.3
P0AED5	UvrY	Cytoplasm	Monomer	122.6	103.6	80.0	11.2	9.5	7.3
P42604	UxaA		Monomer	89.0	75.2	58.1	5.3	4.5	3.4
P0A6L7	UxaB		Monomer	88.6	74.8	57.8	5.2	4.4	3.4
P0A8G3	UxaC		Monomer	89.1	75.3	58.1	5.3	4.5	3.5
P24215	UxuA		Monomer	95.8	80.9	62.5	6.3	5.3	4.1
P39160	UxuB		Monomer	89.3	75.5	58.3	5.3	4.5	3.5
P39161	UxuR		Monomer	113.2	95.6	73.9	9.4	7.9	6.1
P07118	ValS	Cytoplasm	Monomer	67.8	57.3	44.3	2.6	2.2	1.7
P76214	Ves		Monomer	127.6	107.8	83.3	12.3	10.4	8.0

UniProtKB A. N.	Protein name	Subcellular location	Subunit	D_0 $\left(\frac{\mu m^2}{s}\right)$			D_{cyto} $\left(\frac{\mu m^2}{s}\right)$		
				310 K	303 K	293 K	310 K	303 K	293 K
P0ADN0	ViaA		Monomer	87.9	74.2	57.3	5.1	4.3	3.3
P25535	VisC		Monomer	96.3	81.4	62.8	6.4	5.4	4.2
P09184	Vsr		Monomer	137.0	115.7	89.4	14.4	12.1	9.4
P0AC75	WaaA	Cell inner membrane	Monomer	93.8	79.3	61.2	6.0	5.1	3.9
			Complex with SecB	65.9	53.8	43.0	2.4	2.0	1.6
			Complex with TF (Tig)	71.2	60.2	46.5	3.0	2.5	2.0
P27242	WaaU	Membrane	Monomer	98.5	83.2	64.3	6.8	5.7	4.4
			Complex with SecB	67.2	54.9	43.8	2.6	2.1	1.7
			Complex with TF (Tig)	72.9	61.6	47.6	3.2	2.7	2.1
P37750	WbbJ		Monomer	127.4	107.6	83.1	12.2	10.3	8.0
P36667	WbbL		Monomer	110.6	93.5	72.2	8.9	7.5	5.8
P77414	WcaA		Monomer	108.0	91.2	70.5	8.4	7.1	5.5
P0ACC9	WcaB		Monomer	138.2	116.7	90.2	14.6	12.4	9.6
P71237	WcaC		Monomer	95.5	80.6	62.3	6.3	5.3	4.1
P71238	WcaD	Cell inner membrane	Monomer	95.3	80.5	62.2	6.2	5.3	4.1
			Complex with SecB	66.3	54.2	43.3	2.5	2.0	1.6
			Complex with TF (Tig)	71.8	60.7	46.8	3.1	2.6	2.0
P71239	WcaE		Monomer	115.4	97.5	75.3	9.8	8.3	6.4
P0ACD2	WcaF		Monomer	131.6	111.2	85.9	13.2	11.1	8.6
P32057	WcaI		Monomer	95.7	80.9	62.5	6.3	5.3	4.1
P71241	WcaJ	Cell inner membrane	Monomer	90.1	76.1	58.8	5.4	4.6	3.6
			Complex with SecB	64.8	52.9	42.3	2.3	1.9	1.5
			Complex with TF (Tig)	69.8	59.0	45.5	2.8	2.4	1.8
P71242	WcaK		Monomer	93.8	79.2	61.2	6.0	5.1	3.9
P71243	WcaL		Monomer	95.4	80.6	62.2	6.3	5.3	4.1
P71244	WcaM		Monomer	90.9	76.8	59.3	5.6	4.7	3.6
P0AC78	WecA	Cell inner membrane	Monomer	99.3	83.9	64.8	6.9	5.8	4.5
			Complex with SecB	67.4	55.0	44.0	2.6	2.1	1.7
			Complex with TF (Tig)	73.2	61.8	47.8	3.2	2.7	2.1
P27828	WecB	Cytoplasm	Monomer	98.1	82.8	64.0	6.7	5.7	4.4
			Homodimer	74.7	61.1	48.8	3.4	2.8	2.2

UniProtKB A. N.	Protein name	Subcellular location	Subunit	D_0 $\left(\frac{\mu m^2}{s}\right)$ 310 K	D_0 303 K	D_0 293 K	D_{cyto} $\left(\frac{\mu m^2}{s}\right)$ 310 K	D_{cyto} 303 K	D_{cyto} 293 K
P27829	WecC		Monomer	95.0	80.2	62.0	6.2	5.2	4.0
P56258	WecF	Cell inner membrane	Monomer	99.6	84.1	65.0	6.9	5.9	4.5
			Complex with SecB	67.5	55.1	44.0	2.6	2.1	1.7
			Complex with TF (Tig)	73.3	61.9	47.8	3.2	2.7	2.1
P27836	WecG		Monomer	115.3	97.4	75.3	9.8	8.3	6.4
P0A8G6	WrbA		Monomer	129.4	109.3	84.4	12.7	10.7	8.3
			Homodimer	98.6	80.5	64.3	6.8	5.5	4.4
			Homotetramer	75.1	63.5	49.0	3.4	2.9	2.2
P0A930	Wza	Cell outer membrane	Monomer	98.4	83.1	64.2	6.7	5.7	4.4
			Complex with SecB	67.1	54.9	43.8	2.6	2.1	1.7
			Complex with TF (Tig)	72.9	61.6	47.6	3.2	2.7	2.1
P0AAB2	Wzb		Monomer	141.1	119.2	92.1	15.3	12.9	10.0
P76387	Wzc	Cell inner membrane	Monomer	76.6	64.7	50.0	3.6	3.1	2.4
			Complex with SecB	59.9	49.0	39.1	1.9	1.5	1.2
			Complex with TF (Tig)	63.6	53.7	41.5	2.2	1.9	1.4
P77377	WzxC	Cell inner membrane	Monomer	89.3	75.4	58.3	5.3	4.5	3.5
			Complex with SecB	64.5	52.7	42.1	2.3	1.9	1.5
			Complex with TF (Tig)	69.4	58.7	45.3	2.8	2.4	1.8
P0AAA7	WzxE	Cell inner membrane	Monomer	95.7	80.9	62.5	6.3	5.3	4.1
			Complex with SecB	66.4	54.3	43.4	2.5	2.0	1.6
			Complex with TF (Tig)	71.9	60.8	46.9	3.1	2.6	2.0
P27835	WzyE	Cell membrane	Monomer	90.7	76.6	59.2	5.5	4.7	3.6
			Complex with SecB	65.0	53.1	42.4	2.3	1.9	1.5
			Complex with TF (Tig)	70.0	59.2	45.7	2.9	2.4	1.9
P76372	WzzB	Cell inner membrane	Monomer	103.9	87.8	67.8	7.7	6.5	5.0
			Complex with SecB	68.5	56.0	44.7	2.7	2.2	1.8
			Complex with TF (Tig)	74.7	63.1	48.7	3.4	2.9	2.2
P0AG00	WzzE	Cell inner membrane	Monomer	100.7	85.1	65.7	7.1	6.0	4.6
			Complex with SecB	67.7	55.3	44.2	2.6	2.1	1.7
			Complex with TF (Tig)	73.7	62.2	48.1	3.3	2.8	2.1
P0AGM9	XanP	Cell inner membrane	Monomer	92.6	78.3	60.4	5.8	4.9	3.8

UniProtKB A. N.	Protein name	Subcellular location	Subunit	D_0 $\left(\frac{\mu m^2}{s}\right)$			D_{cyto} $\left(\frac{\mu m^2}{s}\right)$		
				310 K	303 K	293 K	310 K	303 K	293 K
			Complex with SecB	65.6	53.6	42.8	2.4	2.0	1.6
			Complex with TF (Tig)	70.8	59.8	46.2	2.9	2.5	1.9
P67444	XanQ	Cell inner membrane	Monomer	92.4	78.1	60.3	5.8	4.9	3.8
			Complex with SecB	65.5	53.5	42.8	2.4	2.0	1.6
			Complex with TF (Tig)	70.7	59.7	46.1	2.9	2.5	1.9
P45563	XapA		Monomer	112.4	95.0	73.3	9.2	7.8	6.0
P45562	XapB	Cell inner membrane	Monomer	94.7	80.0	61.8	6.2	5.2	4.0
			Complex with SecB	66.2	54.1	43.2	2.5	2.0	1.6
			Complex with TF (Tig)	71.6	60.5	46.7	3.0	2.6	2.0
P23841	XapR		Monomer	107.2	90.6	70.0	8.3	7.0	5.4
Q46799	XdhA		Monomer	75.9	64.1	49.5	3.5	3.0	2.3
			Heterotrimer (XdhA−XdhB−XdhC)	63.2	53.4	41.2	2.2	1.8	1.4
Q46800	XdhB		Monomer	109.9	92.9	71.7	8.8	7.4	5.7
			Heterotrimer (XdhA−XdhB−XdhC)	63.2	53.4	41.2	2.2	1.8	1.4
Q46801	XdhC		Monomer	140.4	118.6	91.6	15.2	12.8	9.9
			Heterotrimer (XdhA−XdhB−XdhC)	63.2	53.4	41.2	2.2	1.8	1.4
Q46814	XdhD		Monomer	69.0	58.3	45.0	2.7	2.3	1.8
P0A8P6	XerC	Cytoplasm	Monomer	106.9	90.3	69.8	8.2	6.9	5.4
			Heterotetramer ($XerC_2-XerD_2$)	62.0	52.4	40.4	2.1	1.7	1.3
P0A8P8	XerD	Cytoplasm	Monomer	106.5	90.0	69.5	8.1	6.9	5.3
			Heterotetramer ($XerC_2-XerD_2$)	62.0	52.4	40.4	2.1	1.7	1.3
P75970	XisE		Monomer	177.6	150.0	115.9	24.8	21.0	16.2
P04994	XseA	Cytoplasm	Monomer	90.5	76.5	59.1	5.5	4.7	3.6
			Heterooligomer ($(XseA-XseB_4)_2$)	56.1	47.4	36.6	1.6	1.3	1.0
P0A8G9	XseB	Cytoplasm	Monomer	180.2	152.2	117.6	25.5	21.6	16.7
			Heterooligomer ($(XseA-XseB_4)_2$)	56.1	47.4	36.6	1.6	1.3	1.0
P09030	XthA		Monomer	110.8	93.6	72.3	8.9	7.5	5.8
P00944	XylA	Cytoplasm	Monomer	92.0	77.7	60.0	5.7	4.8	3.7
			Homotetramer	53.4	43.6	34.9	1.3	1.1	0.9
P09099	XylB		Monomer	90.0	76.0	58.7	5.4	4.6	3.5
P0AGF4	XylE	Cell inner membrane	Monomer	89.3	75.5	58.3	5.3	4.5	3.5

UniProtKB A. N.	Protein name	Subcellular location	Subunit	D_0 $\left(\frac{\mu m^2}{s}\right)$			D_{cyto} $\left(\frac{\mu m^2}{s}\right)$		
				310 K	303 K	293 K	310 K	303 K	293 K
			Complex with SecB	64.6	52.7	42.1	2.3	1.9	1.5
			Complex with TF (Tig)	69.5	58.7	45.3	2.8	2.4	1.8
P37387	XylF	Periplasm	Monomer	104.7	88.5	68.3	7.8	6.6	5.1
			Complex with SecB	68.7	56.1	44.8	2.7	2.2	1.8
			Complex with TF (Tig)	74.9	63.3	48.9	3.4	2.9	2.2
P37388	XylG	Cell inner membrane	Monomer	87.5	73.9	57.1	5.1	4.3	3.3
			Complex with SecB	64.0	52.3	41.8	2.2	1.8	1.5
			Complex with TF (Tig)	68.7	58.1	44.8	2.7	2.3	1.8
P0AGI4	XylH	Cell inner membrane	Monomer	99.2	83.8	64.7	6.9	5.8	4.5
			Complex with SecB	67.4	55.0	44.0	2.6	2.1	1.7
			Complex with TF (Tig)	73.2	61.8	47.7	3.2	2.7	2.1
P0ACI3	XylR		Monomer	95.8	80.9	62.5	6.3	5.3	4.1
P0A8I3	YaaA		Monomer	112.8	95.3	73.6	9.3	7.8	6.1
P0AC98	YaaH	Cell inner membrane	Monomer	131.3	110.9	85.7	13.1	11.1	8.5
			Complex with SecB	73.2	59.8	47.7	3.2	2.6	2.1
			Complex with TF (Tig)	81.3	68.6	53.0	4.2	3.6	2.7
P28696	YaaI		Monomer	149.2	126.1	97.4	17.3	14.6	11.3
P30143	YaaJ	Cell inner membrane	Monomer	90.6	76.6	59.1	5.5	4.7	3.6
			Complex with SecB	65.0	53.1	42.4	2.3	1.9	1.5
			Complex with TF (Tig)	70.0	59.1	45.7	2.9	2.4	1.9
P31679	YaaU	Cell inner membrane	Monomer	92.8	78.4	60.5	5.8	4.9	3.8
			Complex with SecB	65.6	53.6	42.8	2.4	2.0	1.6
			Complex with TF (Tig)	70.8	59.8	46.2	2.9	2.5	1.9
P75617	YaaW		Monomer	117.5	99.2	76.6	10.2	8.6	6.7
P75616	YaaX		Monomer	164.1	138.7	107.1	21.1	17.8	13.8
P75620	YaaY		Monomer	189.3	159.9	123.5	28.2	23.8	18.4
P30149	YabI	Cell inner membrane	Monomer	114.8	97.0	74.9	9.7	8.2	6.3
			Complex with SecB	70.7	57.8	46.1	2.9	2.4	1.9
			Complex with TF (Tig)	77.7	65.7	50.7	3.8	3.2	2.4
P39220	YabP		Monomer	120.9	102.1	78.9	10.9	9.2	7.1
P39221	YabQ		Monomer	214.5	181.2	140.0	36.1	30.5	23.5

UniProtKB A. N.	Protein name	Subcellular location	Subunit	D_0 $\left(\frac{\mu m^2}{s}\right)$			D_{cyto} $\left(\frac{\mu m^2}{s}\right)$		
				310 K	303 K	293 K	310 K	303 K	293 K
P0AA95	YacC		Monomer	156.3	132.1	102.0	19.1	16.1	12.4
P36680	YacF		Monomer	114.8	96.9	74.9	9.7	8.2	6.3
P0A8H8	YacG		Monomer	195.1	164.8	127.3	30.0	25.3	19.6
P36682	YacH		Monomer	80.7	68.2	52.7	4.1	3.5	2.7
P0A8E5	YacL		Monomer	151.4	127.9	98.8	17.8	15.1	11.6
P31058	YadC	Fimbrium	Monomer	95.8	80.9	62.5	6.3	5.3	4.1
			Complex with SecB	66.5	54.3	43.4	2.5	2.0	1.6
			Complex with TF (Tig)	71.9	60.8	46.9	3.1	2.6	2.0
P31665	YadD		Monomer	106.0	89.6	69.2	8.1	6.8	5.3
P31666	YadE		Monomer	94.6	79.9	61.7	6.1	5.2	4.0
P36879	YadG		Monomer	106.0	89.5	69.2	8.0	6.8	5.2
P0AFN6	YadH	Cell inner membrane	Monomer	114.4	96.6	74.7	9.6	8.1	6.3
			Complex with SecB	70.6	57.7	46.1	2.9	2.4	1.9
			Complex with TF (Tig)	77.6	65.6	50.7	3.7	3.2	2.4
P36881	YadI	Cytoplasm	Monomer	141.6	119.7	92.4	15.4	13.0	10.1
P37016	YadK		Monomer	128.7	108.7	84.0	12.5	10.6	8.2
P37017	YadL		Monomer	128.9	108.9	84.1	12.6	10.6	8.2
P37018	YadM		Monomer	130.7	110.4	85.3	13.0	10.9	8.5
P37050	YadN	Fimbrium	Monomer	131.2	110.8	85.6	13.1	11.0	8.5
			Complex with SecB	73.2	59.8	47.7	3.2	2.6	2.1
			Complex with TF (Tig)	81.2	68.6	53.0	4.2	3.5	2.7
P0AFP0	YadS	Cell inner membrane	Monomer	126.4	106.8	82.5	12.0	10.2	7.8
			Complex with SecB	72.5	59.2	47.3	3.1	2.6	2.0
			Complex with TF (Tig)	80.3	67.8	52.4	4.1	3.4	2.7
P28634	YaeB		Monomer	118.0	99.7	77.0	10.3	8.7	6.7
P37056	YaeF	Cell membrane	Monomer	112.3	94.9	73.3	9.2	7.8	6.0
			Complex with SecB	70.2	57.4	45.8	2.9	2.4	1.9
			Complex with TF (Tig)	77.1	65.1	50.3	3.7	3.1	2.4
P62768	YaeH		Monomer	146.8	124.0	95.8	16.7	14.1	10.9
P37049	YaeI		Monomer	112.4	94.9	73.3	9.2	7.8	6.0
P40711	YaeJ		Monomer	144.8	122.4	94.5	16.2	13.7	10.6

UniProtKB A. N.	Protein name	Subcellular location	Subunit	D_0 $\left(\frac{\mu m^2}{s}\right)$ 310 K	D_0 303 K	D_0 293 K	D_{cyto} $\left(\frac{\mu m^2}{s}\right)$ 310 K	D_{cyto} 303 K	D_{cyto} 293 K
P0A8K5	YaeP		Monomer	196.1	165.6	128.0	30.3	25.6	19.7
P0AA97	YaeQ		Monomer	129.3	109.2	84.4	12.7	10.7	8.3
P52096	YaeR		Monomer	148.2	125.2	96.7	17.0	14.4	11.1
			Monomer	72.7	61.4	47.5	3.2	2.7	2.1
P0A940	YaeT	Cell outer membrane	Complex with SecB	58.2	47.6	38.0	1.7	1.4	1.1
			Complex with TF (Tig)	61.5	52.0	40.2	2.0	1.7	1.3
P30864	YafC		Monomer	107.1	90.4	69.9	8.2	7.0	5.4
P0A8U2	YafD	Cytoplasm	Monomer	112.2	94.8	73.2	9.2	7.8	6.0
P30866	YafE		Monomer	124.5	105.2	81.2	11.6	9.8	7.6
Q2EEP9	YafF		Monomer	198.9	168.1	129.8	31.1	26.3	20.3
Q47147	YafJ		Monomer	114.2	96.5	74.5	9.6	8.1	6.2
P0AA99	YafK		Monomer	115.2	97.3	75.2	9.8	8.2	6.4
			Monomer	114.0	96.3	74.4	9.5	8.0	6.2
Q47151	YafL	Cell membrane	Complex with SecB	70.5	57.6	46.0	2.9	2.4	1.9
			Complex with TF (Tig)	77.5	65.5	50.6	3.7	3.1	2.4
Q47152	YafM		Monomer	131.4	111.0	85.7	13.1	11.1	8.6
Q47156	YafN		Monomer	164.8	139.2	107.6	21.3	18.0	13.9
Q47157	YafO		Monomer	145.3	122.8	94.8	16.3	13.8	10.7
Q47158	YafP		Monomer	138.4	116.9	90.3	14.7	12.4	9.6
Q47149	YafQ		Monomer	167.1	141.2	109.0	21.9	18.5	14.3
P75672	YafS		Monomer	116.5	98.4	76.0	10.0	8.5	6.5
			Monomer	112.7	95.2	73.6	9.3	7.8	6.1
P77339	YafT	Cell membrane	Complex with SecB	70.3	57.4	45.9	2.9	2.4	1.9
			Complex with TF (Tig)	77.2	65.2	50.4	3.7	3.1	2.4
			Monomer	159.9	135.1	104.3	20.0	16.9	13.0
P77354	YafU	Cell inner membrane	Complex with SecB	75.9	62.0	49.5	3.5	2.9	2.3
			Complex with TF (Tig)	85.3	72.0	55.7	4.7	4.0	3.1
Q47679	YafV		Monomer	113.8	96.1	74.2	9.5	8.0	6.2
Q47684	YafW		Monomer	160.9	136.0	105.0	20.3	17.1	13.2
P75676	YafX		Monomer	138.8	117.2	90.6	14.8	12.5	9.6
			Monomer	140.7	118.9	91.8	15.2	12.9	9.9
P77365	YafY	Cell membrane							

UniProtKB A. N.	Protein name	Subcellular location	Subunit	D_0 $\left(\frac{\mu m^2}{s}\right)$			D_{cyto} $\left(\frac{\mu m^2}{s}\right)$		
				310 K	303 K	293 K	310 K	303 K	293 K
			Complex with SecB	74.2	60.7	48.4	3.3	2.7	2.2
			Complex with TF (Tig)	82.8	70.0	54.0	4.4	3.7	2.9
P77206	YafZ		Monomer	110.6	93.5	72.2	8.9	7.5	5.8
P37007	YagA		Monomer	96.7	81.7	63.1	6.5	5.5	4.2
P37008	YagB		Monomer	154.8	130.8	101.0	18.7	15.8	12.2
P75682	YagE		Monomer	107.6	90.9	70.2	8.3	7.0	5.4
P77596	YagF		Monomer	80.7	68.2	52.7	4.1	3.5	2.7
P75683	YagG	Cell inner membrane	Monomer	91.3	77.2	59.6	5.6	4.8	3.7
			Complex with SecB	65.2	53.3	42.5	2.4	1.9	1.5
			Complex with TF (Tig)	70.3	59.4	45.9	2.9	2.4	1.9
P77713	YagH		Monomer	85.0	71.8	55.5	4.7	4.0	3.1
P77300	YagI		Monomer	115.5	97.6	75.4	9.8	8.3	6.4
P77169	YagJ		Monomer	115.5	97.5	75.3	9.8	8.3	6.4
P77657	YagK		Monomer	121.5	102.6	79.3	11.0	9.3	7.2
P77607	YagL		Monomer	116.4	98.4	76.0	10.0	8.4	6.5
P71296	YagM		Monomer	108.5	91.6	70.8	8.5	7.2	5.5
P71297	YagN		Monomer	144.1	121.8	94.1	16.0	13.6	10.5
P75684	YagP		Monomer	145.6	123.0	95.0	16.4	13.9	10.7
P77183	YagQ		Monomer	105.6	89.2	68.9	8.0	6.7	5.2
P77489	YagR		Monomer	77.1	65.1	50.3	3.7	3.1	2.4
			Heterorotrimer (YagR−YagS−YagT)	62.0	52.3	40.4	2.0	1.7	1.3
P77324	YagS		Monomer	107.0	90.4	69.8	8.2	6.9	5.4
			Heterorotrimer (YagR−YagS−YagT)	62.0	52.3	40.4	2.0	1.7	1.3
P77165	YagT		Monomer	121.7	102.8	79.4	11.1	9.3	7.2
			Heterorotrimer (YagR−YagS−YagT)	62.0	52.3	40.4	2.0	1.7	1.3
P0AAA1	YagU	Cell inner membrane	Monomer	124.5	105.2	81.3	11.6	9.8	7.6
			Complex with SecB	72.3	59.0	47.2	3.1	2.5	2.0
			Complex with TF (Tig)	79.9	67.5	52.2	4.0	3.4	2.6
P77263	YagV		Monomer	117.5	99.3	76.7	10.2	8.6	6.7
P77694	YagW		Monomer	85.5	72.2	55.8	4.8	4.0	3.1
P77802	YagX		Monomer	72.5	61.3	47.3	3.1	2.6	2.0

UniProtKB A. N.	Protein name	Subcellular location	Subunit	D_0 $\left(\frac{\mu m^2}{s}\right)$			D_{cyto} $\left(\frac{\mu m^2}{s}\right)$		
				310 K	303 K	293 K	310 K	303 K	293 K
P21514	YahA		Monomer	99.5	84.0	64.9	6.9	5.8	4.5
P77700	YahB		Monomer	105.7	89.3	69.0	8.0	6.8	5.2
P77219	YahC	Cell membrane	Monomer	139.1	117.5	90.8	14.9	12.6	9.7
			Complex with SecB	74.1	60.5	48.3	3.3	2.7	2.2
			Complex with TF (Tig)	82.6	69.8	53.9	4.4	3.7	2.9
P77736	YahD		Monomer	127.4	107.6	83.1	12.2	10.3	8.0
P77297	YahE		Monomer	109.0	92.1	71.1	8.6	7.3	5.6
P77187	YahF		Monomer	88.1	74.4	57.5	5.1	4.3	3.4
P77221	YahG	Cell membrane	Monomer	91.5	77.3	59.7	5.7	4.8	3.7
			Complex with SecB	65.2	53.3	42.6	2.4	1.9	1.5
			Complex with TF (Tig)	70.4	59.4	45.9	2.9	2.4	1.9
Q2EEQ3	YahH		Monomer	168.4	142.3	109.9	22.3	18.8	14.5
P77624	YahI		Monomer	106.9	90.3	69.7	8.2	6.9	5.4
P77554	YahJ		Monomer	91.4	77.2	59.6	5.6	4.8	3.7
P75691	YahK		Monomer	102.2	86.4	66.7	7.4	6.2	4.8
P77393	YahL		Monomer	109.6	92.6	71.5	8.7	7.4	5.7
P75692	YahM		Monomer	180.7	152.6	117.9	25.7	21.7	16.8
P75693	YahN	Cell membrane	Monomer	120.8	102.1	78.8	10.9	9.2	7.1
			Complex with SecB	71.7	58.6	46.8	3.0	2.5	2.0
			Complex with TF (Tig)	79.1	66.9	51.6	3.9	3.3	2.6
P75694	YahO	Periplasm	Monomer	173.2	146.3	113.0	23.6	19.9	15.4
			Complex with SecB	76.7	62.7	50.1	3.6	3.0	2.4
			Complex with TF (Tig)	86.6	73.1	56.5	4.9	4.2	3.2
P0AAN5	YaiA		Monomer	195.4	165.1	127.5	30.0	25.4	19.6
P0C037	YaiE		Monomer	171.0	144.4	111.6	23.0	19.4	15.0
P0A8D3	YaiI		Monomer	140.2	118.5	91.5	15.1	12.8	9.9
P51024	YaiL		Monomer	131.7	111.2	85.9	13.2	11.1	8.6
Q47534	YaiO		Monomer	113.6	96.0	74.1	9.4	8.0	6.2
Q47536	YaiP		Monomer	95.9	81.0	62.6	6.3	5.4	4.1
P71311	YaiS		Monomer	130.2	110.0	85.0	12.9	10.9	8.4
P77199	YaiT		Monomer	68.7	58.0	44.8	2.7	2.3	1.8

UniProtKB A. N.	Protein name	Subcellular location	Subunit	D_0 $\left(\frac{\mu m^2}{s}\right)$			D_{cyto} $\left(\frac{\mu m^2}{s}\right)$		
				310 K	303 K	293 K	310 K	303 K	293 K
P0AAP5	YaiV		Monomer	122.5	103.5	79.9	11.2	9.5	7.3
P77562	YaiW		Monomer	99.8	84.3	65.1	7.0	5.9	4.6
P75697	YaiX		Monomer	120.6	101.9	78.7	10.8	9.1	7.1
P0AAP7	YaiY	Cell inner membrane	Monomer	163.7	138.3	106.9	21.0	17.7	13.7
			Complex with SecB	76.2	62.2	49.7	3.6	2.9	2.3
			Complex with TF (Tig)	85.7	72.4	55.9	4.8	4.1	3.1
P0AAQ0	YaiZ	Cell membrane	Monomer	187.4	158.3	122.3	27.7	23.4	18.0
			Complex with SecB	77.4	63.3	50.5	3.7	3.0	2.4
			Complex with TF (Tig)	87.6	74.0	57.2	5.1	4.3	3.3
P0ADZ7	YajC	Cell inner membrane	Monomer	161.2	136.2	105.2	20.3	17.2	13.3
			Complex with SecB	76.0	62.1	49.6	3.5	2.9	2.3
			Complex with TF (Tig)	85.4	72.2	55.7	4.8	4.0	3.1
P0AAQ2	YajD		Monomer	154.0	130.1	100.5	18.5	15.6	12.0
P0ADA5	YajG	Cell membrane	Monomer	129.1	109.1	84.2	12.6	10.7	8.2
			Complex with SecB	72.9	59.5	47.6	3.2	2.6	2.1
			Complex with TF (Tig)	80.8	68.3	52.8	4.1	3.5	2.7
P46122	YajI	Cell membrane	Monomer	132.6	112.0	86.5	13.4	11.3	8.7
			Complex with SecB	73.3	59.9	47.9	3.2	2.6	2.1
			Complex with TF (Tig)	81.5	68.8	53.2	4.2	3.6	2.8
Q46948	YajL		Monomer	129.5	109.4	84.5	12.7	10.7	8.3
			Homodimer	98.7	80.6	64.4	6.8	5.5	4.4
P77735	YajO		Monomer	103.9	87.8	67.8	7.7	6.5	5.0
P0A8E7	YajQ		Monomer	136.0	114.9	88.7	14.1	11.9	9.2
P77726	YajR	Cell inner membrane	Monomer	92.7	78.3	60.5	5.8	4.9	3.8
			Complex with SecB	65.6	53.6	42.8	2.4	2.0	1.6
			Complex with TF (Tig)	70.8	59.8	46.2	2.9	2.5	1.9
P0AAQ6	YbaA		Monomer	154.2	130.3	100.6	18.5	15.6	12.1
P0A8B5	YbaB		Monomer	160.5	135.6	104.8	20.2	17.0	13.2
			Homodimer	122.3	99.9	79.8	11.2	9.1	7.3
P46890	YbaE		Monomer	82.8	70.0	54.1	4.4	3.7	2.9
P0AAR3	YbaK	Cytoplasm	Monomer	139.8	118.1	91.2	15.0	12.7	9.8

UniProtKB A. N.	Protein name	Subcellular location	Subunit	D_0 $\left(\frac{\mu m^2}{s}\right)$			D_{cyto} $\left(\frac{\mu m^2}{s}\right)$		
				310 K	303 K	293 K	310 K	303 K	293 K
P39830	YbaL	Cell inner membrane	Monomer	85.8	72.5	56.0	4.8	4.1	3.1
			Complex with SecB	63.4	51.8	41.4	2.2	1.8	1.4
			Complex with TF (Tig)	68.0	57.4	44.4	2.6	2.2	1.7
P45807	YbaM		Monomer	210.4	177.8	137.3	34.8	29.4	22.7
P0AAR5	YbaN	Cell inner membrane	Monomer	148.1	125.1	96.6	17.0	14.4	11.1
			Complex with SecB	74.9	61.2	48.9	3.4	2.8	2.2
			Complex with TF (Tig)	83.9	70.9	54.7	4.6	3.8	3.0
P0ACJ5	YbaO		Monomer	138.7	117.2	90.5	14.8	12.5	9.6
P77301	YbaP		Monomer	112.3	94.9	73.3	9.2	7.8	6.0
P0A9T6	YbaQ		Monomer	154.9	130.8	101.1	18.7	15.8	12.2
P77400	YbaT	Cell inner membrane	Monomer	95.1	80.4	62.1	6.2	5.3	4.1
			Complex with SecB	66.3	54.1	43.3	2.5	2.0	1.6
			Complex with TF (Tig)	71.7	60.6	46.8	3.0	2.6	2.0
P0AAR8	YbaV		Monomer	157.1	132.7	102.5	19.3	16.3	12.6
P77717	YbaY	Cell membrane	Monomer	133.0	112.3	86.8	13.5	11.4	8.8
			Complex with SecB	73.4	59.9	47.9	3.2	2.6	2.1
			Complex with TF (Tig)	81.6	68.9	53.2	4.2	3.6	2.8
P0AFP2	YbaZ		Monomer	149.3	126.2	97.4	17.3	14.6	11.3
P0A9T8	YbbA		Monomer	121.4	102.5	79.2	11.0	9.3	7.2
P33668	YbbC		Monomer	150.6	127.2	98.2	17.6	14.9	11.5
P33669	YbbD	Membrane	Monomer	172.2	145.4	112.3	23.3	19.7	15.2
			Complex with SecB	76.7	62.6	50.0	3.6	3.0	2.4
			Complex with TF (Tig)	86.5	73.0	56.4	4.9	4.2	3.2
P0AAS3	YbbJ	Cell inner membrane	Monomer	140.3	118.5	91.6	15.1	12.8	9.9
			Complex with SecB	74.2	60.6	48.4	3.3	2.7	2.2
			Complex with TF (Tig)	82.8	69.9	54.0	4.4	3.7	2.9
P77279	YbbL		Monomer	119.7	101.2	78.1	10.7	9.0	7.0
P77307	YbbM	Cell inner membrane	Monomer	115.0	97.1	75.0	9.7	8.2	6.3
			Complex with SecB	70.7	57.8	46.1	2.9	2.4	1.9
			Complex with TF (Tig)	77.8	65.7	50.7	3.8	3.2	2.5
P77395	YbbN		Monomer	109.6	92.6	71.5	8.7	7.4	5.7

UniProtKB A. N.	Protein name	Subcellular location	Subunit	D_0 $\left(\frac{\mu m^2}{s}\right)$			D_{cyto} $\left(\frac{\mu m^2}{s}\right)$		
				310 K	303 K	293 K	310 K	303 K	293 K
P0AFP4	YbbO		Monomer	113.0	95.5	73.8	9.3	7.9	6.1
P77504	YbbP	Cell membrane	Monomer	73.1	61.8	47.7	3.2	2.7	2.1
			Complex with SecB	58.4	47.7	38.1	1.7	1.4	1.1
			Complex with TF (Tig)	61.7	52.2	40.3	2.0	1.7	1.3
P75711	YbbV		Monomer	167.1	141.2	109.1	21.9	18.5	14.3
P75712	YbbW	Cell inner membrane	Monomer	90.1	76.1	58.8	5.4	4.6	3.6
			Complex with SecB	64.8	52.9	42.3	2.3	1.9	1.5
			Complex with TF (Tig)	69.8	59.0	45.5	2.8	2.4	1.8
P77328	YbbY	Cell inner membrane	Monomer	93.9	79.3	61.3	6.0	5.1	3.9
			Complex with SecB	65.9	53.9	43.0	2.4	2.0	1.6
			Complex with TF (Tig)	71.3	60.2	46.5	3.0	2.5	2.0
P75717	YbcC		Monomer	169.2	142.9	110.4	22.5	19.0	14.7
P77528	YbcD		Monomer	183.9	155.4	120.0	26.6	22.5	17.4
P37325	YbcH		Monomer	108.0	91.2	70.5	8.4	7.1	5.5
P45570	YbcI	Cell inner membrane	Monomer	132.7	112.1	86.6	13.4	11.3	8.7
			Complex with SecB	73.3	59.9	47.9	3.2	2.6	2.1
			Complex with TF (Tig)	81.5	68.9	53.2	4.2	3.6	2.8
P0AAS7	YbcJ		Monomer	194.2	164.1	126.7	29.7	25.1	19.4
P77698	YbcK		Monomer	86.9	73.4	56.7	5.0	4.2	3.2
P77368	YbcL	Periplasm	Monomer	132.8	112.2	86.7	13.4	11.3	8.8
			Complex with SecB	73.4	59.9	47.9	3.2	2.6	2.1
			Complex with TF (Tig)	81.5	68.9	53.2	4.2	3.6	2.8
P77634	YbcM		Monomer	110.9	93.7	72.4	8.9	7.6	5.8
Q47269	YbcN		Monomer	138.7	117.2	90.5	14.8	12.5	9.6
P68661	YbcO		Monomer	170.2	143.8	111.1	22.8	19.2	14.8
P77598	YbcV		Monomer	142.1	120.0	92.7	15.5	13.1	10.1
P64435	YbcW		Monomer	191.9	162.1	125.2	29.0	24.5	18.9
P77460	YbcY		Monomer	128.7	108.7	84.0	12.5	10.6	8.2
P0A8Y8	YbdB		Monomer	147.3	124.4	96.1	16.8	14.2	11.0
P0AAS9	YbdD		Monomer	193.4	163.4	126.2	29.5	24.9	19.2
P0AAT2	YbdF		Monomer	151.0	127.6	98.5	17.7	15.0	11.6

UniProtKB A. N.	Protein name	Subcellular location	Subunit	D_0 $\left(\frac{\mu m^2}{s}\right)$			D_{cyto} $\left(\frac{\mu m^2}{s}\right)$		
				310 K	303 K	293 K	310 K	303 K	293 K
P45579	YbdH		Monomer	101.1	85.4	66.0	7.2	6.1	4.7
P77506	YbdJ	Cell membrane	Monomer	176.7	149.3	115.3	24.6	20.8	16.0
			Complex with SecB	76.9	62.8	50.2	3.7	3.0	2.4
			Complex with TF (Tig)	86.8	73.4	56.7	5.0	4.2	3.2
P77213	YbdK		Monomer	98.6	83.3	64.3	6.8	5.7	4.4
			Homodimer	75.1	61.4	49.0	3.4	2.8	2.2
P77806	YbdL	Cytoplasm	Monomer	97.4	82.3	63.6	6.6	5.6	4.3
			Homodimer	74.2	60.7	48.4	3.3	2.7	2.2
P77174	YbdM		Monomer	122.5	103.5	79.9	11.2	9.5	7.3
P77216	YbdN		Monomer	93.4	78.9	61.0	5.9	5.0	3.9
P77746	YbdO		Monomer	106.3	89.8	69.4	8.1	6.8	5.3
P77316	YbdR		Monomer	96.4	81.4	62.9	6.4	5.4	4.2
P18393	YbdZ		Monomer	185.8	157.0	121.3	27.2	23.0	17.7
P0AAT6	YbeB	Cytoplasm	Monomer	162.9	137.6	106.3	20.8	17.5	13.6
P0A8J4	YbeD		Monomer	173.7	146.7	113.3	23.7	20.0	15.5
P30979	YbeF		Monomer	104.5	88.2	68.2	7.8	6.6	5.1
P0AAT9	YbeL		Monomer	134.7	113.8	87.9	13.9	11.7	9.0
P39874	YbeM		Monomer	114.2	96.5	74.5	9.6	8.1	6.2
P77234	YbeQ		Monomer	103.3	87.2	67.4	7.6	6.4	4.9
P77627	YbeR		Monomer	115.7	97.7	75.5	9.8	8.3	6.4
P77296	YbeT		Monomer	129.2	109.1	84.3	12.6	10.7	8.2
P77427	YbeU		Monomer	116.8	98.7	76.2	10.1	8.5	6.6
P0A898	YbeY	Cytoplasm	Monomer	138.5	117.0	90.3	14.7	12.4	9.6
P0A9K3	YbeZ	Cytoplasm	Monomer	101.1	85.5	66.0	7.2	6.1	4.7
P0AAU2	YbfA		Monomer	185.8	157.0	121.3	27.2	23.0	17.7
P0AAU5	YbfB	Cell membrane	Monomer	157.8	133.3	103.0	19.4	16.4	12.7
			Complex with SecB	75.7	61.9	49.4	3.5	2.9	2.3
			Complex with TF (Tig)	85.1	71.9	55.5	4.7	4.0	3.1
P28915	YbfC		Monomer	126.7	107.1	82.7	12.1	10.2	7.9
P28916	YbfD		Monomer	113.9	96.2	74.3	9.5	8.0	6.2
P0AAU7	YbfE		Monomer	164.6	139.0	107.4	21.2	17.9	13.8

UniProtKB A. N.	Protein name	Subcellular location	Subunit	D_0 $\left(\frac{\mu m^2}{s}\right)$			D_{cyto} $\left(\frac{\mu m^2}{s}\right)$		
				310 K	303 K	293 K	310 K	303 K	293 K
P75736	YbfF		Monomer	114.5	96.8	74.7	9.6	8.1	6.3
P37003	YbfG		Monomer	121.2	102.4	79.1	11.0	9.3	7.1
P39901	YbfI		Monomer	190.0	160.5	124.0	28.4	24.0	18.5
P46121	YbfK		Monomer	176.0	148.7	114.9	24.4	20.6	15.9
P75741	YbfL		Monomer	97.2	82.2	63.5	6.6	5.5	4.3
P75733	YbfM		Monomer	89.9	75.9	58.6	5.4	4.6	3.5
P75734	YbfN	Cell membrane	Monomer	160.6	135.7	104.8	20.2	17.0	13.2
			Complex with SecB	75.9	62.0	49.6	3.5	2.9	2.3
			Complex with TF (Tig)	85.4	72.1	55.7	4.8	4.0	3.1
P77779	YbfO		Monomer	88.3	74.6	57.6	5.2	4.4	3.4
P75737	YbfP	Cell membrane	Monomer	136.2	115.0	88.9	14.2	12.0	9.3
			Complex with SecB	73.8	60.3	48.1	3.3	2.7	2.1
			Complex with TF (Tig)	82.1	69.4	53.6	4.3	3.6	2.8
Q2EEQ8	YbfQ		Monomer	175.6	148.4	114.6	24.3	20.5	15.8
P24252	YbgA		Monomer	130.9	110.6	85.4	13.0	11.0	8.5
P0A8Z3	YbgC	Cell inner membrane	Monomer	145.1	122.5	94.7	16.3	13.7	10.6
P37909	YbgD	Fimbrium	Monomer	131.9	111.4	86.1	13.2	11.2	8.6
			Complex with SecB	73.2	59.8	47.8	3.2	2.6	2.1
			Complex with TF (Tig)	81.4	68.7	53.1	4.2	3.6	2.8
P0AAV0	YbgE		Monomer	166.6	140.7	108.7	21.8	18.4	14.2
P45955	YbgF		Monomer	114.9	97.0	74.9	9.7	8.2	6.3
P0AFP6	YbgI		Monomer	117.1	98.9	76.4	10.1	8.6	6.6
			Homohexamer	58.0	47.4	37.8	1.7	1.4	1.1
P0AAV4	YbgJ		Monomer	122.5	103.5	79.9	11.2	9.5	7.3
P75745	YbgK		Monomer	106.3	89.8	69.4	8.1	6.8	5.3
P75746	YbgL		Monomer	119.0	100.5	77.6	10.5	8.9	6.9
P75748	YbgO		Monomer	100.6	85.0	65.7	7.1	6.0	4.6
P75749	YbgP	Periplasm	Monomer	117.0	98.8	76.3	10.1	8.5	6.6
			Complex with SecB	71.1	58.1	46.4	3.0	2.4	1.9
			Complex with TF (Tig)	78.2	66.1	51.1	3.8	3.2	2.5
P75750	YbgQ	Cell outer membrane	Monomer	72.9	61.6	47.5	3.2	2.7	2.1

UniProtKB A. N.	Protein name	Subcellular location	Subunit	D_0 $\left(\frac{\mu m^2}{s}\right)$ 310 K	D_0 303 K	D_0 293 K	D_{cyto} $\left(\frac{\mu m^2}{s}\right)$ 310 K	D_{cyto} 303 K	D_{cyto} 293 K
			Complex with SecB	58.3	47.6	38.0	1.7	1.4	1.1
			Complex with TF (Tig)	61.6	52.0	40.2	2.0	1.7	1.3
P0AAV6	YbgS		Monomer	156.3	132.0	102.0	19.0	16.1	12.4
			Monomer	246.1	207.9	160.6	46.8	39.6	30.6
P56100	YbgT	Membrane	Complex with SecB	79.1	64.6	51.6	3.9	3.2	2.6
			Complex with TF (Tig)	90.2	76.2	58.9	5.5	4.6	3.6
P21829	YbhA		Monomer	111.9	94.5	73.0	9.1	7.7	5.9
P12994	YbhB	Cytoplasm	Monomer	139.8	118.1	91.3	15.0	12.7	9.8
			Homodimer	106.6	87.1	69.5	8.1	6.7	5.3
			Monomer	94.8	80.1	61.9	6.2	5.2	4.0
P46130	YbhC	Cell outer membrane	Complex with SecB	66.2	54.1	43.2	2.5	2.0	1.6
			Complex with TF (Tig)	71.6	60.5	46.7	3.0	2.6	2.0
P52696	YbhD		Monomer	104.3	88.2	68.1	7.8	6.6	5.1
P0A9U1	YbhF		Monomer	83.8	70.8	54.7	4.5	3.8	3.0
			Monomer	103.9	87.8	67.8	7.7	6.5	5.0
P75777	YbhG	Periplasm	Complex with SecB	68.5	56.0	44.7	2.7	2.2	1.8
			Complex with TF (Tig)	74.7	63.1	48.7	3.4	2.9	2.2
P0AAV8	YbhH		Monomer	103.2	87.2	67.4	7.6	6.4	4.9
			Monomer	90.8	76.7	59.3	5.6	4.7	3.6
P75763	YbhI	Cell inner membrane	Complex with SecB	65.0	53.1	42.4	2.3	1.9	1.5
			Complex with TF (Tig)	70.1	59.2	45.7	2.9	2.4	1.9
P75764	YbhJ		Monomer	75.8	64.0	49.5	3.5	3.0	2.3
P75767	YbhK		Monomer	108.3	91.5	70.7	8.5	7.1	5.5
			Monomer	118.8	100.4	77.5	10.5	8.8	6.8
P0AAC4	YbhL	Cell inner membrane	Complex with SecB	71.4	58.3	46.6	3.0	2.5	2.0
			Complex with TF (Tig)	78.7	66.5	51.3	3.9	3.3	2.5
			Monomer	118.4	100.0	77.3	10.4	8.8	6.8
P75769	YbhM	Cell membrane	Complex with SecB	71.3	58.3	46.5	3.0	2.4	2.0
			Complex with TF (Tig)	78.6	66.4	51.3	3.9	3.3	2.5
			Monomer	104.6	88.3	68.2	7.8	6.6	5.1
P75770	YbhN	Cell inner membrane	Complex with SecB	68.6	56.1	44.8	2.7	2.2	1.8

UniProtKB A. N.	Protein name	Subcellular location	Subunit	D_0 $\left(\frac{\mu m^2}{s}\right)$			D_{cyto} $\left(\frac{\mu m^2}{s}\right)$		
				310 K	303 K	293 K	310 K	303 K	293 K
			Complex with TF (Tig)	74.9	63.3	48.9	3.4	2.9	2.2
P0AA84	YbhO	Cell membrane	Monomer	93.6	79.0	61.1	6.0	5.0	3.9
			Complex with SecB	65.8	53.8	43.0	2.4	2.0	1.6
			Complex with TF (Tig)	71.1	60.1	46.4	3.0	2.5	1.9
P0AAW1	YbhP		Monomer	114.0	96.3	74.4	9.5	8.0	6.2
P0AAW5	YbhQ	Cell inner membrane	Monomer	145.4	122.8	94.9	16.3	13.8	10.7
			Complex with SecB	74.7	61.0	48.7	3.4	2.8	2.2
			Complex with TF (Tig)	83.5	70.5	54.5	4.5	3.8	2.9
P0AFP9	YbhR	Cell inner membrane	Monomer	98.7	83.4	64.4	6.8	5.7	4.4
			Complex with SecB	67.2	54.9	43.9	2.6	2.1	1.7
			Complex with TF (Tig)	73.0	61.7	47.6	3.2	2.7	2.1
P0AFQ2	YbhS	Cell inner membrane	Monomer	98.2	83.0	64.1	6.7	5.7	4.4
			Complex with SecB	67.1	54.8	43.8	2.5	2.1	1.7
			Complex with TF (Tig)	72.8	61.5	47.5	3.2	2.7	2.1
P0AAW9	YbhT	Membrane	Monomer	221.3	186.9	144.4	38.3	32.4	25.0
			Complex with SecB	78.6	64.2	51.3	3.9	3.1	2.5
			Complex with TF (Tig)	89.4	75.5	58.3	5.3	4.5	3.5
P30176	YbiA		Monomer	135.1	114.1	88.1	13.9	11.8	9.1
P30177	YbiB		Monomer	105.5	89.1	68.9	8.0	6.7	5.2
P30178	YbiC	Cytoplasm	Monomer	101.3	85.6	66.1	7.2	6.1	4.7
P0ACU0	YbiH		Monomer	120.4	101.7	78.6	10.8	9.1	7.0
P41039	YbiI		Monomer	174.2	147.2	113.7	23.9	20.2	15.6
P0AAX3	YbiJ	Periplasm	Monomer	183.3	154.8	119.6	26.4	22.3	17.3
			Complex with SecB	77.2	63.1	50.4	3.7	3.0	2.4
			Complex with TF (Tig)	87.3	73.8	57.0	5.0	4.3	3.3
P75783	YbiO	Cell membrane	Monomer	75.7	63.9	49.4	3.5	3.0	2.3
			Complex with SecB	59.5	48.6	38.8	1.8	1.5	1.2
			Complex with TF (Tig)	63.1	53.3	41.2	2.2	1.8	1.4
P75785	YbiP	Cell inner membrane	Monomer	85.6	72.3	55.9	4.8	4.1	3.1
			Complex with SecB	63.3	51.8	41.3	2.2	1.8	1.4
			Complex with TF (Tig)	67.9	57.4	44.3	2.6	2.2	1.7

UniProtKB A. N.	Protein name	Subcellular location	Subunit	$D_0 \left(\frac{\mu m^2}{s}\right)$			$D_{\text{cyto}} \left(\frac{\mu m^2}{s}\right)$		
				310 K	303 K	293 K	310 K	303 K	293 K
P75788	YbiR	Cell inner membrane	Monomer	99.1	83.7	64.6	6.9	5.8	4.5
			Complex with SecB	67.3	55.0	43.9	2.6	2.1	1.7
			Complex with TF (Tig)	73.1	61.8	47.7	3.2	2.7	2.1
P0AAX8	YbiS	Periplasm	Monomer	107.6	90.9	70.2	8.3	7.0	5.4
			Complex with SecB	69.3	56.6	45.2	2.8	2.3	1.8
			Complex with TF (Tig)	75.8	64.0	49.5	3.5	3.0	2.3
P0A9U3	YbiT		Monomer	85.5	72.3	55.8	4.8	4.0	3.1
P75791	YbiU		Monomer	93.8	79.2	61.2	6.0	5.1	3.9
P75793	YbiW	Cytoplasm	Monomer	72.9	61.6	47.5	3.2	2.7	2.1
P75779	YbiX		Monomer	119.5	100.9	78.0	10.6	9.0	6.9
P75794	YbiY	Cytoplasm	Monomer	108.0	91.2	70.5	8.4	7.1	5.5
P46119	YbjC		Monomer	169.1	142.9	110.4	22.4	19.0	14.6
P75828	YbjD		Monomer	83.5	70.6	54.5	4.5	3.8	2.9
P75826	YbjE	Cell membrane	Monomer	108.9	92.0	71.1	8.6	7.2	5.6
			Complex with SecB	69.6	56.8	45.4	2.8	2.3	1.8
			Complex with TF (Tig)	76.2	64.3	49.7	3.6	3.0	2.3
P75806	YbjG	Cell inner membrane	Monomer	125.8	106.2	82.1	11.9	10.1	7.8
			Complex with SecB	72.4	59.2	47.3	3.1	2.6	2.0
			Complex with TF (Tig)	80.2	67.7	52.3	4.1	3.4	2.7
P0AAY4	YbjH		Monomer	172.7	145.9	112.7	23.4	19.8	15.3
P75809	YbjI		Monomer	111.9	94.5	73.0	9.1	7.7	6.0
P75810	YbjJ	Cell inner membrane	Monomer	98.4	83.2	64.2	6.7	5.7	4.4
			Complex with SecB	67.2	54.9	43.8	2.6	2.1	1.7
			Complex with TF (Tig)	72.9	61.6	47.6	3.2	2.7	2.1
P75811	YbjK		Monomer	130.7	110.4	85.3	13.0	10.9	8.5
P60869	YbjL	Cell membrane	Monomer	85.3	72.0	55.6	4.7	4.0	3.1
			Complex with SecB	63.2	51.6	41.3	2.2	1.8	1.4
			Complex with TF (Tig)	67.7	57.2	44.2	2.6	2.2	1.7
P64439	YbjM	Cell inner membrane	Monomer	150.3	127.0	98.1	17.6	14.8	11.5
			Complex with SecB	75.1	61.4	49.0	3.4	2.8	2.2
			Complex with TF (Tig)	84.2	71.1	54.9	4.6	3.9	3.0

UniProtKB A. N.	Protein name	Subcellular location	Subunit	D_0 $\left(\frac{\mu m^2}{s}\right)$			D_{cyto} $\left(\frac{\mu m^2}{s}\right)$		
				310 K	303 K	293 K	310 K	303 K	293 K
P0AAY6	YbjN		Monomer	138.0	116.6	90.1	14.6	12.3	9.5
P0AAZ0	YbjO	Cell inner membrane	Monomer	135.5	114.5	88.4	14.0	11.9	9.2
			Complex with SecB	73.7	60.2	48.1	3.3	2.7	2.1
			Complex with TF (Tig)	82.0	69.3	53.5	4.3	3.6	2.8
P75818	YbjP	Cell membrane	Monomer	134.2	113.3	87.6	13.7	11.6	9.0
			Complex with SecB	73.5	60.1	48.0	3.2	2.7	2.1
			Complex with TF (Tig)	81.8	69.1	53.4	4.3	3.6	2.8
P0A8C1	YbjQ		Monomer	163.7	138.3	106.8	21.0	17.7	13.7
P75821	YbjS		Monomer	102.1	86.3	66.6	7.4	6.2	4.8
P75822	YbjT		Monomer	89.3	75.4	58.2	5.3	4.5	3.5
P75829	YbjX		Monomer	101.9	86.0	66.5	7.3	6.2	4.8
P21367	YcaC		Monomer	124.3	105.0	81.1	11.6	9.8	7.6
			Homooctamer	55.0	44.9	35.9	1.5	1.2	1.0
P21503	YcaD	Cell inner membrane	Monomer	98.8	83.5	64.5	6.8	5.8	4.4
			Complex with SecB	67.3	54.9	43.9	2.6	2.1	1.7
			Complex with TF (Tig)	73.0	61.7	47.7	3.2	2.7	2.1
P37443	YcaI	Cell membrane	Monomer	74.7	63.1	48.8	3.4	2.9	2.2
			Complex with SecB	59.1	48.3	38.6	1.8	1.5	1.2
			Complex with TF (Tig)	62.6	52.9	40.9	2.1	1.8	1.4
P43340	YcaK		Monomer	124.4	105.1	81.2	11.6	9.8	7.6
P43674	YcaL		Monomer	117.3	99.1	76.6	10.2	8.6	6.6
P75835	YcaM	Cell inner membrane	Monomer	90.1	76.1	58.8	5.4	4.6	3.5
			Complex with SecB	64.8	52.9	42.3	2.3	1.9	1.5
			Complex with TF (Tig)	69.8	58.9	45.5	2.8	2.4	1.8
P75836	YcaN		Monomer	106.4	89.9	69.4	8.1	6.9	5.3
P75838	YcaO		Monomer	82.5	69.7	53.8	4.4	3.7	2.9
P75839	YcaP	Cell membrane	Monomer	118.1	99.8	77.1	10.3	8.7	6.7
			Complex with SecB	71.3	58.2	46.5	3.0	2.4	2.0
			Complex with TF (Tig)	78.5	66.3	51.2	3.9	3.3	2.5
P75843	YcaQ		Monomer	93.5	79.0	61.0	6.0	5.0	3.9
P0AAZ7	YcaR		Monomer	200.0	169.0	130.5	31.5	26.6	20.5

UniProtKB A. N.	Protein name	Subcellular location	Subunit	D_0 $\left(\frac{\mu m^2}{s}\right)$			D_{cyto} $\left(\frac{\mu m^2}{s}\right)$		
				310 K	303 K	293 K	310 K	303 K	293 K
P22525	YcbB	Membrane	Monomer	81.5	68.8	53.2	4.2	3.6	2.8
			Complex with SecB	61.8	50.5	40.4	2.0	1.7	1.3
			Complex with TF (Tig)	66.0	55.8	43.1	2.4	2.1	1.6
P0AB01	YcbC		Monomer	114.2	96.5	74.5	9.6	8.1	6.2
P40876	YcbF	Periplasm	Monomer	118.9	100.4	77.6	10.5	8.9	6.8
			Complex with SecB	71.4	58.3	46.6	3.0	2.5	2.0
			Complex with TF (Tig)	78.7	66.5	51.4	3.9	3.3	2.5
P0A8N0	YcbG		Monomer	137.9	116.5	90.0	14.6	12.3	9.5
P0AB03	YcbJ		Monomer	106.2	89.7	69.3	8.1	6.8	5.3
P0AB06	YcbK		Monomer	130.6	110.3	85.2	12.9	10.9	8.4
P75849	YcbL		Monomer	122.8	103.8	80.2	11.3	9.5	7.4
P75855	YcbQ	Fimbrium	Monomer	135.0	114.1	88.1	13.9	11.8	9.1
			Complex with SecB	73.6	60.1	48.0	3.3	2.7	2.1
			Complex with TF (Tig)	81.9	69.2	53.5	4.3	3.6	2.8
P75856	YcbR	Periplasm	Monomer	119.7	101.2	78.1	10.7	9.0	7.0
			Complex with SecB	71.5	58.4	46.7	3.0	2.5	2.0
			Complex with TF (Tig)	78.9	66.7	51.5	3.9	3.3	2.5
P75857	YcbS	Cell outer membrane	Monomer	71.3	60.2	46.5	3.0	2.5	2.0
			Complex with SecB	57.6	47.0	37.6	1.7	1.4	1.1
			Complex with TF (Tig)	60.7	51.3	39.6	1.9	1.6	1.3
P75858	YcbT	Fimbrium	Monomer	102.4	86.5	66.8	7.4	6.3	4.8
			Complex with SecB	68.1	55.7	44.5	2.7	2.2	1.7
			Complex with TF (Tig)	74.2	62.7	48.4	3.3	2.8	2.2
P75859	YcbU		Monomer	134.4	113.5	87.7	13.8	11.6	9.0
P75860	YcbV		Monomer	135.5	114.5	88.4	14.0	11.9	9.2
P75862	YcbW		Monomer	130.0	109.8	84.8	12.8	10.8	8.4
P75863	YcbX		Monomer	99.6	84.1	65.0	6.9	5.9	4.5
P75867	YcbZ		Monomer	82.4	69.6	53.8	4.4	3.7	2.8
P0AAC6	YccA	Cell inner membrane	Monomer	123.7	104.5	80.7	11.5	9.7	7.5
			Complex with SecB	72.1	58.9	47.1	3.1	2.5	2.0
			Complex with TF (Tig)	79.8	67.4	52.1	4.0	3.4	2.6

UniProtKB A. N.	Protein name	Subcellular location	Subunit	D_0 $\left(\frac{\mu m^2}{s}\right)$			D_{cyto} $\left(\frac{\mu m^2}{s}\right)$		
				310 K	303 K	293 K	310 K	303 K	293 K
P24244	YccB	Membrane	Monomer	257.6	217.6	168.1	51.0	43.1	33.3
			Complex with SecB	79.3	64.8	51.7	3.9	3.2	2.6
			Complex with TF (Tig)	90.5	76.5	59.1	5.5	4.7	3.6
P36661	YccE		Monomer	93.0	78.6	60.7	5.9	5.0	3.8
P0AB12	YccF	Cell inner membrane	Monomer	142.5	120.4	93.0	15.7	13.2	10.2
			Complex with SecB	74.4	60.8	48.6	3.4	2.7	2.2
			Complex with TF (Tig)	83.1	70.2	54.2	4.4	3.8	2.9
P0AB14	YccJ		Monomer	183.7	155.2	119.8	26.6	22.4	17.3
P52636	YccM	Cell inner membrane	Monomer	100.1	84.6	65.3	7.0	5.9	4.6
			Complex with SecB	67.6	55.2	44.1	2.6	2.1	1.7
			Complex with TF (Tig)	73.5	62.1	47.9	3.2	2.7	2.1
P75870	YccS	Cell inner membrane	Monomer	75.7	64.0	49.4	3.5	3.0	2.3
			Complex with SecB	59.6	48.7	38.9	1.8	1.5	1.2
			Complex with TF (Tig)	63.1	53.3	41.2	2.2	1.8	1.4
P0A8X4	YccT		Monomer	121.2	102.4	79.1	11.0	9.3	7.2
P75874	YccU		Monomer	148.3	125.3	96.8	17.1	14.4	11.1
P0AB65	YccX		Monomer	170.5	144.1	111.3	22.8	19.3	14.9
P31545	YcdB	Periplasm	Monomer	94.2	79.6	61.5	6.1	5.1	4.0
			Complex with SecB	66.0	53.9	43.1	2.4	2.0	1.6
			Complex with TF (Tig)	71.4	60.3	46.6	3.0	2.5	2.0
P0AB24	YcdO	Periplasm	Monomer	99.1	83.7	64.7	6.9	5.8	4.5
			Complex with SecB	67.3	55.0	43.9	2.6	2.1	1.7
			Complex with TF (Tig)	73.1	61.8	47.7	3.2	2.7	2.1
P75908	YcdT	Cell inner membrane	Monomer	90.5	76.5	59.1	5.5	4.7	3.6
			Complex with SecB	64.9	53.1	42.4	2.3	1.9	1.5
			Complex with TF (Tig)	70.0	59.1	45.7	2.8	2.4	1.9
P75910	YcdU	Cell membrane	Monomer	101.5	85.8	66.3	7.3	6.1	4.7
			Complex with SecB	67.9	55.5	44.3	2.6	2.2	1.7
			Complex with TF (Tig)	73.9	62.5	48.2	3.3	2.8	2.2
P75914	YcdX		Monomer	117.1	98.9	76.4	10.1	8.6	6.6
			Homotrimer	76.1	62.2	49.7	3.6	2.9	2.3

UniProtKB A. N.	Protein name	Subcellular location	Subunit	D_0 $\left(\frac{\mu m^2}{s}\right)$			D_{cyto} $\left(\frac{\mu m^2}{s}\right)$		
				310 K	303 K	293 K	310 K	303 K	293 K
P75915	YcdY		Monomer	129.7	109.5	84.6	12.7	10.8	8.3
P75916	YcdZ	Cell inner membrane	Monomer	139.9	118.2	91.3	15.0	12.7	9.8
			Complex with SecB	74.2	60.6	48.4	3.3	2.7	2.2
			Complex with TF (Tig)	82.7	69.9	54.0	4.4	3.7	2.9
P24188	YceA		Monomer	100.4	84.8	65.5	7.1	6.0	4.6
P0AB26	YceB	Cell membrane	Monomer	130.2	110.0	85.0	12.9	10.9	8.4
			Complex with SecB	73.0	59.7	47.7	3.2	2.6	2.1
			Complex with TF (Tig)	81.1	68.5	52.9	4.2	3.5	2.7
P0AB28	YceD		Monomer	133.3	112.6	87.0	13.5	11.4	8.8
P0A729	YceF	Cytoplasm	Monomer	127.4	107.6	83.1	12.2	10.3	8.0
P28306	YceG		Monomer	102.0	86.1	66.5	7.3	6.2	4.8
P29217	YceH		Monomer	122.1	103.1	79.6	11.1	9.4	7.3
P0A8X2	YceI	Periplasm	Monomer	129.2	109.1	84.3	12.6	10.7	8.2
			Complex with SecB	72.9	59.6	47.6	3.2	2.6	2.1
			Complex with TF (Tig)	80.9	68.3	52.8	4.2	3.5	2.7
P75925	YceJ	Cell inner membrane	Monomer	129.6	109.5	84.6	12.7	10.8	8.3
			Complex with SecB	73.0	59.6	47.6	3.2	2.6	2.1
			Complex with TF (Tig)	80.9	68.4	52.8	4.2	3.5	2.7
P0AB31	YceK		Monomer	184.4	155.7	120.3	26.8	22.6	17.5
P64442	YceO		Monomer	212.2	179.3	138.5	35.3	29.8	23.1
P62066	YceQ		Monomer	160.1	135.3	104.5	20.0	16.9	13.1
P27431	YcfD		Monomer	97.8	82.6	63.8	6.6	5.6	4.3
P0AFQ7	YcfH		Monomer	112.4	95.0	73.4	9.2	7.8	6.0
P0AB35	YcfJ	Membrane	Monomer	134.4	113.5	87.7	13.8	11.6	9.0
			Complex with SecB	73.5	60.1	48.0	3.3	2.7	2.1
			Complex with TF (Tig)	81.8	69.1	53.4	4.3	3.6	2.8
P45581	YcfK		Monomer	126.6	107.0	82.6	12.1	10.2	7.9
P75946	YcfL		Monomer	151.3	127.8	98.7	17.8	15.0	11.6
P0A8E1	YcfP		Monomer	128.4	108.5	83.8	12.5	10.5	8.1
P75952	YcfQ		Monomer	123.7	104.5	80.7	11.5	9.7	7.5
P75954	YcfS	Periplasm	Monomer	106.0	89.6	69.2	8.0	6.8	5.3

UniProtKB A. N.	Protein name	Subcellular location	Subunit	D_0 $\left(\frac{\mu m^2}{s}\right)$ 310 K	303 K	293 K	D_{cyto} $\left(\frac{\mu m^2}{s}\right)$ 310 K	303 K	293 K
			Complex with SecB	69.0	56.3	45.0	2.7	2.2	1.8
			Complex with TF (Tig)	75.3	63.6	49.1	3.5	2.9	2.3
P75955	YcfT	Cell inner membrane	Monomer	99.4	84.0	64.9	6.9	5.8	4.5
			Complex with SecB	67.4	55.1	44.0	2.6	2.1	1.7
			Complex with TF (Tig)	73.2	61.9	47.8	3.2	2.7	2.1
P75961	YcfZ	Cell inner membrane	Monomer	113.9	96.2	74.3	9.5	8.0	6.2
			Complex with SecB	70.5	57.6	46.0	2.9	2.4	1.9
			Complex with TF (Tig)	77.5	65.5	50.6	3.7	3.1	2.4
P29013	YcgB		Monomer	85.0	71.8	55.5	4.7	4.0	3.1
P75989	YcgE		Monomer	114.8	97.0	74.9	9.7	8.2	6.3
P75990	YcgF		Monomer	95.4	80.6	62.3	6.3	5.3	4.1
P75995	YcgG	Cytoplasm	Monomer	87.3	73.7	56.9	5.0	4.2	3.3
P76000	YcgI		Monomer	141.7	119.7	92.4	15.5	13.1	10.1
P76001	YcgJ		Monomer	153.8	129.9	100.3	18.4	15.6	12.0
P76002	YcgK		Monomer	147.5	124.6	96.3	16.9	14.2	11.0
P0AB43	YcgL		Monomer	164.8	139.2	107.5	21.3	18.0	13.9
P76004	YcgM		Monomer	123.0	103.9	80.3	11.3	9.6	7.4
P0A8L5	YcgN		Monomer	137.3	116.0	89.6	14.4	12.2	9.4
P76010	YcgR	Bacterial flagellum basal body	Monomer	115.5	97.5	75.3	9.8	8.3	6.4
			Complex with SecB	70.8	57.8	46.2	2.9	2.4	1.9
			Complex with TF (Tig)	77.9	65.8	50.8	3.8	3.2	2.5
P76017	YcgV		Monomer	70.0	59.2	45.7	2.9	2.4	1.9
P75988	YcgX		Monomer	144.3	121.9	94.2	16.1	13.6	10.5
P76012	YcgY		Monomer	141.1	119.2	92.1	15.3	12.9	10.0
P75991	YcgZ		Monomer	181.6	153.4	118.5	26.0	21.9	16.9
P25743	YchE	Cell membrane	Monomer	123.4	104.2	80.5	11.4	9.6	7.4
			Complex with SecB	72.1	58.9	47.0	3.1	2.5	2.0
			Complex with TF (Tig)	79.7	67.3	52.0	4.0	3.4	2.6
P30192	YchG		Monomer	107.0	90.4	69.8	8.2	7.0	5.4
P0AB49	YchH		Monomer	169.3	143.1	110.5	22.5	19.0	14.7
P37052	YchJ		Monomer	140.2	118.4	91.5	15.1	12.8	9.9

UniProtKB A. N.	Protein name	Subcellular location	Subunit	D_0 $\left(\frac{\mu m^2}{s}\right)$			D_{cyto} $\left(\frac{\mu m^2}{s}\right)$		
				310 K	303 K	293 K	310 K	303 K	293 K
P0AFR2	YchM	Cell inner membrane	Monomer	85.8	72.5	56.0	4.8	4.1	3.1
			Complex with SecB	63.4	51.8	41.4	2.2	1.8	1.4
			Complex with TF (Tig)	68.0	57.4	44.4	2.6	2.2	1.7
P0AB52	YchN		Monomer	157.1	132.7	102.5	19.3	16.3	12.6
			Homohexamer	77.8	63.6	50.8	3.8	3.1	2.5
P39165	YchO	Periplasm	Monomer	90.3	76.3	58.9	5.5	4.6	3.6
			Complex with SecB	64.9	53.0	42.3	2.3	1.9	1.5
			Complex with TF (Tig)	69.9	59.0	45.6	2.8	2.4	1.9
P76023	YchS		Monomer	175.3	148.1	114.4	24.1	20.4	15.8
P0A8Z0	YciA		Monomer	150.2	126.9	98.0	17.5	14.8	11.4
P21365	YciC	Cell inner membrane	Monomer	117.9	99.6	76.9	10.3	8.7	6.7
			Complex with SecB	71.2	58.2	46.5	3.0	2.4	1.9
			Complex with TF (Tig)	78.5	66.3	51.2	3.8	3.2	2.5
P21363	YciE		Monomer	134.2	113.4	87.6	13.7	11.6	9.0
P21362	YciF		Monomer	135.3	114.3	88.3	14.0	11.8	9.1
			Homodimer	103.1	84.2	67.3	7.5	6.2	4.9
P21361	YciG		Monomer	210.7	178.0	137.5	34.9	29.4	22.7
P08245	YciH		Monomer	163.9	138.5	107.0	21.0	17.8	13.7
P0AB55	YciI		Monomer	168.6	142.4	110.0	22.3	18.8	14.6
			Homodimer	128.5	105.0	83.8	12.5	10.2	8.1
P31808	YciK		Monomer	115.3	97.4	75.3	9.8	8.3	6.4
P0AB58	YciM		Monomer	96.1	81.2	62.7	6.4	5.4	4.2
P0AB61	YciN		Monomer	176.9	149.4	115.4	24.6	20.8	16.1
P0AFR4	YciO		Monomer	124.0	104.8	80.9	11.5	9.7	7.5
P45848	YciQ		Monomer	79.9	67.5	52.1	4.0	3.4	2.6
P0ACV4	YciS	Cell inner membrane	Monomer	164.2	138.7	107.1	21.1	17.8	13.8
			Complex with SecB	76.2	62.2	49.7	3.6	2.9	2.3
			Complex with TF (Tig)	85.7	72.4	55.9	4.8	4.1	3.1
P76034	YciT		Monomer	115.9	97.9	75.6	9.9	8.4	6.5
P0A8L7	YciU		Monomer	157.2	132.8	102.5	19.3	16.3	12.6
P76035	YciW		Monomer	98.1	82.9	64.0	6.7	5.7	4.4

UniProtKB A. N.	Protein name	Subcellular location	Subunit	D_0 $\left(\frac{\mu m^2}{s}\right)$			D_{cyto} $\left(\frac{\mu m^2}{s}\right)$		
				310 K	303 K	293 K	310 K	303 K	293 K
P58094	YciX		Monomer	206.3	174.3	134.6	33.4	28.2	21.8
A5A613	YciY		Monomer	197.3	166.7	128.7	30.6	25.9	20.0
A5A614	YciZ		Monomer	205.0	173.2	133.8	33.0	27.9	21.5
P45736	YcjD		Monomer	151.2	127.8	98.7	17.8	15.0	11.6
P0A8R7	YcjF	Cell inner membrane	Monomer	100.8	85.2	65.8	7.1	6.0	4.7
			Complex with SecB	67.8	55.4	44.2	2.6	2.1	1.7
			Complex with TF (Tig)	73.7	62.3	48.1	3.3	2.8	2.1
P51981	YcjG		Monomer	106.0	89.5	69.1	8.0	6.8	5.2
P76041	YcjM		Monomer	83.2	70.3	54.3	4.5	3.8	2.9
P76042	YcjN	Periplasm	Monomer	94.2	79.6	61.5	6.1	5.1	4.0
			Complex with SecB	66.0	53.9	43.1	2.4	2.0	1.6
			Complex with TF (Tig)	71.4	60.3	46.6	3.0	2.5	2.0
P0AFR7	YcjO	Cell inner membrane	Monomer	107.8	91.0	70.3	8.4	7.1	5.5
			Complex with SecB	69.3	56.6	45.2	2.8	2.3	1.8
			Complex with TF (Tig)	75.8	64.1	49.5	3.5	3.0	2.3
P77716	YcjP	Cell inner membrane	Monomer	110.4	93.2	72.0	8.8	7.5	5.8
			Complex with SecB	69.9	57.1	45.6	2.8	2.3	1.9
			Complex with TF (Tig)	76.6	64.7	50.0	3.6	3.0	2.4
P76043	YcjQ		Monomer	102.0	86.2	66.6	7.3	6.2	4.8
P76044	YcjR		Monomer	112.4	95.0	73.3	9.2	7.8	6.0
P77503	YcjS		Monomer	101.5	85.8	66.3	7.3	6.1	4.7
P77154	YcjT		Monomer	74.6	63.0	48.7	3.4	2.9	2.2
P77366	YcjU		Monomer	123.3	104.2	80.4	11.4	9.6	7.4
P77481	YcjV		Monomer	100.0	84.5	65.3	7.0	5.9	4.6
P77615	YcjW		Monomer	104.3	88.1	68.0	7.7	6.5	5.0
P76046	YcjX		Monomer	90.0	76.0	58.7	5.4	4.6	3.5
P76049	YcjY		Monomer	107.2	90.6	70.0	8.3	7.0	5.4
P77333	YcjZ		Monomer	107.4	90.8	70.1	8.3	7.0	5.4
P33230	YdaC		Monomer	189.6	160.1	123.7	28.3	23.9	18.5
P38394	YdaE		Monomer	203.2	171.7	132.6	32.5	27.4	21.2
P0ACW0	YdaF		Monomer	214.0	180.8	139.7	35.9	30.3	23.4

UniProtKB A. N.	Protein name	Subcellular location	Subunit	$D_0 \left(\frac{\mu m^2}{s}\right)$			$D_{\text{cyto}} \left(\frac{\mu m^2}{s}\right)$		
				310 K	303 K	293 K	310 K	303 K	293 K
P76061	YdaG		Monomer	223.0	188.4	145.5	38.9	32.8	25.4
P76053	YdaL		Monomer	127.8	107.9	83.4	12.3	10.4	8.0
P77302	YdaM		Monomer	94.5	79.8	61.7	6.1	5.2	4.0
			Homodimer	72.0	58.8	47.0	3.1	2.5	2.0
P76057	YdaQ		Monomer	184.2	155.6	120.2	26.7	22.6	17.4
P76063	YdaS		Monomer	166.3	140.5	108.5	21.7	18.3	14.2
P76064	YdaT		Monomer	144.4	122.0	94.2	16.1	13.6	10.5
P76065	YdaU		Monomer	108.7	91.8	70.9	8.5	7.2	5.6
P77546	YdaV		Monomer	115.0	97.2	75.1	9.7	8.2	6.3
P76066	YdaW		Monomer	126.4	106.8	82.5	12.0	10.2	7.8
P76069	YdaY		Monomer	153.5	129.7	100.2	18.3	15.5	12.0
P33666	YdbA		Monomer	52.7	44.5	34.4	1.3	1.1	0.8
P25906	YdbC		Monomer	111.1	93.9	72.5	9.0	7.6	5.9
P25907	YdbD		Monomer	74.0	62.5	48.3	3.3	2.8	2.2
P52645	YdbH		Monomer	70.8	59.9	46.2	2.9	2.5	1.9
P0ACW2	YdbJ		Monomer	182.2	154.0	118.9	26.1	22.1	17.1
P52647	YdbK		Monomer	63.3	53.5	41.3	2.2	1.8	1.4
P76076	YdbL		Monomer	161.3	136.2	105.2	20.3	17.2	13.3
P0ACW4	YdcA		Monomer	212.7	179.7	138.8	35.5	30.0	23.2
P28917	YdcC		Monomer	97.3	82.2	63.5	6.6	5.5	4.3
P31991	YdcD		Monomer	134.4	113.5	87.7	13.8	11.6	9.0
P34209	YdcF		Monomer	112.6	95.1	73.5	9.3	7.8	6.0
P0ACW6	YdcH		Monomer	180.8	152.7	118.0	25.7	21.7	16.8
P77171	YdcI		Monomer	107.5	90.8	70.2	8.3	7.0	5.4
P76097	YdcJ		Monomer	91.1	76.9	59.4	5.6	4.7	3.6
P76100	YdcK		Monomer	104.6	88.4	68.2	7.8	6.6	5.1
P64451	YdcL	Cell membrane	Monomer	121.6	102.7	79.3	11.0	9.3	7.2
			Complex with SecB	71.8	58.7	46.9	3.1	2.5	2.0
			Complex with TF (Tig)	79.3	67.0	51.8	4.0	3.3	2.6
P77626	YdcN		Monomer	132.2	111.7	86.2	13.3	11.2	8.7
P76103	YdcO	Cell inner membrane	Monomer	99.6	84.2	65.0	6.9	5.9	4.5

UniProtKB A. N.	Protein name	Subcellular location	Subunit	D_0 $\left(\frac{\mu m^2}{s}\right)$			D_{cyto} $\left(\frac{\mu m^2}{s}\right)$		
				310 K	303 K	293 K	310 K	303 K	293 K
			Complex with SecB	67.5	55.1	44.0	2.6	2.1	1.7
			Complex with TF (Tig)	73.3	61.9	47.8	3.2	2.7	2.1
P76104	YdcP		Monomer	79.3	67.0	51.7	3.9	3.3	2.6
P77730	YdcR		Monomer	89.9	75.9	58.6	5.4	4.6	3.5
P76108	YdcS	Periplasm	Monomer	98.0	82.8	64.0	6.7	5.6	4.4
			Complex with SecB	67.1	54.8	43.8	2.5	2.1	1.7
			Complex with TF (Tig)	72.8	61.5	47.5	3.2	2.7	2.1
P77795	YdcT		Monomer	103.3	87.2	67.4	7.6	6.4	4.9
P77156	YdcU	Cell inner membrane	Monomer	106.3	89.8	69.4	8.1	6.8	5.3
			Complex with SecB	69.0	56.4	45.0	2.7	2.2	1.8
			Complex with TF (Tig)	75.4	63.7	49.2	3.5	2.9	2.3
P0AFR9	YdcV	Cell inner membrane	Monomer	114.1	96.4	74.4	9.5	8.1	6.2
			Complex with SecB	70.6	57.6	46.0	2.9	2.4	1.9
			Complex with TF (Tig)	77.5	65.5	50.6	3.7	3.2	2.4
P64453	YdcX	Cell membrane	Monomer	202.5	171.1	132.1	32.2	27.2	21.0
			Complex with SecB	78.0	63.7	50.9	3.8	3.1	2.5
			Complex with TF (Tig)	88.5	74.8	57.8	5.2	4.4	3.4
P64455	YdcY		Monomer	181.4	153.2	118.4	25.9	21.9	16.9
P76111	YdcZ	Cell inner membrane	Monomer	144.0	121.7	94.0	16.0	13.5	10.4
			Complex with SecB	74.6	60.9	48.7	3.4	2.8	2.2
			Complex with TF (Tig)	83.3	70.4	54.4	4.5	3.8	2.9
P31826	YddA	Cell inner membrane	Monomer	82.8	70.0	54.1	4.4	3.7	2.9
			Complex with SecB	62.4	50.9	40.7	2.1	1.7	1.4
			Complex with TF (Tig)	66.6	56.3	43.5	2.5	2.1	1.6
P31827	YddB		Monomer	73.1	61.8	47.7	3.2	2.7	2.1
P37757	YddE		Monomer	108.9	92.0	71.1	8.6	7.2	5.6
			Homodimer	83.0	67.8	54.2	4.4	3.6	2.9
			Homotetramer	63.3	53.4	41.3	2.2	1.8	1.4
P46136	YddG	Cell inner membrane	Monomer	110.0	92.9	71.8	8.8	7.4	5.7
			Complex with SecB	69.8	57.0	45.5	2.8	2.3	1.8
			Complex with TF (Tig)	76.5	64.6	49.9	3.6	3.0	2.3

UniProtKB A. N.	Protein name	Subcellular location	Subunit	D_0 $\left(\frac{\mu m^2}{s}\right)$			D_{cyto} $\left(\frac{\mu m^2}{s}\right)$		
				310 K	303 K	293 K	310 K	303 K	293 K
P76121	YddH		Monomer	129.1	109.0	84.2	12.6	10.6	8.2
P76122	YddJ		Monomer	156.3	132.0	102.0	19.0	16.1	12.4
P76123	YddK		Monomer	104.1	88.0	68.0	7.7	6.5	5.0
P77519	YddL		Monomer	167.8	141.7	109.5	22.1	18.7	14.4
P67699	YddM		Monomer	169.9	143.5	110.9	22.7	19.1	14.8
P64426	YddW	Cell membrane	Monomer	92.1	77.8	60.1	5.7	4.9	3.7
			Complex with SecB	65.4	53.4	42.7	2.4	1.9	1.6
			Complex with TF (Tig)	70.6	59.6	46.1	2.9	2.5	1.9
P31126	YdeE	Cell membrane	Monomer	97.7	82.5	63.8	6.6	5.6	4.3
			Complex with SecB	67.0	54.7	43.7	2.5	2.1	1.7
			Complex with TF (Tig)	72.6	61.4	47.4	3.1	2.7	2.1
P31129	YdeH		Monomer	106.9	90.4	69.8	8.2	6.9	5.4
			Homodimer	81.5	66.6	53.2	4.2	3.5	2.8
P31130	YdeI		Monomer	150.7	127.3	98.3	17.6	14.9	11.5
P31131	YdeJ		Monomer	136.1	115.0	88.8	14.2	12.0	9.2
P32051	YdeK	Cell membrane	Monomer	61.9	52.3	40.4	2.0	1.7	1.3
			Complex with SecB	52.7	43.1	34.4	1.3	1.1	0.8
			Complex with TF (Tig)	55.0	46.5	35.9	1.5	1.2	1.0
P76134	YdeM		Monomer	96.1	81.2	62.7	6.4	5.4	4.2
P77318	YdeN		Monomer	84.0	70.9	54.8	4.6	3.9	3.0
P76135	YdeO		Monomer	114.1	96.4	74.4	9.5	8.1	6.2
P77561	YdeP		Monomer	75.1	63.4	49.0	3.4	2.9	2.2
P77588	YdeQ	Fimbrium	Monomer	109.3	92.3	71.3	8.6	7.3	5.6
			Complex with SecB	69.6	56.9	45.4	2.8	2.3	1.8
			Complex with TF (Tig)	76.3	64.4	49.8	3.6	3.0	2.3
P77294	YdeR	Fimbrium	Monomer	136.6	115.4	89.1	14.3	12.1	9.3
			Complex with SecB	73.8	60.3	48.2	3.3	2.7	2.1
			Complex with TF (Tig)	82.2	69.4	53.6	4.3	3.7	2.8
P77789	YdeS	Fimbrium	Monomer	134.3	113.4	87.6	13.8	11.6	9.0
			Complex with SecB	73.5	60.1	48.0	3.2	2.7	2.1
			Complex with TF (Tig)	81.8	69.1	53.4	4.3	3.6	2.8

UniProtKB A. N.	Protein name	Subcellular location	Subunit	D_0 $\left(\frac{\mu m^2}{s}\right)$			D_{cyto} $\left(\frac{\mu m^2}{s}\right)$		
				310 K	303 K	293 K	310 K	303 K	293 K
P76137	YdeT		Monomer	98.6	83.3	64.4	6.8	5.7	4.4
P77286	YdeU		Monomer	91.4	77.2	59.7	5.6	4.8	3.7
P0ACW8	YdfA		Monomer	213.7	180.5	139.5	35.8	30.3	23.4
P29009	YdfB		Monomer	232.2	196.1	151.5	42.0	35.5	27.4
P21418	YdfC		Monomer	185.2	156.4	120.8	27.0	22.8	17.6
P29010	YdfD		Monomer	200.2	169.1	130.6	31.5	26.6	20.6
Q47138	YdfE		Monomer	113.5	95.9	74.1	9.4	8.0	6.2
P39831	YdfG		Monomer	116.5	98.4	76.0	10.0	8.5	6.5
			Homotetramer	67.6	55.3	44.1	2.6	2.1	1.7
P0ACM2	YdfH		Monomer	117.6	99.4	76.8	10.2	8.6	6.7
P77260	YdfI		Monomer	89.3	75.4	58.3	5.3	4.5	3.5
P77228	YdfJ	Cell inner membrane	Monomer	94.6	79.9	61.8	6.1	5.2	4.0
			Complex with SecB	66.1	54.0	43.2	2.5	2.0	1.6
			Complex with TF (Tig)	71.5	60.4	46.7	3.0	2.6	2.0
P76154	YdfK		Monomer	171.6	145.0	112.0	23.1	19.5	15.1
P76156	YdfO		Monomer	141.8	119.8	92.5	15.5	13.1	10.1
P76160	YdfR		Monomer	160.1	135.3	104.5	20.0	16.9	13.1
P76162	YdfU		Monomer	100.9	85.3	65.9	7.2	6.1	4.7
P76163	YdfV		Monomer	164.4	138.9	107.3	21.2	17.9	13.8
P76164	YdfW		Monomer	182.0	153.7	118.7	26.1	22.0	17.0
P76165	YdfX		Monomer	166.3	140.5	108.5	21.7	18.3	14.2
P64463	YdfZ		Monomer	195.4	165.1	127.5	30.1	25.4	19.6
P77804	YdgA	Cell inner membrane	Monomer	88.6	74.9	57.8	5.2	4.4	3.4
			Complex with SecB	64.3	52.6	42.0	2.3	1.9	1.5
			Complex with TF (Tig)	69.2	58.4	45.1	2.8	2.3	1.8
P0ACX0	YdgC	Cell inner membrane	Monomer	159.0	134.3	103.7	19.7	16.7	12.9
			Complex with SecB	75.8	61.9	49.5	3.5	2.9	2.3
			Complex with TF (Tig)	85.2	72.0	55.6	4.7	4.0	3.1
P76176	YdgD		Monomer	113.2	95.7	73.9	9.4	7.9	6.1
P76177	YdgH		Monomer	106.9	90.3	69.8	8.2	6.9	5.4
P0AAE5	YdgI	Cell inner membrane	Monomer	92.2	77.9	60.1	5.8	4.9	3.8

UniProtKB A. N.	Protein name	Subcellular location	Subunit	D_0 $\left(\frac{\mu m^2}{s}\right)$			D_{cyto} $\left(\frac{\mu m^2}{s}\right)$		
				310 K	303 K	293 K	310 K	303 K	293 K
			Complex with SecB	65.4	53.5	42.7	2.4	1.9	1.6
			Complex with TF (Tig)	70.6	59.6	46.1	2.9	2.5	1.9
P77376	YdgJ		Monomer	102.0	86.1	66.5	7.3	6.2	4.8
P76180	YdgK	Cell inner membrane	Monomer	142.4	120.3	92.9	15.6	13.2	10.2
			Complex with SecB	74.4	60.8	48.6	3.4	2.7	2.2
			Complex with TF (Tig)	83.1	70.2	54.2	4.4	3.8	2.9
A5A617	YdgU		Monomer	268.5	226.8	175.2	55.0	46.5	35.9
P0ACR2	YdhB		Monomer	105.3	88.9	68.7	7.9	6.7	5.2
P37597	YdhC	Cell inner membrane	Monomer	97.1	82.0	63.3	6.5	5.5	4.3
			Complex with SecB	66.8	54.6	43.6	2.5	2.1	1.6
			Complex with TF (Tig)	72.4	61.2	47.3	3.1	2.6	2.0
P76187	YdhF		Monomer	107.2	90.5	69.9	8.3	7.0	5.4
P64471	YdhI	Cell membrane	Monomer	180.7	152.6	117.9	25.7	21.7	16.8
			Complex with SecB	77.1	63.0	50.3	3.7	3.0	2.4
			Complex with TF (Tig)	87.2	73.6	56.9	5.0	4.2	3.3
P76185	YdhJ	Membrane	Monomer	110.0	93.0	71.8	8.8	7.4	5.7
			Complex with SecB	69.8	57.0	45.5	2.8	2.3	1.8
			Complex with TF (Tig)	76.5	64.6	49.9	3.6	3.0	2.3
P76186	YdhK	Cell membrane	Monomer	78.2	66.1	51.0	3.8	3.2	2.5
			Complex with SecB	60.6	49.5	39.5	1.9	1.6	1.3
			Complex with TF (Tig)	64.4	54.4	42.0	2.3	1.9	1.5
P64474	YdhL		Monomer	177.1	149.6	115.5	24.7	20.8	16.1
P76190	YdhO		Monomer	112.3	94.8	73.3	9.2	7.8	6.0
P77389	YdhP	Cell inner membrane	Monomer	100.1	84.6	65.3	7.0	5.9	4.6
			Complex with SecB	67.6	55.2	44.1	2.6	2.1	1.7
			Complex with TF (Tig)	73.5	62.1	47.9	3.2	2.7	2.1
P77552	YdhQ		Monomer	97.5	82.4	63.6	6.6	5.6	4.3
P0ACX3	YdhR		Monomer	164.5	139.0	107.4	21.2	17.9	13.8
			Homodimer	125.4	102.4	81.8	11.8	9.7	7.7
P77148	YdhS		Monomer	85.0	71.9	55.5	4.7	4.0	3.1
P77147	YdhT		Monomer	113.1	95.6	73.8	9.4	7.9	6.1

UniProtKB A. N.	Protein name	Subcellular location	Subunit	D_0 $\left(\frac{\mu m^2}{s}\right)$			D_{cyto} $\left(\frac{\mu m^2}{s}\right)$		
				310 K	303 K	293 K	310 K	303 K	293 K
P77409	YdhU	Cell inner membrane	Monomer	112.8	95.3	73.6	9.3	7.8	6.1
			Complex with SecB	70.3	57.4	45.9	2.9	2.4	1.9
			Complex with TF (Tig)	77.2	65.2	50.4	3.7	3.1	2.4
P76192	YdhV		Monomer	77.2	65.2	50.4	3.7	3.1	2.4
P77564	YdhW		Monomer	121.6	102.7	79.3	11.0	9.3	7.2
P77375	YdhX		Monomer	120.3	101.6	78.5	10.8	9.1	7.0
P0AAL6	YdhY		Monomer	125.8	106.3	82.1	11.9	10.1	7.8
P0ACX5	YdhZ		Monomer	189.2	159.8	123.4	28.2	23.8	18.4
P0A8A4	YdiA		Monomer	110.4	93.3	72.1	8.8	7.5	5.8
P0A6D5	YdiB		Monomer	110.4	93.3	72.0	8.8	7.5	5.8
			Homodimer	84.1	68.7	54.9	4.6	3.7	3.0
P0ACX9	YdiE		Monomer	197.1	166.5	128.6	30.6	25.8	20.0
P37766	YdiF		Monomer	86.9	73.4	56.7	5.0	4.2	3.2
P64476	YdiH		Monomer	195.5	165.2	127.6	30.1	25.4	19.6
P77781	YdiI		Monomer	147.4	124.5	96.2	16.8	14.2	11.0
			Homodimer	112.3	91.8	73.3	9.2	7.5	6.0
			Homotetramer	85.6	72.3	55.9	4.8	4.0	3.1
P77748	YdiJ		Monomer	66.6	56.3	43.5	2.5	2.1	1.6
P0AFS7	YdiK	Cell inner membrane	Monomer	100.3	84.8	65.5	7.1	6.0	4.6
			Complex with SecB	67.6	55.3	44.1	2.6	2.1	1.7
			Complex with TF (Tig)	73.5	62.1	48.0	3.2	2.7	2.1
P76196	YdiL		Monomer	151.5	128.0	98.9	17.8	15.1	11.6
P76197	YdiM	Cell inner membrane	Monomer	96.0	81.1	62.6	6.4	5.4	4.1
			Complex with SecB	66.5	54.3	43.4	2.5	2.0	1.6
			Complex with TF (Tig)	72.0	60.9	47.0	3.1	2.6	2.0
P76198	YdiN	Cell inner membrane	Monomer	94.8	80.1	61.9	6.2	5.2	4.0
			Complex with SecB	66.2	54.1	43.2	2.5	2.0	1.6
			Complex with TF (Tig)	71.6	60.5	46.7	3.0	2.6	2.0
P0A9U8	YdiO		Monomer	97.4	82.3	63.6	6.6	5.6	4.3
P77402	YdiP		Monomer	105.7	89.3	69.0	8.0	6.8	5.2
P76201	YdiQ		Monomer	116.1	98.1	75.8	9.9	8.4	6.5

UniProtKB A. N.	Protein name	Subcellular location	Subunit	D_0 $\left(\frac{\mu m^2}{s}\right)$			D_{cyto} $\left(\frac{\mu m^2}{s}\right)$		
				310 K	303 K	293 K	310 K	303 K	293 K
			Heterodimer (YdiQ−YdiR)	84.8	71.6	55.3	4.7	4.0	3.1
P77378	YdiR		Monomer	107.1	90.5	69.9	8.2	7.0	5.4
			Heterodimer (YdiQ−YdiR)	84.8	71.6	55.3	4.7	4.0	3.1
P77337	YdiS		Monomer	94.9	80.2	61.9	6.2	5.2	4.0
P77714	YdiT		Monomer	167.9	141.8	109.5	22.1	18.7	14.4
P77649	YdiU		Monomer	88.8	75.0	58.0	5.3	4.4	3.4
P76204	YdiV		Monomer	116.8	98.7	76.2	10.1	8.5	6.6
P76206	YdiY		Monomer	115.9	97.9	75.6	9.9	8.4	6.5
P64479	YdiZ		Monomer	167.0	141.1	109.0	21.9	18.5	14.3
P0ACY1	YdjA		Monomer	131.3	110.9	85.7	13.1	11.1	8.5
			Homodimer	100.1	81.8	65.3	7.0	5.7	4.6
P38055	YdjE	Cell inner membrane	Monomer	91.6	77.4	59.8	5.7	4.8	3.7
			Complex with SecB	65.3	53.3	42.6	2.4	1.9	1.5
			Complex with TF (Tig)	70.4	59.4	45.9	2.9	2.4	1.9
P77721	YdjF		Monomer	114.8	97.0	74.9	9.7	8.2	6.3
P77256	YdjG		Monomer	104.0	87.9	67.9	7.7	6.5	5.0
P77493	YdjH		Monomer	106.6	90.1	69.6	8.2	6.9	5.3
P77704	YdjI		Monomer	111.0	93.8	72.4	9.0	7.6	5.8
P77280	YdjJ		Monomer	102.5	86.6	66.9	7.4	6.3	4.9
P76230	YdjK	Cell inner membrane	Monomer	92.1	77.8	60.1	5.7	4.9	3.7
			Complex with SecB	65.4	53.4	42.7	2.4	1.9	1.6
			Complex with TF (Tig)	70.6	59.6	46.1	2.9	2.5	1.9
P77539	YdjL		Monomer	101.5	85.7	66.2	7.3	6.1	4.7
P64481	YdjM	Cell inner membrane	Monomer	125.9	106.3	82.1	11.9	10.1	7.8
			Complex with SecB	72.4	59.2	47.3	3.1	2.6	2.0
			Complex with TF (Tig)	80.2	67.8	52.3	4.1	3.4	2.7
P77529	YdjN	Cell membrane	Monomer	92.8	78.4	60.5	5.8	4.9	3.8
			Complex with SecB	65.6	53.6	42.8	2.4	2.0	1.6
			Complex with TF (Tig)	70.8	59.8	46.2	2.9	2.5	1.9
P76210	YdjO		Monomer	111.9	94.5	73.0	9.1	7.7	6.0
P76219	YdjX	Cell membrane	Monomer	118.5	100.1	77.3	10.4	8.8	6.8

UniProtKB A. N.	Protein name	Subcellular location	Subunit	D_0 $\left(\frac{\mu m^2}{s}\right)$			D_{cyto} $\left(\frac{\mu m^2}{s}\right)$		
				310 K	303 K	293 K	310 K	303 K	293 K
			Complex with SecB	71.3	58.3	46.5	3.0	2.4	2.0
			Complex with TF (Tig)	78.6	66.4	51.3	3.9	3.3	2.5
P76220	YdjY		Monomer	122.2	103.2	79.7	11.1	9.4	7.3
P76221	YdjZ	Cell inner membrane	Monomer	118.3	100.0	77.2	10.4	8.8	6.8
			Complex with SecB	71.3	58.2	46.5	3.0	2.4	2.0
			Complex with TF (Tig)	78.6	66.4	51.3	3.9	3.3	2.5
P76231	YeaC		Monomer	170.3	143.9	111.1	22.8	19.2	14.9
P39173	YeaD		Monomer	108.5	91.6	70.8	8.5	7.2	5.5
P76234	YeaE		Monomer	110.7	93.6	72.3	8.9	7.5	5.8
P0ACY3	YeaG		Monomer	78.5	66.3	51.2	3.9	3.3	2.5
P76235	YeaH		Monomer	92.2	77.9	60.2	5.8	4.9	3.8
P76236	YeaI	Cell inner membrane	Monomer	87.7	74.1	57.3	5.1	4.3	3.3
			Complex with SecB	64.1	52.3	41.8	2.2	1.8	1.5
			Complex with TF (Tig)	68.8	58.1	44.9	2.7	2.3	1.8
P76237	YeaJ	Cell inner membrane	Monomer	87.4	73.9	57.1	5.1	4.3	3.3
			Complex with SecB	64.0	52.2	41.7	2.2	1.8	1.5
			Complex with TF (Tig)	68.7	58.0	44.8	2.7	2.3	1.8
P64483	YeaK		Monomer	137.5	116.1	89.7	14.5	12.2	9.4
P0ACY6	YeaL	Cell membrane	Monomer	146.2	123.5	95.4	16.5	14.0	10.8
			Complex with SecB	74.8	61.1	48.8	3.4	2.8	2.2
			Complex with TF (Tig)	83.6	70.6	54.6	4.5	3.8	2.9
P76241	YeaM		Monomer	111.3	94.1	72.7	9.0	7.6	5.9
P76242	YeaN	Cell inner membrane	Monomer	99.0	83.7	64.6	6.8	5.8	4.5
			Complex with SecB	67.3	55.0	43.9	2.6	2.1	1.7
			Complex with TF (Tig)	73.1	61.8	47.7	3.2	2.7	2.1
P76243	YeaO		Monomer	153.9	130.0	100.4	18.4	15.6	12.0
P76245	YeaP		Monomer	101.7	85.9	66.3	7.3	6.2	4.8
			Homodimer	77.5	63.3	50.6	3.7	3.0	2.4
P64485	YeaQ	Cell membrane	Monomer	182.4	154.1	119.0	26.2	22.1	17.1
			Complex with SecB	77.2	63.1	50.4	3.7	3.0	2.4
			Complex with TF (Tig)	87.3	73.7	57.0	5.0	4.3	3.3

UniProtKB A. N.	Protein name	Subcellular location	Subunit	D_0 $\left(\frac{\mu m^2}{s}\right)$			D_{cyto} $\left(\frac{\mu m^2}{s}\right)$		
				310 K	303 K	293 K	310 K	303 K	293 K
P64488	YeaR		Monomer	152.9	129.2	99.8	18.2	15.4	11.9
			Monomer	89.8	75.9	58.6	5.4	4.6	3.5
P0ABD1	YeaV	Cell inner membrane	Complex with SecB	64.7	52.9	42.2	2.3	1.9	1.5
			Complex with TF (Tig)	69.7	58.9	45.5	2.8	2.4	1.8
P0ABR7	YeaW		Monomer	97.8	82.6	63.8	6.6	5.6	4.3
			Heterodimer (YeaW−YeaX)	77.0	65.1	50.3	3.7	3.1	2.4
P76254	YeaX		Monomer	104.8	88.5	68.4	7.8	6.6	5.1
			Heterodimer (YeaW−YeaX)	77.0	65.1	50.3	3.7	3.1	2.4
			Monomer	129.2	109.1	84.3	12.6	10.7	8.2
P0AA91	YeaY	Cell membrane	Complex with SecB	72.9	59.6	47.6	3.2	2.6	2.1
			Complex with TF (Tig)	80.9	68.3	52.8	4.2	3.5	2.7
P76256	YeaZ		Monomer	120.1	101.5	78.4	10.7	9.1	7.0
			Monomer	92.5	78.1	60.4	5.8	4.9	3.8
P0AFS9	YebA	Cell membrane	Complex with SecB	65.5	53.5	42.8	2.4	2.0	1.6
			Complex with TF (Tig)	70.7	59.8	46.2	2.9	2.5	1.9
P24238	YebB		Monomer	125.1	105.7	81.6	11.8	9.9	7.7
P0A8A0	YebC		Monomer	117.9	99.6	76.9	10.3	8.7	6.7
			Monomer	123.0	103.9	80.3	11.3	9.6	7.4
P33218	YebE	Cell inner membrane	Complex with SecB	72.0	58.9	47.0	3.1	2.5	2.0
			Complex with TF (Tig)	79.6	67.3	52.0	4.0	3.4	2.6
			Monomer	155.8	131.7	101.7	18.9	16.0	12.4
P33219	YebF	Secreted	Complex with SecB	75.6	61.8	49.3	3.5	2.9	2.3
			Complex with TF (Tig)	84.8	71.7	55.4	4.7	4.0	3.1
P0ACY9	YebG		Monomer	167.9	141.8	109.6	22.1	18.7	14.4
			Monomer	131.2	110.8	85.6	13.1	11.0	8.5
P76264	YebN	Cell inner membrane	Complex with SecB	73.2	59.8	47.7	3.2	2.6	2.1
			Complex with TF (Tig)	81.2	68.6	53.0	4.2	3.5	2.7
			Monomer	167.4	141.5	109.3	22.0	18.6	14.4
P64499	YebO	Cell membrane	Complex with SecB	76.4	62.4	49.8	3.6	2.9	2.3
			Complex with TF (Tig)	86.0	72.7	56.1	4.9	4.1	3.2
P76269	YebQ	Cell inner membrane	Monomer	93.2	78.7	60.8	5.9	5.0	3.9

UniProtKB A. N.	Protein name	Subcellular location	Subunit	D_0 $\left(\frac{\mu m^2}{s}\right)$			D_{cyto} $\left(\frac{\mu m^2}{s}\right)$		
				310 K	303 K	293 K	310 K	303 K	293 K
			Complex with SecB	65.7	53.7	42.9	2.4	2.0	1.6
			Complex with TF (Tig)	71.0	60.0	46.3	3.0	2.5	1.9
P76270	YebR		Monomer	136.7	115.4	89.2	14.3	12.1	9.3
P0AD03	YebS	Cell inner membrane	Monomer	93.1	78.6	60.7	5.9	5.0	3.8
			Complex with SecB	65.7	53.7	42.9	2.4	2.0	1.6
			Complex with TF (Tig)	71.0	59.9	46.3	3.0	2.5	1.9
P76272	YebT	Membrane	Monomer	71.4	60.3	46.6	3.0	2.5	2.0
			Complex with SecB	57.6	47.1	37.6	1.7	1.4	1.1
			Complex with TF (Tig)	60.8	51.3	39.7	1.9	1.6	1.3
P64503	YebV		Monomer	181.8	153.6	118.6	26.0	22.0	17.0
P76275	YebW		Monomer	195.0	164.7	127.3	29.9	25.3	19.5
P64506	YebY		Monomer	158.7	134.1	103.5	19.7	16.6	12.8
P76278	YebZ	Cell inner membrane	Monomer	108.7	91.8	70.9	8.5	7.2	5.6
			Complex with SecB	69.5	56.8	45.4	2.8	2.3	1.8
			Complex with TF (Tig)	76.1	64.3	49.6	3.6	3.0	2.3
P0AD05	YecA		Monomer	120.4	101.7	78.6	10.8	9.1	7.0
P37774	YecC	Cell inner membrane	Monomer	115.8	97.8	75.5	9.9	8.3	6.4
			Complex with SecB	70.9	57.9	46.2	2.9	2.4	1.9
			Complex with TF (Tig)	78.0	65.9	50.9	3.8	3.2	2.5
P0ADI7	YecD		Monomer	130.3	110.1	85.0	12.9	10.9	8.4
P37348	YecE		Monomer	110.1	93.0	71.8	8.8	7.4	5.7
P0AD07	YecF		Monomer	186.1	157.2	121.5	27.3	23.0	17.8
P46887	YecH		Monomer	182.8	154.4	119.3	26.3	22.2	17.2
P0AD10	YecJ		Monomer	178.8	151.1	116.7	25.2	21.3	16.4
P52007	YecM		Monomer	128.5	108.6	83.8	12.5	10.5	8.1
P64515	YecN	Cell inner membrane	Monomer	146.3	123.6	95.5	16.6	14.0	10.8
			Complex with SecB	74.8	61.1	48.8	3.4	2.8	2.2
			Complex with TF (Tig)	83.6	70.7	54.6	4.5	3.8	3.0
P76308	YecR		Monomer	162.0	136.9	105.7	20.5	17.3	13.4
P0AFT2	YecS	Cell inner membrane	Monomer	120.8	102.1	78.9	10.9	9.2	7.1
			Complex with SecB	71.7	58.6	46.8	3.0	2.5	2.0

UniProtKB A. N.	Protein name	Subcellular location	Subunit	D_0 $\left(\frac{\mu m^2}{s}\right)$ 310 K	D_0 303 K	D_0 293 K	D_{cyto} $\left(\frac{\mu m^2}{s}\right)$ 310 K	D_{cyto} 303 K	D_{cyto} 293 K
			Complex with TF (Tig)	79.1	66.9	51.6	3.9	3.3	2.6
P76296	YecT		Monomer	134.5	113.6	87.7	13.8	11.7	9.0
P0AA70	YedA	Cell inner membrane	Monomer	109.1	92.2	71.2	8.6	7.3	5.6
			Complex with SecB	69.6	56.9	45.4	2.8	2.3	1.8
			Complex with TF (Tig)	76.2	64.4	49.7	3.6	3.0	2.3
P31063	YedD	Cell membrane	Monomer	147.2	124.4	96.1	16.8	14.2	11.0
			Complex with SecB	74.9	61.2	48.9	3.4	2.8	2.2
			Complex with TF (Tig)	83.8	70.8	54.7	4.5	3.8	3.0
P31064	YedE	Cell inner membrane	Monomer	96.1	81.2	62.7	6.4	5.4	4.2
			Complex with SecB	66.6	54.4	43.4	2.5	2.0	1.6
			Complex with TF (Tig)	72.1	60.9	47.0	3.1	2.6	2.0
P0AA31	YedF		Monomer	182.7	154.3	119.2	26.3	22.2	17.1
P46125	YedI	Cell inner membrane	Monomer	109.1	92.2	71.2	8.6	7.3	5.6
			Complex with SecB	69.6	56.9	45.4	2.8	2.3	1.8
			Complex with TF (Tig)	76.2	64.4	49.7	3.6	3.0	2.3
P46144	YedJ		Monomer	118.8	100.4	77.5	10.5	8.8	6.8
P76318	YedK		Monomer	120.5	101.8	78.6	10.8	9.1	7.1
P76319	YedL		Monomer	137.3	116.0	89.6	14.4	12.2	9.4
P76322	YedM		Monomer	153.8	130.0	100.4	18.4	15.6	12.0
P76321	YedN		Monomer	131.6	111.2	85.9	13.2	11.1	8.6
P76329	YedP	Cytoplasm	Monomer	111.5	94.2	72.8	9.1	7.6	5.9
P76330	YedQ	Cell inner membrane	Monomer	83.2	70.3	54.3	4.5	3.8	2.9
			Complex with SecB	62.5	51.0	40.8	2.1	1.7	1.4
			Complex with TF (Tig)	66.8	56.4	43.6	2.5	2.1	1.6
P76334	YedR	Cell inner membrane	Monomer	151.9	128.3	99.1	17.9	15.2	11.7
			Complex with SecB	75.3	61.5	49.1	3.5	2.8	2.3
			Complex with TF (Tig)	84.4	71.3	55.0	4.6	3.9	3.0
P76335	YedS	Cell outer membrane	Monomer	97.4	82.3	63.6	6.6	5.6	4.3
			Complex with SecB	66.9	54.6	43.7	2.5	2.1	1.6
			Complex with TF (Tig)	72.5	61.3	47.3	3.1	2.6	2.0
P76339	YedV	Cell inner membrane	Monomer	91.2	77.0	59.5	5.6	4.7	3.7

UniProtKB A. N.	Protein name	Subcellular location	Subunit	D_0 $\left(\frac{\mu m^2}{s}\right)$			D_{cyto} $\left(\frac{\mu m^2}{s}\right)$		
				310 K	303 K	293 K	310 K	303 K	293 K
			Complex with SecB	65.1	53.2	42.5	2.4	1.9	1.5
			Complex with TF (Tig)	70.2	59.3	45.8	2.9	2.4	1.9
P76340	YedW	Cytoplasm	Monomer	120.4	101.7	78.6	10.8	9.1	7.0
P76342	YedY	Periplasm	Monomer	102.9	86.9	67.1	7.5	6.3	4.9
			Complex with SecB	68.3	55.8	44.5	2.7	2.2	1.7
			Complex with TF (Tig)	74.4	62.8	48.5	3.3	2.8	2.2
P76343	YedZ	Cell inner membrane	Monomer	122.3	103.3	79.8	11.2	9.4	7.3
			Complex with SecB	71.9	58.8	46.9	3.1	2.5	2.0
			Complex with TF (Tig)	79.5	67.1	51.9	4.0	3.4	2.6
P33011	YeeA	Cell inner membrane	Monomer	100.2	84.7	65.4	7.0	6.0	4.6
			Complex with SecB	67.6	55.2	44.1	2.6	2.1	1.7
			Complex with TF (Tig)	73.5	62.1	48.0	3.2	2.7	2.1
P33014	YeeD		Monomer	186.9	157.9	122.0	27.5	23.2	18.0
P33015	YeeE	Cell inner membrane	Monomer	102.1	86.3	66.6	7.4	6.2	4.8
			Complex with SecB	68.1	55.6	44.4	2.6	2.2	1.7
			Complex with TF (Tig)	74.1	62.6	48.4	3.3	2.8	2.2
P0AA47	YeeF	Cell inner membrane	Monomer	92.1	77.8	60.1	5.7	4.9	3.8
			Complex with SecB	65.4	53.4	42.7	2.4	1.9	1.6
			Complex with TF (Tig)	70.6	59.6	46.1	2.9	2.5	1.9
P76347	YeeJ		Monomer	49.0	41.4	31.9	1.0	0.9	0.7
P76349	YeeL		Monomer	100.8	85.1	65.8	7.1	6.0	4.7
P0A8A2	YeeN		Monomer	118.9	100.4	77.6	10.5	8.9	6.8
P76352	YeeO	Cell inner membrane	Monomer	88.9	75.1	58.0	5.3	4.5	3.4
			Complex with SecB	64.4	52.6	42.1	2.3	1.9	1.5
			Complex with TF (Tig)	69.3	58.6	45.2	2.8	2.3	1.8
P76359	YeeP		Monomer	116.8	98.6	76.2	10.1	8.5	6.6
P76361	YeeR	Cell inner membrane	Monomer	87.1	73.6	56.8	5.0	4.2	3.3
			Complex with SecB	63.8	52.1	41.7	2.2	1.8	1.5
			Complex with TF (Tig)	68.5	57.9	44.7	2.7	2.3	1.8
P76362	YeeS		Monomer	141.6	119.6	92.4	15.4	13.0	10.1
P64521	YeeT		Monomer	184.6	156.0	120.5	26.8	22.7	17.5

UniProtKB A. N.	Protein name	Subcellular location	Subunit	D_0 $\left(\frac{\mu m^2}{s}\right)$			D_{cyto} $\left(\frac{\mu m^2}{s}\right)$		
				310 K	303 K	293 K	310 K	303 K	293 K
P76364	YeeU		Monomer	152.6	128.9	99.6	18.1	15.3	11.8
P64526	YeeW		Monomer	198.9	168.0	129.8	31.1	26.3	20.3
P0A8M6	YeeX		Monomer	156.7	132.4	102.3	19.2	16.2	12.5
P76369	YeeY		Monomer	106.6	90.0	69.5	8.1	6.9	5.3
P0AD12	YeeZ		Monomer	112.6	95.1	73.5	9.3	7.8	6.0
P37749	YefG	Cytoplasm	Monomer	102.5	86.6	66.9	7.4	6.3	4.8
P37751	YefI	Cell inner membrane	Monomer	97.2	82.1	63.4	6.6	5.5	4.3
			Complex with SecB	66.8	54.6	43.6	2.5	2.1	1.6
			Complex with TF (Tig)	72.5	61.2	47.3	3.1	2.6	2.0
P69346	YefM	other	Monomer	177.4	149.9	115.8	24.8	20.9	16.2
			Complex with SecB	77.0	62.9	50.2	3.7	3.0	2.4
			Complex with TF (Tig)	86.9	73.4	56.7	5.0	4.2	3.2
P36928	YegD		Monomer	92.3	77.9	60.2	5.8	4.9	3.8
P38097	YegE		Monomer	64.3	54.3	42.0	2.3	1.9	1.5
			Homodimer	49.0	40.0	32.0	1.0	0.9	0.7
P76389	YegH	Cell membrane	Monomer	85.8	72.5	56.0	4.8	4.1	3.1
			Complex with SecB	63.4	51.8	41.4	2.2	1.8	1.4
			Complex with TF (Tig)	68.0	57.4	44.4	2.6	2.2	1.7
P76393	YegI		Monomer	79.7	67.4	52.0	4.0	3.4	2.6
P76394	YegJ		Monomer	138.8	117.2	90.5	14.8	12.5	9.6
P76395	YegK		Monomer	116.5	98.4	76.0	10.0	8.5	6.5
P76396	YegL		Monomer	122.0	103.0	79.6	11.1	9.4	7.2
P76402	YegP		Monomer	160.5	135.6	104.7	20.1	17.0	13.1
P76403	YegQ		Monomer	91.0	76.8	59.4	5.6	4.7	3.6
P76406	YegR		Monomer	163.5	138.1	106.7	20.9	17.7	13.7
P76407	YegS	Cytoplasm	Monomer	109.3	92.3	71.3	8.6	7.3	5.6
P76417	YegT	Cell inner membrane	Monomer	93.8	79.2	61.2	6.0	5.1	3.9
			Complex with SecB	65.9	53.8	43.0	2.4	2.0	1.6
			Complex with TF (Tig)	71.2	60.2	46.5	3.0	2.5	1.9
P76418	YegU		Monomer	104.8	88.6	68.4	7.8	6.6	5.1
P76419	YegV		Monomer	106.3	89.8	69.4	8.1	6.8	5.3

UniProtKB A. N.	Protein name	Subcellular location	Subunit	D_0 $\left(\frac{\mu m^2}{s}\right)$			D_{cyto} $\left(\frac{\mu m^2}{s}\right)$		
				310 K	303 K	293 K	310 K	303 K	293 K
P0ACM5	YegW		Monomer	114.8	97.0	74.9	9.7	8.2	6.3
P76421	YegX		Monomer	109.9	92.8	71.7	8.7	7.4	5.7
P33340	YehA		Monomer	103.4	87.4	67.5	7.6	6.4	5.0
P33341	YehB	Cell outer membrane	Monomer	72.2	61.0	47.1	3.1	2.6	2.0
			Complex with SecB	58.0	47.4	37.8	1.7	1.4	1.1
			Complex with TF (Tig)	61.2	51.7	40.0	2.0	1.7	1.3
P33342	YehC	Periplasm	Monomer	117.6	99.3	76.7	10.2	8.6	6.7
			Complex with SecB	71.2	58.1	46.4	3.0	2.4	1.9
			Complex with TF (Tig)	78.4	66.2	51.2	3.8	3.2	2.5
P33343	YehD		Monomer	134.0	113.2	87.4	13.7	11.6	8.9
P33344	YehE		Monomer	171.6	145.0	112.0	23.1	19.5	15.1
P33345	YehH		Monomer	61.2	51.7	39.9	2.0	1.7	1.3
P33346	YehI		Monomer	61.6	52.1	40.2	2.0	1.7	1.3
P33347	YehK		Monomer	157.6	133.1	102.8	19.4	16.4	12.6
P33348	YehL		Monomer	100.5	84.9	65.6	7.1	6.0	4.6
P33349	YehM		Monomer	75.1	63.5	49.0	3.4	2.9	2.2
P33352	YehP		Monomer	98.2	82.9	64.1	6.7	5.7	4.4
P33353	YehQ		Monomer	81.5	68.9	53.2	4.2	3.6	2.8
P33354	YehR	Cell membrane	Monomer	141.5	119.5	92.3	15.4	13.0	10.1
			Complex with SecB	74.3	60.7	48.5	3.3	2.7	2.2
			Complex with TF (Tig)	82.9	70.1	54.1	4.4	3.7	2.9
P33355	YehS		Monomer	137.1	115.8	89.5	14.4	12.2	9.4
P0AFT5	YehT		Monomer	116.2	98.2	75.8	10.0	8.4	6.5
P0AD14	YehU	Cell inner membrane	Monomer	84.3	71.2	55.0	4.6	3.9	3.0
			Complex with SecB	62.9	51.4	41.0	2.1	1.7	1.4
			Complex with TF (Tig)	67.3	56.9	43.9	2.6	2.2	1.7
P33359	YehW	Cell inner membrane	Monomer	119.5	101.0	78.0	10.6	9.0	6.9
			Complex with SecB	71.5	58.4	46.7	3.0	2.5	2.0
			Complex with TF (Tig)	78.8	66.6	51.4	3.9	3.3	2.5
P33360	YehX		Monomer	106.3	89.8	69.3	8.1	6.8	5.3
P33361	YehY	Cell inner membrane	Monomer	99.1	83.7	64.7	6.9	5.8	4.5

UniProtKB A. N.	Protein name	Subcellular location	Subunit	D_0 $\left(\frac{\mu m^2}{s}\right)$			D_{cyto} $\left(\frac{\mu m^2}{s}\right)$		
				310 K	303 K	293 K	310 K	303 K	293 K
			Complex with SecB	67.3	55.0	43.9	2.6	2.1	1.7
			Complex with TF (Tig)	73.1	61.8	47.7	3.2	2.7	2.1
P25889	YeiA		Monomer	95.6	80.8	62.4	6.3	5.3	4.1
			Heterotetramer ($YeiA_2-YeiT_2$)	55.7	47.1	36.4	1.5	1.3	1.0
P25747	YeiB	Cell membrane	Monomer	97.0	82.0	63.3	6.5	5.5	4.3
			Complex with SecB	66.8	54.6	43.6	2.5	2.1	1.6
			Complex with TF (Tig)	72.4	61.2	47.3	3.1	2.6	2.0
P0ACR4	YeiE		Monomer	108.4	91.6	70.7	8.5	7.2	5.5
P33018	YeiG		Monomer	110.4	93.2	72.0	8.8	7.5	5.8
			Homotetramer	64.1	52.4	41.8	2.2	1.8	1.5
P62723	YeiH	Cell inner membrane	Monomer	103.4	87.4	67.5	7.6	6.4	5.0
			Complex with SecB	68.4	55.9	44.6	2.7	2.2	1.7
			Complex with TF (Tig)	74.5	63.0	48.6	3.4	2.8	2.2
P33020	YeiI		Monomer	100.5	84.9	65.6	7.1	6.0	4.6
P0A6N8	YeiP		Monomer	127.7	107.9	83.3	12.3	10.4	8.0
P33029	YeiQ		Monomer	89.0	75.2	58.1	5.3	4.5	3.5
P33030	YeiR		Monomer	104.3	88.1	68.1	7.7	6.5	5.1
P64536	YeiS	Membrane	Monomer	177.8	150.2	116.0	24.9	21.0	16.2
			Complex with SecB	77.0	62.9	50.2	3.7	3.0	2.4
			Complex with TF (Tig)	86.9	73.4	56.7	5.0	4.2	3.3
P76440	YeiT		Monomer	96.2	81.3	62.8	6.4	5.4	4.2
			Heterotetramer ($YeiA_2-YeiT_2$)	55.7	47.1	36.4	1.5	1.3	1.0
P76445	YeiU	Cell inner membrane	Monomer	117.3	99.1	76.5	10.2	8.6	6.6
			Complex with SecB	71.1	58.1	46.4	3.0	2.4	1.9
			Complex with TF (Tig)	78.3	66.2	51.1	3.8	3.2	2.5
P0AFT8	YeiW		Monomer	180.2	152.2	117.6	25.5	21.6	16.7
P33913	YejA		Monomer	80.6	68.1	52.6	4.1	3.5	2.7
P0AFU0	YejB	Cell inner membrane	Monomer	99.8	84.3	65.2	7.0	5.9	4.6
			Complex with SecB	67.5	55.2	44.1	2.6	2.1	1.7
			Complex with TF (Tig)	73.4	62.0	47.9	3.2	2.7	2.1
P33915	YejE	Cell inner membrane	Monomer	102.1	86.3	66.6	7.4	6.2	4.8

UniProtKB A. N.	Protein name	Subcellular location	Subunit	D_0 $\left(\frac{\mu m^2}{s}\right)$			D_{cyto} $\left(\frac{\mu m^2}{s}\right)$		
				310 K	303 K	293 K	310 K	303 K	293 K
			Complex with SecB	68.1	55.6	44.4	2.6	2.2	1.7
			Complex with TF (Tig)	74.1	62.6	48.4	3.3	2.8	2.2
P33916	YejF		Monomer	86.2	72.8	56.2	4.9	4.1	3.2
P0AD21	YejG		Monomer	157.9	133.4	103.1	19.5	16.5	12.7
P33919	YejH		Monomer	82.1	69.4	53.6	4.3	3.6	2.8
P33920	YejK	Cytoplasm > nucleoid	Monomer	102.4	86.5	66.8	7.4	6.3	4.8
P0AD24	YejL		Monomer	185.7	156.9	121.2	27.1	22.9	17.7
P0AD27	YejM	Cell inner membrane	Monomer	81.7	69.0	53.3	4.3	3.6	2.8
			Complex with SecB	61.9	50.6	40.4	2.0	1.7	1.3
			Complex with TF (Tig)	66.1	55.9	43.1	2.4	2.1	1.6
P33924	YejO	Cell outer membrane	Monomer	72.5	61.3	47.3	3.1	2.6	2.0
			Complex with SecB	58.1	47.5	37.9	1.7	1.4	1.1
			Complex with TF (Tig)	61.4	51.9	40.1	2.0	1.7	1.3
P17994	YfaA		Monomer	84.0	71.0	54.8	4.6	3.9	3.0
P37014	YfaD		Monomer	105.7	89.3	69.0	8.0	6.7	5.2
P0ABW3	YfaE		Monomer	177.5	150.0	115.9	24.8	20.9	16.2
P45505	YfaH		Monomer	192.9	163.0	125.9	29.3	24.8	19.1
P45508	YfaL		Monomer	62.9	53.1	41.0	2.1	1.8	1.4
P76462	YfaP		Monomer	114.7	96.9	74.9	9.7	8.2	6.3
P76463	YfaQ		Monomer	84.7	71.5	55.2	4.7	3.9	3.0
P76464	YfaS		Monomer	56.8	48.0	37.1	1.6	1.4	1.1
P76466	YfaT		Monomer	122.9	103.8	80.2	11.3	9.5	7.4
P77808	YfaY		Monomer	96.3	81.4	62.9	6.4	5.4	4.2
P76471	YfaZ		Monomer	135.2	114.2	88.2	14.0	11.8	9.1
P76481	YfbK		Monomer	83.5	70.6	54.5	4.5	3.8	2.9
P76482	YfbL	Membrane	Monomer	104.5	88.3	68.2	7.8	6.6	5.1
			Complex with SecB	68.6	56.1	44.8	2.7	2.2	1.8
			Complex with TF (Tig)	74.9	63.2	48.9	3.4	2.9	2.2
P76483	YfbM		Monomer	134.1	113.3	87.5	13.7	11.6	8.9
P76484	YfbN		Monomer	115.2	97.4	75.2	9.8	8.2	6.4

UniProtKB A. N.	Protein name	Subcellular location	Subunit	D_0 $\left(\frac{\mu m^2}{s}\right)$			D_{cyto} $\left(\frac{\mu m^2}{s}\right)$		
				310 K	303 K	293 K	310 K	303 K	293 K
P76485	YfbO		Monomer	142.9	120.7	93.3	15.8	13.3	10.3
P76486	YfbP		Monomer	109.0	92.1	71.1	8.6	7.3	5.6
P0A959	YfbQ	Cytoplasm	Monomer	95.2	80.5	62.2	6.2	5.3	4.1
			Homodimer	72.6	59.3	47.4	3.1	2.6	2.0
P76491	YfbR	Cytoplasm	Monomer	125.1	105.7	81.6	11.8	9.9	7.7
			Homooligomer	81.3	68.7	53.1	4.2	3.6	2.7
P0AFU2	YfbS	Cell inner membrane	Monomer	82.4	69.6	53.8	4.4	3.7	2.8
			Complex with SecB	62.2	50.8	40.6	2.1	1.7	1.4
			Complex with TF (Tig)	66.4	56.1	43.4	2.5	2.1	1.6
P77625	YfbT		Monomer	124.4	105.1	81.2	11.6	9.8	7.6
P0A8W8	YfbU		Monomer	132.7	112.1	86.6	13.4	11.3	8.7
P0A8D9	YfbV	Cell inner membrane	Monomer	139.4	117.8	91.0	14.9	12.6	9.7
			Complex with SecB	74.1	60.5	48.4	3.3	2.7	2.2
			Complex with TF (Tig)	82.6	69.8	53.9	4.4	3.7	2.9
P0AD30	YfcA	Cell inner membrane	Monomer	114.2	96.5	74.5	9.6	8.1	6.2
			Complex with SecB	70.6	57.7	46.1	2.9	2.4	1.9
			Complex with TF (Tig)	77.6	65.5	50.6	3.7	3.2	2.4
P39199	YfcB		Monomer	105.6	89.2	68.9	8.0	6.7	5.2
P39263	YfcC	Cell membrane	Monomer	88.6	74.8	57.8	5.2	4.4	3.4
			Complex with SecB	64.3	52.5	42.0	2.3	1.9	1.5
			Complex with TF (Tig)	69.2	58.4	45.1	2.8	2.3	1.8
P65556	YfcD		Monomer	130.5	110.3	85.2	12.9	10.9	8.4
P67095	YfcE		Monomer	131.2	110.8	85.6	13.1	11.0	8.5
P77544	YfcF		Monomer	121.8	102.9	79.5	11.1	9.3	7.2
P77526	YfcG		Monomer	121.4	102.5	79.2	11.0	9.3	7.2
P77775	YfcH		Monomer	108.4	91.6	70.8	8.5	7.2	5.5
P77768	YfcI		Monomer	106.6	90.0	69.5	8.1	6.9	5.3
P77549	YfcJ	Cell inner membrane	Monomer	99.7	84.3	65.1	7.0	5.9	4.5
			Complex with SecB	67.5	55.1	44.0	2.6	2.1	1.7
			Complex with TF (Tig)	73.3	62.0	47.9	3.2	2.7	2.1
P64540	YfcL		Monomer	172.5	145.7	112.6	23.4	19.8	15.3

UniProtKB A. N.	Protein name	Subcellular location	Subunit	D_0 $\left(\frac{\mu m^2}{s}\right)$			D_{cyto} $\left(\frac{\mu m^2}{s}\right)$		
				310 K	303 K	293 K	310 K	303 K	293 K
P76938	YfcM		Monomer	128.6	108.7	83.9	12.5	10.6	8.2
P0A8B2	YfcN		Monomer	128.9	108.9	84.1	12.6	10.6	8.2
P76498	YfcO		Monomer	111.8	94.4	72.9	9.1	7.7	5.9
P76499	YfcP	Fimbrium	Monomer	134.3	113.4	87.6	13.8	11.6	9.0
			Complex with SecB	73.5	60.1	48.0	3.2	2.7	2.1
			Complex with TF (Tig)	81.8	69.1	53.4	4.3	3.6	2.8
P76500	YfcQ		Monomer	137.3	116.0	89.6	14.4	12.2	9.4
P76501	YfcR		Monomer	137.3	116.0	89.6	14.4	12.2	9.4
P77599	YfcS	Periplasm	Monomer	115.7	97.7	75.5	9.8	8.3	6.4
			Complex with SecB	70.8	57.9	46.2	2.9	2.4	1.9
			Complex with TF (Tig)	77.9	65.8	50.9	3.8	3.2	2.5
P77196	YfcU	Cell outer membrane	Monomer	70.7	59.7	46.1	2.9	2.5	1.9
			Complex with SecB	57.3	46.8	37.4	1.6	1.3	1.1
			Complex with TF (Tig)	60.4	51.0	39.4	1.9	1.6	1.2
P77288	YfcV		Monomer	131.9	111.4	86.0	13.2	11.2	8.6
P0AD33	YfcZ		Monomer	170.4	144.0	111.2	22.8	19.3	14.9
P37327	YfdC	Cell inner membrane	Monomer	106.2	89.7	69.3	8.1	6.8	5.3
			Complex with SecB	69.0	56.4	45.0	2.7	2.2	1.8
			Complex with TF (Tig)	75.4	63.7	49.2	3.5	2.9	2.3
P76518	YfdE		Monomer	98.6	83.3	64.3	6.8	5.7	4.4
P76505	YfdF		Monomer	99.9	84.4	65.2	7.0	5.9	4.6
P77682	YfdG	Cell inner membrane	Monomer	154.6	130.6	100.9	18.6	15.7	12.2
			Complex with SecB	75.5	61.7	49.3	3.5	2.8	2.3
			Complex with TF (Tig)	84.7	71.5	55.3	4.7	3.9	3.0
P77293	YfdH	Cell inner membrane	Monomer	106.0	89.6	69.2	8.0	6.8	5.3
			Complex with SecB	69.0	56.3	45.0	2.7	2.2	1.8
			Complex with TF (Tig)	75.3	63.6	49.1	3.5	2.9	2.3
P76507	YfdI	Cell membrane	Monomer	90.8	76.7	59.2	5.5	4.7	3.6
			Complex with SecB	65.0	53.1	42.4	2.3	1.9	1.5
			Complex with TF (Tig)	70.0	59.2	45.7	2.9	2.4	1.9
P77656	YfdK		Monomer	142.1	120.0	92.7	15.6	13.1	10.1

UniProtKB A. N.	Protein name	Subcellular location	Subunit	$D_0 \left(\frac{\mu m^2}{s}\right)$			$D_{\text{cyto}} \left(\frac{\mu m^2}{s}\right)$		
				310 K	303 K	293 K	310 K	303 K	293 K
P76508	YfdL		Monomer	136.3	115.1	88.9	14.2	12.0	9.3
P76509	YfdM		Monomer	170.3	143.9	111.1	22.8	19.2	14.9
P76510	YfdN		Monomer	134.8	113.9	88.0	13.9	11.7	9.1
P0AD35	YfdO		Monomer	149.5	126.3	97.6	17.4	14.7	11.3
P76512	YfdP		Monomer	155.2	131.1	101.3	18.8	15.9	12.2
P76513	YfdQ		Monomer	111.5	94.2	72.8	9.1	7.6	5.9
P76514	YfdR		Monomer	130.8	110.5	85.4	13.0	11.0	8.5
P76515	YfdS		Monomer	152.6	128.9	99.6	18.1	15.3	11.8
P76516	YfdT		Monomer	159.9	135.1	104.4	20.0	16.9	13.0
P0AA49	YfdV	Cell membrane	Monomer	107.3	90.6	70.0	8.3	7.0	5.4
			Complex with SecB	69.2	56.6	45.2	2.8	2.3	1.8
			Complex with TF (Tig)	75.7	63.9	49.4	3.5	3.0	2.3
P76520	YfdX		Monomer	124.5	105.2	81.2	11.6	9.8	7.6
P76521	YfdY	Cell membrane	Monomer	180.8	152.7	118.0	25.7	21.7	16.8
			Complex with SecB	77.1	63.0	50.3	3.7	3.0	2.4
			Complex with TF (Tig)	87.2	73.6	56.9	5.0	4.2	3.3
P77434	YfdZ	Cytoplasm	Monomer	94.7	80.0	61.8	6.1	5.2	4.0
			Homodimer	72.1	58.9	47.1	3.1	2.5	2.0
P23842	YfeA		Monomer	75.1	63.4	49.0	3.4	2.9	2.2
P0AD37	YfeC		Monomer	157.0	132.6	102.4	19.2	16.2	12.5
P27238	YfeD		Monomer	147.5	124.6	96.3	16.9	14.2	11.0
P39836	YfeH		Monomer	104.0	87.8	67.8	7.7	6.5	5.0
Q47702	YfeK		Monomer	152.3	128.7	99.4	18.1	15.3	11.8
P45564	YfeN		Monomer	113.3	95.7	73.9	9.4	7.9	6.1
P67729	YfeO	Cell membrane	Monomer	96.9	81.8	63.2	6.5	5.5	4.2
			Complex with SecB	66.8	54.5	43.6	2.5	2.1	1.6
			Complex with TF (Tig)	72.4	61.1	47.2	3.1	2.6	2.0
P0ACR7	YfeR		Monomer	106.9	90.3	69.8	8.2	6.9	5.4
P78271	YfeS		Monomer	114.8	97.0	74.9	9.7	8.2	6.3
P77619	YfeW		Monomer	93.5	79.0	61.0	6.0	5.0	3.9
P76536	YfeX		Monomer	108.0	91.2	70.5	8.4	7.1	5.5

UniProtKB A. N.	Protein name	Subcellular location	Subunit	D_0 $\left(\frac{\mu m^2}{s}\right)$			D_{cyto} $\left(\frac{\mu m^2}{s}\right)$		
				310 K	303 K	293 K	310 K	303 K	293 K
P76537	YfeY		Monomer	129.2	109.2	84.3	12.6	10.7	8.2
P76538	YfeZ	Cell inner membrane	Monomer	139.6	117.9	91.1	15.0	12.6	9.8
			Complex with SecB	74.1	60.6	48.4	3.3	2.7	2.2
			Complex with TF (Tig)	82.6	69.8	53.9	4.4	3.7	2.9
P24178	YffB		Monomer	152.9	129.2	99.8	18.2	15.4	11.9
P76543	YffL		Monomer	120.2	101.5	78.4	10.7	9.1	7.0
P76544	YffM		Monomer	178.2	150.5	116.3	25.0	21.1	16.3
P76545	YffN		Monomer	152.1	128.5	99.3	18.0	15.2	11.7
P76546	YffO		Monomer	144.5	122.0	94.3	16.1	13.6	10.5
P76547	YffP		Monomer	124.7	105.3	81.3	11.7	9.9	7.6
P76548	YffQ		Monomer	152.0	128.4	99.2	18.0	15.2	11.7
P76549	YffR		Monomer	148.9	125.8	97.2	17.2	14.5	11.2
P76550	YffS		Monomer	112.5	95.1	73.4	9.2	7.8	6.0
P66948	YfgC		Monomer	89.1	75.3	58.2	5.3	4.5	3.5
P76569	YfgD		Monomer	153.8	130.0	100.4	18.4	15.6	12.0
P77172	YfgF	Cell inner membrane	Monomer	74.4	62.8	48.5	3.3	2.8	2.2
			Complex with SecB	59.0	48.2	38.5	1.8	1.5	1.2
			Complex with TF (Tig)	62.4	52.7	40.7	2.1	1.8	1.4
P64545	YfgG	Membrane	Monomer	193.5	163.5	126.3	29.5	24.9	19.2
			Complex with SecB	77.7	63.5	50.7	3.7	3.1	2.4
			Complex with TF (Tig)	88.0	74.4	57.4	5.1	4.3	3.4
P65290	YfgH	Cell membrane	Monomer	137.9	116.5	90.0	14.6	12.3	9.5
			Complex with SecB	73.9	60.4	48.3	3.3	2.7	2.2
			Complex with TF (Tig)	82.4	69.6	53.8	4.4	3.7	2.8
P76573	YfgI		Monomer	130.9	110.6	85.4	13.0	11.0	8.5
P76575	YfgJ		Monomer	188.3	159.1	122.9	27.9	23.6	18.2
P77774	YfgL	Cell outer membrane	Monomer	98.4	83.1	64.2	6.7	5.7	4.4
			Complex with SecB	67.2	54.9	43.8	2.6	2.1	1.7
			Complex with TF (Tig)	72.9	61.6	47.6	3.2	2.7	2.1
P76576	YfgM	Membrane	Monomer	126.3	106.7	82.4	12.0	10.1	7.8
			Complex with SecB	72.5	59.2	47.3	3.1	2.6	2.0

UniProtKB A. N.	Protein name	Subcellular location	Subunit	D_0 $\left(\frac{\mu m^2}{s}\right)$			D_{cyto} $\left(\frac{\mu m^2}{s}\right)$		
				310 K	303 K	293 K	310 K	303 K	293 K
			Complex with TF (Tig)	80.3	67.8	52.4	4.1	3.4	2.7
P0AFU4	YfhA		Monomer	92.4	78.1	60.3	5.8	4.9	3.8
P0AD42	YfhB		Monomer	121.5	102.7	79.3	11.0	9.3	7.2
P0AD44	YfhG		Monomer	116.3	98.2	75.9	10.0	8.4	6.5
P37767	YfhH		Monomer	111.1	93.9	72.5	9.0	7.6	5.9
P52101	YfhK	Cell inner membrane	Monomer	89.5	75.6	58.4	5.4	4.5	3.5
			Complex with SecB	64.6	52.8	42.2	2.3	1.9	1.5
			Complex with TF (Tig)	69.5	58.8	45.4	2.8	2.4	1.8
P52102	YfhL		Monomer	174.0	147.0	113.5	23.8	20.1	15.5
P76578	YfhM	Cell membrane	Monomer	55.4	46.8	36.1	1.5	1.3	1.0
			Complex with SecB	48.8	39.9	31.8	1.0	0.8	0.7
			Complex with TF (Tig)	50.5	42.7	32.9	1.1	1.0	0.7
P77538	YfhR	Membrane	Monomer	110.0	92.9	71.8	8.8	7.4	5.7
			Complex with SecB	69.8	57.0	45.5	2.8	2.3	1.8
			Complex with TF (Tig)	76.5	64.6	49.9	3.6	3.0	2.3
P07021	YfiB	Cell membrane	Monomer	139.3	117.7	90.9	14.9	12.6	9.7
			Complex with SecB	74.1	60.5	48.4	3.3	2.7	2.2
			Complex with TF (Tig)	82.6	69.8	53.9	4.4	3.7	2.9
P31825	YfiC	Cytoplasm	Monomer	116.4	98.4	76.0	10.0	8.4	6.5
P33634	YfiE		Monomer	107.7	91.0	70.3	8.4	7.1	5.5
P0AGJ5	YfiF		Monomer	102.5	86.6	66.9	7.4	6.3	4.8
P33644	YfiH		Monomer	118.0	99.7	77.0	10.3	8.7	6.7
P11289	YfiL		Monomer	154.7	130.7	101.0	18.6	15.8	12.2
P46126	YfiM		Monomer	161.7	136.6	105.5	20.4	17.3	13.3
P46139	YfiN	Cell inner membrane	Monomer	94.9	80.1	61.9	6.2	5.2	4.0
			Complex with SecB	66.2	54.1	43.2	2.5	2.0	1.6
			Complex with TF (Tig)	71.6	60.5	46.7	3.0	2.6	2.0
P0AC02	YfiO	Cell outer membrane	Monomer	115.5	97.6	75.4	9.8	8.3	6.4
			Complex with SecB	70.8	57.9	46.2	2.9	2.4	1.9
			Complex with TF (Tig)	77.9	65.8	50.8	3.8	3.2	2.5
Q47319	YfiP		Monomer	118.6	100.2	77.4	10.4	8.8	6.8

UniProtKB A. N.	Protein name	Subcellular location	Subunit	D_0 $\left(\frac{\mu m^2}{s}\right)$			D_{cyto} $\left(\frac{\mu m^2}{s}\right)$		
				310 K	303 K	293 K	310 K	303 K	293 K
P76594	YfiQ		Monomer	70.5	59.6	46.0	2.9	2.5	1.9
P64548	YfiR		Monomer	134.2	113.4	87.6	13.7	11.6	9.0
P37908	YfjD	Cell inner membrane	Monomer	93.2	78.8	60.8	5.9	5.0	3.9
			Complex with SecB	65.7	53.7	42.9	2.4	2.0	1.6
			Complex with TF (Tig)	71.0	60.0	46.3	3.0	2.5	1.9
P52123	YfjH		Monomer	103.1	87.1	67.3	7.5	6.4	4.9
P52124	YfjI		Monomer	89.1	75.2	58.1	5.3	4.5	3.5
P52125	YfjJ		Monomer	121.3	102.5	79.2	11.0	9.3	7.2
P52126	YfjK		Monomer	75.2	63.6	49.1	3.4	2.9	2.3
P52127	YfjL		Monomer	84.4	71.3	55.1	4.6	3.9	3.0
P52128	YfjM		Monomer	172.7	145.9	112.7	23.4	19.8	15.3
P52129	YfjN		Monomer	100.1	84.6	65.3	7.0	5.9	4.6
P52130	YfjO		Monomer	152.6	129.0	99.6	18.1	15.3	11.8
P52131	YfjP		Monomer	109.2	92.3	71.3	8.6	7.3	5.6
P52132	YfjQ		Monomer	110.4	93.2	72.0	8.8	7.5	5.8
P52133	YfjR		Monomer	117.6	99.3	76.7	10.2	8.6	6.7
O52982	YfjS	Cell membrane	Monomer	141.0	119.1	92.0	15.3	12.9	10.0
			Complex with SecB	74.3	60.7	48.5	3.3	2.7	2.2
			Complex with TF (Tig)	82.9	70.0	54.1	4.4	3.7	2.9
P52135	YfjT		Monomer	139.8	118.1	91.2	15.0	12.7	9.8
P0CF86	YfjU		Monomer	213.8	180.6	139.5	35.8	30.3	23.4
P52137	YfjV	Cell inner membrane	Monomer	104.6	88.4	68.3	7.8	6.6	5.1
			Complex with SecB	68.7	56.1	44.8	2.7	2.2	1.8
			Complex with TF (Tig)	74.9	63.3	48.9	3.4	2.9	2.2
P52138	YfjW	Cell membrane	Monomer	83.2	70.3	54.3	4.5	3.8	2.9
			Complex with SecB	62.5	51.1	40.8	2.1	1.7	1.4
			Complex with TF (Tig)	66.8	56.5	43.6	2.5	2.1	1.6
P52139	YfjX		Monomer	139.2	117.6	90.8	14.9	12.6	9.7
P52140	YfjY		Monomer	137.1	115.8	89.5	14.4	12.2	9.4
P52141	YfjZ		Monomer	162.0	136.9	105.7	20.5	17.4	13.4
P0AD53	YgaC		Monomer	155.3	131.2	101.4	18.8	15.9	12.3

UniProtKB A. N.	Protein name	Subcellular location	Subunit	D_0 $\left(\frac{\mu m^2}{s}\right)$			D_{cyto} $\left(\frac{\mu m^2}{s}\right)$		
				310 K	303 K	293 K	310 K	303 K	293 K
P0A6G3	YgaD		Monomer	138.3	116.8	90.2	14.7	12.4	9.6
P43667	YgaH		Monomer	160.5	135.6	104.7	20.1	17.0	13.1
P0ADQ7	YgaM		Monomer	159.1	134.4	103.8	19.8	16.7	12.9
P55734	YgaP	Cell inner membrane	Monomer	135.2	114.2	88.2	14.0	11.8	9.1
			Complex with SecB	73.6	60.2	48.1	3.3	2.7	2.1
			Complex with TF (Tig)	81.9	69.2	53.5	4.3	3.6	2.8
P76616	YgaQ		Monomer	74.5	62.9	48.6	3.4	2.8	2.2
P0ADE6	YgaU		Monomer	143.3	121.0	93.5	15.8	13.4	10.3
P77295	YgaV		Monomer	168.6	142.5	110.1	22.3	18.9	14.6
			Homodimer	128.5	105.0	83.9	12.5	10.2	8.1
P64550	YgaW	Cell membrane	Monomer	140.3	118.5	91.5	15.1	12.8	9.9
			Complex with SecB	74.2	60.6	48.4	3.3	2.7	2.2
			Complex with TF (Tig)	82.8	69.9	54.0	4.4	3.7	2.9
P76628	YgaY	Cell inner membrane	Monomer	98.6	83.3	64.3	6.8	5.7	4.4
			Complex with SecB	67.2	54.9	43.9	2.6	2.1	1.7
			Complex with TF (Tig)	73.0	61.6	47.6	3.2	2.7	2.1
P76630	YgaZ	Cell inner membrane	Monomer	118.4	100.1	77.3	10.4	8.8	6.8
			Complex with SecB	71.3	58.3	46.5	3.0	2.4	2.0
			Complex with TF (Tig)	78.6	66.4	51.3	3.9	3.3	2.5
P25728	YgbA		Monomer	151.5	128.0	98.8	17.8	15.1	11.6
P46141	YgbE	Cell inner membrane	Monomer	160.5	135.6	104.8	20.2	17.0	13.1
			Complex with SecB	75.9	62.0	49.6	3.5	2.9	2.3
			Complex with TF (Tig)	85.3	72.1	55.7	4.8	4.0	3.1
P45956	YgbF		Monomer	169.1	142.9	110.4	22.5	19.0	14.7
P52598	YgbI		Monomer	116.1	98.1	75.8	9.9	8.4	6.5
Q46888	YgbJ		Monomer	111.1	93.8	72.5	9.0	7.6	5.9
Q46889	YgbK		Monomer	98.9	83.6	64.5	6.8	5.8	4.5
Q46890	YgbL		Monomer	124.0	104.8	80.9	11.5	9.7	7.5
Q46891	YgbM		Monomer	113.3	95.7	73.9	9.4	7.9	6.1
Q46892	YgbN	Cell inner membrane	Monomer	94.2	79.6	61.5	6.1	5.1	4.0
			Complex with SecB	66.0	53.9	43.1	2.4	2.0	1.6

UniProtKB A. N.	Protein name	Subcellular location	Subunit	D_0 $\left(\frac{\mu m^2}{s}\right)$			D_{cyto} $\left(\frac{\mu m^2}{s}\right)$		
				310 K	303 K	293 K	310 K	303 K	293 K
			Complex with TF (Tig)	71.4	60.3	46.6	3.0	2.5	2.0
Q46896	YgbT		Monomer	107.8	91.1	70.3	8.4	7.1	5.5
P38036	YgcB		Monomer	69.8	59.0	45.6	2.8	2.4	1.8
P55138	YgcE		Monomer	89.0	75.2	58.0	5.3	4.5	3.4
P64554	YgcF		Monomer	120.4	101.7	78.6	10.8	9.1	7.0
P55140	YgcG	Cell membrane	Monomer	109.1	92.1	71.2	8.6	7.3	5.6
			Complex with SecB	69.6	56.9	45.4	2.8	2.3	1.8
			Complex with TF (Tig)	76.2	64.4	49.7	3.6	3.0	2.3
Q46897	YgcH		Monomer	126.0	106.4	82.2	11.9	10.1	7.8
Q46898	YgcI		Monomer	120.1	101.4	78.3	10.7	9.1	7.0
Q46899	YgcJ		Monomer	100.2	84.6	65.4	7.0	5.9	4.6
P76632	YgcK		Monomer	135.0	114.0	88.1	13.9	11.8	9.1
Q46901	YgcL		Monomer	87.9	74.2	57.3	5.1	4.3	3.3
Q46904	YgcN		Monomer	94.5	79.9	61.7	6.1	5.2	4.0
Q46905	YgcO		Monomer	174.9	147.7	114.1	24.0	20.3	15.7
Q46906	YgcP		Monomer	129.5	109.4	84.5	12.7	10.7	8.3
Q46907	YgcQ		Monomer	111.7	94.4	72.9	9.1	7.7	5.9
			Heterodimer (YgcQ−YgcR)	86.0	72.7	56.1	4.9	4.1	3.2
Q46908	YgcR		Monomer	114.1	96.4	74.4	9.5	8.1	6.2
			Heterodimer (YgcQ−YgcR)	86.0	72.7	56.1	4.9	4.1	3.2
Q46909	YgcS	Cell inner membrane	Monomer	93.1	78.7	60.8	5.9	5.0	3.8
			Complex with SecB	65.7	53.7	42.9	2.4	2.0	1.6
			Complex with TF (Tig)	71.0	59.9	46.3	3.0	2.5	1.9
Q46911	YgcU		Monomer	89.2	75.4	58.2	5.3	4.5	3.5
P76633	YgcW		Monomer	115.2	97.3	75.1	9.7	8.2	6.4
P08370	YgdB		Monomer	147.3	124.4	96.1	16.8	14.2	11.0
P0ADR2	YgdD	Cell inner membrane	Monomer	149.8	126.6	97.8	17.4	14.7	11.4
			Complex with SecB	75.1	61.4	49.0	3.4	2.8	2.2
			Complex with TF (Tig)	84.1	71.0	54.9	4.6	3.9	3.0
P38506	YgdG		Monomer	115.0	97.1	75.0	9.7	8.2	6.3
P0ADR8	YgdH		Monomer	91.1	77.0	59.5	5.6	4.7	3.7

UniProtKB A. N.	Protein name	Subcellular location	Subunit	D_0 $\left(\frac{\mu m^2}{s}\right)$			D_{cyto} $\left(\frac{\mu m^2}{s}\right)$		
				310 K	303 K	293 K	310 K	303 K	293 K
P65292	YgdI	Cell membrane	Monomer	186.7	157.7	121.8	27.4	23.2	17.9
			Complex with SecB	77.4	63.2	50.5	3.7	3.0	2.4
			Complex with TF (Tig)	87.6	74.0	57.2	5.1	4.3	3.3
P0AGF2	YgdK		Monomer	143.7	121.4	93.8	15.9	13.5	10.4
Q46927	YgdL	Membrane	Monomer	114.3	96.6	74.6	9.6	8.1	6.3
			Complex with SecB	70.6	57.7	46.1	2.9	2.4	1.9
			Complex with TF (Tig)	77.6	65.6	50.6	3.7	3.2	2.4
P67127	YgdQ	Cell inner membrane	Monomer	118.3	100.0	77.2	10.4	8.8	6.8
			Complex with SecB	71.3	58.2	46.5	3.0	2.4	2.0
			Complex with TF (Tig)	78.6	66.4	51.3	3.9	3.3	2.5
P65294	YgdR	Cell membrane	Monomer	189.4	160.0	123.6	28.3	23.9	18.4
			Complex with SecB	77.5	63.3	50.6	3.7	3.0	2.4
			Complex with TF (Tig)	87.8	74.1	57.3	5.1	4.3	3.3
P03813	YgeA		Monomer	120.0	101.4	78.3	10.7	9.0	7.0
Q46786	YgeF		Monomer	140.5	118.7	91.7	15.2	12.8	9.9
Q46787	YgeG		Monomer	133.9	113.1	87.4	13.7	11.5	8.9
P76639	YgeH		Monomer	89.9	75.9	58.7	5.4	4.6	3.5
Q46789	YgeI		Monomer	187.8	158.6	122.5	27.8	23.5	18.1
Q46791	YgeK		Monomer	123.6	104.4	80.6	11.4	9.7	7.5
Q46793	YgeN		Monomer	116.3	98.2	75.9	10.0	8.4	6.5
Q46795	YgeO		Monomer	145.1	122.6	94.7	16.3	13.7	10.6
Q46796	YgeP		Monomer	164.5	139.0	107.3	21.2	17.9	13.8
Q46797	YgeQ		Monomer	110.3	93.2	72.0	8.8	7.5	5.8
Q46798	YgeR	Cell membrane	Monomer	117.6	99.4	76.8	10.2	8.6	6.7
			Complex with SecB	71.2	58.2	46.4	3.0	2.4	1.9
			Complex with TF (Tig)	78.4	66.2	51.2	3.8	3.2	2.5
Q46802	YgeV		Monomer	82.3	69.5	53.7	4.3	3.7	2.8
Q46803	YgeW		Monomer	96.4	81.4	62.9	6.4	5.4	4.2
P66899	YgeX		Monomer	97.1	82.0	63.4	6.5	5.5	4.3
P65807	YgeY		Monomer	95.8	81.0	62.5	6.3	5.3	4.1
P0AC28	YgfA		Monomer	128.7	108.8	84.0	12.5	10.6	8.2

UniProtKB A. N.	Protein name	Subcellular location	Subunit	D_0 $\left(\frac{\mu m^2}{s}\right)$ 310 K	303 K	293 K	D_{cyto} $\left(\frac{\mu m^2}{s}\right)$ 310 K	303 K	293 K
P0A8C4	YgfB		Monomer	128.4	108.5	83.8	12.5	10.5	8.1
P52037	YgfF		Monomer	118.7	100.3	77.5	10.5	8.8	6.8
P52043	YgfH		Monomer	89.2	75.3	58.2	5.3	4.5	3.5
P52044	YgfI		Monomer	106.0	89.5	69.1	8.0	6.8	5.2
Q46810	YgfJ		Monomer	127.8	107.9	83.4	12.3	10.4	8.0
Q46811	YgfK		Monomer	66.1	55.8	43.1	2.4	2.1	1.6
P64557	YgfM		Monomer	114.2	96.5	74.5	9.6	8.1	6.2
Q46817	YgfQ	Cell membrane	Monomer	94.2	79.6	61.5	6.1	5.1	4.0
			Complex with SecB	66.0	53.9	43.1	2.4	2.0	1.6
			Complex with TF (Tig)	71.4	60.3	46.6	3.0	2.5	2.0
Q46819	YgfS		Monomer	138.5	117.0	90.4	14.7	12.4	9.6
Q46820	YgfT		Monomer	80.9	68.3	52.8	4.2	3.5	2.7
Q46821	YgfU	Cell inner membrane	Monomer	90.6	76.5	59.1	5.5	4.7	3.6
			Complex with SecB	64.9	53.1	42.4	2.3	1.9	1.5
			Complex with TF (Tig)	70.0	59.1	45.7	2.8	2.4	1.9
Q46824	YgfX	Membrane	Monomer	143.3	121.0	93.5	15.8	13.4	10.3
			Complex with SecB	74.5	60.9	48.6	3.4	2.7	2.2
			Complex with TF (Tig)	83.2	70.3	54.3	4.5	3.8	2.9
P64559	YgfY		Monomer	169.0	142.7	110.3	22.4	18.9	14.6
P0ADE8	YgfZ	Cytoplasm	Monomer	104.3	88.1	68.1	7.7	6.5	5.1
P11664	YggC		Monomer	116.7	98.6	76.1	10.0	8.5	6.6
P11663	YggD		Monomer	133.4	112.7	87.0	13.6	11.5	8.8
P0ADS6	YggE		Monomer	117.5	99.3	76.7	10.2	8.6	6.7
P21437	YggF		Monomer	106.4	89.9	69.4	8.1	6.9	5.3
			Homodimer	81.1	66.2	52.9	4.2	3.4	2.7
P25894	YggG		Monomer	117.1	99.0	76.4	10.1	8.6	6.6
P38521	YggL		Monomer	156.2	132.0	101.9	19.0	16.1	12.4
P46142	YggM		Monomer	101.7	86.0	66.4	7.3	6.2	4.8
P0ADS9	YggN		Monomer	117.9	99.6	76.9	10.3	8.7	6.7
P52048	YggP		Monomer	95.0	80.3	62.0	6.2	5.2	4.0
P52052	YggR		Monomer	104.4	88.2	68.1	7.8	6.6	5.1

UniProtKB A. N.	Protein name	Subcellular location	Subunit	D_0 $\left(\frac{\mu m^2}{s}\right)$			D_{cyto} $\left(\frac{\mu m^2}{s}\right)$		
				310 K	303 K	293 K	310 K	303 K	293 K
P67080	YggS		Monomer	119.0	100.5	77.7	10.5	8.9	6.9
P64564	YggT	Cell membrane	Monomer	128.6	108.6	83.9	12.5	10.6	8.2
			Complex with SecB	72.8	59.5	47.5	3.2	2.6	2.1
			Complex with TF (Tig)	80.7	68.2	52.7	4.1	3.5	2.7
P52060	YggU		Monomer	169.5	143.2	110.6	22.6	19.1	14.7
P52062	YggW		Monomer	97.8	82.6	63.8	6.6	5.6	4.3
P0A8P3	YggX		Monomer	166.5	140.6	108.6	21.7	18.4	14.2
P0AG84	YghA		Monomer	110.0	93.0	71.8	8.8	7.4	5.7
P0AA60	YghB	Cell inner membrane	Monomer	122.1	103.2	79.7	11.1	9.4	7.3
			Complex with SecB	71.9	58.7	46.9	3.1	2.5	2.0
			Complex with TF (Tig)	79.4	67.1	51.8	4.0	3.4	2.6
Q46832	YghD	Cell inner membrane	Monomer	130.5	110.2	85.1	12.9	10.9	8.4
			Complex with SecB	73.1	59.7	47.7	3.2	2.6	2.1
			Complex with TF (Tig)	81.1	68.5	52.9	4.2	3.5	2.7
Q46833	YghE	Cell inner membrane	Monomer	109.3	92.3	71.3	8.6	7.3	5.6
			Complex with SecB	69.6	56.9	45.4	2.8	2.3	1.8
			Complex with TF (Tig)	76.3	64.4	49.8	3.6	3.0	2.3
Q46834	YghF	Periplasm	Monomer	109.2	92.2	71.3	8.6	7.3	5.6
			Complex with SecB	69.6	56.9	45.4	2.8	2.3	1.8
			Complex with TF (Tig)	76.2	64.4	49.7	3.6	3.0	2.3
Q46835	YghG	Cell membrane	Monomer	148.5	125.4	96.9	17.1	14.4	11.1
			Complex with SecB	75.0	61.3	48.9	3.4	2.8	2.2
			Complex with TF (Tig)	83.9	70.9	54.8	4.6	3.9	3.0
P0CK95	YghJ	Cell membrane	Monomer	57.2	48.3	37.3	1.6	1.4	1.1
			Complex with SecB	49.9	40.8	32.6	1.1	0.9	0.7
			Complex with TF (Tig)	51.8	43.7	33.8	1.2	1.0	0.8
Q46840	YghO		Monomer	98.5	83.2	64.3	6.8	5.7	4.4
Q46841	YghQ	Cell inner membrane	Monomer	101.7	85.9	66.3	7.3	6.2	4.8
			Complex with SecB	68.0	55.5	44.4	2.6	2.2	1.7
			Complex with TF (Tig)	74.0	62.5	48.3	3.3	2.8	2.2
P64572	YghR		Monomer	115.0	97.1	75.0	9.7	8.2	6.3

UniProtKB A. N.	Protein name	Subcellular location	Subunit	D_0 $\left(\frac{\mu m^2}{s}\right)$			D_{cyto} $\left(\frac{\mu m^2}{s}\right)$		
				310 K	303 K	293 K	310 K	303 K	293 K
Q46843	YghS		Monomer	118.0	99.7	77.0	10.3	8.7	6.7
Q46844	YghT		Monomer	119.8	101.2	78.2	10.7	9.0	7.0
Q46845	YghU		Monomer	108.8	91.9	71.0	8.6	7.2	5.6
			Homodimer	82.9	67.8	54.1	4.4	3.6	2.9
P64574	YghW		Monomer	166.5	140.7	108.7	21.7	18.4	14.2
Q7DFU6	YghX		Monomer	111.7	94.4	72.9	9.1	7.7	5.9
Q46851	YghZ		Monomer	101.4	85.6	66.1	7.2	6.1	4.7
Q79CP2	YgiA		Monomer	173.1	146.2	112.9	23.5	19.9	15.4
P0ADT2	YgiB		Monomer	123.5	104.3	80.6	11.4	9.6	7.4
P0ADT5	YgiC		Monomer	95.6	80.8	62.4	6.3	5.3	4.1
P24197	YgiD		Monomer	113.8	96.2	74.3	9.5	8.0	6.2
P30871	YgiF		Monomer	93.0	78.6	60.7	5.9	5.0	3.8
P39834	YgiL	Fimbrium	Monomer	132.8	112.2	86.7	13.4	11.3	8.8
			Complex with SecB	73.4	59.9	47.9	3.2	2.6	2.1
			Complex with TF (Tig)	81.5	68.9	53.2	4.2	3.6	2.8
P0ADT8	YgiM	Membrane	Monomer	124.3	105.0	81.1	11.6	9.8	7.6
			Complex with SecB	72.2	59.0	47.1	3.1	2.5	2.0
			Complex with TF (Tig)	79.9	67.5	52.1	4.0	3.4	2.6
P0ADU2	YgiN		Monomer	163.1	137.8	106.5	20.8	17.6	13.6
			Homodimer	124.3	101.6	81.1	11.6	9.5	7.6
Q46861	YgiQ		Monomer	75.1	63.4	49.0	3.4	2.9	2.2
Q46863	YgiS	Periplasm	Monomer	85.1	71.9	55.5	4.7	4.0	3.1
			Complex with SecB	63.2	51.6	41.2	2.2	1.8	1.4
			Complex with TF (Tig)	67.7	57.2	44.2	2.6	2.2	1.7
Q46866	YgiV		Monomer	137.5	116.2	89.7	14.5	12.2	9.5
P0ADU5	YgiW	Periplasm	Monomer	151.2	127.7	98.6	17.8	15.0	11.6
			Complex with SecB	75.2	61.4	49.1	3.4	2.8	2.2
			Complex with TF (Tig)	84.3	71.2	55.0	4.6	3.9	3.0
Q46867	YgiZ	Cell inner membrane	Monomer	154.7	130.7	101.0	18.7	15.8	12.2
			Complex with SecB	75.5	61.7	49.3	3.5	2.8	2.3
			Complex with TF (Tig)	84.7	71.6	55.3	4.7	3.9	3.0

UniProtKB A. N.	Protein name	Subcellular location	Subunit	D_0 $\left(\frac{\mu m^2}{s}\right)$			D_{cyto} $\left(\frac{\mu m^2}{s}\right)$		
				310 K	303 K	293 K	310 K	303 K	293 K
P42589	YgjH		Monomer	159.0	134.3	103.8	19.8	16.7	12.9
			Homodimer	121.2	99.0	79.1	10.9	8.9	7.1
P42590	YgjI	Cell inner membrane	Monomer	90.3	76.3	58.9	5.5	4.6	3.6
			Complex with SecB	64.9	53.0	42.3	2.3	1.9	1.5
			Complex with TF (Tig)	69.9	59.0	45.6	2.8	2.4	1.9
P42591	YgjJ		Monomer	100.1	84.6	65.3	7.0	5.9	4.6
P42592	YgjK		Monomer	73.4	62.0	47.9	3.2	2.7	2.1
P42597	YgjP		Monomer	132.8	112.2	86.7	13.4	11.3	8.8
P42598	YgjQ	Membrane	Monomer	119.6	101.0	78.0	10.6	9.0	6.9
			Complex with SecB	71.5	58.4	46.7	3.0	2.5	2.0
			Complex with TF (Tig)	78.9	66.6	51.5	3.9	3.3	2.5
P42599	YgjR	Membrane	Monomer	104.2	88.0	68.0	7.7	6.5	5.0
			Complex with SecB	68.5	56.0	44.7	2.7	2.2	1.8
			Complex with TF (Tig)	74.8	63.2	48.8	3.4	2.9	2.2
P42603	YgjV	Cell inner membrane	Monomer	130.2	110.0	84.9	12.8	10.9	8.4
			Complex with SecB	73.0	59.7	47.7	3.2	2.6	2.1
			Complex with TF (Tig)	81.0	68.5	52.9	4.2	3.5	2.7
P11865	YhaB		Monomer	130.0	109.8	84.8	12.8	10.8	8.4
P11864	YhaC		Monomer	95.5	80.6	62.3	6.3	5.3	4.1
P64590	YhaH	Cell inner membrane	Monomer	150.0	126.7	97.9	17.5	14.8	11.4
			Complex with SecB	75.1	61.4	49.0	3.4	2.8	2.2
			Complex with TF (Tig)	84.1	71.1	54.9	4.6	3.9	3.0
P64592	YhaI	Cell inner membrane	Monomer	153.6	129.7	100.2	18.4	15.5	12.0
			Complex with SecB	75.4	61.6	49.2	3.5	2.8	2.3
			Complex with TF (Tig)	84.6	71.4	55.2	4.6	3.9	3.0
P67660	YhaJ		Monomer	107.7	91.0	70.3	8.4	7.1	5.5
P42624	YhaK	Cytoplasm	Monomer	118.8	100.4	77.5	10.5	8.8	6.8
P42625	YhaL		Monomer	211.3	178.5	137.9	35.0	29.6	22.9
P42626	YhaM		Monomer	95.4	80.6	62.2	6.3	5.3	4.1
P42628	YhaO	Cell inner membrane	Monomer	93.0	78.6	60.7	5.9	5.0	3.8
			Complex with SecB	65.7	53.7	42.9	2.4	2.0	1.6

UniProtKB A. N.	Protein name	Subcellular location	Subunit	D_0 $\left(\frac{\mu m^2}{s}\right)$			D_{cyto} $\left(\frac{\mu m^2}{s}\right)$		
				310 K	303 K	293 K	310 K	303 K	293 K
			Complex with TF (Tig)	70.9	59.9	46.3	3.0	2.5	1.9
P64594	YhaV		Monomer	137.5	116.2	89.7	14.5	12.2	9.5
			Homohexamer	68.1	55.7	44.5	2.7	2.2	1.7
P0AA73	YhbE	Cell inner membrane	Monomer	105.6	89.2	68.9	8.0	6.7	5.2
			Complex with SecB	68.9	56.3	44.9	2.7	2.2	1.8
			Complex with TF (Tig)	75.2	63.5	49.1	3.4	2.9	2.2
P0AFX0	YhbH		Monomer	167.7	141.7	109.4	22.1	18.6	14.4
P0A894	YhbJ		Monomer	108.7	91.8	70.9	8.5	7.2	5.6
P45470	YhbO		Monomer	134.5	113.7	87.8	13.8	11.7	9.0
			Homodimer	102.5	83.8	66.9	7.4	6.1	4.9
P67762	YhbP		Monomer	140.8	119.0	91.9	15.3	12.9	10.0
P45472	YhbQ		Monomer	164.6	139.1	107.4	21.2	17.9	13.9
P63417	YhbS		Monomer	135.5	114.4	88.4	14.0	11.8	9.1
P64599	YhbT		Monomer	132.3	111.8	86.3	13.3	11.3	8.7
P45527	YhbU		Monomer	103.2	87.2	67.4	7.6	6.4	4.9
P45475	YhbV		Monomer	108.6	91.8	70.9	8.5	7.2	5.6
P0ADV5	YhbW		Monomer	103.2	87.1	67.3	7.5	6.4	4.9
P42640	YhbX	Cell inner membrane	Monomer	85.0	71.9	55.5	4.7	4.0	3.1
			Complex with SecB	63.1	51.6	41.2	2.2	1.8	1.4
			Complex with TF (Tig)	67.6	57.1	44.1	2.6	2.2	1.7
P0AGK4	YhbY	Cytoplasm	Monomer	167.5	141.5	109.3	22.0	18.6	14.4
P28722	YhcA	Periplasm	Monomer	119.8	101.2	78.2	10.7	9.0	7.0
			Complex with SecB	71.5	58.4	46.7	3.0	2.5	2.0
			Complex with TF (Tig)	78.9	66.7	51.5	3.9	3.3	2.5
P0ADW3	YhcB	Cell inner membrane	Monomer	147.3	124.5	96.1	16.8	14.2	11.0
			Complex with SecB	74.9	61.2	48.9	3.4	2.8	2.2
			Complex with TF (Tig)	83.8	70.8	54.7	4.5	3.8	3.0
P0ADW6	YhcC		Monomer	106.0	89.6	69.2	8.1	6.8	5.3
P45420	YhcD	Cell outer membrane	Monomer	74.1	62.6	48.4	3.3	2.8	2.2
			Complex with SecB	58.9	48.1	38.4	1.8	1.5	1.2
			Complex with TF (Tig)	62.3	52.6	40.6	2.1	1.8	1.4

UniProtKB A. N.	Protein name	Subcellular location	Subunit	D_0 $\left(\frac{\mu m^2}{s}\right)$			D_{cyto} $\left(\frac{\mu m^2}{s}\right)$		
				310 K	303 K	293 K	310 K	303 K	293 K
P45421	YhcE		Monomer	133.6	112.9	87.2	13.6	11.5	8.9
P45422	YhcF		Monomer	120.6	101.9	78.7	10.8	9.2	7.1
P45423	YhcG		Monomer	97.1	82.1	63.4	6.5	5.5	4.3
P45424	YhcH		Monomer	140.0	118.3	91.4	15.1	12.7	9.8
P64612	YhcM		Monomer	97.3	82.2	63.5	6.6	5.5	4.3
P64614	YhcN	Periplasm	Monomer	178.3	150.6	116.3	25.0	21.1	16.3
			Complex with SecB	77.0	62.9	50.2	3.7	3.0	2.4
			Complex with TF (Tig)	87.0	73.5	56.8	5.0	4.2	3.3
P64616	YhcO		Monomer	167.4	141.4	109.2	22.0	18.6	14.3
P25536	YhdE	Cytoplasm	Monomer	127.8	107.9	83.4	12.3	10.4	8.0
P26646	YhdH	Cytoplasm	Monomer	105.9	89.5	69.1	8.0	6.8	5.2
			Homodimer	80.7	65.9	52.7	4.1	3.4	2.7
P28638	YhdJ		Monomer	107.5	90.8	70.2	8.3	7.0	5.4
P36675	YhdL		Monomer	186.7	157.8	121.9	27.5	23.2	17.9
P36677	YhdN		Monomer	151.4	127.9	98.8	17.8	15.1	11.6
P46474	YhdP		Monomer	61.5	51.9	40.1	2.0	1.7	1.3
P45566	YhdT	Cell membrane	Monomer	179.0	151.2	116.8	25.2	21.3	16.5
			Complex with SecB	77.0	62.9	50.3	3.7	3.0	2.4
			Complex with TF (Tig)	87.0	73.5	56.8	5.0	4.2	3.3
P64619	YhdU	Membrane	Monomer	196.2	165.7	128.0	30.3	25.6	19.8
			Complex with SecB	77.8	63.5	50.8	3.8	3.1	2.5
			Complex with TF (Tig)	88.2	74.5	57.5	5.2	4.4	3.4
P64622	YhdV	Cell membrane	Monomer	193.6	163.6	126.3	29.5	24.9	19.3
			Complex with SecB	77.7	63.5	50.7	3.7	3.1	2.4
			Complex with TF (Tig)	88.0	74.4	57.4	5.1	4.3	3.4
P45766	YhdW	Periplasm	Monomer	103.3	87.2	67.4	7.6	6.4	4.9
			Complex with SecB	68.3	55.8	44.6	2.7	2.2	1.7
			Complex with TF (Tig)	74.5	62.9	48.6	3.4	2.8	2.2
P45767	YhdX	Cell inner membrane	Monomer	97.2	82.1	63.4	6.5	5.5	4.3
			Complex with SecB	66.8	54.6	43.6	2.5	2.1	1.6
			Complex with TF (Tig)	72.5	61.2	47.3	3.1	2.6	2.0

UniProtKB A. N.	Protein name	Subcellular location	Subunit	D_0 $\left(\frac{\mu m^2}{s}\right)$			D_{cyto} $\left(\frac{\mu m^2}{s}\right)$		
				310 K	303 K	293 K	310 K	303 K	293 K
			Monomer	98.8	83.4	64.5	6.8	5.7	4.4
P45768	YhdY	Cell inner membrane	Complex with SecB	67.3	54.9	43.9	2.6	2.1	1.7
			Complex with TF (Tig)	73.0	61.7	47.6	3.2	2.7	2.1
			Monomer	114.3	96.6	74.6	9.6	8.1	6.3
P45769	YhdZ	Cell inner membrane	Complex with SecB	70.6	57.7	46.1	2.9	2.4	1.9
			Complex with TF (Tig)	77.6	65.6	50.6	3.7	3.2	2.4
P64624	YheO		Monomer	117.2	99.0	76.5	10.1	8.6	6.6
P63389	YheS		Monomer	79.6	67.3	52.0	4.0	3.4	2.6
P45524	YheT		Monomer	101.7	85.9	66.4	7.3	6.2	4.8
P67624	YheU		Monomer	184.1	155.5	120.1	26.7	22.5	17.4
P0ADW8	YheV		Monomer	192.1	162.3	125.4	29.1	24.5	19.0
P0ADX1	YhfA		Monomer	149.1	125.9	97.3	17.2	14.6	11.2
P0ADX5	YhfG		Monomer	203.0	171.5	132.5	32.4	27.4	21.2
			Monomer	76.4	64.5	49.8	3.6	3.0	2.3
P45537	YhfK	Cell membrane	Complex with SecB	59.8	48.9	39.0	1.9	1.5	1.2
			Complex with TF (Tig)	63.5	53.6	41.4	2.2	1.9	1.4
			Monomer	213.3	180.2	139.2	35.7	30.1	23.3
P64627	YhfL	Membrane	Complex with SecB	78.3	64.0	51.1	3.8	3.1	2.5
			Complex with TF (Tig)	89.1	75.2	58.1	5.3	4.5	3.5
P45545	YhfS		Monomer	101.6	85.8	66.3	7.3	6.1	4.7
			Monomer	94.4	79.8	61.6	6.1	5.2	4.0
P45546	YhfT	Cell membrane	Complex with SecB	66.1	54.0	43.1	2.4	2.0	1.6
			Complex with TF (Tig)	71.5	60.4	46.6	3.0	2.5	2.0
P64631	YhfU		Monomer	158.2	133.6	103.2	19.5	16.5	12.7
P45549	YhfW		Monomer	96.0	81.1	62.7	6.4	5.4	4.2
P45550	YhfX		Monomer	98.0	82.8	64.0	6.7	5.6	4.4
P45551	YhfY		Monomer	153.2	129.5	100.0	18.3	15.4	11.9
P45552	YhfZ		Monomer	107.4	90.7	70.1	8.3	7.0	5.4
P31667	YhgA		Monomer	107.7	91.0	70.3	8.3	7.1	5.4
			Monomer	83.0	70.1	54.2	4.4	3.7	2.9
P45804	YhgE	Cell membrane	Complex with SecB	62.4	51.0	40.7	2.1	1.7	1.4

UniProtKB A. N.	Protein name	Subcellular location	Subunit	D_0 $\left(\frac{\mu m^2}{s}\right)$			D_{cyto} $\left(\frac{\mu m^2}{s}\right)$		
				310 K	303 K	293 K	310 K	303 K	293 K
			Complex with TF (Tig)	66.7	56.4	43.5	2.5	2.1	1.6
P46837	YhgF		Monomer	74.5	63.0	48.6	3.4	2.8	2.2
P67143	YhgN	Cell inner membrane	Monomer	127.8	108.0	83.4	12.3	10.4	8.0
			Complex with SecB	72.7	59.4	47.5	3.2	2.6	2.1
			Complex with TF (Tig)	80.6	68.1	52.6	4.1	3.5	2.7
P0ADX7	YhhA		Monomer	141.4	119.4	92.2	15.4	13.0	10.0
P28911	YhhH		Monomer	149.0	125.9	97.2	17.2	14.6	11.2
P28912	YhhI		Monomer	97.3	82.2	63.5	6.6	5.5	4.3
P0AGH1	YhhJ	Cell inner membrane	Monomer	99.2	83.8	64.7	6.9	5.8	4.5
			Complex with SecB	67.4	55.0	44.0	2.6	2.1	1.7
			Complex with TF (Tig)	73.1	61.8	47.7	3.2	2.7	2.1
P37613	YhhK		Monomer	149.1	126.0	97.3	17.2	14.6	11.3
P37614	YhhL		Monomer	170.8	144.3	111.5	22.9	19.4	15.0
P37615	YhhM		Monomer	153.4	129.6	100.1	18.3	15.5	12.0
P0ADI9	YhhN	Cell membrane	Monomer	122.8	103.8	80.1	11.3	9.5	7.4
			Complex with SecB	72.0	58.8	47.0	3.1	2.5	2.0
			Complex with TF (Tig)	79.6	67.2	51.9	4.0	3.4	2.6
P37619	YhhQ	Cell inner membrane	Monomer	120.0	101.3	78.3	10.7	9.0	7.0
			Complex with SecB	71.6	58.5	46.7	3.0	2.5	2.0
			Complex with TF (Tig)	78.9	66.7	51.5	3.9	3.3	2.5
P37621	YhhS	Cell inner membrane	Monomer	98.1	82.9	64.0	6.7	5.7	4.4
			Complex with SecB	67.1	54.8	43.8	2.5	2.1	1.7
			Complex with TF (Tig)	72.8	61.5	47.5	3.2	2.7	2.1
P0AGM0	YhhT	Cell inner membrane	Monomer	101.7	85.9	66.4	7.3	6.2	4.8
			Complex with SecB	68.0	55.5	44.4	2.6	2.2	1.7
			Complex with TF (Tig)	74.0	62.5	48.3	3.3	2.8	2.2
P46852	YhhW		Monomer	118.1	99.8	77.1	10.3	8.7	6.7
P46853	YhhX		Monomer	101.4	85.7	66.2	7.2	6.1	4.7
P46854	YhhY		Monomer	134.7	113.8	87.9	13.9	11.7	9.0
P46855	YhhZ		Monomer	96.3	81.4	62.9	6.4	5.4	4.2
P0AFV2	YhiD	Cell inner membrane	Monomer	124.0	104.8	80.9	11.5	9.7	7.5

UniProtKB A. N.	Protein name	Subcellular location	Subunit	D_0 $\left(\frac{\mu m^2}{s}\right)$			D_{cyto} $\left(\frac{\mu m^2}{s}\right)$		
				310 K	303 K	293 K	310 K	303 K	293 K
			Complex with SecB	72.2	59.0	47.1	3.1	2.5	2.0
			Complex with TF (Tig)	79.8	67.4	52.1	4.0	3.4	2.6
P37624	YhiH	Cell inner membrane	Monomer	69.7	58.9	45.5	2.8	2.4	1.8
			Complex with SecB	56.8	46.4	37.1	1.6	1.3	1.1
			Complex with TF (Tig)	59.8	50.6	39.0	1.9	1.6	1.2
P37626	YhiI		Monomer	101.4	85.6	66.1	7.2	6.1	4.7
P37627	YhiJ		Monomer	84.8	71.7	55.4	4.7	4.0	3.1
P37629	YhiL		Monomer	84.6	71.5	55.2	4.7	3.9	3.0
P37630	YhiM	Cell inner membrane	Monomer	102.6	86.7	66.9	7.4	6.3	4.9
			Complex with SecB	68.2	55.7	44.5	2.7	2.2	1.7
			Complex with TF (Tig)	74.3	62.7	48.5	3.3	2.8	2.2
P37631	YhiN		Monomer	96.7	81.7	63.1	6.5	5.5	4.2
P68567	YhiQ		Monomer	117.0	98.8	76.3	10.1	8.5	6.6
P37634	YhiR		Monomer	109.4	92.4	71.4	8.7	7.3	5.7
P37635	YhiS		Monomer	113.0	95.4	73.7	9.3	7.9	6.1
P37197	YhjA		Monomer	90.7	76.6	59.2	5.5	4.7	3.6
P37640	YhjB		Monomer	125.3	105.9	81.8	11.8	10.0	7.7
P37641	YhjC		Monomer	107.6	90.9	70.2	8.3	7.0	5.4
P37642	YhjD	Cell inner membrane	Monomer	102.3	86.4	66.8	7.4	6.3	4.8
			Complex with SecB	68.1	55.7	44.5	2.7	2.2	1.7
			Complex with TF (Tig)	74.2	62.7	48.4	3.3	2.8	2.2
P37643	YhjE	Cell inner membrane	Monomer	93.9	79.3	61.3	6.0	5.1	3.9
			Complex with SecB	65.9	53.9	43.0	2.4	2.0	1.6
			Complex with TF (Tig)	71.3	60.2	46.5	3.0	2.5	2.0
P37645	YhjG	Cell membrane	Monomer	78.5	66.3	51.2	3.9	3.3	2.5
			Complex with SecB	60.7	49.6	39.6	1.9	1.6	1.3
			Complex with TF (Tig)	64.6	54.6	42.1	2.3	1.9	1.5
P37646	YhjH		Monomer	112.7	95.2	73.6	9.3	7.8	6.1
P37648	YhjJ	Periplasm	Monomer	88.1	74.4	57.5	5.1	4.4	3.4
			Complex with SecB	64.2	52.4	41.9	2.3	1.8	1.5
			Complex with TF (Tig)	69.0	58.3	45.0	2.7	2.3	1.8

UniProtKB A. N.	Protein name	Subcellular location	Subunit	D_0 $\left(\frac{\mu m^2}{s}\right)$			D_{cyto} $\left(\frac{\mu m^2}{s}\right)$		
				310 K	303 K	293 K	310 K	303 K	293 K
P37649	YhjK	Cell membrane	Monomer	78.6	66.4	51.3	3.9	3.3	2.5
			Complex with SecB	60.7	49.6	39.6	1.9	1.6	1.3
			Complex with TF (Tig)	64.6	54.6	42.1	2.3	1.9	1.5
P37655	YhjQ		Monomer	115.4	97.5	75.3	9.8	8.3	6.4
P0ADJ3	YhjR		Monomer	198.2	167.4	129.3	30.9	26.1	20.2
P37657	YhjS		Monomer	85.8	72.5	56.0	4.8	4.1	3.1
P0ADJ5	YhjT		Monomer	194.5	164.3	126.9	29.8	25.2	19.4
P37659	YhjU		Monomer	84.4	71.3	55.0	4.6	3.9	3.0
P37660	YhjV	Cell inner membrane	Monomer	93.9	79.3	61.2	6.0	5.1	3.9
			Complex with SecB	65.9	53.9	43.0	2.4	2.0	1.6
			Complex with TF (Tig)	71.2	60.2	46.5	3.0	2.5	2.0
P37662	YhjX	Cell inner membrane	Monomer	97.4	82.3	63.6	6.6	5.6	4.3
			Complex with SecB	66.9	54.7	43.7	2.5	2.1	1.6
			Complex with TF (Tig)	72.5	61.3	47.3	3.1	2.6	2.0
P37663	YhjY		Monomer	118.7	100.3	77.5	10.4	8.8	6.8
P0ADJ8	YiaA	Cell inner membrane	Monomer	143.1	120.9	93.4	15.8	13.3	10.3
			Complex with SecB	74.5	60.8	48.6	3.4	2.7	2.2
			Complex with TF (Tig)	83.2	70.3	54.3	4.5	3.8	2.9
P11286	YiaB	Cell inner membrane	Monomer	157.8	133.3	103.0	19.4	16.4	12.7
			Complex with SecB	75.7	61.9	49.4	3.5	2.9	2.3
			Complex with TF (Tig)	85.1	71.9	55.5	4.7	4.0	3.1
P37664	YiaC		Monomer	139.8	118.1	91.2	15.0	12.7	9.8
P37665	YiaD	Cell inner membrane	Monomer	126.2	106.6	82.4	12.0	10.1	7.8
			Complex with SecB	72.5	59.2	47.3	3.1	2.6	2.0
			Complex with TF (Tig)	80.3	67.8	52.4	4.1	3.4	2.7
P0ADK0	YiaF		Monomer	119.2	100.7	77.8	10.6	8.9	6.9
P0A9V5	YiaG		Monomer	166.0	140.2	108.3	21.6	18.3	14.1
P37669	YiaH	Cell inner membrane	Monomer	102.7	86.7	67.0	7.5	6.3	4.9
			Complex with SecB	68.2	55.7	44.5	2.7	2.2	1.7
			Complex with TF (Tig)	74.3	62.8	48.5	3.3	2.8	2.2
P37671	YiaJ		Monomer	110.6	93.5	72.2	8.9	7.5	5.8

UniProtKB A. N.	Protein name	Subcellular location	Subunit	D_0 $\left(\frac{\mu m^2}{s}\right)$			D_{cyto} $\left(\frac{\mu m^2}{s}\right)$		
				310 K	303 K	293 K	310 K	303 K	293 K
P37673	YiaL		Monomer	138.4	116.9	90.3	14.7	12.4	9.6
			Monomer	138.5	117.0	90.4	14.7	12.4	9.6
P37674	YiaM	Cell inner membrane	Complex with SecB	74.0	60.5	48.3	3.3	2.7	2.2
			Complex with TF (Tig)	82.5	69.7	53.8	4.4	3.7	2.8
			Monomer	95.4	80.6	62.2	6.3	5.3	4.1
P37675	YiaN	Cell inner membrane	Complex with SecB	66.3	54.2	43.3	2.5	2.0	1.6
			Complex with TF (Tig)	71.8	60.7	46.9	3.1	2.6	2.0
			Monomer	104.4	88.2	68.2	7.8	6.6	5.1
P37676	YiaO	Periplasm	Complex with SecB	68.6	56.1	44.8	2.7	2.2	1.8
			Complex with TF (Tig)	74.8	63.2	48.8	3.4	2.9	2.2
			Monomer	116.2	98.2	75.8	9.9	8.4	6.5
P37681	YiaT	Cell outer membrane	Complex with SecB	70.9	57.9	46.3	3.0	2.4	1.9
			Complex with TF (Tig)	78.1	65.9	50.9	3.8	3.2	2.5
P37682	YiaU		Monomer	103.0	87.0	67.2	7.5	6.4	4.9
			Monomer	98.5	83.2	64.3	6.8	5.7	4.4
P37683	YiaV	Cell inner membrane	Complex with SecB	67.2	54.9	43.8	2.6	2.1	1.7
			Complex with TF (Tig)	72.9	61.6	47.6	3.2	2.7	2.1
			Monomer	158.5	133.9	103.4	19.6	16.6	12.8
P0ADK4	YiaW	Cell inner membrane	Complex with SecB	75.8	61.9	49.5	3.5	2.9	2.3
			Complex with TF (Tig)	85.1	71.9	55.6	4.7	4.0	3.1
P37686	YiaY		Monomer	99.8	84.4	65.2	7.0	5.9	4.6
P0ADK6	YibA		Monomer	109.5	92.5	71.5	8.7	7.3	5.7
P11290	YibD		Monomer	99.7	84.2	65.0	7.0	5.9	4.5
P0ACA1	YibF		Monomer	125.4	106.0	81.9	11.8	10.0	7.7
P32106	YibG		Monomer	136.8	115.6	89.3	14.3	12.1	9.3
			Monomer	98.3	83.0	64.1	6.7	5.7	4.4
P0AFV0	YibH	Cell inner membrane	Complex with SecB	67.1	54.8	43.8	2.6	2.1	1.7
			Complex with TF (Tig)	72.8	61.5	47.5	3.2	2.7	2.1
P32108	YibI		Monomer	151.8	128.2	99.0	17.9	15.1	11.7
P32109	YibJ		Monomer	117.8	99.5	76.9	10.3	8.7	6.7
P0ADK8	YibL	Cytoplasm	Monomer	152.5	128.8	99.5	18.1	15.3	11.8

UniProtKB A. N.	Protein name	Subcellular location	Subunit	D_0 $\left(\frac{\mu m^2}{s}\right)$			D_{cyto} $\left(\frac{\mu m^2}{s}\right)$		
				310 K	303 K	293 K	310 K	303 K	293 K
P0AG27	YibN		Monomer	144.9	122.4	94.6	16.2	13.7	10.6
P37691	YibQ		Monomer	105.2	88.8	68.6	7.9	6.7	5.2
Q2M7R5	YibT		Monomer	188.3	159.1	122.9	27.9	23.6	18.2
A5A625	YibV		Monomer	158.9	134.2	103.7	19.7	16.7	12.9
P23839	YicC		Monomer	107.8	91.1	70.4	8.4	7.1	5.5
P0AGM2	YicG	Cell inner membrane	Monomer	126.5	106.9	82.6	12.1	10.2	7.9
			Complex with SecB	72.5	59.3	47.3	3.1	2.6	2.0
			Complex with TF (Tig)	80.3	67.9	52.4	4.1	3.5	2.7
P31433	YicH		Monomer	84.2	71.2	55.0	4.6	3.9	3.0
P31434	YicI		Monomer	73.5	62.1	48.0	3.2	2.7	2.1
			Homohexamer	36.4	29.8	23.8	0.4	0.3	0.3
P31435	YicJ	Cell inner membrane	Monomer	91.1	77.0	59.5	5.6	4.7	3.7
			Complex with SecB	65.1	53.2	42.5	2.3	1.9	1.5
			Complex with TF (Tig)	70.2	59.3	45.8	2.9	2.4	1.9
P31437	YicL	Cell inner membrane	Monomer	107.9	91.1	70.4	8.4	7.1	5.5
			Complex with SecB	69.4	56.7	45.3	2.8	2.3	1.8
			Complex with TF (Tig)	75.9	64.1	49.5	3.5	3.0	2.3
P0ADL3	YicN		Monomer	139.4	117.8	91.0	14.9	12.6	9.7
P31440	YicO	Cell inner membrane	Monomer	94.1	79.5	61.4	6.1	5.1	4.0
			Complex with SecB	66.0	53.9	43.1	2.4	2.0	1.6
			Complex with TF (Tig)	71.3	60.3	46.6	3.0	2.5	2.0
P25531	YicR		Monomer	119.8	101.2	78.2	10.7	9.0	7.0
Q2M7X4	YicS		Monomer	165.8	140.1	108.2	21.5	18.2	14.1
P0A8Y5	YidA		Monomer	112.6	95.1	73.5	9.3	7.8	6.0
P09996	YidB		Monomer	152.1	128.5	99.3	18.0	15.2	11.7
P0A8C8	YidD		Monomer	176.9	149.4	115.4	24.6	20.8	16.1
P60872	YidE	Cell membrane	Monomer	86.1	72.7	56.2	4.9	4.1	3.2
			Complex with SecB	63.5	51.9	41.4	2.2	1.8	1.4
			Complex with TF (Tig)	68.1	57.5	44.4	2.7	2.2	1.7
P31443	YidF		Monomer	133.6	112.9	87.2	13.6	11.5	8.9
P0ADL6	YidG	Cell inner membrane	Monomer	152.2	128.6	99.3	18.0	15.2	11.8

UniProtKB A. N.	Protein name	Subcellular location	Subunit	D_0 $\left(\frac{\mu m^2}{s}\right)$			D_{cyto} $\left(\frac{\mu m^2}{s}\right)$		
				310 K	303 K	293 K	310 K	303 K	293 K
			Complex with SecB	75.3	61.5	49.1	3.5	2.8	2.3
			Complex with TF (Tig)	84.4	71.3	55.1	4.6	3.9	3.0
P0ADM0	YidH	Cell inner membrane	Monomer	156.7	132.4	102.3	19.2	16.2	12.5
			Complex with SecB	75.7	61.8	49.4	3.5	2.9	2.3
			Complex with TF (Tig)	84.9	71.8	55.4	4.7	4.0	3.1
P31446	YidI	Cell inner membrane	Monomer	144.5	122.0	94.3	16.1	13.6	10.5
			Complex with SecB	74.6	61.0	48.7	3.4	2.8	2.2
			Complex with TF (Tig)	83.4	70.4	54.4	4.5	3.8	2.9
P31447	YidJ		Monomer	87.0	73.5	56.8	5.0	4.2	3.3
P31448	YidK	Cell inner membrane	Monomer	84.3	71.2	55.0	4.6	3.9	3.0
			Complex with SecB	62.9	51.4	41.0	2.1	1.7	1.4
			Complex with TF (Tig)	67.3	56.9	43.9	2.6	2.2	1.7
P31449	YidL		Monomer	106.8	90.3	69.7	8.2	6.9	5.3
P31453	YidP		Monomer	116.3	98.3	75.9	10.0	8.4	6.5
P0ADM4	YidQ		Monomer	161.5	136.5	105.4	20.4	17.2	13.3
P31455	YidR		Monomer	94.7	80.0	61.8	6.1	5.2	4.0
P0ADM6	YidX	Membrane	Monomer	122.1	103.1	79.7	11.1	9.4	7.3
			Complex with SecB	71.9	58.7	46.9	3.1	2.5	2.0
			Complex with TF (Tig)	79.4	67.1	51.8	4.0	3.3	2.6
P31463	YidZ		Monomer	103.4	87.4	67.5	7.6	6.4	5.0
P0ADM8	YieE		Monomer	115.8	97.8	75.6	9.9	8.3	6.4
P0AGE6	YieF		Monomer	130.5	110.3	85.2	12.9	10.9	8.4
P31467	YieH		Monomer	121.1	102.3	79.0	10.9	9.2	7.1
P31470	YieK		Monomer	118.1	99.8	77.1	10.3	8.7	6.7
P31471	YieL		Monomer	97.6	82.5	63.7	6.6	5.6	4.3
P31475	YieP		Monomer	118.5	100.1	77.3	10.4	8.8	6.8
P22787	YifB		Monomer	88.3	74.6	57.6	5.2	4.4	3.4
P0ADN2	YifE		Monomer	155.0	131.0	101.2	18.7	15.8	12.2
P27837	YifK	Cell inner membrane	Monomer	91.5	77.3	59.7	5.7	4.8	3.7
			Complex with SecB	65.2	53.3	42.6	2.4	1.9	1.5
			Complex with TF (Tig)	70.3	59.4	45.9	2.9	2.4	1.9

UniProtKB A. N.	Protein name	Subcellular location	Subunit	D_0 $\left(\frac{\mu m^2}{s}\right)$			D_{cyto} $\left(\frac{\mu m^2}{s}\right)$		
				310 K	303 K	293 K	310 K	303 K	293 K
P0ADN6	YifL	Cell membrane	Monomer	196.5	166.0	128.2	30.4	25.7	19.8
			Complex with SecB	77.8	63.5	50.8	3.8	3.1	2.5
			Complex with TF (Tig)	88.2	74.5	57.6	5.2	4.4	3.4
P56259	YifN		Monomer	140.5	118.7	91.7	15.2	12.8	9.9
P23305	YigA		Monomer	117.3	99.1	76.6	10.2	8.6	6.6
P0ADP0	YigB		Monomer	116.7	98.6	76.1	10.0	8.5	6.6
P27840	YigE		Monomer	115.4	97.5	75.3	9.8	8.3	6.4
P27842	YigF		Monomer	148.9	125.8	97.2	17.2	14.5	11.2
P27843	YigG	Cell inner membrane	Monomer	149.5	126.3	97.6	17.3	14.7	11.3
			Complex with SecB	75.1	61.3	49.0	3.4	2.8	2.2
			Complex with TF (Tig)	84.1	71.0	54.9	4.6	3.9	3.0
P0ADP2	YigI		Monomer	139.6	117.9	91.1	15.0	12.6	9.8
P27848	YigL		Monomer	112.6	95.1	73.5	9.3	7.8	6.0
P0ADP5	YigM	Cell membrane	Monomer	107.1	90.5	69.9	8.2	7.0	5.4
			Complex with SecB	69.2	56.5	45.2	2.8	2.3	1.8
			Complex with TF (Tig)	75.6	63.9	49.4	3.5	3.0	2.3
P0ADP7	YigP		Monomer	126.3	106.7	82.4	12.0	10.1	7.8
P27862	YigZ		Monomer	127.2	107.5	83.0	12.2	10.3	8.0
P0ADP9	YihD		Monomer	170.7	144.2	111.4	22.9	19.3	14.9
P32128	YihF		Monomer	90.0	76.0	58.7	5.4	4.6	3.5
P32129	YihG	Cell inner membrane	Monomer	104.1	87.9	67.9	7.7	6.5	5.0
			Complex with SecB	68.5	56.0	44.7	2.7	2.2	1.8
			Complex with TF (Tig)	74.7	63.1	48.8	3.4	2.9	2.2
P0A8H6	YihI		Monomer	134.0	113.2	87.4	13.7	11.6	8.9
			Homodimer	102.1	83.4	66.6	7.4	6.0	4.8
P0ACM9	YihL		Monomer	117.0	98.8	76.3	10.1	8.5	6.6
P32134	YihM		Monomer	103.4	87.3	67.4	7.6	6.4	4.9
P32135	YihN	Cell inner membrane	Monomer	94.6	79.9	61.7	6.1	5.2	4.0
			Complex with SecB	66.1	54.0	43.2	2.4	2.0	1.6
			Complex with TF (Tig)	71.5	60.4	46.7	3.0	2.6	2.0
P32136	YihO	Cell inner membrane	Monomer	90.7	76.6	59.2	5.5	4.7	3.6

UniProtKB A. N.	Protein name	Subcellular location	Subunit	D_0 $\left(\frac{\mu m^2}{s}\right)$			D_{cyto} $\left(\frac{\mu m^2}{s}\right)$		
				310 K	303 K	293 K	310 K	303 K	293 K
			Complex with SecB	65.0	53.1	42.4	2.3	1.9	1.5
			Complex with TF (Tig)	70.0	59.2	45.7	2.9	2.4	1.9
P32137	YihP	Cell inner membrane	Monomer	91.1	77.0	59.4	5.6	4.7	3.6
			Complex with SecB	65.1	53.2	42.5	2.3	1.9	1.5
			Complex with TF (Tig)	70.2	59.3	45.8	2.9	2.4	1.9
P32138	YihQ		Monomer	77.4	65.4	50.5	3.7	3.1	2.4
P32139	YihR		Monomer	106.8	90.2	69.7	8.2	6.9	5.3
P32140	YihS		Monomer	93.7	79.2	61.2	6.0	5.1	3.9
P32141	YihT		Monomer	109.4	92.4	71.4	8.7	7.3	5.6
P0A9V8	YihU		Monomer	110.5	93.4	72.1	8.9	7.5	5.8
P32143	YihV		Monomer	109.7	92.7	71.6	8.7	7.4	5.7
P32144	YihW		Monomer	114.4	96.7	74.7	9.6	8.1	6.3
P0A8Y3	YihX		Monomer	125.0	105.6	81.6	11.7	9.9	7.7
P0A8K8	YihY	Cell inner membrane	Monomer	108.2	91.4	70.6	8.4	7.1	5.5
			Complex with SecB	69.4	56.7	45.3	2.8	2.3	1.8
			Complex with TF (Tig)	76.0	64.2	49.6	3.5	3.0	2.3
P0ADQ2	YiiD		Monomer	103.2	87.2	67.3	7.6	6.4	4.9
P0ADQ5	YiiE		Monomer	186.4	157.5	121.7	27.4	23.1	17.9
P0AFU6	YiiF		Monomer	184.4	155.8	120.3	26.8	22.6	17.5
P32151	YiiG		Monomer	100.9	85.2	65.8	7.2	6.0	4.7
P32157	YiiM		Monomer	119.8	101.2	78.2	10.7	9.0	7.0
P32160	YiiQ		Monomer	127.2	107.5	83.0	12.2	10.3	8.0
P0AF34	YiiR		Monomer	141.6	119.6	92.4	15.4	13.0	10.1
P32162	YiiS		Monomer	167.5	141.5	109.3	22.0	18.6	14.4
P32167	YiiX		Monomer	124.2	104.9	81.0	11.6	9.8	7.5
P0AF40	YijD	Cell inner membrane	Monomer	155.5	131.4	101.5	18.9	15.9	12.3
			Complex with SecB	75.6	61.7	49.3	3.5	2.9	2.3
			Complex with TF (Tig)	84.8	71.6	55.3	4.7	4.0	3.1
P0ABT8	YijE	Cell inner membrane	Monomer	108.2	91.4	70.6	8.4	7.1	5.5
			Complex with SecB	69.4	56.7	45.3	2.8	2.3	1.8
			Complex with TF (Tig)	76.0	64.2	49.6	3.5	3.0	2.3

UniProtKB A. N.	Protein name	Subcellular location	Subunit	D_0 $\left(\frac{\mu m^2}{s}\right)$			D_{cyto} $\left(\frac{\mu m^2}{s}\right)$		
				310 K	303 K	293 K	310 K	303 K	293 K
P32668	YijF		Monomer	124.6	105.2	81.3	11.6	9.8	7.6
P32677	YijO		Monomer	109.1	92.2	71.2	8.6	7.3	5.6
P09162	YjaA		Monomer	149.3	126.2	97.5	17.3	14.6	11.3
P09163	YjaB		Monomer	141.9	119.9	92.6	15.5	13.1	10.1
P32680	YjaG		Monomer	125.3	105.8	81.8	11.8	10.0	7.7
P32681	YjaH		Monomer	118.1	99.7	77.0	10.3	8.7	6.7
P0AF43	YjbB	Cell membrane	Monomer	85.8	72.5	56.0	4.8	4.1	3.1
			Complex with SecB	63.4	51.8	41.4	2.2	1.8	1.4
			Complex with TF (Tig)	68.0	57.4	44.3	2.6	2.2	1.7
P32685	YjbD		Monomer	169.0	142.8	110.3	22.4	18.9	14.6
P0AF45	YjbE		Monomer	194.3	164.1	126.8	29.7	25.1	19.4
P32687	YjbF	Cell membrane	Monomer	122.9	103.8	80.2	11.3	9.5	7.4
			Complex with SecB	72.0	58.8	47.0	3.1	2.5	2.0
			Complex with TF (Tig)	79.6	67.2	51.9	4.0	3.4	2.6
P32688	YjbG		Monomer	118.1	99.8	77.1	10.3	8.7	6.7
P32689	YjbH	Cell membrane	Monomer	76.9	65.0	50.2	3.7	3.1	2.4
			Complex with SecB	60.1	49.1	39.2	1.9	1.5	1.2
			Complex with TF (Tig)	63.8	53.9	41.6	2.2	1.9	1.4
P32690	YjbI		Monomer	90.6	76.5	59.1	5.5	4.7	3.6
P68206	YjbJ		Monomer	185.4	156.6	121.0	27.1	22.9	17.7
P32693	YjbL		Monomer	174.2	147.2	113.7	23.9	20.2	15.6
P32694	YjbM		Monomer	117.4	99.2	76.6	10.2	8.6	6.7
P0AF48	YjbQ		Monomer	144.7	122.3	94.4	16.2	13.7	10.6
P0AF50	YjbR		Monomer	153.3	129.5	100.0	18.3	15.5	11.9
P58036	YjbS		Monomer	189.3	159.9	123.5	28.2	23.8	18.4
A5A628	YjbT		Monomer	172.2	145.5	112.4	23.3	19.7	15.2
P32700	YjcB		Monomer	170.5	144.0	111.3	22.8	19.3	14.9
P32701	YjcC	Cell inner membrane	Monomer	85.0	71.8	55.5	4.7	4.0	3.1
			Complex with SecB	63.1	51.6	41.2	2.2	1.8	1.4
			Complex with TF (Tig)	67.6	57.1	44.1	2.6	2.2	1.7
P0AF52	YjcD	Cell inner membrane	Monomer	95.1	80.3	62.0	6.2	5.2	4.1

UniProtKB A. N.	Protein name	Subcellular location	Subunit	$D_0 \left(\frac{\mu m^2}{s}\right)$			$D_{\text{cyto}} \left(\frac{\mu m^2}{s}\right)$		
				310 K	303 K	293 K	310 K	303 K	293 K
			Complex with SecB	66.3	54.1	43.2	2.5	2.0	1.6
			Complex with TF (Tig)	71.7	60.6	46.8	3.0	2.6	2.0
P32703	YjcE	Cell inner membrane	Monomer	85.2	72.0	55.6	4.7	4.0	3.1
			Complex with SecB	63.2	51.6	41.2	2.2	1.8	1.4
			Complex with TF (Tig)	67.7	57.2	44.2	2.6	2.2	1.7
P32704	YjcF		Monomer	92.3	77.9	60.2	5.8	4.9	3.8
P0AF54	YjcH	Cell inner membrane	Monomer	162.4	137.2	106.0	20.6	17.4	13.5
			Complex with SecB	76.1	62.1	49.6	3.5	2.9	2.3
			Complex with TF (Tig)	85.5	72.3	55.8	4.8	4.0	3.1
P0AF56	YjcO		Monomer	120.3	101.6	78.5	10.8	9.1	7.0
P32717	YjcS		Monomer	79.1	66.8	51.6	3.9	3.3	2.6
P39267	YjcZ		Monomer	108.2	91.4	70.6	8.4	7.1	5.5
P16694	YjdA		Monomer	74.8	63.2	48.8	3.4	2.9	2.2
P0ACU7	YjdC		Monomer	126.8	107.1	82.7	12.1	10.2	7.9
P39270	YjdF	Cell inner membrane	Monomer	123.5	104.4	80.6	11.4	9.7	7.5
			Complex with SecB	72.1	58.9	47.1	3.1	2.5	2.0
			Complex with TF (Tig)	79.7	67.4	52.0	4.0	3.4	2.6
P0AF59	YjdI		Monomer	183.4	155.0	119.7	26.5	22.4	17.3
P39274	YjdJ		Monomer	169.5	143.2	110.6	22.5	19.0	14.7
P0AF61	YjdK		Monomer	163.5	138.1	106.7	20.9	17.7	13.7
P39276	YjdL	Cell inner membrane	Monomer	89.7	75.8	58.5	5.4	4.5	3.5
			Complex with SecB	64.7	52.8	42.2	2.3	1.9	1.5
			Complex with TF (Tig)	69.6	58.8	45.4	2.8	2.4	1.8
P64646	YjdO	Cell membrane	Monomer	203.6	172.0	132.8	32.6	27.5	21.3
			Complex with SecB	78.0	63.8	50.9	3.8	3.1	2.5
			Complex with TF (Tig)	88.6	74.8	57.8	5.2	4.4	3.4
Q6BEX5	YjdP		Monomer	153.5	129.7	100.2	18.3	15.5	12.0
P0AF67	YjeE		Monomer	140.6	118.8	91.7	15.2	12.8	9.9
P31806	YjeF		Monomer	88.7	74.9	57.9	5.2	4.4	3.4
P39277	YjeH	Cell inner membrane	Monomer	95.9	81.0	62.6	6.3	5.3	4.1
			Complex with SecB	66.5	54.3	43.4	2.5	2.0	1.6

UniProtKB A. N.	Protein name	Subcellular location	Subunit	D_0 $\left(\frac{\mu m^2}{s}\right)$			D_{cyto} $\left(\frac{\mu m^2}{s}\right)$		
				310 K	303 K	293 K	310 K	303 K	293 K
			Complex with TF (Tig)	72.0	60.8	47.0	3.1	2.6	2.0
P0AF70	YjeI		Monomer	160.8	135.9	105.0	20.2	17.1	13.2
P39279	YjeJ		Monomer	108.1	91.3	70.5	8.4	7.1	5.5
P39280	YjeK		Monomer	101.4	85.7	66.2	7.3	6.1	4.7
			Monomer	88.6	74.9	57.8	5.2	4.4	3.4
P39282	YjeM	Cell inner membrane	Complex with SecB	64.3	52.6	42.0	2.3	1.9	1.5
			Complex with TF (Tig)	69.2	58.4	45.1	2.8	2.3	1.8
P39283	YjeN		Monomer	161.6	136.5	105.4	20.4	17.3	13.3
			Monomer	157.4	133.0	102.7	19.3	16.3	12.6
P39284	YjeO	Cell inner membrane	Complex with SecB	75.7	61.8	49.4	3.5	2.9	2.3
			Complex with TF (Tig)	85.0	71.8	55.5	4.7	4.0	3.1
			Monomer	64.3	54.3	42.0	2.3	1.9	1.5
P39285	YjeP	Cell membrane	Complex with SecB	54.1	44.2	35.3	1.4	1.1	0.9
			Complex with TF (Tig)	56.5	47.8	36.9	1.6	1.3	1.0
P39288	YjeS		Monomer	97.3	82.2	63.5	6.6	5.5	4.3
			Monomer	196.6	166.1	128.3	30.4	25.7	19.8
P0AF73	YjeT	Cell membrane	Complex with SecB	77.8	63.6	50.8	3.8	3.1	2.5
			Complex with TF (Tig)	88.2	74.5	57.6	5.2	4.4	3.4
C1P621	YjeV		Monomer	320.7	271.0	209.3	75.7	64.0	49.4
P33222	YjfC		Monomer	95.7	80.8	62.4	6.3	5.3	4.1
			Monomer	105.6	89.2	68.9	8.0	6.7	5.2
P37772	YjfF	Cell inner membrane	Complex with SecB	68.9	56.3	44.9	2.7	2.2	1.8
			Complex with TF (Tig)	75.2	63.5	49.1	3.4	2.9	2.2
P0AF76	YjfI		Monomer	147.4	124.5	96.2	16.8	14.2	11.0
P0AF78	YjfJ		Monomer	119.8	101.2	78.2	10.7	9.0	7.0
P39293	YjfK		Monomer	120.4	101.8	78.6	10.8	9.1	7.0
			Monomer	150.3	126.9	98.1	17.5	14.8	11.4
P0AF80	YjfL	Cell inner membrane	Complex with SecB	75.1	61.4	49.0	3.4	2.8	2.2
			Complex with TF (Tig)	84.2	71.1	54.9	4.6	3.9	3.0
P39295	YjfM		Monomer	123.7	104.5	80.7	11.5	9.7	7.5
			Monomer	172.9	146.0	112.8	23.5	19.8	15.3
P0AF82	YjfN	Periplasm							

UniProtKB A. N.	Protein name	Subcellular location	Subunit	D_0 $\left(\frac{\mu m^2}{s}\right)$			D_{cyto} $\left(\frac{\mu m^2}{s}\right)$		
				310 K	303 K	293 K	310 K	303 K	293 K
			Complex with SecB	76.7	62.7	50.1	3.6	3.0	2.4
			Complex with TF (Tig)	86.5	73.1	56.5	4.9	4.2	3.2
P39298	YjfP		Monomer	115.9	97.9	75.6	9.9	8.4	6.5
P0AF86	YjfY	Periplasm	Monomer	171.5	144.9	111.9	23.1	19.5	15.1
			Complex with SecB	76.6	62.6	50.0	3.6	3.0	2.4
			Complex with TF (Tig)	86.4	73.0	56.4	4.9	4.1	3.2
P39308	YjfZ	Membrane	Monomer	112.5	95.1	73.4	9.2	7.8	6.0
			Complex with SecB	70.3	57.4	45.9	2.9	2.4	1.9
			Complex with TF (Tig)	77.1	65.2	50.3	3.7	3.1	2.4
P0A8X0	YjgA	Cytoplasm	Monomer	128.1	108.2	83.6	12.4	10.5	8.1
P27250	YjgB		Monomer	103.8	87.7	67.8	7.7	6.5	5.0
P0AF93	YjgF		Monomer	152.9	129.2	99.8	18.2	15.4	11.9
			Homotrimer	99.4	81.2	64.9	6.9	5.6	4.5
P39332	YjgH		Monomer	148.8	125.7	97.1	17.2	14.5	11.2
P39333	YjgI		Monomer	121.2	102.4	79.1	11.0	9.3	7.1
P39334	YjgJ		Monomer	127.8	108.0	83.4	12.3	10.4	8.0
P0AF96	YjgK		Monomer	140.6	118.7	91.7	15.2	12.8	9.9
P39336	YjgL		Monomer	80.6	68.1	52.6	4.1	3.5	2.7
P39337	YjgM		Monomer	135.2	114.2	88.2	14.0	11.8	9.1
P39338	YjgN	Cell inner membrane	Monomer	95.8	80.9	62.5	6.3	5.3	4.1
			Complex with SecB	66.5	54.3	43.4	2.5	2.0	1.6
			Complex with TF (Tig)	71.9	60.8	46.9	3.1	2.6	2.0
P39342	YjgR		Monomer	88.9	75.1	58.0	5.3	4.4	3.4
Q9Z3A0	YjgW		Monomer	155.3	131.2	101.3	18.8	15.9	12.3
P39349	YjgX	Cell membrane	Monomer	98.6	83.3	64.3	6.8	5.7	4.4
			Complex with SecB	67.2	54.9	43.9	2.6	2.1	1.7
			Complex with TF (Tig)	73.0	61.6	47.6	3.2	2.7	2.1
P39351	YjgZ		Monomer	159.8	135.0	104.3	20.0	16.9	13.0
P39352	YjhB	Cell inner membrane	Monomer	96.4	81.5	62.9	6.4	5.4	4.2
			Complex with SecB	66.6	54.4	43.5	2.5	2.0	1.6
			Complex with TF (Tig)	72.2	61.0	47.1	3.1	2.6	2.0

UniProtKB A. N.	Protein name	Subcellular location	Subunit	D_0 $\left(\frac{\mu m^2}{s}\right)$			D_{cyto} $\left(\frac{\mu m^2}{s}\right)$		
				310 K	303 K	293 K	310 K	303 K	293 K
P39353	YjhC		Monomer	98.9	83.5	64.5	6.8	5.8	4.5
P39354	YjhD		Monomer	181.2	153.1	118.3	25.8	21.8	16.9
P39355	YjhE	Cell membrane	Monomer	182.1	153.8	118.8	26.1	22.0	17.0
			Complex with SecB	77.2	63.1	50.4	3.7	3.0	2.4
			Complex with TF (Tig)	87.3	73.7	56.9	5.0	4.2	3.3
P39357	YjhF	Cell inner membrane	Monomer	94.0	79.5	61.4	6.0	5.1	3.9
			Complex with SecB	66.0	53.9	43.1	2.4	2.0	1.6
			Complex with TF (Tig)	71.3	60.3	46.5	3.0	2.5	2.0
P39358	YjhG		Monomer	80.4	68.0	52.5	4.1	3.5	2.7
P39359	YjhH		Monomer	108.4	91.6	70.7	8.5	7.2	5.5
P39360	YjhI		Monomer	112.3	94.9	73.3	9.2	7.8	6.0
P39367	YjhP	Membrane	Monomer	116.3	98.2	75.9	10.0	8.4	6.5
			Complex with SecB	71.0	58.0	46.3	3.0	2.4	1.9
			Complex with TF (Tig)	78.1	66.0	51.0	3.8	3.2	2.5
P39368	YjhQ		Monomer	131.6	111.2	85.9	13.2	11.1	8.6
P39369	YjhR		Monomer	102.2	86.4	66.7	7.4	6.2	4.8
P39356	YjhU		Monomer	104.3	88.1	68.1	7.7	6.5	5.1
Q2EEU2	YjhX		Monomer	174.4	147.3	113.8	23.9	20.2	15.6
P24203	YjiA		Monomer	104.8	88.5	68.4	7.8	6.6	5.1
P39374	YjiC		Monomer	111.3	94.0	72.6	9.0	7.6	5.9
P0AEH8	YjiG	Cell inner membrane	Monomer	142.8	120.7	93.2	15.7	13.3	10.3
			Complex with SecB	74.5	60.8	48.6	3.4	2.7	2.2
			Complex with TF (Tig)	83.1	70.2	54.3	4.5	3.8	2.9
P39379	YjiH	Cell membrane	Monomer	122.8	103.8	80.1	11.3	9.5	7.4
			Complex with SecB	72.0	58.8	47.0	3.1	2.5	2.0
			Complex with TF (Tig)	79.6	67.2	51.9	4.0	3.4	2.6
P39381	YjiJ	Cell membrane	Monomer	98.9	83.5	64.5	6.8	5.8	4.5
			Complex with SecB	67.3	55.0	43.9	2.6	2.1	1.7
			Complex with TF (Tig)	73.0	61.7	47.7	3.2	2.7	2.1
P39382	YjiK	Cell membrane	Monomer	109.4	92.4	71.4	8.7	7.3	5.7
			Complex with SecB	69.7	56.9	45.5	2.8	2.3	1.8

UniProtKB A. N.	Protein name	Subcellular location	Subunit	$D_0 \left(\frac{\mu m^2}{s}\right)$			$D_{\text{cyto}} \left(\frac{\mu m^2}{s}\right)$		
				310 K	303 K	293 K	310 K	303 K	293 K
			Complex with TF (Tig)	76.3	64.5	49.8	3.6	3.0	2.3
P39383	YjiL		Monomer	116.4	98.4	76.0	10.0	8.4	6.5
			Homodimer	88.7	72.5	57.9	5.2	4.3	3.4
P39384	YjiM		Monomer	97.6	82.5	63.7	6.6	5.6	4.3
P39385	YjiN	Cell membrane	Monomer	93.1	78.6	60.7	5.9	5.0	3.8
			Complex with SecB	65.7	53.7	42.9	2.4	2.0	1.6
			Complex with TF (Tig)	71.0	59.9	46.3	3.0	2.5	1.9
P39387	YjiP		Monomer	104.5	88.3	68.2	7.8	6.6	5.1
P39389	YjiR		Monomer	89.7	75.8	58.5	5.4	4.5	3.5
P39390	YjiS		Monomer	201.7	170.4	131.6	32.0	27.0	20.9
P39391	YjiT		Monomer	86.3	72.9	56.3	4.9	4.1	3.2
P39393	YjiV		Monomer	69.0	58.3	45.1	2.8	2.3	1.8
P0ADC8	YjiX		Monomer	190.8	161.2	124.5	28.7	24.2	18.7
P39396	YjiY	Cell inner membrane	Monomer	77.4	65.4	50.5	3.7	3.1	2.4
			Complex with SecB	60.2	49.2	39.3	1.9	1.5	1.2
			Complex with TF (Tig)	64.0	54.1	41.8	2.2	1.9	1.5
P18390	YjjA		Monomer	139.0	117.4	90.7	14.8	12.5	9.7
P0ADD2	YjjB	Cell membrane	Monomer	140.0	118.2	91.3	15.1	12.7	9.8
			Complex with SecB	74.2	60.6	48.4	3.3	2.7	2.2
			Complex with TF (Tig)	82.7	69.9	54.0	4.4	3.7	2.9
P0A8Y1	YjjG	Cytoplasm	Monomer	119.9	101.3	78.2	10.7	9.0	7.0
			Homodimer	91.4	74.6	59.6	5.6	4.6	3.7
P37342	YjjI		Monomer	86.6	73.2	56.5	4.9	4.2	3.2
P39410	YjjJ		Monomer	92.0	77.7	60.0	5.7	4.8	3.7
P0A9W3	YjjK		Monomer	84.1	71.1	54.9	4.6	3.9	3.0
P39398	YjjL	Cell inner membrane	Monomer	92.2	77.9	60.2	5.8	4.9	3.8
			Complex with SecB	65.4	53.5	42.7	2.4	1.9	1.6
			Complex with TF (Tig)	70.6	59.7	46.1	2.9	2.5	1.9
P39399	YjjM		Monomer	105.1	88.8	68.6	7.9	6.7	5.1
P39400	YjjN		Monomer	103.9	87.8	67.8	7.7	6.5	5.0
P0ADD5	YjjP	Cell inner membrane	Monomer	115.2	97.3	75.2	9.8	8.2	6.4

UniProtKB A. N.	Protein name	Subcellular location	Subunit	D_0 $\left(\frac{\mu m^2}{s}\right)$			D_{cyto} $\left(\frac{\mu m^2}{s}\right)$		
				310 K	303 K	293 K	310 K	303 K	293 K
			Complex with SecB	70.8	57.8	46.2	2.9	2.4	1.9
			Complex with TF (Tig)	77.8	65.7	50.8	3.8	3.2	2.5
P0ADD7	YjjQ		Monomer	116.8	98.7	76.2	10.1	8.5	6.6
			Homodimer	89.0	72.7	58.1	5.3	4.3	3.4
P39407	YjjU		Monomer	100.4	84.8	65.5	7.1	6.0	4.6
P39408	YjjV		Monomer	113.8	96.1	74.3	9.5	8.0	6.2
P39409	YjjW		Monomer	110.0	93.0	71.8	8.8	7.4	5.7
P39411	YjjX		Monomer	136.4	115.2	89.0	14.2	12.0	9.3
P0ADD9	YjjY		Monomer	228.4	193.0	149.1	40.7	34.4	26.6
P55914	YjjZ		Monomer	182.2	153.9	118.9	26.1	22.1	17.1
P75678	YkfA		Monomer	109.5	92.5	71.5	8.7	7.3	5.7
P77162	YkfB		Monomer	140.0	118.3	91.4	15.1	12.7	9.8
Q47688	YkfC		Monomer	97.2	82.2	63.5	6.6	5.5	4.3
P75677	YkfF		Monomer	179.7	151.8	117.3	25.4	21.5	16.6
Q47685	YkfG		Monomer	136.7	115.5	89.2	14.3	12.1	9.3
Q9XB42	YkfH		Monomer	183.2	154.8	119.5	26.4	22.3	17.2
P77692	YkfI		Monomer	156.1	131.9	101.9	19.0	16.1	12.4
P75675	YkfJ		Monomer	173.8	146.8	113.4	23.7	20.0	15.5
A5A605	YkfM	Membrane	Monomer	136.7	115.5	89.2	14.3	12.1	9.3
			Complex with SecB	73.8	60.3	48.2	3.3	2.7	2.1
			Complex with TF (Tig)	82.2	69.4	53.6	4.3	3.7	2.8
P77601	YkgA		Monomer	114.5	96.7	74.7	9.6	8.1	6.3
P75685	YkgB	Cell inner membrane	Monomer	126.9	107.2	82.8	12.1	10.3	7.9
			Complex with SecB	72.6	59.3	47.4	3.1	2.6	2.0
			Complex with TF (Tig)	80.4	67.9	52.5	4.1	3.5	2.7
P77212	YkgC		Monomer	93.1	78.6	60.7	5.9	5.0	3.8
P77379	YkgD		Monomer	110.1	93.0	71.8	8.8	7.4	5.7
P77252	YkgE		Monomer	118.6	100.2	77.4	10.4	8.8	6.8
P77536	YkgF		Monomer	89.7	75.8	58.5	5.4	4.5	3.5
P77433	YkgG		Monomer	120.1	101.4	78.3	10.7	9.1	7.0
P77180	YkgH	Cell membrane	Monomer	119.4	100.8	77.9	10.6	8.9	6.9

UniProtKB A. N.	Protein name	Subcellular location	Subunit	D_0 $\left(\frac{\mu m^2}{s}\right)$			D_{cyto} $\left(\frac{\mu m^2}{s}\right)$		
				310 K	303 K	293 K	310 K	303 K	293 K
			Complex with SecB	71.5	58.4	46.6	3.0	2.5	2.0
			Complex with TF (Tig)	78.8	66.6	51.4	3.9	3.3	2.5
P75687	YkgI	Periplasm	Monomer	184.1	155.5	120.1	26.7	22.5	17.4
			Complex with SecB	77.3	63.1	50.4	3.7	3.0	2.4
			Complex with TF (Tig)	87.4	73.8	57.0	5.0	4.3	3.3
P0AAL9	YkgJ		Monomer	161.7	136.6	105.5	20.5	17.3	13.3
P56257	YkgL		Monomer	181.6	153.5	118.5	26.0	21.9	16.9
Q79E92	YkgN		Monomer	156.3	132.1	102.0	19.1	16.1	12.4
C1P5Z8	YkgR	Membrane	Monomer	247.1	208.8	161.2	47.2	39.9	30.8
			Complex with SecB	79.1	64.6	51.6	3.9	3.2	2.6
			Complex with TF (Tig)	90.3	76.3	58.9	5.5	4.6	3.6
P75704	YkiA		Monomer	168.5	142.4	110.0	22.3	18.8	14.5
P77473	YlaB	Cell inner membrane	Monomer	86.2	72.8	56.2	4.9	4.1	3.2
			Complex with SecB	63.5	51.9	41.5	2.2	1.8	1.4
			Complex with TF (Tig)	68.1	57.6	44.5	2.7	2.2	1.7
P0AAS0	YlaC	Cell inner membrane	Monomer	136.2	115.1	88.9	14.2	12.0	9.3
			Complex with SecB	73.8	60.3	48.1	3.3	2.7	2.1
			Complex with TF (Tig)	82.1	69.4	53.6	4.3	3.6	2.8
P75713	YlbA		Monomer	114.1	96.4	74.4	9.5	8.1	6.2
P77129	YlbE		Monomer	95.7	80.8	62.4	6.3	5.3	4.1
P0AAS5	YlbF		Monomer	112.7	95.3	73.6	9.3	7.8	6.1
P77688	YlbG		Monomer	151.6	128.0	98.9	17.9	15.1	11.7
P77759	YlbH		Monomer	116.9	98.8	76.3	10.1	8.5	6.6
P77087	YlcE		Monomer	197.4	166.8	128.8	30.7	25.9	20.0
Q47272	YlcG		Monomer	218.2	184.3	142.4	37.3	31.5	24.3
Q47268	YlcH		Monomer	256.3	216.5	167.2	50.5	42.7	33.0
A5A607	YlcI		Monomer	192.9	163.0	125.9	29.3	24.7	19.1
P75800	YliE		Monomer	72.9	61.6	47.6	3.2	2.7	2.1
P75801	YliF	Cell membrane	Monomer	91.9	77.6	60.0	5.7	4.8	3.7
			Complex with SecB	65.3	53.4	42.6	2.4	1.9	1.5
			Complex with TF (Tig)	70.5	59.6	46.0	2.9	2.5	1.9

UniProtKB A. N.	Protein name	Subcellular location	Subunit	D_0 $\left(\frac{\mu m^2}{s}\right)$			D_{cyto} $\left(\frac{\mu m^2}{s}\right)$		
				310 K	303 K	293 K	310 K	303 K	293 K
P75804	YliI		Monomer	99.2	83.8	64.7	6.9	5.8	4.5
P0ACA7	YliJ		Monomer	123.0	103.9	80.3	11.3	9.6	7.4
P75786	YliL		Monomer	172.1	145.4	112.3	23.3	19.7	15.2
P0AB10	YmbA	Cell membrane	Monomer	129.9	109.7	84.7	12.8	10.8	8.3
			Complex with SecB	73.0	59.6	47.6	3.2	2.6	2.1
			Complex with TF (Tig)	81.0	68.4	52.9	4.2	3.5	2.7
P75917	YmdA		Monomer	165.1	139.4	107.7	21.3	18.0	13.9
P0A8D6	YmdB		Monomer	134.5	113.6	87.8	13.8	11.7	9.0
			Homodimer	102.5	83.7	66.9	7.4	6.1	4.8
P75919	YmdC		Monomer	89.3	75.4	58.3	5.3	4.5	3.5
Q7DFV4	YmdE		Monomer	160.6	135.7	104.8	20.2	17.0	13.2
P56614	YmdF		Monomer	212.4	179.4	138.6	35.4	29.9	23.1
P75962	YmfA	Cell inner membrane	Monomer	138.8	117.3	90.6	14.8	12.5	9.6
			Complex with SecB	74.0	60.5	48.3	3.3	2.7	2.2
			Complex with TF (Tig)	82.5	69.7	53.9	4.4	3.7	2.9
P75967	YmfD		Monomer	120.2	101.5	78.4	10.7	9.1	7.0
P75968	YmfE	Cell membrane	Monomer	115.9	97.9	75.6	9.9	8.4	6.5
			Complex with SecB	70.9	57.9	46.3	2.9	2.4	1.9
			Complex with TF (Tig)	78.0	65.9	50.9	3.8	3.2	2.5
P75972	YmfI		Monomer	156.2	132.0	101.9	19.0	16.1	12.4
P75973	YmfJ		Monomer	163.6	138.2	106.7	20.9	17.7	13.7
P75976	YmfL		Monomer	130.9	110.6	85.4	13.0	11.0	8.5
P75977	YmfM		Monomer	159.8	135.0	104.3	20.0	16.9	13.0
P75978	YmfN		Monomer	91.2	77.0	59.5	5.6	4.7	3.7
P75980	YmfO		Monomer	138.2	116.8	90.2	14.6	12.4	9.6
P75981	YmfP		Monomer	114.9	97.0	74.9	9.7	8.2	6.3
P75982	YmfQ		Monomer	127.5	107.7	83.2	12.3	10.4	8.0
P75979	YmfR	Cell membrane	Monomer	205.7	173.8	134.3	33.3	28.1	21.7
			Complex with SecB	78.1	63.8	51.0	3.8	3.1	2.5
			Complex with TF (Tig)	88.7	74.9	57.9	5.2	4.4	3.4
P09154	YmfS		Monomer	145.5	122.9	95.0	16.4	13.8	10.7

UniProtKB A. N.	Protein name	Subcellular location	Subunit	D_0 $\left(\frac{\mu m^2}{s}\right)$			D_{cyto} $\left(\frac{\mu m^2}{s}\right)$		
				310 K	303 K	293 K	310 K	303 K	293 K
P75992	YmgA		Monomer	170.2	143.8	111.1	22.8	19.2	14.8
P75994	YmgC		Monomer	174.6	147.5	113.9	24.0	20.2	15.6
P0AB46	YmgD		Monomer	161.4	136.4	105.3	20.4	17.2	13.3
P76011	YmgE	Cell membrane	Monomer	181.6	153.4	118.5	26.0	21.9	16.9
			Complex with SecB	77.2	63.0	50.4	3.7	3.0	2.4
			Complex with TF (Tig)	87.2	73.7	56.9	5.0	4.2	3.3
P58034	YmgF		Monomer	186.0	157.1	121.4	27.2	23.0	17.8
Q7DFV3	YmgG		Monomer	167.3	141.4	109.2	22.0	18.6	14.3
A5A611	YmgI		Monomer	204.0	172.4	133.1	32.7	27.6	21.4
A5A612	YmgJ		Monomer	194.5	164.3	126.9	29.8	25.2	19.4
P0CB62	YmiA	Membrane	Monomer	228.8	193.3	149.3	40.8	34.5	26.6
			Complex with SecB	78.7	64.3	51.4	3.9	3.2	2.5
			Complex with TF (Tig)	89.7	75.8	58.5	5.4	4.5	3.5
C1P5Z9	YmiB	Membrane	Monomer	244.2	206.3	159.3	46.2	39.0	30.1
			Complex with SecB	79.0	64.6	51.6	3.9	3.2	2.6
			Complex with TF (Tig)	90.2	76.2	58.8	5.5	4.6	3.6
P0ACV8	YmjA		Monomer	177.3	149.8	115.7	24.7	20.9	16.1
Q2EER5	YmjC		Monomer	201.5	170.3	131.5	31.9	27.0	20.8
P0CD93	YmjD		Monomer	298.7	252.3	194.9	66.7	56.4	43.6
P77658	YnaA		Monomer	103.2	87.2	67.4	7.6	6.4	4.9
P76073	YnaE		Monomer	171.8	145.1	112.1	23.2	19.6	15.1
P0AEB5	YnaI	Cell inner membrane	Monomer	101.4	85.7	66.2	7.3	6.1	4.7
			Complex with SecB	67.9	55.5	44.3	2.6	2.1	1.7
			Complex with TF (Tig)	73.9	62.4	48.2	3.3	2.8	2.1
P64445	YnaJ	Cell membrane	Monomer	177.2	149.7	115.6	24.7	20.9	16.1
			Complex with SecB	76.9	62.9	50.2	3.7	3.0	2.4
			Complex with TF (Tig)	86.9	73.4	56.7	5.0	4.2	3.2
P76068	YnaK		Monomer	174.0	147.0	113.5	23.8	20.1	15.5
P76090	YnbA	Cell inner membrane	Monomer	125.5	106.0	81.9	11.8	10.0	7.7
			Complex with SecB	72.4	59.1	47.2	3.1	2.5	2.0
			Complex with TF (Tig)	80.1	67.7	52.3	4.1	3.4	2.6

UniProtKB A. N.	Protein name	Subcellular location	Subunit	D_0 $\left(\frac{\mu m^2}{s}\right)$			D_{cyto} $\left(\frac{\mu m^2}{s}\right)$		
				310 K	303 K	293 K	310 K	303 K	293 K
P76091	YnbB	Cell membrane	Monomer	108.0	91.2	70.5	8.4	7.1	5.5
			Complex with SecB	69.4	56.7	45.3	2.8	2.3	1.8
			Complex with TF (Tig)	75.9	64.1	49.5	3.5	3.0	2.3
P76092	YnbC		Monomer	82.6	69.8	53.9	4.4	3.7	2.9
P76093	YnbD	Cell membrane	Monomer	92.1	77.8	60.1	5.7	4.9	3.8
			Complex with SecB	65.4	53.4	42.7	2.4	1.9	1.6
			Complex with TF (Tig)	70.6	59.6	46.1	2.9	2.5	1.9
P64448	YnbE		Monomer	200.2	169.1	130.6	31.5	26.6	20.6
C1P600	YnbG		Monomer	290.0	245.0	189.3	63.3	53.5	41.3
P76112	YncA		Monomer	133.5	112.7	87.1	13.6	11.5	8.9
P76113	YncB		Monomer	102.6	86.7	67.0	7.5	6.3	4.9
P76115	YncD	Cell outer membrane	Monomer	77.4	65.4	50.5	3.7	3.1	2.4
			Complex with SecB	60.3	49.2	39.3	1.9	1.5	1.2
			Complex with TF (Tig)	64.0	54.1	41.8	2.2	1.9	1.5
P76116	YncE		Monomer	101.6	85.8	66.3	7.3	6.1	4.7
P76117	YncG		Monomer	123.8	104.6	80.8	11.5	9.7	7.5
P76118	YncH		Monomer	185.9	157.0	121.3	27.2	23.0	17.7
P76119	YncI		Monomer	97.7	82.5	63.8	6.6	5.6	4.3
P64459	YncJ		Monomer	182.1	153.9	118.8	26.1	22.1	17.0
A5A615	YncL	Membrane	Monomer	259.3	219.0	169.2	51.6	43.6	33.7
			Complex with SecB	79.3	64.8	51.7	3.9	3.2	2.6
			Complex with TF (Tig)	90.6	76.5	59.1	5.5	4.7	3.6
P76146	YneE	Cell membrane	Monomer	105.8	89.4	69.0	8.0	6.8	5.2
			Complex with SecB	68.9	56.3	45.0	2.7	2.2	1.8
			Complex with TF (Tig)	75.3	63.6	49.1	3.5	2.9	2.3
P76147	YneF	Cell membrane	Monomer	106.4	89.9	69.4	8.1	6.9	5.3
			Complex with SecB	69.0	56.4	45.1	2.7	2.2	1.8
			Complex with TF (Tig)	75.4	63.7	49.2	3.5	2.9	2.3
P76148	YneG		Monomer	153.2	129.4	100.0	18.3	15.4	11.9
P77309	YneJ		Monomer	108.7	91.8	70.9	8.5	7.2	5.6
P76150	YneK		Monomer	97.5	82.4	63.6	6.6	5.6	4.3

UniProtKB A. N.	Protein name	Subcellular location	Subunit	D_0 $\left(\frac{\mu m^2}{s}\right)$			D_{cyto} $\left(\frac{\mu m^2}{s}\right)$		
				310 K	303 K	293 K	310 K	303 K	293 K
P76138	YneL		Monomer	199.8	168.8	130.4	31.4	26.5	20.5
			Monomer	260.1	219.7	169.7	51.9	43.9	33.9
A5A616	YneM	Membrane	Complex with SecB	79.3	64.8	51.8	4.0	3.2	2.6
			Complex with TF (Tig)	90.6	76.5	59.1	5.5	4.7	3.6
			Monomer	161.0	136.0	105.1	20.3	17.1	13.2
P76169	YnfA	Cell inner membrane	Complex with SecB	76.0	62.1	49.6	3.5	2.9	2.3
			Complex with TF (Tig)	85.4	72.2	55.7	4.8	4.0	3.1
P76170	YnfB		Monomer	156.1	131.9	101.9	19.0	16.1	12.4
			Monomer	117.7	99.5	76.8	10.3	8.7	6.7
P67553	YnfC	Cell membrane	Complex with SecB	71.2	58.2	46.5	3.0	2.4	1.9
			Complex with TF (Tig)	78.4	66.3	51.2	3.8	3.2	2.5
P76172	YnfD		Monomer	169.4	143.1	110.5	22.5	19.0	14.7
P77374	YnfE	Cell membrane	Monomer	73.0	61.7	47.6	3.2	2.7	2.1
P77783	YnfF	Cell membrane	Monomer	72.9	61.6	47.6	3.2	2.7	2.1
P0AAJ1	YnfG		Monomer	125.0	105.6	81.6	11.7	9.9	7.7
			Monomer	111.4	94.1	72.7	9.0	7.6	5.9
P76173	YnfH	Cell inner membrane	Complex with SecB	70.1	57.2	45.7	2.9	2.3	1.9
			Complex with TF (Tig)	76.8	64.9	50.1	3.6	3.1	2.4
P77559	YnfL		Monomer	107.7	91.0	70.3	8.3	7.1	5.4
			Monomer	95.5	80.6	62.3	6.3	5.3	4.1
P43531	YnfM	Cell inner membrane	Complex with SecB	66.4	54.2	43.3	2.5	2.0	1.6
			Complex with TF (Tig)	71.8	60.7	46.9	3.1	2.6	2.0
P76157	YnfN		Monomer	216.0	182.4	140.9	36.5	30.9	23.8
Q2EES0	YnfO		Monomer	179.6	151.7	117.2	25.4	21.4	16.6
			Monomer	276.6	233.7	180.5	58.1	49.1	37.9
A5A618	YnhF	Membrane	Complex with SecB	79.5	65.0	51.9	4.0	3.3	2.6
			Complex with TF (Tig)	91.0	76.8	59.4	5.6	4.7	3.6
			Monomer	104.3	88.1	68.1	7.7	6.5	5.1
P76193	YnhG	Periplasm	Complex with SecB	68.6	56.0	44.8	2.7	2.2	1.8
			Complex with TF (Tig)	74.8	63.2	48.8	3.4	2.9	2.2
P77739	YniA		Monomer	108.7	91.9	71.0	8.5	7.2	5.6

UniProtKB A. N.	Protein name	Subcellular location	Subunit	D_0 $\left(\frac{\mu m^2}{s}\right)$			D_{cyto} $\left(\frac{\mu m^2}{s}\right)$		
				310 K	303 K	293 K	310 K	303 K	293 K
P76208	YniB	Cell membrane	Monomer	130.5	110.3	85.2	12.9	10.9	8.4
			Complex with SecB	73.1	59.7	47.7	3.2	2.6	2.1
			Complex with TF (Tig)	81.1	68.5	52.9	4.2	3.5	2.7
P77247	YniC		Monomer	121.7	102.9	79.4	11.1	9.3	7.2
Q2EES1	YniD	Membrane	Monomer	244.3	206.4	159.4	46.2	39.1	30.2
			Complex with SecB	79.0	64.6	51.6	3.9	3.2	2.6
			Complex with TF (Tig)	90.2	76.2	58.9	5.5	4.6	3.6
P76222	YnjA		Monomer	130.1	109.9	84.9	12.8	10.8	8.4
P76223	YnjB		Monomer	97.5	82.4	63.6	6.6	5.6	4.3
P76224	YnjC	Cell inner membrane	Monomer	87.7	74.1	57.2	5.1	4.3	3.3
			Complex with SecB	64.0	52.3	41.8	2.2	1.8	1.5
			Complex with TF (Tig)	68.8	58.1	44.9	2.7	2.3	1.8
P76909	YnjD		Monomer	122.4	103.4	79.9	11.2	9.5	7.3
P78067	YnjE	Periplasm	Monomer	93.1	78.7	60.8	5.9	5.0	3.8
			Complex with SecB	65.7	53.7	42.9	2.4	2.0	1.6
			Complex with TF (Tig)	71.0	60.0	46.3	3.0	2.5	1.9
P76226	YnjF	Cell inner membrane	Monomer	125.0	105.6	81.6	11.7	9.9	7.7
			Complex with SecB	72.3	59.1	47.2	3.1	2.5	2.0
			Complex with TF (Tig)	80.0	67.6	52.2	4.0	3.4	2.6
P76227	YnjH		Monomer	172.9	146.0	112.8	23.5	19.8	15.3
P76228	YnjI	Cell inner membrane	Monomer	99.9	84.4	65.2	7.0	5.9	4.6
			Complex with SecB	67.5	55.2	44.1	2.6	2.1	1.7
			Complex with TF (Tig)	73.4	62.0	47.9	3.2	2.7	2.1
P76257	YoaA		Monomer	80.3	67.8	52.4	4.1	3.4	2.7
P0AEB7	YoaB		Monomer	158.1	133.6	103.2	19.5	16.5	12.7
P64490	YoaC		Monomer	167.2	141.2	109.1	21.9	18.5	14.3
P0AEC0	YoaE	Cell inner membrane	Monomer	87.5	73.9	57.1	5.1	4.3	3.3
			Complex with SecB	64.0	52.3	41.7	2.2	1.8	1.5
			Complex with TF (Tig)	68.7	58.0	44.8	2.7	2.3	1.8
P64493	YoaF		Monomer	180.2	152.3	117.6	25.6	21.6	16.7
P64496	YoaG		Monomer	202.9	171.4	132.4	32.4	27.4	21.1

UniProtKB A. N.	Protein name	Subcellular location	Subunit	D_0 $\left(\frac{\mu m^2}{s}\right)$ 310 K	D_0 303 K	D_0 293 K	D_{cyto} $\left(\frac{\mu m^2}{s}\right)$ 310 K	D_{cyto} 303 K	D_{cyto} 293 K
			Homodimer	154.7	126.3	100.9	18.6	15.2	12.2
P67338	YoaH		Monomer	203.6	172.0	132.9	32.6	27.5	21.3
P76239	YoaI	Membrane	Monomer	252.8	213.6	165.0	49.3	41.6	32.1
			Complex with SecB	79.2	64.7	51.7	3.9	3.2	2.6
			Complex with TF (Tig)	90.4	76.4	59.0	5.5	4.6	3.6
C1P603	YoaJ	Membrane	Monomer	288.6	243.8	188.3	62.8	53.0	41.0
			Complex with SecB	79.7	65.1	52.0	4.0	3.3	2.6
			Complex with TF (Tig)	91.2	77.0	59.5	5.6	4.7	3.7
C1P602	YoaK	Membrane	Monomer	261.6	221.0	170.7	52.5	44.3	34.2
			Complex with SecB	79.3	64.8	51.8	4.0	3.2	2.6
			Complex with TF (Tig)	90.6	76.6	59.1	5.5	4.7	3.6
P0AA57	YobA	Periplasm	Monomer	153.8	129.9	100.3	18.4	15.6	12.0
			Complex with SecB	75.4	61.6	49.2	3.5	2.8	2.3
			Complex with TF (Tig)	84.6	71.5	55.2	4.7	3.9	3.0
P76280	YobB		Monomer	121.6	102.7	79.4	11.0	9.3	7.2
P67601	YobD	Cell inner membrane	Monomer	138.2	116.7	90.2	14.6	12.4	9.6
			Complex with SecB	74.0	60.4	48.3	3.3	2.7	2.2
			Complex with TF (Tig)	82.4	69.6	53.8	4.4	3.7	2.8
P64508	YobF		Monomer	222.7	188.2	145.3	38.8	32.8	25.3
Q2MB16	YobH		Monomer	183.7	155.2	119.9	26.6	22.5	17.3
C1P604	YobI		Monomer	289.7	244.7	189.0	63.2	53.4	41.2
P76345	YodB	Cell inner membrane	Monomer	130.6	110.3	85.2	12.9	10.9	8.4
			Complex with SecB	73.1	59.7	47.7	3.2	2.6	2.1
			Complex with TF (Tig)	81.1	68.5	52.9	4.2	3.5	2.7
P64517	YodC		Monomer	202.1	170.7	131.9	32.1	27.1	21.0
P64519	YodD		Monomer	183.2	154.8	119.5	26.4	22.3	17.2
P76356	YoeA		Monomer	137.1	115.8	89.4	14.4	12.2	9.4
P69348	YoeB		Monomer	171.1	144.5	111.6	23.0	19.4	15.0
Q2EES3	YoeF		Monomer	155.8	131.6	101.7	18.9	16.0	12.4
C1P606	YoeI		Monomer	310.0	261.9	202.3	71.3	60.2	46.5
P0AD17	YohC	Cell inner membrane	Monomer	127.4	107.6	83.1	12.2	10.3	8.0

UniProtKB A. N.	Protein name	Subcellular location	Subunit	D_0 $\left(\frac{\mu m^2}{s}\right)$			D_{cyto} $\left(\frac{\mu m^2}{s}\right)$		
				310 K	303 K	293 K	310 K	303 K	293 K
			Complex with SecB	72.7	59.4	47.4	3.1	2.6	2.1
			Complex with TF (Tig)	80.5	68.0	52.5	4.1	3.5	2.7
P33366	YohD	Cell inner membrane	Monomer	127.9	108.1	83.5	12.4	10.4	8.1
			Complex with SecB	72.7	59.4	47.5	3.2	2.6	2.1
			Complex with TF (Tig)	80.6	68.1	52.6	4.1	3.5	2.7
P33368	YohF		Monomer	117.0	98.8	76.3	10.1	8.5	6.6
P60632	YohJ	Cell inner membrane	Monomer	148.8	125.7	97.1	17.2	14.5	11.2
			Complex with SecB	75.0	61.3	49.0	3.4	2.8	2.2
			Complex with TF (Tig)	84.0	70.9	54.8	4.6	3.9	3.0
P0AD19	YohK	Cell inner membrane	Monomer	121.5	102.6	79.3	11.0	9.3	7.2
			Complex with SecB	71.8	58.7	46.9	3.1	2.5	2.0
			Complex with TF (Tig)	79.3	67.0	51.7	3.9	3.3	2.6
Q2EES6	YohO	Cell inner membrane	Monomer	256.3	216.5	167.2	50.5	42.7	33.0
			Complex with SecB	79.2	64.7	51.7	3.9	3.2	2.6
			Complex with TF (Tig)	90.5	76.5	59.1	5.5	4.7	3.6
C1P609	YohP	Membrane	Monomer	273.3	230.9	178.4	56.9	48.0	37.1
			Complex with SecB	79.5	64.9	51.9	4.0	3.2	2.6
			Complex with TF (Tig)	90.9	76.8	59.3	5.6	4.7	3.6
P33941	YojI	Cell inner membrane	Monomer	84.6	71.5	55.2	4.7	3.9	3.0
			Complex with SecB	63.0	51.5	41.1	2.1	1.8	1.4
			Complex with TF (Tig)	67.5	57.0	44.0	2.6	2.2	1.7
A5A619	YojO		Monomer	207.8	175.6	135.6	33.9	28.7	22.1
P0AA93	YpdA	Cell inner membrane	Monomer	84.0	71.0	54.8	4.6	3.9	3.0
			Complex with SecB	62.8	51.3	41.0	2.1	1.7	1.4
			Complex with TF (Tig)	67.2	56.8	43.8	2.6	2.2	1.7
P0AE39	YpdB		Monomer	114.1	96.4	74.4	9.5	8.1	6.2
P77396	YpdC		Monomer	108.9	92.0	71.0	8.6	7.2	5.6
P77585	YpdE		Monomer	102.9	86.9	67.1	7.5	6.3	4.9
P76524	YpdF		Monomer	100.6	85.0	65.6	7.1	6.0	4.6
O32528	YpdI	Cell membrane	Monomer	171.4	144.8	111.9	23.1	19.5	15.1
			Complex with SecB	76.6	62.6	50.0	3.6	3.0	2.4

UniProtKB A. N.	Protein name	Subcellular location	Subunit	D_0 $\left(\frac{\mu m^2}{s}\right)$			D_{cyto} $\left(\frac{\mu m^2}{s}\right)$		
				310 K	303 K	293 K	310 K	303 K	293 K
			Complex with TF (Tig)	86.4	73.0	56.4	4.9	4.1	3.2
Q2EET0	YpdJ		Monomer	221.0	186.7	144.2	38.2	32.3	24.9
C1P610	YpdK	Membrane	Monomer	288.1	243.4	188.0	62.5	52.8	40.8
			Complex with SecB	79.7	65.1	52.0	4.0	3.3	2.6
			Complex with TF (Tig)	91.2	77.0	59.5	5.6	4.7	3.7
P76539	YpeA		Monomer	142.4	120.3	92.9	15.6	13.2	10.2
P0AD40	YpeB		Monomer	184.6	155.9	120.5	26.8	22.7	17.5
P64542	YpeC		Monomer	154.4	130.5	100.8	18.6	15.7	12.1
P76559	YpfG		Monomer	101.4	85.7	66.2	7.3	6.1	4.7
P76561	YpfH		Monomer	120.7	102.0	78.8	10.9	9.2	7.1
P64429	YpfJ	Membrane	Monomer	110.1	93.0	71.8	8.8	7.4	5.7
			Complex with SecB	69.8	57.0	45.5	2.8	2.3	1.8
			Complex with TF (Tig)	76.5	64.6	49.9	3.6	3.0	2.3
A5A621	YpfM	Cytoplasm	Monomer	301.5	254.7	196.7	67.8	57.3	44.3
Q2EET2	YpfN	Cell membrane	Monomer	187.6	158.5	122.4	27.7	23.4	18.1
			Complex with SecB	77.4	63.3	50.5	3.7	3.0	2.4
			Complex with TF (Tig)	87.6	74.0	57.2	5.1	4.3	3.3
P0AD47	YphA	Cell inner membrane	Monomer	146.4	123.6	95.5	16.6	14.0	10.8
			Complex with SecB	74.8	61.1	48.8	3.4	2.8	2.2
			Complex with TF (Tig)	83.6	70.7	54.6	4.5	3.8	3.0
P76584	YphB		Monomer	108.4	91.6	70.7	8.5	7.2	5.5
P77360	YphC		Monomer	102.2	86.3	66.7	7.4	6.2	4.8
P77315	YphD	Cell inner membrane	Monomer	106.2	89.7	69.3	8.1	6.8	5.3
			Complex with SecB	69.0	56.4	45.0	2.7	2.2	1.8
			Complex with TF (Tig)	75.4	63.7	49.2	3.5	2.9	2.3
P77509	YphE		Monomer	88.4	74.7	57.7	5.2	4.4	3.4
P77269	YphF	Periplasm	Monomer	105.5	89.1	68.8	8.0	6.7	5.2
			Complex with SecB	68.8	56.2	44.9	2.7	2.2	1.8
			Complex with TF (Tig)	75.2	63.5	49.1	3.4	2.9	2.2
P76585	YphG		Monomer	64.3	54.4	42.0	2.3	1.9	1.5
P76586	YphH		Monomer	96.1	81.2	62.7	6.4	5.4	4.2

UniProtKB A. N.	Protein name	Subcellular location	Subunit	D_0 $\left(\frac{\mu m^2}{s}\right)$			D_{cyto} $\left(\frac{\mu m^2}{s}\right)$		
				310 K	303 K	293 K	310 K	303 K	293 K
P52143	YpjA	Cell outer membrane	Monomer	58.5	49.5	38.2	1.8	1.5	1.1
			Complex with SecB	50.8	41.5	33.1	1.2	0.9	0.8
			Complex with TF (Tig)	52.7	44.5	34.4	1.3	1.1	0.8
P76612	YpjB		Monomer	112.5	95.0	73.4	9.2	7.8	6.0
P76613	YpjC		Monomer	136.9	115.7	89.4	14.4	12.1	9.4
P64432	YpjD	Cell inner membrane	Monomer	113.3	95.7	73.9	9.4	7.9	6.1
			Complex with SecB	70.4	57.5	45.9	2.9	2.4	1.9
			Complex with TF (Tig)	77.3	65.3	50.5	3.7	3.1	2.4
Q46953	YpjF		Monomer	159.0	134.4	103.8	19.8	16.7	12.9
P58095	YpjI		Monomer	172.0	145.3	112.3	23.2	19.6	15.2
P58033	YpjJ		Monomer	189.6	160.2	123.7	28.3	23.9	18.5
P52134	YpjK		Monomer	191.1	161.4	124.7	28.8	24.3	18.8
P0ADR0	YqaA	Cell inner membrane	Monomer	145.0	122.5	94.6	16.2	13.7	10.6
			Complex with SecB	74.7	61.0	48.7	3.4	2.8	2.2
			Complex with TF (Tig)	83.4	70.5	54.5	4.5	3.8	2.9
P77475	YqaB		Monomer	129.5	109.4	84.5	12.7	10.7	8.3
P0AE42	YqaE	Cell membrane	Monomer	212.7	179.7	138.8	35.5	30.0	23.1
			Complex with SecB	78.3	64.0	51.1	3.8	3.1	2.5
			Complex with TF (Tig)	89.0	75.2	58.1	5.3	4.5	3.4
P65367	YqcA		Monomer	142.5	120.4	93.0	15.7	13.2	10.2
Q46919	YqcC		Monomer	156.7	132.4	102.2	19.1	16.2	12.5
P77031	YqcE	Cell inner membrane	Monomer	94.2	79.6	61.5	6.1	5.1	4.0
			Complex with SecB	66.0	53.9	43.1	2.4	2.0	1.6
			Complex with TF (Tig)	71.4	60.3	46.6	3.0	2.5	2.0
C1P612	YqcG		Monomer	219.9	185.8	143.5	37.9	32.0	24.7
Q46807	YqeA		Monomer	107.9	91.2	70.4	8.4	7.1	5.5
Q46808	YqeB		Monomer	86.8	73.3	56.6	5.0	4.2	3.2
Q46809	YqeC		Monomer	115.1	97.2	75.1	9.7	8.2	6.3
Q46939	YqeF	Cytoplasm	Monomer	99.2	83.8	64.7	6.9	5.8	4.5
P63340	YqeG	Cell inner membrane	Monomer	95.6	80.8	62.4	6.3	5.3	4.1
			Complex with SecB	66.4	54.3	43.3	2.5	2.0	1.6

UniProtKB A. N.	Protein name	Subcellular location	Subunit	D_0 $\left(\frac{\mu m^2}{s}\right)$			D_{cyto} $\left(\frac{\mu m^2}{s}\right)$		
				310 K	303 K	293 K	310 K	303 K	293 K
			Complex with TF (Tig)	71.9	60.7	46.9	3.1	2.6	2.0
Q46941	YqeH		Monomer	121.8	102.9	79.5	11.1	9.4	7.2
Q46942	YqeI		Monomer	110.9	93.7	72.4	8.9	7.6	5.8
Q46943	YqeJ	Membrane	Monomer	134.9	114.0	88.0	13.9	11.7	9.1
			Complex with SecB	73.6	60.1	48.0	3.3	2.7	2.1
			Complex with TF (Tig)	81.9	69.2	53.4	4.3	3.6	2.8
P77136	YqeK		Monomer	142.5	120.4	93.0	15.7	13.2	10.2
C1P613	YqeL		Monomer	269.6	227.7	175.9	55.4	46.8	36.2
P67153	YqfA	Cell inner membrane	Monomer	122.7	103.7	80.1	11.3	9.5	7.3
			Complex with SecB	72.0	58.8	47.0	3.1	2.5	2.0
			Complex with TF (Tig)	79.6	67.2	51.9	4.0	3.4	2.6
P67603	YqfB		Monomer	161.1	136.1	105.1	20.3	17.2	13.3
P64562	YqfE		Monomer	185.0	156.3	120.7	26.9	22.8	17.6
C1P614	YqfG		Monomer	230.2	194.5	150.2	41.3	34.9	27.0
Q46831	YqgA	Cell membrane	Monomer	121.2	102.4	79.1	10.9	9.3	7.1
			Complex with SecB	71.8	58.6	46.8	3.0	2.5	2.0
			Complex with TF (Tig)	79.2	66.9	51.7	3.9	3.3	2.6
P64567	YqgB	Cytoplasm	Monomer	228.9	193.4	149.4	40.9	34.5	26.7
P64570	YqgC		Monomer	187.2	158.2	122.2	27.6	23.3	18.0
P46879	YqgD		Monomer	175.9	148.6	114.8	24.3	20.6	15.9
P0A8W5	YqgE		Monomer	129.7	109.6	84.7	12.8	10.8	8.3
P0A8I1	YqgF	Cytoplasm	Monomer	146.5	123.7	95.6	16.6	14.0	10.8
P67244	YqhA	Cell membrane	Monomer	135.1	114.2	88.2	13.9	11.8	9.1
			Complex with SecB	73.6	60.2	48.1	3.3	2.7	2.1
			Complex with TF (Tig)	81.9	69.2	53.5	4.3	3.6	2.8
Q46855	YqhC		Monomer	104.5	88.3	68.2	7.8	6.6	5.1
Q46856	YqhD		Monomer	98.2	83.0	64.1	6.7	5.7	4.4
			Homodimer	74.8	61.1	48.8	3.4	2.8	2.2
Q46858	YqhG		Monomer	105.6	89.2	68.9	8.0	6.7	5.2
P65298	YqhH	Cell outer membrane	Monomer	175.9	148.6	114.8	24.3	20.6	15.9
			Complex with SecB	76.9	62.8	50.2	3.6	3.0	2.4

UniProtKB A. N.	Protein name	Subcellular location	Subunit	D_0 $\left(\frac{\mu m^2}{s}\right)$			D_{cyto} $\left(\frac{\mu m^2}{s}\right)$		
				310 K	303 K	293 K	310 K	303 K	293 K
			Complex with TF (Tig)	86.8	73.3	56.6	5.0	4.2	3.2
P0A8Z7	YqiA		Monomer	127.5	107.7	83.2	12.3	10.4	8.0
P0ADU7	YqiB		Monomer	141.6	119.6	92.4	15.4	13.0	10.1
Q46868	YqiC		Monomer	164.6	139.0	107.4	21.2	17.9	13.8
P76655	YqiG	Cell outer membrane	Monomer	72.3	61.1	47.2	3.1	2.6	2.0
			Complex with SecB	58.1	47.4	37.9	1.7	1.4	1.1
			Complex with TF (Tig)	61.3	51.8	40.0	2.0	1.7	1.3
P77616	YqiH	Periplasm	Monomer	114.4	96.7	74.7	9.6	8.1	6.3
			Complex with SecB	70.6	57.7	46.1	2.9	2.4	1.9
			Complex with TF (Tig)	77.6	65.6	50.7	3.7	3.2	2.4
P76656	YqiI		Monomer	101.6	85.9	66.3	7.3	6.2	4.8
P76657	YqiJ	Cell inner membrane	Monomer	124.3	105.0	81.1	11.6	9.8	7.6
			Complex with SecB	72.2	59.0	47.1	3.1	2.5	2.0
			Complex with TF (Tig)	79.9	67.5	52.1	4.0	3.4	2.6
P77306	YqiK	Cell inner membrane	Monomer	85.1	71.9	55.5	4.7	4.0	3.1
			Complex with SecB	63.2	51.6	41.2	2.2	1.8	1.4
			Complex with TF (Tig)	67.7	57.2	44.2	2.6	2.2	1.7
P0AA63	YqjA	Cell inner membrane	Monomer	121.3	102.4	79.1	11.0	9.3	7.2
			Complex with SecB	71.8	58.6	46.8	3.0	2.5	2.0
			Complex with TF (Tig)	79.2	66.9	51.7	3.9	3.3	2.6
P42615	YqjB	Membrane	Monomer	150.5	127.1	98.2	17.6	14.9	11.5
			Complex with SecB	75.2	61.4	49.0	3.4	2.8	2.2
			Complex with TF (Tig)	84.2	71.1	54.9	4.6	3.9	3.0
P42616	YqjC		Monomer	151.6	128.1	98.9	17.9	15.1	11.7
P64581	YqjD		Monomer	165.9	140.1	108.3	21.6	18.2	14.1
P64585	YqjE	Cell inner membrane	Monomer	146.6	123.9	95.7	16.6	14.1	10.9
			Complex with SecB	74.8	61.1	48.8	3.4	2.8	2.2
			Complex with TF (Tig)	83.7	70.7	54.6	4.5	3.8	3.0
P42619	YqjF	Cell inner membrane	Monomer	150.8	127.4	98.4	17.7	14.9	11.5
			Complex with SecB	75.2	61.4	49.1	3.4	2.8	2.2
			Complex with TF (Tig)	84.2	71.1	55.0	4.6	3.9	3.0

UniProtKB A. N.	Protein name	Subcellular location	Subunit	$D_0\left(\frac{\mu m^2}{s}\right)$			$D_{\text{cyto}}\left(\frac{\mu m^2}{s}\right)$		
				310 K	303 K	293 K	310 K	303 K	293 K
P42620	YqjG		Monomer	102.9	86.9	67.1	7.5	6.3	4.9
Q46871	YqjH	Cytoplasm	Monomer	113.8	96.2	74.3	9.5	8.0	6.2
P64588	YqjI		Monomer	123.6	104.4	80.7	11.4	9.7	7.5
Q47710	YqjK		Monomer	161.6	136.5	105.5	20.4	17.3	13.3
P42913	YraH	Fimbrium	Monomer	129.6	109.5	84.6	12.7	10.7	8.3
			Complex with SecB	73.0	59.6	47.6	3.2	2.6	2.1
			Complex with TF (Tig)	80.9	68.4	52.8	4.2	3.5	2.7
P42914	YraI	Periplasm	Monomer	119.2	100.7	77.8	10.5	8.9	6.9
			Complex with SecB	71.4	58.4	46.6	3.0	2.5	2.0
			Complex with TF (Tig)	78.8	66.6	51.4	3.9	3.3	2.5
P42915	YraJ	Cell outer membrane	Monomer	71.8	60.6	46.8	3.1	2.6	2.0
			Complex with SecB	57.8	47.2	37.7	1.7	1.4	1.1
			Complex with TF (Tig)	61.0	51.5	39.8	2.0	1.7	1.3
P43319	YraK	Fimbrium	Monomer	101.7	85.9	66.3	7.3	6.2	4.8
			Complex with SecB	68.0	55.5	44.4	2.6	2.2	1.7
			Complex with TF (Tig)	74.0	62.5	48.3	3.3	2.8	2.2
P45465	YraN		Monomer	147.9	125.0	96.5	17.0	14.3	11.1
P64596	YraP	Periplasm	Monomer	131.4	111.0	85.7	13.1	11.1	8.6
			Complex with SecB	73.2	59.8	47.8	3.2	2.6	2.1
			Complex with TF (Tig)	81.3	68.7	53.0	4.2	3.6	2.7
P45468	YraQ	Cell membrane	Monomer	103.0	87.0	67.2	7.5	6.4	4.9
			Complex with SecB	68.3	55.8	44.6	2.7	2.2	1.7
			Complex with TF (Tig)	74.4	62.9	48.6	3.4	2.8	2.2
P45469	YraR		Monomer	124.0	104.8	80.9	11.5	9.7	7.5
P0A9W6	YrbA		Monomer	176.4	149.0	115.1	24.5	20.7	16.0
P45394	YrbG	Cell inner membrane	Monomer	105.9	89.5	69.1	8.0	6.8	5.2
			Complex with SecB	68.9	56.3	45.0	2.7	2.2	1.8
			Complex with TF (Tig)	75.3	63.6	49.1	3.5	2.9	2.3
P64610	YrbL		Monomer	121.7	102.8	79.4	11.1	9.3	7.2
C1P618	YrbN		Monomer	277.3	234.3	181.0	58.4	49.3	38.1
P0A9W9	YrdA		Monomer	130.8	110.5	85.4	13.0	11.0	8.5

UniProtKB A. N.	Protein name	Subcellular location	Subunit	D_0 $\left(\frac{\mu m^2}{s}\right)$			D_{cyto} $\left(\frac{\mu m^2}{s}\right)$		
				310 K	303 K	293 K	310 K	303 K	293 K
P45795	YrdB		Monomer	172.5	145.7	112.5	23.4	19.7	15.3
P45771	YrdD		Monomer	131.8	111.4	86.0	13.2	11.2	8.6
P45800	YrfF	Cell inner membrane	Monomer	76.5	64.7	49.9	3.6	3.0	2.4
			Complex with SecB	59.9	48.9	39.1	1.9	1.5	1.2
			Complex with TF (Tig)	63.6	53.7	41.5	2.2	1.9	1.4
P64636	YrfG		Monomer	119.7	101.1	78.1	10.6	9.0	6.9
P46856	YrhA		Monomer	143.7	121.4	93.7	15.9	13.5	10.4
P46857	YrhB		Monomer	168.5	142.4	110.0	22.3	18.8	14.5
P58037	YrhD		Monomer	210.4	177.7	137.3	34.7	29.4	22.7
P56256	YsaA		Monomer	138.7	117.1	90.5	14.8	12.5	9.6
Q2M7M3	YsaB	Cell membrane	Monomer	164.2	138.8	107.2	21.1	17.9	13.8
			Complex with SecB	76.2	62.2	49.7	3.6	2.9	2.3
			Complex with TF (Tig)	85.7	72.4	55.9	4.8	4.1	3.1
P56262	YsgA		Monomer	113.0	95.5	73.7	9.3	7.9	6.1
C1P620	YshB		Monomer	257.0	217.1	167.7	50.8	42.9	33.1
A5A630	YtcA	Cell membrane	Monomer	170.8	144.3	111.5	22.9	19.4	15.0
			Complex with SecB	76.6	62.6	50.0	3.6	3.0	2.4
			Complex with TF (Tig)	86.3	72.9	56.3	4.9	4.1	3.2
P39309	YtfA		Monomer	159.3	134.6	104.0	19.8	16.8	12.9
P39310	YtfB	Membrane	Monomer	123.4	104.3	80.5	11.4	9.6	7.4
			Complex with SecB	72.1	58.9	47.0	3.1	2.5	2.0
			Complex with TF (Tig)	79.7	67.3	52.0	4.0	3.4	2.6
P69506	YtfE	Cytoplasm	Monomer	120.7	102.0	78.8	10.8	9.2	7.1
			Homodimer	92.0	75.1	60.0	5.7	4.7	3.7
P39314	YtfF	Cell inner membrane	Monomer	105.3	89.0	68.7	7.9	6.7	5.2
			Complex with SecB	68.8	56.2	44.9	2.7	2.2	1.8
			Complex with TF (Tig)	75.1	63.5	49.0	3.4	2.9	2.2
P0ACN2	YtfH		Monomer	150.9	127.5	98.5	17.7	14.9	11.5
P39317	YtfI		Monomer	104.5	88.3	68.2	7.8	6.6	5.1
P39187	YtfJ	Periplasm	Monomer	130.4	110.2	85.1	12.9	10.9	8.4
			Complex with SecB	73.1	59.7	47.7	3.2	2.6	2.1

UniProtKB A. N.	Protein name	Subcellular location	Subunit	D_0 $\left(\frac{\mu m^2}{s}\right)$			D_{cyto} $\left(\frac{\mu m^2}{s}\right)$		
				310 K	303 K	293 K	310 K	303 K	293 K
			Complex with TF (Tig)	81.1	68.5	52.9	4.2	3.5	2.7
P0ADE2	YtfK		Monomer	187.6	158.5	122.4	27.7	23.4	18.1
P0AE45	YtfL	Cell inner membrane	Monomer	92.0	77.7	60.0	5.7	4.8	3.7
			Complex with SecB	65.4	53.4	42.7	2.4	1.9	1.5
			Complex with TF (Tig)	70.5	59.6	46.0	2.9	2.5	1.9
P0ADE4	YtfM		Monomer	82.9	70.1	54.1	4.4	3.7	2.9
P39321	YtfN		Monomer	61.9	52.3	40.4	2.0	1.7	1.3
P0AE48	YtfP		Monomer	156.3	132.0	102.0	19.1	16.1	12.4
P39325	YtfQ	Periplasm	Monomer	106.4	89.9	69.4	8.1	6.9	5.3
			Complex with SecB	69.0	56.4	45.0	2.7	2.2	1.8
			Complex with TF (Tig)	75.4	63.7	49.2	3.5	2.9	2.3
Q6BEX0	YtfR		Monomer	88.3	74.6	57.6	5.2	4.4	3.4
P39328	YtfT	Cell inner membrane	Monomer	104.8	88.5	68.4	7.8	6.6	5.1
			Complex with SecB	68.7	56.1	44.8	2.7	2.2	1.8
			Complex with TF (Tig)	75.0	63.3	48.9	3.4	2.9	2.2
A8DYQ1	YthA	Cell membrane	Monomer	230.4	194.7	150.4	41.4	35.0	27.0
			Complex with SecB	78.8	64.3	51.4	3.9	3.2	2.5
			Complex with TF (Tig)	89.7	75.8	58.6	5.4	4.6	3.5
Q2M5U1	YtjA	Cell membrane	Monomer	217.5	183.8	141.9	37.1	31.3	24.2
			Complex with SecB	78.5	64.1	51.2	3.8	3.1	2.5
			Complex with TF (Tig)	89.2	75.4	58.2	5.3	4.5	3.5
P76692	YzgL		Monomer	171.7	145.0	112.0	23.1	19.6	15.1
P0ADS2	ZapA	Cytoplasm	Monomer	157.6	133.1	102.8	19.4	16.4	12.7
			Homodimer	120.1	98.1	78.4	10.7	8.8	7.0
P0AF36	ZapB	Cytoplasm	Monomer	175.1	147.9	114.2	24.1	20.4	15.7
			Homodimer	133.4	109.0	87.1	13.6	11.1	8.8
P76344	ZinT	Cytoplasm<br>Periplasm	Monomer	120.9	102.1	78.9	10.9	9.2	7.1
P77173	ZipA	Cell inner membrane	Monomer	103.9	87.8	67.8	7.7	6.5	5.0
			Complex with SecB	68.5	55.9	44.7	2.7	2.2	1.8
			Complex with TF (Tig)	74.7	63.1	48.7	3.4	2.9	2.2

UniProtKB A. N.	Protein name	Subcellular location	Subunit	$D_0 \left(\frac{\mu m^2}{s}\right)$ 310 K	D_0 303 K	D_0 293 K	$D_{\text{cyto}} \left(\frac{\mu m^2}{s}\right)$ 310 K	D_{cyto} 303 K	D_{cyto} 293 K
P75757	ZitB	Cell inner membrane	Monomer	106.0	89.5	69.1	8.0	6.8	5.2
			Complex with SecB	68.9	56.3	45.0	2.7	2.2	1.8
			Complex with TF (Tig)	75.3	63.6	49.1	3.5	2.9	2.3
P37617	ZntA	Cell inner membrane	Monomer	77.6	65.5	50.6	3.7	3.2	2.4
			Complex with SecB	60.3	49.3	39.4	1.9	1.6	1.2
			Complex with TF (Tig)	64.1	54.1	41.8	2.2	1.9	1.5
P64423	ZntB	Cell inner membrane	Monomer	103.7	87.6	67.7	7.6	6.5	5.0
			Complex with SecB	68.4	55.9	44.7	2.7	2.2	1.8
			Complex with TF (Tig)	74.6	63.0	48.7	3.4	2.9	2.2
P0ACS5	ZntR		Monomer	142.9	120.7	93.2	15.7	13.3	10.3
			Homodimer	108.9	88.9	71.0	8.6	7.0	5.6
P39172	ZnuA	Periplasm	Monomer	107.1	90.4	69.9	8.2	7.0	5.4
			Complex with SecB	69.2	56.5	45.1	2.8	2.3	1.8
			Complex with TF (Tig)	75.6	63.9	49.4	3.5	3.0	2.3
P39832	ZnuB	Cell inner membrane	Monomer	115.7	97.7	75.5	9.8	8.3	6.4
			Complex with SecB	70.8	57.9	46.2	2.9	2.4	1.9
			Complex with TF (Tig)	77.9	65.8	50.9	3.8	3.2	2.5
P0A9X1	ZnuC	Cell inner membrane	Monomer	115.4	97.5	75.3	9.8	8.3	6.4
			Complex with SecB	70.8	57.8	46.2	2.9	2.4	1.9
			Complex with TF (Tig)	77.9	65.8	50.8	3.8	3.2	2.5
P0AAA9	ZraP	Periplasm	Monomer	146.4	123.7	95.5	16.6	14.0	10.8
			Complex with SecB	74.8	61.1	48.8	3.4	2.8	2.2
			Complex with TF (Tig)	83.6	70.7	54.6	4.5	3.8	3.0
P14375	ZraR	Cytoplasm	Monomer	93.0	78.6	60.7	5.9	5.0	3.8
P14377	ZraS	Cell inner membrane	Monomer	91.1	76.9	59.4	5.6	4.7	3.6
			Complex with SecB	65.1	53.2	42.5	2.3	1.9	1.5
			Complex with TF (Tig)	70.2	59.3	45.8	2.9	2.4	1.9
P0A8H3	ZupT	Cell membrane	Monomer	117.8	99.5	76.8	10.3	8.7	6.7
			Complex with SecB	71.2	58.2	46.5	3.0	2.4	1.9
			Complex with TF (Tig)	78.4	66.3	51.2	3.8	3.2	2.5
P0AC51	Zur		Monomer	133.4	112.7	87.1	13.6	11.5	8.9

UniProtKB A. N.	Protein name	Subcellular location	Subunit	D_0 $\left(\frac{\mu m^2}{s}\right)$			D_{cyto} $\left(\frac{\mu m^2}{s}\right)$		
				310 K	303 K	293 K	310 K	303 K	293 K
P0AC53	Zwf		Monomer	88.0	74.3	57.4	5.1	4.3	3.4
